# Switching Selectivity in Borylative Allyl–Allyl Cross-Coupling through Synergistic Catalysis

**DOI:** 10.1021/jacs.4c07188

**Published:** 2024-07-24

**Authors:** Nuria Vázquez-Galiñanes, Giuseppe Sciortino, Martín Piñeiro-Suárez, Balázs L. Tóth, Feliu Maseras, Martín Fañanás-Mastral

**Affiliations:** † Centro Singular de Investigación en Química Biolóxica e Materiais Moleculares (CiQUS), Universidade de Santiago de Compostela, 15782 Santiago de Compostela, Spain; ‡ Institute of Chemical Research of Catalonia (ICIQ-CERCA), The Barcelona Institute of Science and Technology, Avgda, Països Catalans 16, 43007 Tarragona, Spain

## Abstract

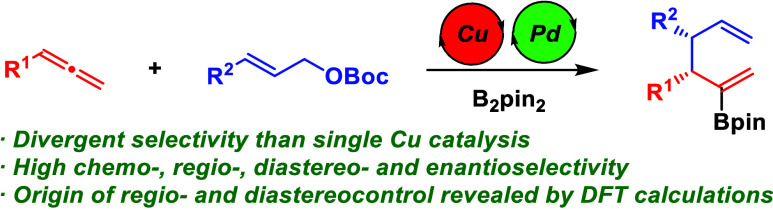

A Cu/Pd-catalyzed borylative coupling of allenes with allyl carbonates is reported. Synergistic Cu/Pd catalysis enables a divergent selectivity compared to Cu catalysis and allows for the regio-, diastereo-, and enantioselective formation of synthetically versatile chiral borylated 1,5-dienes featuring two adjacent tertiary stereocenters. DFT calculations support a closed inner-sphere S_E_2′ transmetalation between the catalytic allyl copper and allyl palladium intermediates and point at the reductive elimination of the resulting bis(allyl)Pd intermediate as the regio- and diastereo-determining step.

## Introduction

**Scheme 1:**
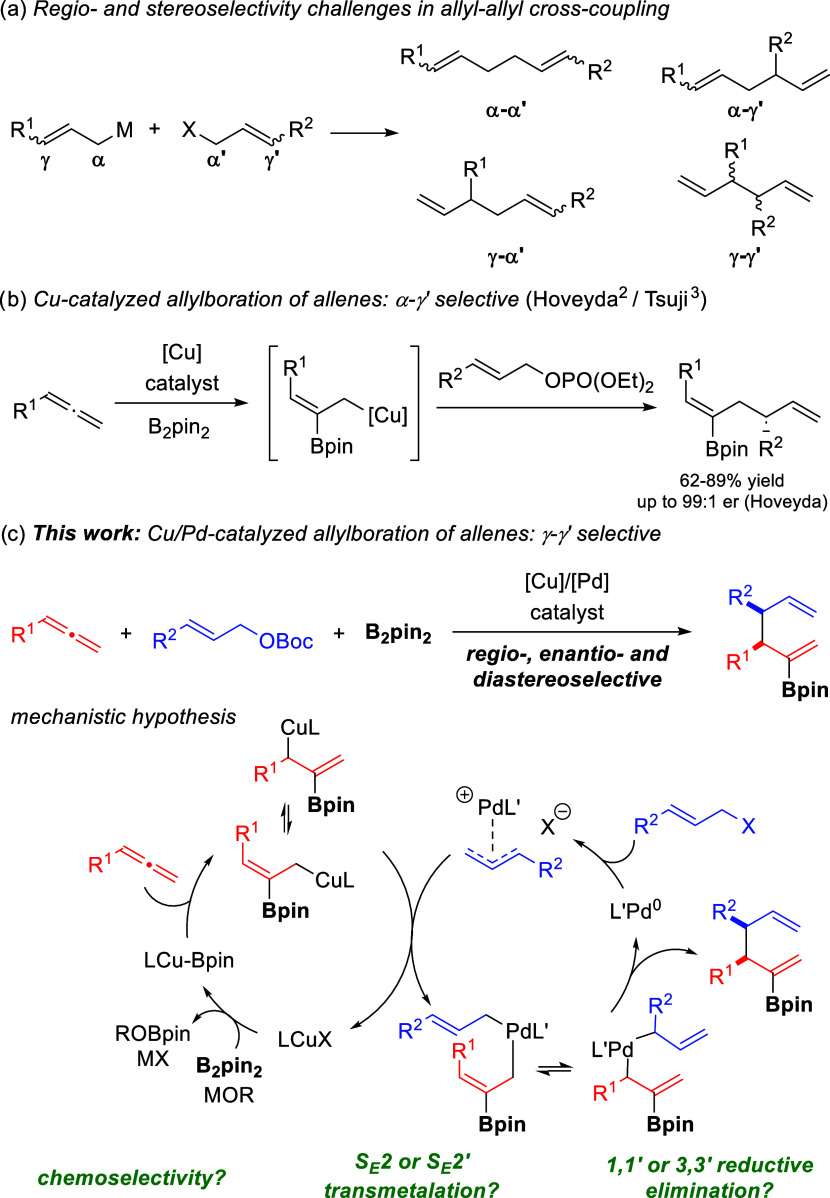
Borylative Allyl–Allyl Coupling

Carboboration of π-bonds has emerged as an efficient tool for the transformation of simple hydrocarbons into synthetically versatile organoboron-containing molecules.^1^ Compared to traditional cross-couplings that are based on the stoichiometric use of organometallic reagents, copper-catalyzed borylative couplings involve the catalytic formation of a nucleophilic organocopper intermediate by LCu-Bpin addition across the unsaturated hydrocarbon, followed by electrophilic trapping, thus resulting in concomitant C–C and C–B bond formation. An inherent challenge with such a process is the control over the regio- and the stereoselectivity. Thus, access to each isomer in a predictable manner represents a major goal in these transformations. In this context, a particularly challenging subtype of carboboration reaction is the allylboration of allenes, where an in situ-formed borylated allyl copper intermediate couples with an allylic substrate. In this case, besides the selectivity issues associated with the hydrocarbon borylcupration step, the coupling between the allylic nucleophile and the allylic electrophile imposes additional challenges since it can occur either at the α- or γ-position of each coupling partner. Therefore, formation of up to four different regioisomers is possible, having a more complex scenario when stereoisomers are also considered (Scheme 1a). Hoveyda^2^ and Tsuji^3^ independently reported a copper-catalyzed allylboration of allenes with allylic phosphates (Scheme 1b).^4^ Under a single copper catalysis regime, borylcupration of the allene generates an allyl copper intermediate, which reacts through the α-carbon with an allyl phosphate via an S_N_2′-type reaction to afford the α,γ′-coupling product with excellent regio- and stereoselectivity. However, only one regioisomer out of the possible four can be obtained from this reaction. Access to other regioisomers represents a desirable goal and would broaden the product chemical space of borylative couplings.

Cooperative catalysis offers alternative opportunities compared to single catalyst systems due to the possibility of tailoring each catalytic cycle to achieve selective and divergent product outcomes.^5,6^ On the basis of our studies on the Cu/Pd-catalyzed allylboration of alkynes,^7^ and inspired by the work of Echavarren^8^ and Morken^9^ on the Pd-catalyzed coupling of allylic electrophiles with allyl-metal reagents, we envisioned that the merge of Cu and Pd catalysis could serve as an efficient platform to achieve the desired selectivity switch in the borylative allyl–allyl cross-coupling. In our mechanistic hypothesis, the allyl copper species catalytically generated from the borylcupration of the allene would undergo a transmetalation with the allyl-Pd(II) complex generated by oxidative addition of an allyl carbonate into a Pd(0) catalyst. Controlled evolution of the resulting bis(allyl)-Pd(II) intermediate through a putative 3,3′-reductive elimination^8−10^ step would provide the borylated 1,5-diene. If successful, this strategy would result in a new synthetic tool to access borylated 1,5-dienes with different connectivity (Scheme 1c). The proposed transformation imposes several questions and challenges that should be overcome to successfully implement this idea. (1) Chemoselective activation of both unsaturated substrates by each transition metal catalyst must be achieved while avoiding direct allylic borylation pathways.^11,12^ (2) Many factors may control the regioselectivity of this transformation. After allene borylcupration, the resulting allyl copper intermediate may exist in different isomeric forms which could undergo either S_E_2 or S_E_2′ transmetalation^13,14^ with the allyl palladium complex leading to different isomeric bis(allyl)Pd(II) complexes. Potential evolution of these intermediates by either 1,1′- or 3,3′-reductive elimination pathways could lead to the formation of several isomeric products, thus resulting in a poorly selective reaction. (3) The mode and rate of transmetalation may also be crucial for the diastereoselectivity since the final relative configuration of the two carbon stereogenic centers may be influenced by the prior stereochemistry of both double bonds present in the bis(allyl)Pd intermediate.

Here, we report a Cu/Pd-catalyzed allylboration of allenes that provide borylated 1,5-dienes bearing two adjacent tertiary stereogenic centers with excellent chemo-, regio-, and diastereoselectivity, in a process that involves a γ–γ′-coupling that can also be carried out in an enantioselective manner. The synthetic utility of this new type of borylated 1,5-dienes is demonstrated with the synthesis of diverse chemical structures. In addition, we describe mechanistic studies that have led to unveil the mode of transmetalation between the key allyl copper(I) and allyl palladium(II) intermediates and the key parameters that are responsible for the high levels of selectivity.

## Results and Discussion

### Reaction Optimization and Substrate Scope

At the outset of our investigation, we selected the reaction between penta-3,4-dien-1-ylbenzene (**1**), *tert*-butyl cinnamyl carbonate (**2**), and bis(pinacolato)diboron (B_2_pin_2_) to evaluate both the feasibility and selectivity of the process (Table 1). Initial experiments, where each transition metal catalyst was separately prepared prior to the reaction (see the Supporting Information for details), already showed the high selectivity of this transformation toward the formation of branched borylated 1,5-diene **3**.

**Table 1:**
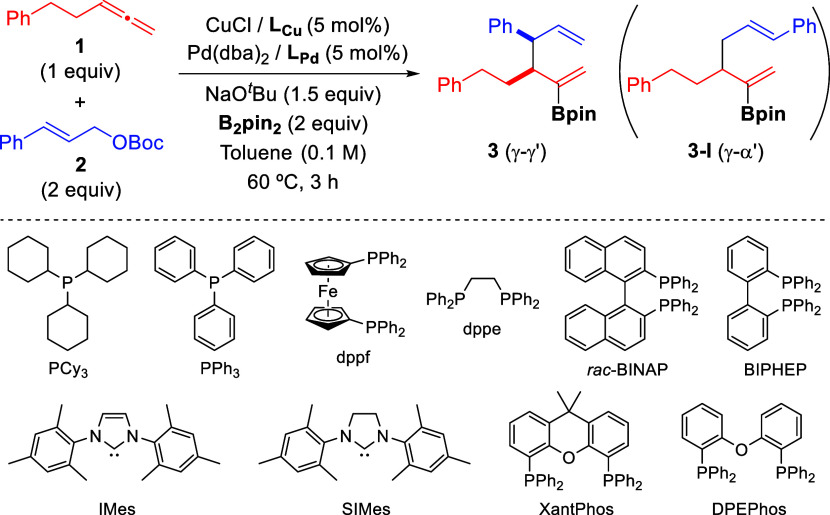
Optimization Studies

entry^a^	L_Cu_	L_Pd_	**3** yield (%)^b^	**3** dr^c^
1	PCy_3_	dppf	18	4:1
2	PPh_3_	dppf	6^d^	n.d.
3	IMes^e^	dppf	46	7:1
4	SIMes^f^	dppf	12	n.d.
5	dppe	dppf	18^g^	3:1
6	*rac*-BINAP	dppf	60	8:1
7	BIPHEP	dppf	76	14:1
8	XantPhos	dppf	77	5:1
9	DPEPhos	dppf	54	3:1
10	dppf	dppf	45	6:1
11	BIPHEP	PPh_3_^h^	40	>20:1
12	BIPHEP	BIPHEP	75	14:1
13	BIPHEP	*rac*-BINAP	63	17:1
14	BIPHEP	DPEPhos	60	6:1
15^i^	BIPHEP	BIPHEP	76	>20:1
16^j^	BIPHEP	BIPHEP	45	>20:1

a Reactions performed on a 0.4 mmol scale. Regioisomeric ratio (**3**:**3-l**) > 20:1 unless otherwise noted.

b Yield of isolated product.

c Determined by ^1^H NMR analysis.

d **3-l** was obtained in 9% yield.

e Commercially available IMesCuCl was used.

f In situ made from the imidazolium salt.

g **3-l** was obtained in 36% yield.

h 10 mol %.

i 30 °C, **2** (1.5 equiv).

j 30 °C, **2** (1.5 equiv), NaO^*t*^Bu (20 mol %). n.d. = not determined.

Screening of different copper complexes by using Pd(dba)_2_/dppf as the Pd catalyst, NaO^*t*^Bu as base, and toluene as solvent at 60 °C revealed that the use of copper catalysts based on monophosphines or NHC ligands provided the desired product **3**, albeit in low yield and with moderate diastereoselectivity (entries 1–4). A major improvement in the reaction outcome was observed when Cu catalysts derived from bisphosphines were used (entries 5–10), with CuCl/BIPHEP being the most efficient catalyst (76% yield, 14:1 dr). Keeping this Cu catalyst, we then screened different Pd catalysts. The use of PPh_3_ as the Pd ligand resulted in the formation of product **3** as a single diastereomer, although in diminished yield (entry 11).^15^

Other Pd catalysts derived from bisphosphine ligands proved to be also efficient for this transformation (entries 12–14), with BIPHEP and *rac*-BINAP giving similar results to those observed for dppf (entries 12–13 vs 7). For the sake of simplicity, we chose the bimetallic Cu/Pd catalytic system comprising an identical ligand (BIPHEP) to continue our study. This system allows for a simplified procedure in which all of the solids can be mixed together at start without prior separate formation of each transition metal catalyst.

Gratifyingly, by lowering the temperature to 30 °C, the reaction could be carried out with less allylic carbonate and furnished product **3** as a single *syn* diastereomer in 76% yield (entry 15). The relative *syn* configuration was confirmed by X-ray analysis of product **3**.^16^ Interestingly, the reaction could also be carried out with a catalytic amount of NaO^*t*^Bu, albeit with a significant decrease in the yield (entry 16). Evaluation of different bases, solvents, or allylic substrates did not lead to any further improvement (see the Supporting Information). Importantly, no formation of **3** was observed in the absence of either copper or palladium catalysts. These results highlight the key cooperative effect of both transition metal catalysts, which is crucial for reactivity but also for regio- and stereocontrol.

**Scheme 2:**
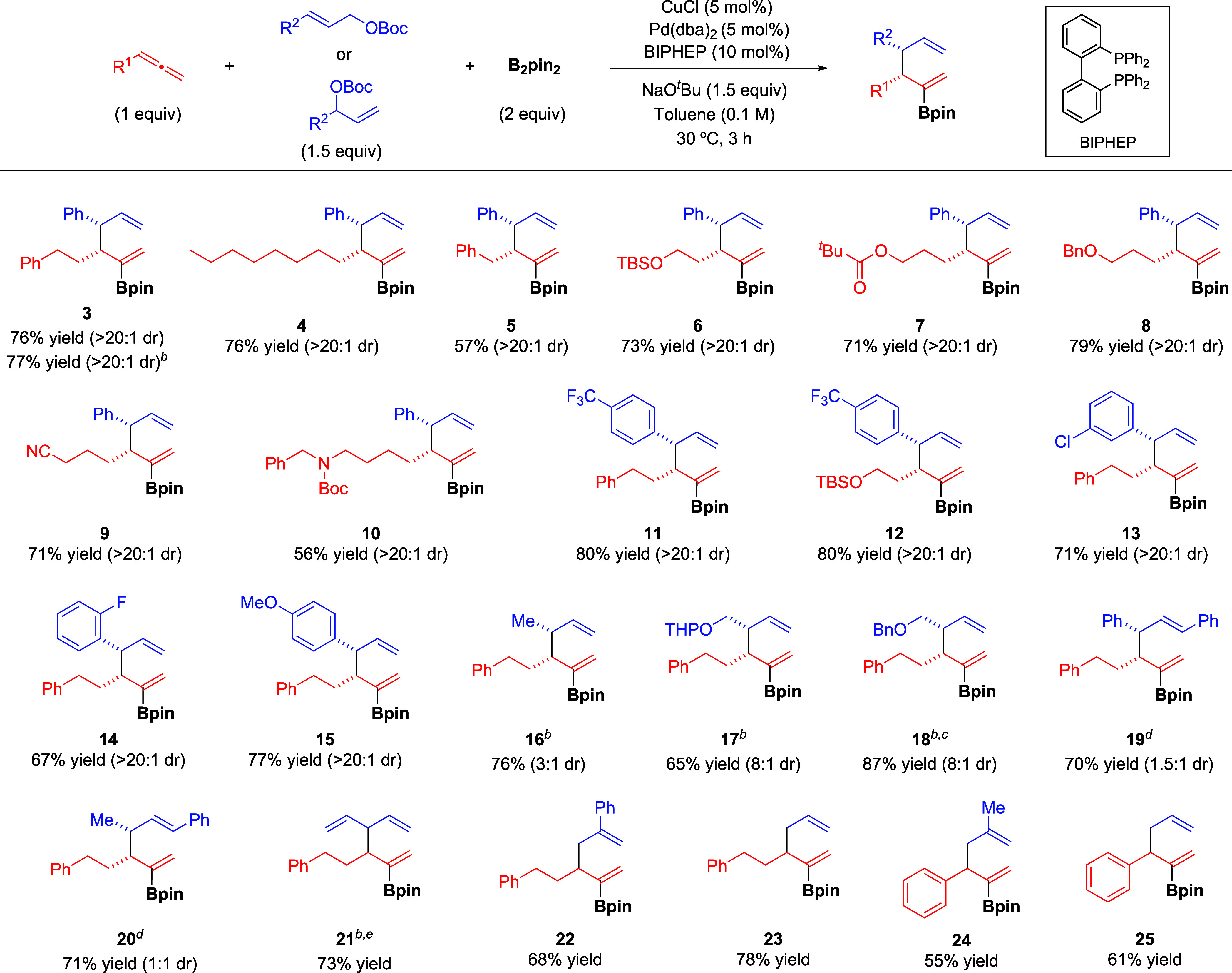
Substrate Scope of the Cu/Pd-Catalyzed Borylative Allyl–Allyl Coupling Reactions were performed on a 0.4 mmol scale under optimized conditions (Table 1, entry 15). Yield values refer to isolated products. Regioisomeric ratio (rr = γ–γ′:γ–α′) > 20:1 unless otherwise noted. Branched allylic carbonate was used. rr = 8:1. Reaction run at 60 °C. rr = 10:1.

Having established the optimized conditions, we investigated the scope of the reaction (Scheme 2). Allenes bearing aliphatic substituents proved to be efficient substrates and reacted with **2** and B_2_pin_2_ affording borylated 1,5-dienes **4**-**10** in good yields and with excellent diastereoselectivity in all cases. Functional groups such as silyl ether (**6**), ester (**7**), ether (**8**), nitrile (**9**), or carbamate (**10**) were well tolerated.

Both linear and branched allylic carbonates were equally effective for this transformation, as illustrated by the synthesis of **3**. Different cinnamyl carbonate derivatives featuring different substitution patterns proved to be similarly efficient under optimal conditions and furnished borylated dienes **11**-**15** with excellent selectivity regardless of the electronic properties and position of the substituent on the aromatic ring. Aliphatic allylic carbonates were also suitable substrates for this transformation and afforded the corresponding borylated 1,5-dienes **16**-**20** in good yield. The use of a crotyl carbonate bearing a small methyl group led to a drop in diastereoselectivity (**16**), which was restored when using bigger α-functionalized substituents (**17**-**18**). Interestingly, this borylative coupling could also be carried out successfully with internal 1,3-substituted allylic carbonates. The use of these substrates imposes additional challenges such as new regioselectivity issues and the competing formation of 1,3-dienes by β-hydride elimination.^9f^ Although the diastereoselectivity values were not high in these cases, it is remarkable that products **19** and **20** were isolated in good yields with excellent regioselectivity, even when an unsymmetrical 1,3-substituted allylic carbonate was used (**20**). The catalytic system also demonstrated an excellent ability to control the regioselectivity with a symmetrical vinyl-substituted allylic carbonate (**21**), a 2-phenyl-substituted substrate (**22**), and a simple allylic carbonate (**23**). Finally, aromatic allenes proved also efficient for this transformation (**24**, **25**).

### Enantioselective Borylative Allyl–Allyl Coupling

An enantioselective version of this borylative coupling was also investigated. Screening of different chiral bisphosphine ligands revealed (*S*)-methoxy(furyl)biphep (**L1**) as the most efficient ligand for this asymmetric reaction (Table 2). Importantly, control experiments indicated the requirement of using a chiral ligand with the same configuration at both Cu and Pd complexes to achieve high enantioselectivity since combinations of Cu/**L1** and Pd/***enant*****-L1** (and vice versa) led to nearly racemic product (see entries 14 and 15, and Supporting Information, Section 6.2).

**Table 2:**
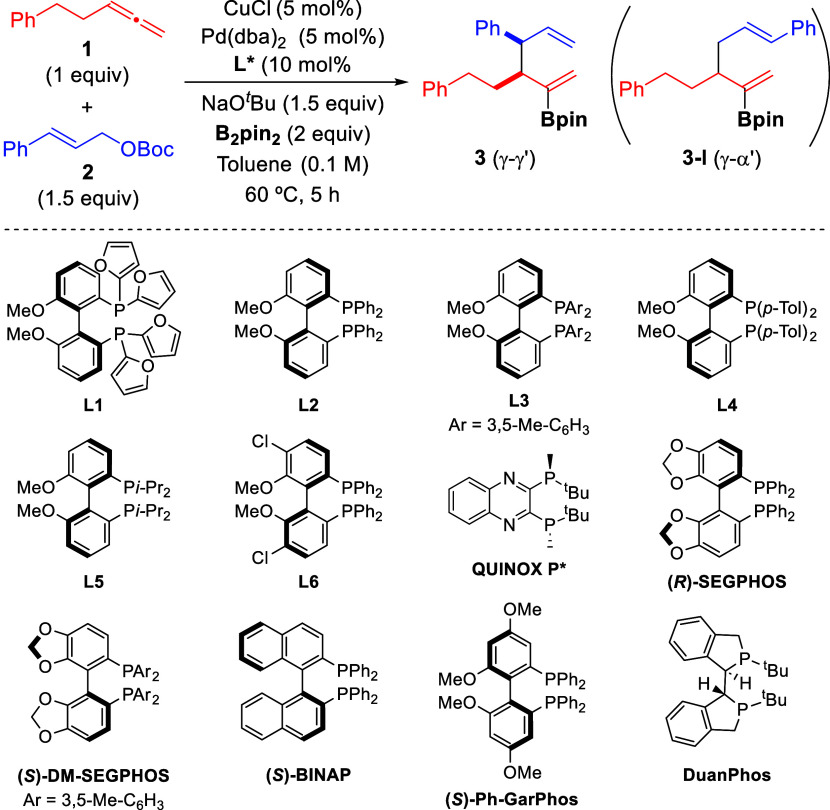
Optimization of Enantioselective Cu/Pd-Catalyzed Borylative Allyl–Allyl Coupling

entry^a^	L*	yield (%)^b^, (rr 3:3-l)	3 dr^c^	3 er^d^
1	L1	33 (>20:1)	>20:1	97:3
2	L2	59 (5:1)	12:1	8:92
3	L3	-	-	-
4	L4	32 (1:1)	11:1	11:89
5	L5	-	-	-
6	L6	43 (>20:1)	10:1	21:79
7	Quinox P*	-	-	-
8	(*R*)-SegPhos	53 (2:1)	11:1	9:91
9	(*S*)-DM-SegPhos	37 (1:5)	7:1	n.d.
10	(*S*)-BINAP	49 (4:1)	13:1	83:17
11	(*S*)-Ph-GarPhos	50 (1:1.5)	9:1	93:7
12	DuanPhos	-	-	-
13^e^	L1	59 (>20:1)	>20:1	98.5:1.5
14	Cu/(*S*)-L1 + Pd/(*R*)-L1^f^	35 (>20:1)	>20:1	62:38
15	Cu/(*R*)-L1 + Pd/(*S*)-L1^f^	32 (>20:1)	>20:1	56:44

a Reactions performed on a 0.2 mmol scale.

b Yield of isolated product.

c Determined by ^1^H NMR analysis.

d Determined by either SFC analysis or HPLC analysis of the oxidized product.

e Reaction run at 30 °C during 18 h.

f Each transition metal complex (5 mol %) was performed separately prior to the reaction. n.d. = not determined.

**Scheme 3:**
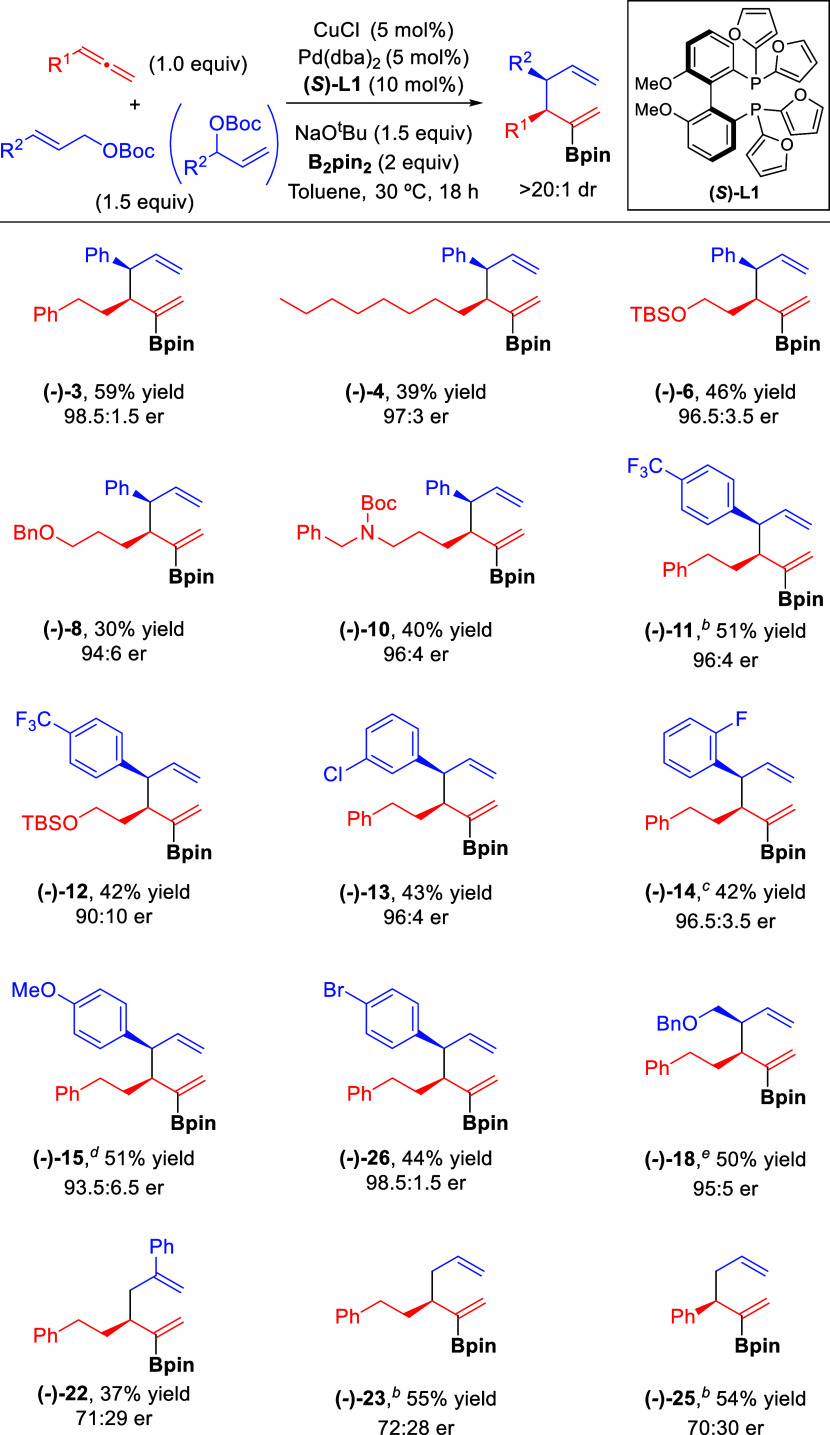
Enantioselective Cu/Pd-Catalyzed Borylative Allyl–Allyl Coupling Reaction run at 60 °C during 5 h. dr = 5:1, rr = 5:1. rr = 3:1. Branched allylic carbonate was used. Reactions were performed on a 0.4 mmol scale. Yield values refer to isolated products. Diastereomeric ratio (dr) and regioisomeric ratio (rr = γ–γ′:γ–α′) > 20:1 unless otherwise noted.

The Cu/Pd/**L1** catalytic system proved to be a little bit less reactive than the BIPHEP-based system and required longer times or higher temperatures. Nevertheless, it provided a range of branched borylated 1,5-dienes with high levels of regio-, diastereo-, and enantioselectivity (Scheme 3). The combination of several allenes with cinnamyl carbonate derivatives bearing different types of substitution pattern proved to be successful for this enantioselective borylative coupling (90:10–98.5:1.5 er). A branched racemic allylic carbonate featuring an aliphatic substituent was also efficient, as illustrated with the synthesis of **(−)-18** that highlights the enantioconvergent nature of this transformation. Simple and 2-substituted allylic carbonates also underwent the reaction catalyzed by the Cu/Pd/**L1** system, although the corresponding products were obtained with lower enantioselectivity. Absolute stereochemistry of the products was determined by X-ray diffraction analysis of the ketone product **(−)-30** resulting from the oxidation of product **(−)-11** (see the Supporting Information for details).^16^

### Synthetic Modifications

**Scheme 4:**
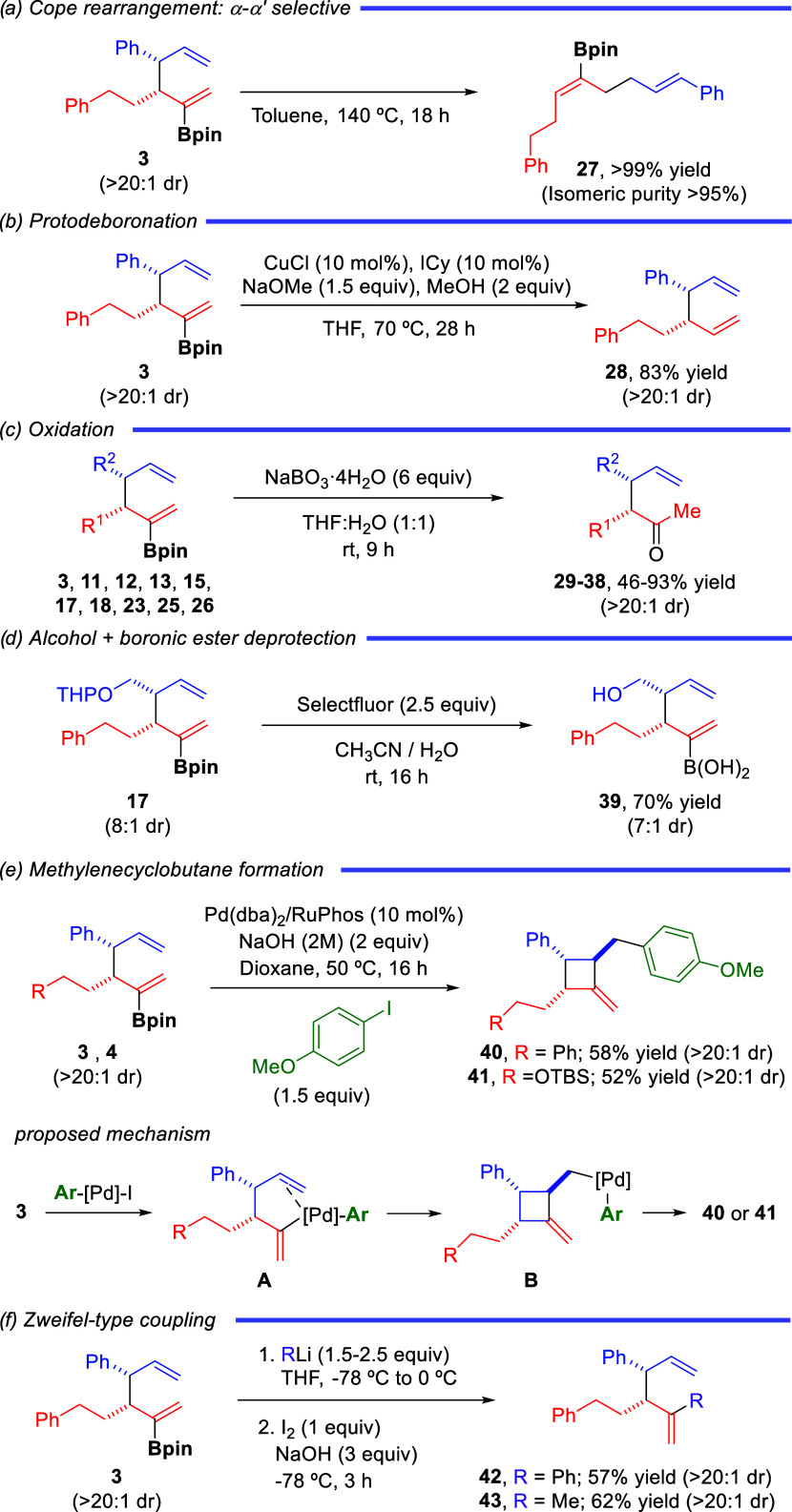
Synthetic Modifications

The products obtained from the Cu/Pd-catalyzed allylboration of allenes proved to be versatile compounds for the stereoselective synthesis of a range of different structures. Borylated 1,5-diene **3** underwent efficient thermal Cope rearrangement to furnish linear borylated diene **27** as a single *Z*,*E*-isomer in excellent yield (Scheme 4a). This transformation is relevant since it represents an alternative to access the α,α′-isomer of the borylative allyl–allyl coupling. Protodeboronation of **3** afforded product **28** as a pure *syn* 1,5-diene (Scheme 4b). Notably, this transformation allows access to branched 1,5-dienes with opposite relative configuration to the *anti*-1,5-dienes obtained by the Pd-catalyzed cross-coupling of γ-substituted allylboronates with allylic chlorides described by Morken and co-workers.^9c^

Diastereoselective synthesis of several branched methyl ketones **29**-**38** was also possible by treatment of the corresponding borylated 1,5-dienes with sodium perborate (Scheme 4c). Interestingly, concomitant deprotection of the THP group and conversion of the boronic ester into the corresponding boronic acid **39** could be accomplished by the treatment of **17** with Selectfluor (Scheme 4d). A remarkable observation was made when we attempted to carry out Suzuki-Miyaura cross-coupling with compounds **3** and **4** (Scheme 4e). When these borylated 1,5-dienes were treated with 4-iodoanisole and an aqueous solution of sodium hydroxide in 1,4-dioxane using Pd(dba)_2_/RuPhos as a catalyst, no tractable amount of the Suzuki-Miyaura cross-coupled product was observed. Instead, methylenecyclobutanes **40** and **41** were obtained in 58% and 52% yields, respectively, as single diastereomers. This transformation is proposed to occur via a mechanism in which intermediate **A**, resulting from transmetalation between the boronic ester and the aryl-Pd(II) complex generated by oxidative addition of the aryl iodide to the Pd(0) catalyst, would prefer to undergo olefin insertion instead of the expected reductive elimination. This intramolecular olefin insertion would take place in a regio- and stereoselective manner to afford *exo*-adduct **B** that gives rise to the cyclobutane product by reductive elimination. Finally, rather than via Suzuki-Miyaura coupling, simple C–B to C–C transformation could be achieved by means of a Zweifel-type coupling. By using this strategy, compound **3** was coupled with PhLi and MeLi to access products **42** and **43** in good yields with complete stereochemical retention (Scheme 4f).

### DFT Calculations

**Scheme 5:**
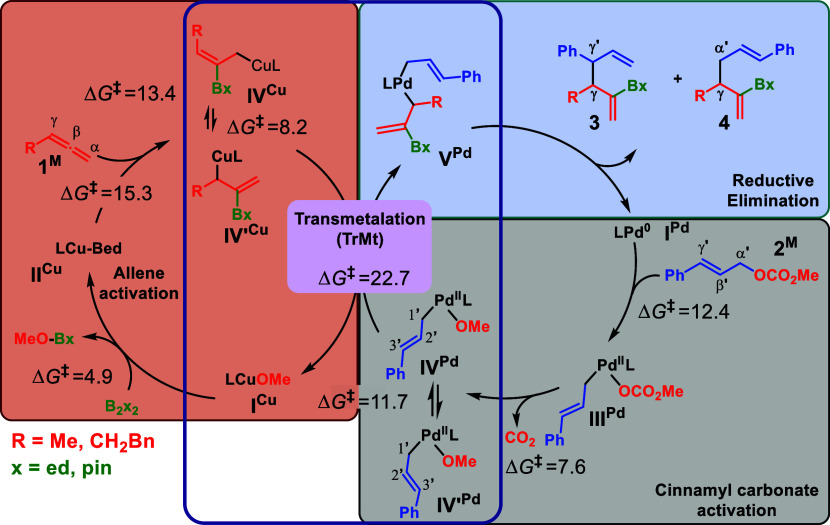
General Overview of the Reaction Mechanism with Indication of the Main Steps and Computed Free Energies in kcal·mol^–1^

The remarkable regio- and diastereoselectivity of the overall reaction led us to undertake a full DFT exploration of the molecular factors governing it. It is important to emphasize that our DFT analysis was entirely focused on the nonenantioselective aspects of the process. This targeted approach allows us to provide in-depth insights into how the reaction achieves its regio- and diastereoselectivity. We have recently shown how DFT modeling is able to identify the key factors governing multimetallic cooperative processes.^17^ Calculations were carried out with the B3LYP-D3 functional in a toluene implicit solvent. Full computational details are supplied in the Supporting Information. A collection of computational results is available in the ioChem-BD repository and can be accessed via: https://doi.org/10.19061/iochem-bd-1-334.^18^ The general mechanistic analysis was carried out using the full Cu/Pd/BIPHEP catalytic system and a slightly simplified set of reactants: 1,2-butadiene (**1**^**M**^), methyl cinnamyl carbonate (**2**^**M**^), and bis(1,2-ethanediolate)diboron (B_2_ed_2_). This small chemical simplification from the experimental system allows a significant reduction of computational time (176 vs 210 atoms), which is especially relevant for the study of the bimetallic intermediates. For the key selectivity points (see below), calculations of the full experimental system were carried out. The overall reaction mechanism is presented in Scheme 5. It can be divided into four blocks: (i) allene borylcupration; (ii) activation of the cinnamyl carbonate by the palladium catalyst; (iii) Cu–Pd transmetalation (TrMt), with transfer of the borylated allyl group from copper to palladium; and (iv) reductive elimination from bis(allyl)palladium(II) intermediate releasing the diene product.

The allene activation cycle (Scheme 5, brown block) starts with the reaction between the diboron compound and the copper alkoxide, leading to boryl-complex **II**^**Cu**^. This intermediate then coordinates the incoming allene prior to the insertion step (see the Supporting Information, Figure S1). In line with previous reports,^19,20^ allene borylcupration is highly exergonic and can occur at both C=C bonds with moderate free energy barriers for the formation of both linear **IV**^**Cu**^ (Δ*G*^‡^ = 15.3 kcal·mol^–1^) and branched **IV’**^**Cu**^ (Δ*G*^‡^ = 13.4 kcal·mol^–1^).^20c^ Moreover, these intermediates can easily isomerize to different allyl-Cu species featuring different coordination modes (Scheme S1). The most representative ones (**IV**^**Cu**^ and **IV’**^**Cu**^) are shown in Scheme 5. Isomerization between these two allyl species features a rather low energy barrier of 8.2 kcal·mol^–1^. As this isomerization is relevant to the whole selectivity outcome (vide infra), we confirmed the low barrier on additional calculations for the real system, i.e., substrates **1** and B_2_pin_2_, obtaining an activation energy barrier of 8.9 kcal·mol^–1^ (Scheme S2).

The activation of the cinnamyl carbonate by Pd cocatalyst (Scheme 5, gray block) proceeds via an oxidative addition (OA) and decarboxylation pathway. The highest energy barrier of 12.4 kcal·mol^–1^ corresponds to the OA. The key reactive allyl-Pd intermediate **IV**^**Pd**^ displays two lowest-energy conformational isomers accessible through rotation along the C1′–C2′ bond of the cinnamyl moiety with a relative energy barrier of 11.7 kcal·mol^–1^ (Figure S2). It is important to note here that along the activation of both the allene and the allylic substrate, neither regio- nor stereoselection is predicted because different isomers of **IV**^**Cu**^ and **IV’**^**Pd**^ are accessible through affordable energy barriers.

**Figure 1:**
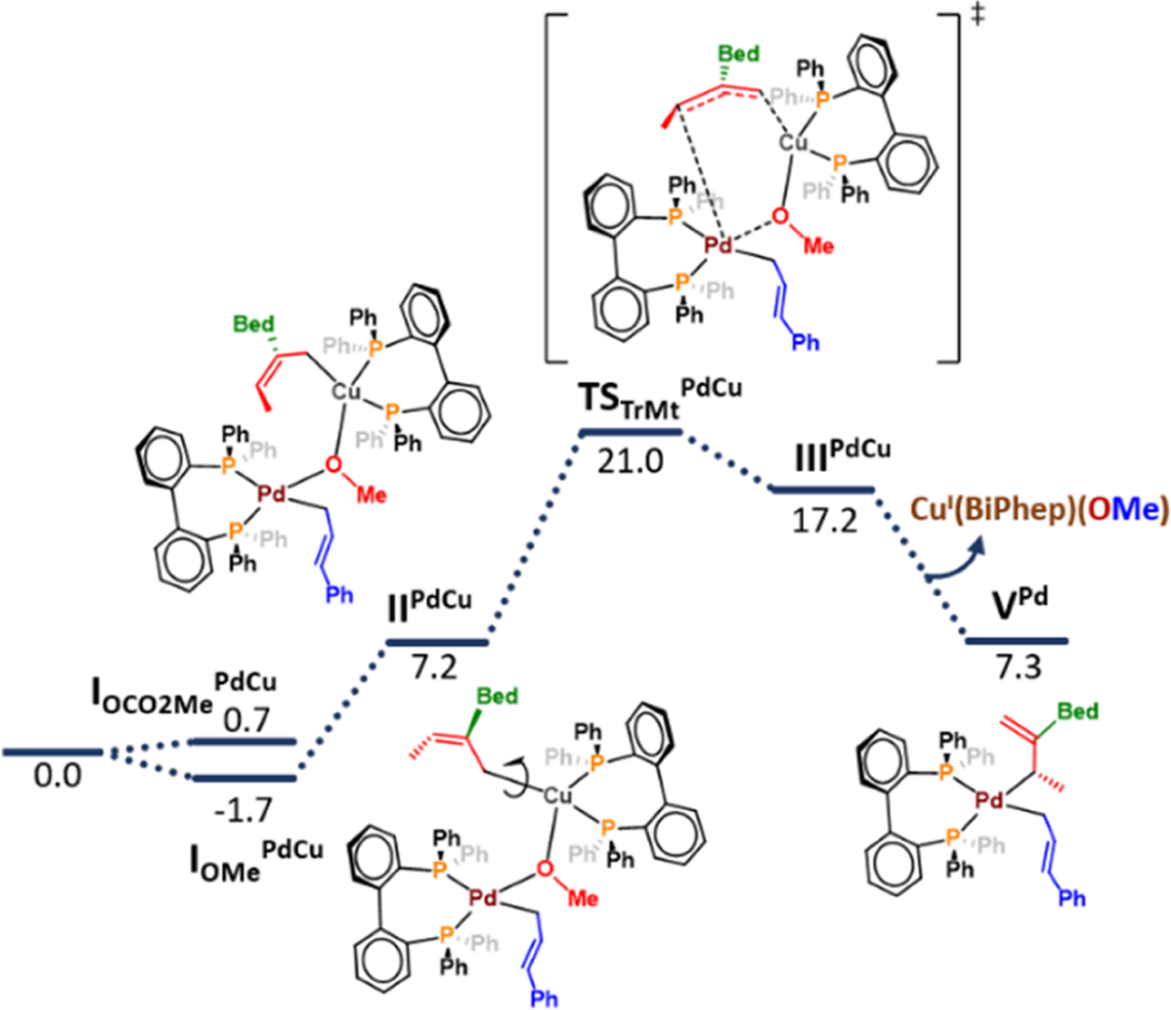
DFT computed (B3LYP-D3 in toluene) free energy profile for the Cu–Pd transmetalation process. Gibbs energies are shown in kcal·mol^–1^, taking as zero-point energy the separated activated cocatalysts **IV**^**Cu**^ and **IV**^**Pd**^.

Next, we moved our attention to the TrMt process (Scheme 5, central block). The first step is the aggregation of both cocatalysts. Formation of the Pd–Cu dinuclear species can potentially occur either from Pd precursor **III**^**Pd**^ or **IV**^**Pd**^, albeit the alkoxo-bridged complex between **IV**^**Cu**^ and **IV**^**Pd**^ (**I**_**OMe**_^**PdCu**^) resulted in the only species able to promote TrMt (Figure 1). In fact, all of the attempts to find TrMt from **I**_**OCO2Me**_^**PdCu**^ failed or featured higher energies. After the formation of **I**_**OMe**_^**PdCu**^, conformational change to **II**_**OMe**_^**PdCu**^ is required in order to reach a pro-reactive conformation leading to the key transition state **TS**_**TrMt**_. **TS**_**TrMt**_ corresponds to a closed S_E_2′ mechanism, featuring a 6-membered ring where the alkoxy group and the borylated allyl fragment are exchanged between the metal centers.

**Figure 2:**
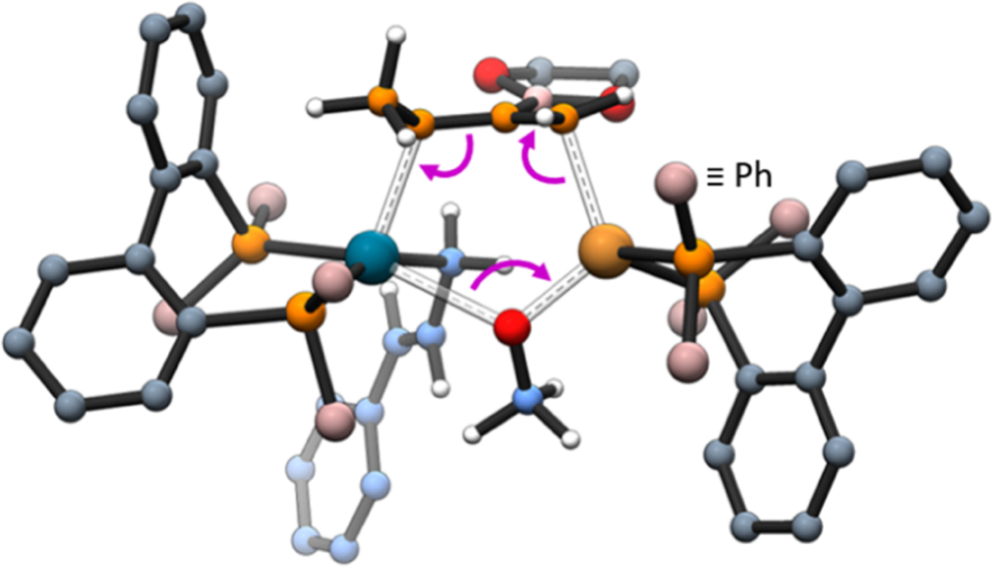
Molecular structure of the computed most stable transition state for the TrMt step giving rise to bis(ally)Pd intermediate **V**^**Pd**^. Bond breaking and forming are shown through pink, curly arrows.

A conformational analysis of this TS was carried out, and up to five different conformations along with their enantiomeric forms were computed (Figure S4). The most stable one features an activation energy of 22.7 kcal·mol^–1^, and it is shown in Figure 2. It is important to note that all of the attempts to find alternative transmetalation pathways from **IV’**^**Cu**^ or involving a S_E_2 mechanism from **IV**^**Cu**^ failed or featured higher energies.

**Figure 3:**
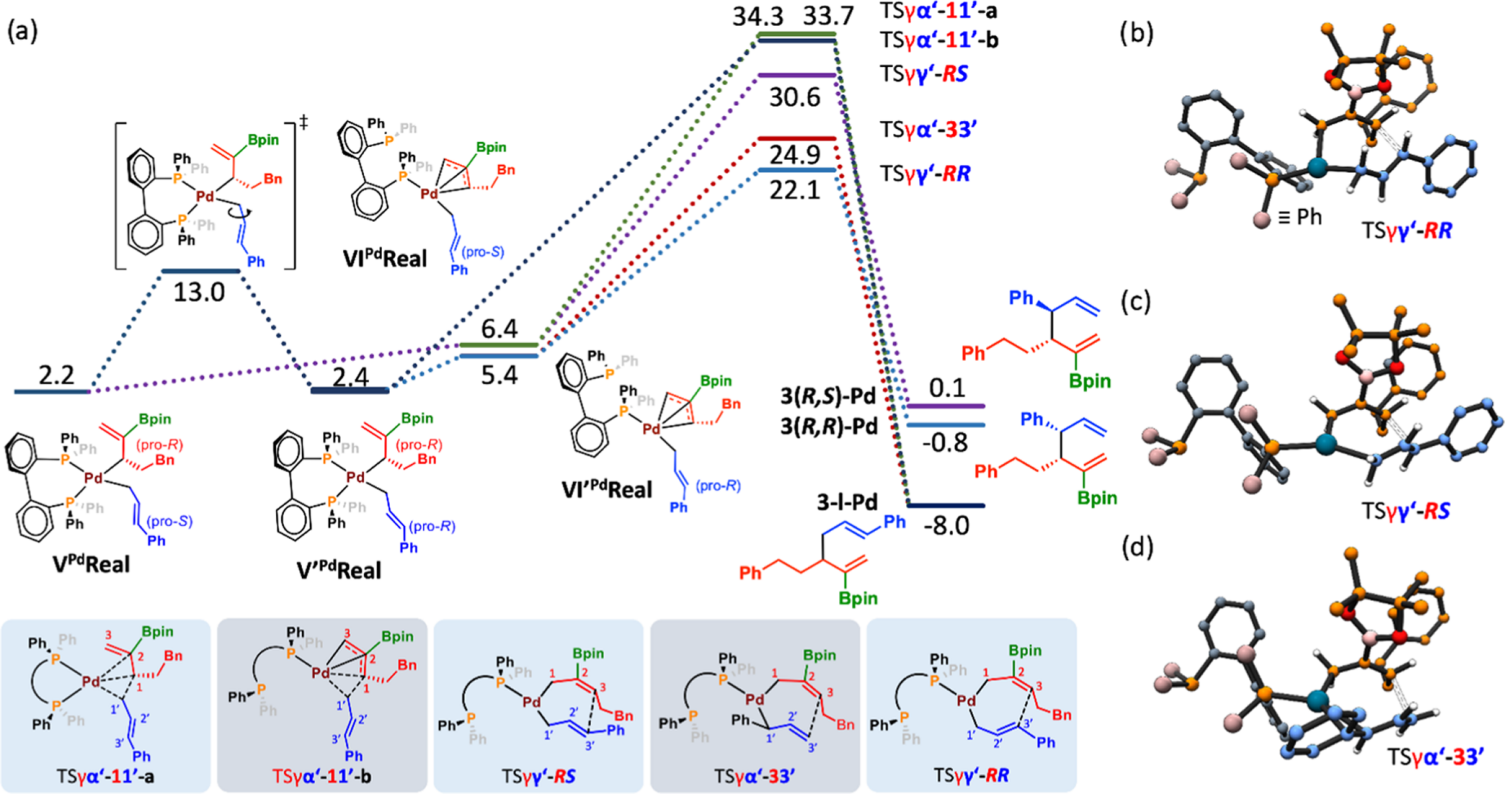
(a) DFT computed (B3LYP-D3 in toluene) free energy profile for the reductive elimination over the bis(allyl)Pd derived from the real substrates (**1**, B_2_(pin)_2_ and **2**), along with the corresponding transition state structures (bottom) and product structures. The numbers are relative Gibbs energies in kcal·mol^–1^, referred to as **I**_**OMe**_^**PdCu**^**Real** (see the Supporting Information, Figure S5). Computed molecular structures of (b) **TSγ,γ′-*****RR***, (c) **TSγ,γ′-*****RS***, and (d) **TSγ,α′-3,3′**. The last final Gibbs energy values refer to the Pd-coordinated products.

Once the bis(allyl)Pd complex **V**^**Pd**^ is formed, the reaction is ready for the final step, reductive elimination (RE) (Scheme 5, blue block). This is the step where the new C–C bond is formed, and when both the regioselectivity and the diastereoselectivity of the product are decided. We examined a variety of possible isomeric forms for this transition state, and because of its relevance, we used in these calculations the real substrates **1**, **2**, and B_2_pin_2_ (Figure 3). Importantly, in analogy with **IV**^**Pd**^ (see Figure S2), two possible conformers regarding the cinnamyl moiety are accessible in **V**^**Pd**^ through rotation along its C1′–C2′ bond with an affordable activation energy of 10.8 kcal·mol^–1^. Thus, both the *Re* and *Si* faces of C3′ are accessible for the formation of the C–C bond. In sharp contrast, pro-R* to pro-S* isomerization of the borylated allyl fragment is sterically hindered (see Supporting Information, Figure S7) indicating that the transmetalation step defines itself the chiral configuration at C3.^21^ It was found that prior to reductive elimination, isomerization from **V**^**Pd**^ to **VI**^**Pd**^ which features a κ^1^-BIPHEP coordination and a η^3^-borylated allyl fragment is necessary to achieve a 3,3′-reductive elimination. Pathways involving 1,1′-reductive elimination either from κ^2^-coordinated complex **V**^**Pd**^ (Figure 3a, dark blue pathway) or from κ^1^-complex **VI’**^**Pd**^ (Figure 3a, green pathway) led to energy barriers higher than 30 kcal·mol^–1^ and thus were discarded. The regioselectivity is then decided by the arrangement of the two interacting allyl groups through a 3,3′-reductive elimination. This step can, in principle, afford both γ–γ′ or γ–α′ coupling products. We found that the lowest energy transition state corresponds to the formation of the γ–γ′ coupling product from intermediate **VI**^**Pd**^ through transition state **TSγ,γ′-*****RR*** (Figure 3a, light blue pathway, and Figure 3b). The most favored transition state for the formation of the γ–α′ coupling product involves isomerization to a branched cinnamyl fragment and 3,3′-reductive elimination through transition state **TSγ,α′-3,3′** (Figure 3a, red pathway, and Figure 3d) that is 2.8 kcal·mol^–1^ higher, thus being not competitive. As far as the diastereoselectivity is concerned, the R*,R* product is favored by 8.5 kcal·mol^–1^ with respect to the R*,S* isomer, which forms through **TSγ,γ′-*****RS*** (Figure 3a, purple pathway and Figure 3c). These results perfectly match the observed experimental regioselectivity (γ–γ′ product) and diastereoselectivity (R*,R* diastereomer) and could be reached only after the systematic exploration of isomeric forms and the conformations of the different intermediates and transition states.

To further validate our model and explore factors influencing diastereoselectivity, we analyzed the RE step for the synthesis of product **16** that features diminished diastereoselectivity (3:1 dr, see Scheme 2). Replacement of the bulkier phenyl ring by a methyl group (i.e., use of 3-buten-2-yl carbonate instead of cinnamyl carbonate) has a 2-fold effect: it reduces steric clashes with the sizable Bpin unit in transition state **TSγγ′-*RS*** and, in a minor extent, removes favorable π–π stacking interactions in transition state **TSγγ′-*RR***. This dual effect significantly reduces the energy span between both transition states (from 8.5 to 2.5 kcal·mol^–1^), thus explaining the observed decrease in selectivity (for further details, see Supporting Information, Figure S6).

## Conclusions

In conclusion, we have disclosed a regio-, diastereo-, and enantioselective borylative allyl–allyl cross-coupling between allenes and allylic carbonates that provides chiral borylated 1,5-dienes. This has been achieved by employing synergistic copper/palladium catalysis that enables a divergent selectivity outcome than the one observed under single copper catalysis regime.^2−4^ Remarkable features of the method are the formation of the γ–γ′ coupling product with excellent regioselectivity, the *syn*-selective formation of two adjacent tertiary stereocenters, the possibility of turning the reaction enantioselective by using a chiral bisphosphine ligand, and the synthetic versatility of this new type of borylated 1,5-dienes. Intrinsic mechanistic features were characterized by DFT calculations. The key transmetalation step follows a closed inner-sphere S_E_2′ pathway that establishes stereoselectivity at the Cγ stereogenic center of the borylated allyl fragment. The regio- and diastereoselectivity are established through a 3,3′-reductive elimination, this process being the regio- and diastereo-determining step.

## Supporting Information Available

List of starting materials, optimization studies, experimental procedures and compound characterization data, computational details, Cartesian coordinates, imaginary frequencies, and absolute energies in hartrees for all optimized geometries (PDF)
