# Supplementary material for: Switching Selectivity in Borylative Allyl–Allyl Cross-Coupling through Synergistic Catalysis
Source: J Am Chem Soc. 2024 Jul 24;146(31):21977–88. doi: 10.1021/jacs.4c07188 (PMC11311230; doi:10.1021/jacs.4c07188)
Supplement: Supplementary file 1 — ja4c07188_si_001.pdf [file ja4c07188_si_001.pdf]

Supporting information

Switching Selectivity in Borylative Allyl-Allyl Cross-Coupling through Synergistic Catalysis

Nuria Vázquez-Galiñanes,[a] Giuseppe Sciortino,[b] Martín Piñeiro-Suárez,[a] Balázs L. Tóth,[a] Feliu Maseras*[b] and Martín Fañanás-Mastral*[a]

[a] Centro Singular de Investigación en Química Biolóxica e Materiais Moleculares (CiQUS), Universidade de Santiago de Compostela, Jenaro de la Fuente s/n, 15782 Santiago de Compostela (Spain)

Correspondence to: martin.fananas@usc.es

[b] Institute of Chemical Research of Catalonia (ICIQ-CERCA), The Barcelona Institute of Science and Technology, Av. Països Catalans 16, 43007 Tarragona (Spain)

Correspondence to: fmaseras@iciq.es

Table of contents

1. General methods ... 3

2. List of starting materials ... 4

3. Optimization studies ... 9

- **3.1 Screening of L_{Cu}** ... 9
- **3.2 Screening of L_{Pd}** ... 10
- **3.3 Cooperative catalysis test controls** ... 10
- **3.4 Screening of temperatures and stoichiometry** ... 10
- **3.5 Screening of bases** ... 11
- **3.6 Screening of solvents** ... 11
- **3.7 Screening of leaving groups** ... 11

4. General procedure for the Cu/Pd-catalyzed borylative allyl-allyl coupling (General Procedure A) ... 12

5. Compound characterization ... 12

6. Enantioselective Cu/Pd-catalyzed borylative allyl-allyl coupling ... 20

- **6.1 Screening of chiral ligands** ... 20
- **6.2 Experiments with different combinations of Cu and Pd complexes ($L_{Cu} \neq L_{Pd}$)** ... 21
- **6.3 General procedure for the enantioselective Cu/Pd-catalyzed borylative allyl-allyl coupling (General Procedure B)** ... 21
- **6.4 Characterization of enantioenriched compounds** ... 22

7. Derivatization of products ... 32

- **7.1 Cope rearrangement** ... 32
- **7.2 Protodeboronation** ... 32
- **7.3 Oxidation (General Procedure C)** ... 33
- **7.4 Alcohol + Boronic ester deprotection** ... 37
- **7.5 Methylene cyclobutane formation (General procedure D)** ... 37
- **7.6 Zweifel-type coupling (General procedure E)** ... 38

8. NMR spectra ... 40

9. X-ray diffraction analysis data for product 3 ... 87

10. X-ray diffraction analysis data for product (-)-30 ... 88

11. DFT calculations ... 89

- **11.1 Computational Details** ... 89
- **11.2 Gibbs Energy Profiles** ... 89
  - 11.2.1 Copper cycle ... 89
  - 11.2.2 Palladium cycle ... 90
  - 11.2.3 Copper-palladium cooperative cycle ... 91
  - 11.2.4 Calculations with real substrates ... 93
- **11.3 Cartesian coordinates and absolute energies of the optimized structures** ... 97

12. References ... 227

1. General methods

- All reactions were performed under argon atmosphere using oven dried glassware and using standard Schlenk techniques. Solvents were dried using an MBraun SPS 800 system. All chemicals and copper complexes were purchased from Acros Organics Ltd., Aldrich Chemical Co. Ltd., Alfa Aesar, Apollo, Strem Chemicals Inc., Fluorochem Ltd. or TCI Europe N.V. chemical companies and used without further purification, unless otherwise noted.

- Analytical thin layer chromatography was carried out on silica-coated aluminum plates (silica gel 60 F254 Merck) and components were visualized by UV light, I_2 and $KMnO_4$ staining. Flash column chromatography was performed on silica gel 60 (Merck, 230-400 mesh) without previous deactivation, unless otherwise stated.

- GC-MS analyses were performed in an Agilent instrument GC-6890N equipped with Chemical Ionization (CI) MS-5973 detector.

- High Resolution Mass spectrometry was carried out on a Bruker microTOF spectrometer using APCI. For halogenated compounds the molecular ion peak containing the ^{35}Cl and the ^{79}Br isotopes was considered for HRMS analysis.

- ^{1}H- and ^{13}C-NMR experiments were carried out using Varian Inova 500MHz or Mercury 300MHz NMR spectrometers. Chemical shift values are reported in ppm with the solvent resonance as the internal standard ($CHCl_3$: δ 7.26 for ^{1}H, δ 77.16 for ^{13}C). Coupling constants *J* are given in Hertz (Hz). Multiplicities are reported as follows: s = singlet, d = doublet, t=triplet, q=quartet, p=pentet, m=multiplet or as a combination of them.

- Because of quadrupolar relaxation, in all cases the carbon directly attached to the boron atom was not detected by ^{13}C NMR technique.

- In order to preclude side hydroboration reactions, commercial alkynes and B_2pin_2 were dried over Na_2SO_4 prior to being used.

2. List of starting materials

Allenes **1,**[1] **44,**[1] **45,**[1] **46,**[2] **47,**[3] **48,**[4] **49,**[5] **50,**[6] **51** [7] and allylic carbonates **2,**[8] **2',**[9] **52,**[10] **53,**[11] **54,**[12] **55,**[13] **56,**[11] **58,**[9] **57,**[14] **59,**[15] **62,**[16] **63,**[17] **64,**[18] **65**[19] were prepared according to reported procedures.

- **Allenes**

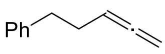

1

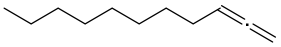

44

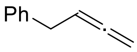

45

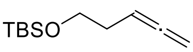

46

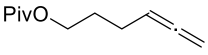

47

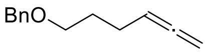

48

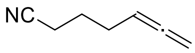

49

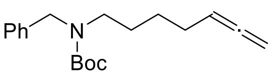

50

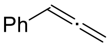

51

- **Allyl carbonates**

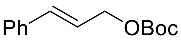

2

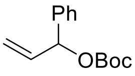

2'

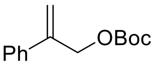

52

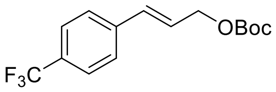

53

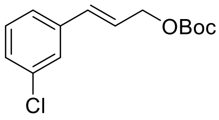

54

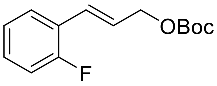

55

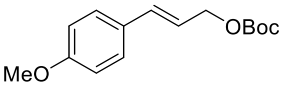

56

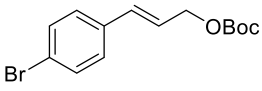

57

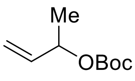

58

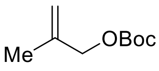

59

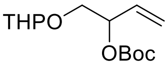

60

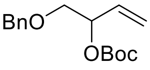

61

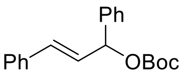

62

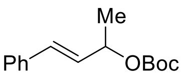

63

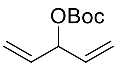

64

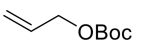

65

- **Synthesis of allylic carbonates 60 and 61**

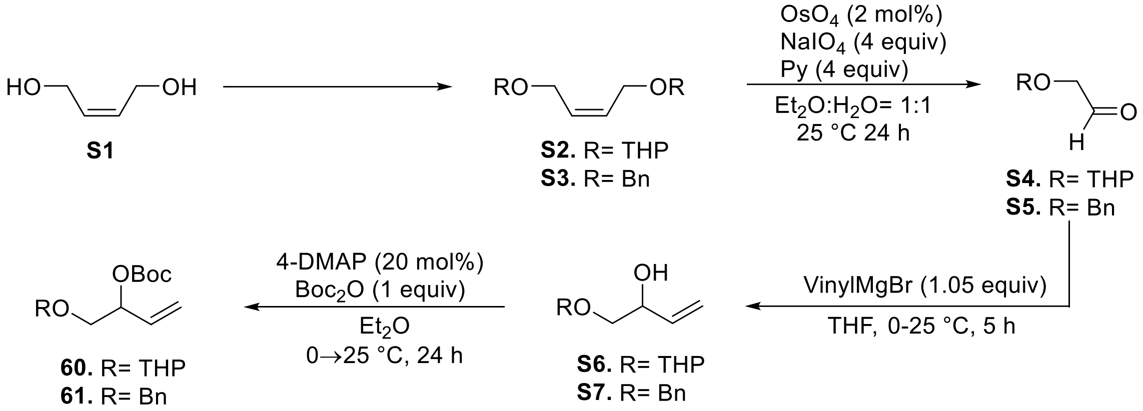

- **First step**

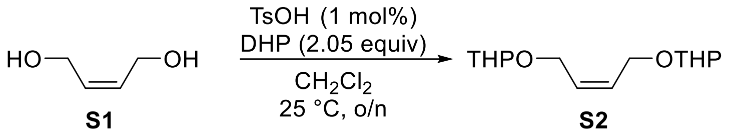

A 100 ml flame-dried round-bottom flask was equipped with a magnetic stirring bar and charged with *p*-toluenesulfonic acid monohydrate (0.01 equiv, 0.2 mmol). Then the flask was evacuated and backfilled with argon gas. Dichloromethane (60 ml) was added as solvent and (*Z*)-but-2-ene-1,4-diol (**S1**, 1 equiv, 20 mmol) and 3,4-dihydro-2H-pyran (DHP, 2.05 equiv, 41 mmol) were added. The resulting clear solution was stirred overnight at room temperature. When the reaction was finished, the solution was extracted 2 times with an aqueous saturated solution of $NaHCO_3$, with water and with an aqueous saturated solution of NaCl. The organic layer was dried over $MgSO_4$, filtered and solvent was removed under reduce pressure. Crude product was purified by column chromatography using silica gel and hexane: ethyl acetate 4:1 as solvent. **S2** was obtained as a colorless oil in 56% yield.

(*Z*)-1,4-Bis((tetrahydro-2*H*-pyran-2-yl)oxy)but-2-ene (S2)

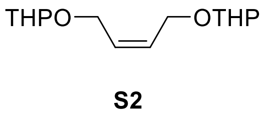

^{1}H NMR (300 MHz, $CDCl_3$) δ 5.72 (t, *J* = 4.2 Hz, 2H), 4.73 – 4.49 (m, 2H), 4.41 – 4.17 (m, 2H), 4.18 – 3.99 (m, 2H), 3.95 – 3.71 (m, 2H), 3.63 – 3.29 (m, 2H), 1.86 – 1.77 (m, 2H), 1.74 – 1.65 (m, 2H), 1.60 – 1.49 (m, 8H). **^{13}C NMR** (75 MHz, $CDCl_3$) δ 129.3, 129.2*, 98.1, 98.1*, 63.0, 62.9*, 62.3, 30.7, 25.6, 19.5.

**Corresponds to the other THP diastereomer.*

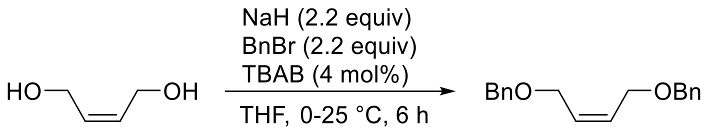

S1 **S3**

A flame-dried 100 ml round-bottom flask equipped with a magnetic stirring bar was charged with sodium hydride (60 m/m% in mineral oil, 2.2 equiv, 44 mmol, 1760 mg). Under argon atmosphere, the solid was washed with dry hexane (3 times 4 ml) and dried in vacuum. Dry tetrahydrofuran (THF, 20 ml) was added as solvent, and cooled to 0 °C by ice/water bath, then (*Z*)-but-2-ene-1,4-diol (**S1**, 1 equiv, 20 mmol) was added dropwise by syringe over 10 minutes to the vigorously stirred solution (CAUTION: H_2 gas formation). The mixture was stirred for 10 minutes at 0 °C, then the cooling bath was removed, and the reaction was warmed up to room temperature. Then a solution of THF (5 ml) THF, tetrabuthylamonium bromide (0.04 equiv, 0.8 mmol) and benzyl bromide (92.2 equiv, 7602 mg) was added in one portion and stirred for 6 hours. The reaction mixture was quenched by careful addition of water and diethyl ether and stirred for 20 minutes. Then the mixture was extracted with diethyl ether. The organic phase was washed 2 times with water and with an aqueous saturated solution of NaCl. The organic layer was dried over $MgSO_4$, filtered and solvent was removed under reduce pressure. Crude product was purified by gradient column chromatography using silica gel and hexane: ethyl acetate 25:1 to 10:1 as eluent. **S3** was obtained as a colorless oil with 91% yield.

(*Z*)-1,4-Bis(benzyloxy)but-2-ene (S3)

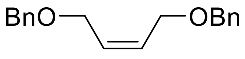

^{1}H NMR (300 MHz, $CDCl_3$) δ 7.90 – 6.97 (m, 10H), 6.53 – 5.39 (m, 2H), 4.54 (s, 4H), 4.11 (d, *J* = 4.6 Hz, 4H). **^{13}C NMR** (75 MHz, $CDCl_3$) δ 138.3, 129.6, 128.5, 127.9, 127.8, 72.4, 65.9.

- **Second step**

A 250 ml round-bottom flask equipped with a magnetic stirring bar was charged with **S2** or **S3** (1 equiv, 6 mmol), 50 ml of diethyl ether was added as solvent and pyridine (4 equiv, 24 mmol). The solution of osmium tetroxide (4% water solution, 0.02 equiv, 0.12 mmol) was added in one portion. Then a solution of sodium periodate (4 equiv, 24 mmol) in distilled water (50 ml) was added dropwise by syringe over 40 minutes. The reaction stirred at room temperature during 24 h. After the reaction finished, diethyl ether and water were added and were extracted 3 times with diethyl ether. Combined organic layer were dried over $MgSO_4$, filtered and the solvent was removed under reduced pressure. Crude product was purified by gradient column chromatography using silica gel and diethyl ether eluent to obtain **S4** or **S5** respectively.

2-((Tetrahydro-2H-pyran-2-yl)oxy)acetaldehyde (S4)

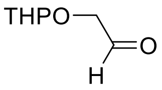

S4 was isolated as a colorless oil in 26% yield. **¹H NMR** (300 MHz, $CDCl_3$) δ 9.74 (t, *J* = 1.1 Hz, 1H), 4.65 (t, *J* = 3.7 Hz, 1H), 4.18 (dd, *J* = 4.5, 1.2 Hz, 2H), 4.01 – 3.77 (m, 1H), 3.65 – 3.40 (m, 1H), 1.94 – 1.64 (m, 3H), 1.60 – 1.48 (m, 3H).

2-(Benzyloxy)acetaldehyde (S5)

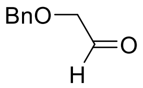

S5 was obtained as an oil in 68% yield. **¹H NMR** (300 MHz, $CDCl_3$) δ 9.75 (s, 1H), 8.40 – 6.90 (m, 5H), 4.66 (s, 2H), 4.12 (s, 2H). **¹³C NMR** (75 MHz, $CDCl_3$) δ 200.5, 137.0, 128.7, 128.3, 128.1, 75.4, 73.8.

- **Third step**

A 100 ml flame-dried round-bottom flask equipped with a magnetic stirring bar was evacuated and backfilled with argon gas and charged with **S4** or **S5** (1 equiv, 5 mmol). Dry tetrahydrofuran was added as solvent to obtain a clear solution, then it was cooled to 0 °C with an ice/water bath. The vinylmagnesium bromide solution (1M in THF, 1.2 equiv, 6 mmol) was added dropwise by syringe over 15 minutes, while the solution turned to yellow. The solution was stirred for 20 minutes at 0 °C, then allowed to warm up to room temperature and stirred for 2 hours. The reaction mixture was quenched carefully by dropwise addition of an aqueous saturated solution of NH_4Cl and stirred for 20 minutes to obtain clear layers, then extracted with ethyl acetate. The organic phase washed 3 times with water and an aqueous saturated solution of NaCl solution. The combined organic phase was separated, dried over $MgSO_4$, filtered and the solvent was removed under reduced pressure. Crude product was purified through flash column chromatography using the indicated mixture of solvents as eluent.

1-((Tetrahydro-2H-pyran-2-yl)oxy)but-3-en-2-ol (S6)

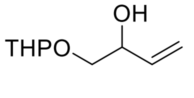

S6

Colorless oil obtained in 75% after column chromatography (hexane: Et_2O, 1:2). **¹H NMR** (300 MHz, $CDCl_3$) δ 6.12 – 5.65 (m, 1H), 5.53 – 5.25 (m, 1H), 5.25 – 4.94 (m, 1H), 4.59 – 4.54 (m, 1H), 4.44 – 4.13 (m, 1H), 4.00 – 3.82 (m, 1H), 3.72 (ddd, *J* = 28.4, 10.8, 3.1 Hz, 1H), 3.59 – 3.46 (m, 2H), 3.00 (bs, 1H), 2.03 – 1.66 (m, 2H), 1.67 – 1.36 (m, 4H). **¹³C NMR** (75 MHz, $CDCl_3$) δ 136.9, 136.5*, 116.3, 116.2*, 100.1, 100.1*, 73.5, 72.6*, 71.7, 71.7*, 63.3, 63.0*, 30.8, 30.6*, 25.2, 25.2*, 19.9, 19.8*.

**Corresponds to the other THP diastereomer.*

1-(Benzyloxy)but-3-en-2-ol (S7)

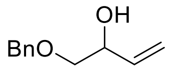

Colorless oil obtained in 47% after column chromatography (Hexane: AcOEt, 8:1 to 4:1). **^{1}H NMR** (300 MHz, $CDCl_3$) δ 7.61 – 7.30 (m, 5H), 5.88 (ddd, *J* = 17.3, 10.6, 5.6 Hz, 1H), 5.39 (dt, *J* = 16.7, 1.2 Hz, 1H), 5.23 (dt, *J* = 10.6, 1.2 Hz, 1H), 4.61 (s, 2H), 4.38 (bs, 1H), 3.81 – 3.51 (m, 1H), 3.50 – 3.26 (m 1H), 2.59 (d, *J* = 3.3 Hz, 1H). **^{13}C NMR** (75 MHz, $CDCl_3$) δ 138.0, 136.8, 128.6, 127.9, 127.9, 116.5, 74.2, 73.5, 71.6.

- **Fourth step**

A 100 ml flame-dried round-bottom flask equipped with a magnetic stirring bar was charged with *N*,*N*-dimethylpyridin-4-amine (4-DMAP, 20 mol%), evacuated and backfilled with argon gas. Then a solution of dry diethyl ether (0.1-0.3 M) with **S6** or **S7** (1 equiv) was added, and the mixture was cooled to 0 °C with an ice/water bath. After 10 minutes of stirring, *di-tert-butyl dicarbonate* (1 equiv) was added in one portion and stirred for additional 10 minutes at 0 °C, then the cooling bath was removed, and the reaction was warmed up to room temperature. The reaction mixture was stirred at room temperature over 24 h. Then, the reaction was quenched by addition of water and diethyl ether and stirred to obtain clear layers. The organic phase was separated, dried over $MgSO_4$, filtered and the solvent was removed under reduced pressure. Crude product was purified through flash column chromatography using the indicated mixture of solvents as eluent.

***tert*-Butyl (1-((tetrahydro-2H-pyran-2-yl)oxy)but-3-en-2-yl) carbonate (60)**

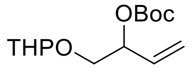

Colorless oil obtained in 73% after column chromatography (hexane: Et_2O, 4:1). The product was obtained as a 1:1 mixture of diastereomers. **^{1}H NMR** (300 MHz, $CDCl_3$) δ 6.06 – 5.64 (m, 1H), 5.36 (dd, *J* = 17.3, 1.1 Hz, 1H), 5.31 – 5.19 (m, 2H), 4.64 (dt, *J* = 12.8, 3.2 Hz, 1H), 3.94 – 3.72 (m, 2H), 3.67 – 3.37 (m, 2H), 1.93 – 1.49 (m, 6H), 1.47 (s, 9H). **^{13}C NMR** (75 MHz, $CDCl_3$) δ 153.1, 153.0*, 133.5, 133.4*, 118.3, 118.6*, 99.2, 98.1*, 82.2, 82.2*, 76.7, 76.0*, 68.8, 68.2*, 62.1, 61.8*, 30.5, 30.4*, 27.9, 25.6, 19.3, 19.0*. **HRMS (APCI)** Calc. for $C_{14}H_{25}O_5$ [M+H$^+$]: 273.1697, found 273.1693.

**Corresponds to the other THP diastereomer.*

1-(Benzyloxy)but-3-en-2-yl tert-butyl carbonate (61)

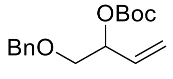

Colorless oil obtained in 73% after column chromatography (hexane: AcOEt, 25:1 to 10:1). **^{1}H NMR** (300 MHz, $CDCl_3$) δ 8.37 – 7.00 (m, 5H), 5.89 (ddd, *J* = 17.1, 10.6, 6.3 Hz, 1H), 5.40 (dt, *J* = 17.3, 1.3 Hz, 1H), 5.36 – 5.24 (m, 2H), 4.60 (s, 2H), 3.84 – 3.53 (m, 2H), 1.52 (s, 9H). **^{13}C NMR** (75 MHz, $CDCl_3$) δ 153.0, 138.1, 133.4, 128.5, 127.7, 118.3, 82.3, 76.2, 73.3, 71.5, 27.9. **HRMS (APCI)** Calc. for $C_{16}H_{23}O_4$ [M+H$^+$]: 279.1591, found 279.1584.

3. Optimization studies

General procedure used for the optimization studies

In a vial, CuCl (5 mol%, 0.02 mmol) and L_{Cu} (5 mol%, 0.02 mmol) were dissolved in toluene (1 ml) and stirred for 15 minutes at room temperature. In a separate vial, $Pd(dba)_2$ (5 mol%, 0.02 mmol) and L_{Pd} (5 mol%, 0.02 mmol) were dissolved in toluene (1 ml) and stirred for 15 minutes at room temperature. The solution of the CuCl/L_{Cu} complex was added to a Schlenk tube containing B_2pin_2 (2 equiv, 0.8 mmol) and MO^tBu (1.5 equiv, 0.6 mmol) to afford a red suspension. Then, allene **1** (1 equiv, 0.4 mmol) was dissolved in dry toluene (1 ml) and added to the Schlenk tube. The solution of the Pd/L_{Pd} complex was then added to the Schlenk tube. Finally, allylic carbonate **2** was dissolved in dry toluene (1 ml) and added to the Schlenk tube. The final solution was stirred at the indicated temperature during 3 h. After this time, the final mixture was filtered through a small pad of silica and solvent was removed under vacuum. The final product was purified by silica gel column chromatography.

3.1 Screening of L_{Cu}

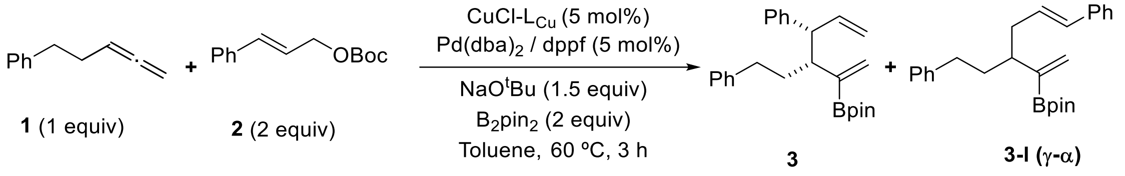

Entry	L_{Cu}	3 yield (%)[a]	3-l yield (%)[a]	dr
**1**	BINAP	60	-	8:1
**2**	dppf	45	-	6:1
**3**	BIPHEP	76	-	14:1
**4**	IMes	46	-	7:1
**5**	SIMes	12[b]	-	-
**6**	XantPhos	77	-	5:1
**8**	dppe	18	36	3:1
**9**	PPh_3	6	9	18:1
**10**	DPEPhos	54	-	3:1

Table S1. [a] Yield of isolated product. [b] Measured by ^{1}H-NMR analysis using trimethyl benzene-1,3,5-tricarboxylate as internal standard.

3.2 Screening of L_{Pd}

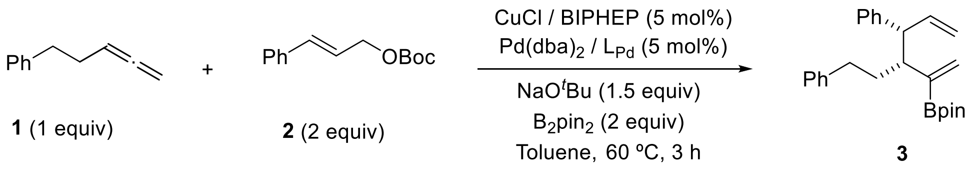

Entry	L_{Pd}	3 yield (%)[a]	dr
1	dppf	76	14:1
2	BIPHEP	75	14:1
3	BINAP	63	17:1
4	PPh_3[b]	40	1:0
5	DPEPhos	60	6:1

Table S2. [a] Yield of isolated product. [b] 10 mol% was used.

3.3 Cooperative catalysis test controls

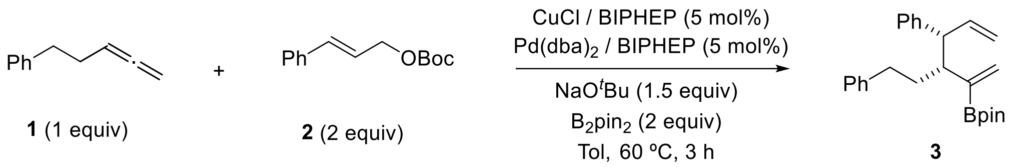

Entry	CuCl-BIPHEP	$Pd(dba)_2$-BIPHEP	3 yield (%)[a]	dr
1[b]	-	5%	-	-
2[c]	5%	-	-	-

Table S3. [a] Determined by ^{1}H-NMR analysis. [b] Decomposition of **1** and **2** was observed [c] **2** was recovered. 24% of protoboration of **1** was observed.

3.4 Screening of temperatures and stoichiometry

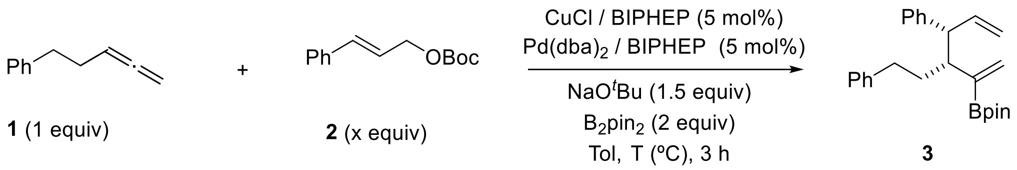

Entry	T (°C)	Equiv. 2	3 yield (%)[a]	dr
1	60	2	75	14:1
2	60	1.5	71	18:1
3	30	1.5	76	>20:1

Table S4. [a] Yield of isolated product.

3.5 Screening of bases

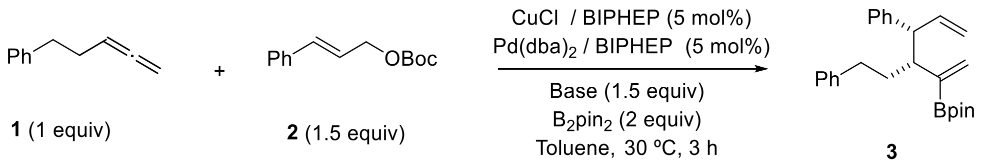

Entry	Base	3 yield (%)[a]	dr
1	NaO^{t}Bu	76	>20:1
2	LiO^{t}Bu	14[b]	-
3	KO^{t}Bu	43	19:1

Table S5. [a] Yield of isolated product. [b] Determined by ^{1}H-NMR analysis.

3.6 Screening of solvents

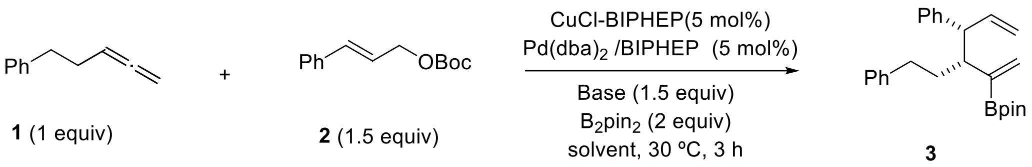

Entry	Solvent	3 yield (%)[a]	dr
1	Toluene	76	>20:1
2	THF	43	>20:1

Table S6. [a] Yield of isolated product.

3.7 Screening of leaving groups

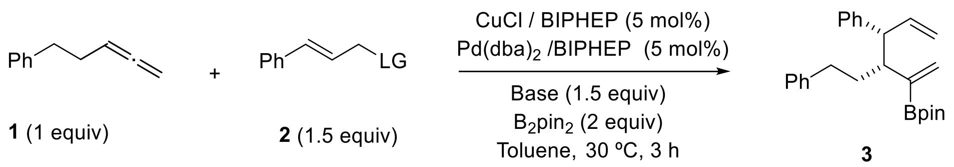

Entry	LG	3 yield (%)[a]	dr
1	$OCO_2{}^tBu$	76	>20:1
2	Cl	-	-

Table S7. [a] Yield of isolated product.

4. General procedure for the Cu/Pd-catalyzed borylative allyl-allyl coupling (General Procedure A)

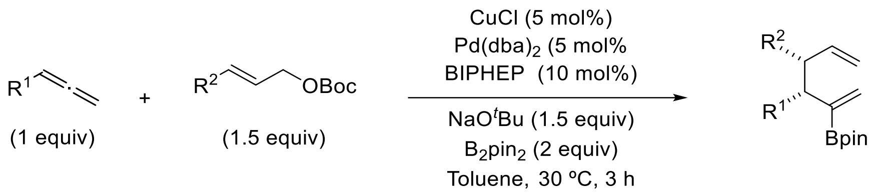

In a Schlenk flask which contains B_2pin_2 (2 equiv, 0.8 mmol), NaO^tBu (1.5 equiv, 0.6 mmol), CuCl (5 mol%, 0.02 mmol), $Pd(dba)_2$ (5 mol%, 0.02 mmol) and BIPHEP (10 mol%, 0.04mmol) was added a solution of the corresponding allene (0.4 mmol) in 2ml of dry toluene and allyl carbonate (1.5 equiv, 0.6 mmol) in 2 ml of dry toluene. The final solution was stirred at 30 °C for 3 hours. After this time, the final mixture was filtered through a small amount of silica and solvent was removed under vacuum. The final product was purified by silica gel column chromatography.

5. Compound characterization

4,4,5,5-Tetramethyl-2-((3*R,4*R**)-3-phenethyl-4-phenylhexa-1,5-dien-2-yl)-1,3,2-dioxaborolane (3).**

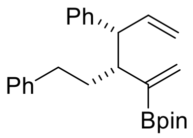

Synthesized from **1**, **2** and B_2pin_2 according to general procedure A. Yellow oil obtained in 76% yield (>20:1 dr) after column chromatography (Hexane/ EtOAc 4:1). **^{1}H NMR** (300 MHz, $CDCl_3$) δ 7.38 – 7.13 (m, 10H), 6.14 – 5.93 (m, 1H), 5.84 (d, *J* = 3.4 Hz, 1H), 5.49 (d, *J* = 3.4 Hz, 1H), 5.18 – 5.06 (m, 2H), 3.61 (t, *J* = 9.7 Hz, 1H), 2.74 – 2.42 (m, 3H), 2.20 – 1.85 (m, 2H), 1.33 (s, 6H), 1.30 (s, 6H). **^{13}C NMR** (75 MHz, $CDCl_3$) δ 144.3, 143.2, 141.8, 132.1, 128.5, 128.4, 128.3, 128.2, 125.6, 125.5, 115.3, 83.1, 55.4, 51.0, 34.3, 34.2, 24.8, 24.7. **^{11}B NMR** (160 MHz, $CDCl_3$) δ 30.5. **HRMS (APCI)** Calc. for $C_{28}H_{34}BO_2$ [M+H$^+$]: 389.2646, found 389.2657.

4,4,5,5-Tetramethyl-2-((*R)-3-((*R**)-1-phenylallyl)dec-1-en-2-yl)-1,3,2-dioxaborolane (4).**

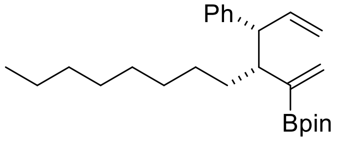

Synthesized from **44**, **2** and B_2pin_2 according to general procedure A. Yellow oil obtained in 76% yield (>20:1 dr) after column chromatography (Hexane/ EtOAc 4:1). **^{1}H NMR** (300 MHz, $CDCl_3$) δ 7.36 – 7.28 (m, 1H), 7.19 (t, *J* = 7.5 Hz, 2H), 7.09 (d, *J* = 7.3 Hz, 2H), 5.95 (dt, *J* = 17.2, 9.7 Hz, 1H), 5.65 (d, *J* = 3.5 Hz, 1H), 5.33 (d, *J* = 3.5 Hz, 1H), 5.10 – 4.94 (m, 2H), 3.48 (t, *J* = 9.8 Hz, 1H), 2.47 (td, *J* = 10.7, 3.4 Hz, 1H), 1.71 – 1.60 (m, 1H), 1.50 – 1.40 (m, 1H), 1.29 – 1.23 (m, 12H), 1.22 (s, 6H), 1.19 (s, 6H), 0.87 (t, *J* = 6.5 Hz, 3H). **^{13}C NMR** (75 MHz, $CDCl_3$) δ 144.5, 142.1, 131.3, 128.4, 128.1, 125.6, 114.9, 82.9, 55.4, 50.9, 32.1, 31.9, 29.7, 29.6, 29.4, 27.7, 24.9, 24.6, 22.7, 14.1. **^{11}B NMR** (160 MHz, $CDCl_3$) δ 30.5, **HRMS (APCI)** Calc. for $C_{26}H_{41}BO_2$[M+H$^+$]: 397.3278, found 397.3299.

2-((3*R,4*R**)-3-Benzyl-4-phenylhexa-1,5-dien-2-yl)-4,4,5,5-tetramethyl-1,3,2-dioxaborolane (5).**

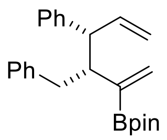

Synthesized from **45**, **2** and B_2pin_2 according to general procedure A. Yellow oil obtained in 57% yield (>20:1 dr) after column chromatography (Hexane/ EtOAc 4:1). **^{1}H NMR** (300 MHz, $CDCl_3$) δ 7.30 – 7.10 (m, 10H), 6.22 – 6.03 (m, 1H), 5.61 – 5.53 (m, 1H), 5.30 – 5.10 (m, 3H), 3.68 (t, *J* = 9.7 Hz, 1H), 3.22 (d, *J* = 12.9, 1H), 2.93 – 2.83 (m, 1H), 2.82 – 2.71 (m, 1H), 1.25 (s, 6H), 1.23 (s, 6H). **^{13}C NMR** (75 MHz, $CDCl_3$) δ 143.9, 142.0, 141.6, 132.4, 129.4, 128.3, 128.2, 127.9, 125.8, 125.4, 115.5, 83.0, 55.4, 52.9, 39.2, 24.85, 24.62. **^{11}B NMR** (160 MHz, $CDCl_3$) δ 30.9. **HRMS (APCI)** Calc. for $C_{25}H_{32}BO_2$ [M+H$^+$]: 375.2490, found 375.2504.

***tert*-Butyldimethyl(((3*R**,4*R**)-4-phenyl-3-(1-(4,4,5,5-tetramethyl-1,3,2-dioxaborolan-2-yl)vinyl)hex-5-en-1-yl)oxy)silane (6).**

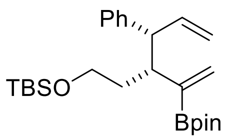

Synthesized from **46**, **2** and B_2pin_2 according to general procedure A. Yellow oil obtained in 73% yield (>20:1 dr) after column chromatography (Hexane/ EtOAc 4:1). **^{1}H NMR** (300 MHz, $CDCl_3$) δ 7.29 – 7.19 (m, 2H), 7.15 – 7.07 (m, 3H), 6.03 – 5.88 (m, 1H), 5.68 (d, *J* = 3.5 Hz, 1H), 5.39 (d, *J* = 3.5 Hz, 1H), 5.15 – 5.02 (m, 2H), 3.66 – 3.40 (m, 3H), 2.64 (td, *J* = 10.7, 3.1 Hz, 1H), 2.00 (dtd, *J* = 13.4, 8.0, 3.1 Hz, 1H), 1.80 – 1.62 (m, 1H), 1.25 (s, 6H), 1.21 (s, 6H), 0.92 (s, 9H), 0.05 (d, *J* = 1.9 Hz, 6H). **^{13}C NMR** (75 MHz, $CDCl_3$) δ 144.2, 141.7, 131.7, 128.3, 128.1, 125.7, 115.2, 83.0, 61.8, 54.9, 47.7, 35.0, 26.0, 24.8, 24.6, 18.3, -5.2. **^{11}B NMR** (160 MHz, $CDCl_3$) δ 31.3. **HRMS (APCI)** Calc. for $C_{26}H_{44}BO_3Si$ [M+H$^+$]: 443.3147, found 443.3157.

(4*R,5*R**)-5-Phenyl-4-(1-(4,4,5,5-tetramethyl-1,3,2-dioxaborolan-2-yl)vinyl)hept-6-en-1-yl pivalate (7).**

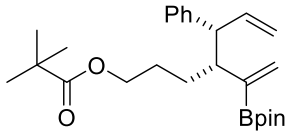

Synthesized from **47**, **2** and B_2pin_2 according to general procedure A. Yellow oil obtained in 71% yield (>20:1 dr) after column chromatography (Hexane/ EtOAc 4:1). **^{1}H NMR** (300 MHz, $CDCl_3$) δ 7.26 – 7.19 (m, 2H), 7.12 – 7.10 (m, 3H), 5.94 (dt, *J* = 16.6, 9.6 Hz, 1H), 5.71 (d, *J* = 3.3 Hz, 1H), 5.39 (d, *J* = 3.3 Hz, 1H), 5.13 – 5.00 (m, 2H), 4.04 (t, *J* = 6.2 Hz, 2H), 3.51 (t, *J* = 9.8 Hz, 1H), 2.52 (td, *J* = 10.4, 3.2 Hz, 1H), 1.87 – 1.74 (m, 1H), 1.70 – 1.40 (m, 3H) , 1.24 (s, 6H), 1.21 (s, 15H). **^{13}C NMR** (75 MHz, $CDCl_3$) δ 178.6, 144.1, 141.7, 131.8, 128.3, 128.1, 125.7, 115.2, 83.1, 64.4, 55.3, 50.4, 38.7, 28.3, 27.2, 26.9, 24.8, 24.6. **^{11}B NMR** (160 MHz, $CDCl_3$) δ 31.2. **HRMS (APCI)** Calc. for $C_{26}H_{40}BO_4$ [M+H$^+$]: 427.3014, found 427.3029.

2-((3*R,4*R**)-3-(3-(Benzyloxy)propyl)-4-phenylhexa-1,5-dien-2-yl)-4,4,5,5-tetramethyl-1,3,2-dioxaborolane (8).**

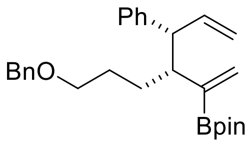

Synthesized from **48**, **2** and B_2pin_2 according to general procedure A. Yellow oil obtained in 79% yield (>20:1 dr) after column chromatography (Hexane/ EtOAc 4:1). **^{1}H NMR** (300 MHz, $CDCl_3$) δ 7.42 – 7.23 (m, 7H), 7.18 – 7.11 (m, 3H), 6.01 (dt, *J* = 17.0, 9.6 Hz, 1H), 5.74 (d, *J* = 3.4 Hz, 1H), 5.41 (d, *J* = 3.4 Hz, 1H), 5.20 – 5.03 (m, 2H), 4.54 (s, 2H), 3.61 – 3.48 (m, 3H), 2.56 (td, *J* = 10.4, 3.3 Hz, 1H), 1.94 – 1.83 (m, 1H), 1.71 – 1.50 (m, 3H), 1.27 (s, 6H), 1.24 (s, 6H). **^{13}C NMR** (75 MHz, $CDCl_3$) δ 144.3, 141.9, 138.9, 131.7, 128.4, 128.3, 128.2, 127.6, 127.4, 125.7, 115.1, 83.0, 72.7, 70.5, 55.5, 50.7, 28.6, 27.9, 24.9, 24.6. **^{11}B NMR** (160 MHz, $CDCl_3$) δ 31.7. **HRMS (APCI)** Calc. for $C_{28}H_{38}BO_3$ [M+H$^+$]: 433.2909, found 433.2917.

(5*R,6*R**)-6-Phenyl-5-(1-(4,4,5,5-tetramethyl-1,3,2-dioxaborolan-2-yl)vinyl)oct-7-enenitrile (9).**

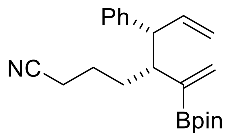

Synthesized from **49**, **2** and B_2pin_2 according to general procedure A. Yellow oil obtained in 71% yield (>20:1 dr) after column chromatography (Hexane/ EtOAc 4:1). **^{1}H NMR** (300 MHz, $CDCl_3$) δ 7.24 – 7.16 (m, 2H), 7.11 – 7.02 (m, 3H), 5.94 (dt, *J* = 16.9, 9.7 Hz, 1H), 5.70 (d, *J* = 3.2 Hz, 1H), 5.38 (d, *J* = 3.3 Hz, 1H), 5.15 – 5.01 (m, 2H), 3.48 (t, *J* = 9.8 Hz, 1H), 2.49 (td, *J* = 10.5, 3.5 Hz, 1H), 2.30 (td, *J* = 7.0, 5.5 Hz, 2H), 1.89 – 1.76 (m, 1H), 1.73 – 1.56 (m, 2H), 1.56 – 1.40 (m, 1H), 1.23 (s, 6H), 1.20 (s, 6H). **^{13}C NMR** (75 MHz, $CDCl_3$) δ 143.7, 141.3, 132.1, 128.2, 125.9, 119.8, 115.6, 83.2, 55.4, 50.1, 31.2, 25.0, 24.9, 24.6, 23.7, 17.0. **^{11}B NMR** (160 MHz, $CDCl_3$) δ 31.1. **HRMS (APCI)** Calc. for $C_{22}H_{31}BNO_2$ [M+H$^+$]: 352.2442, found 352.2453.

***tert*-Butyl benzyl((5*R**,6*R**)-6-phenyl-5-(1-(4,4,5,5-tetramethyl-1,3,2-dioxaborolan-2-yl)vinyl)oct-7-en-1-yl)carbamate (10).**

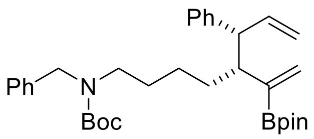

Synthesized from **50**, **2** and B_2pin_2 according to general procedure A. Yellow oil obtained in 56% yield (>20:1 dr) after column chromatography (Hexane/ EtOAc 7:3). **^{1}H NMR** (300 MHz, $CDCl_3$) δ 7.37 – 7.17 (m, 8H), 7.14 – 7.07 (m, 2H), 5.94 (dt, *J* = 17.0, 9.7 Hz, 1H), 5.66 (d, *J* = 3.4 Hz, 1H), 5.33 (d, *J* = 3.5 Hz, 1H), 5.11 – 4.99 (m, 2H), 4.43 (bs, 2H), 3.47 (t, *J* = 9.8 Hz, 1H), 3.11 (bs, 2H), 2.45 (td, *J* = 10.6, 3.4 Hz, 1H), 1.48 (bs, 15H), 1.23 (s, 6H), 1.19 (s, 6H). **^{13}C NMR** (126 MHz, $CDCl_3$) δ 143.3, 142.6, 140.9, 137.6, 130.5, 127.4, 127.3, 127.1, 126.7, 126.0, 124.7, 114.0, 82.2, 82.0, 54.4, 49.4, 48.8, 45.5, 30.7, 27.5, 24.0, 23.8, 23.6, 23.5. **^{11}B NMR** (160 MHz, $CDCl_3$) δ 30.4. **HRMS (APCI)** Calc. for $C_{34}H_{49}BNO_4$ [M+H$^+$]: 546.3749, found 546.3760.

4,4,5,5-Tetramethyl-2-((3*R,4*R**)-3-phenethyl-4-(4-(trifluoromethyl)phenyl)hexa-1,5-dien-2-yl)-1,3,2-dioxaborolane (11).**

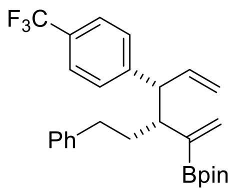

Synthesized from **1**, **53** and B_2pin_2 according to general procedure A. Yellow oil obtained in 80% yield (>20:1 dr) after column chromatography (Hexane/ EtOAc 4:1). **^{1}H NMR** (300 MHz, $CDCl_3$) δ 7.47 (d, *J* = 8.4 Hz, 2H), 7.27 (d, *J* = 7.3 Hz, 2H), 7.24 – 7.12 (m, 5H), 5.90 (dt, *J* = 17.0, 9.7 Hz, 1H), 5.78 (d, *J* = 3.3 Hz, 1H), 5.44 (d, *J* = 3.3 Hz, 1H), 5.14 – 5.00 (m, 2H), 3.59 (t, *J* = 9.8 Hz, 1H), 2.69 – 2.50 (m, 2H), 2.46 – 2.33 (m, 1H), 2.12 – 1.97 (m, 1H), 1.92 – 1.74 (m, 1H), 1.25 (s, 6H), 1.22 (s, 6H). **^{13}C NMR** (75 MHz, $CDCl_3$) δ 148.7, 143.2, 141.1, 132.8, 129.0, 128.8, 128.6, 128.1, 125.9, 125.4 (q, *J* = 4.5 Hz), 124.6 (q, *J* = 262.4 Hz) 116.4, 83.6, 55.6, 51.0, 34.5, 34.4, 25.1, 24.9. **^{19}F NMR** (282 MHz, $CDCl_3$) δ -62.05. **^{11}B NMR** (160 MHz, $CDCl_3$) δ 31.7. **HRMS (APCI)** Calc. for $C_{27}H_{33}BF_3O_2$ [M+H$^+$]: 457.2520, found 457.2522.

***tert*-Butyldimethyl(((3*R**,4*R**)-3-(1-(4,4,5,5-tetramethyl-1,3,2-dioxaborolan-2-yl)vinyl)-4-(4-(trifluoromethyl)phenyl)hex-5-en-1-yl)oxy)silane (12).**

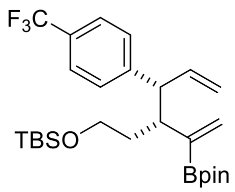

Synthesized from **46**, **53** and B_2pin_2 according to general procedure A. Yellow oil obtained in 80% yield (>20:1 dr) after column chromatography (Hexane/ EtOAc 7:3). **^{1}H NMR** (500 MHz, $CDCl_3$) δ 7.48 (d, *J* = 8.6 Hz, 2H), 7.23 (d, *J* = 7.9 Hz, 2H), 5.94 (ddd, *J* = 16.9, 10.1, 9.1 Hz, 1H), 5.71 (d, *J* = 3.4 Hz, 1H), 5.42 (d, *J* = 3.4 Hz, 1H), 5.17 – 5.06 (m, 2H), 3.59 – 3.50 (m, 2H), 3.48 – 3.37 (m, 1H), 2.68 (td, *J* = 10.8, 3.0 Hz, 1H), 2.00 – 1.90 (m, 1H), 1.70 – 1.59 (m, 1H), 1.24 (s, 6H), 1.21 (s, 6H), 0.95 – 0.90 (s, 9H), 0.00 (s, 3H), -0.01 (s, 3H). **^{13}C NMR** (75 MHz, $CDCl_3$) δ 148.4, 140.8, 132.2, 128.7, 128.0 (q, *J* = 32.6 Hz), 125.0 (q, *J* = 3.8 Hz), 124.4 (q, *J* = 267.4 Hz), 116.1, 83.1, 61.5, 54.8, 47.4, 35.0, 26.0, 24.8, 24.6, 18.3, -5.2, -5.3. **^{19}F NMR** (282 MHz, $CDCl_3$) δ -62.07. **^{11}B NMR** (160 MHz, $CDCl_3$) δ 30.2. **HRMS (APCI)** Calc. for $C_{27}H_{43}BF_3O_3Si$ [M+H$^+$]: 511.2936, found 511.2934.

2-((3*R,4*R**)-4-(3-Chlorophenyl)-3-phenethylhexa-1,5-dien-2-yl)-4,4,5,5-tetramethyl-1,3,2-dioxaborolane (13).**

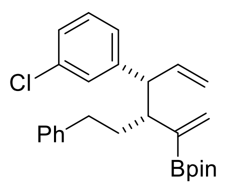

Synthesized from **1**, **54** and B_2pin_2 according to general procedure A. Yellow oil obtained in 71% yield (>20:1 dr) after column chromatography (Hexane/ EtOAc 4:1). **^{1}H NMR** (300 MHz, $CDCl_3$) δ 7.34 – 7.25 (m, 2H), 7.23 – 7.07 (m, 6H), 6.96 (d, *J* = 7.4 Hz, 1H), 5.88 (dt, *J* = 16.7, 9.6 Hz, 1H), 5.77 (d, *J* = 3.3 Hz, 1H), 5.43 (d, *J* = 3.4 Hz, 1H), 5.12 – 5.01 (m, 2H), 3.52 (t, *J* = 9.9 Hz, 1H), 2.70 – 2.33 (m, 3H), 2.13 – 1.97 (m, 1H), 1.89 – 1.73 (m, 1H), 1.28 (s, 6H), 1.24 (s, 6H). **^{13}C NMR** (75 MHz, $CDCl_3$) δ 146.7, 143.3, 141.3, 134.1, 132.8, 129.7, 128.9, 128.8, 128.6, 126.7, 126.2, 125.9, 116.2, 83.5, 55.3, 51.3, 34.4, 25.3, 25.1, 24.9. **^{11}B NMR** (160 MHz, $CDCl_3$) δ 31.5. **HRMS (APCI)** Calc. for $C_{26}H_{33}BClO_2$ [M+H$^+$]: 423.2257, found 423.2272.

2-((3*R,4*R**)-4-(2-Fluorophenyl)-3-phenethylhexa-1,5-dien-2-yl)-4,4,5,5-tetramethyl-1,3,2-dioxaborolane (14).**

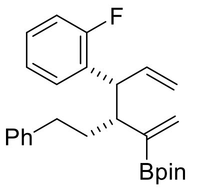

Synthesized from **1**, **55** and B_2pin_2 according to general procedure A. Yellow oil obtained in 67% yield (>20:1 dr) after column chromatography (Hexane/ EtOAc 4:1). **^{1}H NMR** (300 MHz, $CDCl_3$) δ 7.35 – 7.23 (m, 2H), 7.23 – 7.02 (m, 5H), 7.04 – 6.84 (m, 2H), 6.08 – 5.90 (m, 1H), 5.74 (d, *J* = 3.3 Hz, 1H), 5.49 (d, *J* = 3.6 Hz, 1H), 5.17 – 4.98 (m, 2H), 3.79 (t, *J* = 10.0 Hz, 1H), 2.73 – 2.53 (m, 2H), 2.48 – 2.32 (m, 1H), 2.11 – 1.97 (m, 1H), 1.97 – 1.81 (m, 1H), 1.26 (s, 6H), 1.22 (s, 6H). **^{13}C NMR** (75 MHz, $CDCl_3$) δ 160.6 (d, *J* = 237.9 Hz), 143.2, 140.1, 132.1, 130.1 (d, *J* = 5.8 Hz), 128.4, 128.2, 128.1, 127.3 (d, *J* = 8.4 Hz) , 125.5, 123.7 (d, *J* = 3.2 Hz) , 116.0, 115.3 (d, *J* = 23.1 Hz), 83.1, 50.0, 49.9, 49.7, 34.3, 24.9, 24.5. **^{19}F NMR** (282 MHz, $CDCl_3$) δ -116.13. **^{11}B NMR** (160 MHz, $CDCl_3$) δ 31.3. **HRMS (APCI)** Calc. for $C_{26}H_{33}BFO_2$ [M+H$^+$]: 407.3525, found 407.3521.

2-((3*R,4*R**)-4-(4-Methoxyphenyl)-3-phenethylhexa-1,5-dien-2-yl)-4,4,5,5-tetramethyl-1,3,2-dioxaborolane (15).**

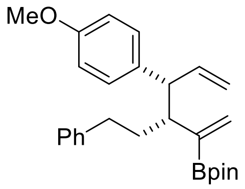

Synthesized from **1**, **56** and B_2pin_2 according to general procedure A. Yellow oil obtained in 77% yield (>20:1 dr) after column chromatography (Hexane/ EtOAc 4:1). **^{1}H NMR** (300 MHz, $CDCl_3$) δ 7.27 (d, *J* = 7.1 Hz, 2H), 7.22 – 7.14 (m, 3H), 7.01 (d, *J* = 8.6 Hz, 2H), 6.77 (d, *J* = 8.6 Hz, 2H), 5.92 (dt, *J* = 16.9, 9.5 Hz, 1H), 5.77 (d, *J* = 3.5 Hz, 1H), 5.42 (d, *J* = 3.4 Hz, 1H), 5.11 – 4.95 (m, 2H), 3.77 (s, 3H), 3.49 (t, *J* = 9.8 Hz, 1H), 2.76 – 2.33 (m, 3H), 2.11 – 1.95 (m, 1H), 1.93 – 1.78 (m, 1H), 1.27 (s, 6H), 1.24 (s, 6H). **^{13}C NMR** (75 MHz, $CDCl_3$) δ 157.9, 143.5, 142.3, 136.7, 132.3, 129.5, 128.8, 128.5, 125.8, 115.2, 113.9, 83.4, 55.4, 54.7, 51.4, 34.6, 34.5, 25.2, 25.0. **^{11}B NMR** (160 MHz, $CDCl_3$) δ 31.7. **HRMS (APCI)** Calc. for $C_{27}H_{36}BO_3$ [M+H$^+$]: 419.2752, found 419.2755.

4,4,5,5-Tetramethyl-2-((3*R,4*R**)-4-methyl-3-phenethylhexa-1,5-dien-2-yl)-1,3,2-dioxaborolane (16) and 4,4,5,5-tetramethyl-2-((3*R**,4*S**)-4-methyl-3-phenethylhexa-1,5-dien-2-yl)-1,3,2-dioxaborolane (16').**

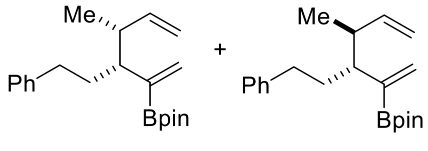

16 **16'**

Synthesized from **1**, **58** and B_2pin_2 according to general procedure A. Yellow oil obtained in 76% yield (3:1 dr) after column chromatography (Hexane/ EtOAc 4:1). **^{1}H NMR** (300 MHz, $CDCl_3$) δ 7.30 – 7.23 (m, 2H **16 +16'**), 7.20 – 7.13 (m, 3H **16 +16'**), 5.96 (d, *J* = 3.6 Hz, 1H **16 +16'**), 5.73 – 5.62 (m, 1H **16 +16'**), 5.61 (d, *J* = 2.5 Hz, 1H **16**), 5.57 (d, *J* = 3.7 Hz, 1H **16'**), 5.03 – 4.86 (m, 2H **16 +16'**), 2.67 – 2.51 (m, 1H **16 +16'**), 2.44 – 2.26 (m, 2H **16 +16'**), 2.00 (td, *J* = 9.8, 8.9, 3.8 Hz, 1H **16 +16'**), 1.91 – 1.75 (m, 2H **16 +16'**), 1.29 (s, 12H **16 +16'**), 0.99 (d, *J* = 6.8 Hz, 3H **16'**), 0.89 (d, *J* = 6.8 Hz, 3H **16**). **^{13}C NMR** (75 MHz, $CDCl_3$) δ 144.2 (**16**), 143.9 (**16'**), 143.4 (**16+ 16'**), 130.8 (**16**), 130.6 (**16'**), 128.4 (**16+ 16'**), 128.2 (**16'**), 128.1 (**16**), 125.4 (**16'**), 125.3 (**16**) 113.5 (**16**), 112.8 (**16'**), 83.1 (**16+ 16'**), 51.8 (**16**), 51.3 (**16'**), 42.1 (**16**), 41.8 (**16'**), 34.4 (**16'**), 34.3 (**16**) 33.8 (**16**), 32.9 (**16'**),

24.8 (**16’**), 24.7 (**16**), 24.7 (**16+ 16’**), 19.2 (**16**), 17.9 (**16’**). **^{11}B NMR** (160 MHz, $CDCl_3$) δ 31.1. **HRMS (APCI)** Calc. for $C_{21}H_{32}BO_2$ [M+H$^+$]: 327.2490, found 327.2494.

4,4,5,5-Tetramethyl-2-((3*R,4*R**)-3-phenethyl-4-(((tetrahydro-2*H*-pyran-2-yl)oxy)methyl)hexa-1,5-dien-2-yl)-1,3,2-dioxaborolane (17).**

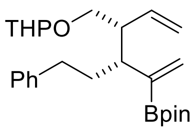

Synthesized from **1**, **60** and B_2pin_2 according to general procedure A. Yellow oil obtained in 65% yield (8:1 dr)** after column chromatography (Hexane/ EtOAc 4:1). **^{1}H NMR** (300 MHz, $CDCl_3$) δ 7.31 – 7.24 (m, 2H), 7.17 (m, 3H), 5.97 (dd, *J* = 3.6, 1.7 Hz, 1H), 5.82 – 5.68 (m, 1H), 5.67 – 5.59 (m, 1H), 5.13 – 5.03 (m, 2H), 4.53 (dt, *J* = 13.7, 3.4 Hz, 1H), 3.92 – 3.68 (m, 1H), 3.62 – 3.43 (m, 2H), 3.40 – 3.21 (m, 1H), 2.65 – 2.47 (m, 2H), 2.41 – 2.18 (m, 2H), 1.94 – 1.75 (m, 3H), 1.74 – 1.44 (m, 5H), 1.30 (s, 12H). **^{13}C NMR** (75 MHz, $CDCl_3$) δ 143.3, 143.3*, 140.2, 140.1*, 131.2, 128.4, 128.2, 125.4, 116.4, 116.3*, 99.0, 98.2*, 83.2, 69.9, 69.7*, 61.8, 61.7*, 48.2, 47.9*, 47.8, 47.3*, 34.2, 34.1*, 33.5, 30.6, 30.6*, 25.6, 24.8, 24.7, 19.4, 19.3*. **^{11}B NMR** (160 MHz, $CDCl_3$) δ 29.8. **HRMS (APCI)** Calc. for $C_{26}H_{40}BO_4$ [M+H$^+$]: 426.2930, found 426.3002.

***Note: diastereomers arising from the THP group are not considered in this value.*

**Corresponds to the other THP diastereomer.*

2-((3*R,4*R**)-4-((Benzyloxy)methyl)-3-phenethylhexa-1,5-dien-2-yl)-4,4,5,5-tetramethyl-1,3,2-dioxaborolane (18).**

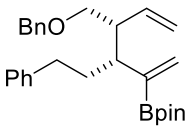

Synthesized from **1**, **61** and B_2pin_2 according to general procedure A. Yellow oil obtained in 87% yield (8:1 dr) after column chromatography (Hexane/EtOAc 4:1). **^{1}H NMR** (300 MHz, $CDCl_3$) δ 7.37 – 7.23 (m, 7H), 7.20 – 7.13 (m, 3H), 5.95 (d, *J* = 3.6 Hz, 1H), 5.79 – 5.62 (m, 2H), 5.17 – 5.07 (m, 2H), 4.46 (d, *J* = 3.6 Hz, 2H), 3.45 (dd, *J* = 9.4, 4.5 Hz, 1H), 3.34 (dd, *J* = 9.4, 7.2 Hz, 1H)., 2.66 – 2.53 (m, 2H), 2.42 – 2.18 (m, 2H), 1.95 – 1.74 (m, 2H), 1.28 (s, 12H). **^{13}C NMR** (75 MHz, $CDCl_3$) δ 143.2, 140.1, 138.9, 131.2, 128.4, 128.2, 128.2, 127.5, 127.3, 125.4, 116.5, 83.2, 72.9, 72.8, 48.1, 47.7, 34.1, 33.5, 24.8, 24.7. **^{11}B NMR** (160 MHz, $CDCl_3$) δ 30.2. **HRMS (APCI)** Calc. for $C_{28}H_{38}BO_3$ [M+H$^+$]: 433.2909, found 433.2920.

4,4,5,5-Tetramethyl-2-((3*R,4*R**,*E*)-3-phenethyl-4,6-diphenylhexa-1,5-dien-2-yl)-1,3,2-dioxaborolane (19) and 4,4,5,5-Tetramethyl-2-((3*R**,4*S**,*E*)-3-phenethyl-4,6-diphenylhexa-1,5-dien-2-yl)-1,3,2-dioxaborolane (19’).**

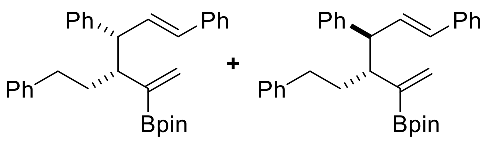

19 **19'**

Synthesized from **1**, **62** and B_2pin_2 according to general procedure A at 60 °C. Yellow oil obtained in 70% yield (1.5:1 dr) after column chromatography (Hexane/ EtOAc 4:1). **^{1}H NMR** (300 MHz, $CDCl_3$) δ 7.33 – 7.13 (m, 15H **19 + 19’**), 6.48 – 6.22 (m, 2H **19 + 19’**), 6.02 (d, *J* = 3.5 Hz, 1H **19’**), 5.79 (d, *J* = 3.4 Hz, 1H **19**), 5.69 (d, *J* = 3.5 Hz, 1H **19’**), 5.46 (d, *J* = 3.4 Hz, 1H **19**), 3.70 (t, *J* = 9.6 Hz, 1H **19 + 19’**), 2.62 (td, *J* = 11.3, 10.3, 5.0 Hz, 2H **19 + 19’**),

2.48 – 2.28 (m, 1H **19 + 19'**), 2.16 – 2.02 (m, 1H **19 + 19'**), 1.98 – 1.85 (m, 1H **19 + 19'**), 1.29 (s, 6H **19**), 1.28 (s, 6H **19**), 1.26 (s, 6H **19'**), 1.24 (s, 6H **19'**). **^{13}C NMR** (75 MHz, $CDCl_3$) δ 144.3, 144.2, 143.1, 142.8, 138.0, 137.8, 134.6, 133.7, 132.4, 132.0, 130.5, 129.6, 128.6, 128.6, 128.5, 128.5, 128.4, 128.3, 128.3, 128.2, 127.0, 126.8, 126.3, 126.2, 125.9, 125.6, 125.5, 83.3, 83.2, 54.6, 54.1, 51.9, 51.6, 34.3, 34.0, 33.6, 25.0, 24.9, 24.7, 24.6. **^{11}B NMR** (160 MHz, $CDCl_3$) δ 29.7. **HRMS (APCI)** Calc. for $C_{32}H_{38}BO_2$ $[M+H^+]$: 465.2959, found 465.2959.

4,4,5,5-Tetramethyl-2-((3*R,4*R**,*E*)-4-methyl-3-phenethyl-6-phenylhexa-1,5-dien-2-yl)-1,3,2-dioxaborolane (20) and 4,4,5,5-Tetramethyl-2-((3*R**,4*S**,*E*)-4-methyl-3-phenethyl-6-phenylhexa-1,5-dien-2-yl)-1,3,2-dioxaborolane (20').**

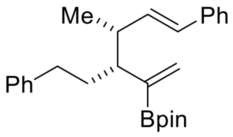

20

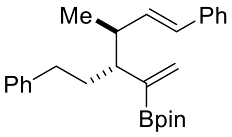

20'

Synthesized from **1**, **63** and B_2pin_2 according to general procedure A at 60 °C. Yellow oil obtained in 71% yield (1:1 dr) after column chromatography (Hexane/ EtOAc 4:1). **^{1}H NMR** (300 MHz, $CDCl_3$) δ 7.39 – 7.08 (m, 10H **20** + **20'**), 6.35 (d, *J* = 15.8 Hz, 1H, **20**), 6.27 (d, *J* = 15.9 Hz, 1H, **20'**), 6.14 – 6.01 (m, 1H **20** +**20'**), 5.99 (d, *J* = 3.7 Hz 1H, **20**), 5.97 (d, *J* = 4.1 Hz, 1H, **20'**), 5.64 (d, *J* = 3.7 Hz, 1H, **20**), 5.61 (d, *J* = 3.6 Hz, 1H, **20'**), 2.70 – 2.33 (m, 3H **20** +**20'**), 2.31 – 2.18 (m, 1H **20**), 2.15 – 2.02 (m, 1H **20'**), 1.95 – 1.79 (m, 2H **20** +**20'**), 1.28 (s, 12H, **20**) 1.25 (s, 6H, **20'**), 1.24 (s, 6H, **20'**), 1.07 (d, *J* = 6.7 Hz, 3H, **20**), 0.97 (d, *J* = 6.7 Hz, 3H, **20'**). **^{13}C NMR** (75 MHz, $CDCl_3$) δ 143.3, 143.3, 138.2, 138.1, 136.3, 136.1, 131.2, 131.1, 129.1, 128.5, 128.5, 128.5, 128.4, 128.3, 128.2, 126.8, 126.7, 126.1, 125.6, 125.5, 83.2, 83.2, 52.4, 51.6, 41.6, 41.3, 34.4, 34.4, 34.0, 33.4, 25.0, 24.8, 24.8, 24.7, 19.7, 18.6. **^{11}B NMR** (160 MHz, $CDCl_3$) δ 30.5. **HRMS (APCI)** Calc. for $C_{27}H_{36}BO_2$ $[M+H^+]$: 403.2803, found 403.2814.

4,4,5,5-Tetramethyl-2-(3-phenethyl-4-vinylhexa-1,5-dien-2-yl)-1,3,2-dioxaborolane (21).

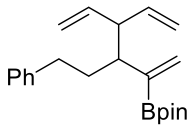

Synthesized from **1**, **64** and B_2pin_2 according to general procedure A. Yellow oil obtained in 73% yield after column chromatography (Hexane/ EtOAc 4:1). **^{1}H NMR** (300 MHz, $CDCl_3$) δ 7.34 – 7.27 (m, 2H), 7.23 – 7.17 (m, 3H), 6.01 (d, *J* = 3.5 Hz, 1H), 5.71 (m, 2H), 5.63 (d, *J* = 3.5 Hz, 1H), 5.11 – 5.02 (m, 2H), 5.02 – 4.94 (m, 2H), 3.00 (q, *J* = 8.5 Hz, 1H), 2.65 (ddd, *J* = 13.4, 9.9, 5.6 Hz, 1H), 2.48 – 2.36 (m, 1H),), 2.31 – 2.19 (m, 1H), 1.96 – 1.85 (m, 2H), 1.32 (s, 12H). **^{13}C NMR** (75 MHz, $CDCl_3$) δ 143.2, 140.8, 140.4, 131.2, 128.5, 128.2, 125.5, 115.3, 114.4, 83.1, 52.9, 50.2, 34.1, 33.4, 24.9, 24.7. **^{11}B NMR** (160 MHz, $CDCl_3$) δ 30.9. **HRMS (APCI)** Calc. for $C_{22}H_{32}BO_2$ $[M+H^+]$: 339.2490, found 339.2493.

4,4,5,5-Tetramethyl-2-(3-phenethyl-5-phenylhexa-1,5-dien-2-yl)-1,3,2-dioxaborolane (22).

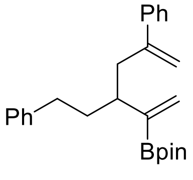

Synthesized from **1**, **52** and B_2pin_2 according to general procedure A. Yellow oil obtained in 68% yield after column chromatography (Hexane/ EtOAc 4:1) **^{1}H NMR** (500 MHz, $CDCl_3$) δ 7.31 – 7.26 (m, 2H), 7.24 – 7.14 (m, 6H), 7.04 – 7.00 (m, 2H), 5.74 (d, *J* = 3.5 Hz, 1H), 5.32 (d, *J* = 3.5 Hz, 1H), 5.14 (d, *J* = 1.9 Hz, 1H), 4.91 (d, *J* = 1.7 Hz, 1H), 2.68 (dd, *J* = 14.1, 8.1 Hz, 1H), 2.61 – 2.53 (m,

1H), 2.52 – 2.38 (m, 1H), 2.33 – 2.25 (m, 1H), 2.25 – 2.12 (m, 1H), 1.83 – 1.75 (m, 1H), 1.74 – 1.67 (m, 1H), 1.21 (s, 6H), 1.20 (s, 6H). **^{13}C NMR** (126 MHz, $CDCl_3$) δ 146.5, 142.1, 140.4, 129.3, 127.3, 127.2, 127.1, 126.1, 125.5, 124.4, 112.8, 82.1, 43.9, 39.9, 34.6, 33.1, 23.8, 23.7. **^{11}B NMR** (160 MHz, $CDCl_3$) δ 30.7. **HRMS (APCI)** Calc. for $C_{26}H_{34}BO_2$ [M+H$^+$]: 389.2646, found 389.2655.

4,4,5,5-Tetramethyl-2-(3-phenethylhexa-1,5-dien-2-yl)-1,3,2-dioxaborolane (23).

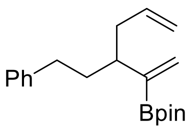

Synthesized from **1**, **65** and B_2pin_2 according to general procedure A. Yellow oil obtained in 78% yield after column chromatography (Hexane/ EtOAc 4:1). **^{1}H NMR** (300 MHz, $CDCl_3$) δ 7.30 – 7.22 (m, 2H), 7.20 – 7.13 (m, 3H), 5.90 (d, *J* = 3.4 Hz, 1H), 5.72 (ddt, *J* = 16.8, 10.2, 6.7 Hz, 1H), 5.60 (d, *J* = 3.4 Hz, 1H), 5.02 – 4.91 (m, 2H), 2.67 – 2.39 (m, 2H), 2.33 – 2.20 (m, 3H), 1.92 – 1.71 (m, 2H), 1.28 (s, 12H). **^{13}C NMR** (75 MHz, $CDCl_3$) δ 143.5, 138.2, 130.2, 128.7, 128.5, 125.8, 115.6, 83.5, 46.1, 39.6, 36.0, 34.2, 25.1, 25.0. **^{11}B NMR** (160 MHz, $CDCl_3$) δ 31.5. **HRMS (APCI)** Calc. for $C_{20}H_{30}BO_2$ [M+H$^+$]: 313.2333, found 313.2334.

4,4,5,5-Tetramethyl-2-(5-methyl-3-phenylhexa-1,5-dien-2-yl)-1,3,2-dioxaborolane (24).

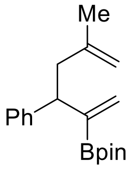

Synthesized from **51**, **59** and B_2pin_2 according to general procedure A. Yellow oil obtained in 55% yield after column chromatography (Hexane/ CH_2Cl_2 90:10 to 80:20). **^{1}H NMR** (500 MHz, $CDCl_3$) δ 7.18 – 7.15 (m, 4H), 7.09 – 7.04 (m, 1H), 5.75 (d, *J* = 2.9 Hz, 1H), 5.53 (d, *J* = 1.5 Hz, 1H), 4.63 – 4.58 (m, 1H), 4.57 – 4.53 (m, 1H), 3.67 (t, *J* = 7.7 Hz, 1H), 2.64 (ddd, *J* = 14.0, 7.6, 1.1 Hz, 1H), 2.40 (ddd, *J* = 14.1, 8.0, 1.1 Hz, 1H), 1.61 (s, 3H), 1.11 (s, 6H), 1.08 (s, 6H). **^{13}C NMR** (126 MHz, $CDCl_3$) δ 144.3, 144.3, 128.5, 128.2, 128.1, 125.9, 112.2, 83.4, 48.7, 42.4, 24.8, 24.8, 22.6. **^{11}B NMR** (160 MHz, CDCl3) δ 30.3. **HRMS (APCI)** Calc. for $C_{19}H_{28}BO_2$ [M+H$^+$]: 299.2177, found 299.2185.

4,4,5,5-Tetramethyl-2-(3-phenylhexa-1,5-dien-2-yl)-1,3,2-dioxaborolane (25).

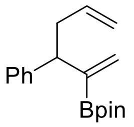

Synthesized from **51**, **65** and B_2pin_2 according to general procedure A. Yellow oil obtained in 61% yield after column chromatography (Hexane/ CH_2Cl_2 90:10 to 80:20). **^{1}H NMR** (300 MHz, $CDCl_3$) δ 7.34 – 7.21 (m, 4H), 7.21 – 7.13 (m, 1H), 5.87 (d, *J* = 2.9 Hz, 1H), 5.82 – 5.69 (m, 1H), 5.65 (d, *J* = 2.9 Hz, 1H), 5.07 – 4.91 (m, 2H), 3.62 (t, *J* = 7.7 Hz, 1H), 2.74 (dt, *J* = 14.2, 7.2 Hz, 1H), 2.57 (dt, *J* = 14.1, 7.4 Hz, 1H), 1.20 (s, 6H), 1.16 (s, 6H). **^{13}C NMR** (75 MHz, $CDCl_3$) δ 143.9, 137.6, 128.4, 128.0, 127.9, 125.9, 115.6, 83.3, 50.4, 38.3, 24.7, 24.5. **^{11}B NMR** (160 MHz, $CDCl_3$) δ 30.1. **HRMS (APCI)** Calc. for $C_{18}H_{26}BO_2$ [M+H$^+$]: 285.2020, found 285.2015.

6. Enantioselective Cu/Pd-catalyzed borylative allyl-allyl coupling

6.1 Screening of chiral ligands

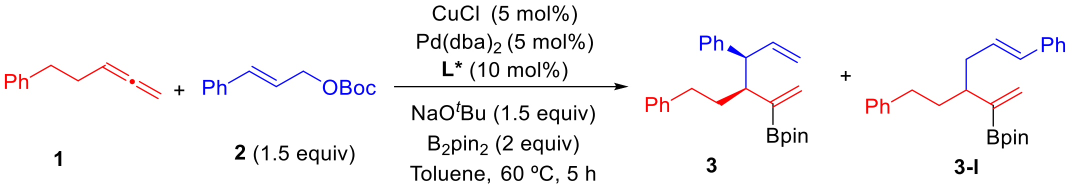

Entry[a]	L*	3 yield, (dr)	3 er	3: 3-l	3-l er
1	L1	33 (>20:1)	97:3[b]	>20:1	-
2	L2	59 (12:1)	8:92[c]	5:1	64:36[c]
3	L3	-	-	-	-
4	L4	32 (11:1)	11:89[c]	1:1	54:46[c]
5	L5	-	-	-	-
6	L6	43 (10:1)	21:79[b]	>20:1	-
7	Quinox P*	-	-	-	-
8	(*R*)-SegPhos	53 (11:1)	9:91[c]	2:1	73:27[c]
9	(*S*)-DM-SegPhos	37 (7:1)	n.d	1:5	32:68[b]
10	*S*-BINAP	49 (13:1)	83:17[b]	4:1	64:36[b]
11	(*S*)-Ph-GarPhos	50 (9:1)	93:7[c]	1:1.5	59:41[c]
12	DuanPhos	-		-	-
13[d]	L1	54% (>20:1)	98.5:1.5	>20:1	-

Table S8. [a] Reactions performed on a 0.2mmol scale. [b] Enantiomeric ratio was measured using HPLC analysis in the oxidized ketone product [c] Enantiomeric ratio was measured using SFC analysis. [d] Reaction run at 30 °C during 18 h.

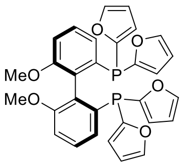

L1

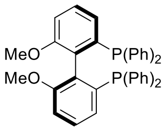

L2

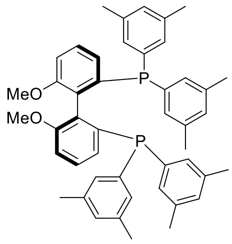

L3

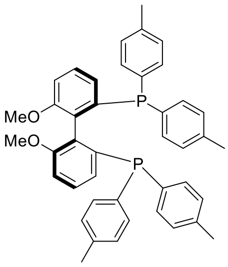

L4

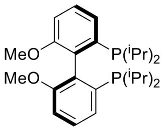

L5

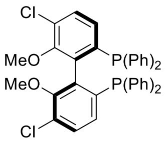

L6

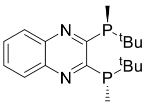

QUINOX P*

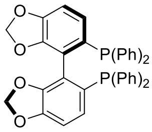

(*R*)-SEGPHOS

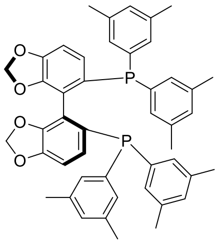

(*S*)-DM-SegPhos

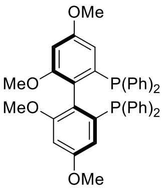

(*S*)-Ph-GarPhos

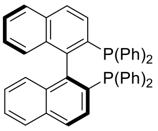

(*S*)-BINAP

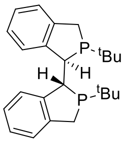

DuanPhos

6.2 Experiments with different combinations of Cu and Pd complexes ($L_{Cu} \neq L_{Pd}$)

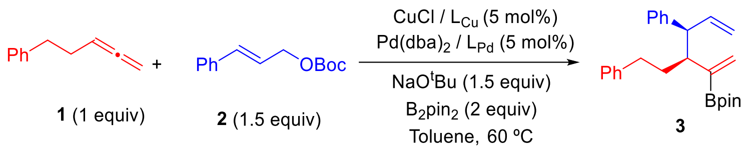

Entry[a]	L_{Cu}	L_{Pd}	3 yield (%)[b]	dr	er
**1**	**L1**	**L1**	33	>20:1	97:3[c]
**2**	BIPHEP	**L1**	38	13:1	66:34[d]
**3**	**L1**	BIPHEP	57	>20:1	49:51[d]
**4**	IMes	**L1**	59	5:1	87:13[d]
**5**	**L1**	***enant*-L1**	35	>20:1	62:38[d]
**6**	***enant*-L1**	**L1**	32	>20:1	56:44[d]

Table S9. [a] Reactions performed in a 0.2 mmol scale. Each transition metal complex was preformed separately prior to the reaction (see general procedure for optimization studies). [b] Yield of isolated product. [c] Determined by HPLC analysis after oxidation of **3**. [d] Determined by SFC analysis of **3**.

Conclusion: Results in Table S9 highlight the requirement of using the same chiral ligand at both complexes and suggest that ligand scrambling may be operating under these reaction conditions.

6.3 General procedure for the enantioselective Cu/Pd-catalyzed borylative allyl-allyl coupling (General Procedure B)

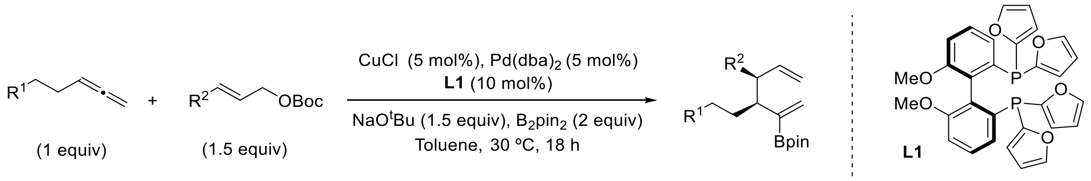

In a Schlenk flask which contains B_2pin_2 (2 equiv, 0.8 mmol), NaO^tBu (1.5 equiv, 0.6 mmol), CuCl (5 mol%, 0.02 mmol), $Pd(dba)_2$ (5 mol%, 0.02 mmol) and **L1** (10 mol%, 0.04mmol) was added a solution of the corresponding allene (0.4 mmol) in 2 ml of dry toluene and allyl carbonate (1.5 equiv, 0.6 mmol) in 4 ml of dry toluene. The final solution was stirred at 30 °C for 18 hours. After this time, the final mixture was filtered through a small amount of silica and solvent was removed under vacuum. The final product was purified by silica gel column chromatography.

6.4 Characterization of enantioenriched compounds

4,4,5,5-Tetramethyl-2-((3*S*,4*S*)-3-phenethyl-4-phenylhexa-1,5-dien-2-yl)-1,3,2-dioxaborolane ((-)-3).

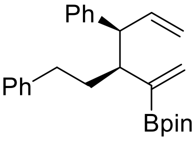

Synthesized from **1**, **2** and B_2pin_2 according to general procedure B. Yellow oil obtained in 59% yield with 98.5:1.5 er after column chromatography (Hexane/ EtOAc 4:1). **^{1}H NMR** (300 MHz, $CDCl_3$) δ 7.38 – 7.13 (m, 10H), 6.14 – 5.93 (m, 1H), 5.84 (d, *J* = 3.4 Hz, 1H), 5.49 (d, *J* = 3.4 Hz, 1H), 5.18 – 5.06 (m, 2H), 3.61 (t, *J* = 9.7 Hz, 1H), 2.74 – 2.42 (m, 3H), 2.20 – 1.85 (m, 2H), 1.33 (s, 6H), 1.30 (s, 6H). **^{13}C NMR** (75 MHz, $CDCl_3$) δ 144.3, 143.2, 141.8, 132.1, 128.5, 128.4, 128.3, 128.2, 125.6, 125.5, 115.3, 83.1, 55.4, 51.0, 34.3, 34.2, 24.8, 24.7. **^{11}B NMR** (160 MHz, $CDCl_3$) δ 30.5. **HRMS (APCI)** Calc. for $C_{28}H_{34}BO_2$ [M+H$^+$]: 389.2646, found 389.2657. **Specific rotation:** $[\alpha]_D^{21}$ -24.3 (*c*=1.1, $CHCl_3$).

Enantiomeric purity was determined on the derived ketone **29** (see section 7.3) by chiral uHPLC analysis [chiralpak IB N-3, T_{oven}: 40 °C, flow: 1 mL/min; 99:1 hexane/*i*PrOH, λ = 226 nm minor enantiomer t_R = 2.10 min, major enantiomer t_R = 1.83 min].

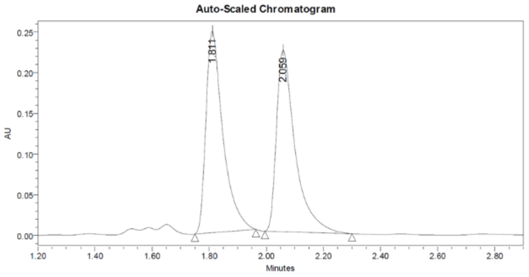

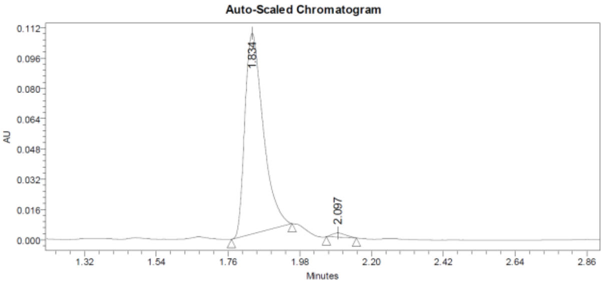

Unknown Peak Results

	RT	Area	% Area	Height
1	1.811	1025972	50.32	247593
2	2.059	1012831	49.68	222948

Unknown Peak Results

	RT	Area	% Area	Height
1	1.834	415840	98.46	105897
2	2.097	6488	1.54	2189

4,4,5,5-Tetramethyl-2-((*S*)-3-((*S*)-1-phenylallyl)undec-1-en-2-yl)-1,3,2-dioxaborolane ((-)-4).

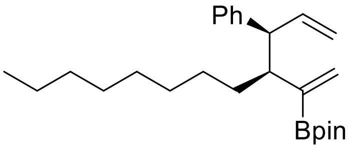

Synthesized from **44**, **2** and B_2pin_2 according to general procedure B. Yellow oil obtained in 39% yield with 97:3 er after column chromatography (Hexane/ EtOAc 4:1). **^{1}H NMR** (300 MHz, $CDCl_3$) δ 7.36 – 7.28 (m, 1H), 7.19 (t, *J* = 7.5 Hz, 2H), 7.09 (d, *J* = 7.3 Hz, 2H), 5.95 (dt, *J* = 17.2, 9.7 Hz, 1H), 5.65 (d, *J* = 3.5 Hz, 1H), 5.33 (d, *J* = 3.5 Hz, 1H), 5.10 – 4.94 (m, 2H), 3.48 (t, *J* = 9.8 Hz, 1H), 2.47 (td, *J* = 10.7, 3.4 Hz, 1H), 1.71 – 1.60 (m, 1H), 1.50 – 1.40 (m, 1H), 1.29 – 1.23 (m, 12H), 1.22 (s, 6H), 1.19 (s, 6H), 0.87 (t, *J* = 6.5 Hz, 3H). **^{13}C NMR** (75 MHz, $CDCl_3$) δ 144.5, 142.1, 131.3, 128.4, 128.1, 125.6, 114.9, 82.9, 55.4, 50.9, 32.1, 31.9, 29.7, 29.6, 29.4, 27.7, 24.9, 24.6, 22.7, 14.1. **^{11}B NMR** (160 MHz, $CDCl_3$) δ 30.5, **HRMS (APCI)** Calc. for $C_{26}H_{41}BO_2$[M+H$^+$]: 397.3278, found 397.3299. **Specific rotation:** $[\alpha]_D^{19}$ -15.6 (*c*=1.0, $CHCl_3$).

Enantiomeric purity was determined by chiral SFC analysis [Lux Cellulose-1, 100 bar, T_{oven}: 40 °C, flow: 2 mL/min; 1% MeOH, λ = 220 nm, minor enantiomer t_R = 11.79 min, major enantiomer t_R = 10.44 min].

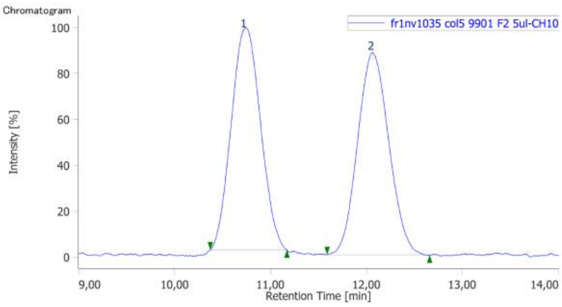

Peak Information

#	Peak Name	CH	tR [min]	Area [µV·sec]	Area%
1	Unknown	10	10,737	570255	50,818
2	Unknown	10	12,063	551906	49,182

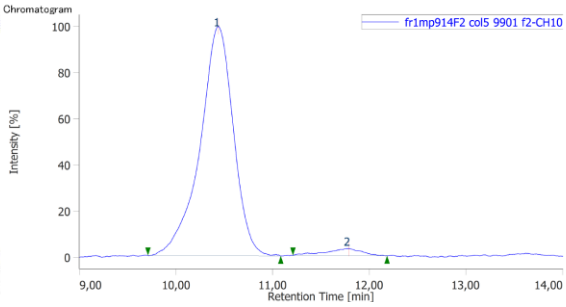

Peak Information

#	Peak Name	CH	tR [min]	Area [µV·sec]	Area%
1	Unknown	10	10,440	1438415	96,856
2	Unknown	10	11,787	46687	3,144

***tert*-Butyldimethyl(((3*S*,4*S*)-4-phenyl-3-(1-(4,4,5,5-tetramethyl-1,3,2-dioxaborolan-2-yl)vinyl)hex-5-en-1-yl)oxy)silane ((-)-6).**

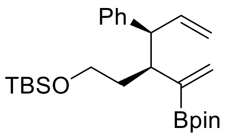

Synthesized from **46**, **2** and B_2pin_2 according to general procedure B. Yellow oil obtained in 46% yield with 96.5:3.5 er after column chromatography (Hexane/ EtOAc 4:1). **^{1}H NMR** (300 MHz, $CDCl_3$) δ 7.29 – 7.19 (m, 2H), 7.15 – 7.07 (m, 3H), 6.03 – 5.88 (m, 1H), 5.68 (d, *J* = 3.5 Hz, 1H), 5.39 (d, *J* = 3.5 Hz, 1H), 5.15 – 5.02 (m, 2H), 3.66 – 3.40 (m, 3H), 2.64 (td, *J* = 10.7, 3.1 Hz, 1H), 2.00 (dtd, *J* = 13.4, 8.0, 3.1 Hz, 1H), 1.80 – 1.62 (m, 1H), 1.25 (s, 6H), 1.21 (s, 6H), 0.92 (s, 9H), 0.05 (d, *J* = 1.9 Hz, 6H). **^{13}C NMR** (75 MHz, $CDCl_3$) δ 144.2, 141.7, 131.7, 128.3, 128.1, 125.7, 115.2, 83.0, 61.8, 54.9, 47.7, 35.0, 26.0, 24.8, 24.6, 18.3, -5.2. **^{11}B NMR** (160 MHz, $CDCl_3$) δ 31.3. **HRMS (APCI)** Calc. for $C_{26}H_{44}BO_3Si$ [M+H$^+$]: 443.3147, found 443.3157. **Specific rotation:** $[\alpha]_D^{19}$ -13.1 (*c*=0.91, $CHCl_3$).

Enantiomeric purity was determined on the derived methylene cyclobutane **41** (see section 7.5) by chiral SFC analysis [Lux Amylose-2, 100 bar, T_{oven}: 40 °C, flow: 2 mL/min; 2% MeOH, λ = 220 nm, minor enantiomer t_R = 24.50 min, major enantiomer t_R = 32.15 min].

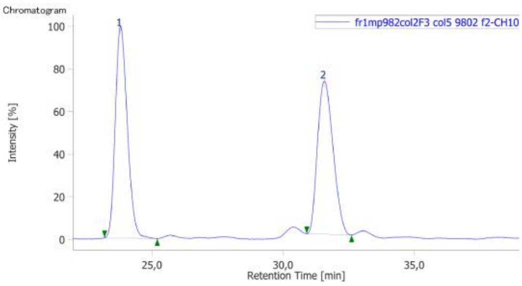

Peak Information

#	Peak Name	CH	tR [min]	Area [µV·sec]	Area%
1	Unknown	10	23,803	3770899	51,595
2	Unknown	10	31,577	3537777	48,405

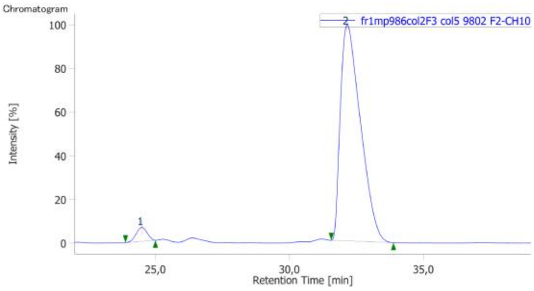

Peak Information

#	Peak Name	CH	tR [min]	Area [µV·sec]	Area%
1	Unknown	10	24,500	572017	3,416
2	Unknown	10	32,153	16172960	96,584

2-((3*S*,4*S*)-3-(3-(Benzyloxy)propyl)-4-phenylhexa-1,5-dien-2-yl)-4,4,5,5-tetramethyl-1,3,2-dioxaborolane ((-)-8).

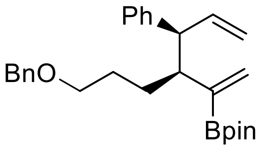

Synthesized from **48**, **2** and B_2pin_2 according to general procedure B. Yellow oil obtained in 30% yield with 94:6 er after column chromatography (Hexane/ EtOAc 4:1). **^{1}H NMR** (300 MHz, $CDCl_3$) δ 7.42 – 7.23 (m, 7H), 7.18 – 7.11 (m, 3H), 6.01 (dt, *J* = 17.0, 9.6 Hz, 1H), 5.74 (d, *J* = 3.4 Hz, 1H), 5.41 (d, *J* = 3.4 Hz, 1H), 5.20 – 5.03 (m, 2H), 4.54 (s, 2H), 3.61 – 3.48 (m, 3H), 2.56 (td, *J* = 10.4, 3.3 Hz, 1H), 1.94 – 1.83 (m, 1H), 1.71 – 1.50 (m, 3H), 1.27 (s, 6H), 1.24 (s, 6H). **^{13}C NMR** (75 MHz, $CDCl_3$) δ 144.3, 141.9, 138.9, 131.7, 128.4, 128.3, 128.2, 127.6, 127.4, 125.7, 115.1, 83.0, 72.7, 70.5, 55.5, 50.7, 28.6, 27.9, 24.9, 24.6. **^{11}B NMR** (160 MHz, $CDCl_3$) δ 31.7. **HRMS (APCI)** Calc. for $C_{28}H_{38}BO_3$ [M+H$^+$]: 433.2909, found 433.2917. **Specific rotation:** $[\alpha]_D^{20}$ -7.7 (*c*=0.96, $CHCl_3$).

Enantiomeric purity was determined by chiral SFC analysis [Lux Cellulose-1, 100 bar, T_{oven}: 40 °C, flow: 1 mL/min; 2% MeOH, λ = 220 nm, minor enantiomer t_R = 59.97 min, major enantiomer t_R = 56.33 min].

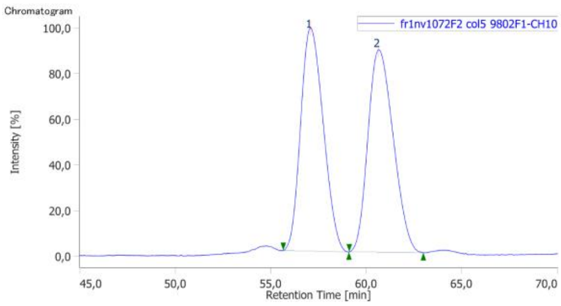

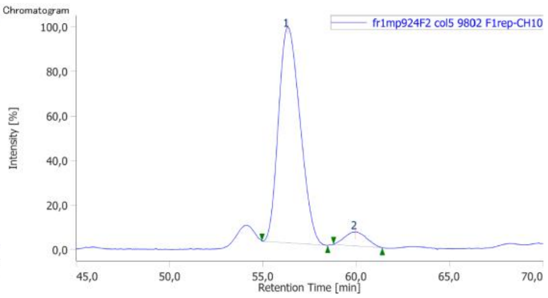

Peak Information

#	Peak Name	CH	tR [min]	Area [µV·sec]	Area%
1	Unknown	10	57,080	4029418	49,646
2	Unknown	10	60,640	4086889	50,354

Peak Information

#	Peak Name	CH	tR [min]	Area [µV·sec]	Area%
1	Unknown	10	56,327	4000363	93,840
2	Unknown	10	59,973	262595	6,160

***tert*-Butyl benzyl((4*S*,5*S*)-5-phenyl-4-(1-(4,4,5,5-tetramethyl-1,3,2-dioxaborolan-2-yl)vinyl)hept-6-en-1-yl)carbamate ((-)-10).**

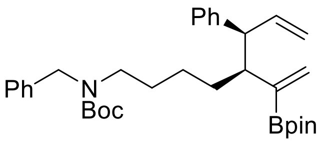

Synthesized from **50**, **2** and B_2pin_2 according to general procedure B. Yellow oil obtained in 40% yield with 96:4 er after column chromatography (Hexane/ EtOAc 7:3). **^{1}H NMR** (300 MHz, $CDCl_3$) δ 7.37 – 7.17 (m, 8H), 7.14 – 7.07 (m, 2H), 5.94 (dt, *J* = 17.0, 9.7 Hz, 1H), 5.66 (d, *J* = 3.4 Hz, 1H), 5.33 (d, *J* = 3.5 Hz, 1H), 5.11 – 4.99 (m, 2H), 4.43 (bs, 2H), 3.47 (t, *J* = 9.8 Hz, 1H), 3.11 (bs, 2H), 2.45 (td, *J* = 10.6, 3.4 Hz, 1H), 1.48 (bs, 15H), 1.23 (s, 6H), 1.19 (s, 6H). **^{13}C NMR** (126 MHz, $CDCl_3$) δ 143.3, 142.6, 140.9, 137.6, 130.5, 127.4, 127.3, 127.1, 126.7, 126.0, 124.7, 114.0, 82.2, 82.0, 54.4, 49.4, 48.8, 45.5, 30.7, 27.5, 24.0, 23.8, 23.6, 23.5. **^{11}B NMR** (160 MHz, $CDCl_3$) δ 30.4. **HRMS (APCI)** Calc. for $C_{34}H_{49}BNO_4$ [M+H$^+$]: 546.3749, found 546.3760. **Specific rotation:** $[\alpha]_D^{19}$ -8.8 (*c*=0.95, $CHCl_3$).

Enantiomeric purity was determined by chiral SFC analysis [Lux Cellulose-1, 100 bar, T_{oven}: 40 °C, flow: 2 mL/min; 5% MeOH, λ = 220 nm, minor enantiomer t_R = 15.23 min, major enantiomer t_R = 14.15 min].

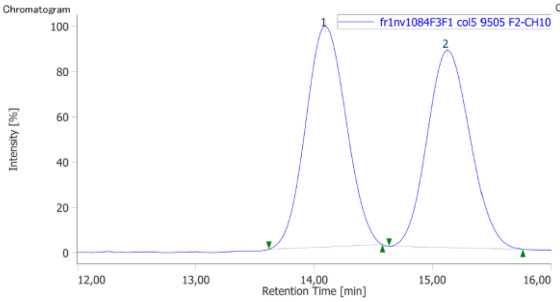

Peak Information

#	Peak Name	CH	tR [min]	Area [μV·sec]	Area%
1	Unknown	10	14,093	1790207	51,450
2	Unknown	10	15,123	1689272	48,550

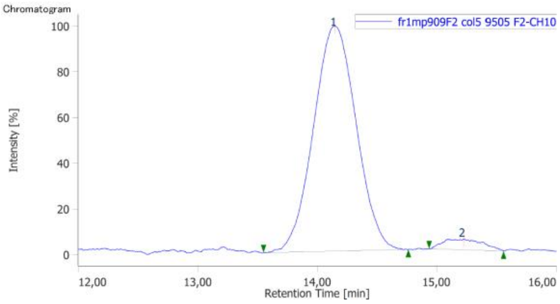

Peak Information

#	Peak Name	CH	tR [min]	Area [μV·sec]	Area%
1	Unknown	10	14,150	438407	95,791
2	Unknown	10	15,227	19262	4,209

4,4,5,5-Tetramethyl-2-((3*S*,4*S*)-3-phenethyl-4-(4-(trifluoromethyl)phenyl)hexa-1,5-dien-2-yl)-1,3,2-dioxaborolane ((-)-11).

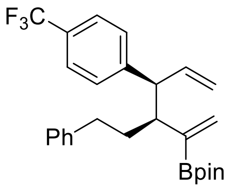

Synthesized from **1**, **53** and B_2pin_2 according to general procedure B at 60 °C over 5 h. Yellow oil obtained in 51% yield with 96:4 er after column chromatography (Hexane/ EtOAc 4:1). **^{1}H NMR** (300 MHz, $CDCl_3$) δ 7.47 (d, *J* = 8.4 Hz, 2H), 7.27 (d, *J* = 7.3 Hz, 2H), 7.24 – 7.12 (m, 5H), 5.90 (dt, *J* = 17.0, 9.7 Hz, 1H), 5.78 (d, *J* = 3.3 Hz, 1H), 5.44 (d, *J* = 3.3 Hz, 1H), 5.14 – 5.00 (m, 2H), 3.59 (t, *J* = 9.8 Hz, 1H), 2.69 – 2.50 (m, 2H), 2.46 – 2.33 (m, 1H), 2.12 – 1.97 (m, 1H), 1.92 – 1.74 (m, 1H), 1.25 (s, 6H), 1.22 (s, 6H). **^{13}C NMR** (75 MHz, $CDCl_3$) δ 148.7, 143.2, 141.1, 132.8, 129.0, 128.8, 128.6, 128.1, 125.9, 125.4 (q, *J* = 4.5 Hz), 124.6 (q, *J* = 262.4 Hz) 116.4, 83.6, 55.6, 51.0, 34.5, 34.4, 25.1, 24.9. **^{19}F NMR** (282 MHz, $CDCl_3$) δ -62.05. **^{11}B NMR** (160 MHz, $CDCl_3$) δ 31.7. **HRMS (APCI)** Calc. for $C_{27}H_{33}BF_3O_2$ [M+H$^+$]: 457.2520, found 457.2522. **Specific rotation:** $[\alpha]_D^{22}$ -56.9 (*c*=0.50, $CHCl_3$).

Enantiomeric purity was determined on the derived ketone **30** (see section 7.3) by chiral SFC analysis [Lux Cellulose-1, 100 bar, T_{oven}: 40 °C, flow: 1 mL/min; 3% MeOH, λ = 220 nm minor enantiomer t_R = 17.33 min, major enantiomer t_R = 14.87 min].

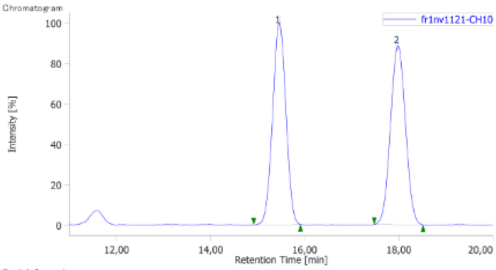

Peak Information

#	Peak Name	CH	tR [min]	Area [μV·sec]	Area%
1	Unknown	10	15,457	1275563	50,057
2	Unknown	10	17,977	1272660	49,943

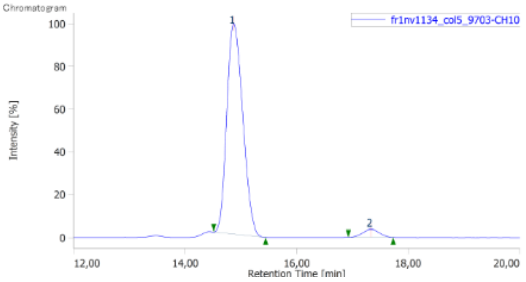

Peak Information

#	Peak Name	CH	tR [min]	Area [μV·sec]	Area%
1	Unknown	10	14,867	17755003	96,315
2	Unknown	10	17,330	679298	3,685

tert-Butyldimethyl(((3*S*,4*S*)-3-(1-(4,4,5,5-tetramethyl-1,3,2-dioxaborolan-2-yl)vinyl)-4-(4-(trifluoromethyl)phenyl)hex-5-en-1-yl)oxy)silane ((-)-12).

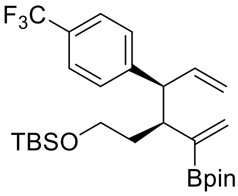

Synthesized from **46**, **53** and B_2pin_2 according to general procedure B. Yellow oil obtained in 42% yield with 90:10 er after column chromatography (Hexane/ EtOAc 7:3). **^{1}H NMR** (500 MHz, $CDCl_3$) δ 7.48 (d, *J* = 8.6 Hz, 2H), 7.23 (d, *J* = 7.9 Hz, 2H), 5.94 (ddd, *J* = 16.9, 10.1, 9.1 Hz, 1H), 5.71 (d, *J* = 3.4 Hz, 1H), 5.42 (d, *J* = 3.4 Hz, 1H), 5.17 – 5.06 (m, 2H), 3.59 – 3.50 (m, 2H), 3.48 – 3.37 (m, 1H), 2.68 (td, *J* = 10.8, 3.0 Hz, 1H), 2.00 – 1.90 (m, 1H), 1.70 – 1.59 (m, 1H), 1.24 (s, 6H), 1.21 (s, 6H), 0.95 – 0.90 (s, 9H), 0.00 (s, 3H), -0.01 (s, 3H). **^{13}C NMR** (75 MHz, $CDCl_3$) δ 148.4, 140.8, 132.2, 128.7, 128.0 (q, *J* = 32.6 Hz), 125.0 (q, *J* = 3.8 Hz), 124.4 (q, *J* = 267.4 Hz), 116.1, 83.1, 61.5, 54.8, 47.4, 35.0, 26.0, 24.8, 24.6, 18.3, -5.2, -5.3. **^{19}F NMR** (282 MHz, $CDCl_3$) δ -62.07. **^{11}B NMR** (160 MHz, $CDCl_3$) δ 30.2. **HRMS (APCI)** Calc. for $C_{27}H_{43}BF_3O_3Si$ [M+H$^+$]: 511.2936, found 511.2934. **Specific rotation:** $[\alpha]_D^{22}$ -18.7 (*c*=1.0, $CHCl_3$).

Enantiomeric purity was determined on the derived ketone **31** (see section 7.3) by chiral uHPLC analysis [Lux i-Cellulose-5, T_{oven}: 40 °C, flow: 1 mL/min; 99.5:0.5 hexane/*i*PrOH, λ = 220 nm minor enantiomer t_R = 4.40 min, major enantiomer t_R = 4.21 min].

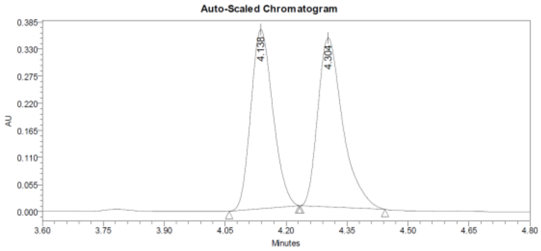

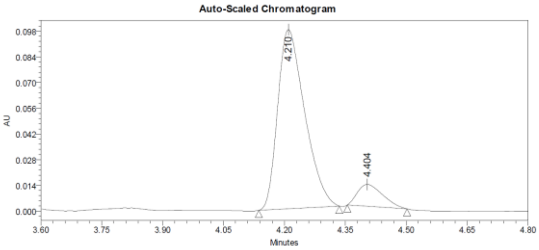

Unknown Peak Results

	RT	Area	% Area	Height
1	4.138	1376933	48.42	363777
2	4.304	1466884	51.58	343438

Unknown Peak Results

	RT	Area	% Area	Height
1	4.210	451377	89.73	97689
2	4.404	51679	10.27	11884

2-((3*S*,4*S*)-4-(3-Chlorophenyl)-3-phenethylhexa-1,5-dien-2-yl)-4,4,5,5-tetramethyl-1,3,2-dioxaborolane ((-)-13).

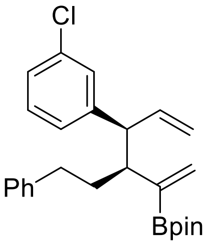

Synthesized from **1**, **54** and B_2pin_2 according to general procedure B. Yellow oil obtained in 43% yield with 96:4 er after column chromatography (Hexane/ EtOAc 4:1). **^{1}H NMR** (300 MHz, $CDCl_3$) δ 7.34 – 7.25 (m, 2H), 7.23 – 7.07 (m, 6H), 6.96 (d, *J* = 7.4 Hz, 1H), 5.88 (dt, *J* = 16.7, 9.6 Hz, 1H), 5.77 (d, *J* = 3.3 Hz, 1H), 5.43 (d, *J* = 3.4 Hz, 1H), 5.12 – 5.01 (m, 2H), 3.52 (t, *J* = 9.9 Hz, 1H), 2.70 – 2.33 (m, 3H), 2.13 – 1.97 (m, 1H), 1.89 – 1.73 (m, 1H), 1.28 (s, 6H), 1.24 (s, 6H). **^{13}C NMR** (75 MHz, $CDCl_3$) δ 146.7, 143.3, 141.3, 134.1, 132.8, 129.7, 128.9, 128.8, 128.6, 126.7, 126.2, 125.9, 116.2, 83.5, 55.3, 51.3, 34.4, 25.3, 25.1, 24.9. **^{11}B NMR** (160 MHz, $CDCl_3$) δ 31.5. **HRMS (APCI)** Calc. for $C_{26}H_{33}BClO_2$ [M+H$^+$]: 423.2257, found 423.2272. **Specific rotation:** $[\alpha]_D^{21}$ -13.6 (*c*=1.0, $CHCl_3$).

Enantiomeric purity was determined on the derived ketone **32** (see section 7.3) by chiral uHPLC analysis [Lux i-Amylose-3, T_{oven}: 40 °C, flow: 0.5 mL/min; 99:1 hexane/*i*PrOH, λ = 209.5 nm minor enantiomer t_R = 22.85 min, major enantiomer t_R = 21.70 min].

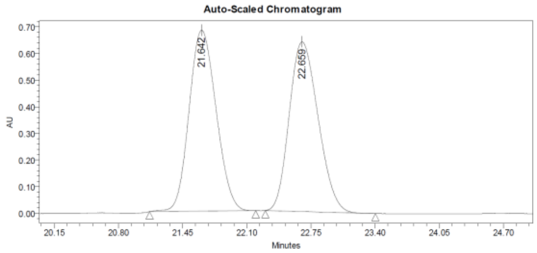

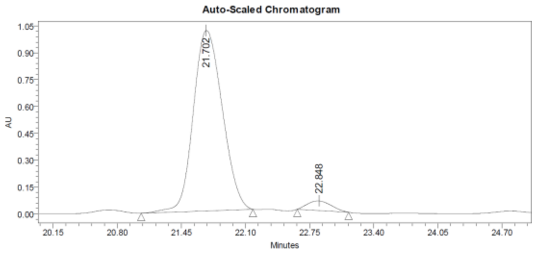

Unknown Peak Results

	RT	Area	% Area	Height
1	21.642	12937724	49.94	679633
2	22.659	12966488	50.06	637302

Unknown Peak Results

	RT	Area	% Area	Height
1	21.702	20594098	95.84	1007189
2	22.848	894008	4.16	53372

2-((3*S*,4*S*)-4-(2-Fluorophenyl)-3-phenethylhexa-1,5-dien-2-yl)-4,4,5,5-tetramethyl-1,3,2-dioxaborolane ((-)-14).

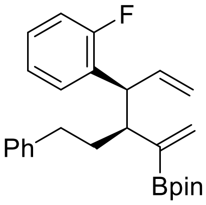

Synthesized from **1**, **55** and B_2pin_2 according to general procedure C. Yellow oil obtained in 42% yield with 96.5:3.5 er (5:1 dr, 5:1 rr) after column chromatography (Hexane/ EtOAc 4:1). **^{1}H NMR** (300 MHz, $CDCl_3$) δ 7.35 – 7.23 (m, 2H), 7.23 – 7.02 (m, 5H), 7.04 – 6.84 (m, 2H), 6.08 – 5.90 (m, 1H), 5.74 (d, *J* = 3.3 Hz, 1H), 5.49 (d, *J* = 3.6 Hz, 1H), 5.17 – 4.98 (m, 2H), 3.79 (t, *J* = 10.0 Hz, 1H), 2.73 – 2.53 (m, 2H), 2.48 – 2.32 (m, 1H), 2.11 – 1.97 (m, 1H), 1.97 – 1.81 (m, 1H), 1.26 (s, 6H), 1.22 (s, 6H). **^{13}C NMR** (75 MHz, $CDCl_3$) δ 160.6 (d, *J* = 237.9 Hz), 143.2, 140.1, 132.1, 130.1 (d, *J* = 5.8 Hz), 128.4, 128.2, 128.1, 127.3 (d, *J* = 8.4 Hz), 125.5, 123.7 (d, *J* = 3.2 Hz) , 116.0, 115.3 (d, *J* = 23.1 Hz), 83.1, 50.0, 49.9, 49.7, 34.3, 24.9, 24.5. **^{19}F NMR** (282 MHz, $CDCl_3$) δ -116.13. **^{11}B NMR** (160 MHz, $CDCl_3$) δ 31.3. **HRMS (APCI)** Calc. for $C_{26}H_{33}BFO_2$ [M+H$^+$]: 407.3525, found 407.3521. **Specific rotation:** $[\alpha]_D^{23}$ -13.5 (*c*=0.78, $CHCl_3$).

Enantiomeric purity was determined by chiral SFC analysis [Lux Cellulose-1, 100 bar, T_{oven}: 40 °C, flow: 1.5 mL/min; 1% MeOH, λ = 220 nm, minor enantiomer t_R = 27.42 min, major enantiomer t_R = 25.39 min].

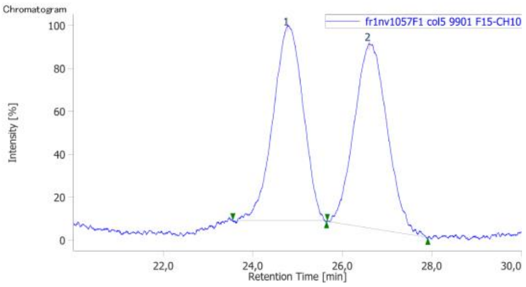

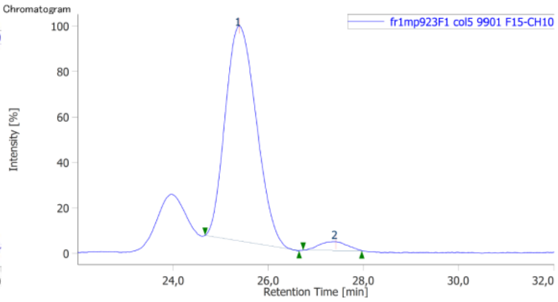

Peak Information

#	Peak Name	CH	tR [min]	Area [μV·sec]	Area%
1	Unknown	10	24,777	361955	49,037
2	Unknown	10	26,597	376174	50,963

Peak Information

#	Peak Name	CH	tR [min]	Area [μV·sec]	Area%
1	Unknown	10	25,393	3472169	96,443
2	Unknown	10	27,423	128045	3,557

2-((3*S*,4*S*)-4-(4-Methoxyphenyl)-3-phenethylhexa-1,5-dien-2-yl)-4,4,5,5-tetramethyl-1,3,2-dioxaborolane ((-)-15).

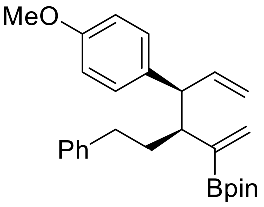

Synthesized from **1**, **56** and B_2pin_2 according to general procedure B. Yellow oil obtained in 51% yield with 93.5:6.5 er (3:1 rr) after column chromatography (Hexane/ EtOAc 4:1). **^{1}H NMR** (300 MHz, $CDCl_3$) δ 7.27 (d, *J* = 7.1 Hz, 2H), 7.22 – 7.14 (m, 3H), 7.01 (d, *J* = 8.6 Hz, 2H), 6.77 (d, *J* = 8.6 Hz, 2H), 5.92 (dt, *J* = 16.9, 9.5 Hz, 1H), 5.77 (d, *J* = 3.5 Hz, 1H), 5.42 (d, *J* = 3.4 Hz, 1H), 5.11 – 4.95 (m, 2H), 3.77 (s, 3H), 3.49 (t, *J* = 9.8 Hz, 1H), 2.76 – 2.33 (m, 3H), 2.11 – 1.95 (m, 1H), 1.93 – 1.78 (m, 1H), 1.27 (s, 6H), 1.24 (s, 6H). **^{13}C NMR** (75 MHz, $CDCl_3$) δ 157.9, 143.5, 142.3, 136.7, 132.3, 129.5, 128.8, 128.5, 125.8, 115.2, 113.9, 83.4, 55.4, 54.7, 51.4, 34.6, 34.5, 25.2, 25.0. **^{11}B NMR** (160 MHz, $CDCl_3$) δ 31.7. **HRMS (APCI)** Calc. for $C_{27}H_{36}BO_3$ [M+H$^+$]: 419.2752, found 419.2755. **Specific rotation:** $[\alpha]_D^{21}$ -9.9 (*c*=1.0, $CHCl_3$).

Enantiomeric purity was determined on the derived ketone **33** (see section 7.3) by chiral SFC analysis [Lux Cellulose-1, 100 bar, T_{oven}: 40 °C, flow: 1 mL/min; 5% MeOH, λ = 220 nm, minor enantiomer t_R = 27.19 min, major enantiomer t_R = 24.14 min].

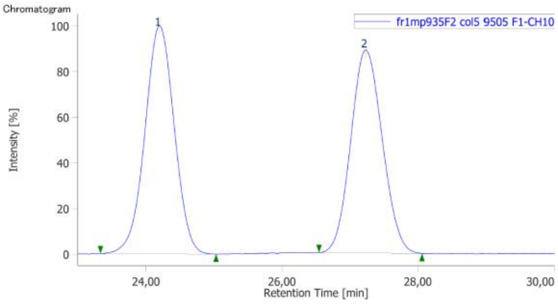

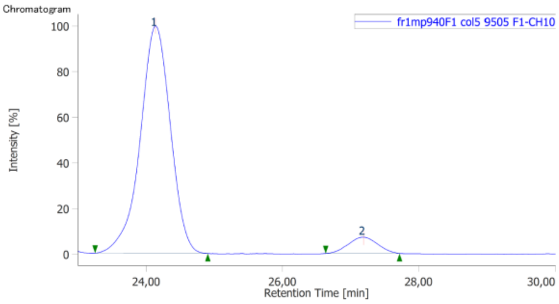

Peak Information

#	Peak Name	CH	tR [min]	Area [μV·sec]	Area%
1	Unknown	10	24,197	3471005	51,145
2	Unknown	10	27,230	3315571	48,855

Peak Information

#	Peak Name	CH	tR [min]	Area [μV·sec]	Area%
1	Unknown	10	24,137	5211464	93,523
2	Unknown	10	27,190	360923	6,477

2-((3*S*,4*S*)-4-(4-Bromophenyl)-3-phenethylhexa-1,5-dien-2-yl)-4,4,5,5-tetramethyl-1,3,2-dioxaborolane ((-)-26).

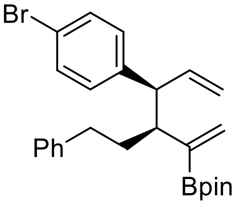

Synthesized from **1**, **57** and B_2pin_2 according to general procedure B. Yellow oil obtained in 44% yield with 98.5:1.5 er after column chromatography (Hexane/ EtOAc 4:1). **^{1}H NMR** (500 MHz, $CDCl_3$) δ 7.24 (d, *J* = 8.3 Hz, 2H), 7.21 – 7.15 (m, 2H), 7.11 – 7.04 (m, 3H), 6.88 (d, *J* = 8.4 Hz, 2H), 5.78 (ddd, *J* = 17.1, 10.1, 9.2 Hz, 1H), 5.69 (d, *J* = 3.4 Hz, 1H), 5.32 (d, *J* = 3.3 Hz, 1H), 5.00 – 4.90 (m, 2H), 3.41 (t, *J* = 9.8 Hz, 1H), 2.52 (ddd, *J* = 13.8, 10.8, 4.7 Hz, 1H), 2.41 (td, *J* = 10.8, 3.2 Hz, 1H), 2.30 (ddd, *J* = 13.8, 10.6, 6.6 Hz, 1H), 1.94 (dddd, *J* = 13.9, 10.9, 6.6, 3.2 Hz, 1H), 1.73 (dtd, *J* = 13.5, 10.8, 4.8 Hz, 1H), 1.17 (s, 6H), 1.14 (s, 6H). **^{13}C NMR** (126 MHz, $CDCl_3$) δ 143.4, 143.1, 141.3, 132.5, 131.3, 130.2, 128.6, 128.4, 125.7, 119.6, 115.8, 83.3, 54.8, 51.0, 34.2, 34.2, 25.0, 24.8. **^{11}B NMR** (160 MHz, $CDCl_3$) δ 29.58. **HRMS**

(APCI) Calc. for $C_{26}H_{33}BBrO_2$ [M+H$^+$]: 467.1751, found 467.1757. **Specific rotation:** $[\alpha]_D^{21}$ -16.7 (*c*=0.81, $CHCl_3$).

Enantiomeric purity was determined on the derived ketone **38** (see section 7.3) by chiral uHPLC analysis [Lux i-Cellulose-5, T_{oven}: 40 °C, flow: 1 mL/min; 99:1 hexane/*i*PrOH, λ = 209.5 nm, minor enantiomer t_R = 9.10 min, major enantiomer t_R = 8.71 min].

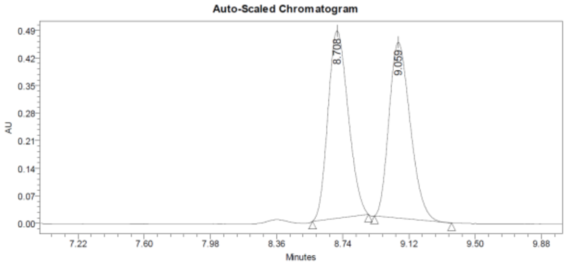

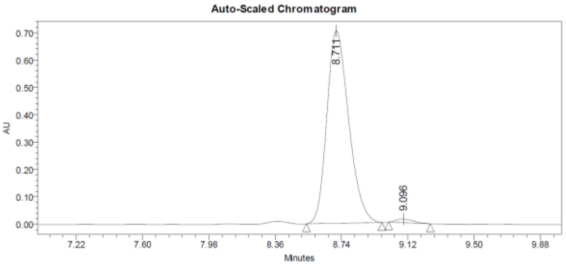

Unknown Peak Results

	RT	Area	% Area	Height
1	8.708	3845036	50.32	475384
2	9.059	3795981	49.68	445990

Unknown Peak Results

	RT	Area	% Area	Height
1	8.711	6146983	98.54	703621
2	9.096	91222	1.46	13647

2-((3*S*,4*S*)-4-((Benzyloxy)methyl)-3-phenethylhexa-1,5-dien-2-yl)-4,4,5,5-tetramethyl-1,3,2-dioxaborolane ((-)-18).

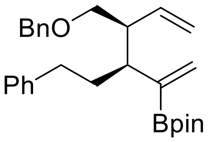

Synthesized from **1**, **61** and B_2pin_2 according to general procedure B. Yellow oil obtained in 50% yield with 95:5 er after column chromatography (Hexane/EtOAc 4:1). **^{1}H NMR** (300 MHz, $CDCl_3$) δ 7.37 – 7.23 (m, 7H), 7.20 – 7.13 (m, 3H), 5.95 (d, *J* = 3.6 Hz, 1H), 5.79 – 5.62 (m, 2H), 5.17 – 5.07 (m, 2H), 4.46 (d, *J* = 3.6 Hz, 2H), 3.45 (dd, *J* = 9.4, 4.5 Hz, 1H), 3.34 (dd, *J* = 9.4, 7.2 Hz, 1H)., 2.66 – 2.53 (m, 2H), 2.42 – 2.18 (m, 2H), 1.95 – 1.74 (m, 2H), 1.28 (s, 12H). **^{13}C NMR** (75 MHz, $CDCl_3$) δ 143.2, 140.1, 138.9, 131.2, 128.4, 128.2, 128.2, 127.5, 127.3, 125.4, 116.5, 83.2, 72.9, 72.8, 48.1, 47.7, 34.1, 33.5, 24.8, 24.7. **^{11}B NMR** (160 MHz, $CDCl_3$) δ 30.2. **HRMS (APCI)** Calc. for $C_{28}H_{38}BO_3$ [M+H$^+$]: 433.2909, found 433.2920. **Specific rotation:** $[\alpha]_D^{23}$ -3.0 (*c*=0.97, $CHCl_3$).

Enantiomeric purity was determined on the derived ketone **35** (see Section 6.2) by chiral uHPLC analysis [chiralpak IB N-3, T_{oven}: 40 °C, flow: 1 mL/min; 99:1 hexane/*i*PrOH, λ = 209.5 nm, minor enantiomer t_R = 2.17 min, major enantiomer t_R = 2.49 min].

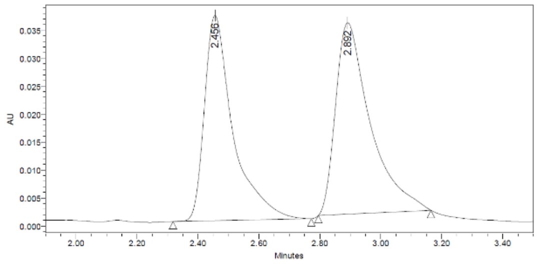

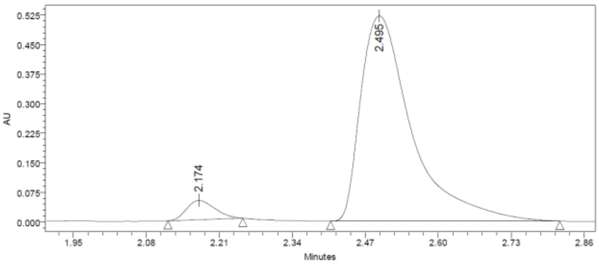

Unknown Peak Results

	RT	Area	% Area	Height
1	2.456	239221	46.88	36675
2	2.892	271060	53.12	34218

Unknown Peak Results

	RT	Area	% Area	Height
1	2.174	172674	4.97	49219
2	2.495	3298756	95.03	521041

(*S*)-4,4,5,5-Tetramethyl-2-(3-phenethyl-5-phenylhexa-1,5-dien-2-yl)-1,3,2-dioxaborolane ((-)-22).

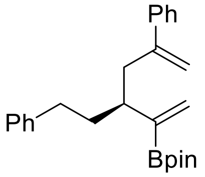

Synthesized from **1**, **52** and B_2pin_2 according to general procedure B. Yellow oil obtained in 37% yield with 71:29 er after column chromatography (Hexane/ EtOAc 4:1). **^{1}H NMR** (500 MHz, $CDCl_3$) δ 7.31 – 7.26 (m, 2H), 7.24 – 7.14 (m, 6H), 7.04 – 7.00 (m, 2H), 5.74 (d, *J* = 3.5 Hz, 1H), 5.32 (d, *J* = 3.5 Hz, 1H), 5.14 (d, *J* = 1.9 Hz, 1H), 4.91 (d, *J* = 1.7 Hz, 1H), 2.68 (dd, *J* = 14.1, 8.1 Hz, 1H), 2.61 – 2.53 (m, 1H), 2.52 – 2.38 (m, 1H), 2.33 – 2.25 (m, 1H), 2.25 – 2.12 (m, 1H), 1.83 – 1.75 (m, 1H), 1.74 – 1.67 (m, 1H), 1.21 (s, 6H), 1.20 (s, 6H). **^{13}C NMR** (126 MHz, $CDCl_3$) δ 146.5, 142.1, 140.4, 129.3, 127.3, 127.2, 127.1, 126.1, 125.5, 124.4, 112.8, 82.1, 43.9, 39.9, 34.6, 33.1, 23.8, 23.7. **^{11}B NMR** (160 MHz, $CDCl_3$) δ 30.7. **HRMS (APCI)** Calc. for $C_{26}H_{34}BO_2$ [$M+H^+$]: 389.2646, found 389.2655. **Specific rotation:** $[\alpha]_D^{20}$ -5.6 (*c*=0.72, $CHCl_3$).

Enantiomeric purity was determined by chiral SFC analysis [Lux Cellulose-1, 100 bar, T_{oven}: 40 °C, flow: 1.5 mL/min; 1% MeOH, λ = 220 nm, minor enantiomer t_R = 43.67 min, major enantiomer t_R = 45.63 min].

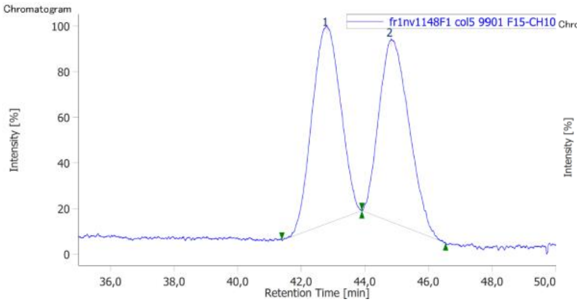

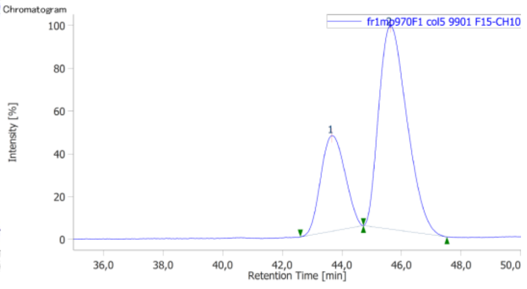

Peak Information

#	Peak Name	CH	tR [min]	Area [µV·sec]	Area%
1	Unknown	10	42.807	726335	50.609
2	Unknown	10	44.827	708850	49.391

Peak Information

#	Peak Name	CH	tR [min]	Area [µV·sec]	Area%
1	Unknown	10	43.667	2350605	28.751
2	Unknown	10	45.627	5825060	71.249

(*S*)-4,4,5,5-Tetramethyl-2-(3-phenethylhexa-1,5-dien-2-yl)-1,3,2-dioxaborolane ((-)-23).

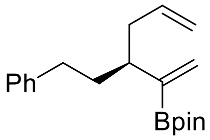

Synthesized from **1**, **65** and B_2pin_2 according to general procedure B at 60 °C over 5 h. Yellow oil obtained in 55% yield with 72:28 er after column chromatography (Hexane/ EtOAc 4:1). **^{1}H NMR** (300 MHz, $CDCl_3$) δ 7.30 – 7.22 (m, 2H), 7.20 – 7.13 (m, 3H), 5.90 (d, *J* = 3.4 Hz, 1H), 5.72 (ddt, *J* = 16.8, 10.2, 6.7 Hz, 1H), 5.60 (d, *J* = 3.4 Hz, 1H), 5.02 – 4.91 (m, 2H), 2.67 – 2.39 (m, 2H), 2.33 – 2.20 (m, 3H), 1.92 – 1.71 (m, 2H), 1.28 (s, 12H). **^{13}C NMR** (75 MHz, $CDCl_3$) δ 143.5, 138.2, 130.2, 128.7, 128.5, 125.8, 115.6, 83.5, 46.1, 39.6, 36.0, 34.2, 25.1, 25.0. **^{11}B NMR** (160 MHz, $CDCl_3$) δ 31.5. **HRMS (APCI)** Calc. for $C_{20}H_{30}BO_2$ [$M+H^+$]: 313.2333, found 313.2334. **Specific rotation:** $[\alpha]_D^{23}$ -2.6 (*c*=0.76, $CHCl_3$).

Enantiomeric purity was determined on the derived ketone **36** (see Section 7.3) by chiral SFC analysis [Lux Amylose-1, 100 bar, T_{oven}: 40 °C, flow: 1 mL/min; 5% MeOH, λ = 220 nm, minor enantiomer t_R = 9.45 min, major enantiomer t_R = 9.99 min].

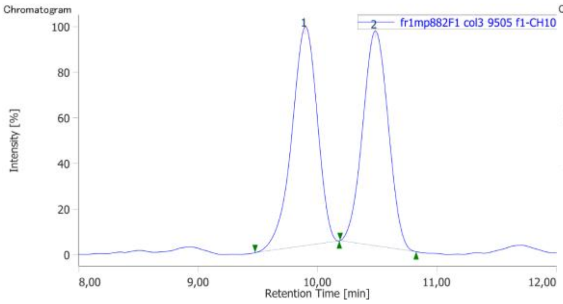

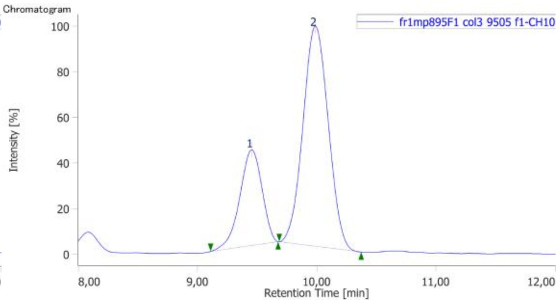

Peak Information

#	Peak Name	CH	tR [min]	Area [μV·sec]	Area%
1	Unknown	10	9,900	2918852	50,456
2	Unknown	10	10,487	2866090	49,544

Peak Information

#	Peak Name	CH	tR [min]	Area [μV·sec]	Area%
1	Unknown	10	9,453	1516653	27,593
2	Unknown	10	9,987	3979796	72,407

(*S*)-4,4,5,5-Tetramethyl-2-(3-phenylhexa-1,5-dien-2-yl)-1,3,2-dioxaborolane ((-)-25).

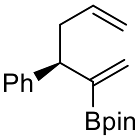

Synthesized from **51**, **65** and B_2pin_2 according to general procedure B at 60 °C over 5 h. Yellow oil obtained in 54% yield with 70:30 er after column chromatography (Hexane/ CH_2Cl_2 90:10 to 80:20). **^{1}H NMR** (300 MHz, $CDCl_3$) δ 7.34 – 7.21 (m, 4H), 7.21 – 7.13 (m, 1H), 5.87 (d, *J* = 2.9 Hz, 1H), 5.82 – 5.69 (m, 1H), 5.65 (d, *J* = 2.9 Hz, 1H), 5.07 – 4.91 (m, 2H), 3.62 (t, *J* = 7.7 Hz, 1H), 2.74 (dt, *J* = 14.2, 7.2 Hz, 1H), 2.57 (dt, *J* = 14.1, 7.4 Hz, 1H), 1.20 (s, 6H), 1.16 (s, 6H). **^{13}C NMR** (75 MHz, $CDCl_3$) δ 143.9, 137.6, 128.4, 128.0, 127.9, 125.9, 115.6, 83.3, 50.4, 38.3, 24.7, 24.5. **^{11}B NMR** (160 MHz, $CDCl_3$) δ 30.1. **HRMS (APCI)** Calc. for $C_{18}H_{26}BO_2$ [M+H$^+$]: 285.2020, found 285.2015. **Specific rotation:** $[\alpha]_D^{21}$ -9.8 (*c*=0.72, $CHCl_3$).

Enantiomeric purity was determined on the derived ketone **37** (see Section 7.3) by chiral uHPLC analysis [Lux i-Cellulose-5, T_{oven}: 40 °C, flow: 1 mL/min; 98:2 hexane/*i*PrOH, λ = 209.5 nm, minor enantiomer t_R = 4.83 min, major enantiomer t_R = 4.97 min].

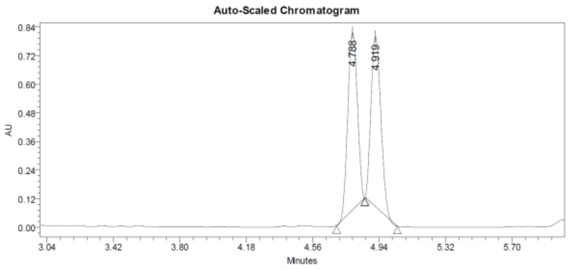

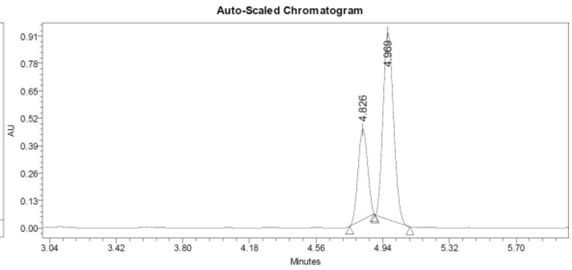

Unknown Peak Results

	RT	Area	% Area	Height
1	4.788	2813477	50.14	743033
2	4.919	2797770	49.86	713747

Unknown Peak Results

	RT	Area	% Area	Height
1	4.826	1616490	30.33	427236
2	4.969	3712911	69.67	884628

7. Derivatization of products

7.1 Cope rearrangement

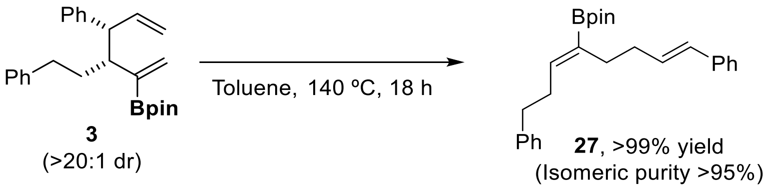

A solution of borylated 1,5-diene **3** (0.1 mmol, 1 equiv) in 1 ml of dry toluene was added to the reaction tube equipped with a magnetic stirring bar. The tube was sealed and the reaction was stirred at 140 °C for 18 h. Then, the mixture was diluted with Et_2O (5 ml) and washed with saturated aqueous solution of NH_4Cl (2x5 ml). The aqueous layer was extracted with Et_2O (5 ml). Combined organic layers were dried over anhydrous Na_2SO_4, filtered and solvent was removed under reduced pressure. Crude product was purified through flash column chromatography (Hexane/ CH_2Cl_2 90:10 to 80:20) affording the desired product as a colorless oil in >99% yield and with >95% of isomeric purity.

2-((3*Z*,7*E*)-1,8-Diphenylocta-3,7-dien-4-yl)-4,4,5,5-tetramethyl-1,3,2-dioxaborolane (27).

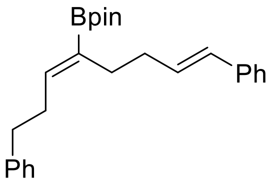

^{1}H NMR (500 MHz, $CDCl_3$) δ 7.27 – 7.22 (m, 2H), 7.22 – 7.16 (m, 4H), 7.13 – 7.05 (m, 4H), 6.35 (t, *J* = 7.0 Hz, 1H), 6.26 (d, *J* = 15.9 Hz, 1H), 6.14 (dt, *J* = 15.7, 6.7 Hz, 1H), 2.66 – 2.59 (m, 2H), 2.39 (dt, *J* = 10.5, 7.1 Hz, 2H), 2.22 (t, *J* = 7.3 Hz, 2H), 2.15 (t, *J* = 7.4 Hz, 2H), 1.17 (s, 12H). **^{13}C NMR** (126 MHz, $CDCl_3$) δ 145.4, 142.1, 138.0, 131.0, 129.8, 128.4, 128.4, 128.3, 126.7, 126.0, 125.8, 83.1, 35.5, 33.5, 30.8, 28.5, 24.8. **^{11}B NMR** (160 MHz, $CDCl_3$) δ 30.5. **HRMS (APCI)** Calc. for $C_{26}H_{34}BO_2$ [M+H$^+$]: 389.2646, found 389.2648.

7.2 Protodeboronation

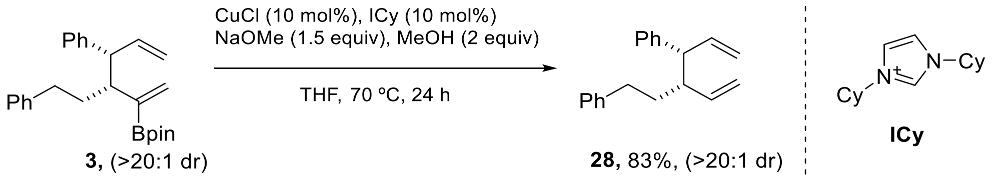

A flame-dried sealed tube equipped with a magnetic stirring bar was charged with CuCl (10 mol%, 0.02 mmol) and NaOMe (1.5 equiv, 0.3 mmol) in a glovebox. The sealed tube was removed from the glovebox, ligand **ICy** (10 mol%, 0.02 mmol) was added, and the mixture was dissolved in dry THF (0.4 ml) and stirred during 30 min at room temperature. The borylated 1,5-diene **3** (1.0 equiv, 0.2 mmol) was dissolved in dry THF (0.4 ml) and added to the sealed tube. Then, Methanol (2.0 equiv, 0.4 mmol, 16.2 uL) was added to the sealed tube. The resulting mixture was stirred over 24 h at 70 °C. Then, the mixture was diluted with CH_2Cl_2 (5 ml) and

washed with saturated aqueous solution of NH_4Cl (2x5 ml). The aqueous layer was extracted with CH_2Cl_2 (5 ml). Combined organic layers were dried over anhydrous Na_2SO_4, filtered and solvent was removed under reduced pressure. Crude product was purified through flash column chromatography (hexane) affording the desired product as a colorless oil in 83% yield and with >20:1 dr.

((3*S,4*R**)-3-Vinylhex-5-ene-1,4-diyl)dibenzene (28).**

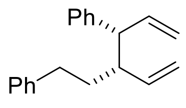

^{1}H NMR (300 MHz, $CDCl_3$) δ 7.35 – 7.25 (m, 4H), 7.24 – 7.11 (m, 6H), 6.03 (dt, *J* = 18.0, 9.2 Hz, 1H), 5.52 (dt, *J* = 17.7, 9.7 Hz, 1H), 5.13 – 5.05 (m, 2H), 5.01 (d, *J* = 10.4 Hz, 1H), 4.91 (d, *J* = 17.1 Hz, 1H), 3.26 (t, *J* = 8.5 Hz, 1H), 2.79 – 2.66 (m, 1H), 2.57 – 2.35 (m, 2H), 2.06 – 1.90 (m, 1H), 1.60 – 1.44 (m, 1H). **^{13}C NMR** (75 MHz, $CDCl_3$) δ 143.1, 142.8, 140.6, 140.4, 128.6, 128.5, 128.4, 128.3, 126.3, 125.8, 116.6, 115.7, 55.6, 48.6, 34.1, 33.6. **HRMS (APCI)** Calc. for $C_{20}H_{23}$ [M+H$^+$]: 262.1752, found 262.1748.

7.3 Oxidation (General Procedure C)

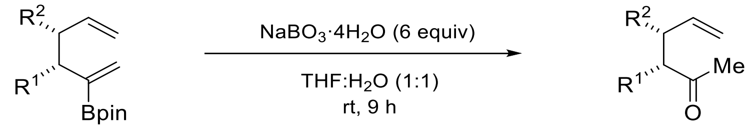

3, 11, 12, 13, 15, 17, 18, 23, 25, 26 **29-38**

A solution of the corresponding borylated diene (0.2 mmol, 1 equiv) in 1 ml of THF and 1 ml of water was stirred in the presence of $NaBO_3 \cdot 4H_2O$ (1.2 mmol, 6 equiv) over 9 hours. After this time, the reaction mixture was diluted in Et_2O and washed with brine. The organic layer was dried, filtered and the solvents were evaporated under vacuum. The final product was purified by SiO_2 column chromatography using Hexane/AcOEt (4:1) as eluent to afford the corresponding ketone (**29**-**38**).

(3*R,4*R**)-3-Phenethyl-4-phenylhex-5-en-2-one (29).**

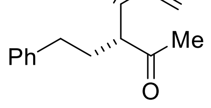

Synthesized from **3** according to general procedure C. Yellow oil obtained in 67% yield after column chromatography (Hexane/ EtOAc 4:1). **^{1}H NMR** (300 MHz, $CDCl_3$) δ 7.30 (td, *J* = 7.4, 2.4 Hz, 4H), 7.25 – 7.15 (m, 6H), 5.92 (dt, *J* = 16.9, 9.7 Hz, 1H), 5.17 – 5.08 (m, 2H), 3.47 (t, *J* = 9.8 Hz, 1H), 3.03 – 2.93 (m, 1H), 2.71 – 2.57 (m, 1H), 2.56 – 2.43 (m, 1H), 2.06 – 1.95 (m, 2H), 1.80 (s, 3H). **^{13}C NMR** (75 MHz, $CDCl_3$) δ 211.9, 142.1, 141.5, 139.0, 128.8, 128.5, 128.4, 127.7, 126.7, 126.0, 116.6, 57.1, 53.3, 33.6, 32.2, 31.2. **HRMS (APCI)** Calc. for $C_{20}H_{23}O$ [M+H$^+$]: 279.1743, found 279.1742.

***S,S*-29** was obtained from **(-)-3** with 98.5:1.5 er (see section 6.3) using the same procedure. **Specific rotation:** $[\alpha]_D^{21}$ -38.1 (*c*=1.0, $CHCl_3$).

(3*R,4*R**)-3-Phenethyl-4-(4-(trifluoromethyl)phenyl)hex-5-en-2-one (30)**

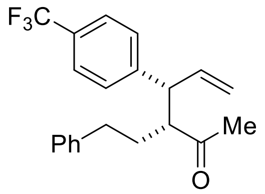

Synthesized from **11** according to general procedure C. Yellow oil obtained in 67% yield after column chromatography (Hexane/ EtOAc 4:1).**^{1}H NMR** (300 MHz, $CDCl_3$) δ 7.54 (d, *J* = 8.4 Hz, 2H), 7.34 – 7.20 (m, 5H), 7.19 – 7.10 (m, 2H), 5.87 (dt, *J* = 17.0, 9.5 Hz, 1H), 5.19 – 5.10 (m, 2H), 3.56 (t, *J* = 9.8 Hz, 1H), 3.05 – 2.95 (m, 1H), 2.72 – 2.56 (m, 1H), 2.54 – 2.42 (m, 1H), 1.99 (td, *J* = 7.6, 5.4 Hz, 2H), 1.83 (s, 3H). **^{13}C NMR** (75 MHz, $CDCl_3$) δ 211.6, 146.6, 141.6, 138.4, 129.3 (d, *J* = 31.8 Hz), 128.8, 128.7, 128.4, 126.5, 126.0 (q, *J* = 3.6 Hz), 124.4 (d, *J* = 271.4 Hz), 117.9, 57.0, 53.0, 33.6, 32.4, 31.5. **^{19}F NMR** (282 MHz, $CDCl_3$) δ -62.24. **HRMS (APCI)** Calc. for $C_{21}H_{22}F_3O$ [M+H$^+$]: 347.1617, found 347.1616.

***S,S*-30** was obtained from **(-)-11** with 96:4 er (see section 6.3) using the same procedure. **Specific rotation:** $[\alpha]_D^{22}$ -51.6 (*c*=0.50, $CHCl_3$).

(3*R,4*R**)-3-(2-((*tert*-Butyldimethylsilyl)oxy)ethyl)-4-(4-(trifluoromethyl)phenyl)hex-5-en-2-one (31).**

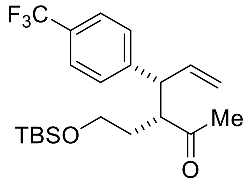

Synthesized from **12** according to general procedure C. Yellow oil obtained in 72% yield after column chromatography (Hexane/ EtOAc 4:1). **^{1}H NMR** (300 MHz, $CDCl_3$) δ 7.54 (d, *J* = 8.0 Hz, 2H), 7.30 (d, *J* = 8.1 Hz, 2H), 5.98 – 5.80 (m, 1H), 5.18 – 5.13 (m, 2H), 3.68 – 3.45 (m, 3H), 3.10 (td, *J* = 10.1, 3.8 Hz, 1H), 1.95 – 1.86 (m, 2H). 1.85 – 1.74 (s, 3H), 0.88 (s, 9H), 0.02 (s, 6H). **^{13}C NMR** (75 MHz, $CDCl_3$) δ 211.4, 146.4, 138.2, 128.3, 128.2, 125.6 (q, *J* = 3.6 Hz), 117.5, 61.1, 54.1, 52.8, 33.8, 31.6, 25.9, 18.3, -5.5, -5.5. *Quaternary C heterocoupled with F could not be seen. **^{19}F NMR** (282 MHz, $CDCl_3$) δ -62.28. **HRMS (APCI)** Calc. for $C_{21}H_{32}F_3O_2Si$ [M+H$^+$]: 401.2118, found 401.2133.

***S,S*-31** was obtained from **(-)-12** with 90:10 er (see section 6.3) using the same procedure. **Specific rotation:** $[\alpha]_D^{22}$ -41.3 (*c*=1.0, $CHCl_3$).

(3*R,4*R**)-4-(3-Chlorophenyl)-3-phenethylhex-5-en-2-one (32).**

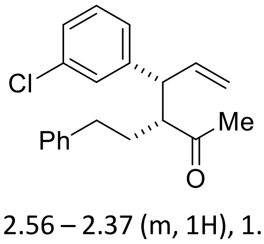

Synthesized from **13** according to general procedure C. Yellow oil obtained in 46% yield after column chromatography (Hexane/ EtOAc 4:1)**.^{1}H NMR** (300 MHz, $CDCl_3$) δ 7.29 (d, *J* = 8.3 Hz, 2H), 7.27 – 7.11 (m, 6H), 7.05 (d, *J* = 7.4 Hz, 1H), 5.87 (dt, *J* = 18.2, 9.4 Hz, 1H), 5.19 – 5.03 (m, 2H), 3.47 (t, *J* = 9.8 Hz, 1H), 2.96 (q, *J* = 7.8 Hz, 1H), 2.72 – 2.56 (m, 1H), 2.56 – 2.37 (m, 1H), 1.98 (q, *J* = 7.8 Hz, 2H), 1.86 (s, 3H). **^{13}C NMR** (75 MHz, $CDCl_3$) δ 211.4, 144.4, 141.5, 138.4, 134.7, 130.1, 128.6, 128.5, 128.0, 127.1, 126.3, 126.1, 117.4, 56.9, 52.8, 33.5, 32.2, 31.4. **HRMS (APCI)** Calc. for $C_{20}H_{22}ClO$ [M+H+]: 313.1354, found 313.1355.

***S,S*-32** was obtained from **(-)-13** with 96:4 er (see section 6.3) using the same procedure. **Specific rotation:** $[\alpha]_D^{20}$ -16.1 (*c*=0.93, $CHCl_3$).

(3*R,4*R**)-4-(4-Methoxyphenyl)-3-phenethylhex-5-en-2-one (33).**

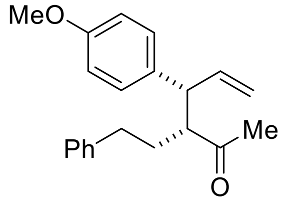

Synthesized from **15** according to general procedure C. Yellow oil obtained in 47% yield after column chromatography (Hexane/ EtOAc 4:1). **^{1}H NMR** (500 MHz, $CDCl_3$) δ 7.26 – 7.16 (m, 2H), 7.15 – 7.09 (m, 1H), 7.09 – 7.04 (m, 2H), 6.99 (d, *J* = 8.6 Hz, 2H), 6.74 (d, *J* = 8.6 Hz, 2H), 5.79 (ddd, *J* = 16.8, 10.2, 9.2 Hz, 1H), 5.03 – 4.96 (m, 2H), 3.69 (s, 3H), 3.32 (t, *J* = 9.8 Hz, 1H), 2.83 (td, *J* = 9.9, 4.2 Hz, 1H), 2.53 (ddd, *J* = 13.8, 9.8, 5.9 Hz, 1H), 2.39 (ddd, *J* = 13.7, 9.6, 7.0 Hz, 1H), 1.96 – 1.82 (m, 2H), 1.71 (s, 3H). **^{13}C NMR** (126 MHz, $CDCl_3$) δ 212.3, 158.4, 141.7, 139.4, 134.2, 128.8, 128.6, 128.5, 126.1, 116.4, 114.3, 57.3, 55.3, 52.6, 33.7, 32.3, 31.3. **HRMS (APCI)** Calc. for $C_{21}H_{25}O_2$ [M+H$^+$]: 309.1849, found 309.1852.

***S,S*-33** was obtained from **(-)-15** with 93.5:6.5 er (see section 6.3) using the same procedure. **Specific rotation:** $[\alpha]_D^{20}$ -24.3 (*c*=0.66, $CHCl_3$).

(3*R,4*R**)-3-Phenethyl-4-(((tetrahydro-2H-pyran-2-yl)oxy)methyl)hex-5-en-2-one (34)**

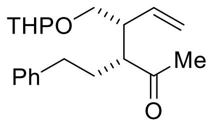

Synthesized from **17** according to general procedure C. Yellow oil obtained in 67% yield after column chromatography (Hexane/ EtOAc 4:1). **^{1}H NMR** (300 MHz, $CDCl_3$) δ 7.30 – 7.22 (m, 2H), 7.21 – 7.10 (m, 3H), 5.87 – 5.67 (m, 1H), 5.26 – 4.49 (m, 2H), 4.52 (bs, 1H), 3.91 – 3.76 (m, 2H), 3.72 (dd, *J* = 10.1, 6.5 Hz, 1H), 3.57 – 3.40 (m, 2H), 3.33 (dd, *J* = 9.8, 6.9 Hz, 1H), 3.09 – 2.96 (m, 1H), 2.95 – 2.83 (m, 1H), 2.79 – 2.60 (m, 2H), 1.88 (s, 3H), 1.84 (s, 3H)*, 1.74 – 1.47 (m, 6H). **^{13}C NMR** (75 MHz, $CDCl_3$) δ 212.0, 211.6*, 139.8, 139.8*, 137.0, 136.8*, 128.9, 128.9*, 128.5, 128.4*, 126.2, 118.0, 117.9*, 99.2, 98.7*, 69.2, 68.9*, 62.3, 62.3*, 55.7, 55.5*, 46.6, 46.6*, 36.7, 36.1*, 32.2, 31.6, 30.4, 30.4*, 25.4, 19.5, 19.5*. **HRMS (APCI)** Calc. for $C_{20}H_{29}O_3$ [M+H$^+$]: 317.1497, found 317.1463.

**Corresponds to the other THP diastereomer.*

(3*R,4*R**)-4-((Benzyloxy)methyl)-3-phenethylhex-5-en-2-one (35)**

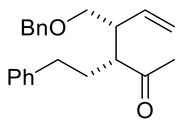

Synthesized from **18** according to general procedure C. Yellow oil obtained in 77% yield after column chromatography (Hexane/ EtOAc 4:1). **^{1}H NMR** (300 MHz, $CDCl_3$) δ 7.40 – 7.25 (m, 7H), 7.23 – 7.12 (m, 3H), 5.63 (ddd, *J* = 16.4, 10.4, 8.4 Hz, 1H), 5.16 – 5.06 (m, 2H), 4.44 (s, 2H), 3.56 – 3.38 (m, 2H), 2.79 – 2.70 (m, 2H), 2.70 – 2.50 (m, 1H), 2.53 – 2.37 (m, 1H), 2.14 (s, 3H), 1.98 – 1.71 (m, 2H). **^{13}C NMR** (75 MHz, $CDCl_3$) δ 211.6, 141.8, 138.1, 136.7, 128.4, 128.4, 127.7, 127.6, 126.0, 117.8, 73.2, 72.2, 53.3, 46.3, 33.5, 30.9, 30.3. **HRMS (APCI)** Calc. for $C_{22}H_{27}O_2$ [M+H$^+$]: 323.2006, found 323.2005.

***S,S*-36** was obtained from **(-)-18** with 95:5 er (see section 6.3) using the same procedure. **Specific rotation:** $[\alpha]_D^{20}$ -14.4 (*c*=0.57, $CHCl_3$).

3-Phenethylhex-5-en-2-one (36)

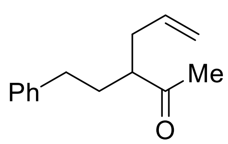

Synthesized from **23** according to general procedure C. Yellow oil obtained in 92% yield after column chromatography (Hexane/ EtOAc 4:1). **^{1}H NMR** (500 MHz, $CDCl_3$) δ 7.24 – 7.16 (m, 2H), 7.15 – 7.04 (m, 3H), 5.63 (ddt, *J* = 17.1, 10.1, 7.0 Hz, 1H), 5.02 – 4.90 (m, 2H), 2.58 – 2.42 (m, 3H), 2.34 – 2.24 (m, 1H), 2.22 – 2.11 (m, 1H), 2.05 (s, 3H), 1.89 (dddd, *J* = 14.0, 9.6, 8.3, 6.0 Hz, 1H), 1.67 (dddd, *J* = 13.8, 9.8, 6.5, 5.4 Hz, 1H). **^{13}C NMR** (126 MHz, $CDCl_3$) δ 211.7, 141.7, 135.4, 128.6, 128.5, 126.2, 117.1, 52.0, 35.9, 33.5, 32.7, 29.4. **HRMS (APCI)** Calc. for $C_{14}H_{19}O$ [M+H$^+$]: 203.1430, found 203.1429.

***S*-37** was obtained from **(-)-23** with 72:28 er (see section 6.3) using the same procedure. **Specific rotation:** $[\alpha]_D^{20}$ +0.91 (*c*=0.9, $CHCl_3$).

3-Phenylhex-5-en-2-one (37)

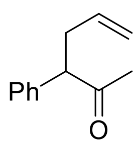

Synthesized from **25** according to general procedure C. Yellow oil obtained in 73% yield after column chromatography (Hexane/ EtOAc 4:1). **^{1}H NMR** (500 MHz, $CDCl_3$) δ 7.30 – 7.23 (m, 2H), 7.22 – 7.17 (m, 1H), 7.16 – 7.11 (m, 2H), 5.60 (ddt, *J* = 17.1, 10.2, 6.9 Hz, 1H), 5.00 – 4.81 (m, 2H), 3.66 – 3.59 (m, 1H), 2.73 (dtt, *J* = 14.3, 7.2, 1.3 Hz, 1H), 2.36 (dddt, *J* = 14.4, 8.0, 6.8, 1.4 Hz, 1H), 1.99 (s, 3H). **^{13}C NMR** (126 MHz, $CDCl_3$) δ 207.8, 138.5, 135.9, 129.1, 128.4, 127.5, 116.8, 59.6, 36.3, 29.3. **HRMS (APCI)** Calc. for $C_{12}H_{15}O$ [M+H$^+$]: 175.1117, found 175.1118.

***S*-38** was obtained from **(-)-25** with 70:30 er (see section 6.3) using the same procedure. **Specific rotation:** $[\alpha]_D^{20}$ -28.0 (*c*=1.5, $CHCl_3$).

(3*S*,4*S*)-4-(4-Bromophenyl)-3-phenethylhex-5-en-2-one (38).

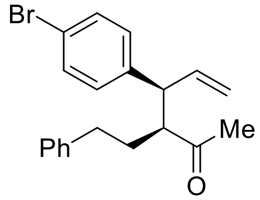

Synthesized from **(-)-26** according to general procedure C. Yellow oil obtained in 74% yield with 98.5:1.5 er (see section 6.3) after column chromatography (Hexane/ EtOAc 4:1). **^{1}H NMR** (500 MHz, $CDCl_3$) δ 7.40 (d, *J* = 8.4 Hz, 2H), 7.31 – 7.27 (m, 2H), 7.22 – 7.11 (m, 3H), 7.03 (d, *J* = 8.4 Hz, 2H), 5.84 (dt, *J* = 17.3, 9.4 Hz, 1H), 5.14 – 5.06 (m, 2H), 3.44 (t, *J* = 9.8 Hz, 1H), 2.92 (ddd, *J* = 10.4, 9.0, 4.5 Hz, 1H), 2.61 (ddd, *J* = 13.6, 9.2, 6.5 Hz, 1H), 2.47 (ddd, *J* = 13.7, 9.3, 7.2 Hz, 1H), 2.01 – 1.91 (m, 2H), 1.82 (s, 3H). **^{13}C NMR** (126 MHz, $CDCl_3$) δ 211.6, 141.5, 141.3, 138.5, 132.0, 129.6, 128.6, 128.5, 126.3, 120.7, 117.3, 57.0, 52.6, 33.5, 32.2, 31.4. **HRMS (APCI)** Calc. for $C_{20}H_{22}BrO$ [M+H$^+$]: 357.0849, found 357.0849. **Specific rotation:** $[\alpha]_D^{20}$ -32.1 (*c*=0.89, $CHCl_3$)

7.4 Alcohol + Boronic ester deprotection

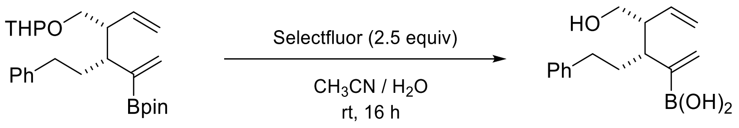

17, (8:1 dr) **39,** 70% (7:1 dr)

A solution of borylated diene **17** (0.1 mmol) and Selectfluor (0.25 mmol) was diluted in 2 ml of CH_3CN and 0.1 ml of water. The mixture was stirring at room temperature for 16 hours. The final product was purified by SiO_2 column chromatography using Hexane / AcOEt (4:1) as eluent to afford the product **39** in 70% yield.

((3*R,4*R**)-4-(Hydroxymethyl)-3-phenethylhexa-1,5-dien-2-yl)boronic acid (39).**

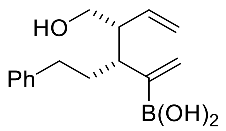

^{1}H NMR (500 MHz, $CDCl_3$) δ 7.23 – 7.19 (m, 2H), 7.13 – 7.08 (m, 3H), 5.92 (dd, *J* = 3.0, 1.4 Hz, 1H), 5.70 – 5.58 (m, 2H), 5.11 – 5.02 (m, 2H), 4.13 (dd, *J* = 11.5, 3.4 Hz, 1H), 3.89 (dd, *J* = 11.5, 4.5 Hz, 1H), 2.64 – 2.56 (m, 1H), 2.56 – 2.46 (m, 1H), 2.36 – 2.27 (m, 2H), 1.85 – 1.76 (m, 1H), 1.72 – 1.63 (m, 1H). **^{13}C NMR** (126 MHz, $CDCl_3$) δ 142.4, 138.4, 128.8, 128.4, 125.8, 116.4, 66.1, 45.1, 44.8, 34.9, 32.2. **^{11}B NMR** (160 MHz, $CDCl_3$) δ 27.26. **HRMS (APCI)** Calc. for $C_{15}H_{22}BO_3$ [M+H$^+$]: 261.1624, found 261.1620.

7.5 Methylene cyclobutane formation (General procedure D)

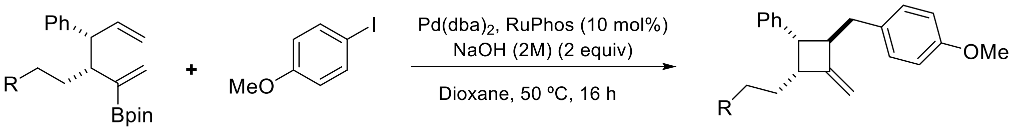

3 (R= Ph)
6 (R= OTBS)

S8
(1.5 equiv)

40, R= Ph; 58% (>20:1 dr)
41, R= OTBS; 52% (>20:1dr)

In a Schlenk flask, $Pd(dba)_2$ (10 mol%, 0.02 mmol) and RuPhos (10 mol%, 0.02 mmol) were added. Then, a solution of the corresponding 1,5-borylated diene (0.2 mmol) was added in 1,4-dioxane (2.0 ml), followed by the addition of the iodoarene **S8** (1.5 equiv, 0.3 mmol) and NaOH (2.0 M, 2.0 equiv, 0.4 mmol). The reaction was stirred at 50 °C for 16 h. After this time, the resulting crude was diluted in Et_2O (15 ml) and washed with H_2O (2 x 10 ml). The organic layer was dried over Na_2SO_4 and filtered. The residue was purified by column chromatography affording the desired product as a yellow oil.

1-Methoxy-4-(((1*R,3*R**,4*S**)-2-Methylene-3-phenethyl-4-phenylcyclobutyl)methyl)benzene (40).**

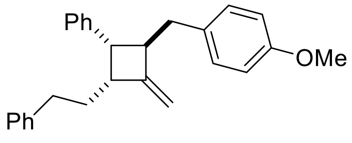

Synthesized from **3** and **S8** according to general procedure D. Yellow oil obtained in 58% yield after column chromatography (Hexane/ CH_2Cl_2 6:4).**^{1}H NMR** (500 MHz, $CDCl_3$) δ 7.20 – 7.16 (m, 2H), 7.13 – 7.06 (m, 5H), 7.04 – 7.00 (m, 3H), 6.84 (d, *J* = 8.2 Hz, 2H), 6.70 (d, J = 8.7 Hz, 2H), 4.80 (t, *J* = 2.2 Hz, 1H), 4.73 (t, *J* = 2.3 Hz, 1H), 3.69 (s, 3H), 3.44 – 3.34 (m, 1H), 3.30 (dd, *J* = 9.2, 7.0 Hz, 1H), 3.18 – 3.03 (m, 1H), 2.84 (dd, *J* = 7.2, 2.3 Hz, 2H), 2.36 (ddd, *J* = 13.2, 10.2, 5.7 Hz, 1H), 2.20 (ddd, *J* = 13.3, 10.2, 5.9 Hz, 1H), 1.50 – 1.43 (m, 1H), 1.40 – 1.32 (m, 1H). **^{13}C NMR** (126 MHz, $CDCl_3$) δ 158.2, 155.4, 142.7, 140.9, 132.6, 130.2, 128.6, 128.6, 128.5, 128.3, 126.3, 125.8, 113.7, 105.2, 55.5, 48.9, 46.2, 45.8, 39.5, 33.8, 32.2. **HRMS (APCI)** Calc. for $C_{27}H_{29}O$ [M+H$^+$]: 369.2213, found 369.2211.

***tert*-Butyl(2-((1*R**,3*R**,4*R**)-3-(4-methoxybenzyl)-2-methylene-4-phenylcyclobutyl)ethoxy) dimethylsilane (41).**

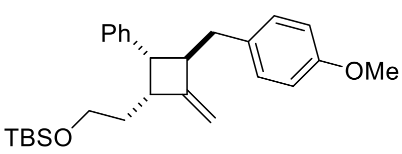

Synthesized from **6** and **S8** according to general procedure D. Yellow oil obtained in 52% yield after column chromatography (Hexane/ CH_2Cl_2 90:10 to 70:30).**^{1}H NMR** (500 MHz, $CDCl_3$) δ 7.34 – 7.29 (m, 2H), 7.25 – 7.13 (m, 5H), 6.84 (d, *J* = 8.5 Hz, 2H), 4.91 (t, *J* = 2.2 Hz, 1H), 4.84 (t, *J* = 2.3 Hz, 1H), 3.83 (s, 3H), 3.54 – 3.33 (m, 5H), 3.03 – 2.95 (m, 2H), 1.50 (dq, *J* = 13.8, 7.0 Hz, 1H), 1.43 (dq, *J* = 13.6, 6.8 Hz, 1H), 0.90 (s, 9H), 0.00 (s, 6H). **^{13}C NMR** (126 MHz, $CDCl_3$) δ 158.0, 155.2, 141.0, 132.5, 130.1, 128.4, 128.2, 126.2, 113.8, 105.0, 61.2, 55.4, 48.9, 45.5, 42.9, 39.4, 33.0, 26.1, 18.5, -5.2, -5.2. **HRMS (APCI)** Calc. for $C_{27}H_{39}O_2Si$ [M+H$^+$]: 423.2714, found 423.2715.

***S,S,S*-41** was obtained from **(-)-6** with 96.5:3.5 er (see section 6.3) using the same procedure. **Specific rotation:** $[\alpha]_D^{20}$ -29.6 (*c*=0.30, $CHCl_3$).

7.6 Zweifel-type coupling (General procedure E)

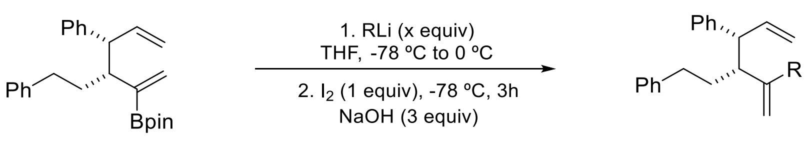

3

42, (Use of 1.5 equiv PhLi) R = Ph; 57%
43, (Use of 2.5 equiv MeLi) R = Me; 62%

In a Schlenk dry flask, the borylated diene **3** (0.2 mmol) was diluted in Et_2O (0.8 ml) and cooled to -78 °C. Then PhLi or MeLi was added. After 30 min, the reaction was warmed to 0 °C and stirred during 1h. After this time, solvents were evaporated under vacuum and a solution of I_2 (0.2 mmol) in dry MeOH (0.4 ml) was added at -78 °C. After 3 hours, a solution of aq. NaOH 3M (0.6 mmol) was added and diluted with Et_2O (15 ml). The organic layer was dried over Na_2SO_4

and filtered. The residue was purified by column chromatography (Hex/ EtOAc 4:1) affording the desired product as a yellow oil.

((3*R,4*R**)-3-Phenethylhexa-1,5-diene-2,4-diyl)dibenzene (42).**

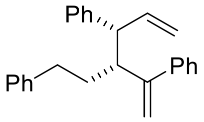

Synthesized from **3** and PhLi (1.5 equiv) according to general procedure E. Yellow oil obtained in 57% yield after column chromatography (Hexane/ EtOAc 4:1). **^{1}H NMR** (300 MHz, $CDCl_3$) δ 7.35 – 7.04 (m, 15H), 6.02 (dt, *J* = 16.6, 10.1 Hz, 1H), 5.32 (bs, 1H), 5.14 – 5.02 (m, 3H), 3.39 (t, *J* = 9.0 Hz, 1H), 3.06 (td, *J* = 9.5, 3.2 Hz, 1H), 2.84 (ddd, *J* = 15.0, 11.1, 4.6 Hz, 1H), 2.58 (ddd, *J* = 13.4, 10.4, 6.5 Hz, 1H), 2.24 – 2.04 (m, 1H), 1.98 – 1.77 (m, 1H). **^{13}C NMR** (75 MHz, $CDCl_3$) δ 150.2, 143.6, 143.4, 142.5, 139.9, 128.4, 128.3, 128.1, 128.0, 126.9, 126.8, 125.9, 125.7, 115.9, 114.7, 55.2, 48.9, 33.4, 33.4. **HRMS (APCI)** Calc. for $C_{26}H_{27}$ [$M+H^+$]: 339.2091, found 339.2094.

((3*R,4*R**)-3-(Prop-1-en-2-yl)hex-5-ene-1,4-diyl)dibenzene (43).**

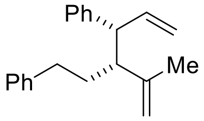

Synthesized from **3** and MeLi (2.5 equiv) according to general procedure E. Yellow oil obtained in 62% yield after column chromatography (Hexane/ EtOAc 4:1) **^{1}H NMR** (300 MHz, $CDCl_3$) δ 7.35 – 7.26 (m, 4H), 7.26 – 7.12 (m, 6H), 5.91 (ddd, *J* = 16.9, 10.0, 9.2 Hz, 1H), 5.10 – 5.00 (m, 2H), 4.72 (bs, 1H), 4.62 (bs, 1H), 3.23 (t, *J* = 9.9 Hz, 1H), 2.67 (ddd, *J* = 15.1, 10.6, 4.8 Hz, 1H), 2.54 – 2.42 (m, 2H), 2.06 (ddt, *J* = 17.2, 10.2, 3.3 Hz, 1H), 1.69 – 1.59 (m, 1H), 1.56 (s, 3H). **^{13}C NMR** (75 MHz, $CDCl_3$) δ 144.7, 143.6, 142.7, 141.4, 128.5, 128.3, 128.2, 127.9, 126.0, 125.6, 115.3, 114.4, 54.9, 51.4, 33.9, 32.6, 18.6. **HRMS (APCI)** Calc. for $C_{21}H_{25}$ [$M+H^+$]: 277.1951, found 277.1941.

8. NMR spectra

^{1}H NMR (300 MHz, $CDCl_3$)

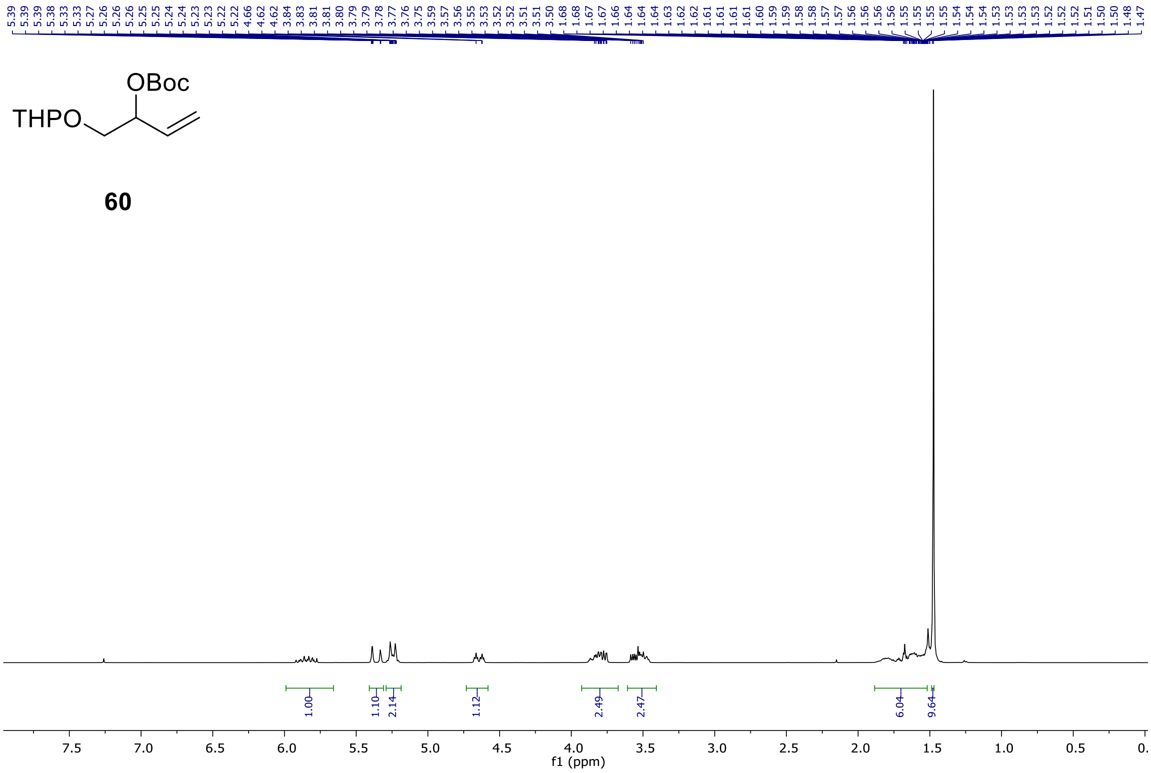

^{13}C NMR (75 MHz, $CDCl_3$)

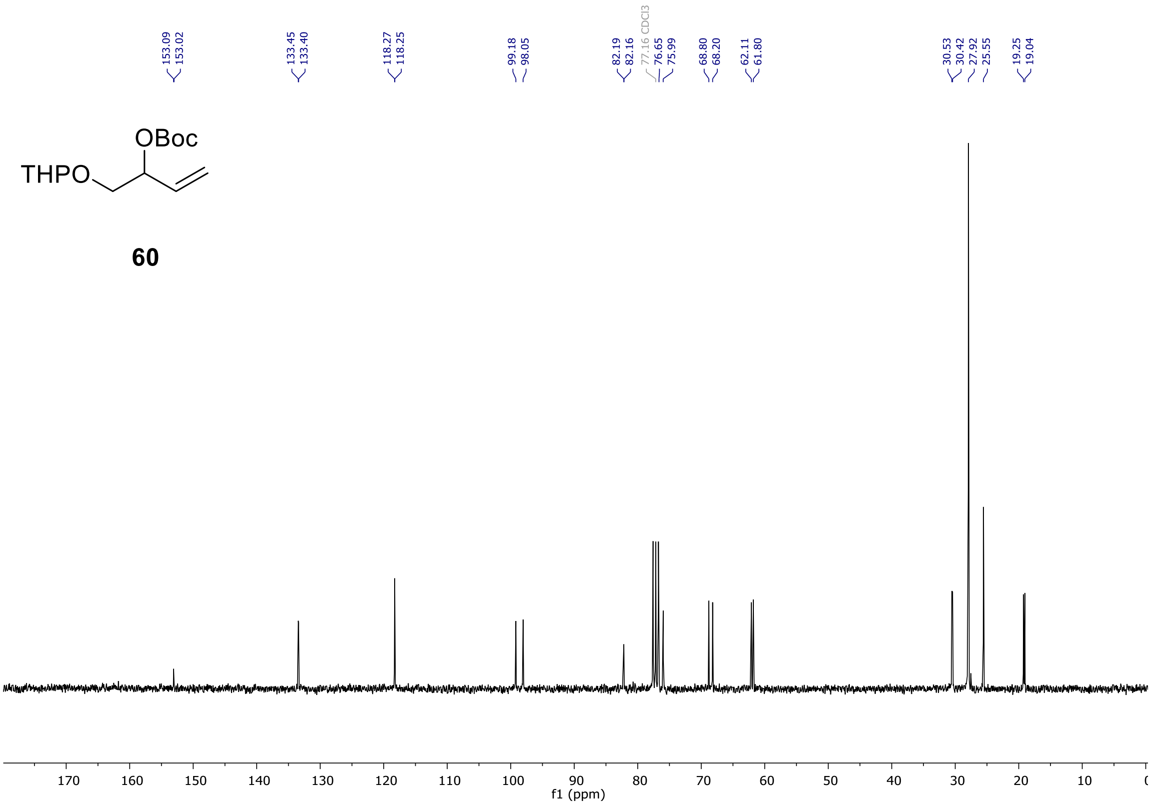

^{1}H NMR (300 MHz, $CDCl_3$)

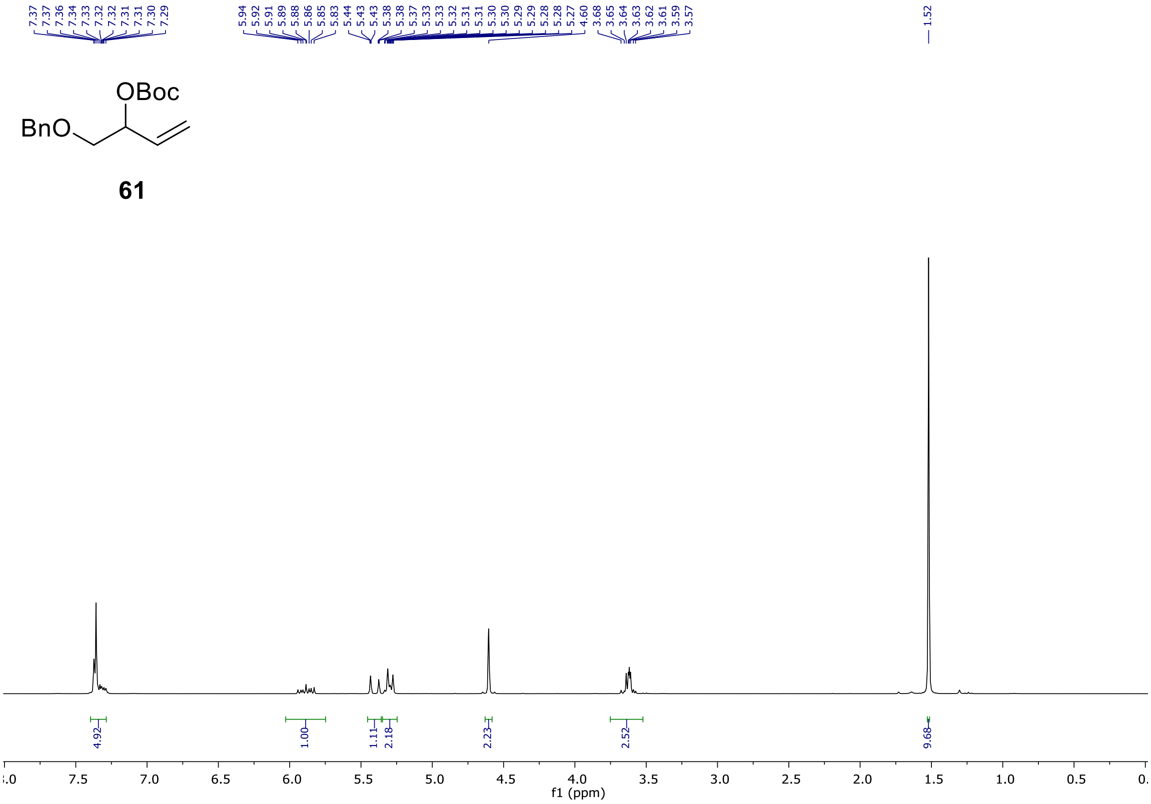

^{13}C NMR (75 MHz, $CDCl_3$)

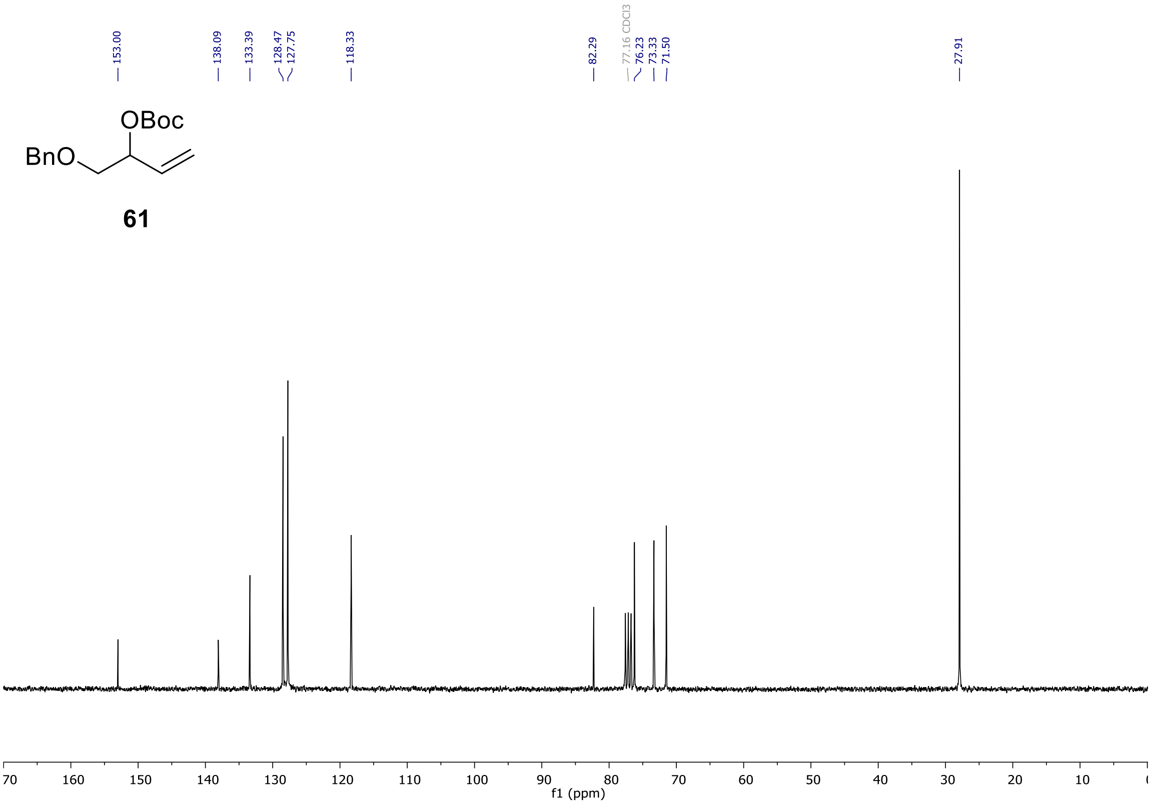

^{1}H NMR (300 MHz, $CDCl_3$)

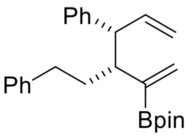

3

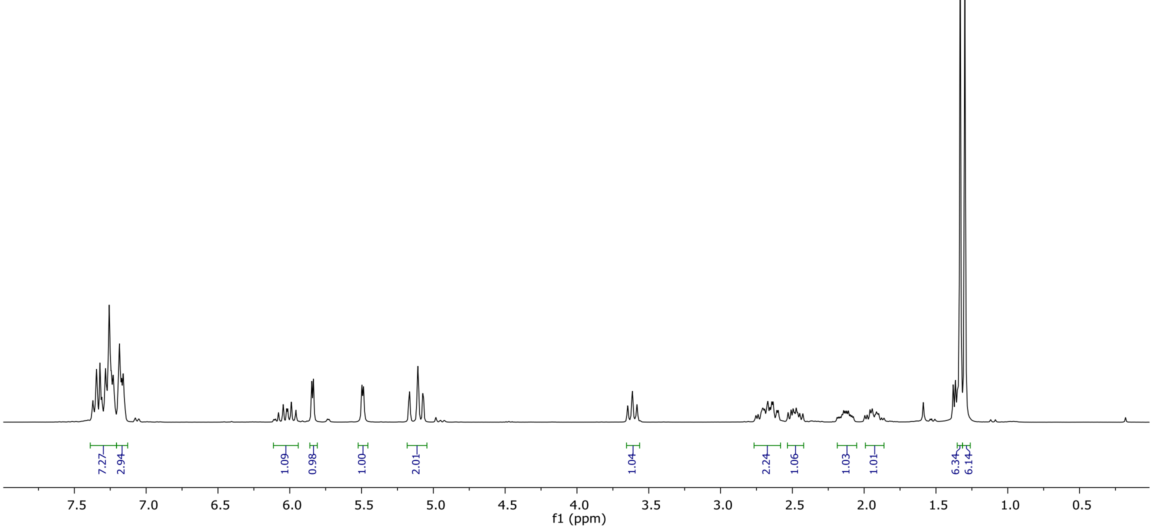

^{13}C NMR (75 MHz, $CDCl_3$)

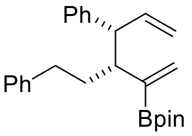

3

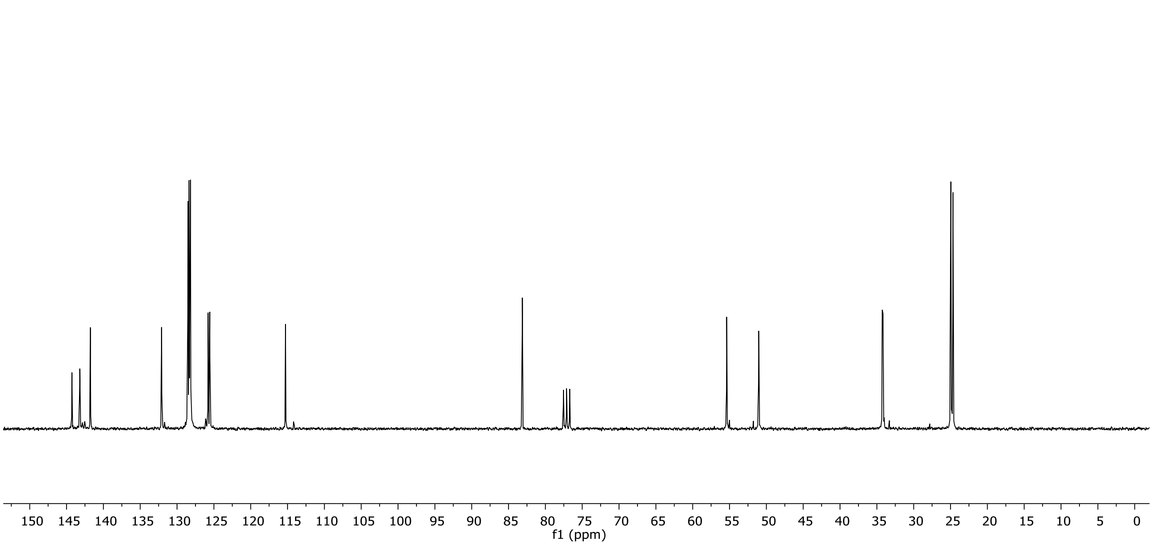

^{1}H NMR (300 MHz, $CDCl_3$)

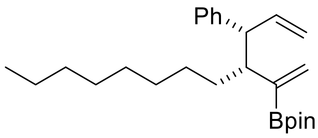

4

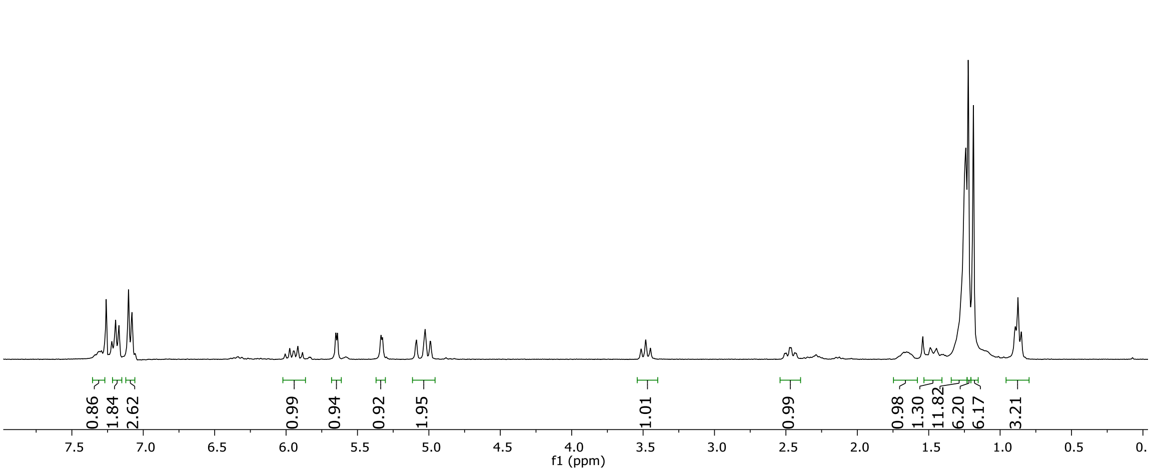

^{13}C NMR (75 MHz, $CDCl_3$)

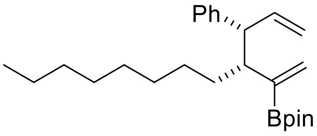

4

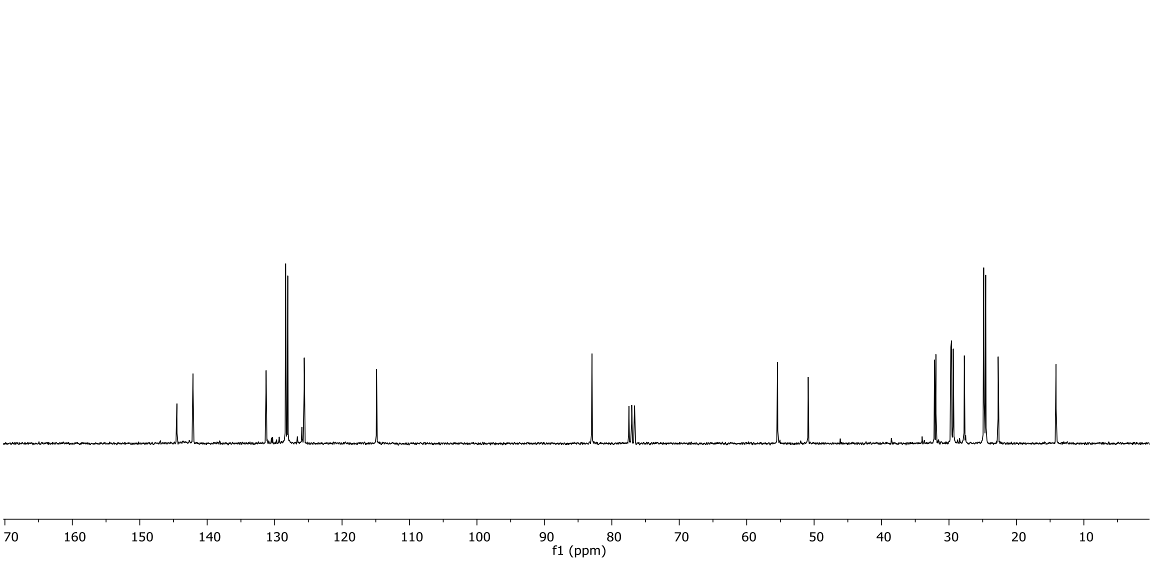

^{1}H NMR (300 MHz, $CDCl_3$)

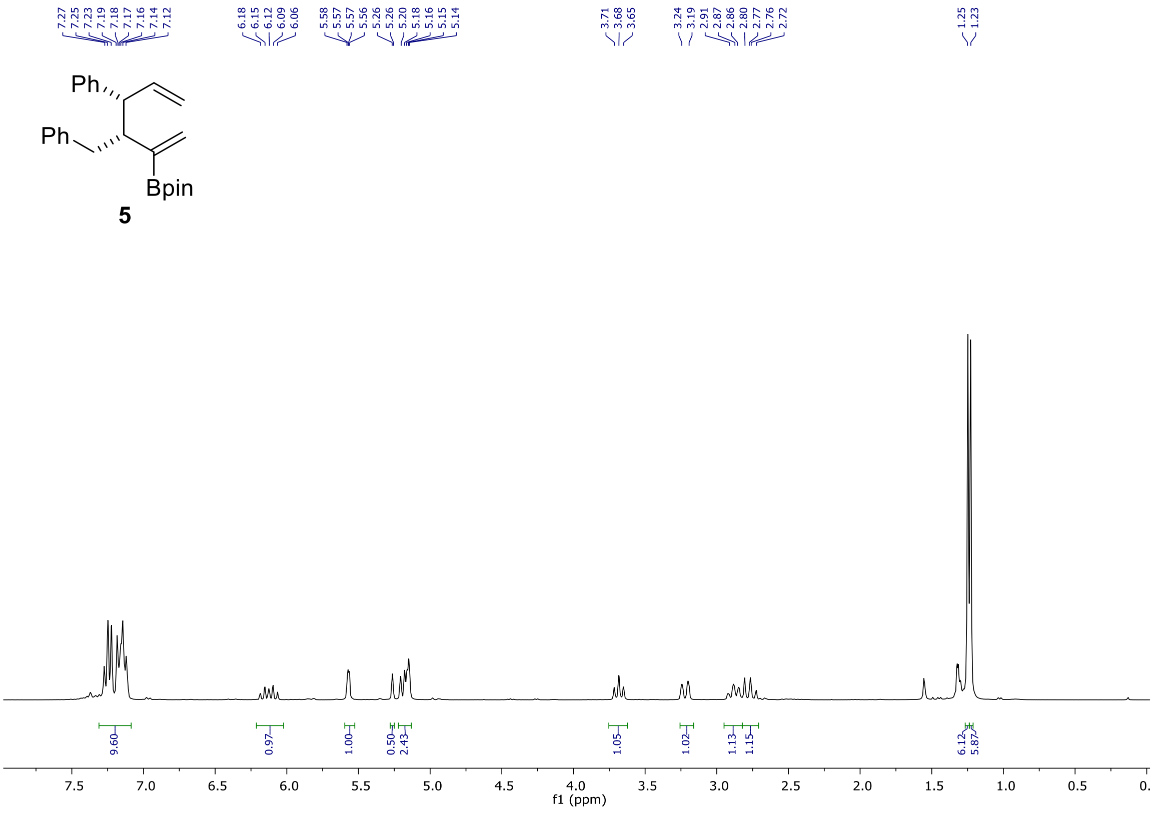

^{13}C NMR (75 MHz, $CDCl_3$)

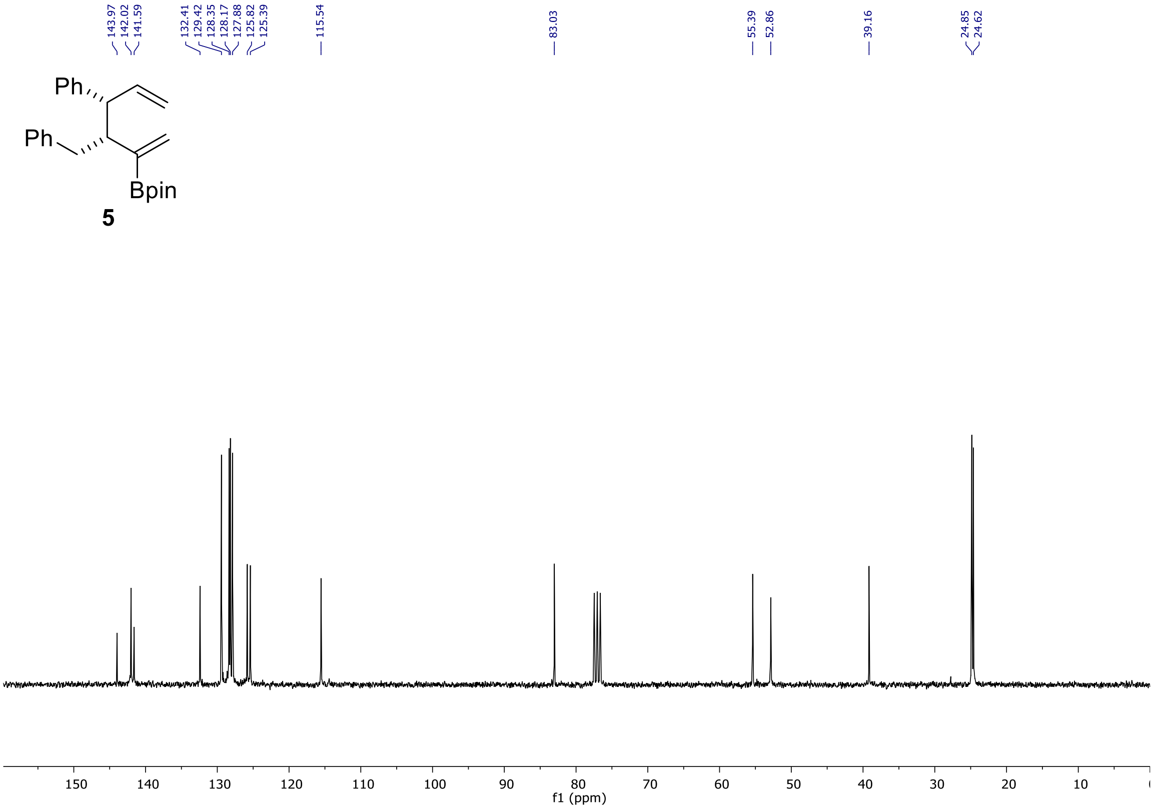

^{1}H NMR (300 MHz, $CDCl_3$)

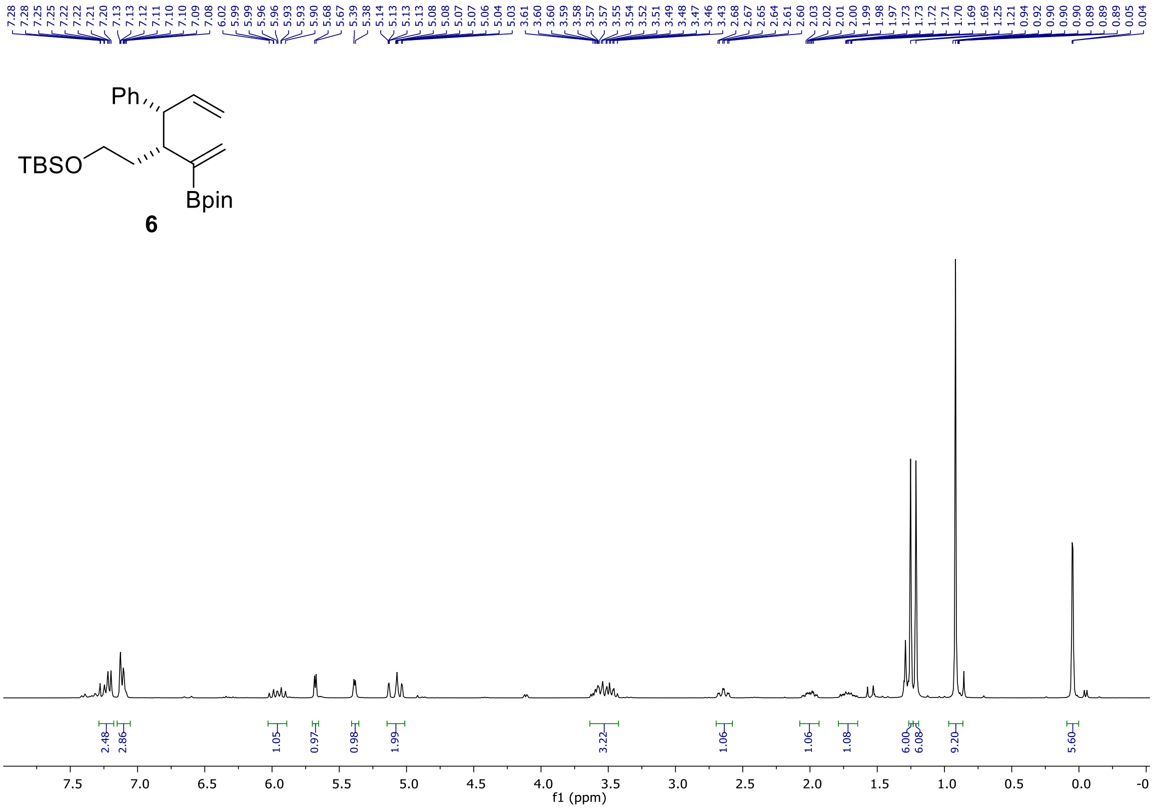

^{13}C NMR (75 MHz, $CDCl_3$)

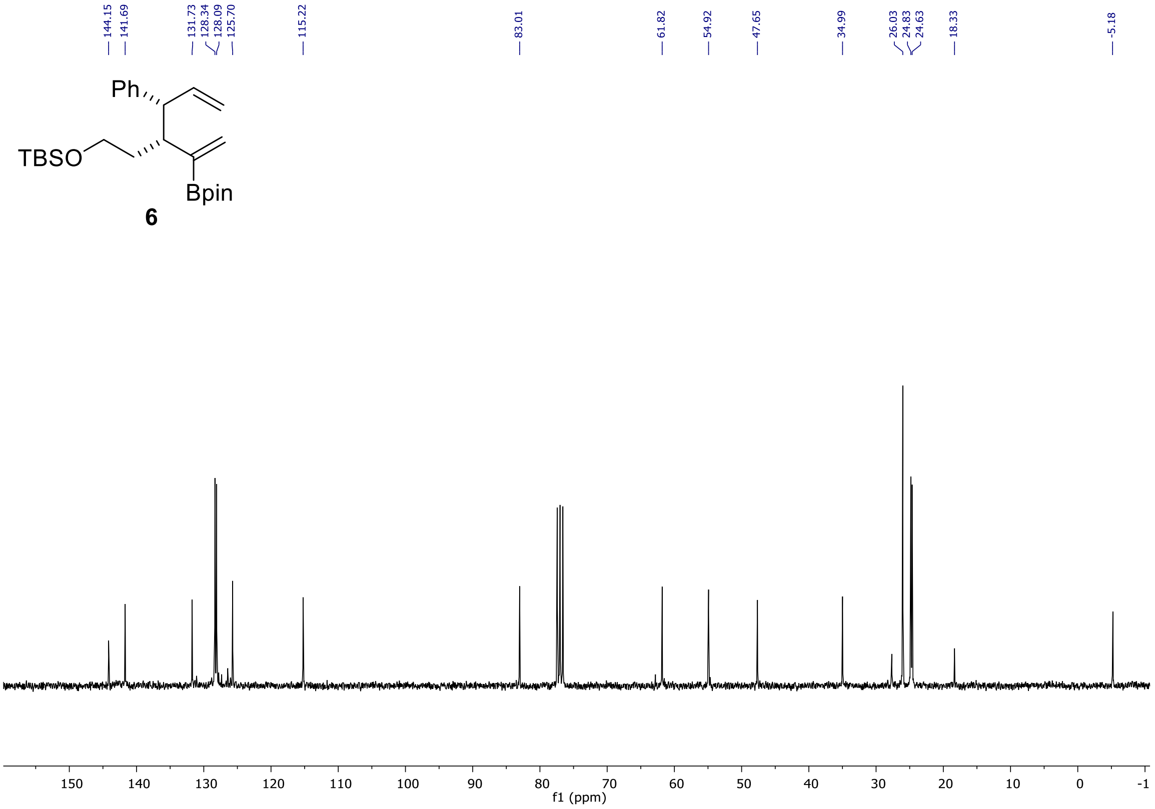

¹H NMR (300 MHz, $CDCl_3$)

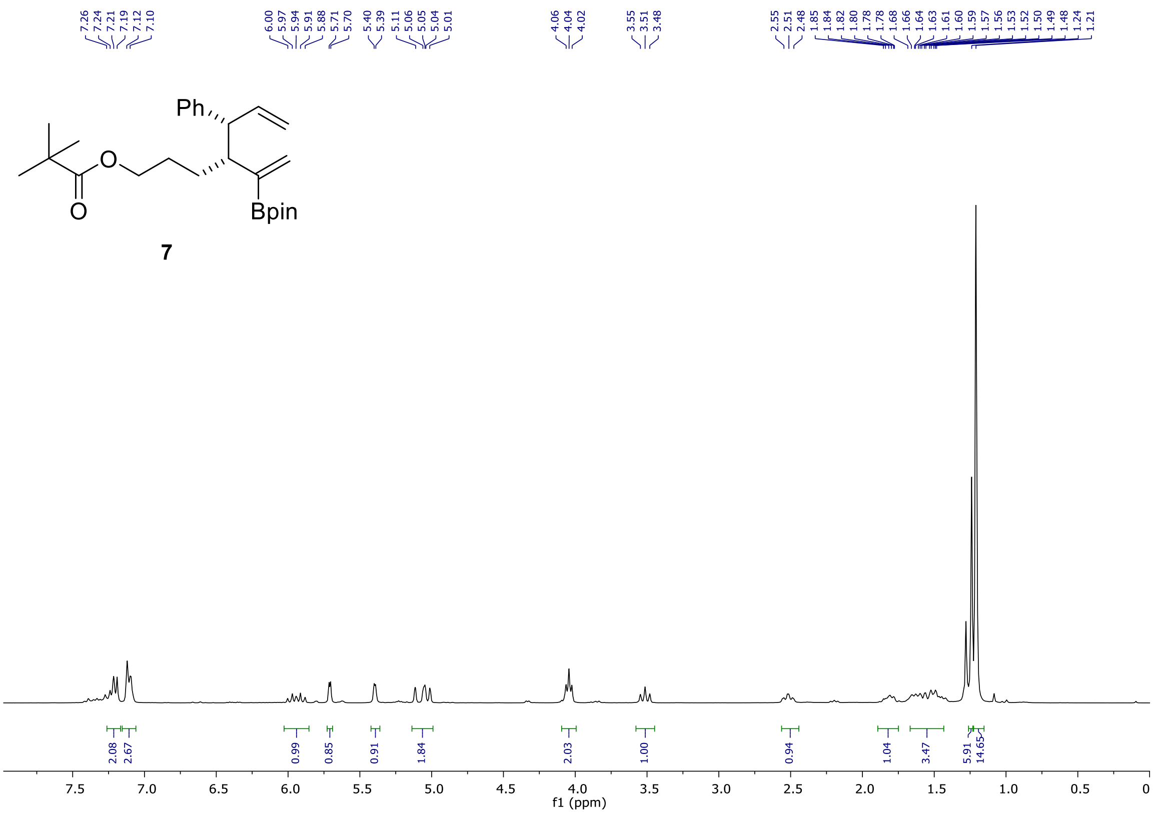

¹³C NMR (75 MHz, $CDCl_3$)

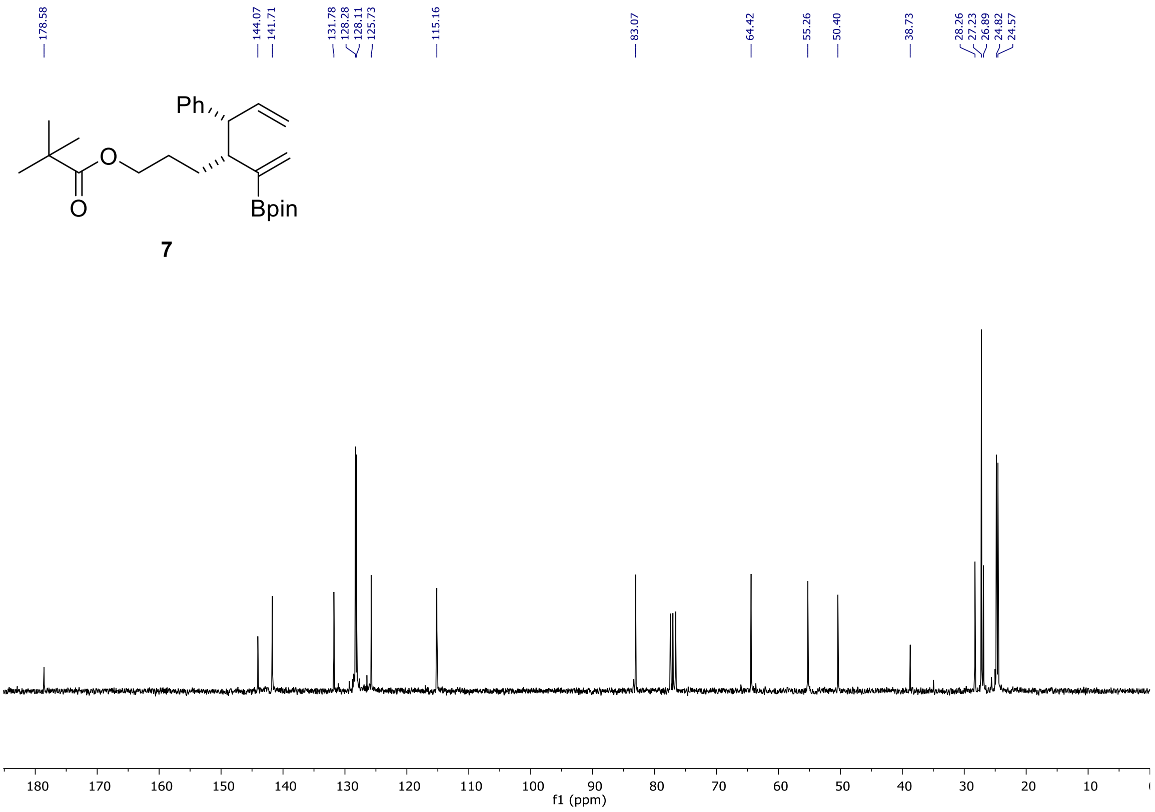

^{1}H NMR (300 MHz, $CDCl_3$)

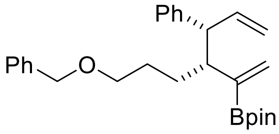

8

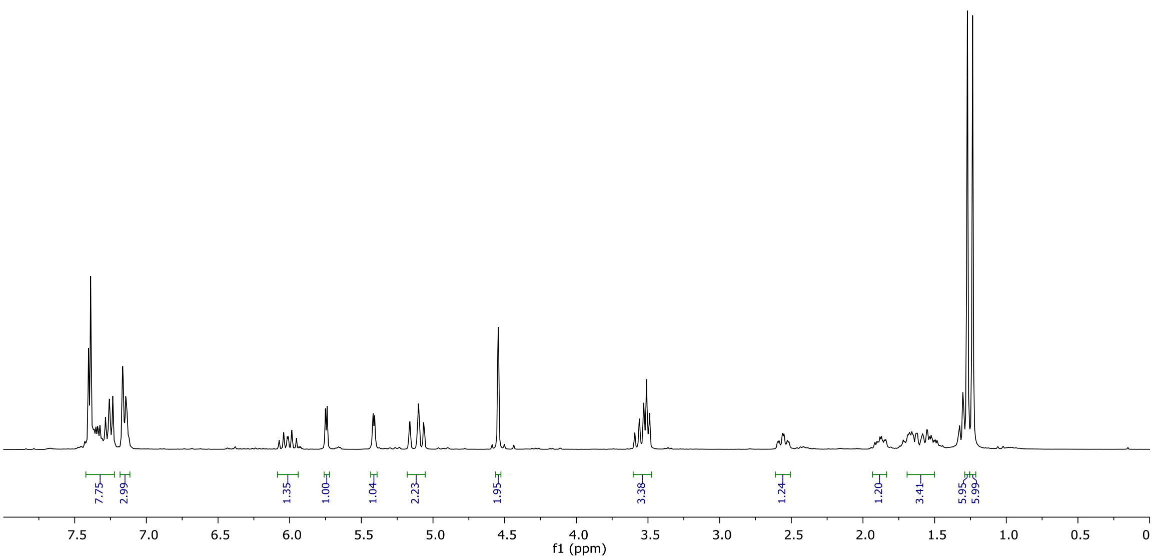

^{13}C NMR (75 MHz, $CDCl_3$)

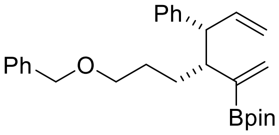

8

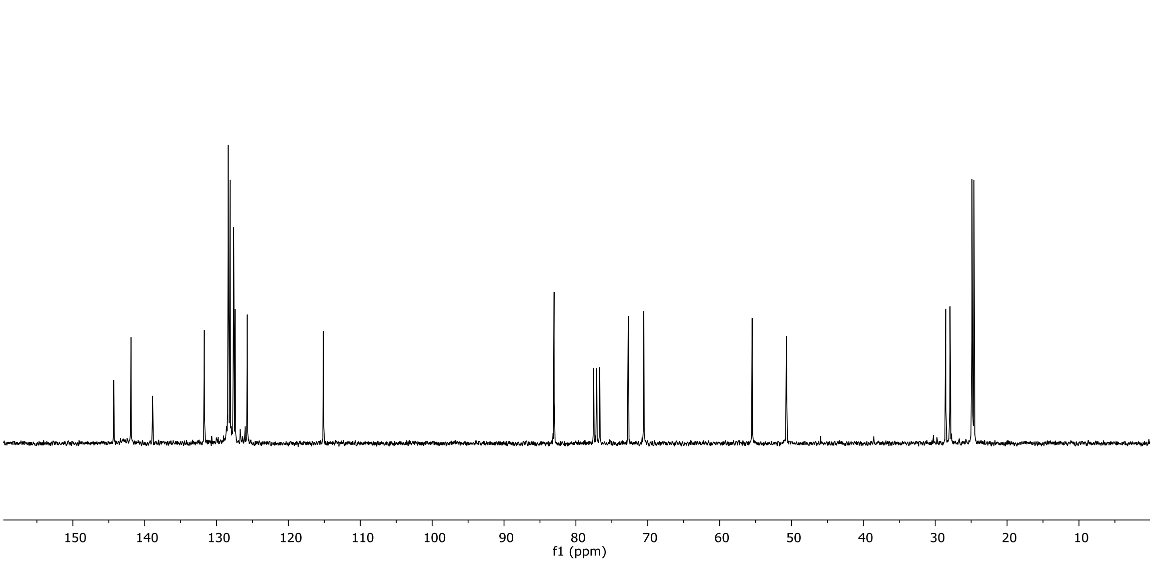

^{1}H NMR (300 MHz, $CDCl_3$)

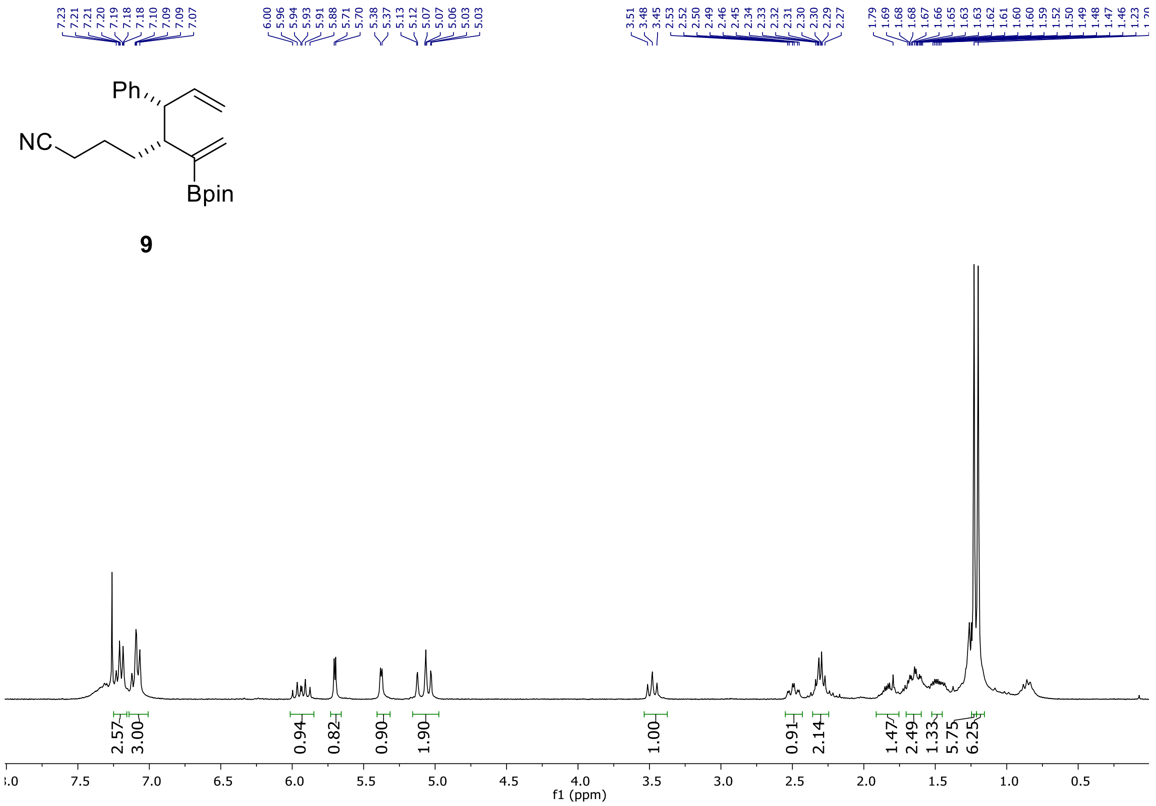

^{13}C NMR (75 MHz, $CDCl_3$)

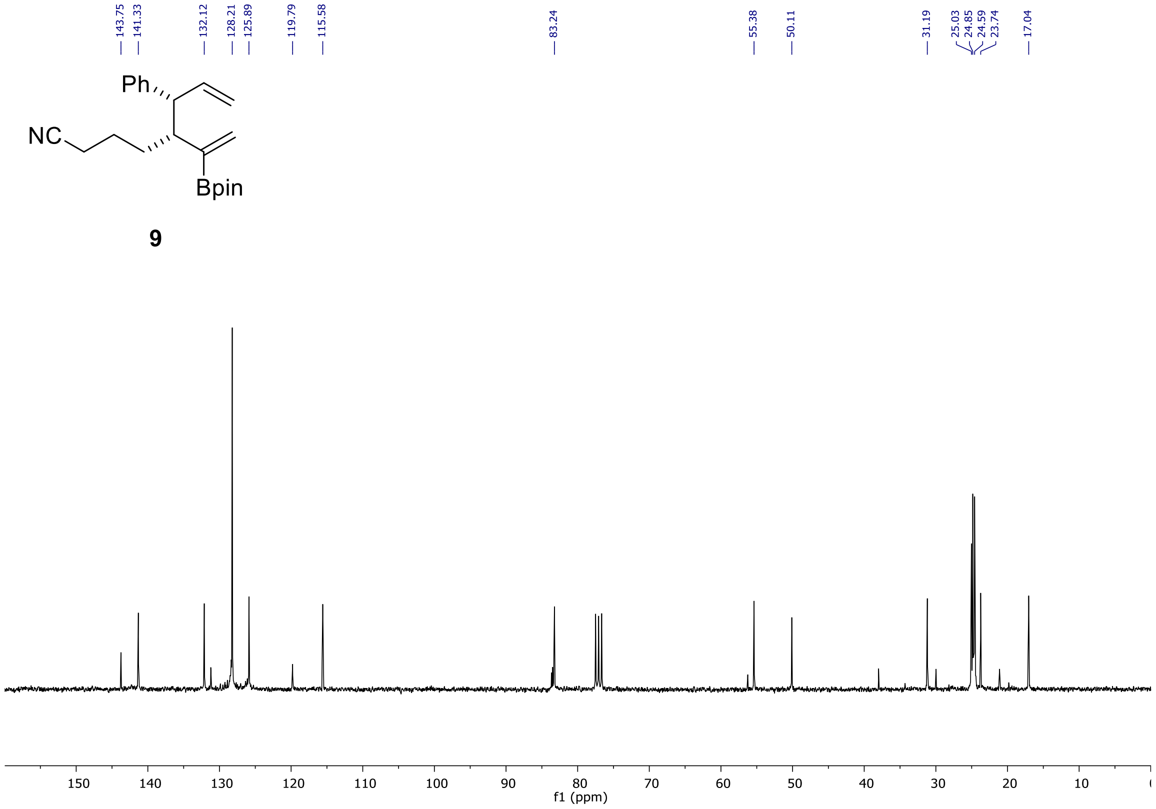

^{1}H NMR (300 MHz, $CDCl_3$)

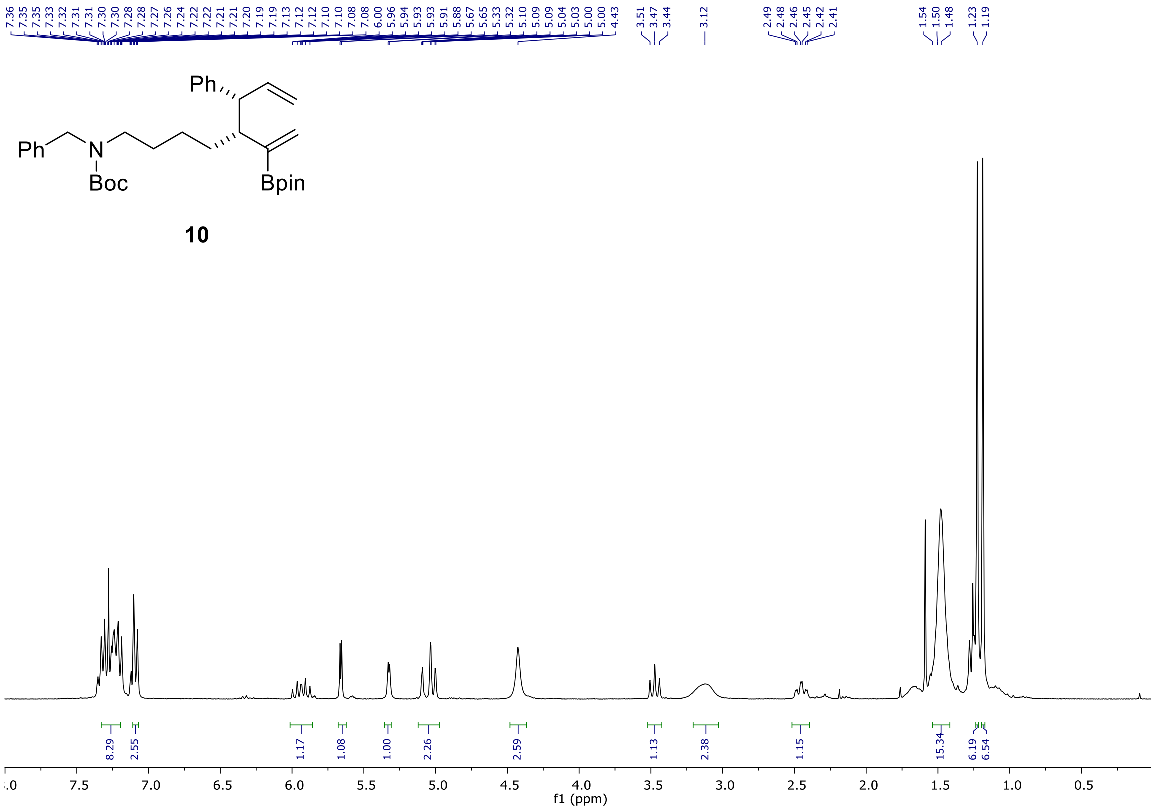

^{13}C NMR (126 MHz, $CDCl_3$)

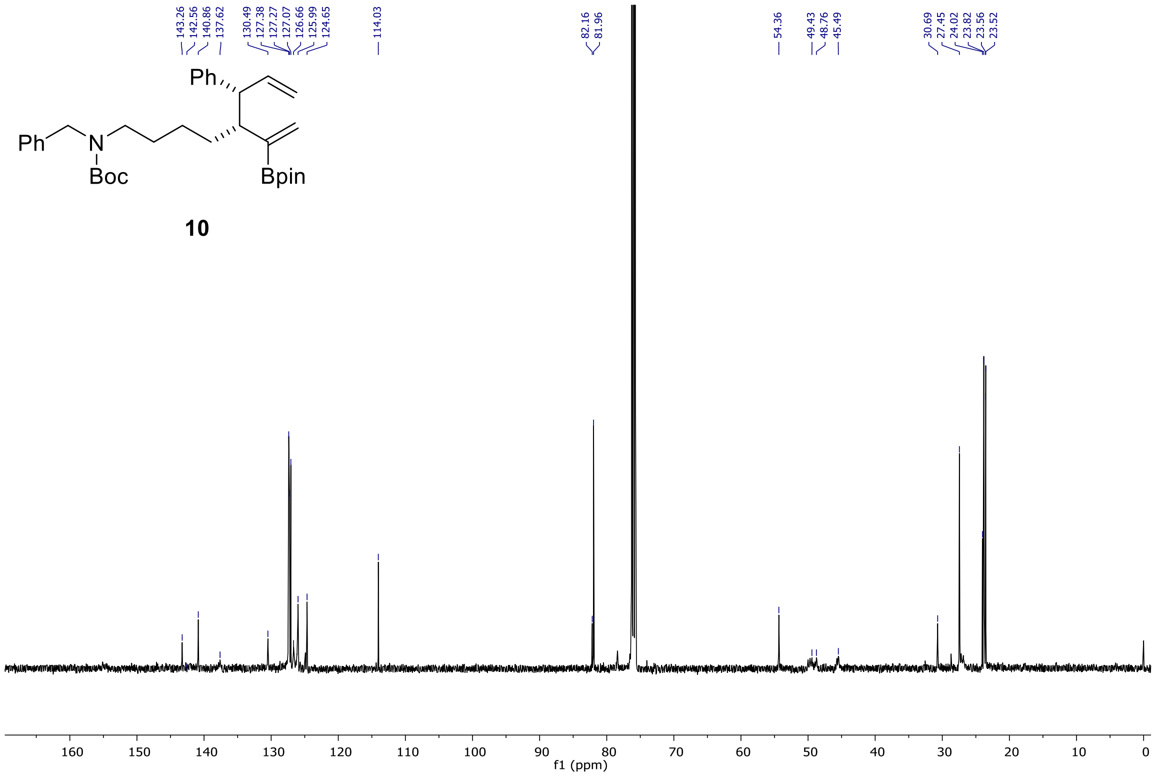

*Because of time relaxation, some carbon atoms were not well defined.

^{1}H NMR (300 MHz, $CDCl_3$)

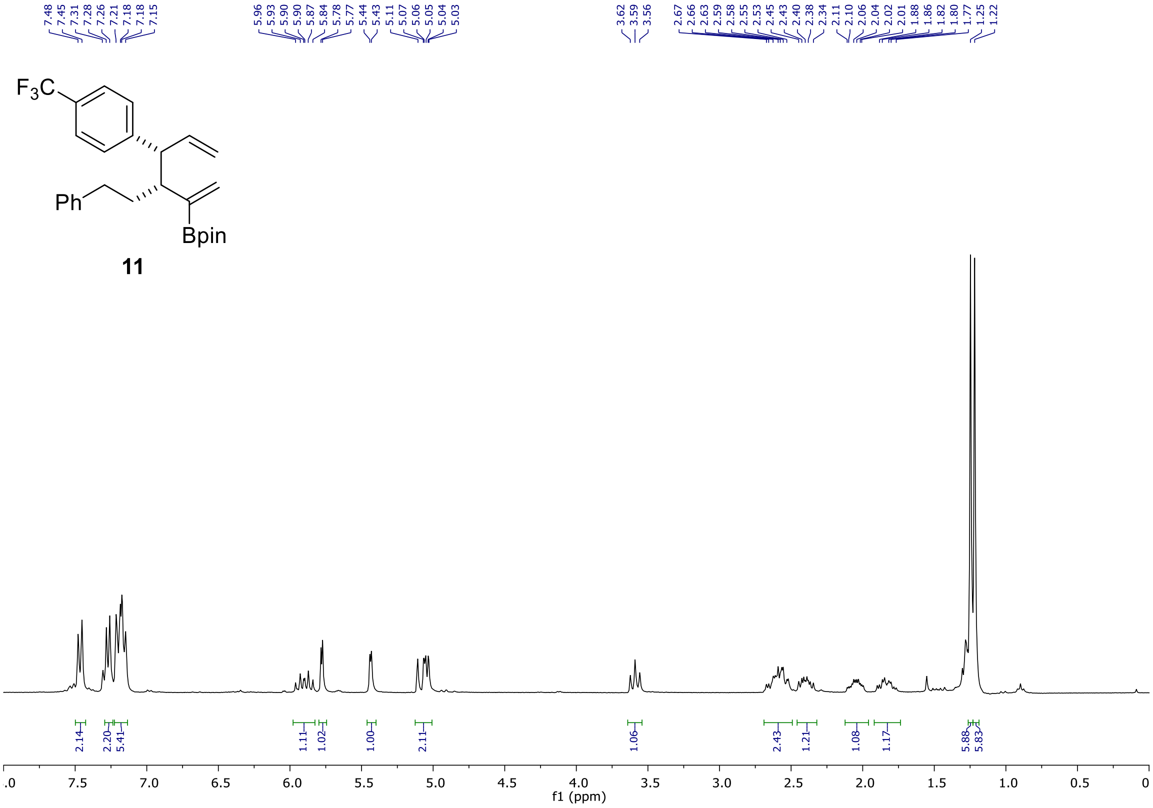

^{13}C NMR (75 MHz, $CDCl_3$)

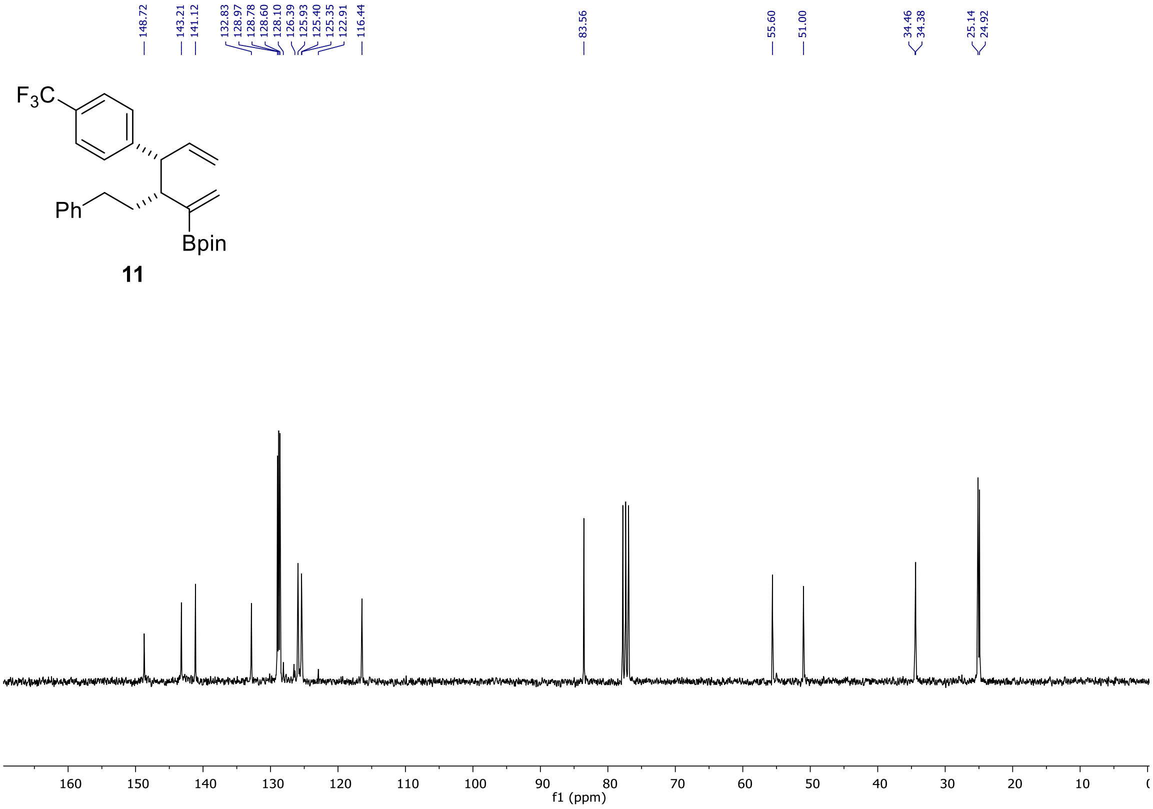

^{1}H NMR (500 MHz, CDCl$_3$)

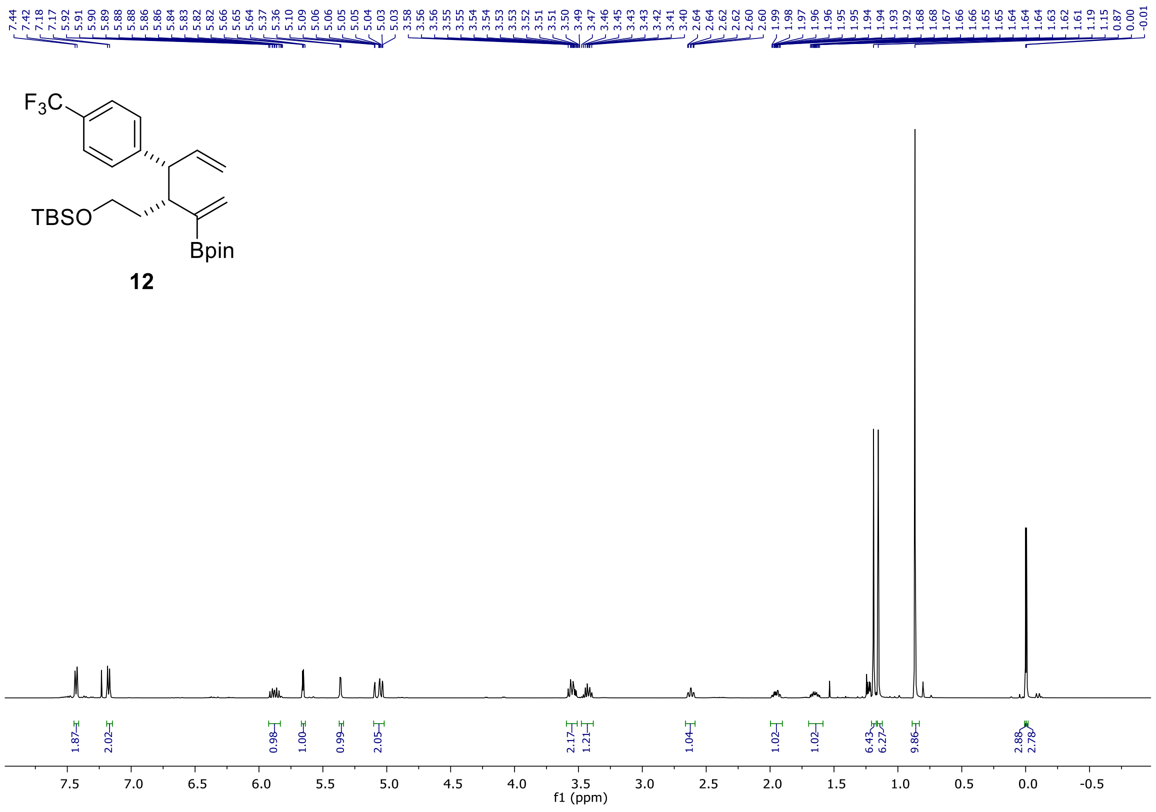

^{13}C NMR (75 MHz, CDCl$_3$)

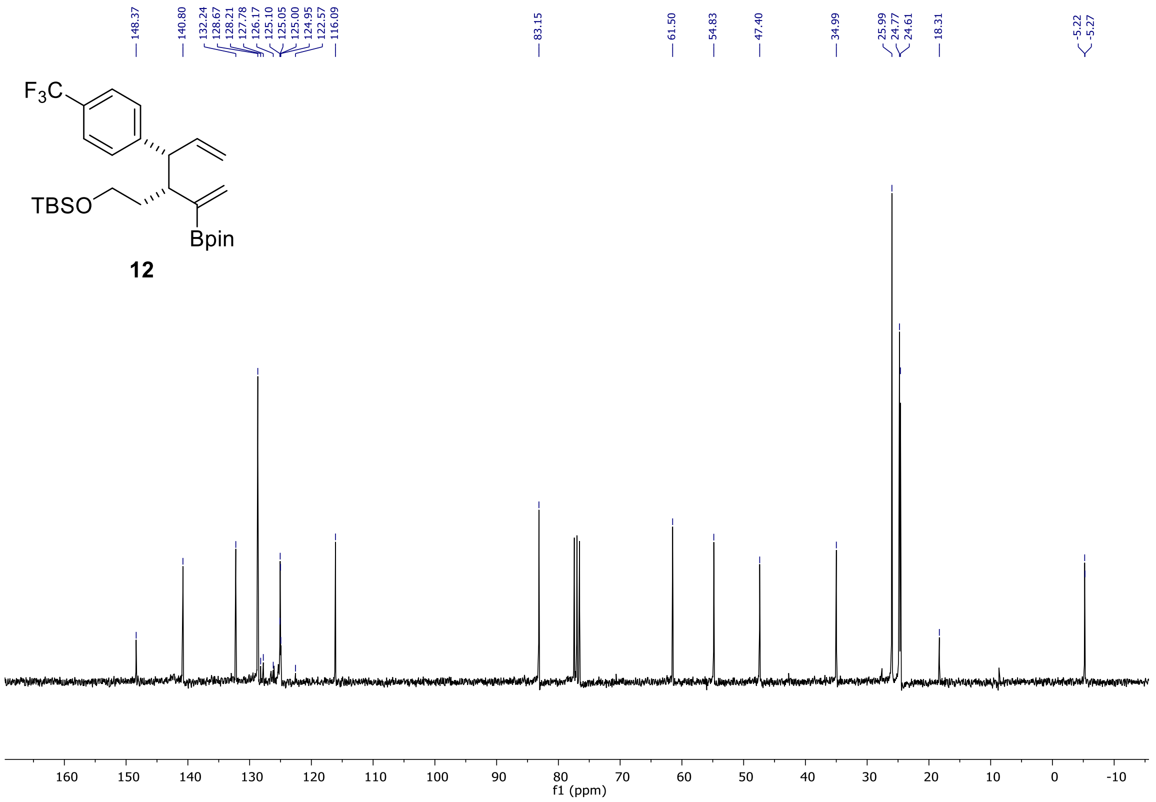

^{1}H NMR (300 MHz, $CDCl_3$)

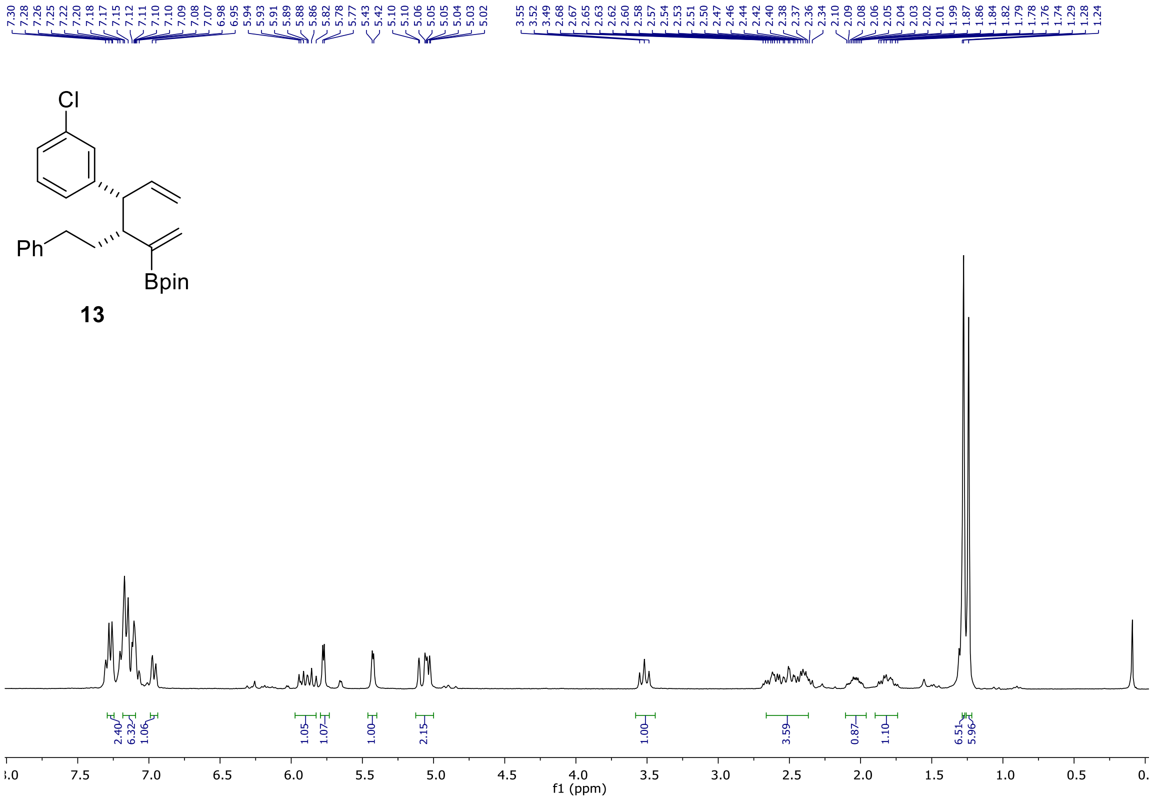

^{13}C NMR (75 MHz, $CDCl_3$)

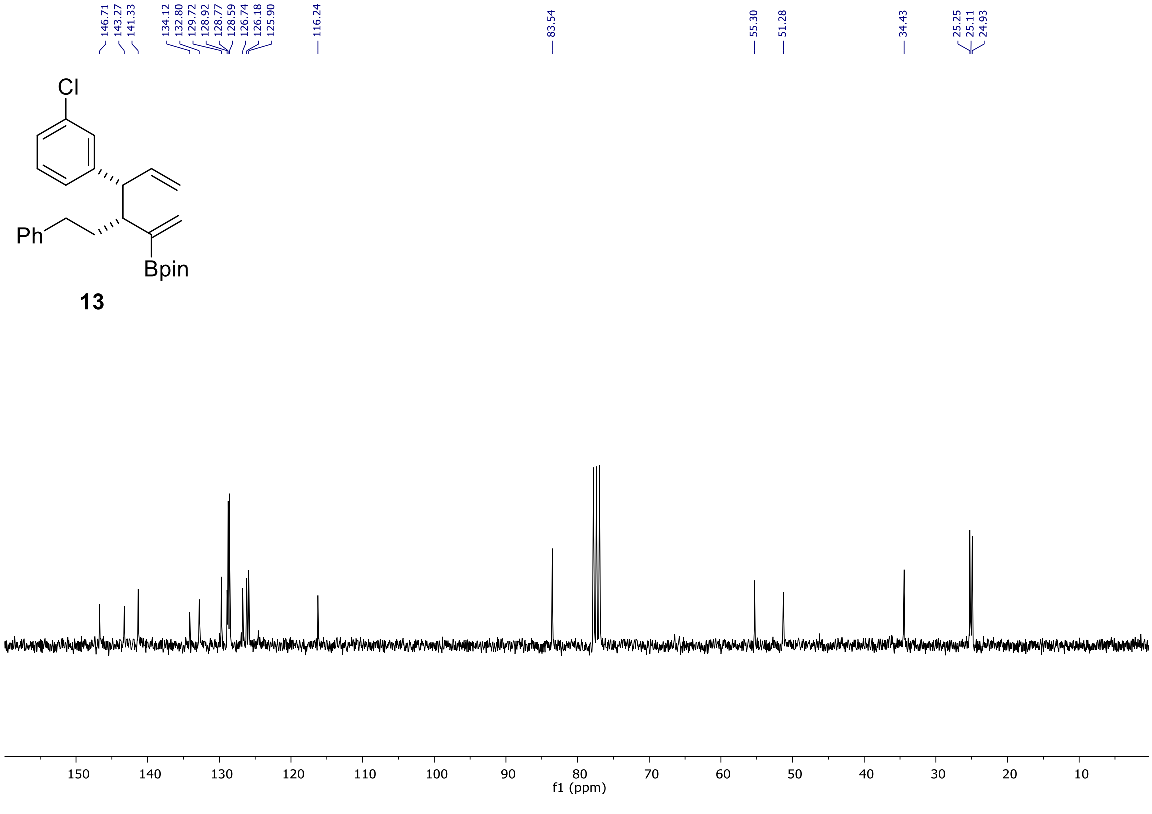

¹H NMR (300 MHz, $CDCl_3$)

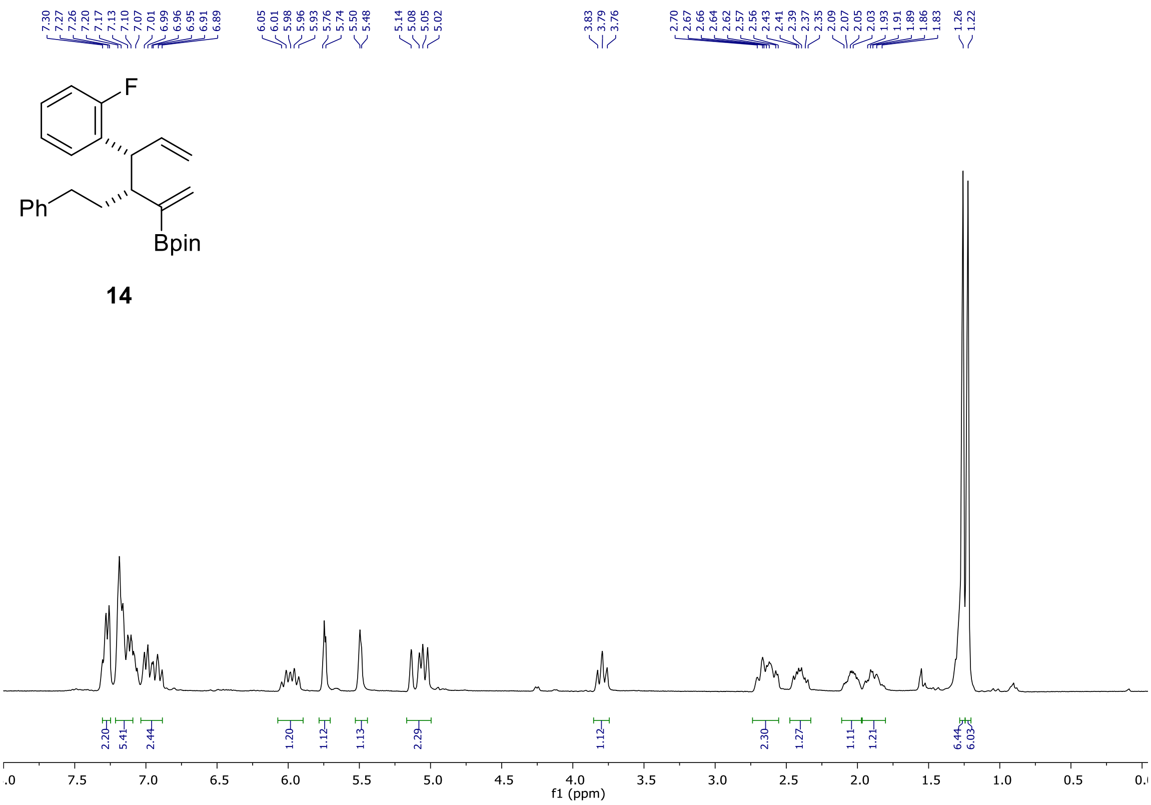

¹³C NMR (75 MHz, $CDCl_3$)

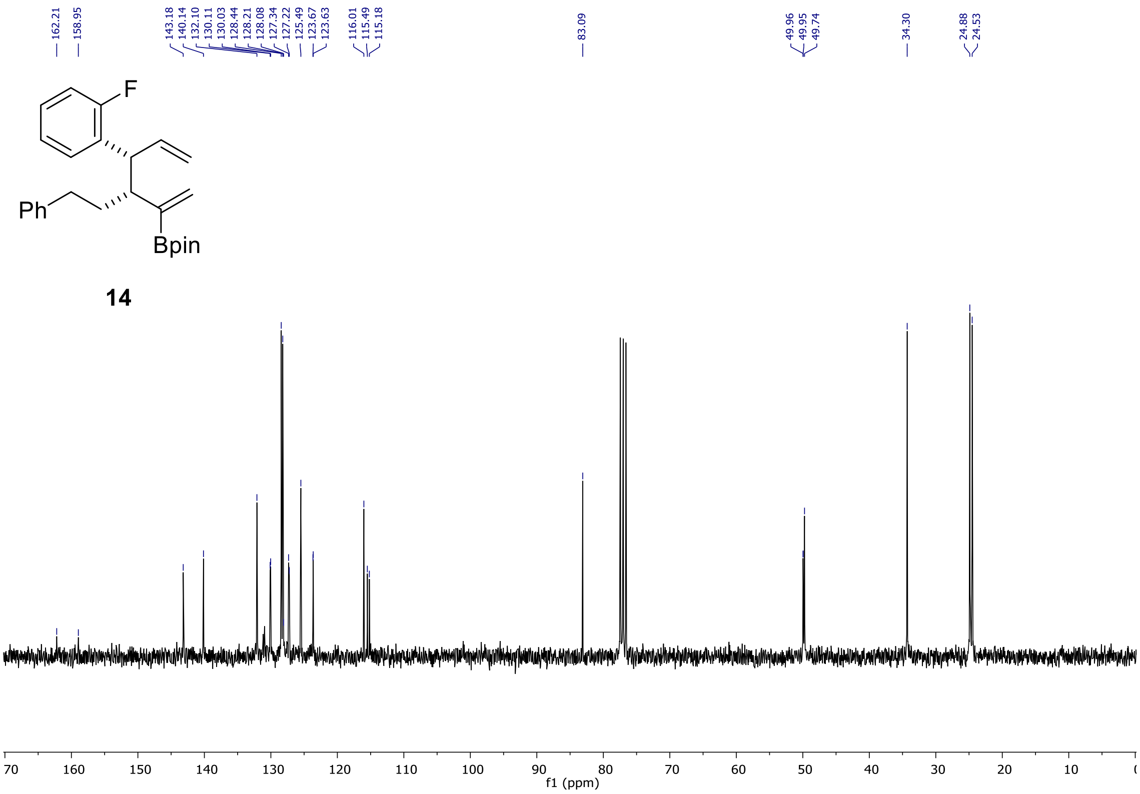

^{1}H NMR (300 MHz, $CDCl_3$)

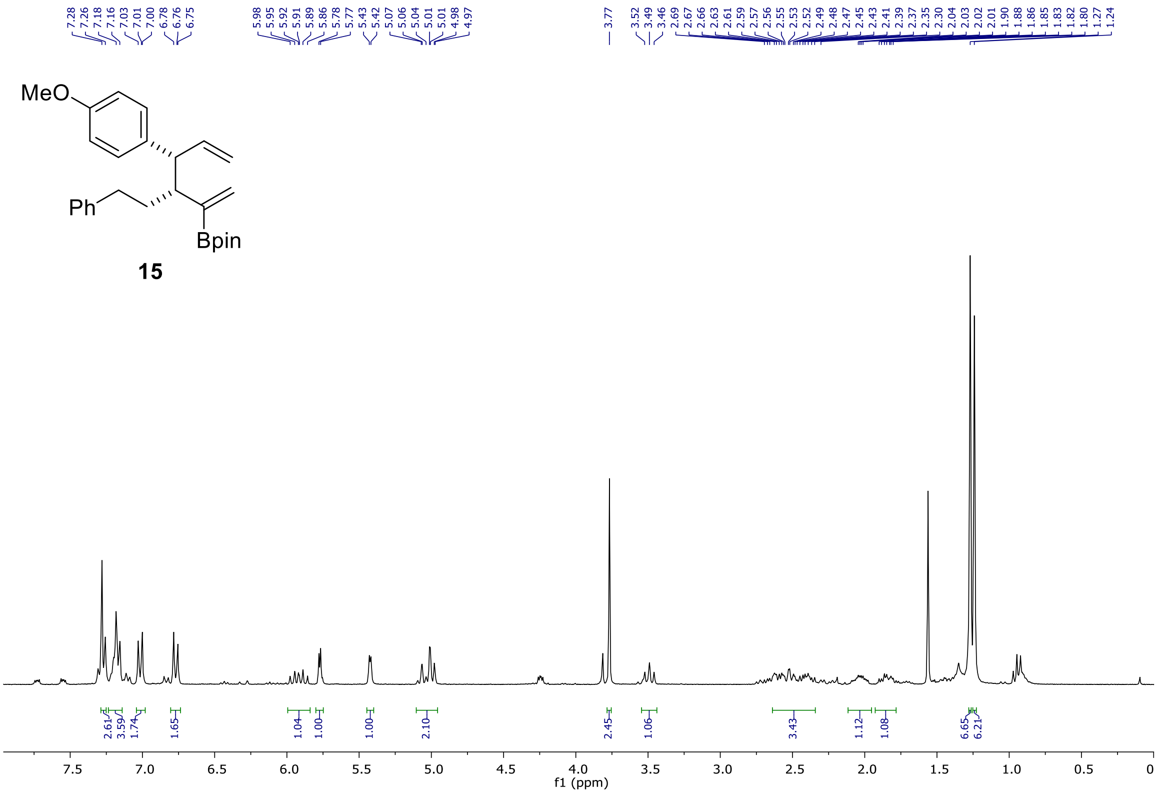

^{13}C NMR (75 MHz, $CDCl_3$)

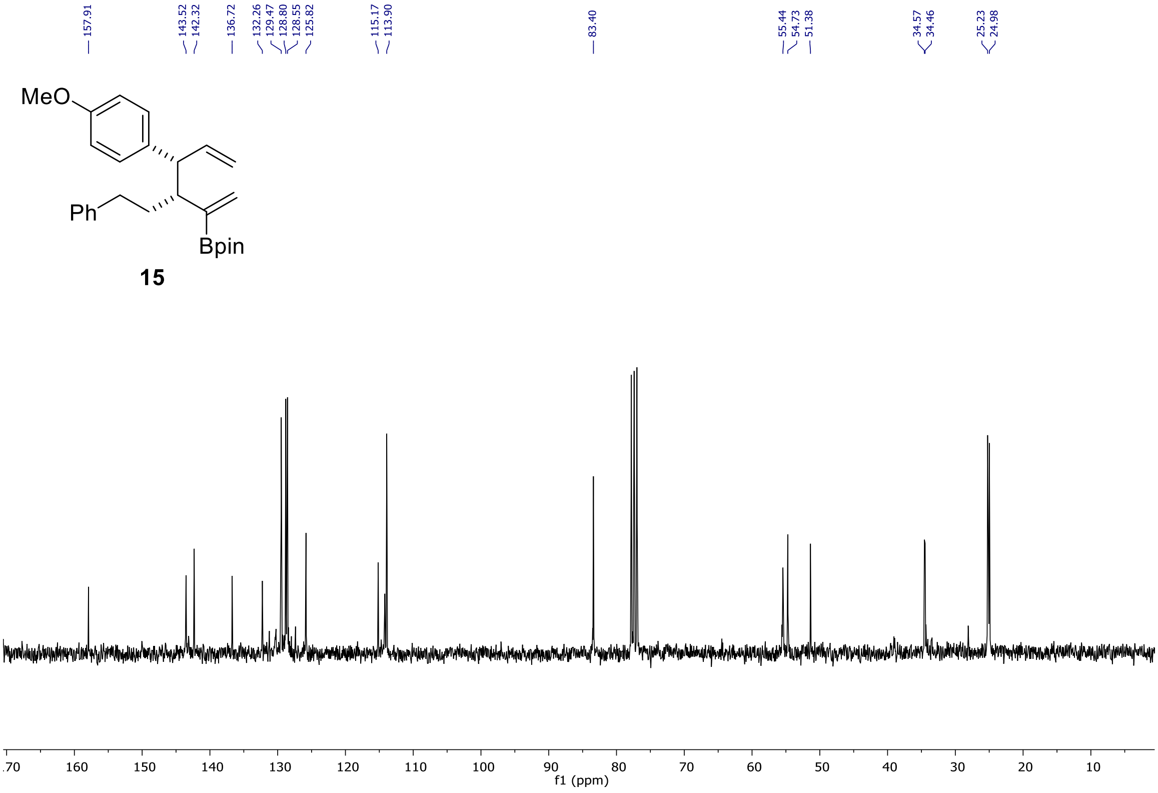

^{1}H NMR (300 MHz, $CDCl_3$)

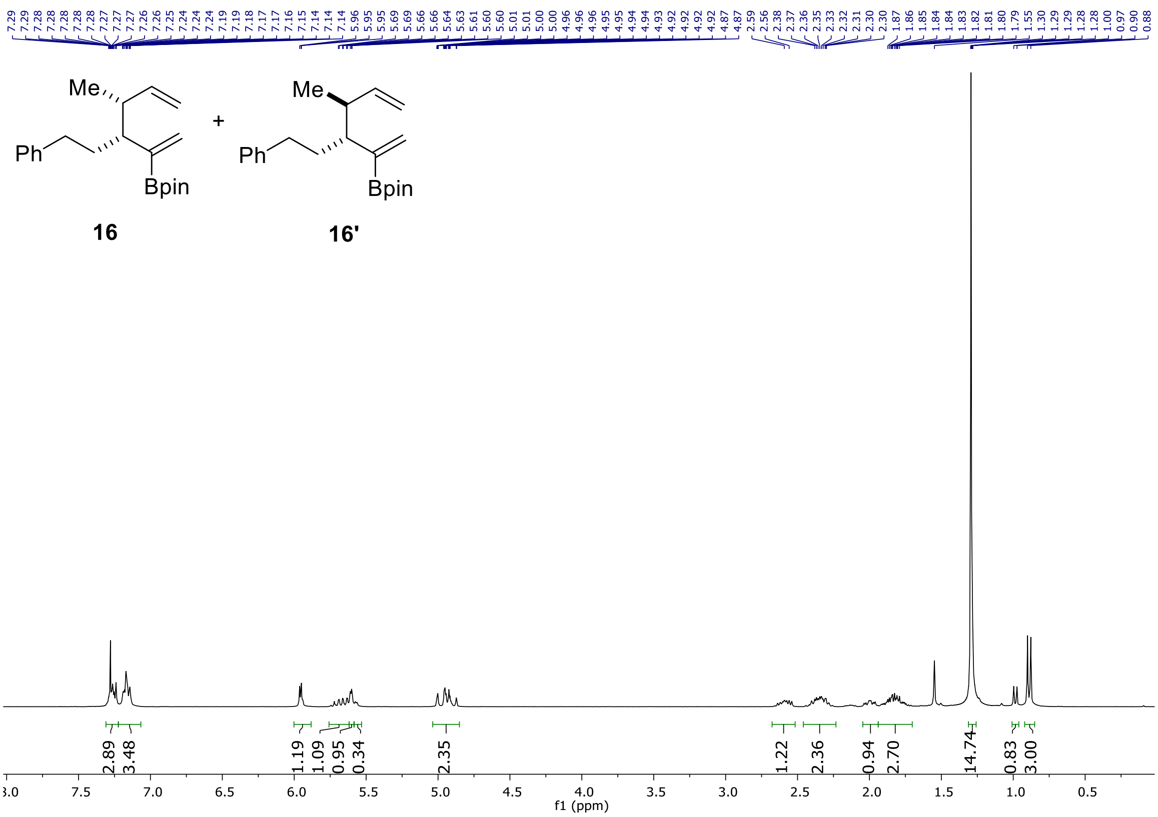

^{13}C NMR (75 MHz, $CDCl_3$)

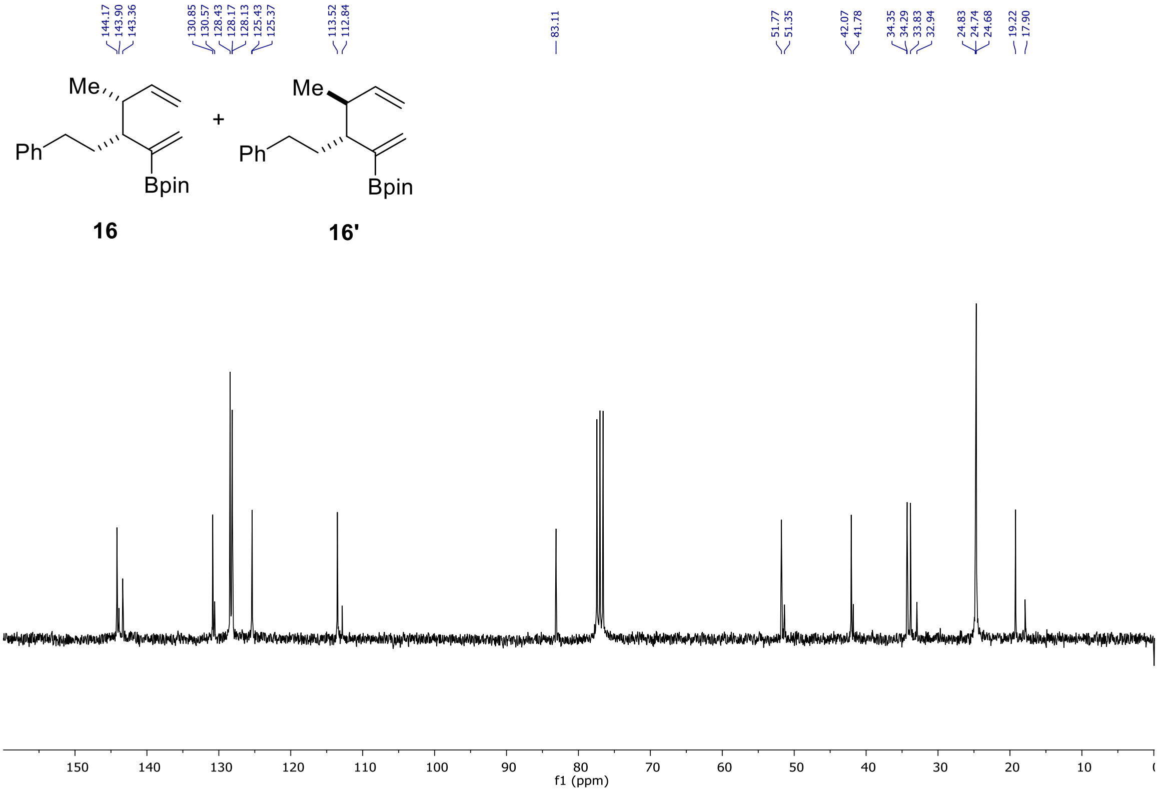

¹H NMR (300 MHz, $CDCl_3$)

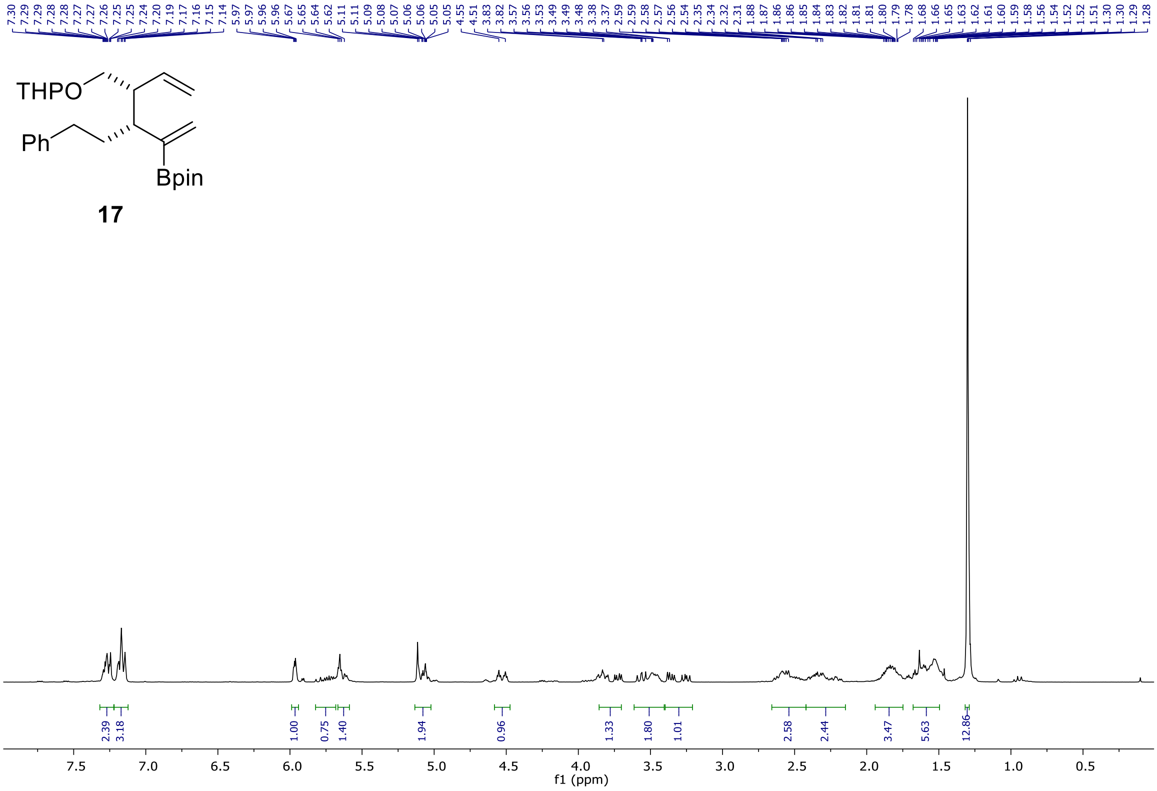

¹³C NMR (75 MHz, $CDCl_3$)

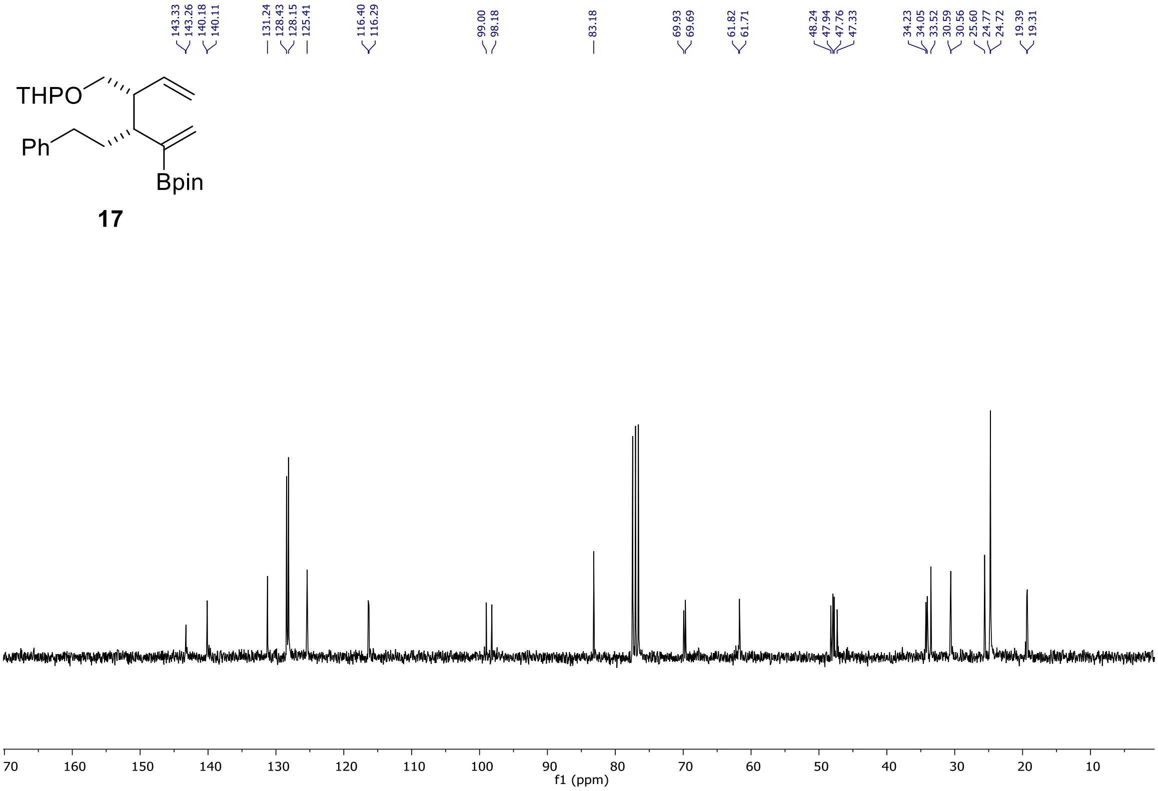

^{1}H NMR (300 MHz, $CDCl_3$)

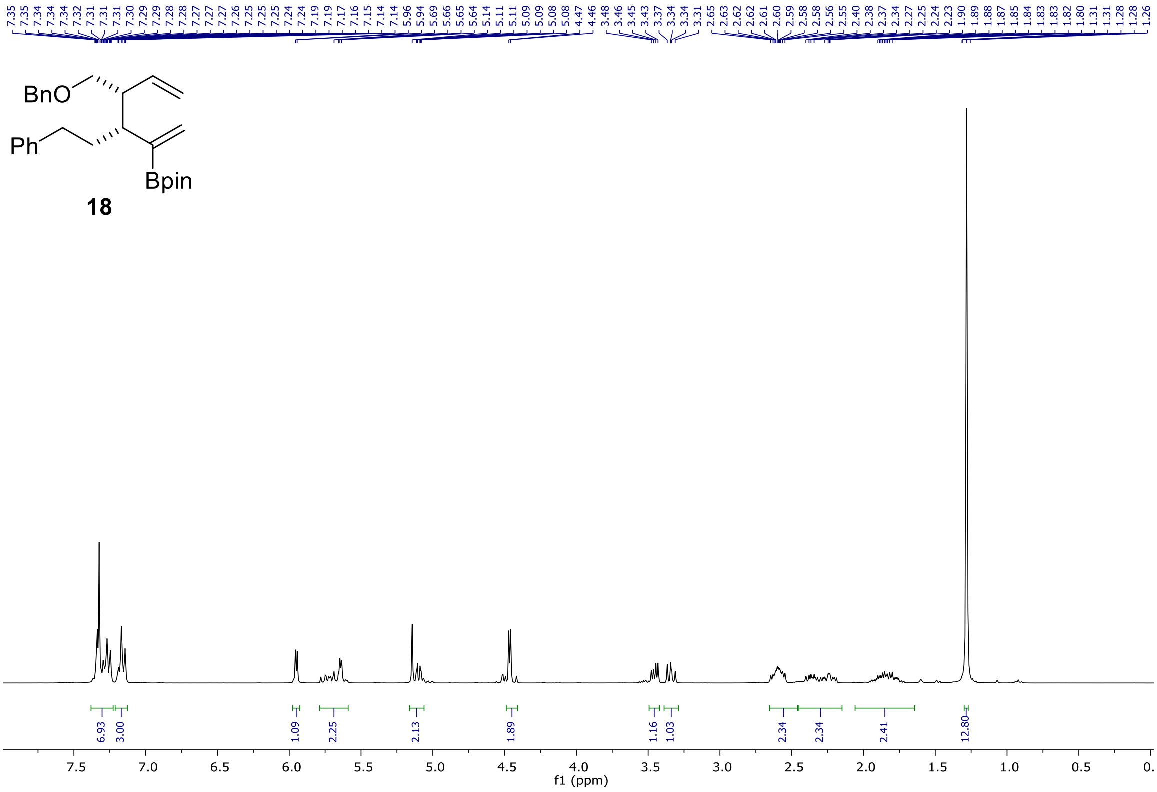

^{13}C NMR (75 MHz, $CDCl_3$)

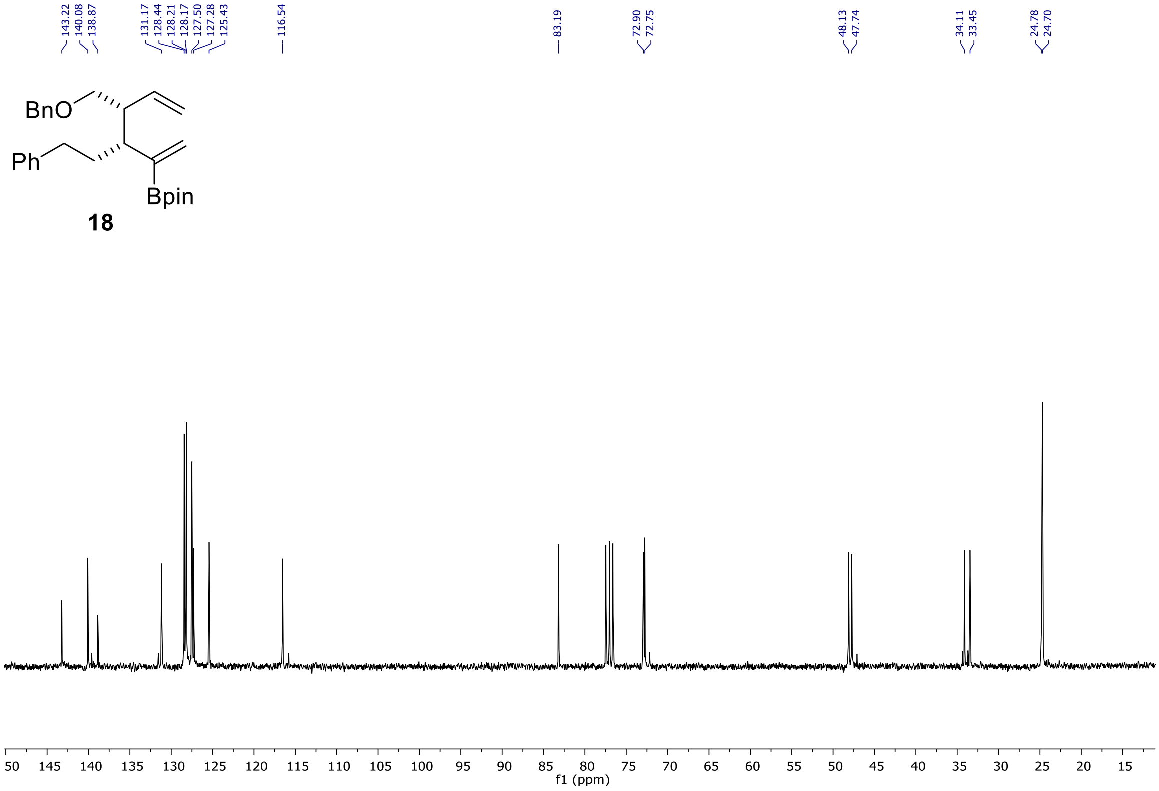

^{1}H NMR (300 MHz, $CDCl_3$)

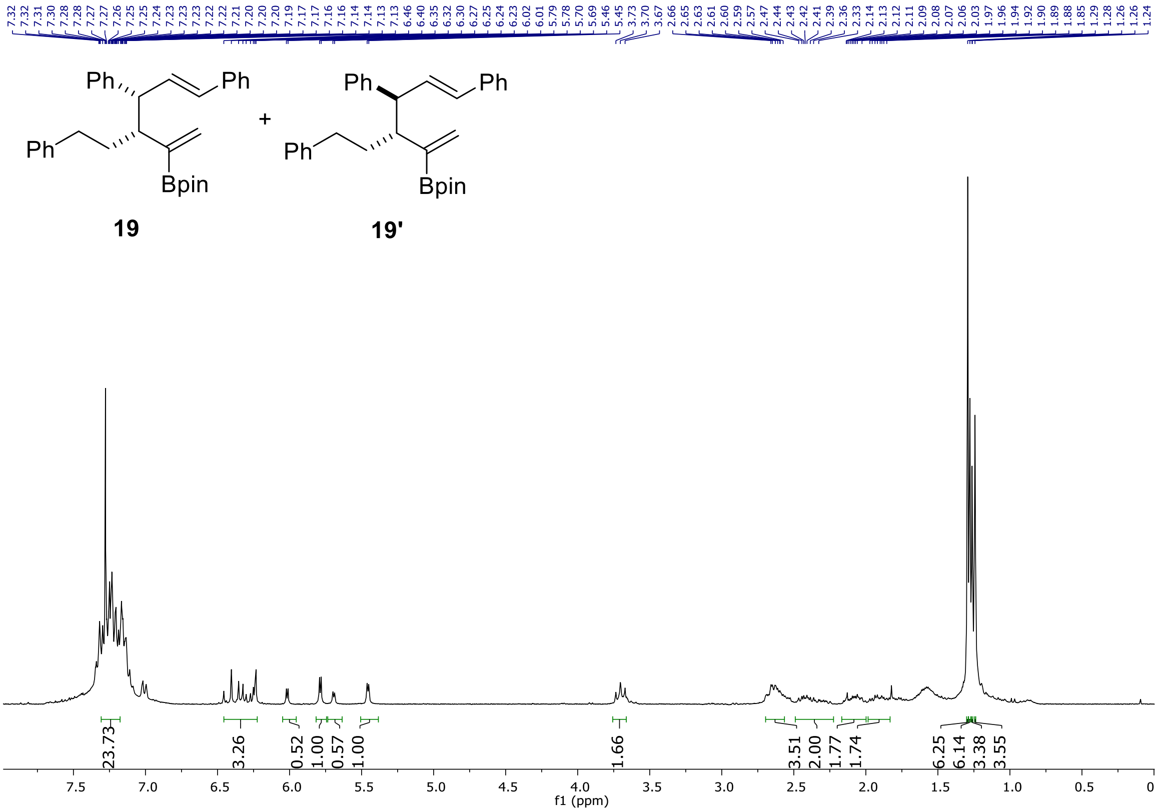

^{13}C NMR (75 MHz, $CDCl_3$)

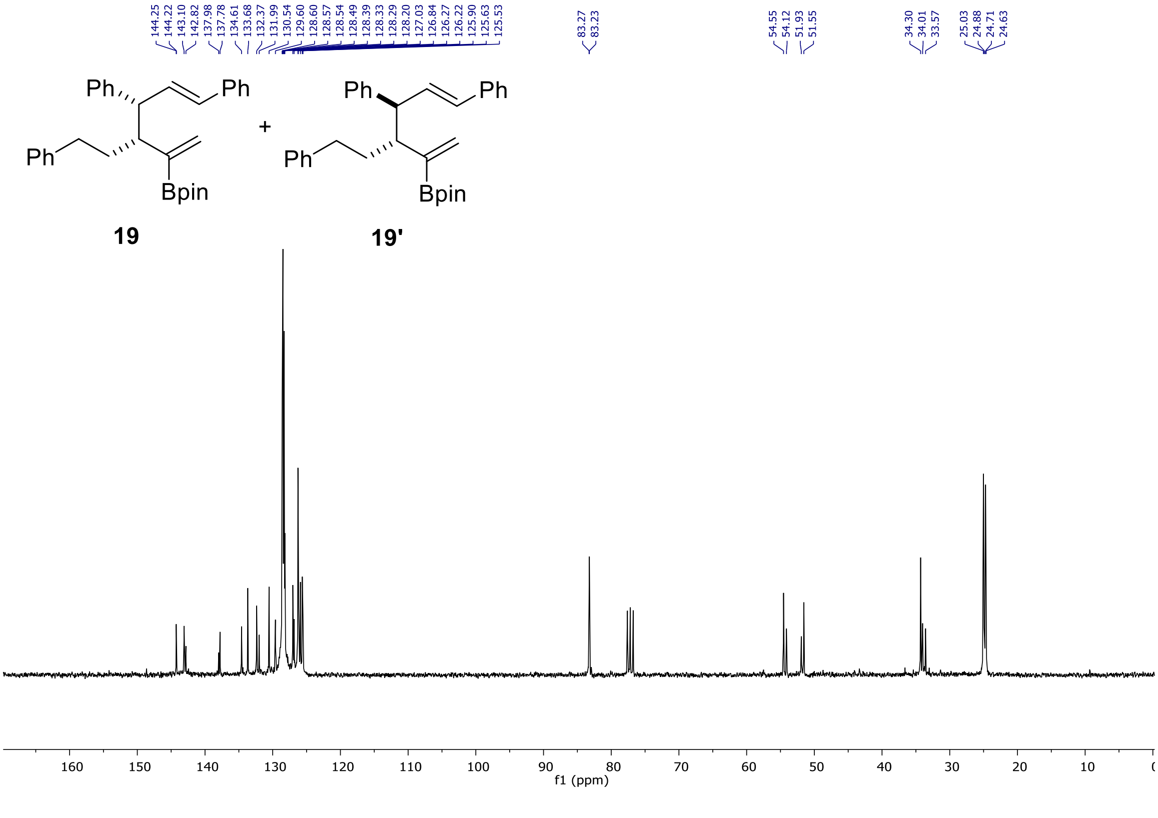

^{1}H NMR (300 MHz, $CDCl_3$)

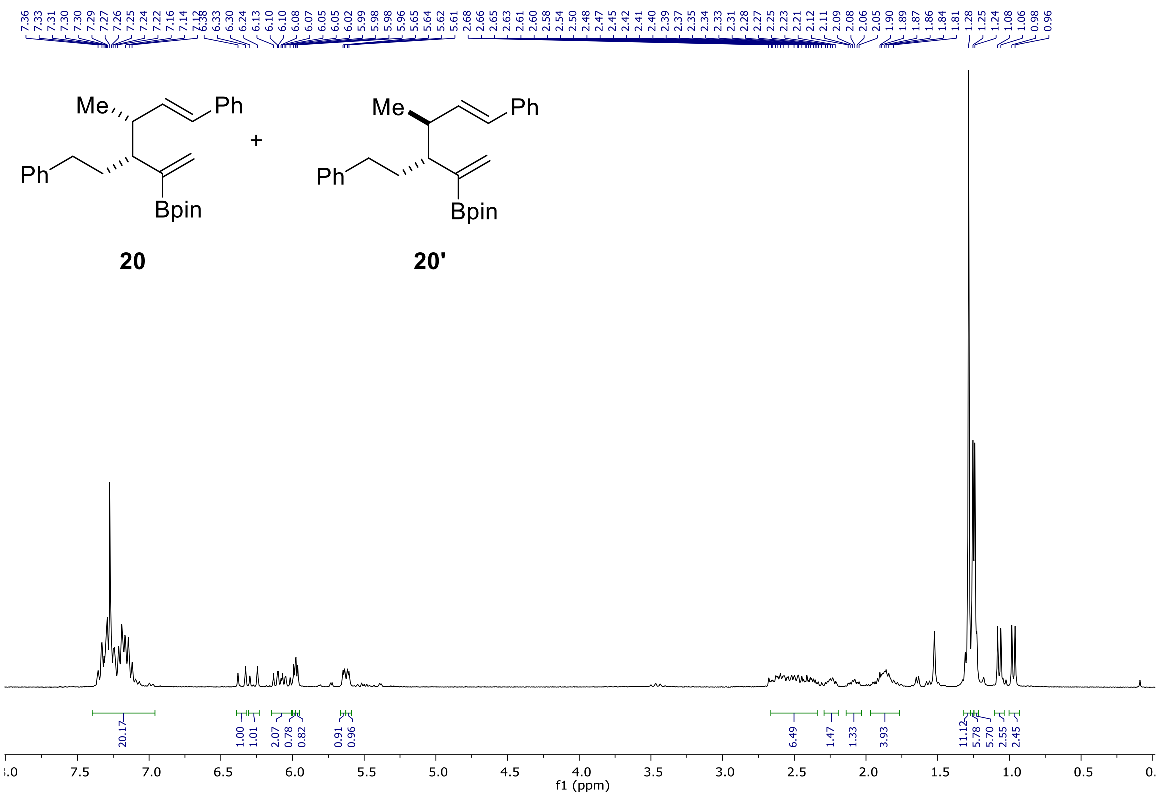

^{13}C NMR (75 MHz, $CDCl_3$)

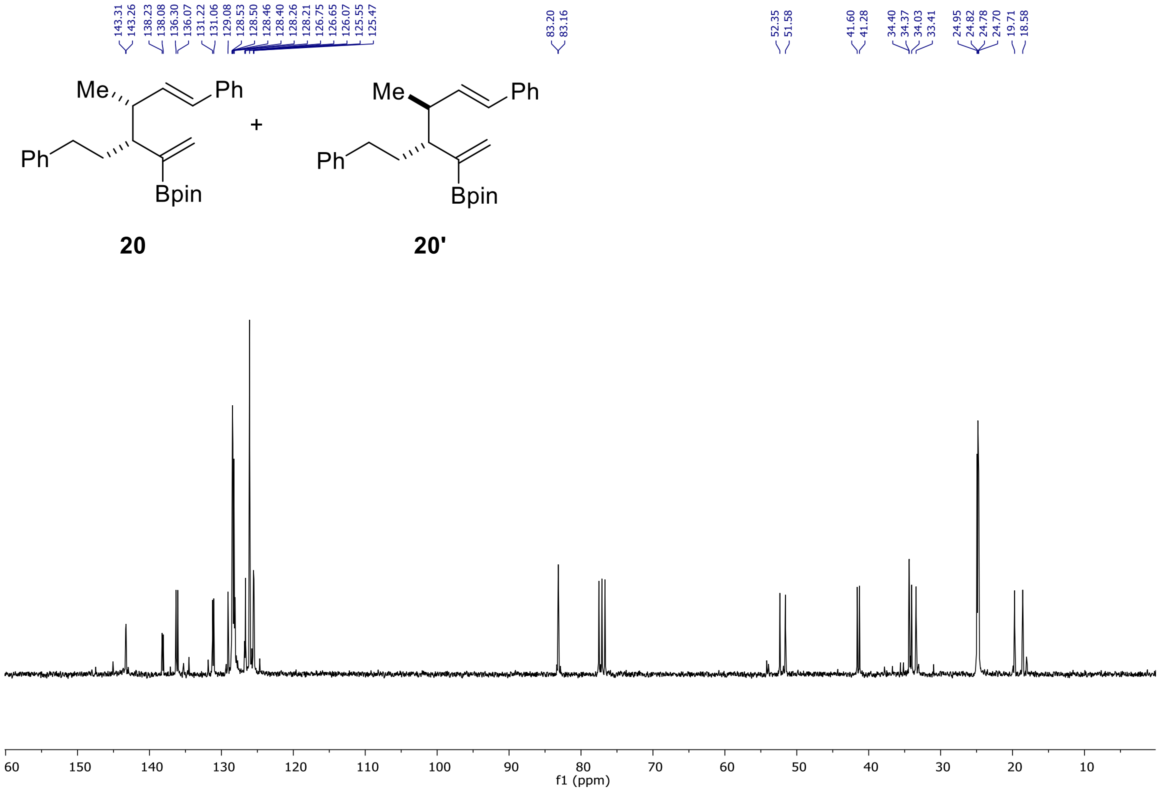

^{1}H NMR (300 MHz, $CDCl_3$)

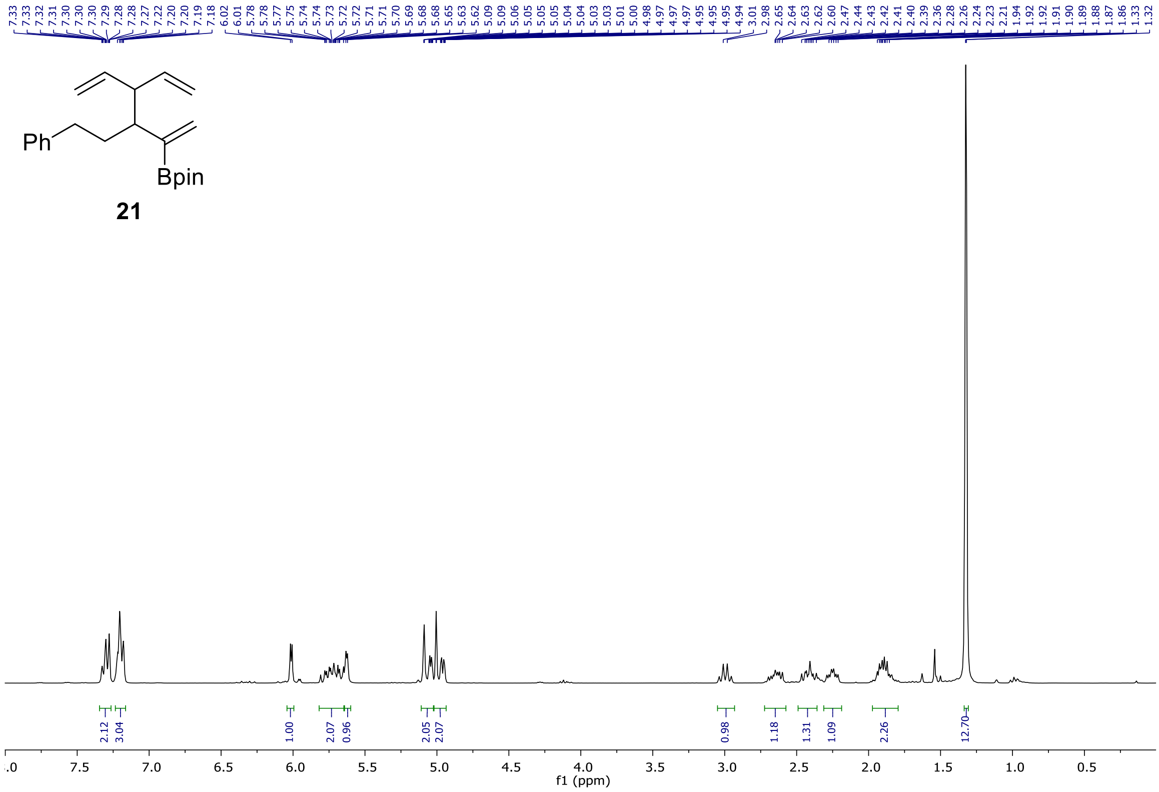

21

^{13}C NMR (75 MHz, $CDCl_3$)

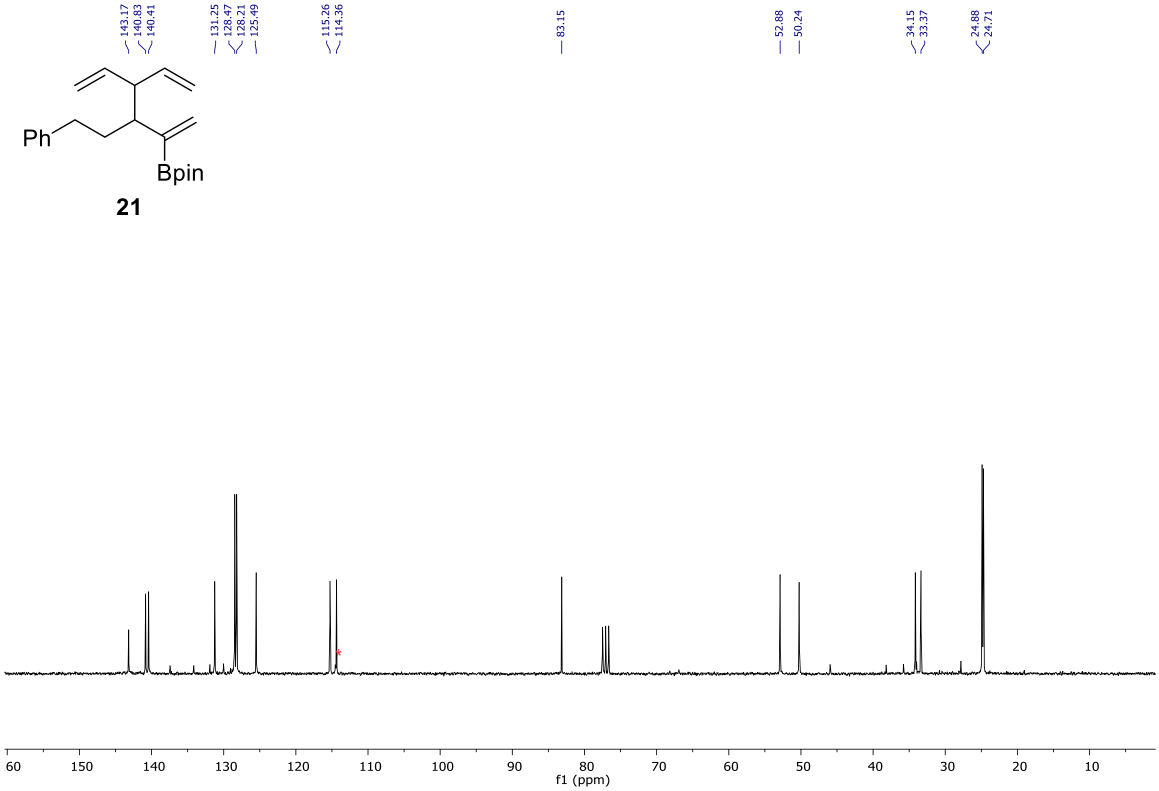

21

^{1}H NMR (500 MHz, $CDCl_3$)

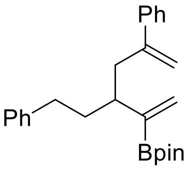

22

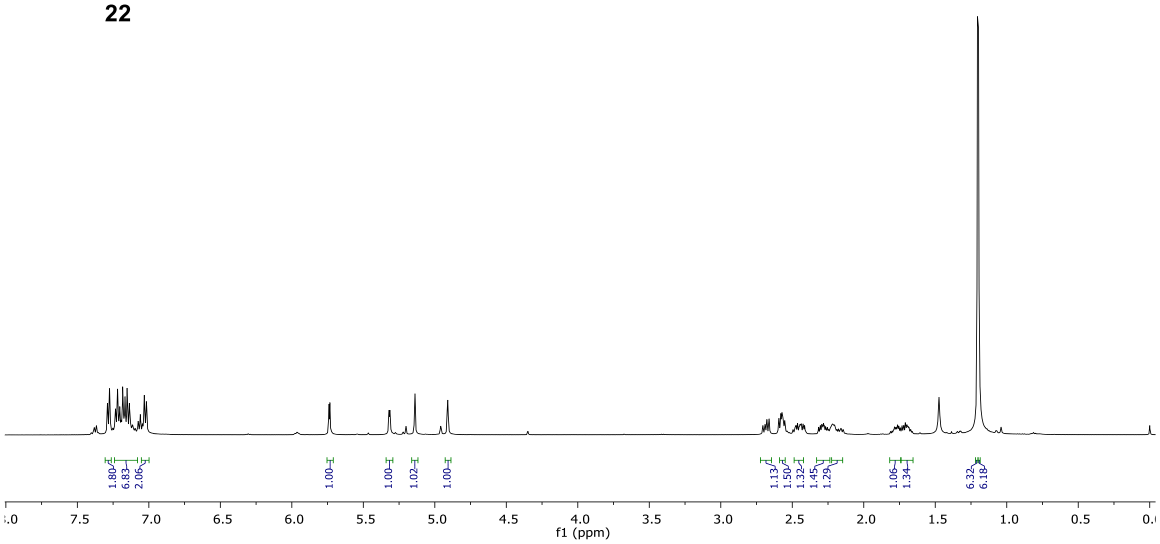

^{13}C NMR (126 MHz, $CDCl_3$)

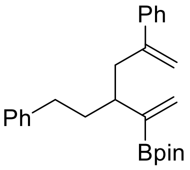

22

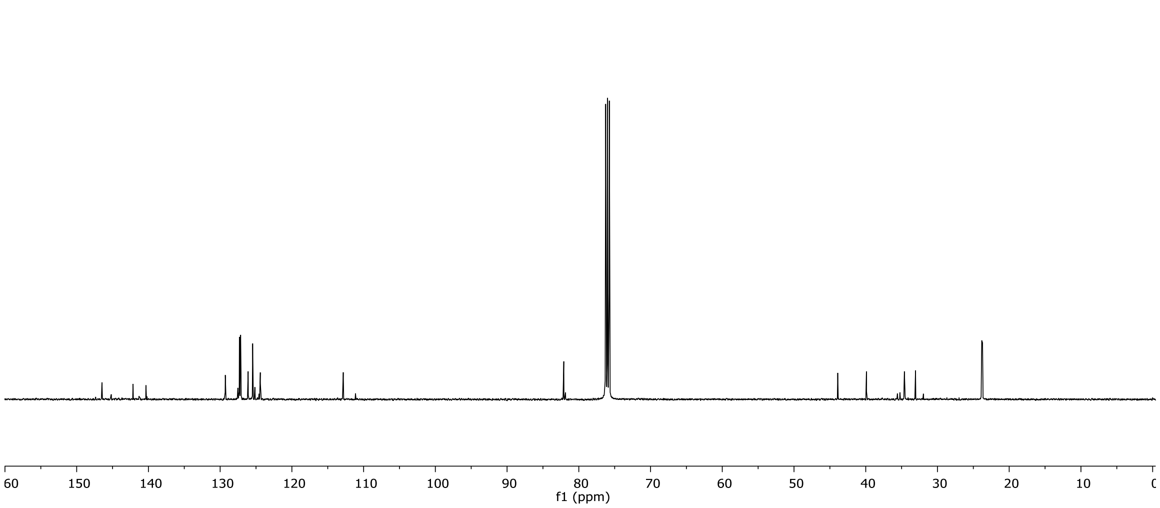

^{1}H NMR (300 MHz, $CDCl_3$)

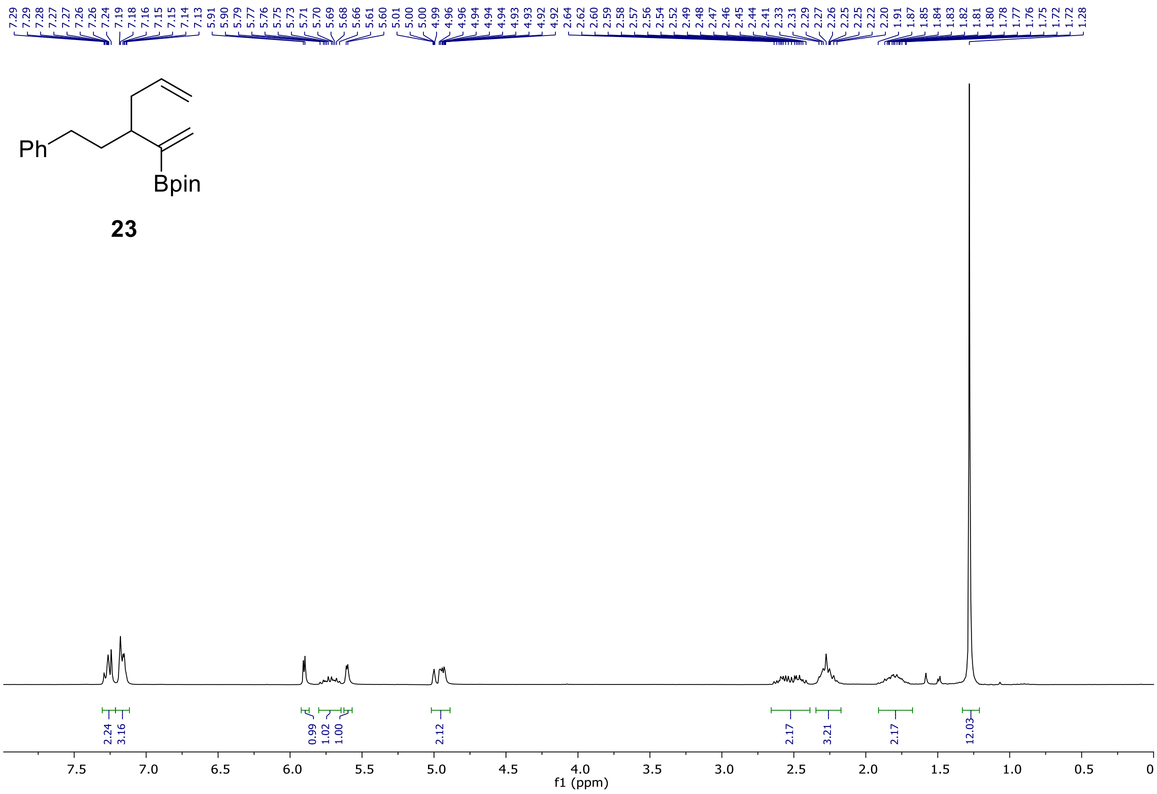

^{13}C NMR (75 MHz, $CDCl_3$)

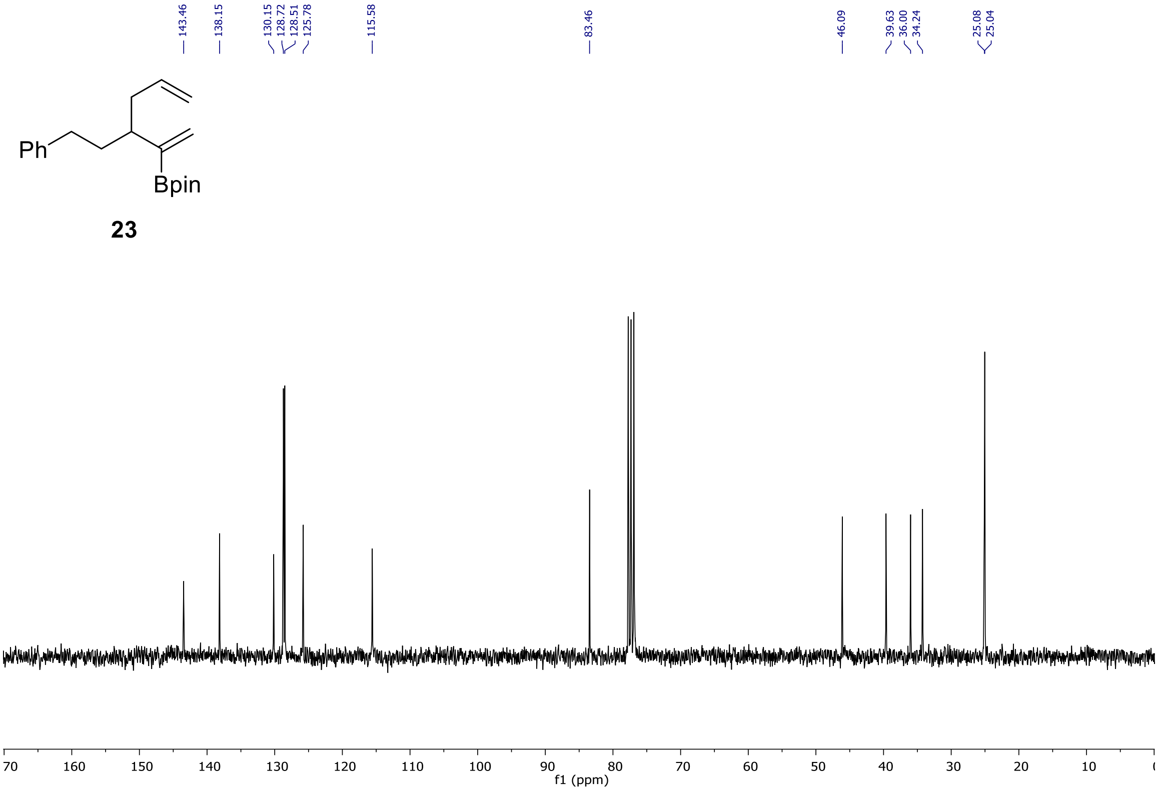

^{1}H NMR (500 MHz, $CDCl_3$)

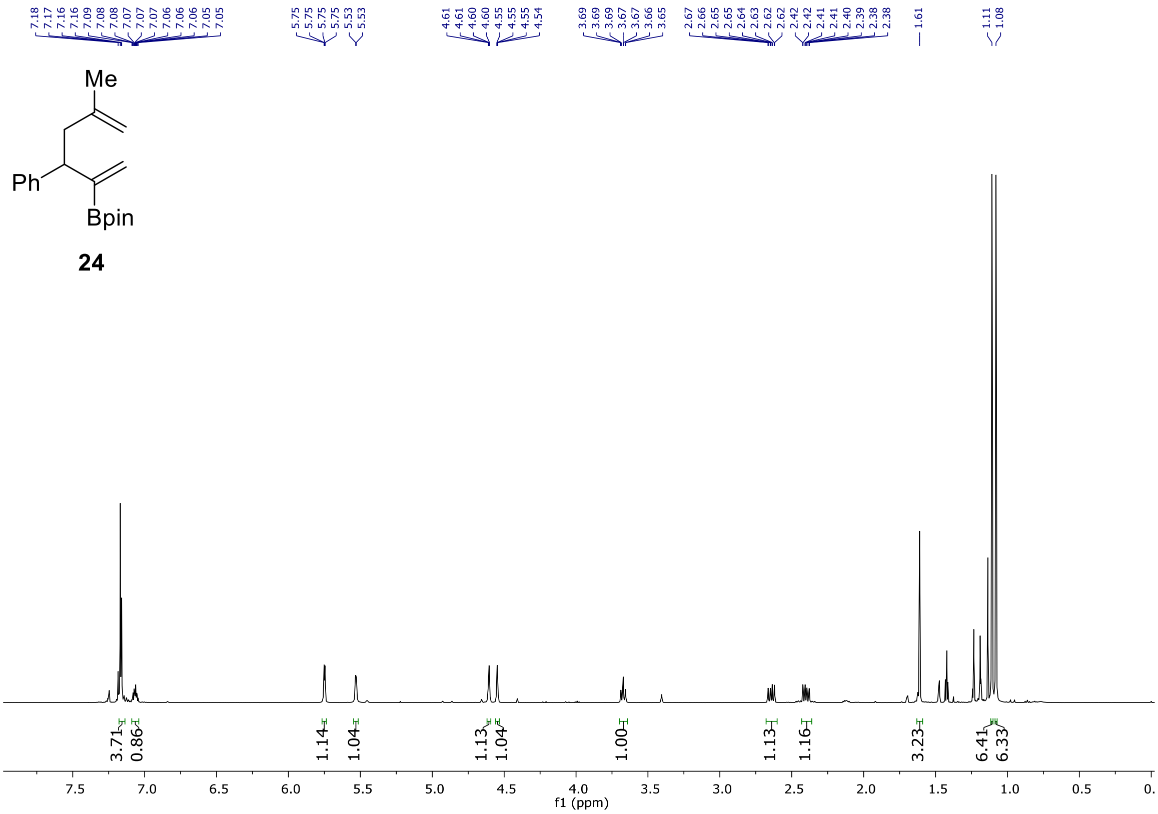

^{13}C NMR (126 MHz, $CDCl_3$)

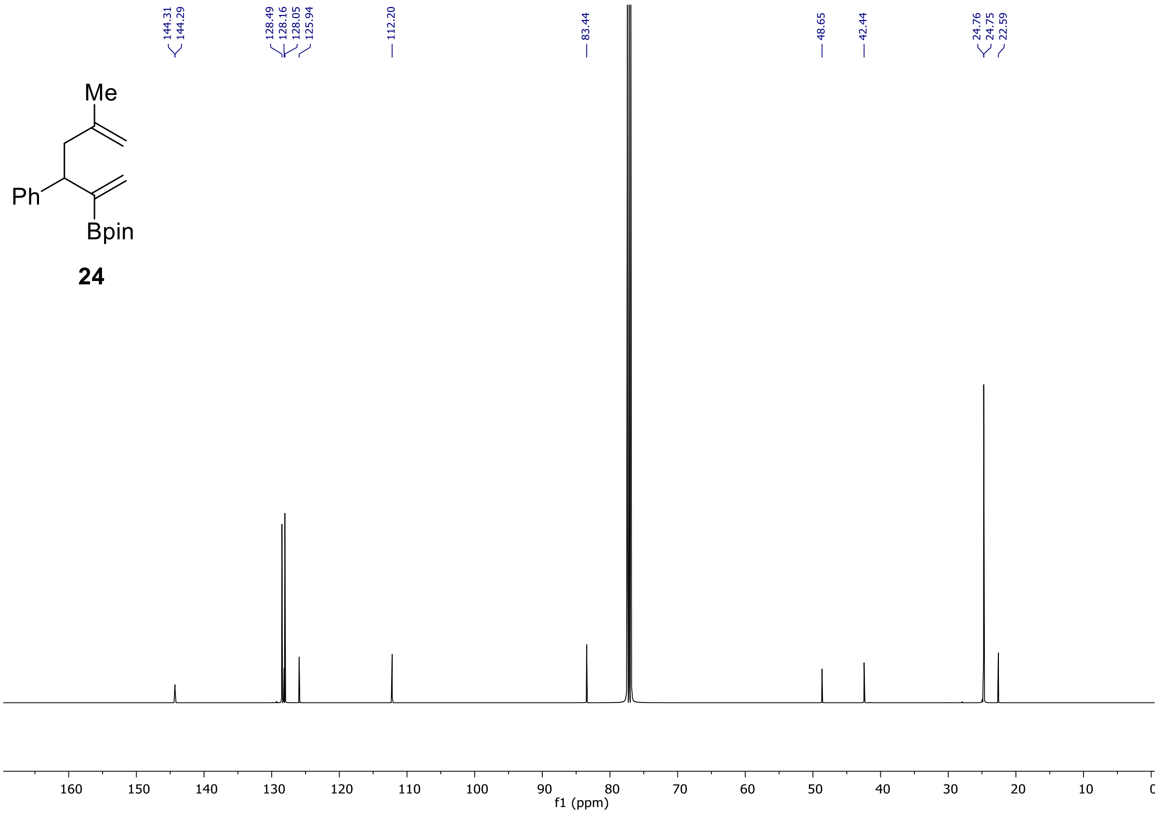

^{1}H NMR (300 MHz, $CDCl_3$)

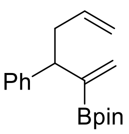

25

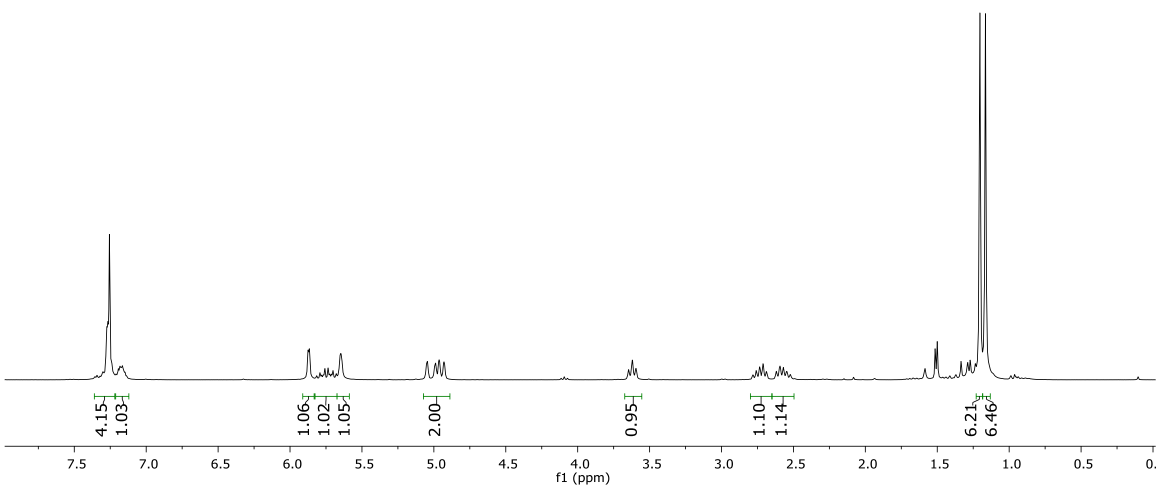

^{13}C NMR (75 MHz, $CDCl_3$)

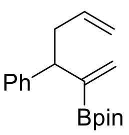

25

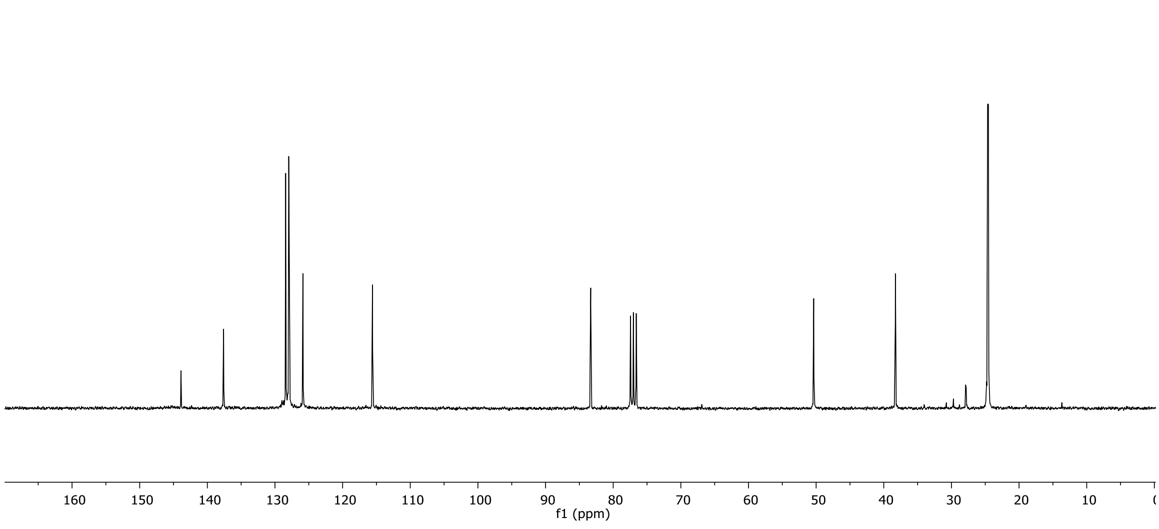

^{1}H NMR (500 MHz, $CDCl_3$)

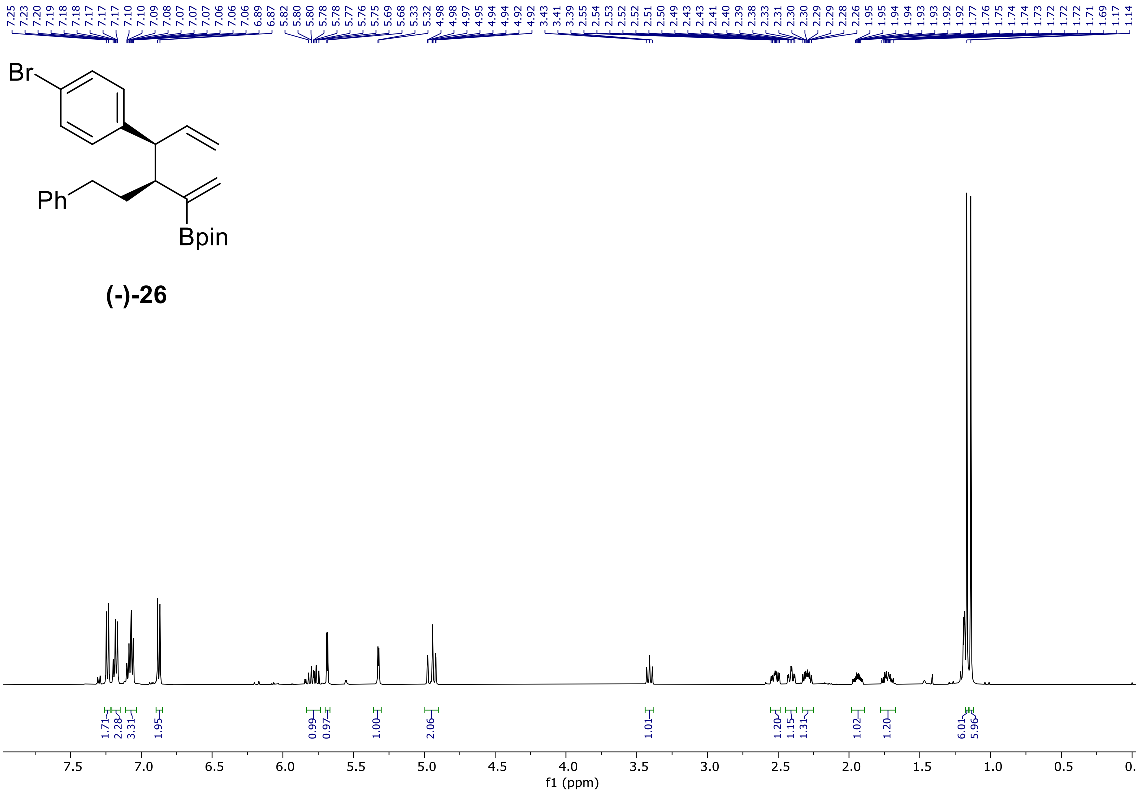

(-)-26

^{13}C NMR (126 MHz, $CDCl_3$)

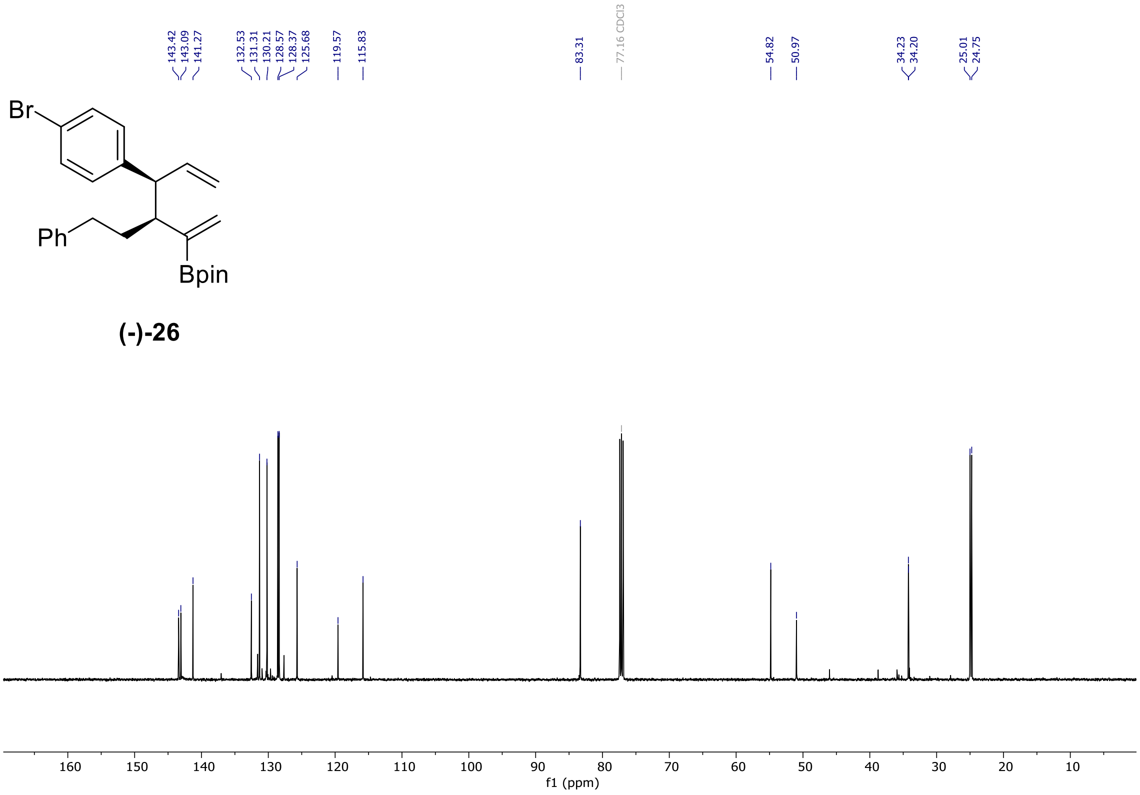

(-)-26

^{1}H NMR (500 MHz, $CDCl_3$)

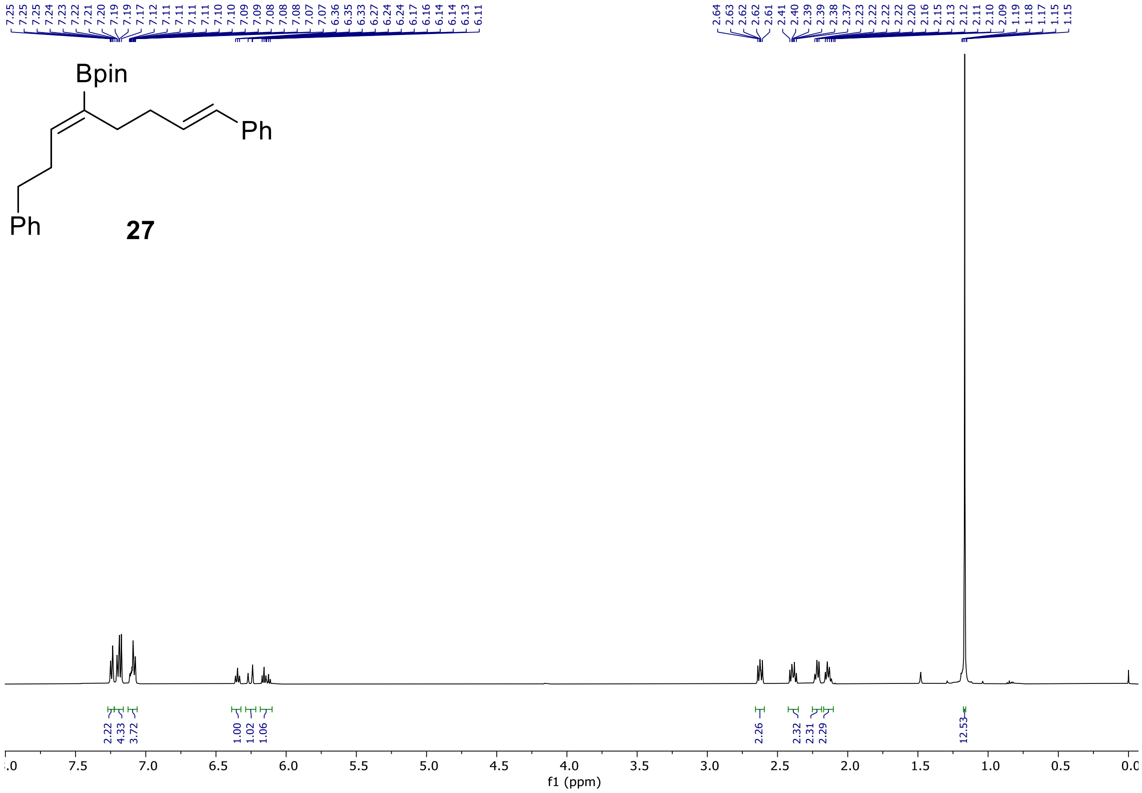

^{13}C NMR (126 MHz, $CDCl_3$)

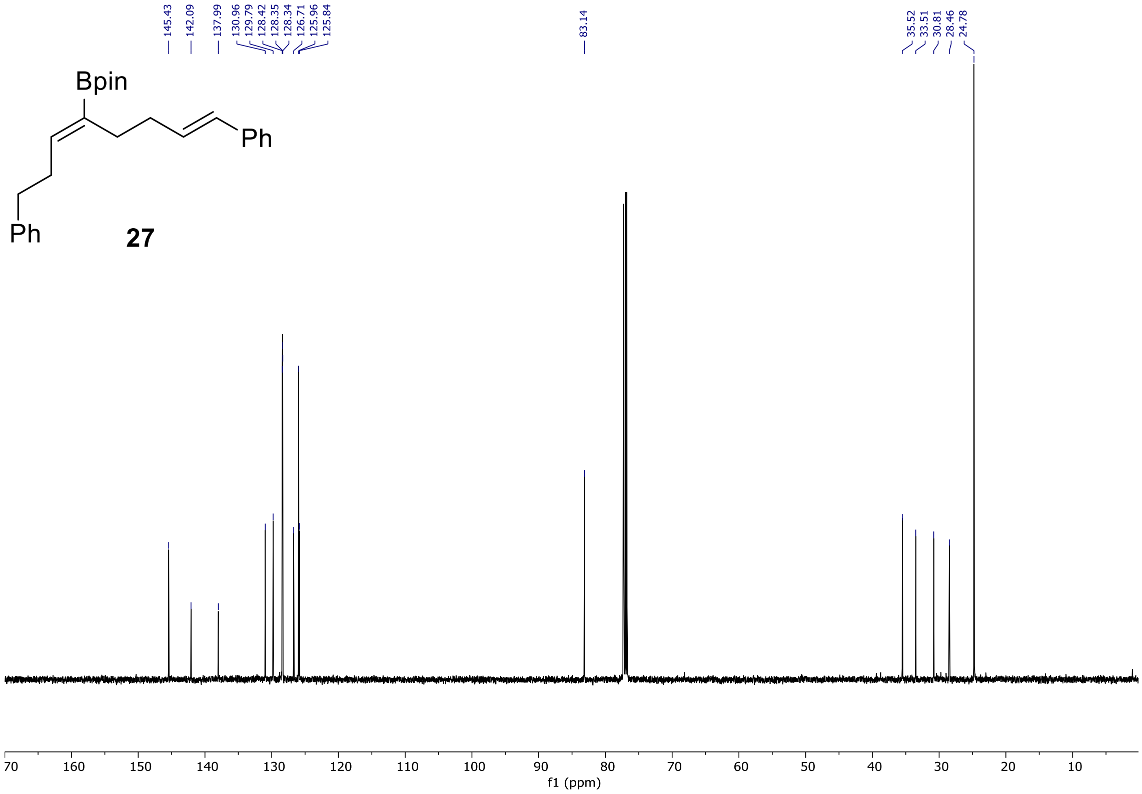

COSY

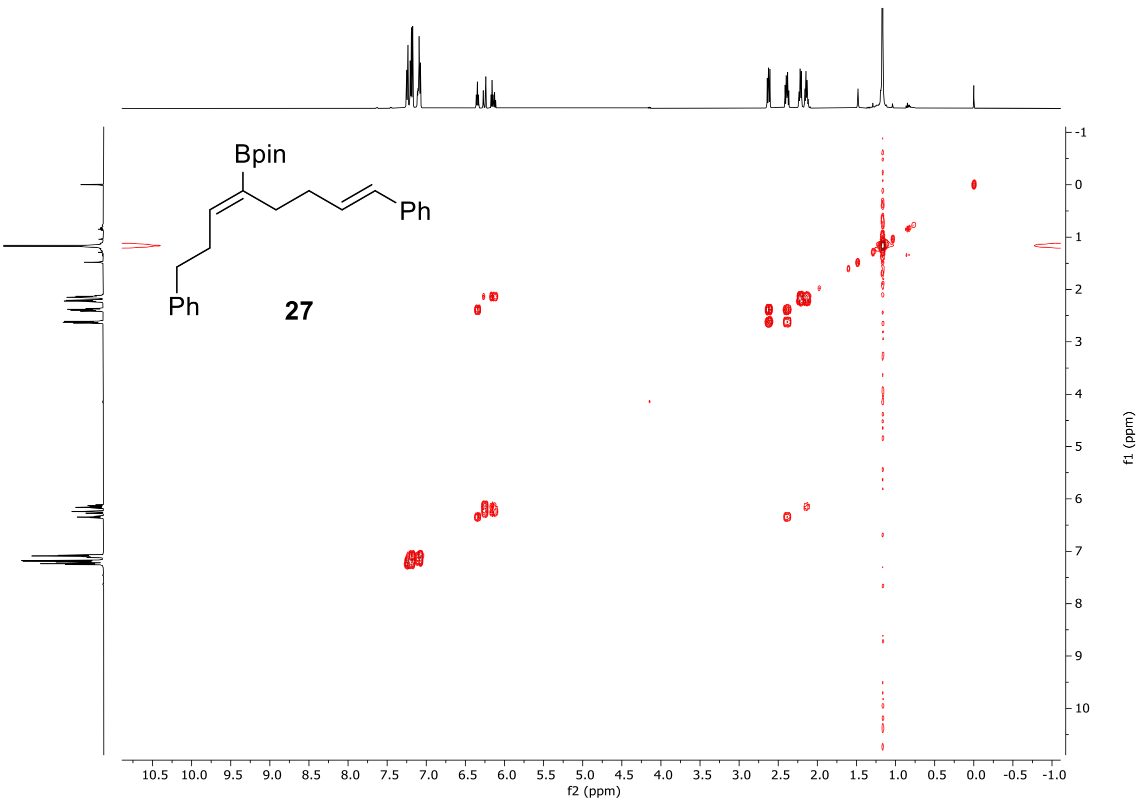

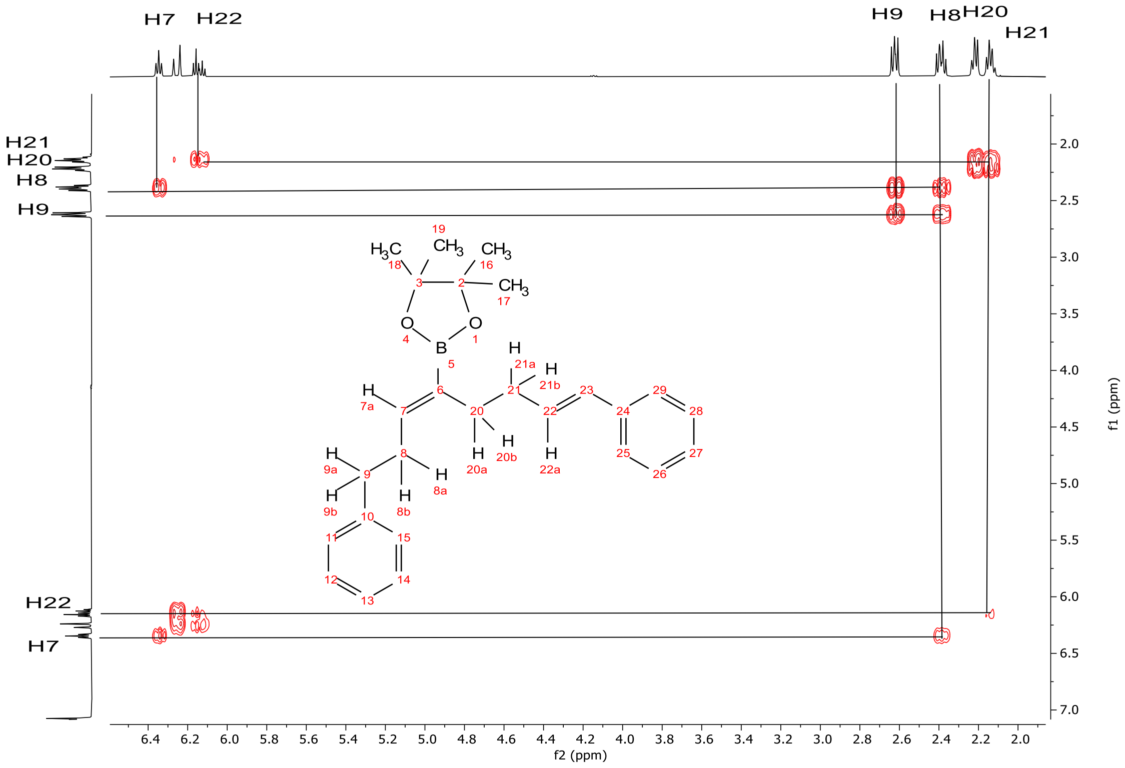

NOESY

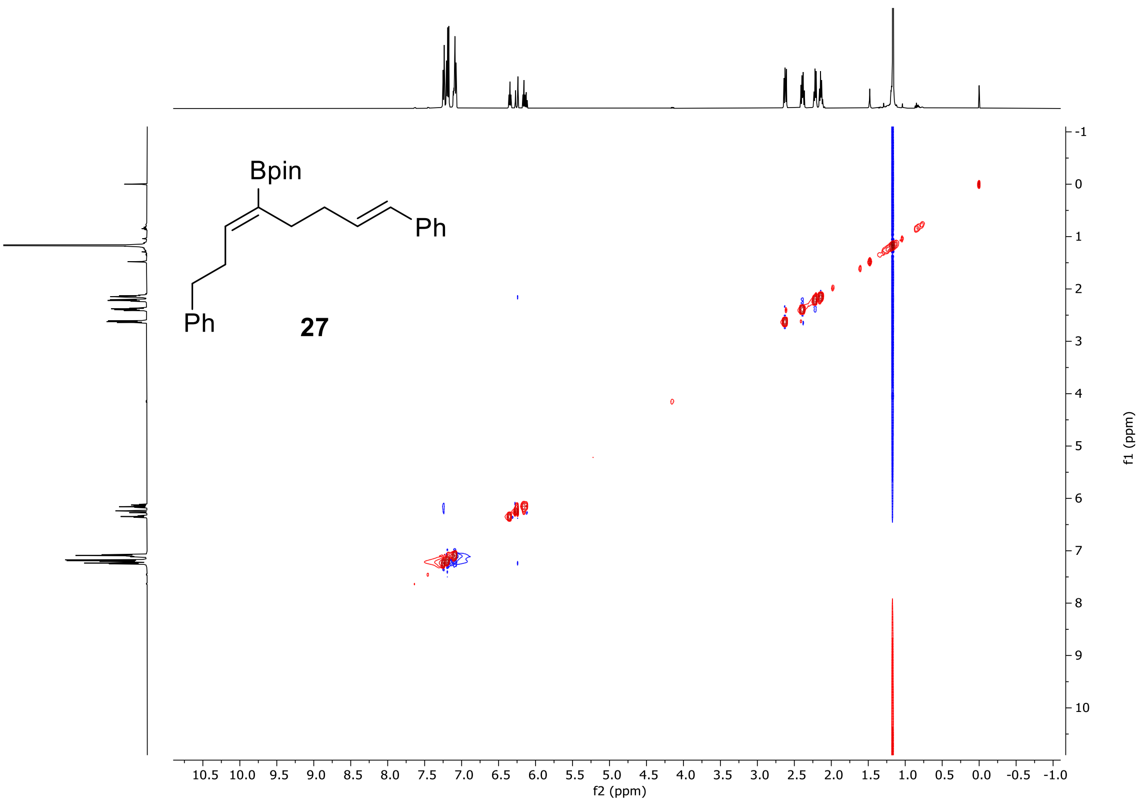

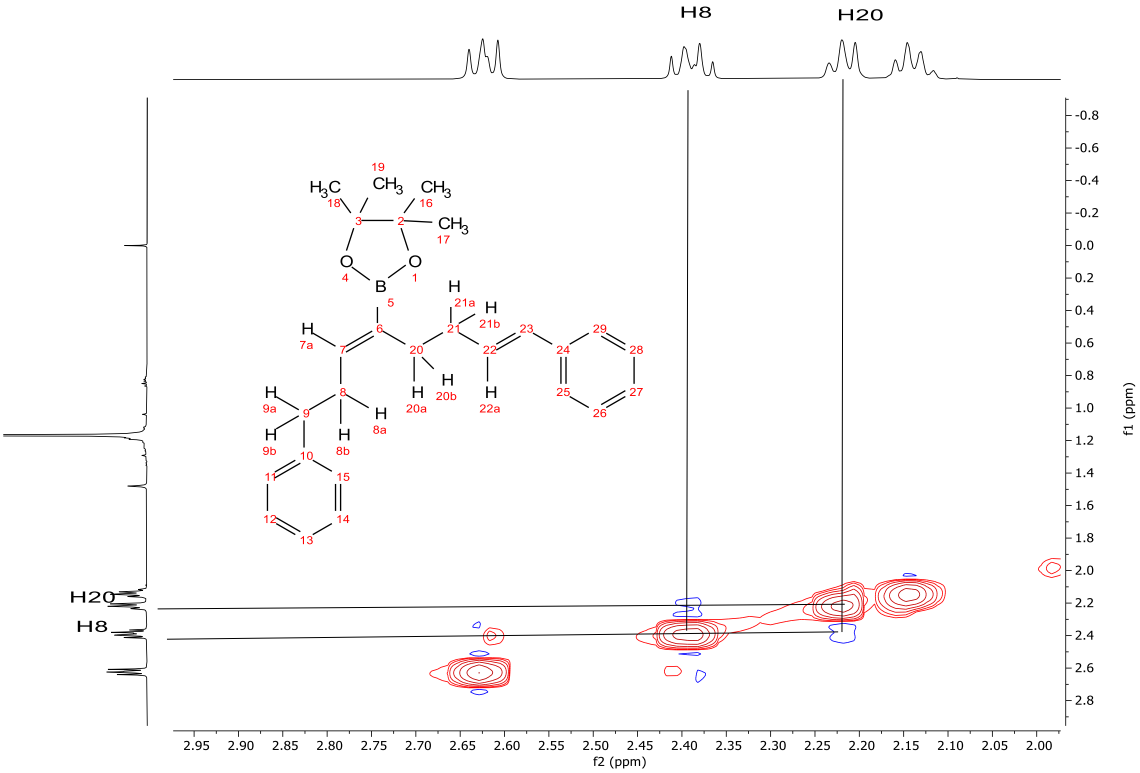

^{1}H NMR (300 MHz, $CDCl_3$)

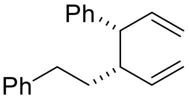

28

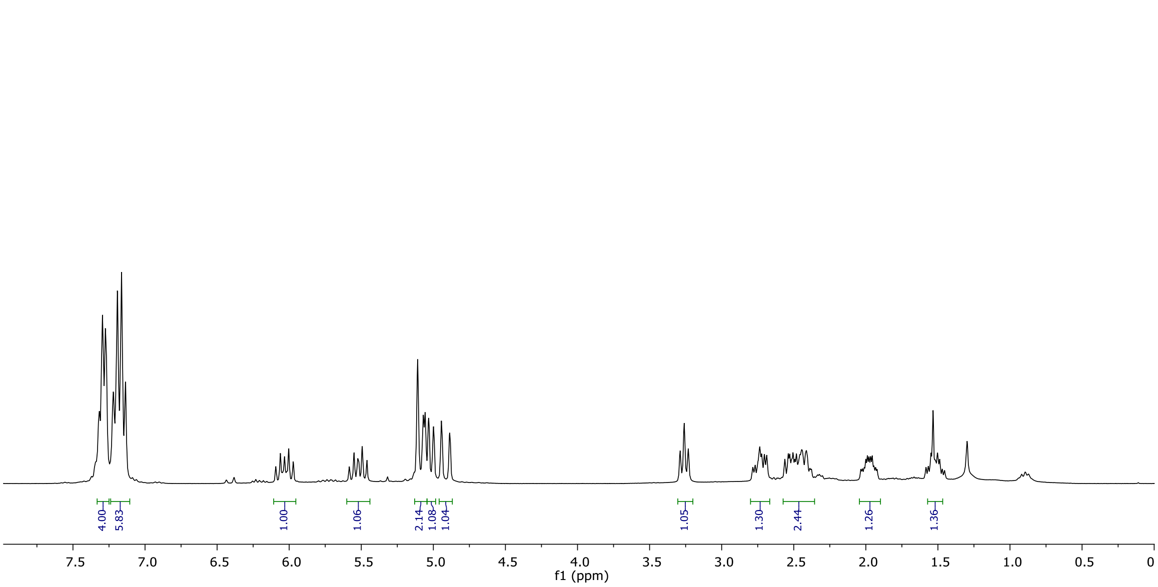

^{13}C NMR (126 MHz, $CDCl_3$)

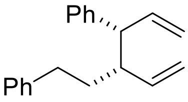

28

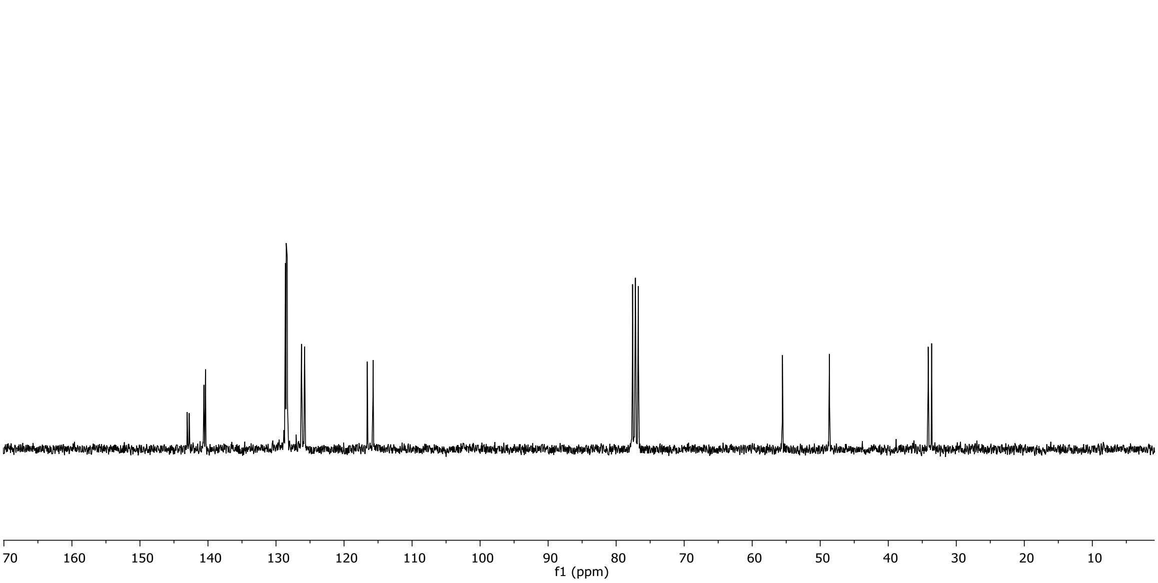

^{1}H NMR (300 MHz, $CDCl_3$)

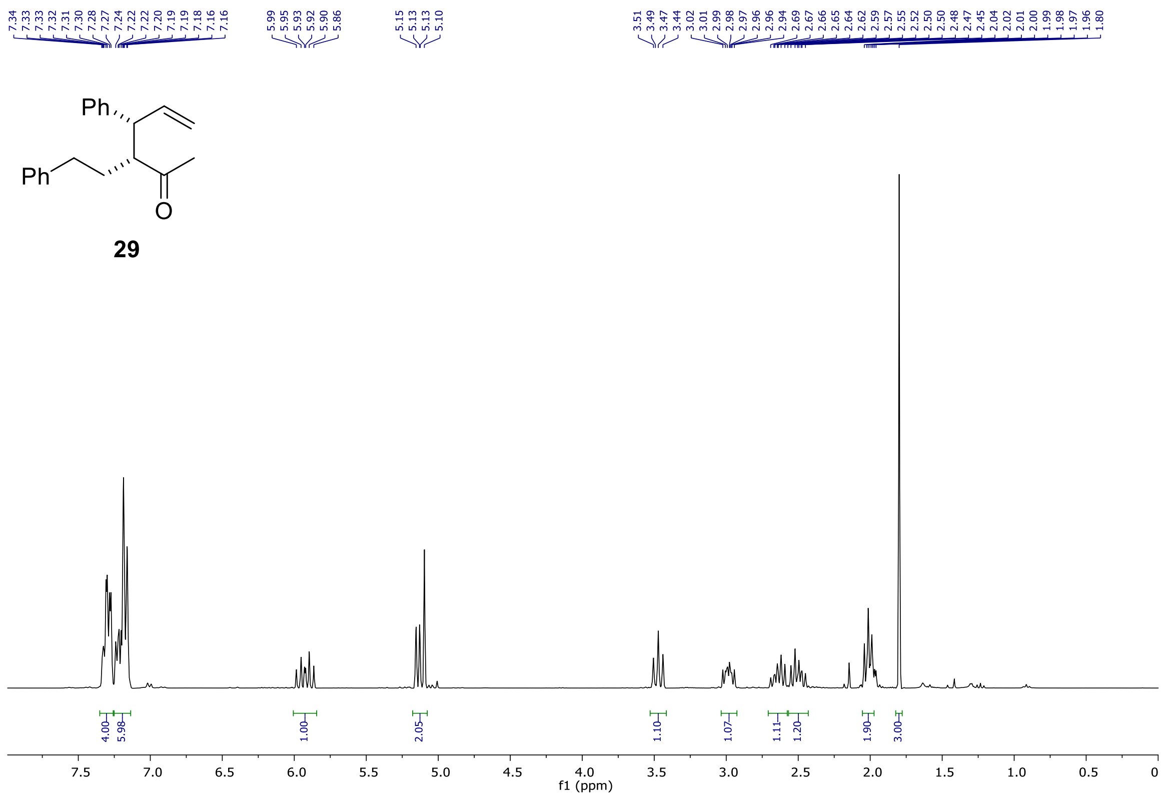

^{13}C NMR (75 MHz, $CDCl_3$)

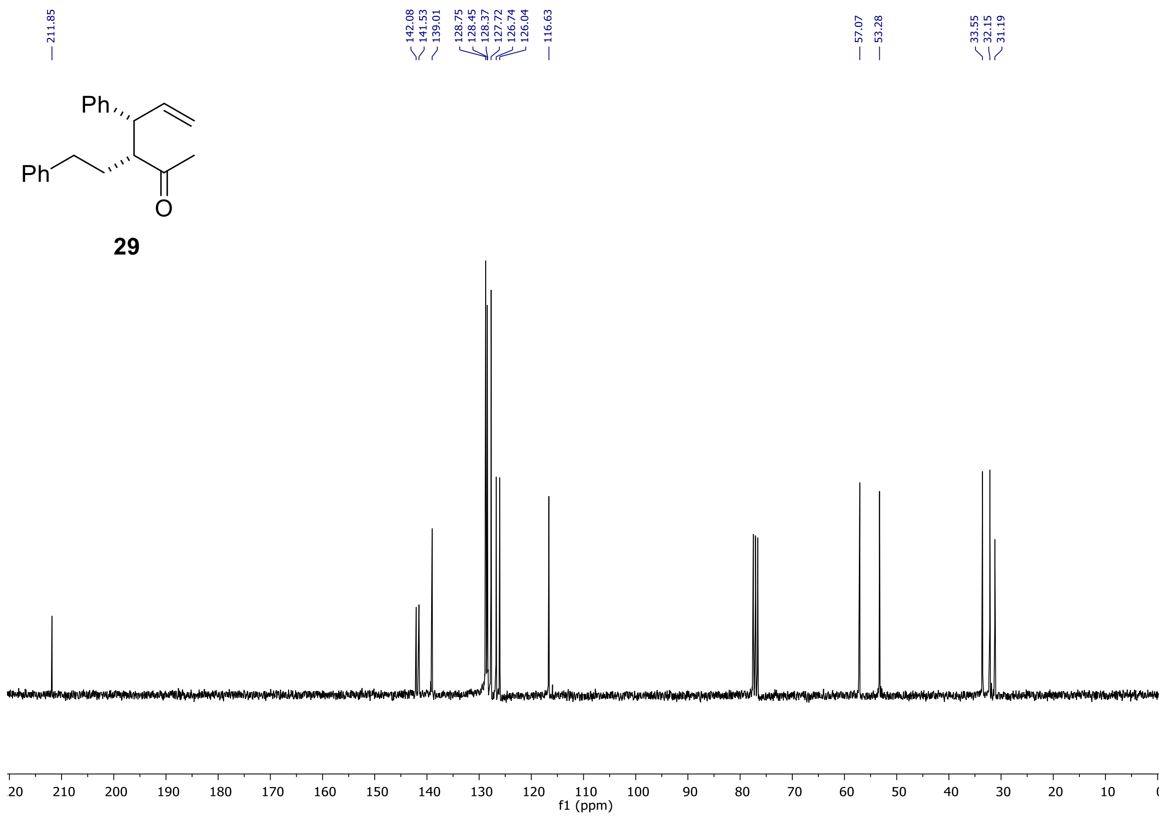

^{1}H NMR (300 MHz, $CDCl_3$)

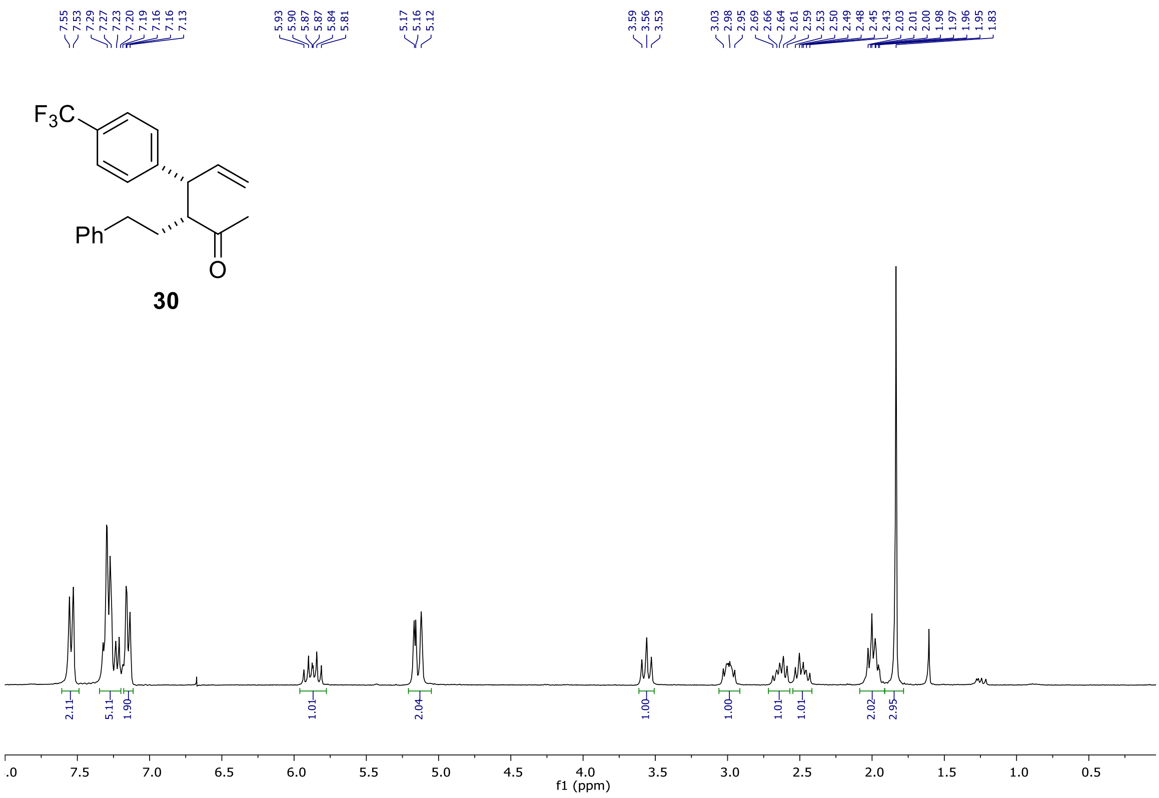

^{13}C NMR (75 MHz, $CDCl_3$)

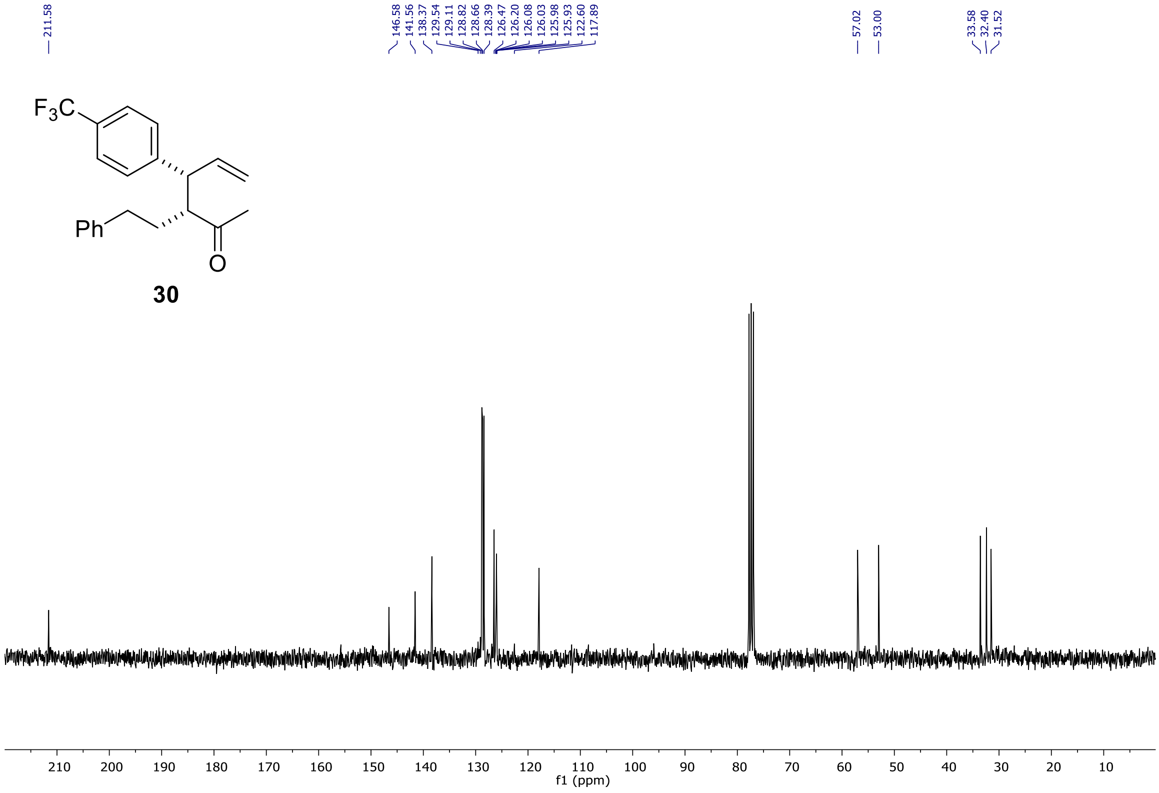

^{1}H NMR (300 MHz, $CDCl_3$)

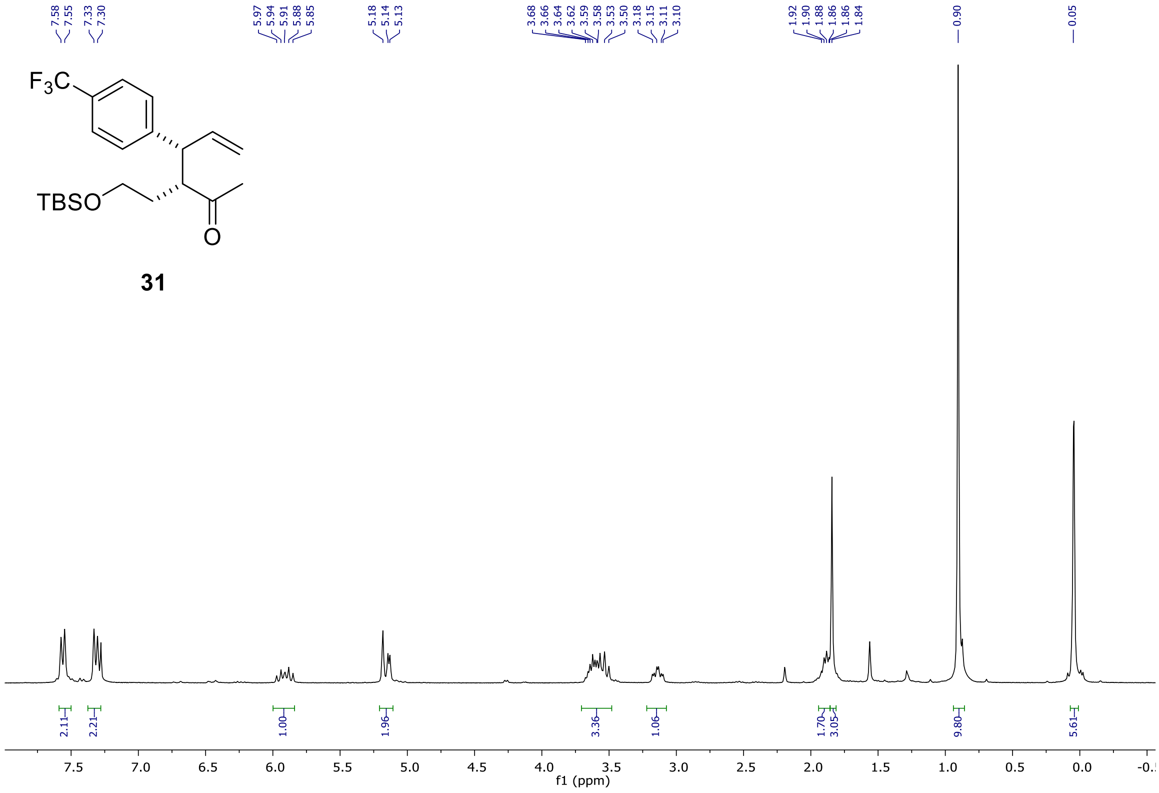

^{13}C NMR (75 MHz, $CDCl_3$)

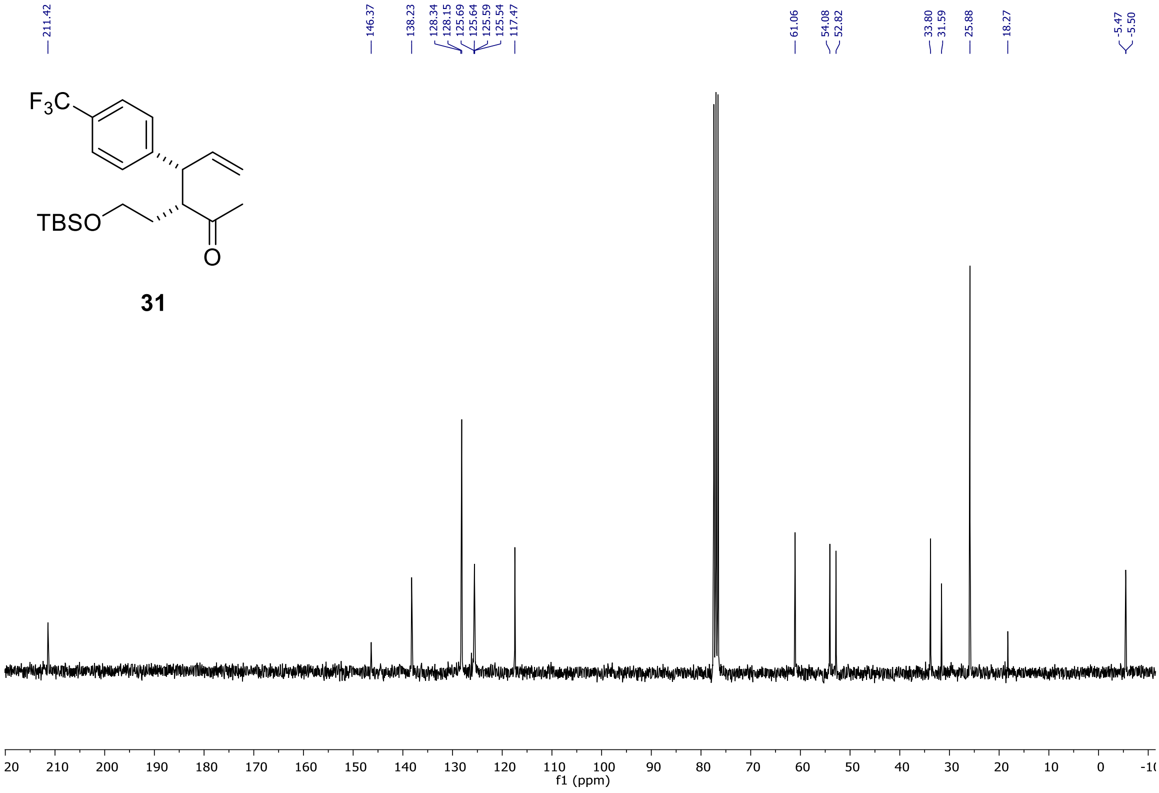

^{1}H NMR (300 MHz, $CDCl_3$)

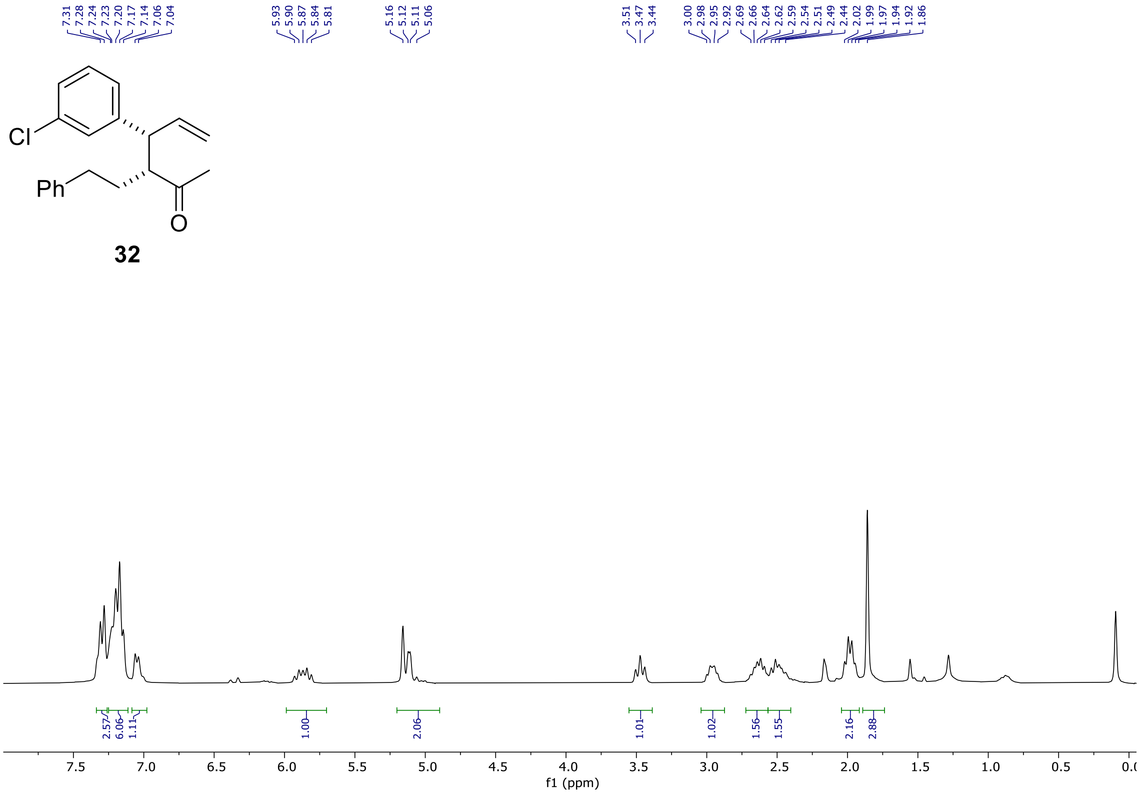

^{13}C NMR (300 MHz, $CDCl_3$)

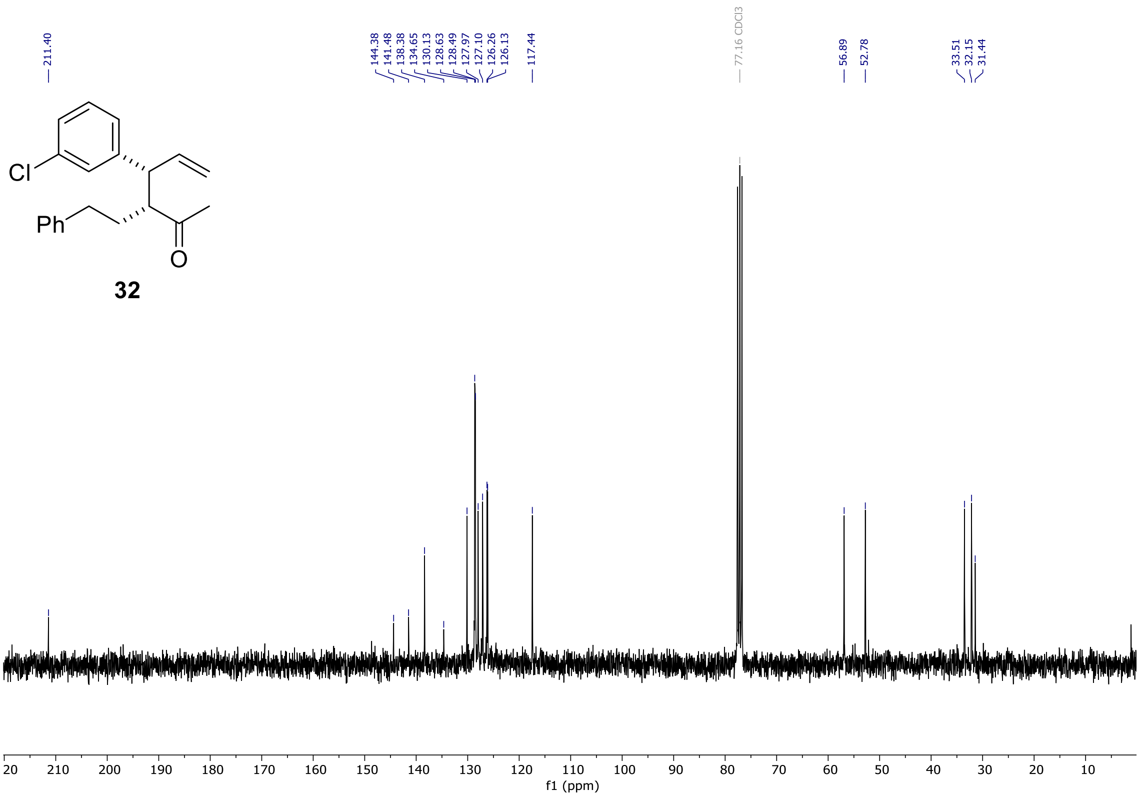

^{1}H NMR (500 MHz, $CDCl_3$)

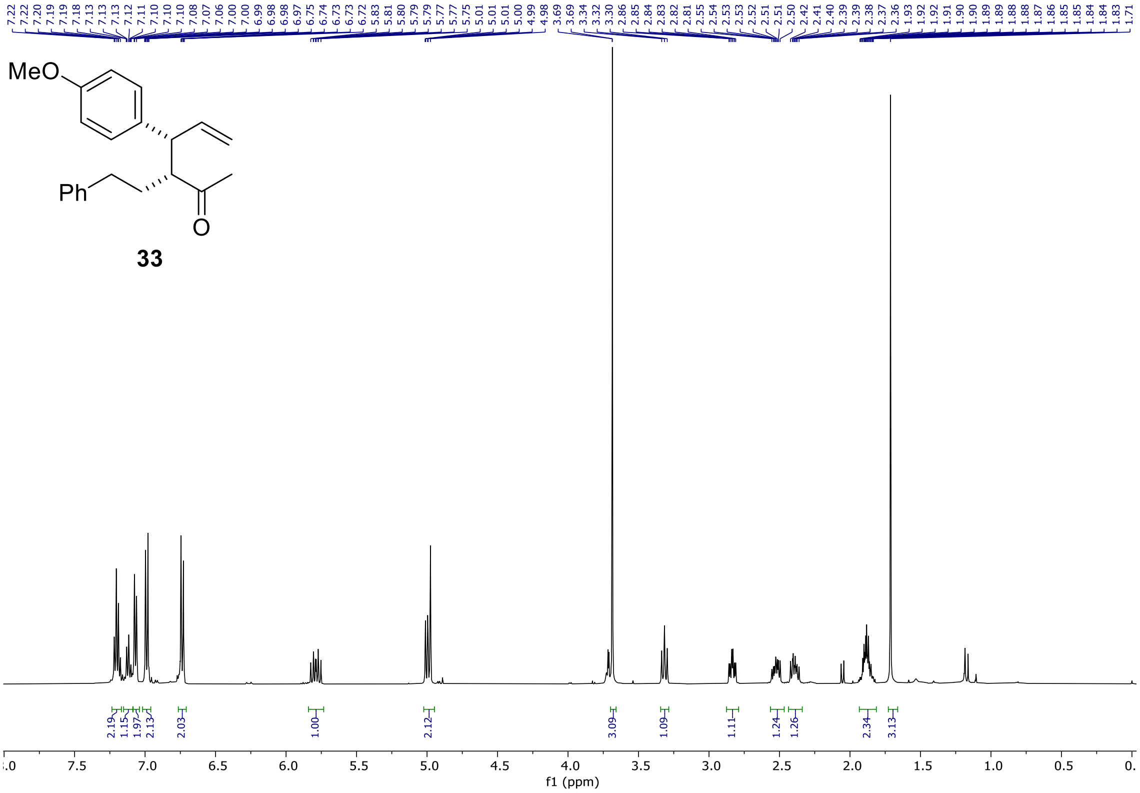

33

^{13}C NMR (126 MHz, $CDCl_3$)

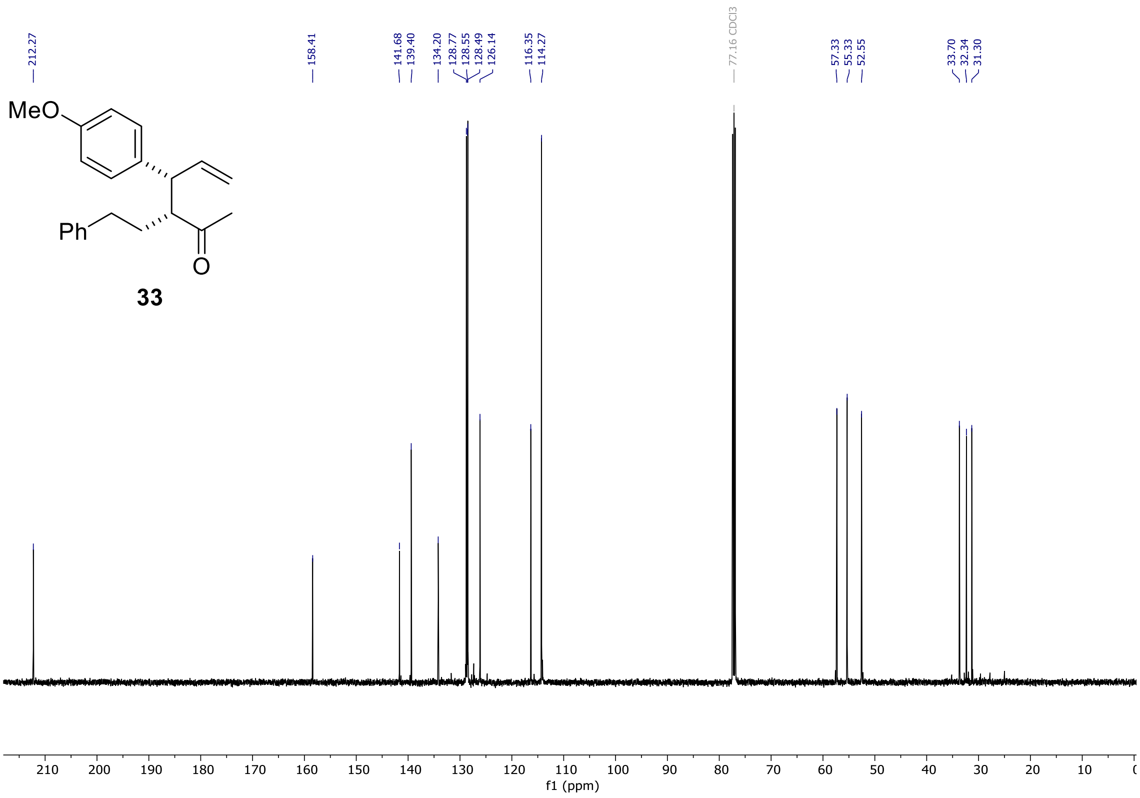

33

^{1}H NMR (300 MHz, $CDCl_3$)

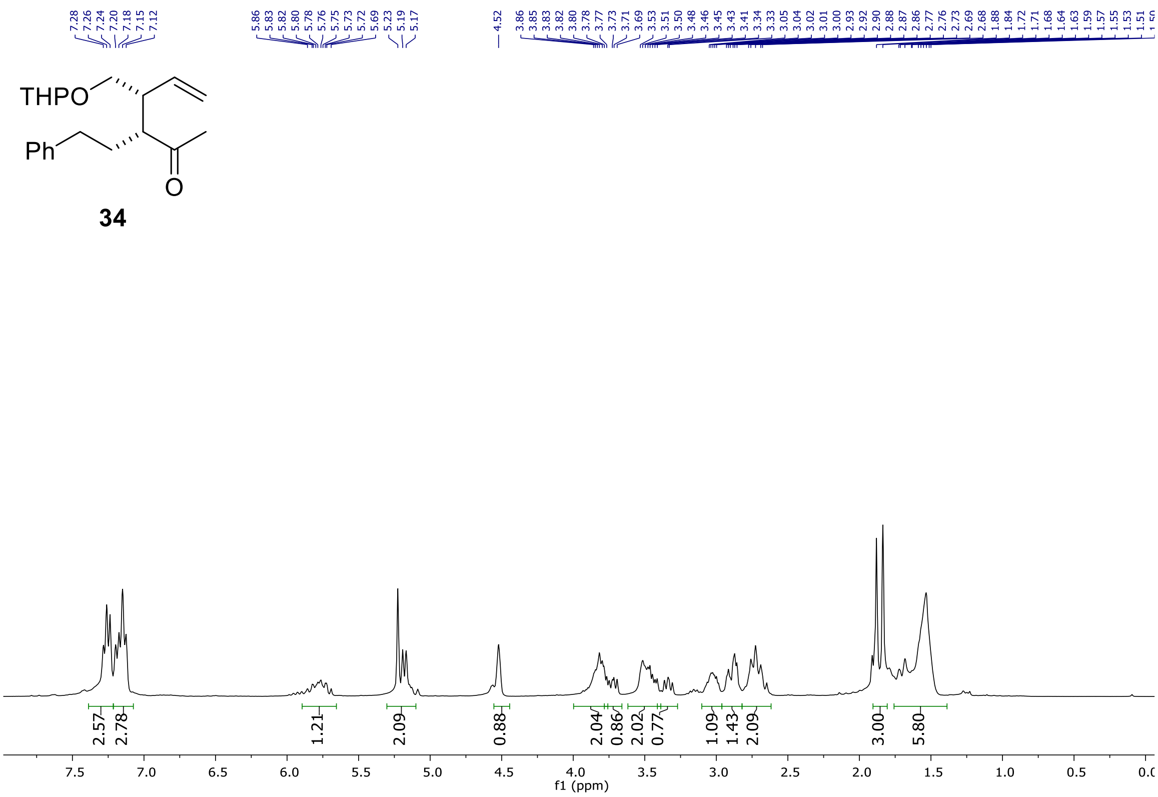

^{13}C NMR (75 MHz, $CDCl_3$)

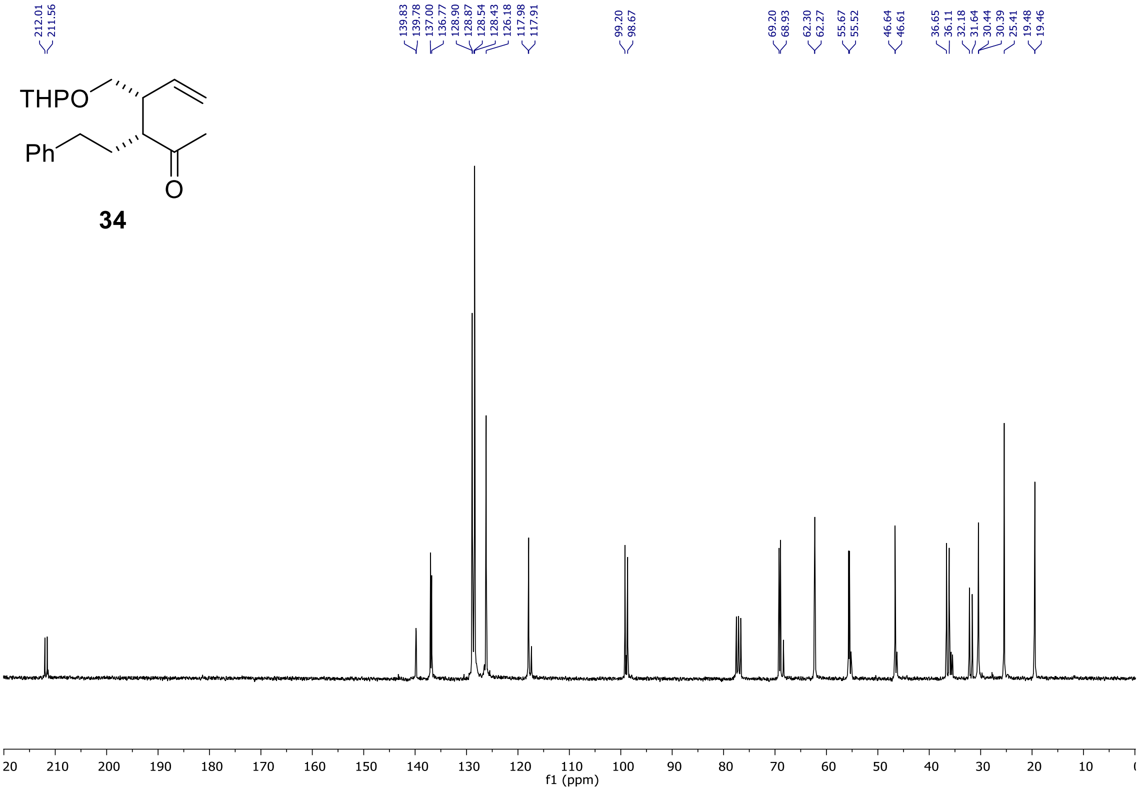

^{1}H NMR (300 MHz, $CDCl_3$)

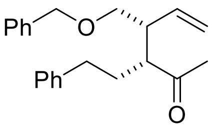

35

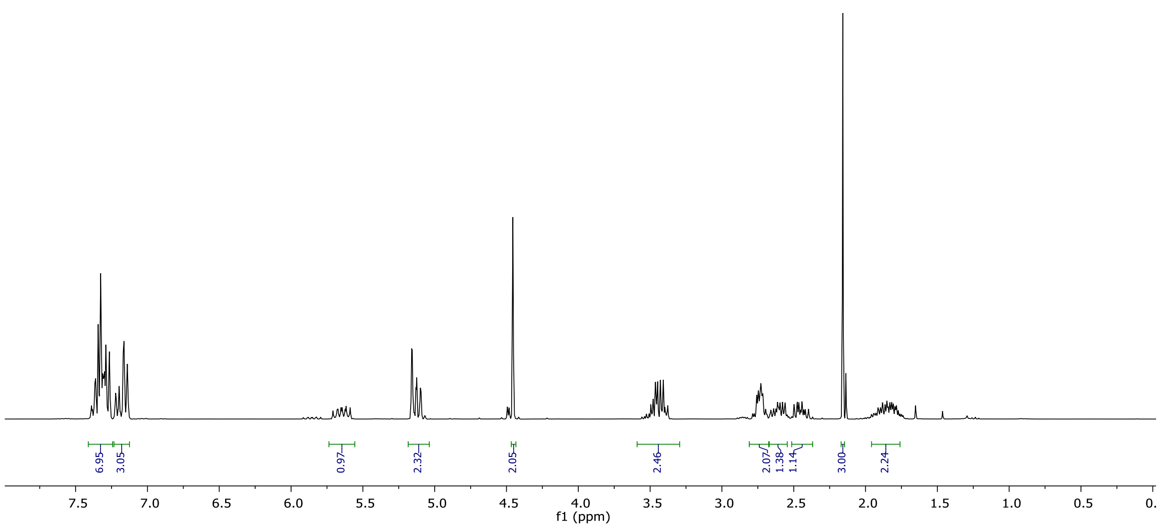

^{13}C NMR (75 MHz, $CDCl_3$)

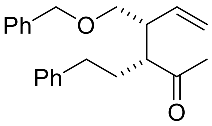

35

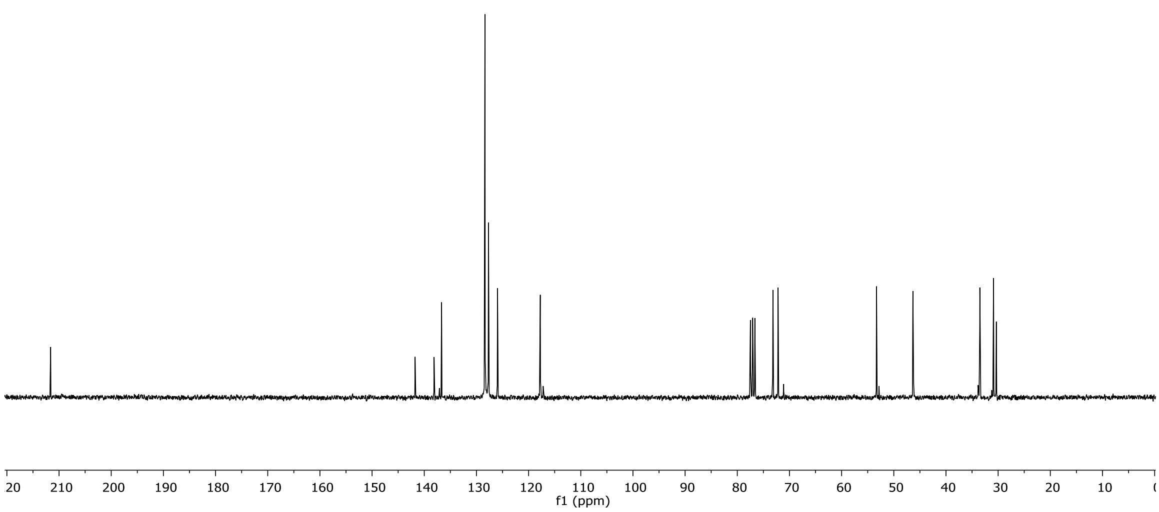

^{1}H NMR (500 MHz, $CDCl_3$)

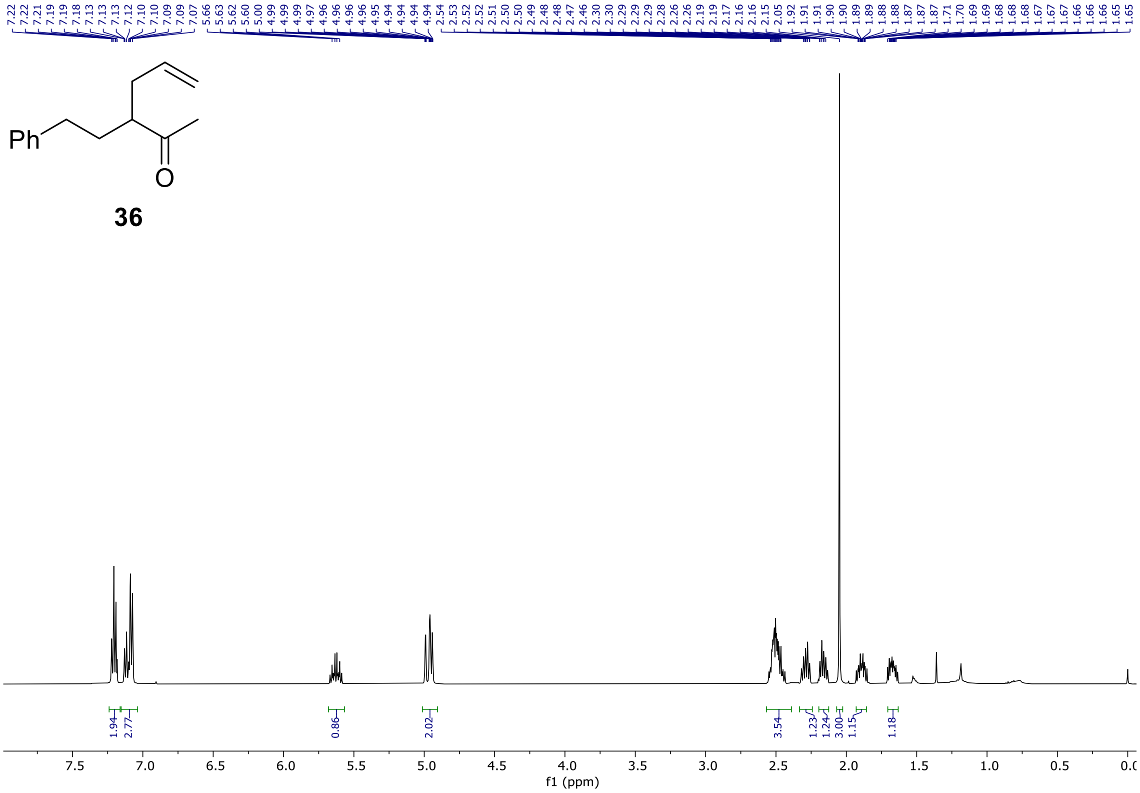

^{13}C NMR (126 MHz, $CDCl_3$)

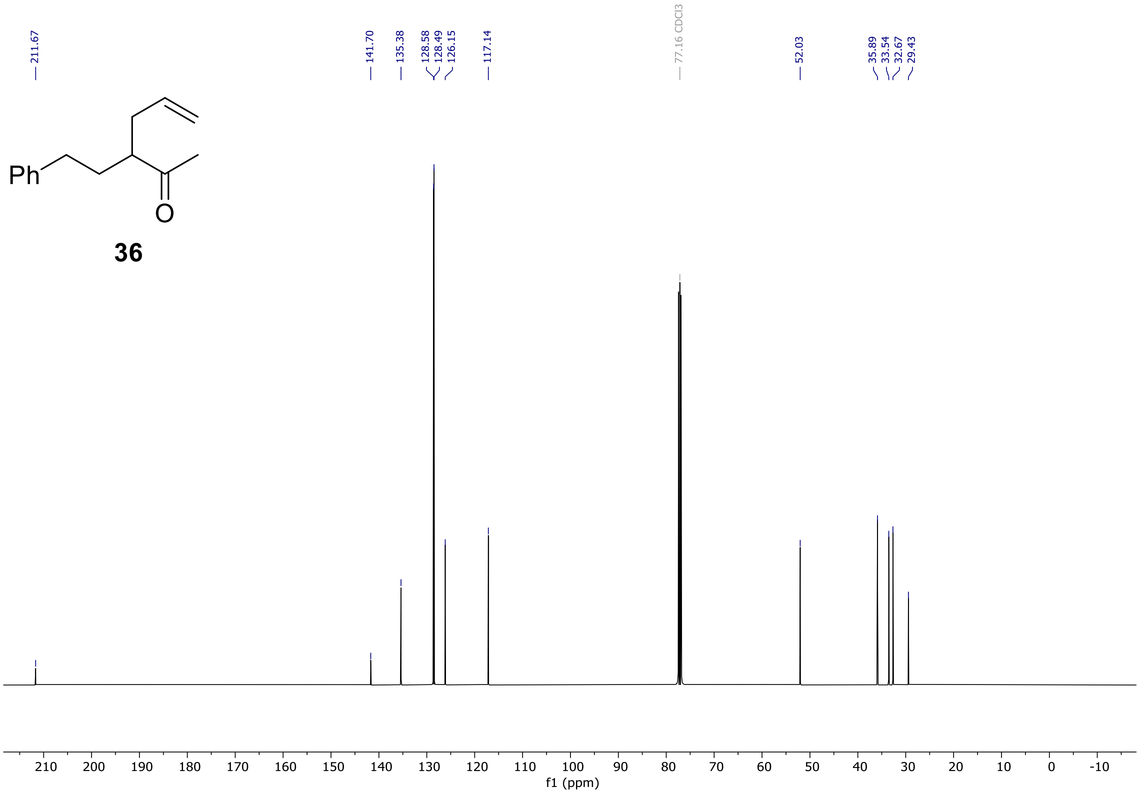

^{1}H NMR (500 MHz, $CDCl_3$)

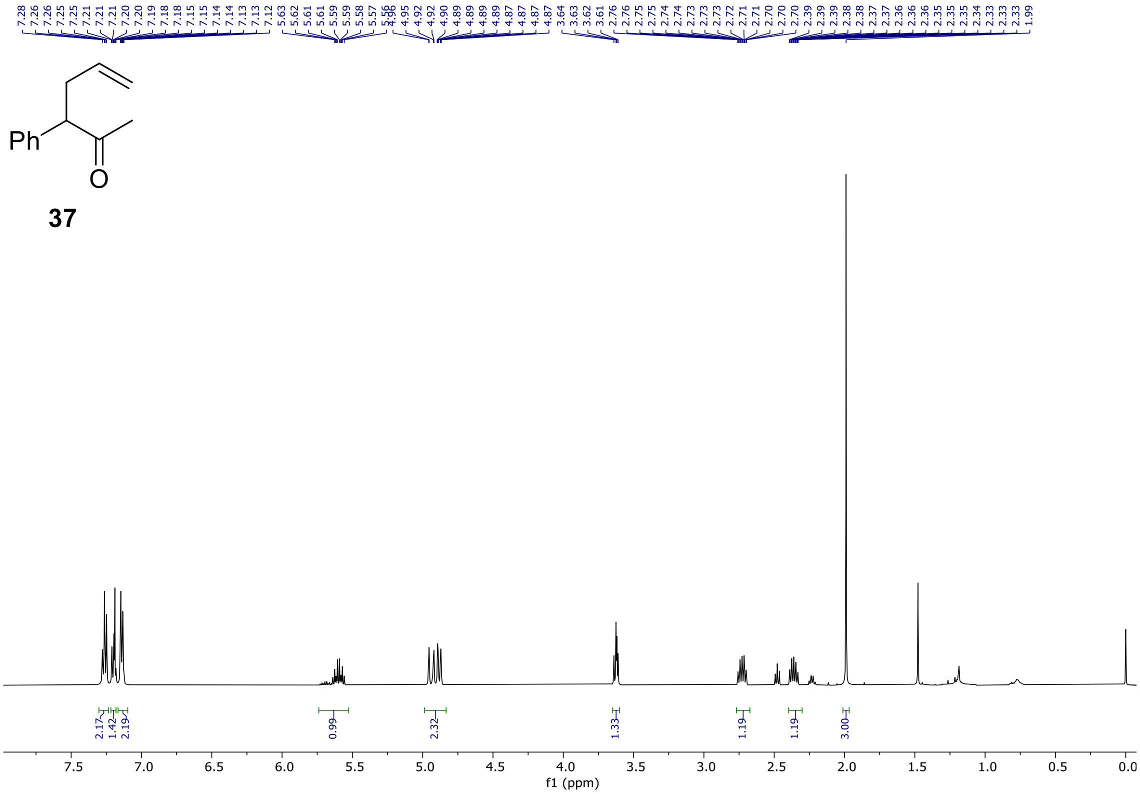

^{13}C NMR (126 MHz, $CDCl_3$)

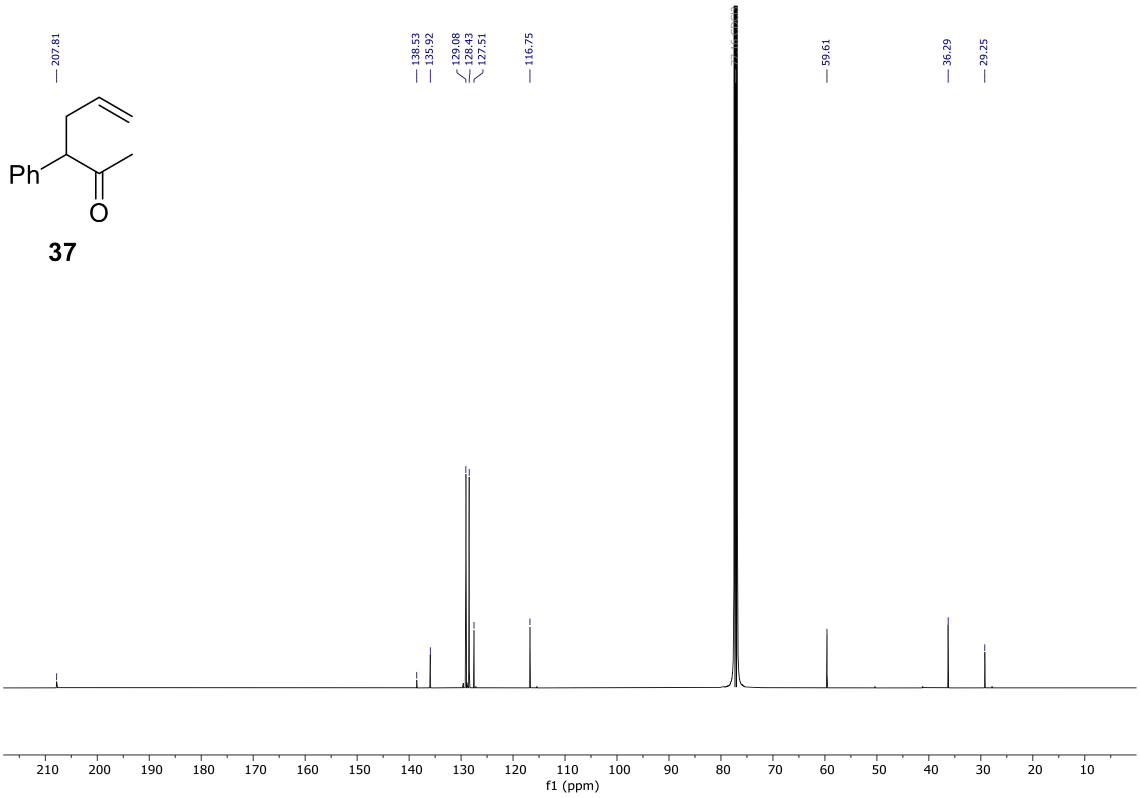

^{1}H NMR (500 MHz, $CDCl_3$)

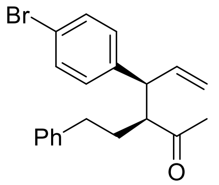

(-)-38

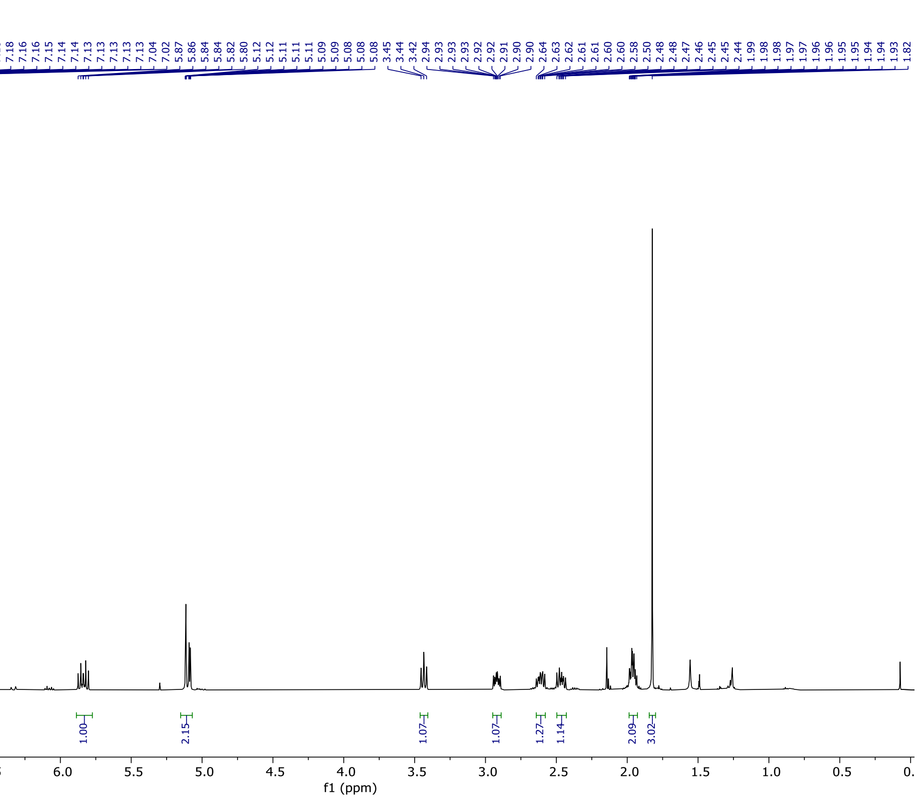

^{13}C NMR (126 MHz, $CDCl_3$)

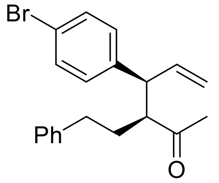

(-)-38

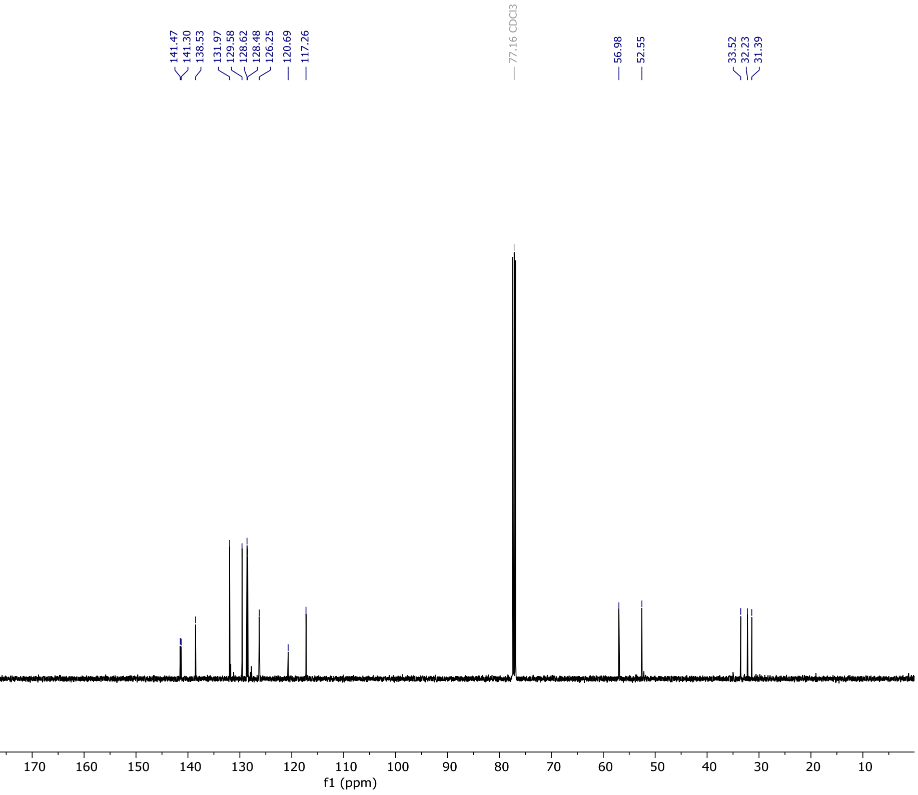

^{1}H NMR (500 MHz, $CDCl_3$)

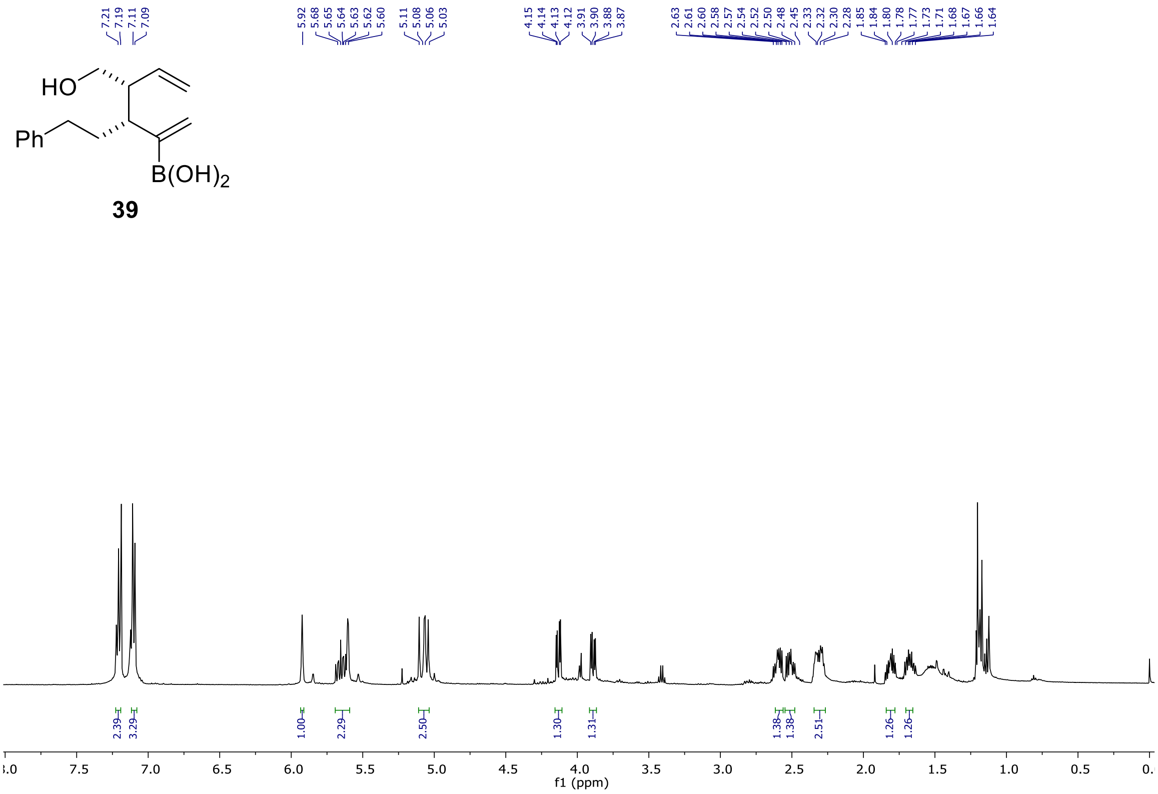

39

^{13}C NMR (126 MHz, $CDCl_3$)

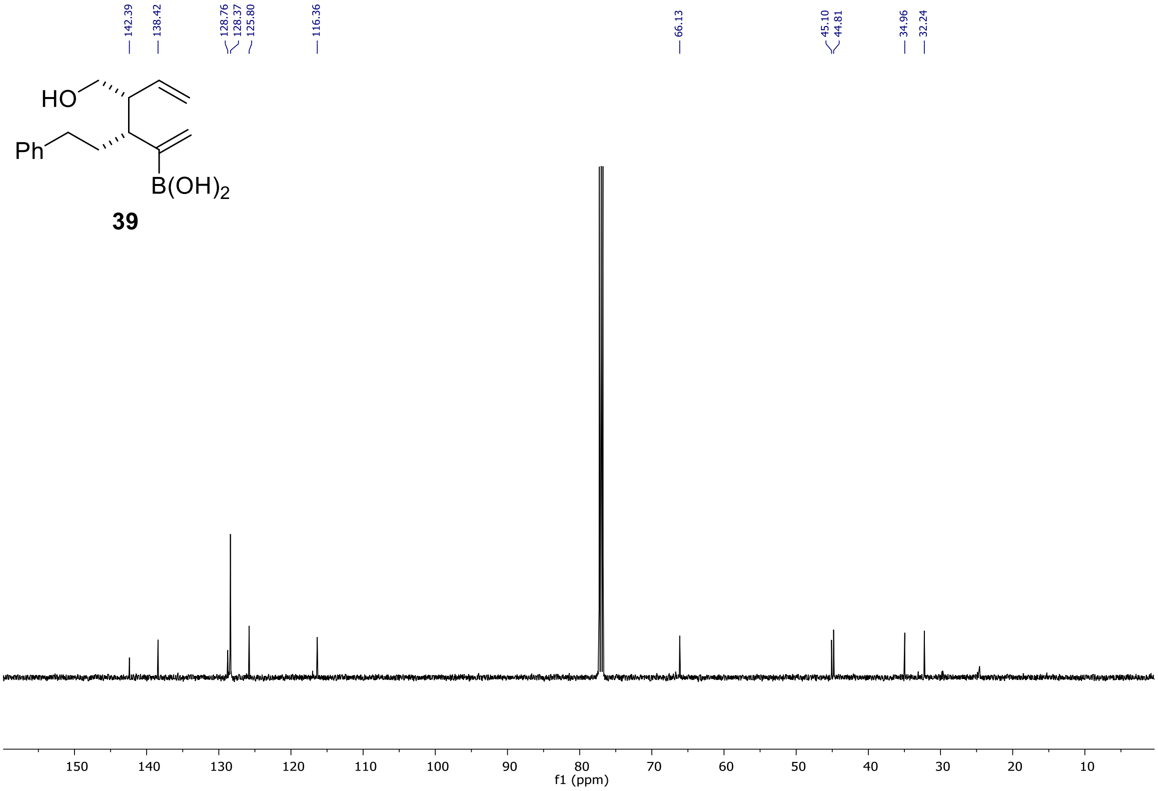

39

^{1}H NMR (500 MHz, $CDCl_3$)

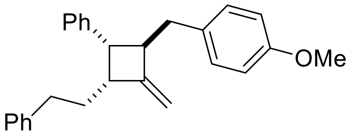

40

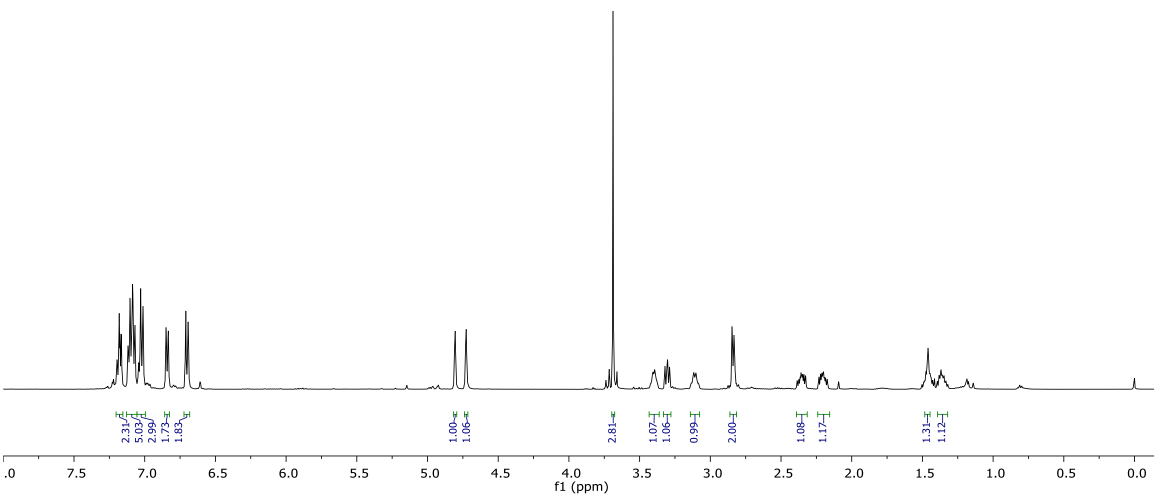

^{13}C NMR (126 MHz, $CDCl_3$)

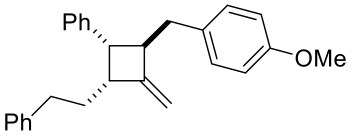

40

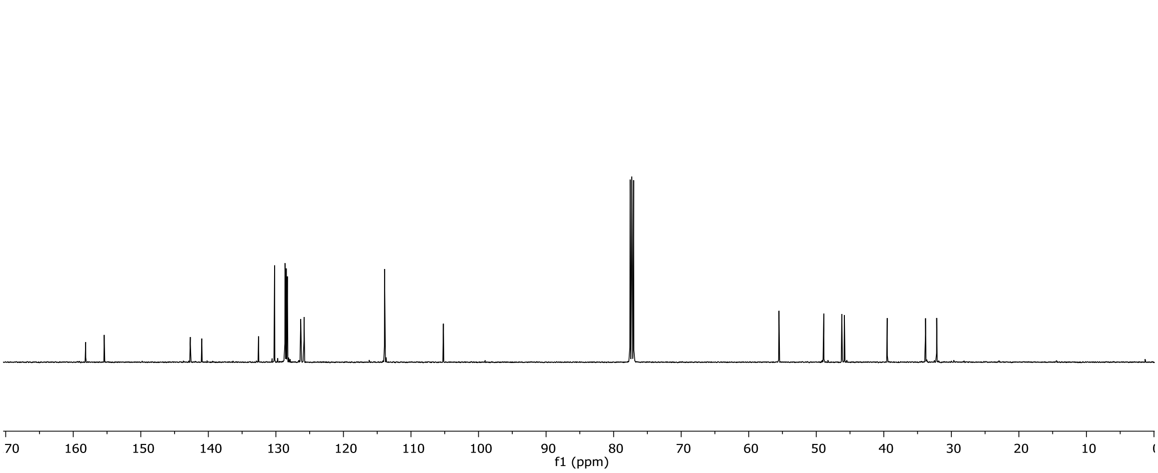

COSY

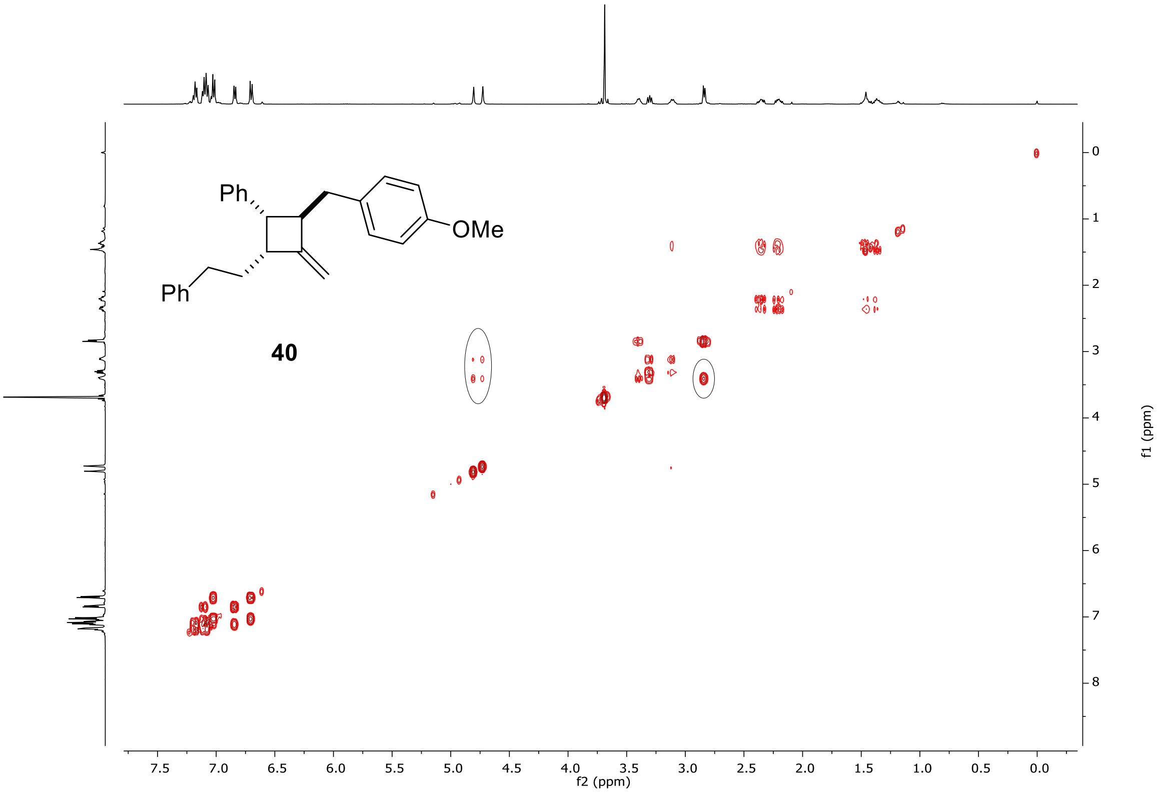
Ph
Ph
OMe
40
7.5
7.0
6.5
6.0
5.5
5.0
4.5
4.0
3.5
3.0
2.5
2.0
1.5
1.0
0.5
0.0
f2 (ppm)
f1 (ppm)

NOESY

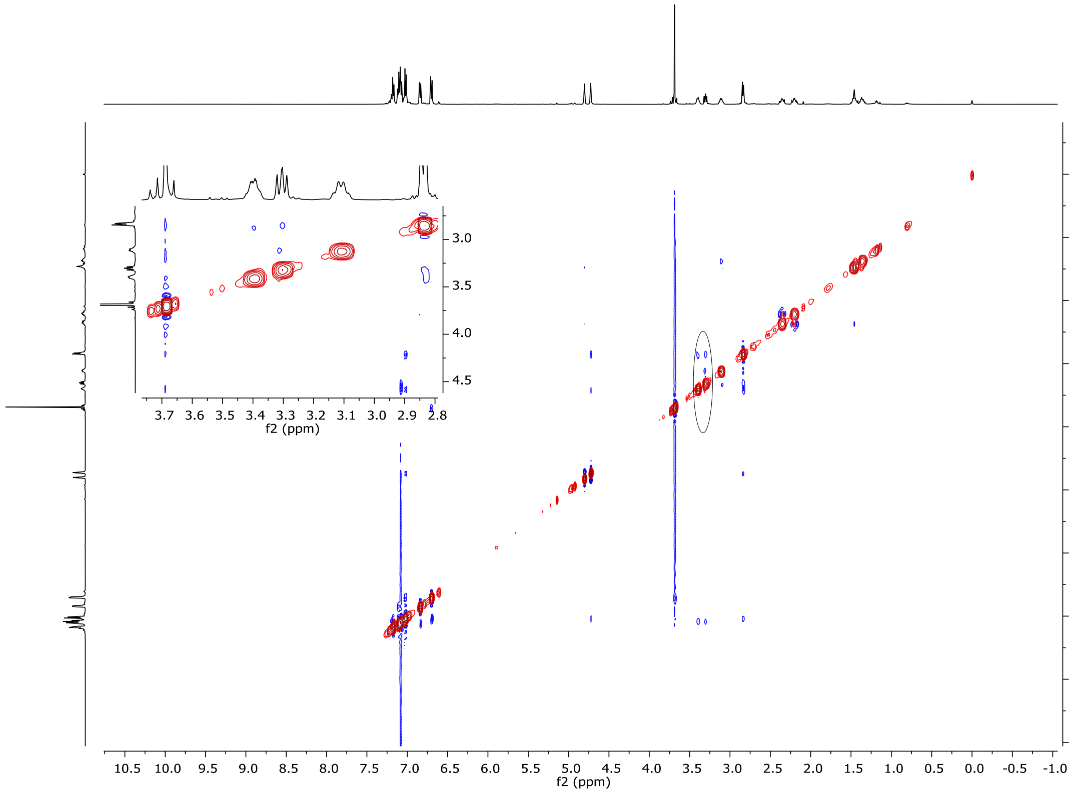
3.7 3.6 3.5 3.4 3.3 3.2 3.1 3.0 2.9 2.8
f2 (ppm)
10.5 10.0 9.5 9.0 8.5 8.0 7.5 7.0 6.5 6.0 5.5 5.0 4.5 4.0 3.5 3.0 2.5 2.0 1.5 1.0 0.5 0.0 -0.5 -1.0
f2 (ppm)

HMBC

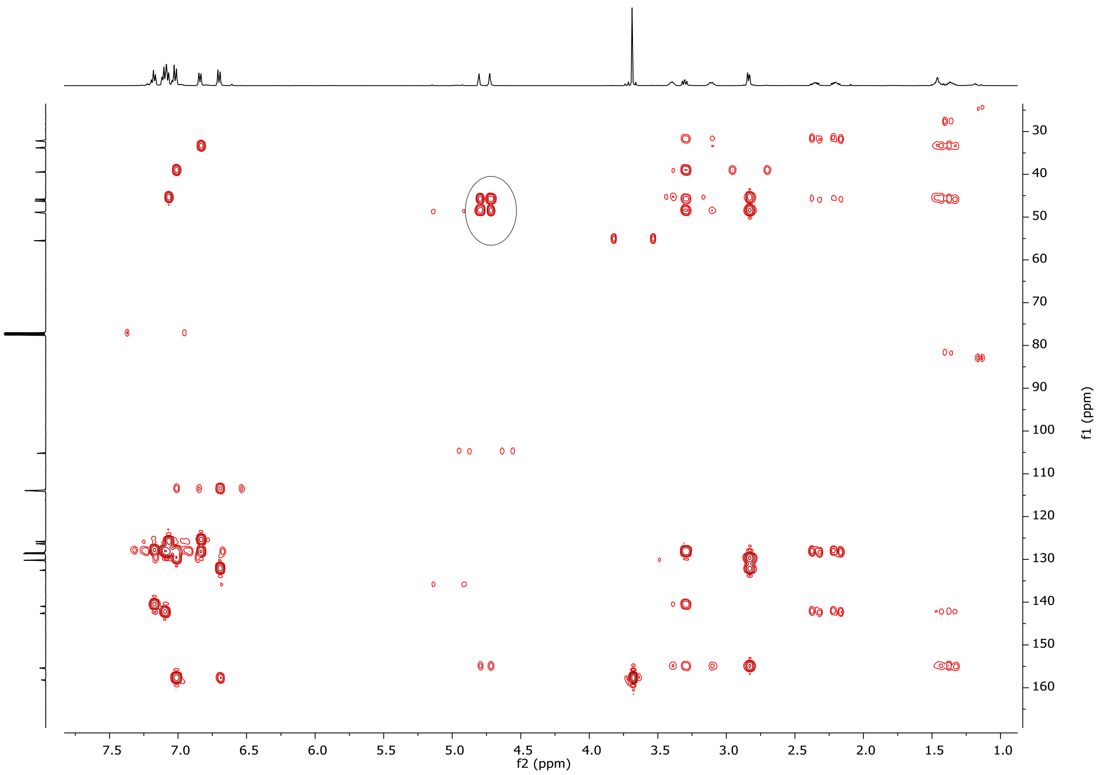

HSQC

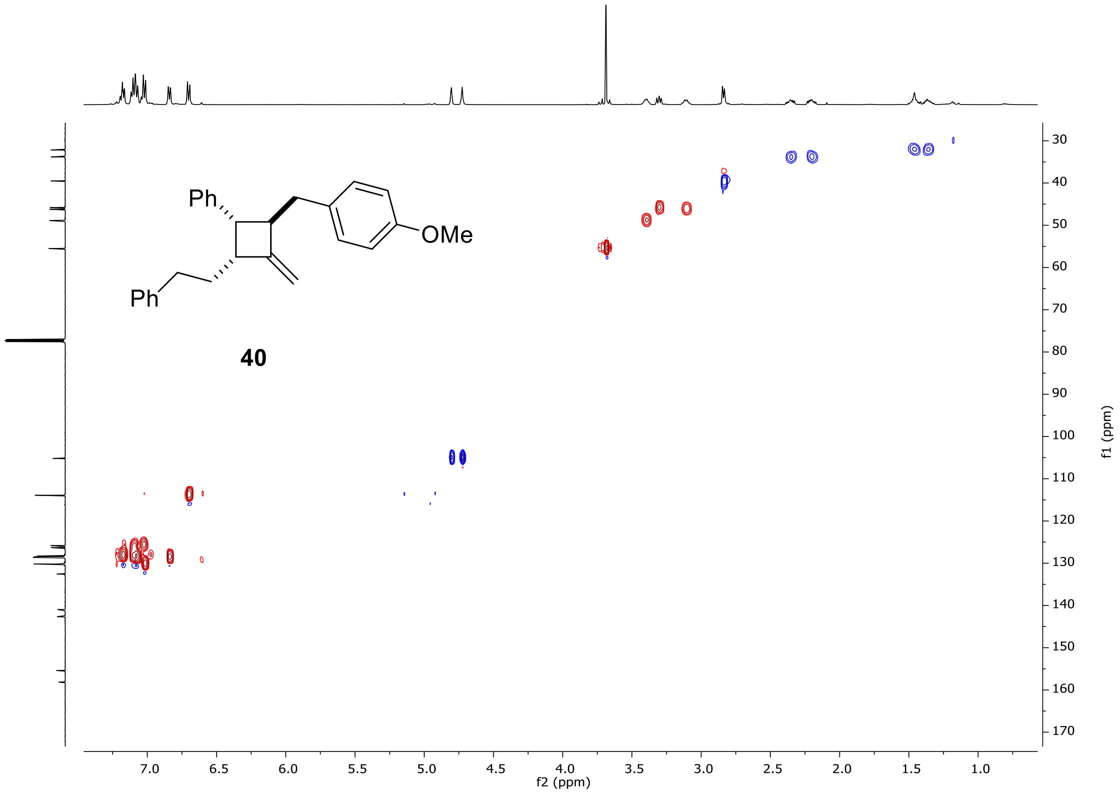

^{1}H NMR (500 MHz, $CDCl_3$)

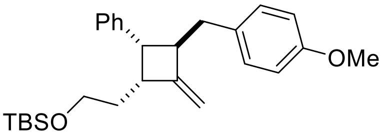

41

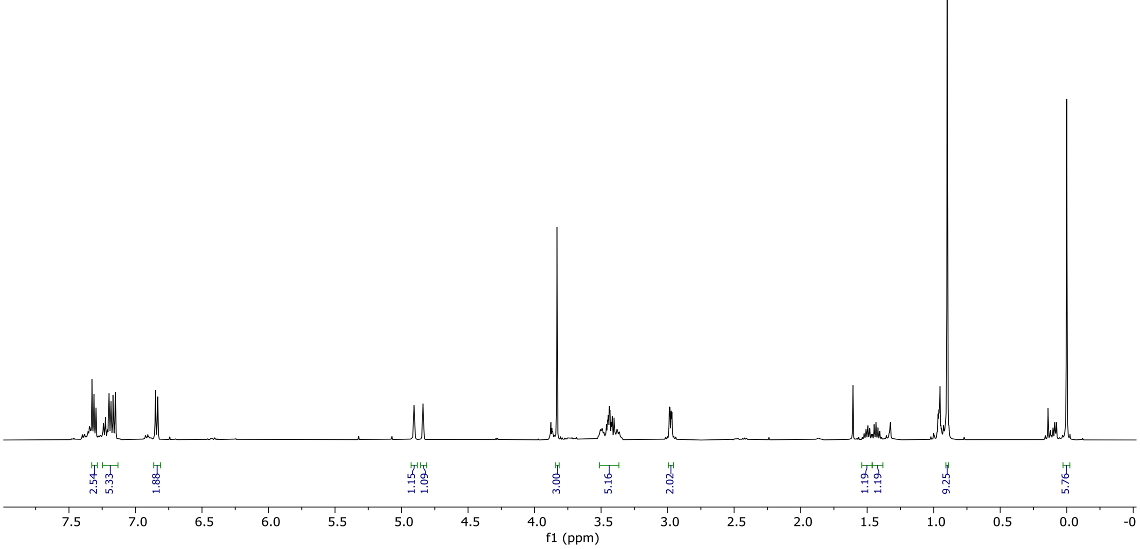

^{13}C NMR (126 MHz, $CDCl_3$)

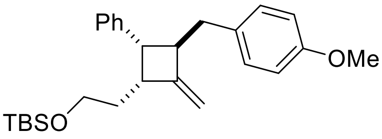

41

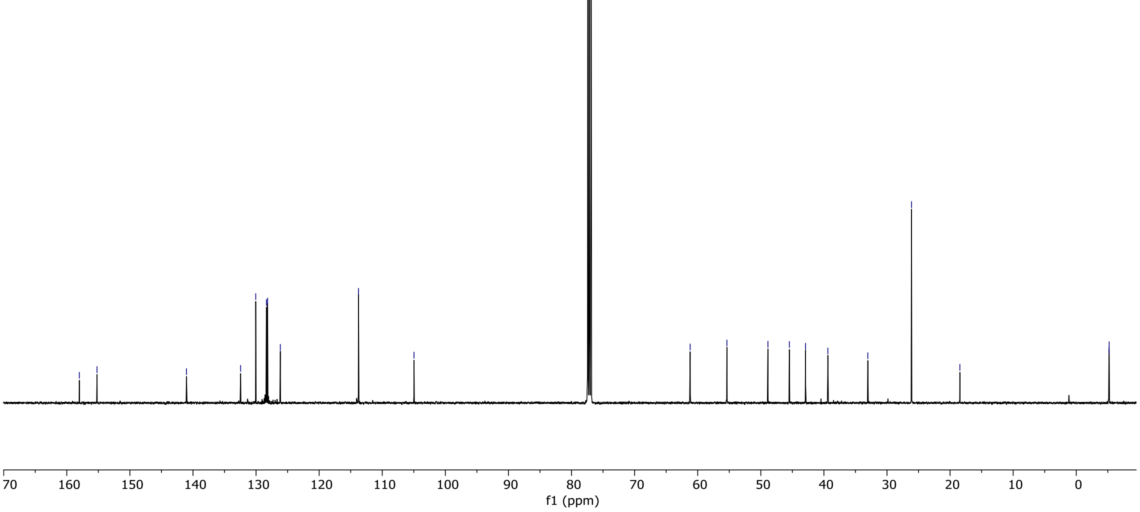

[1]**H NMR** (300 MHz, $CDCl_3$)

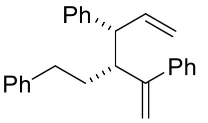

42

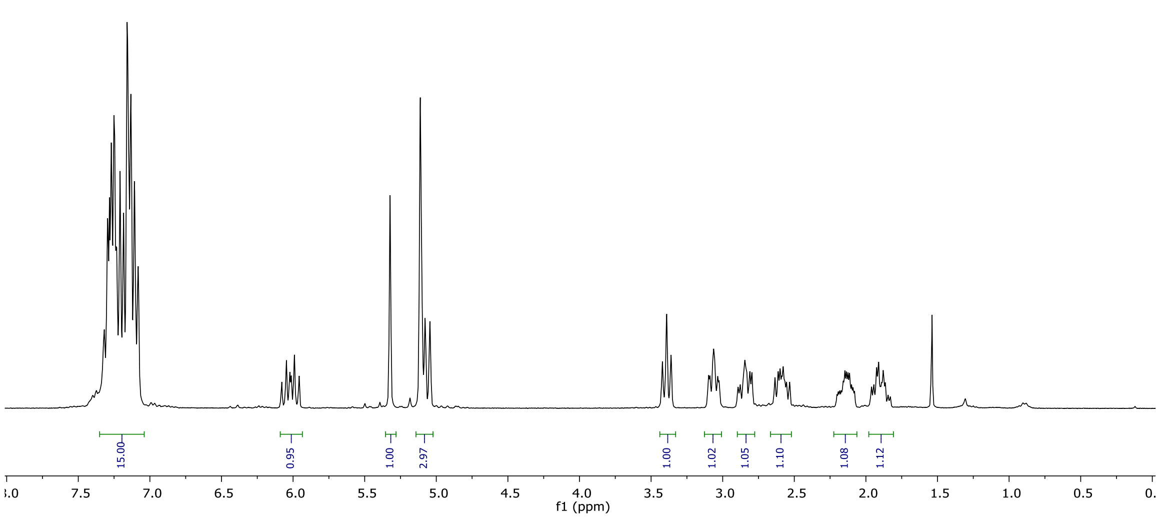

[13]**C NMR** (75 MHz, $CDCl_3$)

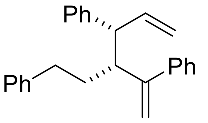

42

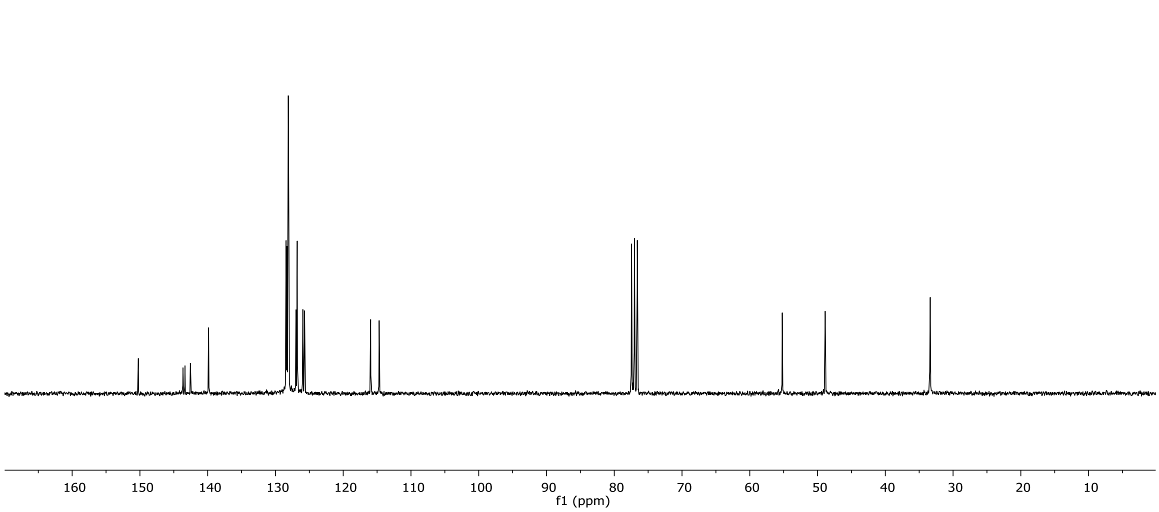

^{1}H NMR (300 MHz, $CDCl_3$)

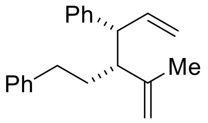

43

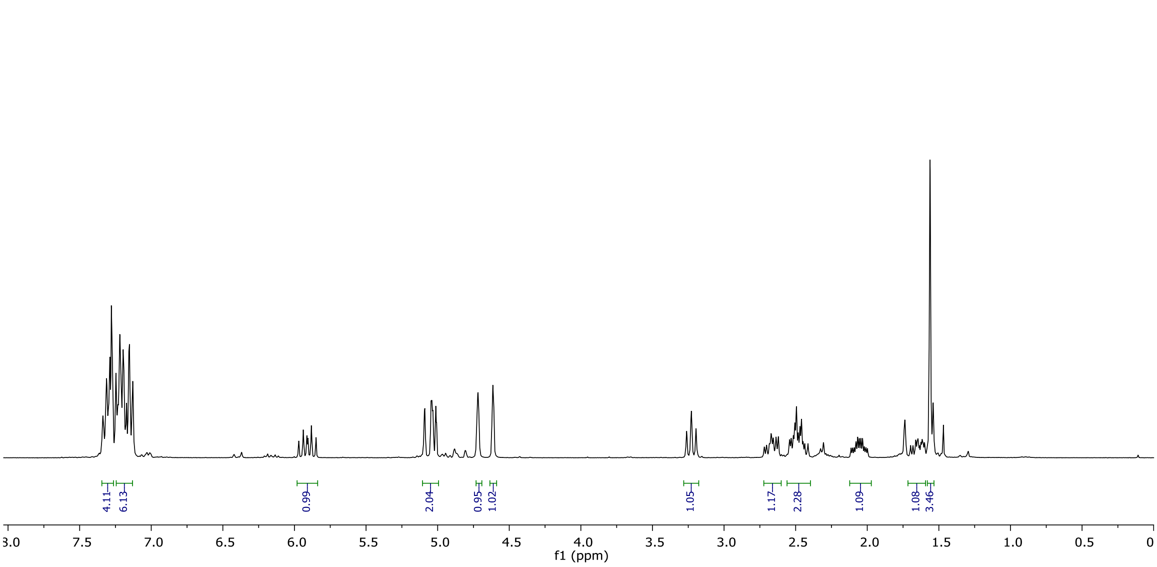

^{13}C NMR (75 MHz, $CDCl_3$)

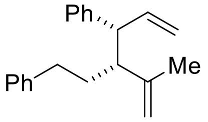

43

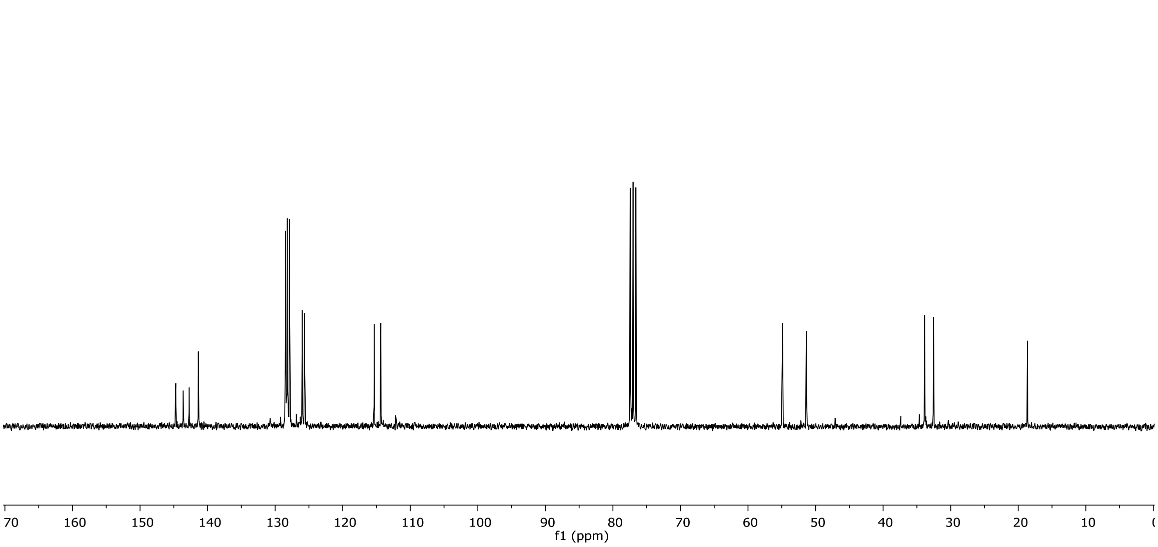

9. X-ray diffraction analysis data for product 3

Product **3** was dissolved in CH_2Cl_2 and left to slow evaporation overnight to yield block, clear colorless type of crystals of an approximate dimensions 0.11 × 0.10 × 0.05 mm. The X-ray intensity data were measured on a Bruker D8 VENTURE PHOTON-III C14 κ-geometry diffractometer system equipped with a Incoatec IµS 3.0 microfocus sealed tube (Cu Kα, λ = 1.54178 Å) and a multilayer mirror monochromator. The structure was solved and refined using the Bruker SHELXTL Software Package.

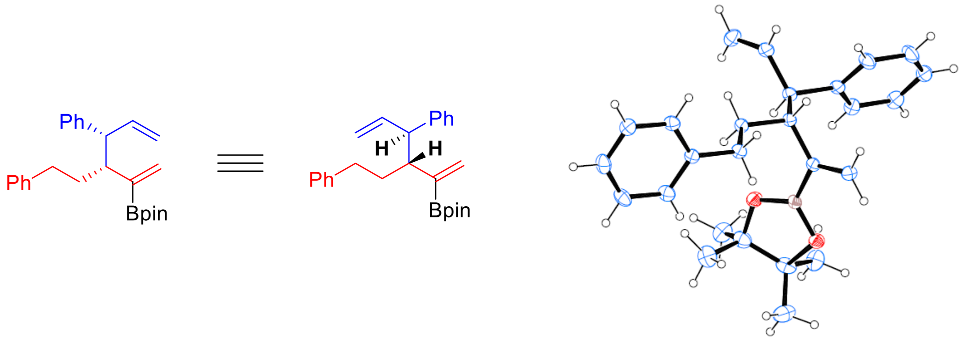

Figure 1. ORTEP plot of **3** with 30% ellipsoids

- **Crystal data**

Chemical formula	$C_{26}H_{33}BO_2$
Formula weight	388.33
Temperature	100 K
Wavelenght	0.71073 Å
Cristal size	0.11 × 0.10 × 0.05 mm
Cristal habit	Plate, clear colourless
Cristal system	Orthorhombic, *Pbca*
Unit cell dimensions	*a* = 11.9391 (10) Å
	b = 10.8076 (9) Å
	c = 35.919 (3) Å
Volume	4634.7 (7) $Å^3$
Z	8
Density (calculated)	1.113 g/cm^3

- **Data collection and structure refinement**

Diffractometer	Bruker D8 Venture Photon III-14
Radiation source	Microfocus sealed tube, Incoatec IµS 3.0
Theta range for data collection	2.3–22.0°
Reflections collected	80219
Independent reflections	4737
Coverage of independent reflections	
Absorption correction	Multi-Scan
Max. and min. transmission	1.00 and 0.88
Structure solution technique	Direct methods
Structure solution program	SHELXT 2018/2 (Sheldrick, 2015)
Refinement method	Full-matrix least-squares on F2
Refinement program	SHELXL-2019/1 (Sheldrick, 2019)

Function minimized	Σ w(Fo^2 - Fc^2)^2
Data / restraints / parameters	4737 / 185 / 326
Goodness-of-fit on	0.165
Final R indices	R[F2 > 2σ(F2)] = 0.055 wR(F2) = 0.165
Weighting scheme	$w = 1/[\sigma^2(F_o^2) + (0.0898P)^2 + 0.6961P]$ where $P = (F_o^2 + 2F_c^2)/3$ where $P=(F_o^2+2F_c^2)/3$
Largest diff. peak and hole	0.21 and -0.23 e Å^{-3}
R.M.S. deviation from mean	0.021 e Å^{-3}

10. X-ray diffraction analysis data for product (-)-30

Product **(-)-30** was dissolved in CH_2Cl_2 and left to slow evaporation overnight to yield block. A clear colorless, block-like specimen of $C_{21}H_{21}F_3O$, approximate dimensions 0.094 mm x 0.316 mm x 0.320 mm, was used for the X-ray crystallographic analysis. The X-ray intensity data were measured on a Bruker D8 VENTURE PHOTON-III C14 κ-geometry diffractometer system equipped with a Incoatec IμS 3.0 microfocus sealed tube (Mo Kα, λ = 0.71073 Å) and a multilayer mirror monochromator. The structure was solved and refined using the Bruker SHELXTL Software Package.

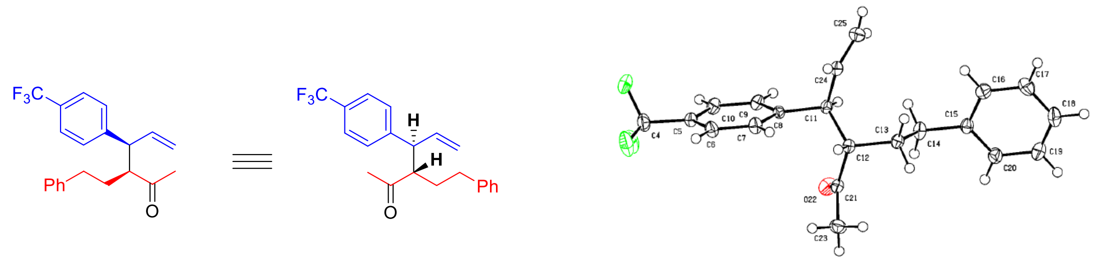

Figure 1. ORTEP plot of **(-)-30** with 30% ellipsoids

- **Crystal data**

Chemical formula	$C_{21}H_{21}F_3O$
Formula weight	346.38
Temperature	100(2) K
Wavelenght	0.71073 Å
Cristal size	0.094 × 0.316 × 0.320 mm
Cristal habit	Clear colourless block
Cristal system	Triclinic
Space group	P 1
Unit cell dimensions	*a* = 5.4040 (4) Å
	b = 8.6939 (6) Å
	c = 10.7504 (8) Å
Volume	453.68 (6) Å^3
Z	1
Density (calculated)	1.268 g/cm^3

- **Data collection and structure refinement**

Diffractometer	Bruker D8 Venture Photon III-14
Radiation source	Microfocus sealed tube, Incoatec IμS 3.0
Theta range for data collection	2.11–32.03°
Reflections collected	32918

Independent reflections	6015
Coverage of independent reflections	99.6%
Absorption correction	Multi-Scan
Max. and min. transmission	0.9910 and 0.9700
Structure solution technique	Direct methods
Structure solution program	SHELXT 2018/2 (Sheldrick, 2015)
Refinement method	Full-matrix least-squares on F2
Refinement program	SHELXL-2019/1 (Sheldrick, 2019)
Function minimized	Σ w(Fo^2 - Fc^2)^2
Data / restraints / parameters	6015 / 3 / 230
Goodness-of-fit on	1.081
Final R indices	5530 data; I>2σ(I) R1= 0.0491, wR2= 0.1093
	all data R1= 0.0557, wR2= 0.1132
Weighting scheme	$w = 1/[\sigma^2(F_o^2) + (0.0365P)^2 + 0.2220P]$ where $P = (F_o^2 + 2F_c^2)/3$
Absolute structure parameter	0.0(2)
Largest diff. peak and hole	0.403 and -0.231 e Å^{-3}
R.M.S. deviation from mean	0.048 e Å^{-3}

11. DFT calculations

11.1 Computational Details

Theoretical calculations were performed at DFT level of theory using Gaussian16 software.[20] The structures of all the intermediates and transition states were optimized in toluene solvent (DCM, ε= 2.37) with the SMD continuum model[21] using the B3LYP functional[22] combined with the Grimme's D3 correction for dispersion.[23] Basis set BS1 was used for the optimizations. BS1 includes the 6-31G(d,p) basis set for the main group elements,[24] and the scalar relativistic Stuttgart-Dresden SDD pseudopotential and its associated double-ζ basis set,[25] complemented with a set of *f* polarization functions,[26] for the Pd and Cu atoms. Frequency calculations were carried out for all the optimized geometries in order to characterize the stationary points as either minima or transition states. It was confirmed that transition states connect with the corresponding intermediates by usual intrinsic reaction coordinate (IRC) calculations and subsequent optimization to minima.

Gibbs energies in toluene were calculated replacing in the scheme above the potential energies obtained with BS1 by those obtained through single point calculations with an extended basis set (BS2). BS2 consists in the *def2*-TZVP basis set for the main group elements[26] and the quadruple-ζ *def2*-QZVP basis set for Pd and Cu.[27] A correction of 1.9 kcal mol^{-1} was applied to all Gibbs values to change the standard state from the gas phase (1 atm) to solution (1 M).[28]

11.2 Gibbs Energy Profiles

11.2.1 Copper cycle

The borocupration of the model allene **1^{M}** proceeds by hetero splitting of the B_2ed_2 forming the Cu-Bed intermediate with concomitant release of MeOBed at -15.7 kcal·mol^{-1}. The B-C bond is formed by insertion of one of the double bonds of the incoming allene **1^{M}** into the coordinated Cu-Bed moiety. The energy barriers for the C-B bond formation involving the concerted C-Cu

coordination to the internal or terminal C=C bonds have associated energy barriers of 13.4 and 15.3 kcal·mol^{-1}, respectively. The whole process is highly exergonic with the borocuprated allene complex, **IV^{Cu}**, falling at -51.7 kcal·mol^{-1}. The final borylated complex displays diverse coordination modes, i.e α (**IV^{Cu}**) and γ (**IV'^{Cu}**) allyl-Cu, in equilibrium with each other. The free energy barrier for the isomerization is quite affordable being of 8.2 kcal·mol^{-1} (Figure S1).

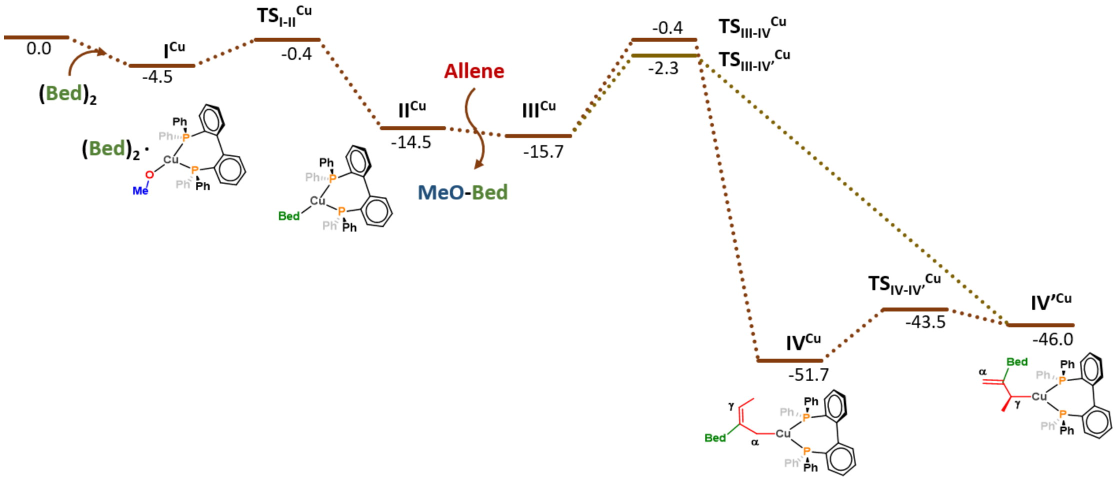

Figure S1. DFT computed mechanism (B3LYP-D3 in Tolene) for the borocupration of **1^{M}**. The numbers are relative Gibbs energies in kcal mol^{-1}, taking as zero-energy the separated co-catalyst and substrate **1^{M}**.

Moreover, for both species **IV^{Cu}** and **IV'^{Cu}** two conformational isomers with similar energy are possible (Scheme S1).

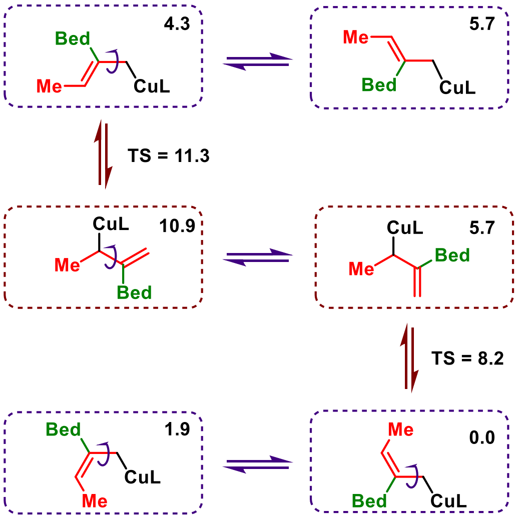

Scheme S1. Conformational isomers of the borylated complex allyl-Cu, in equilibrium with each other.

11.2.2 Palladium cycle

Oxidative addition of cinnamyl carbonate **2^{M}** through **TS$_{I\text{-}II}^{Pd}$** leads to intermediate **II^{Pd}** (η^{3}-coordination of the cinnamyl moiety) which evolves toward intermediate **III^{Pd}**, at 3.2 kcal·mol^{-1}, featuring a η^{1}-allyl fragment and $MeOCO_2^-$ coordinated in the equatorial plane. Decarboxylation via **TS$_{III\text{-}IV}^{Pd}$** (5.1 kcal·mol^{-1}) yields [Pd^{II}(**L$_1$**)(cinnamyl)(MeO)], **IV^{Pd}**, at 2.9 kcal·mol^{-1}. Intermediate

IV^{Pd} is in equilibrium with **IV'^{Pd}** (4.1 kcal·mol^{-1}) overcoming a low energetic Cα-Cβ bond rotational barrier of 14.6 kcal·mol^{-1} (Figure S2).

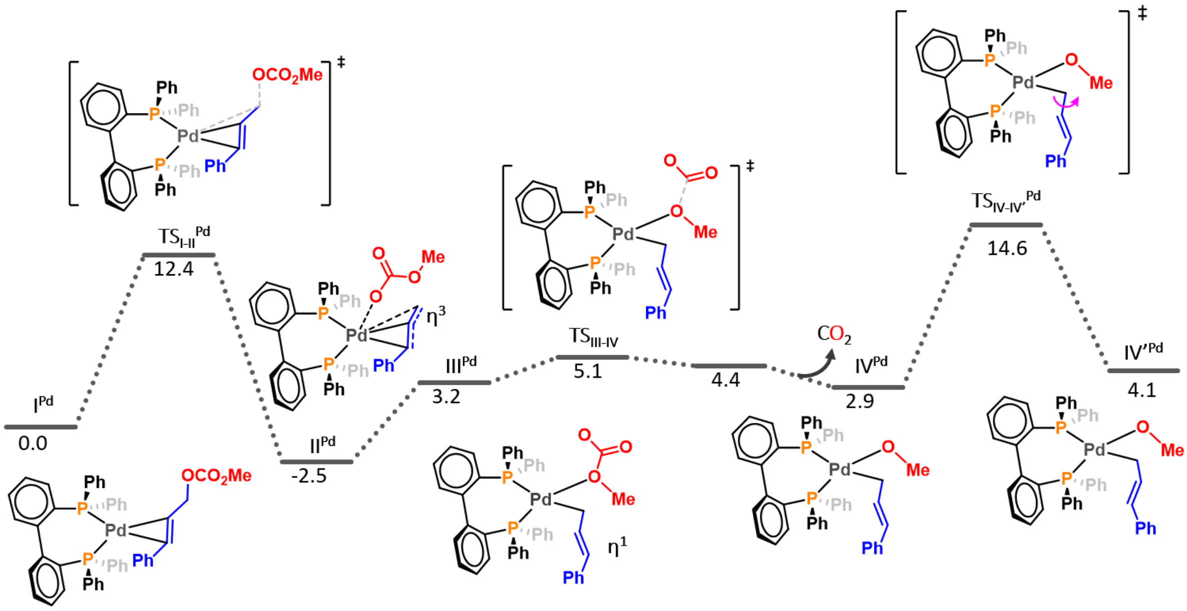

Figure S2. DFT computed mechanism (B3LYP-D3 in Toluene) of the Pd-based activation cycle of cinnamyl carbonate **2^M**. The numbers are relative Gibbs energies in kcal mol^{-1}, taking as zero-energy the separated co-catalyst and substrate **2^M**.

11.2.3 Copper-palladium cooperative cycle

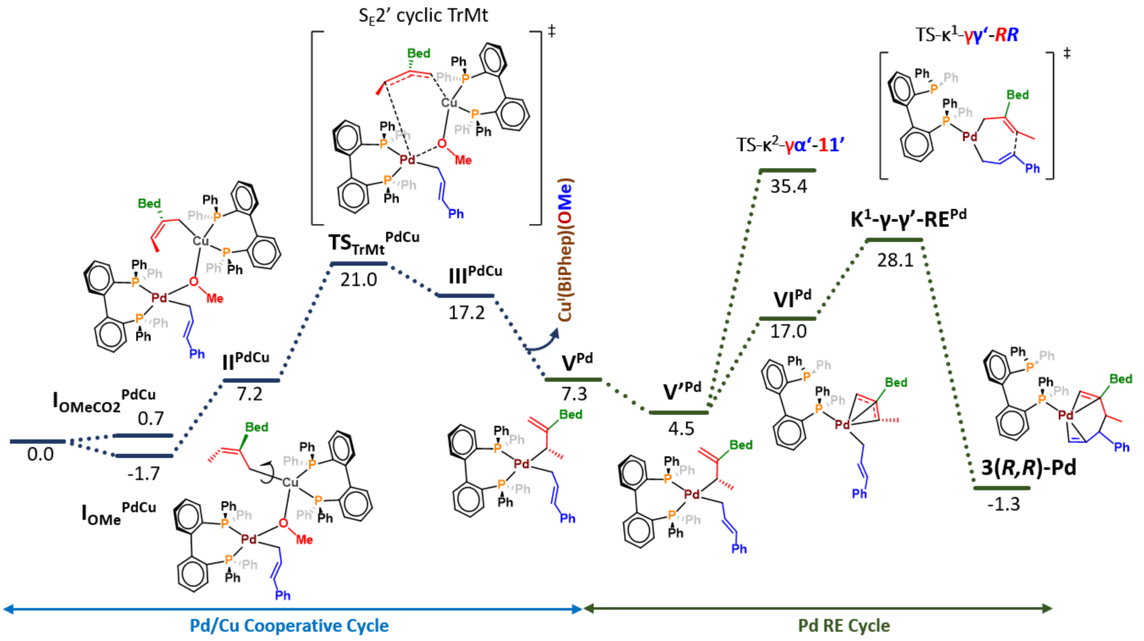

Figure S3. DFT computed general mechanism (B3LYP-D3 in Toluene) for PdCu γ-γ coupling of borylated allene **1^M** and allylic carbonate **2^M**. The numbers are relative Gibbs energies in kcal mol^{-1}, taking as zero-energy the separated activated co-catalysts **IV^{Cu}** and **IV^{Pd}**.

The aggregation of both activated co-catalysts occurs between **IV^{Pd}** and **IV^{Cu}** in which the Pd-Cu dinuclear species is connected by a O-methoxy bridge, **I_{MeO}^{PdCu}**. The O,O'-carboxy bridge species, **I_{OMeCO2}^{PdCu}**, between **III^{Pd}** and **IV^{Cu}** resulted higher in energy by 2.4 kcal·mol^{-1}. Moreover, no trasnmetalation was found starting from this species. The characterized transition state from **I_{MeO}^{PdCu}** is a concerted 6-membered S_E2' cyclic tranmetallation in which the methoxy coordinated by Pd is exchanged for the borylated allyl fragment originally coordinated to copper, forming the bis(allyl)Pd and the Cu-OMe intermediates (Figure S3).

The cycle is closed by reductive elimination (RE) over the bis(allyl)Pd complex. It was observed that k^1-coordination of BIPHEP is required to achieve this step, since RE from the k^2-BIPHEP falls at 35.4 kcal·mol^{-1}(**k^2-RE^{Pd}**).

Considering the possible coordination modes and conformational diversity of both **IV^{Pd}** and **IV^{Cu}**, five different transition states TrMt's (and their corresponding enantiomeric forms) leading either to pro-*R** and pro-*S** Cγ both at the cinnamyl and borylated allyl fragments in the resulting bis(allyl)Pd are possible (Figure S4). We fully explored the prochirality of the borylated allyl fragment and additionally, for the lowest TrMt, the pro-chirality of the cinnamyl fragment as a representative case. The computed TrMts are reported in Figure S4.

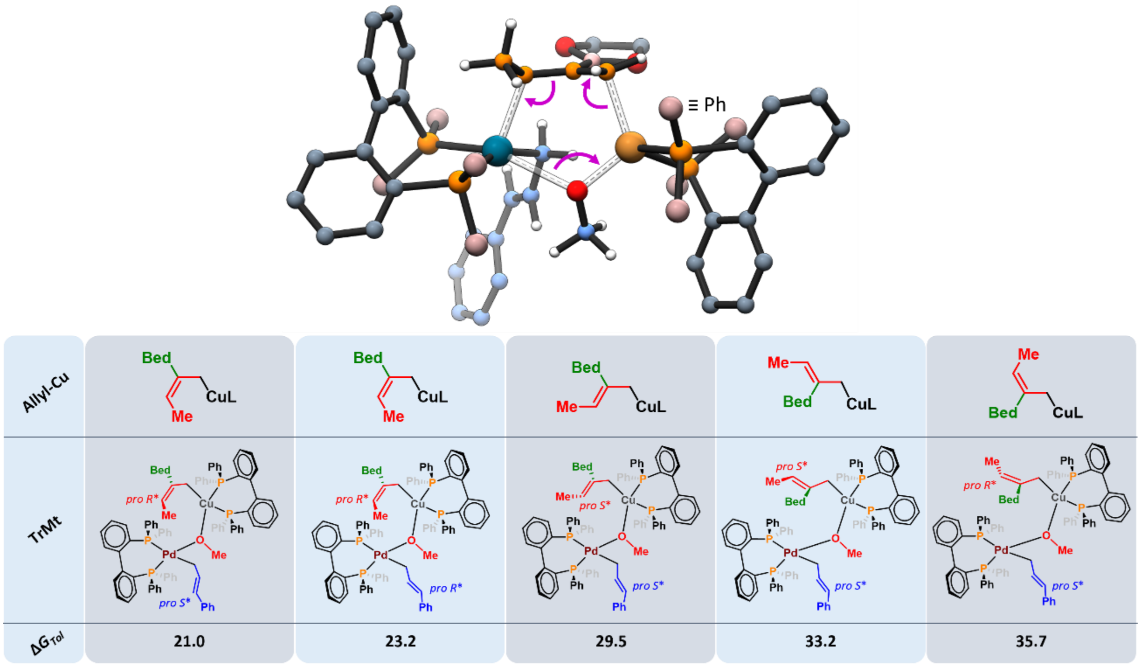

Figure S4. Molecular structure of the computed most stable TrMt (top; bond breaking and forming are shown through curly arrows) and schematization of all the explored prochiral orientations (bottom) along with their relative ΔG_{Tol} (kcal·mol^{-1}) values for the computed transition states.

11.2.4 Calculations with real substrates

*11.2.4.1. **IV^Cu** to **IV'^Cu** and **V^Pd** to **V'^Pd** isomerization processes*

The origins of the regio- and diastereoselectivity of the reaction were studied by using real substrates **1**, **2** and B_2pin_2. First, the isomerization process for both **IV^{Cu}** and **V^{Pd}** intermediates was assessed (Scheme SC2). On the one hand, the real borylated allyl-Cu **IV^{Cu}Real** (α coordination of the borylated allyl moiety) results in equilibrium with **IV'^{Cu}Real** (γ coordination of the borylated allyl moiety) featuring a quite low isomerization barrier of 8.9 kcal·mol^{-1} (Scheme S2a). This is in line with the results obtained using the model substrate **1^{M}** (see Scheme S1). On the other hand, the rotation along the Cα-Cβ bond of the cinnamyl moiety in **V^{Pd}** results affordable, with an activation energy of 13.0 kcal·mol^{-1}. This result indicates that, after transmetalation, both *Re* and *Si* faces of Cγ are accessible for the formation of the C-C bond (Scheme S2b).

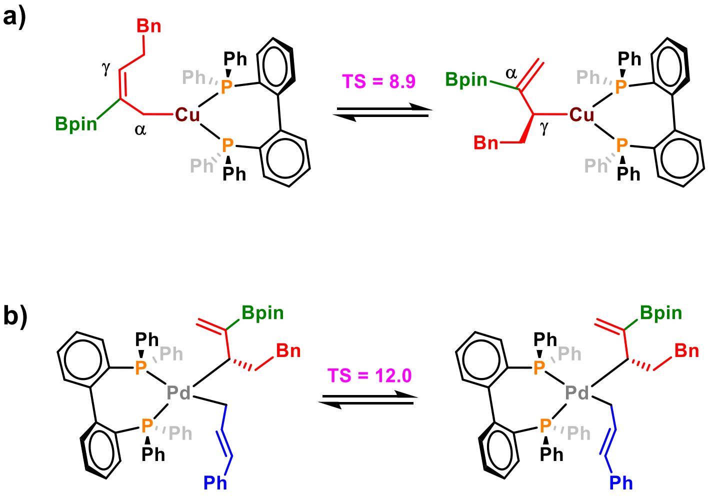

Scheme S2. Isomerization processes for: a) **IV^{Cu}Real** borylated allyl-Cu; and b) **V^{Pd}Real** bis(allyl)Pd intermediate along with the activation free energy barriers in kcal·mol^{-1}.

11.2.4.2 Reductive elimination with real substrates

Pathways leading to either the γ-γ' and γ-α' coupling products, and the formation of both γ-γ' diastereomers were fully explored (Figure S5). Starting from the k^2-BIPHEP bis(allyl)Pd intermediate (**V^{Pd}Real**) coming from the transmetalation step, only a pathway for the formation of γ-α' coupling product could be observed through a 1,1'-RE with an energy barrier of 33.7 kcal·mol^{-1}. Therefore, we extended our exploration to the k^1-BIPHEP bis(allyl)Pd intermediate (**VI^{Pd}Real**), but similarly, the 1,1'-RE that generates the γ-α' coupling product displayed a high barrier of 34.3 kcal·mol^{-1}. However, the formation of the γ-γ' coupling product through a 3,3'-RE mechanism features an affordable energy barrier of 22.1 kcal·mol^{-1}. A lower energy barrier for the formation of the γ-α' coupling product (24.9 kcal·mol^{-1}) was achieved from **VI'^{Pd}Real** by considering an isomerization to a branched cinnamyl fragment and further 3,3'-RE from a Pd intermediate in which the borylated allyl fragment is coordinated to Pd through the C-α. Considering the lowest energetic pathways for both regioisomeric γ-γ' and γ-α' coupling

products, the resulting $\Delta\Delta G_{Tol}$ of 2.8 kcal·mol^{-1}, clearly reproduces the experimentally observed >95% regioselectivity of the reaction.

Our results also supported the observed diastereoselectivity of the reaction since the activation energy barrier for the pro-*R**,*R** transition state is favored by 8.5 kcal·mol^{-1} compared to the lowest energy pathway for the pro-*R**,*S** γ-γ' coupling product.

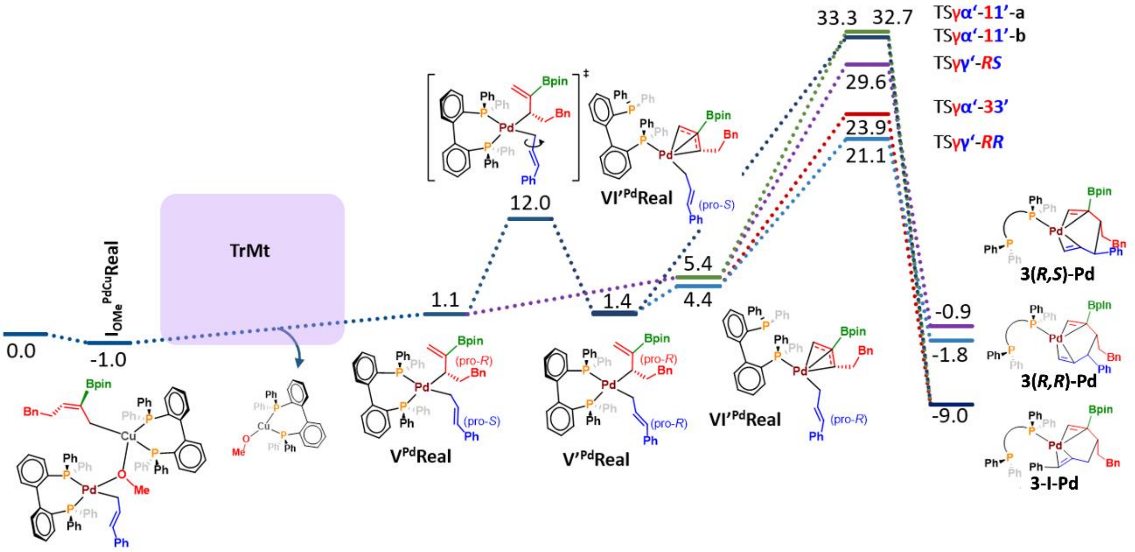

Figure S5. DFT computed (B3LYP-D3 in Toluene) RE over the bis(allyl)Pd derived from the real substrates, **1**, **2** and B_2pin_2. The numbers are relative Gibbs energies in kcal·mol^{-1}, taking as zero-energy the separated activated co-catalysts **IV^{Cu}Real** and **IV^{Pd}Real**.

To validate our model and gain further insight into the factors that influence the diastereoselectivity of the reaction, we further analyzed the reductive elimination step over the bis(allyl)Pd derived from the real substrates **1**, B_2pin_2 and **58** (Figure S6). This reaction led to the formation of product **16** that features a diminished 3:1 dr (see Scheme 2 in the main text). The main difference in this reaction is the replacement of the phenyl ring in **2** by a methyl group. On the one hand, the smaller volume of the methyl group reduces steric clashes between the substituent in the blue allyl fragment and the Bpin unit of the borylated red allyl fragment. This reduction in steric hindrance lowers the energy barrier of the transition state **TSγγ'-*RS*** compared to when the bulkier phenyl group is present. On the other hand, the absence of the phenyl group removes the favorable π-π stacking interactions between the phenyl and benzyl groups of the two allyl fragments, which would result in an increase of the energy of the transition state **TSγγ'-RR** in the formation of product **16**. Consequently, this twofold effect results in a reduction of the energy span between both transition states from 8.5 to 2.5 kcal·mol^{-1}. This corresponds to a 97% selectivity, aligning closely with the observed decrease in diastereoselectivity (3:1 ratio for product **16**), considering associated errors.

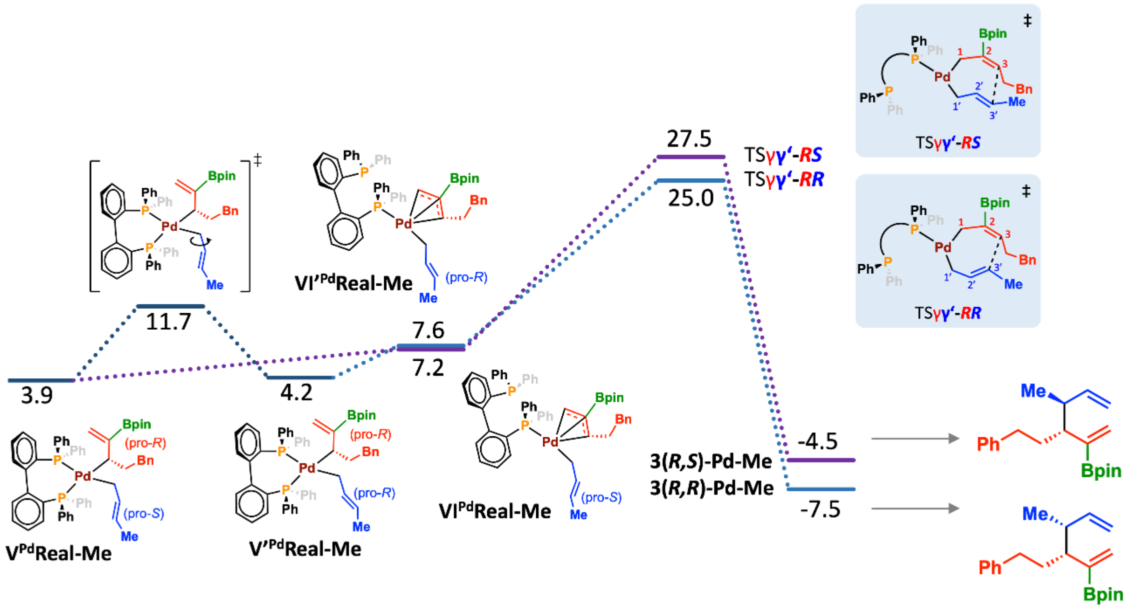

Figure S6. DFT computed (B3LYP-D3 in toluene) free energy profile for the reductive elimination over the bis(allyl)Pd derived from the real substrates **1**, B_2pin_2 and **58** (formation of product **16**), along with the corresponding transition state and products structures. The numbers are relative Gibbs energies in kcal·mol^{-1}, referred to $\mathbf{I_{OMe}^{PdCu}Real}$. The last final Gibbs energy values refer to the Pd-coordinated intermediate.

11.2.4.3. η^1–η^3–η^1 isomerization of the borylated allyl fragment

We additionally explored the internal to terminal isomerization of the borylated-allyl fragment, obtaining a relative energy higher by 14.7 kcal·mol^{-1} for the terminal (**η^1-VIPdReal**) compared to the internal (**η^3-VI^{Pd}Real**) coordination mode. Moreover, from **η^1-VIPdReal**, the estimated rotational barrier along the simple C1-C2 bond which would entail a change in the prochirality of the borylated allyl fragment, resulted in 36.4 kcal·mol^{-1} (Figure S7). Thus, pro-R* to pro-S* isomerization of the borylated allyl fragment results sterically hindered indicating that the transmetalation step defines itself the chiral configuration at C3. This path is not competitive with those reported in the main text.

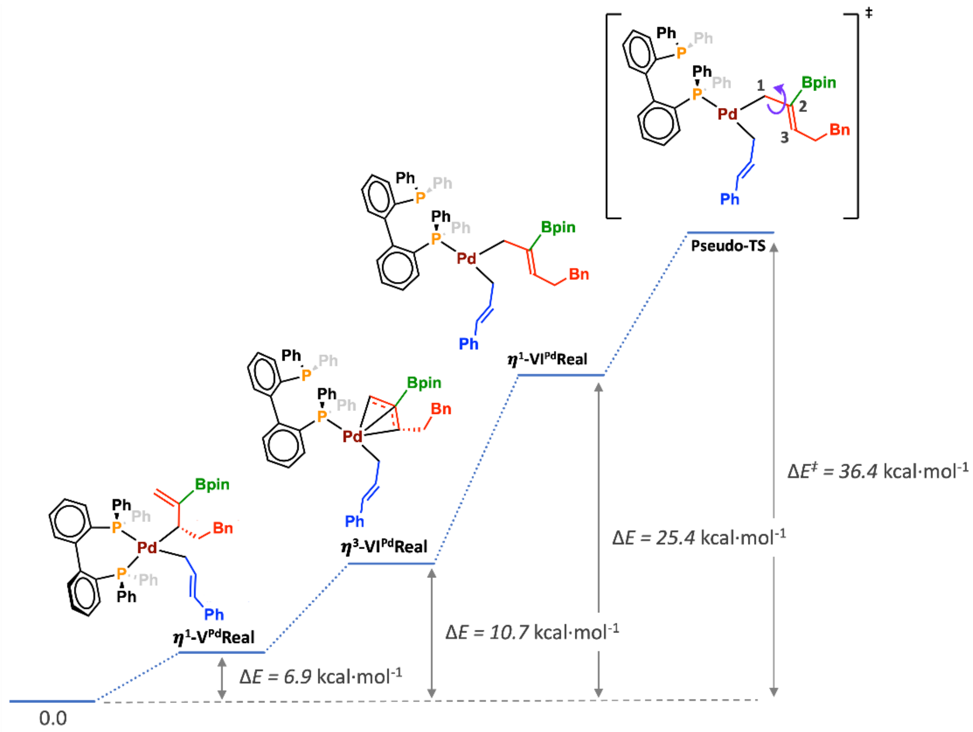

Figure S7. Potential electronic energy profile (B3LYP-D3) of the η^1–η^3–η^1 and pro-*R** to pro-*S** isomerization processes. The rotational barrier was estimated by freezing the core of the Pd complex and exploring the coordinate defined as the Pd-C1-C2-C3 dihedral.

11.3 Cartesian coordinates and absolute energies of the optimized structures

I^{Cu}

	Value
Charge	0
Electronic Energy, BS1 (a.u.)	-2892.152327
Thermal and entropic correction, BS1 (a.u.)	0.630291
Electronic Energy, BS2 (a.u.)	-4336.201007

Molecular Geometry in Cartesian Coordinates

Cu	-0.052622	0.756719	0.232560
P	1.685616	-0.640544	-0.155060
P	-1.915655	-0.456151	0.224397
C	2.887369	-0.140673	-1.443061
C	2.673532	-0.891322	1.368688
C	1.134590	-2.332111	-0.660564
C	-2.279570	-1.443678	-1.278765
C	-1.433660	-1.614428	1.578083
C	-3.529916	0.276245	0.704055
C	2.444416	0.762252	-2.421063
C	4.205765	-0.618016	-1.484283
C	2.739104	0.189051	2.264562
C	3.342783	-2.086345	1.669585
C	0.151185	-3.029112	0.085533
C	1.609403	-2.903746	-1.852006
C	-1.655619	-1.039827	-2.468043
C	-3.153797	-2.540262	-1.297827
C	-1.910363	-1.350508	2.874360
C	-0.408427	-2.580941	1.408289
C	-3.633571	1.671575	0.600467
C	-4.645159	-0.464451	1.129548
H	1.447017	1.185610	-2.355504
C	3.304820	1.155051	-3.446796
C	5.065613	-0.212851	-2.505884
H	4.562862	-1.299854	-0.718125
C	3.480566	0.072171	3.441331
H	2.196155	1.104589	2.037824
C	4.077531	-2.198005	2.851105
H	3.282158	-2.929258	0.987012
C	-0.294323	-4.274276	-0.381407
H	2.357505	-2.378635	-2.434701
C	1.138104	-4.133720	-2.309370
H	-0.962614	-0.202840	-2.448288
C	-1.911072	-1.715381	-3.663020
H	-3.623840	-2.880691	-0.380706
C	-3.407103	-3.213443	-2.491629
H	-2.682554	-0.602595	3.018664
C	-1.405752	-2.021870	3.986467
C	0.076781	-3.250531	2.542525
H	-2.771804	2.252637	0.287768
C	-4.834031	2.317125	0.905756
H	-4.576285	-1.541946	1.240459
C	-5.841002	0.182615	1.437864
H	2.958283	1.860454	-4.196784
C	4.613325	0.668952	-3.491390
H	6.087035	-0.582418	-2.530613
C	4.148693	-1.118934	3.736250
H	3.528965	0.910249	4.131060
H	4.592946	-3.126590	3.081030
C	0.181308	-4.824893	-1.570138
H	1.521990	-4.546002	-3.238094
H	-1.419499	-1.397197	-4.577863
C	-2.788661	-2.800177	-3.675859
H	-4.082489	-4.064397	-2.498612
C	-0.403027	-2.975503	3.821436
H	-1.794498	-1.794238	4.974762
H	-4.897426	3.398485	0.825368
C	-5.938308	1.573363	1.323181

H	-6.697555	-0.397066	1.770335
H	5.284629	0.985961	-4.284905
H	4.719750	-1.208820	4.656230
H	-0.197733	-5.782899	-1.913806
H	-2.986772	-3.328696	-4.604099
H	0.005944	-3.501948	4.678687
H	-6.871755	2.073394	1.565606
H	0.857861	-3.992324	2.407913
H	-1.042111	-4.807013	0.198681
H	-0.090233	3.626653	2.903397
C	-0.578992	2.892301	2.251830
H	-0.937535	2.055051	2.862026
H	-1.443665	3.385321	1.786465
O	0.340416	2.404774	1.286993
B	0.838377	3.462120	0.193436
O	2.072845	2.871326	-0.320736
O	1.207670	4.684186	0.881399
C	3.159556	3.486897	0.350435
C	2.512855	4.440280	1.381141
H	3.778802	4.040820	-0.373658
H	3.804595	2.735889	0.830524
H	3.060441	5.386353	1.487411
H	2.473421	3.963958	2.376897
B	-0.453123	3.506334	-0.960555
O	-0.505828	2.625138	-2.047432
O	-1.630127	4.235717	-0.871275
C	-1.803539	2.711847	-2.664861
C	-2.466390	3.934476	-2.004076
H	-2.354885	1.785112	-2.468241
H	-1.687048	2.821115	-3.747798
H	-3.485507	3.727081	-1.661537
H	-2.494111	4.805678	-2.669736

TSI-II^{Cu}

	Value
Charge	0
Electronic Energy, BS1 (a.u.)	-2892.148455
Thermal and entropic correction, BS1 (a.u.)	0.631266
Electronic Energy, BS2 (a.u.)	-4336.194161

Molecular Geometry in Cartesian Coordinates

Cu	-0.058050	0.846271	-0.486501
P	-1.644558	-0.720372	0.083278
P	1.896589	-0.231772	-0.138732
C	-3.216580	-0.116953	0.821341
C	-2.153423	-1.781638	-1.324793
C	-0.989660	-1.902914	1.352616
C	2.252424	-0.455744	1.654898
C	1.605134	-1.900644	-0.870663
C	3.535137	0.308205	-0.783139
C	-3.180283	1.115071	1.491808
C	-4.422154	-0.829785	0.755264
C	-2.024132	-1.244875	-2.615049
C	-2.653414	-3.082958	-1.162017
C	0.133723	-2.728508	1.090318
C	-1.541682	-1.908197	2.643763
C	1.497249	0.289285	2.571895
C	3.278018	-1.288440	2.126145
C	2.065786	-2.133518	-2.180126
C	0.770571	-2.868181	-0.261576
C	3.722953	1.685940	-0.971246
C	4.608431	-0.568776	-1.015895
H	-2.264881	1.695552	1.510431
C	-4.330028	1.611506	2.107365
C	-5.572921	-0.322641	1.361338
H	-4.471759	-1.773966	0.223273
C	-2.400392	-2.003733	-3.725613
H	-1.627294	-0.240016	-2.741862

C	-3.029659	-3.834653	-2.274716
H	-2.732718	-3.512291	-0.167274
C	0.636626	-3.536550	2.119840
H	-2.400178	-1.282343	2.859780
C	-1.011558	-2.701559	3.660494
H	0.701553	0.936527	2.213028
C	1.759389	0.196765	3.940708
H	3.875210	-1.865674	1.427141
C	3.536373	-1.381051	3.492360
H	2.670102	-1.382501	-2.677324
C	1.765320	-3.311242	-2.859748
C	0.510021	-4.063343	-0.951408
H	2.906057	2.372436	-0.769852
C	4.962680	2.175077	-1.390763
H	4.479211	-1.638486	-0.884564
C	5.841657	-0.075438	-1.440242
H	-4.291225	2.567424	2.622458
C	-5.527805	0.895800	2.042827
H	-6.503904	-0.879463	1.299093
C	-2.902615	-3.295583	-3.558158
H	-2.296502	-1.584046	-4.722339
H	-3.414570	-4.842056	-2.141525
C	0.082971	-3.522540	3.398579
H	-1.459235	-2.678225	4.649941
H	1.164288	0.774201	4.642586
C	2.777068	-0.638563	4.402266
H	4.329971	-2.031958	3.848664
C	0.996158	-4.292215	-2.235521
H	2.135278	-3.458769	-3.870211
H	5.095571	3.243852	-1.534018
C	6.021210	1.297994	-1.629567
H	6.662455	-0.763514	-1.622987
H	-6.424819	1.289194	2.513459
H	-3.191064	-3.884084	-4.424869
H	0.507429	-4.145323	4.180544
H	2.980459	-0.712395	5.466933
H	0.758185	-5.218858	-2.749152
H	6.982595	1.679592	-1.962019
H	-0.117411	-4.808031	-0.471705
H	1.491440	-4.171826	1.907436
H	0.359479	2.938579	-3.887202
C	0.605974	2.162153	-3.152406
H	0.981129	1.277839	-3.679971
H	1.404046	2.546453	-2.505261
O	-0.544675	1.782746	-2.410574
B	-1.034961	2.735368	-1.394104
O	-2.433547	2.482443	-1.129660
O	-0.896542	4.155309	-1.714716
C	-3.027720	3.733257	-0.842317
C	-2.201018	4.729007	-1.668809
H	-2.950668	3.964167	0.232836
H	-4.089436	3.722212	-1.116770
H	-2.160689	5.730924	-1.221814
H	-2.607617	4.824626	-2.688470
B	0.027480	2.931166	0.206034
O	-0.611764	3.188035	1.421721
O	1.308726	3.483635	0.215136
C	0.216827	4.059723	2.214260
C	1.588734	4.010751	1.523276
H	0.243308	3.699861	3.247695
H	-0.216526	5.067625	2.204669
H	2.286097	3.335678	2.036346
H	2.057128	4.995628	1.432792

II^{Cu}

	Value
Charge	0
Electronic Energy, BS1 (a.u.)	-2892.171216
Thermal and entropic correction, BS1 (a.u.)	0.632190
Electronic Energy, BS2 (a.u.)	-4336.218920

Molecular Geometry in Cartesian Coordinates

Cu	-0.249911	0.952920	0.391904
P	-1.042406	-1.232905	0.124839
P	1.997137	0.546011	-0.024203
C	-2.678154	-1.483534	0.944260
C	-1.143034	-2.177670	-1.448036
C	0.129090	-2.248815	1.147461
C	2.969666	0.187088	1.496824
C	2.311706	-0.904756	-1.116891
C	2.899156	1.959992	-0.786059
C	-3.030947	-0.558589	1.941631
C	-3.583201	-2.502284	0.613144
C	-1.166233	-1.462128	-2.652590
C	-1.171409	-3.581836	-1.481025
C	1.444332	-2.533764	0.693742
C	-0.237452	-2.646058	2.443458
C	2.412241	0.571600	2.724501
C	4.221892	-0.445446	1.478474
C	2.759998	-0.730100	-2.436428
C	1.960573	-2.208104	-0.680419
C	2.184404	3.156431	-0.947254
C	4.252712	1.914095	-1.160632
H	-2.365868	0.269505	2.166976
C	-4.250888	-0.668309	2.611308
C	-4.809075	-2.601288	1.275035
H	-3.346855	-3.210483	-0.172994
C	-1.227789	-2.142710	-3.870689
H	-1.136127	-0.378226	-2.637213
C	-1.240197	-4.257572	-2.698604
H	-1.120500	-4.146264	-0.554141
C	2.316113	-3.227689	1.545647
H	-1.238692	-2.436116	2.803035
C	0.655382	-3.310762	3.283767
H	1.435528	1.049713	2.737486
C	3.098728	0.330112	3.916416
H	4.650185	-0.774556	0.536256
C	4.906440	-0.683640	2.669305
H	3.007630	0.264063	-2.791451
C	2.897548	-1.811230	-3.306324
C	2.140075	-3.284628	-1.560562
H	1.148155	3.209059	-0.626008
C	2.809359	4.283302	-1.488589
H	4.821838	0.998218	-1.035544
C	4.872074	3.039092	-1.702679
H	-4.506763	0.057719	3.378104
C	-5.143949	-1.690180	2.279981
H	-5.503599	-3.391092	1.001928
C	-1.268683	-3.537480	-3.896497
H	-1.241399	-1.578735	-4.799151
H	-1.261879	-5.343972	-2.713439
C	1.939222	-3.607753	2.832286
H	0.341133	-3.599359	4.282886
H	2.655350	0.625894	4.863095
C	4.345585	-0.296503	3.890284
H	5.874027	-1.177693	2.646676
C	2.599005	-3.098003	-2.863350
H	3.242404	-1.644069	-4.322805
H	2.245358	5.204526	-1.605682
C	4.149907	4.225510	-1.870147
H	5.918410	2.992623	-1.991855
H	-6.098433	-1.770692	2.792951
H	-1.315561	-4.064130	-4.845834
H	2.646054	-4.125774	3.473801
H	4.878185	-0.488378	4.817690
H	2.705526	-3.950329	-3.527794
H	4.635828	5.101282	-2.291540
H	1.881728	-4.281434	-1.217036
H	3.317662	-3.448600	1.189785
H	-1.611257	3.801986	-3.327740
C	-1.085102	3.156644	-2.613061
H	-0.072613	2.975009	-2.982428
H	-1.032136	3.650411	-1.640126
O	-1.742431	1.888619	-2.516448

B	-2.835702	1.752913	-1.723297
O	-3.463539	0.530891	-1.591992
O	-3.458263	2.778762	-1.039096
C	-4.680203	0.772426	-0.864294
C	-4.469577	2.155378	-0.220153
H	-4.833566	-0.019524	-0.129692
H	-5.521191	0.771303	-1.570495
H	-4.082017	2.071776	0.799562
H	-5.373721	2.771696	-0.222909
B	-1.137677	2.609214	1.111627
O	-2.138395	2.671397	2.094770
O	-0.874897	3.905921	0.643268
C	-2.507263	4.046294	2.321931
C	-1.881324	4.805110	1.145199
H	-2.105424	4.373489	3.290062
H	-3.598746	4.133430	2.353283
H	-1.419322	5.752485	1.441479
H	-2.610163	4.995166	0.348153

III^{Cu}

	Value
Charge	0
Electronic Energy, BS1 (a.u.)	-2678.948629
Thermal and entropic correction, BS1 (a.u.)	0.598597
Electronic Energy, BS2 (a.u.)	-4122.907918

Molecular Geometry in Cartesian Coordinates

Cu	0.001145	1.242592	0.107801
P	-1.714581	-0.325301	0.117263
P	1.798744	-0.207806	-0.006135
C	-3.325443	0.370747	0.698635
C	-2.179691	-1.462387	-1.251838
C	-1.175475	-1.470160	1.472116
C	2.507737	-0.575933	1.652218
C	1.433096	-1.871245	-0.709678
C	3.233487	0.445882	-0.958402
C	-3.276432	1.607254	1.365735
C	-4.571304	-0.244365	0.497744
C	-1.859832	-1.080586	-2.561161
C	-2.832488	-2.687625	-1.036612
C	-0.132076	-2.413994	1.276018
C	-1.708827	-1.307679	2.760734
C	2.212989	0.313097	2.695992
C	3.312691	-1.696216	1.908687
C	1.943098	-2.263147	-1.958171
C	0.507652	-2.721420	-0.050349
C	3.112781	1.742419	-1.481623
C	4.434931	-0.258389	-1.144446
H	-2.320994	2.109511	1.493474
C	-4.446668	2.200485	1.841664
C	-5.740562	0.359969	0.962426
H	-4.638401	-1.187394	-0.032997
C	-2.201261	-1.899110	-3.639903
H	-1.340383	-0.144048	-2.730471
C	-3.176492	-3.501957	-2.114586
H	-3.053802	-3.011432	-0.023603
C	0.301938	-3.172440	2.373552
H	-2.502246	-0.585877	2.922169
C	-1.241883	-2.054030	3.842046
H	1.578159	1.173573	2.498292
C	2.719159	0.084870	3.977361
H	3.523170	-2.407082	1.114879
C	3.818701	-1.919900	3.188408
H	2.633857	-1.612926	-2.483313
C	1.585312	-3.478941	-2.540159
C	0.192204	-3.955476	-0.636123
H	2.202375	2.307849	-1.301960
C	4.167171	2.316527	-2.196512

H	4.549134	-1.257105	-0.734564
C	5.484647	0.316255	-1.859414
H	-4.390955	3.155000	2.357597
C	-5.681103	1.579933	1.640170
H	-6.698729	-0.124009	0.793435
C	-2.863001	-3.107586	-3.418931
H	-1.945733	-1.592307	-4.650270
H	-3.681005	-4.447918	-1.937461
C	-0.233008	-2.995010	3.647935
H	-1.671410	-1.900492	4.827931
H	2.479211	0.775117	4.781227
C	3.521962	-1.029787	4.225101
H	4.438394	-2.791868	3.379626
C	0.716294	-4.337321	-1.870219
H	1.993508	-3.753262	-3.508821
H	4.060821	3.321201	-2.595830
C	5.350635	1.603668	-2.390039
H	6.408971	-0.237242	-2.000098
H	-6.593140	2.047432	2.001115
H	-3.127084	-3.745642	-4.257777
H	0.141544	-3.584496	4.479632
H	3.912184	-1.208979	5.223171
H	0.434215	-5.290561	-2.307389
H	6.170962	2.049414	-2.945804
H	-0.503766	-4.609631	-0.120000
H	1.095164	-3.897084	2.218139
H	1.078577	1.040228	-3.488606
C	0.404123	1.751917	-3.980017
H	1.034475	2.430788	-4.567424
H	-0.240846	1.198992	-4.669289
C	-0.394098	2.516337	-2.951791
C	-1.696244	2.462215	-2.815643
B	0.081555	3.180539	0.621638
O	-0.373049	3.760998	1.812683
O	0.623073	4.179370	-0.204575
C	-0.041914	5.163272	1.826235
C	0.389456	5.472980	0.384562
H	0.768850	5.335549	2.546674
H	-0.913246	5.745472	2.145143
H	1.303609	6.073915	0.331803
H	-0.396976	5.990118	-0.180919
H	0.154938	3.137400	-2.243334
C	-2.993961	2.407560	-2.659551
H	-3.662349	3.101368	-3.167298
H	-3.454494	1.668136	-2.006320

TSIII-IV^{Cu}

	Value
Charge	0
Electronic Energy, BS1 (a.u.)	-2678.933187
Thermal and entropic correction, BS1 (a.u.)	0.605905
Electronic Energy, BS2 (a.u.)	-4122.890795

Molecular Geometry in Cartesian Coordinates

Cu	0.185770	1.142567	-0.525025
P	-1.749245	-0.056671	0.054172
P	1.735450	-0.406998	0.011686
C	-3.096994	0.959430	0.798936
C	-2.651820	-0.919413	-1.296155
C	-1.444118	-1.366389	1.330643
C	1.966191	-0.765240	1.805000
C	1.144688	-1.970233	-0.774265
C	3.475380	-0.202841	-0.559461
C	-2.760941	2.206354	1.343004
C	-4.432951	0.527096	0.843135
C	-2.802426	-0.207470	-2.496162
C	-3.219343	-2.195058	-1.179543
C	-0.575997	-2.461016	1.085442

C	-2.018460	-1.237240	2.605966
C	1.414868	0.139673	2.720547
C	2.703794	-1.860087	2.279493
C	1.636432	-2.307008	-2.049092
C	0.083211	-2.735713	-0.233101
C	3.931664	1.105422	-0.779261
C	4.371046	-1.274947	-0.713476
H	-1.738403	2.562729	1.286571
C	-3.744712	3.005408	1.931228
C	-5.412010	1.328418	1.429268
H	-4.709168	-0.431890	0.415605
C	-3.502574	-0.767231	-3.564189
H	-2.373741	0.787011	-2.582702
C	-3.912949	-2.756876	-2.253575
H	-3.118240	-2.750238	-0.252594
C	-0.356105	-3.394151	2.109758
H	-2.677636	-0.402442	2.813544
C	-1.763596	-2.159819	3.619260
H	0.839529	0.982946	2.345418
C	1.597276	-0.045885	4.092887
H	3.129306	-2.574307	1.581412
C	2.885742	-2.044024	3.648561
H	2.420284	-1.705590	-2.495568
C	1.142550	-3.400625	-2.756295
C	-0.376552	-3.853748	-0.948173
H	3.255335	1.945094	-0.652493
C	5.259338	1.334198	-1.150765
H	4.034406	-2.295103	-0.559422
C	5.693283	-1.041597	-1.088801
H	-3.471889	3.973653	2.342027
C	-5.069328	2.569031	1.976440
H	-6.443133	0.986715	1.456201
C	-4.053866	-2.046171	-3.447168
H	-3.612424	-0.207626	-4.488949
H	-4.343760	-3.749712	-2.155707
C	-0.933033	-3.249893	3.369227
H	-2.218733	-2.025416	4.596318
H	1.161019	0.659031	4.795097
C	2.333372	-1.136062	4.557843
H	3.456120	-2.896158	4.008005
C	0.139542	-4.191157	-2.196374
H	1.545964	-3.633082	-3.737645
H	5.599102	2.352357	-1.319277
C	6.140838	0.264577	-1.308430
H	6.374497	-1.879416	-1.209707
H	-5.835175	3.193434	2.428575
H	-4.592643	-2.485767	-4.281982
H	-0.725997	-3.978125	4.147868
H	2.475872	-1.282039	5.625064
H	-0.249205	-5.053043	-2.730534
H	7.171496	0.444010	-1.601819
H	-1.173748	-4.450131	-0.515211
H	0.303254	-4.233501	1.909958
H	0.515514	-0.286099	-3.902037
C	0.286753	0.786335	-3.953105
H	0.785763	1.177603	-4.853229
H	-0.788808	0.892643	-4.108720
C	0.743642	1.522835	-2.717243
C	-0.034178	2.446807	-2.033613
B	0.660421	3.085111	-0.054357
O	-0.159304	3.964467	0.650827
O	1.929871	3.635258	-0.232102
C	0.595596	5.142778	0.986640
C	1.891675	5.017050	0.167903
H	0.786344	5.151422	2.067640
H	0.016877	6.035926	0.728412
H	2.789884	5.260813	0.744162
H	1.870436	5.645949	-0.731644
H	1.814007	1.498850	-2.521625
C	-0.978474	3.370125	-2.302236
H	-1.037530	3.818165	-3.293059
H	-1.660553	3.734724	-1.542291

IV^{Cu,α,trans}

	Value
Charge	0
Electronic Energy, BS1 (a.u.)	-2679.013871
Thermal and entropic correction, BS1 (a.u.)	0.604380
Electronic Energy, BS2 (a.u.)	-4122.971148

Molecular Geometry in Cartesian Coordinates

C	-0.834574	3.049793	-0.713005
H	-1.024855	2.964232	-1.791934
B	-3.367384	2.509708	-0.457590
O	-4.622361	2.634471	0.104596
O	-3.351445	1.620336	-1.516526
C	-5.496898	1.688427	-0.525267
C	-4.671003	1.087976	-1.689487
H	-6.400468	2.201841	-0.872355
H	-5.785906	0.928681	0.208527
H	-5.052972	1.386847	-2.673070
H	-4.625743	-0.005257	-1.652884
Cu	-0.056229	1.275768	-0.173810
P	-1.128954	-0.759787	0.116459
P	2.134812	0.565755	-0.051251
C	-2.726219	-0.685561	1.028104
C	-1.524679	-1.636730	-1.447932
C	-0.142755	-1.991884	1.083778
C	2.875033	0.074352	1.553493
C	2.256004	-0.930876	-1.130438
C	3.347809	1.747723	-0.780214
C	-2.892131	0.368326	1.939367
C	-3.756535	-1.626893	0.875810
C	-1.678337	-0.858517	-2.605239
C	-1.668066	-3.030572	-1.530927
C	1.100364	-2.474011	0.600357
C	-0.569963	-2.387480	2.362259
C	2.316512	0.630267	2.713708
C	3.951652	-0.818419	1.670774
C	2.802222	-0.802553	-2.418814
C	1.671407	-2.168400	-0.755812
C	2.830929	2.741650	-1.627047
C	4.730804	1.701003	-0.546670
H	-2.121405	1.128158	2.027510
C	-4.061050	0.474028	2.694767
C	-4.925151	-1.518588	1.630676
H	-3.649390	-2.441723	0.167140
C	-1.978393	-1.467789	-3.825259
H	-1.582366	0.220277	-2.535917
C	-1.968023	-3.635286	-2.751634
H	-1.529989	-3.642835	-0.644558
C	1.836592	-3.360155	1.401083
H	-1.518012	-2.026056	2.744495
C	0.194411	-3.242835	3.154780
H	1.470536	1.307673	2.627287
C	2.833626	0.309656	3.970084
H	4.371652	-1.278369	0.780896
C	4.468181	-1.136454	2.926339
H	3.252162	0.138310	-2.716297
C	2.782576	-1.859241	-3.327869
C	1.688328	-3.226093	-1.677971
H	1.757558	2.793330	-1.789020
C	3.682758	3.660534	-2.242689
H	5.148378	0.951124	0.116839
C	5.578687	2.627121	-1.154877
H	-4.182046	1.305926	3.381855
C	-5.077217	-0.471320	2.545250
H	-5.717727	-2.251113	1.504424
C	-2.122240	-2.854710	-3.900648
H	-2.095232	-0.857572	-4.716496
H	-2.075325	-4.715139	-2.807207
C	1.402203	-3.739041	2.669736

H	-0.161929	-3.525010	4.141456
H	2.391520	0.743177	4.862602
C	3.910557	-0.572338	4.077216
H	5.299971	-1.830725	3.007841
C	2.224315	-3.080263	-2.955786
H	3.208549	-1.725660	-4.318176
H	3.269809	4.423843	-2.896108
C	5.057668	3.604972	-2.006768
H	6.647321	2.585618	-0.962753
H	-5.989750	-0.387293	3.128763
H	-2.350426	-3.327720	-4.851837
H	2.007093	-4.410302	3.272236
H	4.310739	-0.825824	5.054882
H	2.201886	-3.913831	-3.651604
H	5.721157	4.324725	-2.478099
H	-0.110735	3.858790	-0.552393
C	-2.093014	3.234697	0.049959
C	-2.173426	3.894953	1.236965
H	-3.130574	3.939675	1.753250
C	-0.989164	4.531279	1.910924
H	-0.148184	3.822465	1.979974
H	-0.602165	5.396014	1.350406
H	-1.225461	4.876932	2.922935
H	1.249158	-4.173970	-1.382830
H	2.780495	-3.737519	1.019499

TSIII-IV’^{Cu}

	Value
Charge	0
Electronic Energy, BS1 (a.u.)	-2678.940045
Thermal and entropic correction, BS1 (a.u.)	0.608157
Electronic Energy, BS2 (a.u.)	-4122.896228

Molecular Geometry in Cartesian Coordinates

Cu	0.035947	0.992722	-0.843433
P	-1.756368	-0.267852	-0.015937
P	1.730417	-0.331858	-0.198656
C	-3.275229	0.713132	0.343304
C	-2.345102	-1.702733	-1.007044
C	-1.297071	-1.046548	1.603057
C	2.203857	-0.220345	1.585769
C	1.215811	-2.090002	-0.429309
C	3.344688	-0.157008	-1.067220
C	-3.084415	2.007199	0.859685
C	-4.581638	0.269836	0.088799
C	-1.920761	-1.795158	-2.338341
C	-3.165818	-2.710393	-0.473924
C	-0.307204	-2.061809	1.660344
C	-1.832560	-0.557293	2.804402
C	1.560531	0.727732	2.391211
C	3.170924	-1.059963	2.158963
C	1.616131	-2.778301	-1.588112
C	0.288657	-2.708995	0.444015
C	3.597192	1.075586	-1.688463
C	4.337233	-1.151688	-1.096611
H	-2.079485	2.380091	1.027147
C	-4.179352	2.823706	1.143822
C	-5.673701	1.097478	0.359208
H	-4.754876	-0.714590	-0.330915
C	-2.322498	-2.872366	-3.131794
H	-1.256900	-1.030154	-2.732439
C	-3.569458	-3.782086	-1.268898
H	-3.474663	-2.662735	0.566675
C	0.088016	-2.553311	2.912650
H	-2.591875	0.216448	2.776219
C	-1.407748	-1.042667	4.040437
H	0.798655	1.366188	1.954950
C	1.874346	0.832998	3.747534

H	3.674440	-1.807024	1.554435
C	3.486663	-0.951507	3.512338
H	2.286817	-2.297951	-2.291683
C	1.166805	-4.068032	-1.860102
C	-0.126298	-4.021680	0.167515
H	2.847621	1.858043	-1.649636
C	4.818066	1.308091	-2.325723
H	4.157595	-2.118351	-0.637390
C	5.552305	-0.917957	-1.739822
H	-4.013801	3.819587	1.545829
C	-5.477188	2.372369	0.892893
H	-6.679363	0.742395	0.151051
C	-3.148737	-3.863401	-2.600227
H	-1.981331	-2.940111	-4.161016
H	-4.204128	-4.557908	-0.849272
C	-0.440902	-2.045004	4.097059
H	-1.836582	-0.638813	4.953257
H	1.357995	1.564279	4.363106
C	2.838295	-0.004092	4.310206
H	4.236179	-1.608156	3.945655
C	0.302539	-4.702329	-0.968290
H	1.492734	-4.571949	-2.765481
H	5.000219	2.268244	-2.800293
C	5.795571	0.312801	-2.355926
H	6.308725	-1.697691	-1.760474
H	-6.329262	3.013002	1.103097
H	-3.458385	-4.702835	-3.216809
H	-0.096998	-2.428312	5.053139
H	3.082062	0.076187	5.366017
H	-0.054333	-5.708850	-1.164991
H	6.742664	0.492122	-2.857308
H	-0.830600	-4.492960	0.845995
H	0.844232	-3.331659	2.947433
C	0.022334	1.498826	-2.938731
C	-0.482200	2.472482	-2.051056
B	0.512673	2.866031	-0.137238
O	-0.199257	3.561612	0.836930
O	1.771228	3.423209	-0.326504
C	0.656014	4.579368	1.394577
C	1.854161	4.643948	0.431983
H	0.954136	4.276368	2.406229
H	0.108463	5.524889	1.459414
H	2.817014	4.689889	0.949895
H	1.783707	5.496077	-0.255922
H	1.069495	1.500191	-3.234559
C	-1.392136	3.460488	-2.063363
H	-1.772603	3.855329	-1.125428
H	-0.668377	1.035539	-3.639567
C	-1.931068	4.072747	-3.329271
H	-1.396759	3.701018	-4.208751
H	-2.997524	3.838811	-3.459473
H	-1.851528	5.168612	-3.312177

TS-IV-IV’^{Cu}

	Value
Charge	0
Electronic Energy, BS1 (a.u.)	-2679.005689
Thermal and entropic correction, BS1 (a.u.)	0.609836
Electronic Energy, BS2 (a.u.)	-4122.96349808

Molecular Geometry in Cartesian Coordinates

C	0.334773	3.507007	0.831853
H	0.875962	3.596593	1.768129
B	2.523396	2.871179	-0.209024
O	3.409475	3.011262	-1.252661
O	3.139423	2.515806	0.973914
C	4.738395	2.812195	-0.742105
C	4.537487	2.318093	0.709865

H	5.279410	3.765101	-0.786343
H	5.260513	2.081333	-1.364755
H	5.129504	2.884829	1.436078
H	4.771015	1.253977	0.820224
Cu	0.052735	1.144278	-0.497867
P	1.382818	-0.623533	0.024656
P	-2.032915	0.344003	-0.053898
C	3.008025	-0.657506	-0.844337
C	1.775446	-0.969131	1.783769
C	0.543537	-2.159477	-0.578295
C	-2.732480	-0.632320	-1.443709
C	-2.011436	-0.857758	1.359262
C	-3.351388	1.561468	0.366349
C	3.071614	-0.001402	-2.084327
C	4.150808	-1.309659	-0.357083
C	1.697504	0.103211	2.683604
C	2.124074	-2.244429	2.255376
C	-0.660625	-2.617095	0.012238
C	1.034068	-2.823155	-1.715267
C	-2.150942	-0.485951	-2.709935
C	-3.809069	-1.517665	-1.276121
C	-2.622733	-0.532145	2.581158
C	-1.277235	-2.068379	1.268805
C	-3.067473	2.518474	1.357738
C	-4.578402	1.632810	-0.308947
H	2.197261	0.534454	-2.443831
C	4.245640	-0.024742	-2.838246
C	5.330547	-1.314599	-1.103821
H	4.125384	-1.808140	0.606181
C	1.973246	-0.098209	4.037294
H	1.418267	1.086706	2.316619
C	2.398039	-2.441710	3.608613
H	2.163042	-3.084399	1.567422
C	-1.307770	-3.727770	-0.552669
H	1.956047	-2.486809	-2.175233
C	0.362710	-3.909873	-2.272797
H	-1.305925	0.188872	-2.827437
C	-2.640047	-1.214181	-3.796608
H	-4.255337	-1.646182	-0.293916
C	-4.297954	-2.240755	-2.363040
H	-3.206102	0.376912	2.664922
C	-2.504959	-1.354173	3.700626
C	-1.189625	-2.890680	2.402960
H	-2.108071	2.493085	1.864243
C	-3.996810	3.505810	1.680749
H	-4.817437	0.916660	-1.087088
C	-5.501077	2.633723	0.005845
H	4.279794	0.486825	-3.795766
C	5.377751	-0.681023	-2.348942
H	6.211325	-1.818225	-0.714996
C	2.322275	-1.368241	4.500927
H	1.909058	0.736791	4.729424
H	2.662185	-3.432538	3.968116
C	-0.816587	-4.366725	-1.688517
H	0.766589	-4.396779	-3.155892
H	-2.178940	-1.100421	-4.773628
C	-3.712917	-2.090171	-3.624452
H	-5.130119	-2.925912	-2.226647
C	-1.778874	-2.539732	3.615119
H	-2.987058	-1.067219	4.630885
H	-3.761171	4.233736	2.451894
C	-5.216755	3.568453	1.001809
H	-6.444219	2.679396	-0.531642
H	6.295467	-0.691829	-2.930444
H	2.529394	-1.524411	5.556103
H	-1.353559	-5.210878	-2.111052
H	-4.091274	-2.659310	-4.469102
H	-1.674080	-3.189932	4.478636
H	-5.936373	4.345615	1.243115
H	-0.698731	3.835212	0.826576
C	0.987031	3.127333	-0.331090
C	0.344542	2.881853	-1.610122
H	1.046216	2.821705	-2.442201
C	-0.955078	3.588722	-1.958654

H	-1.728073	3.438871	-1.197590
H	-0.824886	4.680647	-2.066236
H	-1.366654	3.214384	-2.903403
H	-0.631010	-3.818194	2.324192
H	-2.227071	-4.077060	-0.092928

IV’$^{Cu,\gamma}$

	Value
Charge	0
Electronic Energy, BS1 (a.u.)	-2679.009267
Thermal and entropic correction, BS1 (a.u.)	0.608656
Electronic Energy, BS2 (a.u.)	-4122.966315

Molecular Geometry in Cartesian Coordinates

C	0.605551	4.475600	-0.337643
H	1.114304	4.975179	0.483004
B	2.570356	2.937345	-0.485940
O	3.510501	2.391604	-1.331100
O	3.019012	3.032794	0.818678
C	4.701130	2.118567	-0.576622
C	4.321760	2.429419	0.890883
H	5.509640	2.762222	-0.943003
H	4.990478	1.074955	-0.725395
H	5.018786	3.123479	1.372151
H	4.255077	1.522584	1.502787
Cu	0.079636	0.955524	-0.897571
P	1.345141	-0.767088	-0.034338
P	-1.997319	0.324770	-0.107540
C	2.962710	-1.043205	-0.873472
C	1.746724	-0.786897	1.755226
C	0.407952	-2.334177	-0.342302
C	-2.947957	-0.756886	-1.243335
C	-1.937504	-0.616739	1.480528
C	-3.103573	1.745844	0.258309
C	3.085340	-0.549691	-2.181783
C	4.054367	-1.701120	-0.286261
C	1.784026	0.445234	2.424826
C	2.019722	-1.965786	2.466957
C	-0.784459	-2.643945	0.362280
C	0.802249	-3.168132	-1.402308
C	-2.588187	-0.741580	-2.598995
C	-3.985410	-1.600266	-0.816318
C	-2.419737	-0.033405	2.663714
C	-1.308136	-1.885225	1.548952
C	-2.478227	2.957310	0.594340
C	-4.504465	1.683120	0.222346
H	2.256201	-0.002844	-2.621097
C	4.268213	-0.730623	-2.899038
C	5.242032	-1.869031	-1.000352
H	3.985288	-2.074943	0.729868
C	2.102186	0.496446	3.783950
H	1.579620	1.363845	1.882641
C	2.335948	-1.910882	3.823878
H	1.968363	-2.926160	1.962106
C	-1.509811	-3.785581	-0.012570
H	1.712447	-2.944860	-1.947680
C	0.051211	-4.282419	-1.773499
H	-1.771742	-0.101312	-2.924956
C	-3.261411	-1.550760	-3.515883
H	-4.253205	-1.638377	0.235689
C	-4.657650	-2.406492	-1.734563
H	-2.900930	0.937555	2.623127
C	-2.297605	-0.677672	3.894042
C	-1.226310	-2.529689	2.792122
H	-1.393283	3.026130	0.589024
C	-3.242007	4.083059	0.905650
H	-5.003369	0.758687	-0.050227
C	-5.265026	2.813871	0.523889

H	4.350930	-0.338725	-3.908574
C	5.349218	-1.389386	-2.308857
H	6.083186	-2.374851	-0.534351
C	2.377438	-0.679407	4.484187
H	2.130135	1.456146	4.291953
H	2.542538	-2.828557	4.367709
C	-1.112144	-4.595489	-1.074132
H	0.381691	-4.902700	-2.601775
H	-2.972642	-1.535668	-4.562963
C	-4.296349	-2.382888	-3.084744
H	-5.458629	-3.058089	-1.396302
C	-1.703117	-1.936003	3.959411
H	-2.675215	-0.198331	4.792716
H	-2.744371	5.014545	1.159741
C	-4.636257	4.013018	0.870308
H	-6.349522	2.758490	0.486915
H	6.275217	-1.520365	-2.861781
H	2.618980	-0.638820	5.542670
H	-1.708759	-5.460019	-1.349963
H	-4.817202	-3.016454	-3.797225
H	-1.604745	-2.452639	4.909615
H	-5.231982	4.891355	1.102869
H	-0.351622	4.897017	-0.636914
C	1.155225	3.393443	-0.956017
C	0.522686	2.585645	-2.020498
H	1.282618	2.262821	-2.743929
C	-0.674461	3.215651	-2.727346
H	-1.525053	3.354647	-2.049531
H	-0.443567	4.209590	-3.151082
H	-1.025752	2.579636	-3.549004
H	-0.757053	-3.507799	2.837143
H	-2.416836	-4.021841	0.535243

IV’$^{Cu,\gamma,cis,+180}$

	Value
Charge	0
Electronic Energy, BS1 (a.u.)	-2679.001624
Thermal and entropic correction, BS1 (a.u.)	0.608728
Electronic Energy, BS2 (a.u.)	-4122.958059

Molecular Geometry in Cartesian Coordinates

C	-2.373753	-3.109784	-1.622481
H	-2.819828	-3.771233	-0.884829
B	-0.167852	-3.834763	-0.764078
O	1.203869	-4.005392	-0.832827
O	-0.708558	-4.474424	0.339091
C	1.637193	-4.663387	0.368839
C	0.338067	-5.203304	0.998099
H	2.136857	-3.927785	1.011749
H	2.353475	-5.452010	0.119229
H	0.283516	-5.028223	2.076983
H	0.201785	-6.275585	0.809995
Cu	0.099152	-0.555584	-1.258744
P	-1.728779	0.371617	-0.046803
P	1.805548	0.525816	-0.214160
C	-3.223479	0.442032	-1.115735
C	-2.323414	-0.348335	1.535167
C	-1.388146	2.136482	0.385762
C	2.120913	2.247058	-0.762103
C	1.449332	0.628923	1.596806
C	3.447039	-0.304282	-0.303791
C	-3.011574	0.637990	-2.490977
C	-4.532886	0.271392	-0.645320
C	-2.092538	-1.712733	1.760832
C	-2.985512	0.408182	2.515685
C	-0.365382	2.458493	1.313928
C	-2.073964	3.180319	-0.255676
C	1.689784	2.597505	-2.050018

C	2.762135	3.209261	0.032738
C	2.116318	-0.248166	2.469430
C	0.408912	1.445525	2.110529
C	3.443205	-1.659634	-0.666197
C	4.667612	0.327329	-0.019961
H	-1.995647	0.739411	-2.863730
C	-4.088387	0.677051	-3.376066
C	-5.608617	0.297227	-1.535160
H	-4.714670	0.106571	0.411629
C	-2.515251	-2.310417	2.949476
H	-1.595247	-2.311808	1.007551
C	-3.404959	-0.192097	3.702860
H	-3.164045	1.467201	2.353677
C	-0.098303	3.808927	1.581732
H	-2.861949	2.946279	-0.963025
C	-1.769858	4.516785	0.001661
H	1.176697	1.856446	-2.658869
C	1.906734	3.886306	-2.540450
H	3.069135	2.959029	1.044085
C	2.980689	4.495273	-0.459889
H	2.913676	-0.876486	2.087125
C	1.780011	-0.328607	3.819905
C	0.107321	1.366950	3.478929
H	2.504228	-2.151112	-0.901671
C	4.639742	-2.377168	-0.728990
H	4.691725	1.378429	0.247723
C	5.861964	-0.389908	-0.092846
H	-3.909837	0.827259	-4.437070
C	-5.390274	0.502549	-2.899122
H	-6.618699	0.155303	-1.160760
C	-3.167834	-1.552250	3.922996
H	-2.332204	-3.369323	3.108487
H	-3.915063	0.401450	4.456774
C	-0.780067	4.834661	0.929150
H	-2.314672	5.302368	-0.514272
H	1.565634	4.149253	-3.537699
C	2.553933	4.835103	-1.746728
H	3.474985	5.235320	0.163354
C	0.770967	0.485592	4.329993
H	2.311639	-1.020069	4.467538
H	4.621151	-3.425761	-1.012720
C	5.850343	-1.743467	-0.443630
H	6.803141	0.108532	0.122433
H	-6.229700	0.519597	-3.588596
H	-3.492263	-2.017057	4.850033
H	-0.535161	5.870798	1.143519
H	2.719390	5.839547	-2.126110
H	0.497284	0.436268	5.379766
H	6.783365	-2.297347	-0.500607
H	-3.063972	-2.530428	-2.234803
C	-1.025810	-2.972566	-1.743293
C	-0.385522	-1.958844	-2.627595
H	-1.156832	-1.525442	-3.283307
C	0.775855	-2.451688	-3.503985
H	1.597258	-2.863741	-2.912558
H	0.468482	-3.238539	-4.215827
H	1.188041	-1.625474	-4.096366
H	-0.683623	1.999634	3.869147
H	0.676531	4.049822	2.303290

TS_IV'$^{+180}$-IV^{cis,Cu}

	Value
Charge	0
Electronic Energy, BS1 (a.u.)	-2679.001177
Thermal and entropic correction, BS1 (a.u.)	0.609457
Electronic Energy, BS2 (a.u.)	-4122.958253

Molecular Geometry in Cartesian Coordinates

C	0.735980	3.382613	1.153436
H	1.301672	3.326452	2.077591
B	2.828664	2.694632	0.010807
O	3.768939	3.010105	-0.944544
O	3.375602	2.054216	1.104464
C	5.064435	2.650299	-0.435572
C	4.773858	1.843103	0.849144
H	5.631008	3.567479	-0.234389
H	5.600115	2.063486	-1.186721
H	5.357308	2.189447	1.708422
H	4.949444	0.771550	0.712299
Cu	0.109433	1.165737	-0.445355
P	1.258743	-0.733307	0.040288
P	-2.044986	0.507583	-0.072123
C	2.944693	-0.876309	-0.692619
C	1.479676	-1.207065	1.799314
C	0.329038	-2.139716	-0.725113
C	-2.803489	-0.261937	-1.558229
C	-2.199441	-0.812406	1.219587
C	-3.233490	1.816416	0.442985
C	3.195098	-0.110476	-1.841505
C	3.965380	-1.690544	-0.179898
C	1.458859	-0.177846	2.750775
C	1.663326	-2.533747	2.219126
C	-0.938350	-2.547823	-0.237753
C	0.822330	-2.728867	-1.901762
C	-2.187874	-0.050355	-2.799269
C	-3.955428	-1.061474	-1.487227
C	-2.841651	-0.547210	2.440320
C	-1.577881	-2.074406	1.037721
C	-2.846209	2.663221	1.497150
C	-4.458791	2.049900	-0.197496
H	2.410616	0.535309	-2.224579
C	4.435855	-0.167985	-2.477784
C	5.210567	-1.738170	-0.810009
H	3.797576	-2.276752	0.717224
C	1.628367	-0.473180	4.104645
H	1.305035	0.846810	2.423396
C	1.832756	-2.824719	3.572534
H	1.655452	-3.338869	1.489746
C	-1.642717	-3.537522	-0.942849
H	1.791823	-2.429579	-2.283472
C	0.095348	-3.693131	-2.597134
H	-1.287304	0.557584	-2.845470
C	-2.716352	-0.629482	-3.954823
H	-4.429761	-1.242423	-0.526727
C	-4.483800	-1.634998	-2.642941
H	-3.335772	0.405427	2.590892
C	-2.867045	-1.484486	3.471582
C	-1.637018	-3.011461	2.080876
H	-1.887567	2.506507	1.982040
C	-3.675244	3.702917	1.915209
H	-4.774705	1.418620	-1.020700
C	-5.280317	3.102694	0.213423
H	4.613964	0.433949	-3.364205
C	5.447346	-0.981248	-1.961166
H	5.996214	-2.367633	-0.401214
C	1.815384	-1.794122	4.516696
H	1.609457	0.329378	4.836759
H	1.969313	-3.854530	3.891186
C	-1.146702	-4.101289	-2.115540
H	0.504164	-4.123586	-3.506868
H	-2.228174	-0.466424	-4.911512
C	-3.863611	-1.420701	-3.877877
H	-5.374816	-2.253706	-2.580778
C	-2.258988	-2.725156	3.293831
H	-3.368931	-1.243848	4.404523
H	-3.362053	4.344417	2.734089
C	-4.895098	3.927398	1.271126
H	-6.224190	3.275212	-0.296417
H	6.417296	-1.021390	-2.448923
H	1.941214	-2.022538	5.571527
H	-1.728046	-4.849943	-2.645679
H	-4.273081	-1.874115	-4.776353

H	-2.268502	-3.466210	4.087716
H	-5.536814	4.744778	1.587713
H	-0.277964	3.768902	1.213664
C	1.307441	3.067296	-0.065863
C	0.546122	2.991375	-1.309117
H	-0.395103	3.553669	-1.261530
C	1.223461	3.167052	-2.660767
H	1.511648	4.213765	-2.860733
H	2.139421	2.577797	-2.750848
H	0.553233	2.862141	-3.473866
H	-1.167081	-3.978778	1.931536
H	-2.610631	-3.849326	-0.562899

IV$^{Cu,α,trans,+180}$

	Value
Charge	0
Electronic Energy, BS1 (a.u.)	-2679.009639
Thermal and entropic correction, BS1 (a.u.)	0.604036
Electronic Energy, BS2 (a.u.)	-4122.967763

Molecular Geometry in Cartesian Coordinates

C	-0.976746	1.940923	-2.402315
H	-0.834433	1.601421	-3.437574
B	-2.693587	3.105545	-0.839151
O	-3.933139	3.193204	-0.223272
O	-1.729010	3.838920	-0.165497
C	-3.832069	4.152475	0.839612
C	-2.316016	4.329806	1.049942
H	-4.344820	3.773568	1.729282
H	-4.316421	5.087425	0.529407
H	-1.944626	3.729153	1.890123
H	-2.024500	5.372798	1.208741
Cu	-0.320441	0.528518	-1.129084
P	-1.102650	-1.301505	0.012697
P	1.822866	0.605125	-0.225458
C	-2.931825	-1.485058	0.131001
C	-0.502745	-2.915329	-0.616180
C	-0.537629	-1.227258	1.777347
C	1.959799	1.634762	1.286873
C	2.527615	-1.038421	0.225076
C	3.066346	1.334427	-1.369309
C	-3.678091	-0.296134	0.145058
C	-3.607723	-2.713390	0.188192
C	-0.043055	-2.961753	-1.940221
C	-0.482171	-4.084572	0.159838
C	0.823629	-1.391314	2.145549
C	-1.464124	-0.861686	2.769116
C	1.063417	2.709340	1.406037
C	2.905162	1.409117	2.298319
C	3.587401	-1.575030	-0.526371
C	1.928532	-1.840061	1.230365
C	2.739287	1.377566	-2.732790
C	4.305751	1.837908	-0.944490
H	-3.173037	0.657893	0.063691
C	-5.069191	-0.328698	0.238314
C	-5.000988	-2.744763	0.267134
H	-3.054081	-3.645762	0.159615
C	0.421576	-4.160556	-2.484060
H	-0.043928	-2.051813	-2.535975
C	-0.021281	-5.281980	-0.386125
H	-0.811678	-4.052242	1.194389
C	1.182261	-1.206233	3.490714
H	-2.508328	-0.738459	2.504648
C	-1.079188	-0.657903	4.093120
H	0.321829	2.888528	0.630825
C	1.124665	3.550064	2.518923
H	3.591319	0.570901	2.220326
C	2.956810	2.248207	3.411821

H	4.051621	-0.972993	-1.299599
C	4.062420	-2.865585	-0.299148
C	2.444687	-3.124102	1.462718
H	1.765963	1.017023	-3.056104
C	3.643060	1.900302	-3.660121
H	4.565147	1.824444	0.109769
C	5.204482	2.365350	-1.871395
H	-5.621876	0.605980	0.242085
C	-5.733916	-1.554929	0.297545
H	-5.514892	-3.701440	0.303951
C	0.430585	-5.321241	-1.708355
H	0.780104	-4.185589	-3.509113
H	-0.006981	-6.182566	0.221456
C	0.253213	-0.833355	4.459070
H	-1.822653	-0.372377	4.831897
H	0.432090	4.383021	2.600818
C	2.067686	3.320341	3.523347
H	3.689620	2.064170	4.192734
C	3.492378	-3.644449	0.705558
H	4.879922	-3.252512	-0.900830
H	3.378565	1.931330	-4.713278
C	4.876138	2.393746	-3.230299
H	6.160411	2.756302	-1.533919
H	-6.818380	-1.585831	0.356912
H	0.794912	-6.253917	-2.129789
H	0.571000	-0.681592	5.486448
H	2.109130	3.972452	4.391440
H	3.853015	-4.650609	0.898020
H	5.577550	2.807332	-3.949605
H	-0.342947	2.821096	-2.238591
C	-2.396693	2.207814	-2.065117
C	-3.452909	1.586650	-2.657007
H	-4.457667	1.782014	-2.289174
C	-3.295747	0.538060	-3.718611
H	-2.806191	0.925983	-4.624220
H	-2.649013	-0.278894	-3.354211
H	-4.254495	0.103062	-4.018155
H	1.992257	-3.728332	2.243251
H	2.222406	-1.345370	3.769023

$IV^{Cu,\alpha,cis}$

	Value
Charge	0
Electronic Energy, BS1 (a.u.)	-2679.010075
Thermal and entropic correction, BS1 (a.u.)	0.606117
Electronic Energy, BS2 (a.u.)	-4122.965959

Molecular Geometry in Cartesian Coordinates

C	-0.935597	3.033043	-0.715145
H	-1.007240	3.027316	-1.813296
B	-3.400938	2.265971	-0.725015
O	-4.745205	2.273618	-0.404601
O	-3.116131	1.349736	-1.722799
C	-5.389779	1.202225	-1.109700
C	-4.329641	0.693671	-2.111882
H	-6.291497	1.581018	-1.602383
H	-5.680076	0.430632	-0.388763
H	-4.566925	0.964836	-3.147833
H	-4.185267	-0.389898	-2.061032
Cu	-0.095305	1.277836	-0.226004
P	-1.033957	-0.806558	0.140401
P	2.126010	0.683445	-0.071233
C	-2.706121	-0.813950	0.914578
C	-1.226155	-1.819212	-1.379683
C	-0.030760	-1.873393	1.274012
C	2.810929	0.464271	1.616653
C	2.415732	-0.920679	-0.938503

C	3.286817	1.857355	-0.890275
C	-3.028309	0.270274	1.745734
C	-3.651543	-1.834077	0.725894
C	-1.316303	-1.140111	-2.604285
C	-1.277761	-3.221848	-1.365344
C	1.266657	-2.322888	0.915387
C	-0.508433	-2.153489	2.565533
C	2.165261	1.148554	2.657428
C	3.923158	-0.339219	1.910191
C	3.026804	-0.912676	-2.204344
C	1.902209	-2.143863	-0.434886
C	2.725368	2.796187	-1.769248
C	4.676081	1.852919	-0.689005
H	-2.319959	1.085039	1.862244
C	-4.268432	0.330511	2.383593
C	-4.891848	-1.771031	1.362820
H	-3.424877	-2.674670	0.078432
C	-1.463288	-1.855431	-3.794462
H	-1.289489	-0.054844	-2.611593
C	-1.426951	-3.932827	-2.556041
H	-1.184524	-3.756868	-0.424710
C	2.005837	-3.060821	1.853050
H	-1.498298	-1.817343	2.853214
C	0.257139	-2.860508	3.491433
H	1.291984	1.757234	2.433712
C	2.632171	1.042235	3.968569
H	4.410834	-0.898331	1.116938
C	4.388400	-0.444064	3.220641
H	3.420449	0.017172	-2.600134
C	3.144279	-2.074446	-2.965429
C	2.059058	-3.305743	-1.206493
H	1.647131	2.814602	-1.905189
C	3.539921	3.706055	-2.446795
H	5.125854	1.143012	-0.002455
C	5.486737	2.767418	-1.360916
H	-4.508861	1.185055	3.008166
C	-5.201413	-0.690746	2.194946
H	-5.617565	-2.564786	1.207740
C	-1.518718	-3.250500	-3.772335
H	-1.530589	-1.321345	-4.738146
H	-1.463631	-5.018613	-2.535580
C	1.520803	-3.323423	3.132434
H	-0.140952	-3.054024	4.483474
H	2.123657	1.574102	4.767667
C	3.744672	0.247170	4.250858
H	5.248417	-1.070879	3.439808
C	2.661775	-3.280789	-2.462454
H	3.617692	-2.033060	-3.942234
H	3.093533	4.428773	-3.123936
C	4.920687	3.692640	-2.243329
H	6.560516	2.759553	-1.195077
H	-6.169637	-0.641345	2.685136
H	-1.628698	-3.805775	-4.699727
H	2.128835	-3.879862	3.839667
H	4.105953	0.160030	5.271721
H	2.747499	-4.195963	-3.040885
H	5.555074	4.404578	-2.764089
H	-0.282996	3.871380	-0.427419
C	-2.285736	3.149744	-0.083240
C	-2.497621	3.911949	1.022173
H	-1.645283	4.489719	1.396118
C	-3.743591	4.058455	1.851780
H	-4.562614	3.432972	1.496236
H	-3.547015	3.804533	2.905347
H	-4.096422	5.101142	1.859758
H	1.676216	-4.241785	-0.811623
H	2.992253	-3.414187	1.568115

IV’$^{Cu,\gamma,cis}$

	Value
Charge	0

	Value
Electronic Energy, BS1 (a.u.)	-2679.006036
Thermal and entropic correction, BS1 (a.u.)	0.605555
Electronic Energy, BS2 (a.u.)	-4122.963170

Molecular Geometry in Cartesian Coordinates

C	-1.238452	1.712892	-2.451959
H	-1.158062	1.247293	-3.447204
B	-2.931224	2.783705	-0.822711
O	-4.123323	3.404840	-0.496253
O	-1.931077	3.092302	0.094554
C	-3.896795	4.268224	0.628378
C	-2.516911	3.844208	1.167588
H	-4.699835	4.139687	1.361036
H	-3.903420	5.310284	0.285467
H	-2.599457	3.197876	2.051544
H	-1.873869	4.693559	1.417432
Cu	-0.241918	0.637943	-1.084333
P	-0.926129	-1.267176	0.050679
P	1.876600	0.774767	-0.227959
C	-2.712456	-1.253142	0.485993
C	-0.668503	-2.804173	-0.916974
C	-0.081760	-1.610219	1.661366
C	1.989846	1.350045	1.509109
C	2.648029	-0.901061	-0.302188
C	3.050951	1.862469	-1.139461
C	-3.243923	-0.017521	0.890283
C	-3.559253	-2.366045	0.401796
C	-0.522592	-2.675913	-2.306334
C	-0.587609	-4.076966	-0.330804
C	1.315569	-1.845617	1.714883
C	-0.804911	-1.561292	2.864704
C	0.952064	2.183237	1.957090
C	3.033620	1.017430	2.384505
C	3.550566	-1.170956	-1.346636
C	2.216192	-1.975315	0.519053
C	2.642371	2.319486	-2.401329
C	4.307423	2.245111	-0.644224
H	-2.592898	0.850559	0.926496
C	-4.592915	0.101539	1.218698
C	-4.913839	-2.242802	0.721589
H	-3.168654	-3.325801	0.079227
C	-0.303187	-3.804348	-3.098290
H	-0.573284	-1.687533	-2.757314
C	-0.369238	-5.202789	-1.124665
H	-0.682999	-4.183547	0.745927
C	1.915124	-2.057721	2.965935
H	-1.874441	-1.385827	2.838278
C	-0.182669	-1.742801	4.098945
H	0.134764	2.428174	1.282073
C	0.968612	2.685196	3.259401
H	3.824582	0.350014	2.055049
C	3.044929	1.519034	3.685992
H	3.887978	-0.358927	-1.981643
C	4.024024	-2.458723	-1.590405
C	2.723467	-3.260285	0.267153
H	1.660103	2.036384	-2.771659
C	3.481650	3.136671	-3.162313
H	4.631947	1.910019	0.335913
C	5.141253	3.065480	-1.403166
H	-4.993133	1.067248	1.512594
C	-5.432044	-1.012922	1.132437
H	-5.564589	-3.109633	0.646264
C	-0.225446	-5.067554	-2.508611
H	-0.186453	-3.694763	-4.172717
H	-0.305831	-6.184693	-0.663741
C	1.185447	-1.999259	4.151405
H	-0.770937	-1.695146	5.010999
H	0.162331	3.329222	3.599930
C	2.013906	2.354389	4.124486

H	3.854065	1.252407	4.360340
C	3.607645	-3.512455	-0.779280
H	4.716813	-2.631804	-2.409020
H	3.155059	3.485923	-4.137783
C	4.730887	3.509801	-2.664212
H	6.110975	3.359990	-1.011279
H	-6.487438	-0.919598	1.372281
H	-0.048952	-5.945209	-3.124225
H	1.684795	-2.149302	5.104183
H	2.023084	2.740226	5.140115
H	3.964184	-4.522838	-0.956691
H	5.382543	4.150013	-3.252460
H	-0.747408	2.695836	-2.508302
C	-2.672437	1.847081	-2.038844
C	-3.666027	1.127445	-2.619024
H	-3.382850	0.467310	-3.445914
C	-5.116315	1.037476	-2.235385
H	-5.380470	1.712426	-1.420460
H	-5.767141	1.272146	-3.090954
H	-5.376120	0.015058	-1.923600
H	2.394388	-4.076923	0.902323
H	2.982749	-2.254014	2.999395

I^{Pd}

	Value
Charge	0
Electronic Energy, BS1 (a.u.)	-2851.673920
Thermal and entropic correction, BS1 (a.u.)	0.655385
Electronic Energy, BS2 (a.u.)	-2852.49710748

Molecular Geometry in Cartesian Coordinates

Pd	-0.508030	0.482560	-0.263355
P	1.843347	0.521743	-0.414143
P	-0.744864	-1.777409	0.293476
C	2.425307	1.781244	-1.631447
C	2.796966	0.909192	1.109161
C	2.595878	-1.068298	-0.990034
C	-0.494652	-2.972600	-1.089885
C	0.559715	-2.191582	1.537845
C	-2.316958	-2.398630	1.047675
C	1.689744	1.916602	-2.822440
C	3.484770	2.665549	-1.387622
C	2.148553	1.602463	2.140250
C	4.144048	0.545171	1.266057
C	2.491870	-2.239648	-0.197223
C	3.209117	-1.154459	-2.249453
C	-0.701539	-2.511494	-2.397533
C	-0.145156	-4.316443	-0.889706
C	0.196234	-2.235043	2.895097
C	1.934592	-2.273340	1.199151
C	-3.428474	-1.546016	1.033612
C	-2.463305	-3.693418	1.576050
H	0.848625	1.253523	-3.007962
C	2.014771	2.902710	-3.752497
C	3.798423	3.665147	-2.312543
H	4.056906	2.590746	-0.469416
C	2.836216	1.923942	3.312014
H	1.109288	1.891823	2.026693
C	4.827729	0.866276	2.438301
H	4.653826	0.003327	0.474622
C	3.028961	-3.436768	-0.692150
H	3.303065	-0.264013	-2.861071
C	3.709887	-2.362353	-2.734498
H	-0.955734	-1.466809	-2.558553
C	-0.569056	-3.378243	-3.484767
H	0.050712	-4.682267	0.113369
C	-0.013857	-5.181619	-1.974465
H	-0.850089	-2.155756	3.169594

C	1.147450	-2.377275	3.903617
C	2.872769	-2.443707	2.229999
H	-3.337889	-0.543511	0.637429
C	-4.665972	-1.975132	1.520650
H	-1.614197	-4.367973	1.612040
C	-3.694215	-4.118126	2.072967
H	1.437611	2.992295	-4.668602
C	3.067793	3.786307	-3.495255
H	4.614321	4.351556	-2.103520
C	4.173296	1.554282	3.464602
H	2.322219	2.463677	4.101884
H	5.868902	0.576994	2.552714
C	3.622369	-3.510656	-1.950920
H	4.173993	-2.397977	-3.716057
H	-0.728756	-3.007152	-4.493361
C	-0.227402	-4.714685	-3.274668
H	0.262808	-6.218938	-1.806842
C	2.495626	-2.489271	3.569923
H	0.831143	-2.402824	4.942512
H	-5.510301	-1.293937	1.479959
C	-4.800339	-3.261088	2.042090
H	-3.792802	-5.120054	2.482031
H	3.311471	4.566923	-4.210310
H	4.705971	1.800284	4.379167
H	4.010528	-4.458264	-2.312720
H	-0.121193	-5.389668	-4.119321
H	3.250373	-2.602773	4.342520
H	-5.759558	-3.599597	2.424322
H	3.922759	-2.514522	1.963595
H	2.958668	-4.328101	-0.075995
H	-8.142419	3.395048	-0.523617
C	-7.447480	2.613726	-0.213391
H	-7.147894	2.766158	0.827475
H	-7.917049	1.630537	-0.310323
O	-6.317291	2.732014	-1.094212
C	-5.333169	1.848895	-0.864229
O	-5.351990	0.993335	0.002919
O	-4.357392	2.072868	-1.743273
C	-3.174707	1.205201	-1.586875
H	-3.538297	0.181108	-1.480764
H	-2.651712	1.306172	-2.539999
C	-2.319082	1.636737	-0.433817
C	-1.209204	2.498960	-0.626002
C	-0.624592	3.391018	0.398418
C	-1.073441	3.431045	1.734087
H	-1.913936	2.814985	2.038198
C	-0.442959	4.239580	2.679427
H	-0.808924	4.248853	3.703093
C	0.652349	5.031113	2.321675
H	1.146365	5.654028	3.061820
C	1.099456	5.013509	0.997248
H	1.948934	5.622878	0.699938
C	0.466477	4.212632	0.049976
H	0.833756	4.195258	-0.972284
H	-0.949900	2.767828	-1.650569
H	-2.844932	1.643160	0.518360

TS-I-II^{Pd}

	Value
Charge	0
Electronic Energy, BS1 (a.u.)	-2851.645340
Thermal and entropic correction, BS1 (a.u.)	0.652651
Electronic Energy, BS2 (a.u.)	-2852.474556

Molecular Geometry in Cartesian Coordinates

Pd	0.449922	-0.468888	-0.420597
P	-1.891631	-0.312117	-0.301628
P	0.932744	1.751977	0.186199

C	-2.689580	-1.422321	-1.532578
C	-2.676090	-0.717747	1.305246
C	-2.554812	1.369805	-0.682296
C	0.632763	3.044333	-1.079884
C	-0.152183	2.174371	1.621597
C	2.649813	2.081432	0.776228
C	-2.100222	-1.507295	-2.806595
C	-3.788922	-2.237963	-1.235750
C	-1.940714	-1.446202	2.248926
C	-3.984763	-0.311320	1.613017
C	-2.287569	2.459609	0.182511
C	-3.297929	1.593464	-1.851568
C	0.722598	2.673968	-2.429463
C	0.345298	4.378113	-0.752493
C	0.411457	2.153260	2.907939
C	-1.552221	2.354977	1.488631
C	3.433866	0.990877	1.182098
C	3.213134	3.368461	0.800663
H	-1.232708	-0.894949	-3.039950
C	-2.608062	-2.381182	-3.766309
C	-4.289022	-3.121689	-2.195506
H	-4.245665	-2.203142	-0.253001
C	-2.505532	-1.766652	3.484425
H	-0.931038	-1.765900	2.015607
C	-4.545516	-0.632517	2.848345
H	-4.558185	0.264207	0.892171
C	-2.805425	3.720583	-0.147371
H	-3.516515	0.765617	-2.516371
C	-3.776067	2.862752	-2.175486
H	0.930204	1.638978	-2.688432
C	0.538808	3.623813	-3.435611
H	0.246647	4.670551	0.288760
C	0.163691	5.325855	-1.758936
H	1.482017	2.019087	3.019464
C	-0.375143	2.300354	4.049309
C	-2.320920	2.523229	2.650801
H	3.032104	-0.014325	1.159441
C	4.759273	1.171060	1.580744
H	2.635640	4.228149	0.478930
C	4.530833	3.550420	1.219517
H	-2.142505	-2.436742	-4.746058
C	-3.702262	-3.196356	-3.459343
H	-5.134762	-3.757210	-1.948829
C	-3.805378	-1.358685	3.786503
H	-1.926923	-2.334248	4.207232
H	-5.557420	-0.312949	3.081108
C	-3.533882	3.931903	-1.316244
H	-4.343965	3.007005	-3.089970
H	0.609617	3.326275	-4.477862
C	0.260783	4.950399	-3.101386
H	-0.061448	6.355466	-1.495679
C	-1.750223	2.485246	3.921172
H	0.089774	2.274157	5.030494
H	5.352762	0.295374	1.820126
C	5.307881	2.453168	1.603990
H	4.956198	4.550180	1.230066
H	-4.089643	-3.889219	-4.200687
H	-4.242791	-1.604068	4.750162
H	-3.905545	4.924869	-1.551137
H	0.114677	5.689254	-3.884133
H	-2.377264	2.599920	4.800310
H	6.341812	2.598887	1.904210
H	-3.391735	2.665747	2.544687
H	-2.610680	4.550598	0.524947
H	8.138997	-3.523323	-0.250256
C	7.247372	-2.969375	0.060521
H	6.752755	-3.503714	0.880561
H	7.544174	-1.981735	0.434533
O	6.417166	-2.874731	-1.090172
C	5.210753	-2.205275	-0.895901
O	4.956223	-1.753757	0.239651
O	4.523464	-2.156024	-1.960213
C	2.657695	-1.084522	-1.399783
H	3.406010	-0.409637	-1.022491

H	2.347024	-0.971486	-2.433821
C	2.018648	-1.994002	-0.546811
C	0.777174	-2.599668	-0.879445
C	0.088420	-3.597659	-0.037014
C	0.494322	-3.899600	1.277907
H	1.368560	-3.415555	1.701585
C	-0.217974	-4.811431	2.053688
H	0.115767	-5.029349	3.064714
C	-1.355346	-5.442702	1.540982
H	-1.912298	-6.148494	2.150520
C	-1.762328	-5.162015	0.234977
H	-2.641250	-5.647576	-0.180017
C	-1.046545	-4.255958	-0.545606
H	-1.376170	-4.039474	-1.557275
H	0.499697	-2.627810	-1.933216
H	2.506423	-2.182253	0.404177

II^Pd

	Value
Charge	0
Electronic Energy, BS1 (a.u.)	-2851.677565
Thermal and entropic correction, BS1 (a.u.)	0.656497
Electronic Energy, BS2 (a.u.)	-2852.502188

Molecular Geometry in Cartesian Coordinates

Pd	0.035306	0.387782	-1.195349
P	1.047430	-1.190119	0.229062
P	-2.129100	0.123910	-0.359729
C	2.464966	-2.001656	-0.621710
C	1.667664	-0.651964	1.867522
C	-0.073657	-2.595609	0.685904
C	-3.028166	-1.394410	-0.854383
C	-2.010833	0.078028	1.482457
C	-3.290435	1.498888	-0.745541
C	2.261900	-2.462869	-1.935310
C	3.735111	-2.131155	-0.047564
C	1.650658	0.706584	2.209146
C	2.102081	-1.606676	2.804261
C	-1.181138	-2.369676	1.539417
C	0.155361	-3.891622	0.200141
C	-2.650800	-2.044735	-2.037007
C	-4.102021	-1.899955	-0.104871
C	-2.290618	1.250845	2.200263
C	-1.503329	-1.049090	2.174386
C	-2.746787	2.787538	-0.867236
C	-4.671058	1.315113	-0.919114
H	1.287373	-2.345006	-2.402202
C	3.297027	-3.070304	-2.643269
C	4.775102	-2.729007	-0.764620
H	3.926202	-1.752742	0.949715
C	2.080925	1.098418	3.480797
H	1.270720	1.455667	1.518114
C	2.535363	-1.203978	4.065386
H	2.090411	-2.662888	2.550393
C	-1.997867	-3.455434	1.886184
H	1.007618	-4.086805	-0.439362
C	-0.686114	-4.952260	0.535741
H	-1.810947	-1.664057	-2.611783
C	-3.338925	-3.180412	-2.467740
H	-4.388888	-1.415254	0.823706
C	-4.790993	-3.031306	-0.539329
H	-2.659772	2.125299	1.678681
C	-2.086276	1.321346	3.576950
C	-1.326370	-0.962714	3.563541
H	-1.682151	2.949034	-0.751408
C	-3.572971	3.877886	-1.139316
H	-5.112315	0.327393	-0.847881
C	-5.490949	2.409577	-1.200966

H	3.121728	-3.428927	-3.653594
C	4.559389	-3.206007	-2.056780
H	5.757571	-2.812179	-0.309240
C	2.525382	0.152984	4.404558
H	2.053584	2.150160	3.748496
H	2.872408	-1.945848	4.783967
C	-1.766907	-4.735252	1.386542
H	-0.485097	-5.943639	0.140218
H	-3.035716	-3.680780	-3.382948
C	-4.410197	-3.673245	-1.720890
H	-5.619262	-3.416741	0.048269
C	-1.602830	0.209414	4.263475
H	-2.302679	2.245647	4.104352
H	-3.122618	4.861865	-1.227273
C	-4.946556	3.692067	-1.307359
H	-6.557678	2.256421	-1.339758
H	5.370053	-3.671068	-2.610197
H	2.854943	0.467434	5.391144
H	-2.427082	-5.552468	1.661539
H	-4.944316	-4.558319	-2.054463
H	-1.434346	0.251809	5.335422
H	-5.590496	4.539041	-1.527922
H	-0.936993	-1.828670	4.089575
H	-2.838715	-3.277017	2.549436
H	1.281979	5.403810	2.906195
C	1.081329	4.530967	2.275933
H	2.001720	3.939475	2.185220
H	0.319199	3.903243	2.751801
O	0.651873	5.034064	1.019046
C	0.251294	4.077892	0.079919
O	0.271081	2.868645	0.453188
O	-0.082616	4.551083	-1.021446
C	-0.425547	1.508900	-3.036906
H	-1.241531	2.222878	-3.041868
H	-0.525031	0.669080	-3.724566
C	0.841603	1.916485	-2.580654
C	1.864656	0.954768	-2.418102
C	3.164944	1.221076	-1.774387
C	3.298361	2.132547	-0.709747
H	2.414919	2.626603	-0.319627
C	4.541238	2.347370	-0.120250
H	4.626847	3.047665	0.706149
C	5.671631	1.658862	-0.573864
H	6.638259	1.827716	-0.107287
C	5.546812	0.744954	-1.621604
H	6.414341	0.193576	-1.973505
C	4.303140	0.525287	-2.213162
H	4.207139	-0.202723	-3.013566
H	0.916756	2.901665	-2.122245
H	1.852549	0.093736	-3.086997

TS-II-III^{Pd}

	Value
Charge	0
Electronic Energy, BS1 (a.u.)	-2851.656726
Thermal and entropic correction, BS1 (a.u.)	0.651196
Electronic Energy, BS2 (a.u.)	-2852.484867

Molecular Geometry in Cartesian Coordinates

Pd	-0.056652	0.117402	-1.143022
P	0.224465	-1.654036	0.442744
P	1.098251	1.645233	0.095063
C	-1.348917	-2.601044	0.554125
C	1.521507	-2.899587	0.088938
C	0.523181	-1.163060	2.198970
C	0.344493	2.268834	1.644763
C	2.707244	0.850817	0.576584
C	1.606799	3.158811	-0.831649

C	-2.556784	-1.895461	0.422930
C	-1.383967	-3.983242	0.787083
C	1.888479	-3.080440	-1.253494
C	2.147290	-3.660957	1.087537
C	1.695872	-0.460377	2.559785
C	-0.425119	-1.454940	3.192280
C	-1.040748	2.132102	1.807665
C	1.099530	2.892364	2.650448
C	3.848458	1.118297	-0.197556
C	2.798656	-0.120823	1.604683
C	1.949079	3.008331	-2.188341
C	1.651176	4.436373	-0.256920
H	-2.544901	-0.829244	0.212589
C	-3.777812	-2.559617	0.543299
C	-2.607616	-4.646959	0.890864
H	-0.459663	-4.543976	0.881456
C	2.867818	-4.015096	-1.591116
H	1.400458	-2.489878	-2.022550
C	3.129909	-4.590508	0.744755
H	1.872847	-3.521978	2.129020
C	1.888187	-0.105402	3.902912
H	-1.327709	-1.994671	2.932186
C	-0.229135	-1.068886	4.517707
H	-1.624870	1.634982	1.039737
C	-1.666040	2.623788	2.954899
H	2.177027	2.980019	2.543257
C	0.472029	3.385110	3.793733
H	3.805741	1.863487	-0.982006
C	5.050451	0.445343	0.014502
C	4.022620	-0.776815	1.810199
H	1.878218	2.031555	-2.657180
C	2.357858	4.108769	-2.939889
H	1.370313	4.584319	0.779455
C	2.047366	5.538541	-1.018766
H	-4.701061	-1.998583	0.439259
C	-3.804746	-3.936859	0.774699
H	-2.623793	-5.719296	1.064636
C	3.491710	-4.767701	-0.593634
H	3.143418	-4.148998	-2.633308
H	3.613991	-5.174912	1.522370
C	0.936264	-0.396022	4.877744
H	-0.983667	-1.303145	5.262923
H	-2.739629	2.507538	3.072398
C	-0.911410	3.252637	3.946483
H	1.062753	3.863909	4.569534
C	5.139563	-0.512043	1.022017
H	5.910333	0.675348	-0.607742
H	2.621445	3.974670	-3.985018
C	2.407604	5.377771	-2.356941
H	2.071476	6.523732	-0.561520
H	-4.755451	-4.455969	0.858340
H	4.259394	-5.489997	-0.856891
H	1.104231	-0.093483	5.907172
H	-1.397026	3.632656	4.840868
H	6.067819	-1.047671	1.197349
H	2.714711	6.237233	-2.946105
H	4.082535	-1.518827	2.600358
H	2.795574	0.426225	4.173417
H	-2.327476	-2.074503	-2.459461
C	-1.288350	-2.279924	-2.771661
H	-0.918267	-3.090085	-2.118317
H	-1.327068	-2.689209	-3.793709
O	-0.487606	-1.136133	-2.764764
C	1.145424	-1.069374	-4.285236
O	0.959374	-2.113282	-4.800528
O	1.596116	-0.015326	-4.004442
C	-0.948533	1.589471	-2.378154
H	-0.578439	1.321016	-3.366710
H	-0.709113	2.616540	-2.103454
C	-2.325313	1.199959	-2.097556
C	-3.149286	1.840069	-1.224302
C	-4.480126	1.419702	-0.795073
C	-5.133191	0.269473	-1.292334
H	-4.646305	-0.344860	-2.043255

C	-6.395401	-0.095011	-0.831179
H	-6.874866	-0.983051	-1.235475
C	-7.047106	0.667954	0.145011
H	-8.030818	0.377836	0.502863
C	-6.417265	1.808200	0.649544
H	-6.910322	2.413046	1.406353
C	-5.156500	2.178106	0.184720
H	-4.677126	3.070583	0.581310
H	-2.666791	0.291036	-2.588683
H	-2.776978	2.752029	-0.756135

III^{Pd}

	Value
Charge	0
Electronic Energy, BS1 (a.u.)	-2851.665884
Thermal and entropic correction, BS1 (a.u.)	0.655530
Electronic Energy, BS2 (a.u.)	-2852.492131

Molecular Geometry in Cartesian Coordinates

Pd	-0.195852	0.409966	-0.887178
P	-0.276603	-1.546911	0.451199
P	1.757139	1.196452	-0.066123
C	-2.053371	-1.963857	0.666911
C	0.556187	-3.071381	-0.118865
C	0.373496	-1.275935	2.157245
C	1.727327	2.066004	1.546249
C	2.930126	-0.212643	0.184151
C	2.608155	2.322407	-1.246456
C	-2.936226	-0.920007	1.000540
C	-2.572486	-3.240319	0.413479
C	1.036113	-3.118904	-1.435666
C	0.745623	-4.168866	0.737173
C	1.753243	-1.021367	2.344995
C	-0.483734	-1.237495	3.266797
C	0.498932	2.486639	2.071432
C	2.906267	2.305463	2.270943
C	3.945126	-0.433504	-0.760034
C	2.773751	-1.132847	1.250644
C	2.528778	1.999444	-2.614587
C	3.295041	3.477580	-0.848436
H	-2.555110	0.085122	1.155255
C	-4.306501	-1.152276	1.100554
C	-3.948861	-3.466735	0.498850
H	-1.911268	-4.054747	0.138044
C	1.697062	-4.263563	-1.888522
H	0.912086	-2.264654	-2.097019
C	1.404731	-5.307200	0.276365
H	0.388148	-4.127019	1.762416
C	2.220333	-0.741447	3.636461
H	-1.539501	-1.447103	3.139477
C	-0.002269	-0.938267	4.541465
H	-0.414377	2.274776	1.524724
C	0.448405	3.148634	3.299452
H	3.860473	1.958650	1.885022
C	2.853423	2.971992	3.494042
H	4.077123	0.258822	-1.582325
C	4.794323	-1.534915	-0.668516
C	3.651894	-2.223754	1.333201
H	1.965008	1.126903	-2.938027
C	3.156862	2.810822	-3.558920
H	3.349108	3.754620	0.198295
C	3.908417	4.291859	-1.803514
H	-4.971938	-0.331947	1.349368
C	-4.815593	-2.429021	0.844804
H	-4.341132	-4.458148	0.290589
C	1.881701	-5.354279	-1.037775
H	2.071200	-4.294385	-2.907665
H	1.550750	-6.153996	0.941270

C	1.355150	-0.685743	4.727987
H	-0.688612	-0.911543	5.382953
H	-0.509377	3.464826	3.702495
C	1.624502	3.394805	4.009418
H	3.769276	3.153949	4.049023
C	4.649214	-2.435454	0.383970
H	5.564389	-1.681846	-1.419894
H	3.092486	2.550279	-4.611388
C	3.846456	3.958042	-3.157000
H	4.432968	5.188448	-1.485338
H	-5.885152	-2.609313	0.904356
H	2.399939	-6.240342	-1.394078
H	1.742559	-0.450304	5.714799
H	1.585078	3.907873	4.966018
H	5.301480	-3.299671	0.466404
H	4.324665	4.593741	-3.896886
H	3.525841	-2.926265	2.151094
H	3.280237	-0.551349	3.774576
H	-3.331746	-0.756485	-1.645751
C	-2.852938	-1.042642	-2.582232
H	-2.942631	-2.124384	-2.723497
H	-3.340573	-0.544556	-3.426229
O	-1.479513	-0.657276	-2.491652
C	-0.662307	-0.845366	-3.719631
O	-1.279168	-1.267821	-4.692058
O	0.524457	-0.531396	-3.501465
C	-0.627012	2.280829	-1.805024
H	-0.300911	2.177883	-2.839273
H	-0.137566	3.114975	-1.300570
C	-2.062350	2.173131	-1.584125
C	-2.713464	2.587852	-0.461889
C	-4.109029	2.337793	-0.114354
C	-5.032428	1.727045	-0.991288
H	-4.723785	1.449369	-1.993550
C	-6.339095	1.470083	-0.589360
H	-7.028469	0.997843	-1.283845
C	-6.768831	1.811410	0.698291
H	-7.790008	1.608390	1.007492
C	-5.870891	2.418978	1.579673
H	-6.190239	2.691226	2.582056
C	-4.562027	2.679968	1.176916
H	-3.869540	3.151291	1.870665
H	-2.619589	1.629851	-2.341972
H	-2.133697	3.121679	0.291519

IV^{Pd}

	Value
Charge	0
Electronic Energy, BS1 (a.u.)	-2663.060872
Thermal and entropic correction, BS1 (a.u.)	0.643949
Electronic Energy, BS2 (a.u.)	-2663.802053

Molecular Geometry in Cartesian Coordinates

Pd	0.063190	-0.171318	-1.409665
P	-0.372273	1.723523	-0.013203
P	-1.073649	-1.627993	-0.059704
C	1.155081	2.740586	0.115091
C	-1.685592	2.865284	-0.589383
C	-0.781896	1.410254	1.762540
C	-0.375701	-2.021808	1.589399
C	-2.754348	-0.892621	0.244705
C	-1.437338	-3.274742	-0.814663
C	2.391034	2.073693	0.149059
C	1.126991	4.138860	0.212588
C	-1.974003	2.871626	-1.963912
C	-2.401903	3.705714	0.276419
C	-1.947992	0.694637	2.118381
C	0.074869	1.857289	2.781512

C	0.991410	-1.795410	1.798926
C	-1.152278	-2.562845	2.625934
C	-3.832675	-1.323499	-0.545823
C	-2.961627	0.188263	1.138670
C	-1.731697	-3.315509	-2.189890
C	-1.412116	-4.476226	-0.093298
H	2.429449	0.992795	0.042425
C	3.576351	2.793913	0.300322
C	2.316277	4.857287	0.346674
H	0.180232	4.668343	0.179373
C	-2.967733	3.717221	-2.460409
H	-1.417401	2.214499	-2.630146
C	-3.396321	4.544871	-0.227181
H	-2.188651	3.697956	1.341290
C	-2.226952	0.483030	3.476894
H	0.971604	2.408718	2.526656
C	-0.204746	1.612654	4.125289
H	1.592403	-1.364321	1.004723
C	1.577875	-2.115865	3.024651
H	-2.217027	-2.720924	2.479397
C	-0.563405	-2.884704	3.847975
H	-3.705020	-2.158035	-1.223911
C	-5.080145	-0.704681	-0.484123
C	-4.228479	0.791095	1.191953
H	-1.714652	-2.396365	-2.768698
C	-2.024838	-4.524335	-2.818408
H	-1.167753	-4.478544	0.962638
C	-1.691879	-5.688355	-0.729607
H	4.521983	2.261789	0.325872
C	3.540768	4.186880	0.396619
H	2.284464	5.941167	0.414546
C	-3.680530	4.550643	-1.595490
H	-3.188221	3.718885	-3.524225
H	-3.950144	5.191459	0.447969
C	-1.365342	0.927483	4.477117
H	0.481157	1.965935	4.889726
H	2.637490	-1.931619	3.176334
C	0.802365	-2.662650	4.048174
H	-1.171069	-3.300080	4.646896
C	-5.279980	0.365232	0.384655
H	-5.888459	-1.063657	-1.114492
H	-2.252420	-4.535903	-3.880362
C	-2.004744	-5.715914	-2.088697
H	-1.663013	-6.611435	-0.157505
H	4.464550	4.748546	0.503967
H	-4.457833	5.202212	-1.985188
H	-1.598569	0.733668	5.519989
H	1.257502	-2.909235	5.003292
H	-6.243773	0.862506	0.440098
H	-2.221041	-6.660138	-2.580262
H	-4.375576	1.618728	1.878896
H	-3.129212	-0.060176	3.741643
H	2.337670	1.946457	-2.657107
C	1.338116	2.088716	-3.111816
H	0.896257	2.968297	-2.603821
H	1.512012	2.390996	-4.161024
O	0.538299	0.951229	-3.087010
C	1.086012	-1.694058	-2.474667
H	0.807885	-1.495704	-3.510049
H	0.846224	-2.709270	-2.158718
C	2.424821	-1.252352	-2.101584
C	3.186888	-1.819390	-1.126938
C	4.461681	-1.334111	-0.606724
C	5.120840	-0.185447	-1.100094
H	4.682861	0.377614	-1.918106
C	6.325701	0.243600	-0.549782
H	6.811029	1.128382	-0.954199
C	6.912557	-0.449757	0.515218
H	7.851067	-0.108833	0.942724
C	6.275916	-1.586986	1.017648
H	6.718636	-2.139076	1.842759
C	5.073415	-2.022269	0.463627
H	4.588819	-2.911505	0.860844

H	2.785775	-0.363217	-2.614700
H	2.798200	-2.714835	-0.640583

TS-IV_IV'^{Pd}

	Value
Charge	0
Electronic Energy, BS1 (a.u.)	-2663.038440
Thermal and entropic correction, BS1 (a.u.)	0.642143
Electronic Energy, BS2 (a.u.)	-2663.781633

Molecular Geometry in Cartesian Coordinates

Pd	-0.413918	0.167483	-0.960498
P	0.891613	-1.647297	-0.051966
P	0.873834	1.787812	-0.007004
C	-0.300368	-2.896962	0.585795
C	1.978962	-2.563161	-1.206782
C	1.948153	-1.314032	1.426780
C	0.839808	1.982074	1.814153
C	2.634384	1.414767	-0.480090
C	0.595207	3.497445	-0.647850
C	-1.465427	-2.432213	1.217830
C	-0.097445	-4.277609	0.457657
C	1.602567	-2.569600	-2.560429
C	3.142377	-3.234067	-0.801528
C	2.999035	-0.371743	1.356960
C	1.710695	-1.974997	2.642789
C	-0.313838	1.571369	2.496799
C	1.896956	2.560010	2.532963
C	3.184488	2.090804	-1.581999
C	3.387666	0.370580	0.115008
C	0.258030	3.647237	-2.005069
C	0.698582	4.642159	0.154688
H	-1.636608	-1.362965	1.309380
C	-2.407756	-3.331256	1.715933
C	-1.047935	-5.176199	0.947209
H	0.795017	-4.652552	-0.033149
C	2.388141	-3.248323	-3.493555
H	0.698853	-2.041880	-2.864359
C	3.925017	-3.904550	-1.742624
H	3.437563	-3.228902	0.243557
C	3.789861	-0.150868	2.494657
H	0.911982	-2.704049	2.710153
C	2.488386	-1.720609	3.771660
H	-1.130365	1.117669	1.942087
C	-0.410390	1.738483	3.879021
H	2.799436	2.868403	2.013421
C	1.797803	2.726267	3.913814
H	2.637280	2.902550	-2.044518
C	4.428834	1.747428	-2.108098
C	4.641580	0.045196	-0.426705
H	0.144770	2.765915	-2.629500
C	0.056717	4.914098	-2.550656
H	0.939225	4.554713	1.208080
C	0.483075	5.909392	-0.392119
H	-3.306036	-2.957510	2.199393
C	-2.202932	-4.706513	1.575989
H	-0.885203	-6.244488	0.834406
C	3.548891	-3.911966	-3.088510
H	2.095044	-3.252123	-4.539741
H	4.827230	-4.420409	-1.425383
C	3.538469	-0.808035	3.696845
H	2.276514	-2.243277	4.699893
H	-1.306983	1.415079	4.399876
C	0.644754	2.315402	4.588107
H	2.622931	3.168999	4.464310
C	5.160973	0.711980	-1.533377
H	4.817762	2.291627	-2.963720
H	-0.200396	5.011350	-3.601443

C	0.167883	6.049505	-1.744039
H	0.561810	6.786794	0.243636
H	-2.942086	-5.408333	1.951816
H	4.161086	-4.432869	-3.819668
H	4.156225	-0.603559	4.566387
H	0.571655	2.441614	5.664559
H	6.128590	0.426576	-1.935287
H	0.000873	7.036504	-2.165898
H	5.208370	-0.756914	0.035752
H	4.602482	0.566589	2.429331
H	-2.794516	-2.078533	-1.379460
C	-2.025546	-2.179166	-2.169064
H	-1.404232	-3.051608	-1.886373
H	-2.568521	-2.463814	-3.088719
O	-1.298153	-1.016577	-2.401038
C	-2.015467	1.428966	-1.385757
H	-2.064386	1.480902	-2.479039
H	-1.903722	2.443500	-0.989421
C	-3.238352	0.763503	-0.815824
C	-4.504261	1.179547	-0.987371
C	-5.711378	0.543642	-0.432977
C	-5.714574	-0.758633	0.106713
H	-4.803790	-1.348301	0.092783
C	-6.876279	-1.312674	0.637893
H	-6.851693	-2.320491	1.043837
C	-8.073665	-0.590181	0.636349
H	-8.979645	-1.027536	1.046552
C	-8.093252	0.694608	0.091315
H	-9.017533	1.266433	0.076351
C	-6.928155	1.250503	-0.437744
H	-6.953676	2.253510	-0.857371
H	-3.082869	-0.127456	-0.207773
H	-4.680332	2.079831	-1.577561

IV’^{Pd}

	Value
Charge	0
Electronic Energy, BS1 (a.u.)	-2663.059216
Thermal and entropic correction, BS1 (a.u.)	0.644415
Electronic Energy, BS2 (a.u.)	-2663.800630

Molecular Geometry in Cartesian Coordinates

Pd	-0.086246	0.397381	-1.299738
P	-0.224413	-1.635440	-0.023627
P	1.632877	1.253775	-0.057137
C	-1.992339	-2.069293	0.243556
C	0.542076	-3.120188	-0.777139
C	0.434178	-1.602122	1.703952
C	1.320037	1.760595	1.677867
C	2.952528	-0.053775	0.032235
C	2.488064	2.703710	-0.820045
C	-2.906180	-1.018137	0.416606
C	-2.449446	-3.392936	0.310349
C	0.673106	-3.135217	-2.175375
C	0.997547	-4.212096	-0.022518
C	1.804743	-1.356605	1.948573
C	-0.426365	-1.783981	2.798812
C	-0.004799	1.967592	2.084360
C	2.361144	1.962804	2.597785
C	4.033854	0.007985	-0.862149
C	2.837146	-1.187414	0.875942
C	2.662054	2.697324	-2.216770
C	2.938249	3.812015	-0.089611
H	-2.571152	0.010133	0.325834
C	-4.252457	-1.282046	0.668629
C	-3.799754	-3.655764	0.548779
H	-1.756950	-4.216875	0.170285
C	1.253315	-4.237893	-2.805257

H	0.318996	-2.282843	-2.753600
C	1.579405	-5.308504	-0.659704
H	0.905134	-4.201694	1.059514
C	2.264335	-1.330031	3.273750
H	-1.478728	-1.976181	2.628384
C	0.044439	-1.729511	4.109997
H	-0.812799	1.782901	1.383429
C	-0.285873	2.386418	3.386056
H	3.389738	1.779867	2.299980
C	2.078806	2.384916	3.895974
H	4.155151	0.871751	-1.503539
C	4.972301	-1.018971	-0.949521
C	3.798963	-2.205471	0.778792
H	2.277278	1.865874	-2.800979
C	3.302936	3.755334	-2.858591
H	2.797152	3.857969	0.983887
C	3.566178	4.878720	-0.738283
H	-4.945063	-0.455482	0.791763
C	-4.700670	-2.603494	0.732248
H	-4.147259	-4.684369	0.590677
C	1.708844	-5.321694	-2.051196
H	1.353844	-4.245906	-3.887037
H	1.933187	-6.150221	-0.070619
C	1.398211	-1.505725	4.350564
H	-0.647276	-1.869225	4.935676
H	-1.316998	2.537495	3.692539
C	0.755038	2.599662	4.290899
H	2.889898	2.537585	4.602255
C	4.852164	-2.138011	-0.129444
H	5.792520	-0.936904	-1.656703
H	3.431279	3.730381	-3.936792
C	3.757215	4.850940	-2.119551
H	3.904077	5.732546	-0.157766
H	-5.750864	-2.812971	0.915825
H	2.165901	-6.174731	-2.545265
H	1.779871	-1.463033	5.366454
H	0.536914	2.923189	5.304679
H	5.571292	-2.949541	-0.189228
H	4.245950	5.681826	-2.620224
H	3.700569	-3.069402	1.428685
H	3.319859	-1.147677	3.452088
H	-3.014921	-0.689786	-2.290483
C	-2.224547	-1.147753	-2.913670
H	-2.099664	-2.183762	-2.538066
H	-2.643124	-1.233591	-3.934179
O	-1.046039	-0.412959	-2.951662
C	-0.632178	2.248897	-2.185422
H	0.127228	3.021466	-2.080180
H	-0.824210	1.976742	-3.223522
C	-1.818642	2.463969	-1.365164
C	-3.076801	2.033820	-1.656186
C	-4.240100	2.077665	-0.774982
C	-4.179685	2.483022	0.577580
H	-3.233980	2.809066	1.000596
C	-5.310682	2.459331	1.388696
H	-5.231568	2.773019	2.426558
C	-6.542171	2.027965	0.882180
H	-7.421729	2.006892	1.519173
C	-6.622682	1.621335	-0.452459
H	-7.569955	1.279430	-0.861186
C	-5.490728	1.644205	-1.264591
H	-5.562399	1.313009	-2.297569
H	-1.649639	2.970821	-0.414420
H	-3.233538	1.561791	-2.623538

I-OMe^{PdCu}

	Value
Charge	0
Electronic Energy, BS1 (a.u.)	-5342.122759

	Value
Thermal and entropic correction, BS1 (a.u.)	1.283805
Electronic Energy, BS2 (a.u.)	-6786.808409

Molecular Geometry in Cartesian Coordinates

Cu	1.845328	-0.142984	-0.290008
P	3.565382	0.769836	1.035243
P	2.894046	-2.259326	-0.513648
C	3.982110	2.481833	0.475495
C	3.321954	0.998717	2.850885
C	5.241022	-0.038522	0.981106
C	4.444731	-2.059197	-1.497916
C	3.355989	-2.873543	1.166418
C	2.191796	-3.747559	-1.362698
C	3.837419	2.758519	-0.891568
C	4.448845	3.493267	1.329172
C	2.016352	1.230059	3.310778
C	4.377313	0.972356	3.777159
C	5.429550	-1.363537	1.442001
C	6.356273	0.665350	0.497525
C	4.515818	-0.950745	-2.353766
C	5.496215	-2.986832	-1.492526
C	2.494627	-3.808450	1.773107
C	4.352591	-2.262228	1.970099
C	1.136270	-3.542171	-2.262415
C	2.703373	-5.047147	-1.207887
H	3.455356	1.986089	-1.550201
C	4.161430	4.016751	-1.401485
C	4.754501	4.758197	0.822191
H	4.563885	3.299741	2.390554
C	1.774365	1.435039	4.669679
H	1.191081	1.213230	2.606917
C	4.130488	1.173310	5.136145
H	5.392688	0.793665	3.437935
C	6.726917	-1.900899	1.453102
H	6.234052	1.677437	0.132214
C	7.630947	0.101000	0.481197
H	3.703100	-0.230691	-2.364877
C	5.616721	-0.775278	-3.194899
H	5.460217	-3.844047	-0.827079
C	6.598656	-2.806217	-2.326241
H	1.720097	-4.282372	1.180071
C	2.590953	-4.130870	3.124181
C	4.433511	-2.601449	3.331139
H	0.725811	-2.546963	-2.385545
C	0.599490	-4.607815	-2.988800
H	3.514033	-5.231229	-0.509904
C	2.164038	-6.112303	-1.927897
H	4.035631	4.206062	-2.462993
C	4.614179	5.022232	-0.543301
H	5.103597	5.536860	1.494987
C	2.828137	1.406147	5.584856
H	0.756384	1.594706	5.009560
H	4.955820	1.147637	5.842845
C	7.822684	-1.186477	0.976048
H	8.467413	0.674228	0.091624
H	5.651649	0.086175	-3.856026
C	6.659840	-1.701691	-3.182060
H	7.412467	-3.526215	-2.307579
C	3.560076	-3.515287	3.915629
H	1.903374	-4.853814	3.554133
H	-0.226434	-4.427848	-3.670301
C	1.110266	-5.894909	-2.821010
H	2.566021	-7.112781	-1.792208
H	4.854436	6.006897	-0.935215
H	2.635892	1.557376	6.643765
H	8.810784	-1.637632	0.981014
H	7.519428	-1.565872	-3.832861
H	3.640370	-3.744979	4.974121
H	0.688798	-6.726729	-3.378648
H	5.199906	-2.125000	3.935015

H	6.861816	-2.913475	1.822349
Pd	-1.638596	-0.315913	0.032766
P	-2.265772	1.647705	1.259292
P	-3.237841	-0.045850	-1.590658
C	-2.361629	1.056385	3.005138
C	-1.242504	3.165748	1.339140
C	-3.963203	2.318594	0.966070
C	-5.017329	-0.147216	-1.149813
C	-3.004751	1.672748	-2.250656
C	-3.117066	-1.194342	-3.036798
C	-2.943517	-0.201601	3.243217
C	-1.895335	1.802420	4.095335
C	0.102560	3.092448	0.957940
C	-1.769425	4.385087	1.802416
C	-4.333407	2.782408	-0.317276
C	-4.897460	2.390469	2.011424
C	-5.401370	-0.806514	0.022974
C	-6.004837	0.365299	-2.007019
C	-2.265967	1.866655	-3.427699
C	-3.433813	2.811767	-1.519187
C	-1.872284	-1.373435	-3.666785
C	-4.199308	-1.983099	-3.456982
H	-3.287336	-0.804182	2.408414
C	-3.072526	-0.692719	4.541149
C	-2.013985	1.302323	5.395709
H	-1.437175	2.772657	3.935809
C	0.917001	4.221330	1.068934
H	0.508478	2.164411	0.568788
C	-0.952626	5.509943	1.902002
H	-2.815136	4.452253	2.087092
C	-5.619137	3.317226	-0.494583
H	-4.621815	2.044618	3.000788
C	-6.177092	2.905695	1.809575
H	-4.644869	-1.211533	0.683369
C	-6.754000	-0.959533	0.336278
H	-5.715696	0.883030	-2.917246
C	-7.353777	0.214079	-1.692442
H	-1.923682	1.017140	-4.001920
C	-1.952565	3.142260	-3.893658
C	-3.107723	4.086843	-2.008973
H	-1.007770	-0.816989	-3.321914
C	-1.726825	-2.277039	-4.717556
H	-5.166388	-1.891113	-2.977618
C	-4.044614	-2.904561	-4.496517
H	-3.533739	-1.663087	4.699702
C	-2.604210	0.058285	5.622859
H	-1.645621	1.891438	6.231166
C	0.395893	5.425115	1.540737
H	1.953014	4.157968	0.764239
H	-1.366278	6.448154	2.261957
C	-6.539624	3.375303	0.549195
H	-6.880296	2.942681	2.636578
H	-7.035847	-1.484052	1.243976
C	-7.730166	-0.450325	-0.520576
H	-8.111267	0.616460	-2.359185
C	-2.374868	4.260285	-3.180698
H	-1.370119	3.248360	-4.803816
H	-0.756996	-2.386349	-5.194276
C	-2.814014	-3.048682	-5.136627
H	-4.894194	-3.507839	-4.804036
H	-2.696855	-0.325195	6.634997
H	1.037307	6.299101	1.618056
H	-7.532402	3.779602	0.374423
H	-8.782559	-0.568134	-0.277752
H	-2.140531	5.261507	-3.530218
H	-2.698874	-3.761776	-5.948008
H	-3.443249	4.953728	-1.447708
H	-5.899921	3.672390	-1.481506
H	-0.370735	-1.122987	3.002857
C	0.331926	-1.278576	2.167412
H	1.348310	-1.195362	2.571977
H	0.227094	-2.319568	1.829222
O	0.149941	-0.340157	1.137513
C	-1.321836	-2.354148	-0.410119

H	-0.253449	-2.438426	-0.222494
H	-1.571975	-2.638681	-1.429923
C	-2.175954	-2.963470	0.604622
C	-3.395987	-3.518675	0.385708
C	-4.341033	-3.963719	1.407816
C	-4.044776	-3.982161	2.790076
H	-3.055444	-3.695979	3.133855
C	-5.000907	-4.365912	3.726067
H	-4.742008	-4.374303	4.781991
C	-6.285701	-4.745098	3.320055
H	-7.028857	-5.043192	4.053956
C	-6.595949	-4.740288	1.958265
H	-7.587749	-5.034397	1.624435
C	-5.638974	-4.357272	1.019291
H	-5.895798	-4.347037	-0.037096
C	0.838736	0.548489	-1.973582
H	0.444366	-0.412960	-2.315931
H	0.017113	1.107079	-1.510340
C	1.423685	1.327371	-3.086016
C	1.964203	0.818378	-4.230533
H	2.358000	1.513720	-4.970688
C	2.013937	-0.636794	-4.606035
H	2.007663	-1.291189	-3.731488
H	2.911494	-0.877505	-5.188255
H	1.151970	-0.923377	-5.231713
B	1.357042	2.876004	-2.993380
O	1.907713	3.747881	-3.925051
C	1.470586	5.073591	-3.594544
H	0.715797	5.395261	-4.324485
H	2.316438	5.767409	-3.649689
C	0.891555	4.950494	-2.170115
H	1.588867	5.322284	-1.412153
H	-0.064347	5.466613	-2.045567
O	0.704822	3.545447	-1.981944
H	-1.817280	-2.897648	1.630363
H	-3.753395	-3.576851	-0.642639

I-OMeCO2^{PdCu}

	Value
Charge	0
Electronic Energy, BS1 (a.u.)	-5530.731991
Thermal and entropic correction, BS1 (a.u.)	1.291564
Electronic Energy, BS2 (a.u.)	-6975.499954

Molecular Geometry in Cartesian Coordinates

C	-2.389840	1.236819	2.750437
H	-3.087538	0.879032	3.523998
B	-0.862842	-0.715958	3.512073
O	-1.937002	-1.493525	3.900957
O	0.343514	-1.385444	3.663893
C	-1.458097	-2.782909	4.306084
C	0.084716	-2.670110	4.249484
H	-1.844799	-3.539620	3.617101
H	-1.826368	-3.004521	5.313436
H	0.538625	-3.449062	3.629352
H	0.542724	-2.709176	5.245573
Cu	-3.171747	0.441578	1.071192
P	-4.500397	1.626193	-0.447303
P	-3.907137	-1.683592	0.550731
C	-4.627290	3.409684	0.010750
C	-4.207118	1.615593	-2.259723
C	-6.230889	0.981996	-0.284966
C	-5.320541	-2.140013	1.640753
C	-4.537691	-1.848159	-1.180074
C	-2.809551	-3.151104	0.750852
C	-4.632035	3.705861	1.386310
C	-4.625724	4.470238	-0.907015
C	-3.001612	1.066704	-2.715410

C	-5.149054	2.089112	-3.188682
C	-6.578378	-0.286920	-0.815430
C	-7.198828	1.702957	0.432973
C	-5.269695	-1.680993	2.966768
C	-6.393209	-2.947046	1.236165
C	-3.769020	-2.581985	-2.099710
C	-5.667153	-1.134620	-1.656032
C	-1.438345	-2.929158	0.919757
C	-3.295046	-4.468819	0.723996
H	-4.593342	2.892122	2.104340
C	-4.662853	5.026914	1.831211
C	-4.644064	5.793799	-0.458631
H	-4.593637	4.270908	-1.972061
C	-2.736016	1.007013	-4.085683
H	-2.286708	0.646222	-2.016313
C	-4.874684	2.036834	-4.554421
H	-6.100474	2.484051	-2.842830
C	-7.889446	-0.754085	-0.635986
H	-6.950159	2.676578	0.839637
C	-8.485822	1.203142	0.627320
H	-4.427303	-1.074611	3.287261
C	-6.278233	-2.024595	3.868965
H	-6.447210	-3.298854	0.210533
C	-7.400507	-3.287044	2.139809
H	-2.875035	-3.085580	-1.757558
C	-4.106275	-2.644480	-3.448808
C	-6.007051	-1.237547	-3.015145
H	-1.054724	-1.916385	0.950698
C	-0.558341	-4.010178	1.023445
H	-4.358491	-4.655114	0.608088
C	-2.418065	-5.546221	0.845986
H	-4.668737	5.235069	2.897423
C	-4.666700	6.076295	0.908385
H	-4.638706	6.604161	-1.182750
C	-3.664756	1.496780	-5.004141
H	-1.806792	0.558485	-4.423150
H	-5.606644	2.406012	-5.267890
C	-8.837183	-0.030859	0.084548
H	-9.209849	1.785208	1.190613
H	-6.229150	-1.663765	4.892789
C	-7.346360	-2.824650	3.457047
H	-8.230092	-3.908740	1.813761
C	-5.238924	-1.976754	-3.910781
H	-3.480542	-3.210587	-4.133211
H	0.504499	-3.825847	1.128612
C	-1.045326	-5.317898	0.988499
H	-2.804172	-6.562172	0.825064
H	-4.678806	7.106541	1.253030
H	-3.457168	1.447305	-6.069959
H	-9.837715	-0.431126	0.220462
H	-8.134057	-3.086571	4.158330
H	-5.516320	-2.014722	-4.960309
H	-0.359318	-6.156447	1.073515
H	-2.453154	2.331417	2.735643
C	-1.010657	0.754914	3.039495
C	0.125332	1.501151	2.969906
H	1.075935	1.009466	3.169497
C	0.223773	2.974534	2.692923
H	-0.724888	3.406589	2.369248
H	0.548782	3.518542	3.592428
H	0.967135	3.193059	1.919184
H	-6.877519	-0.694747	-3.370829
H	-8.155804	-1.716710	-1.061951
Pd	2.645950	-0.348958	0.365266
P	2.506915	1.356745	-1.313103
P	4.388722	0.563007	1.488225
C	2.182510	0.502243	-2.909910
C	1.170268	2.595261	-1.160846
C	4.038798	2.326135	-1.661115
C	6.006373	0.470425	0.628327
C	4.079418	2.368599	1.776252
C	4.701428	-0.103225	3.180553
C	2.942150	-0.638192	-3.220943
C	1.224009	0.956508	-3.825341

C	-0.017219	2.207347	-0.520657
C	1.290830	3.894725	-1.679867
C	4.652370	3.101433	-0.650259
C	4.634781	2.276471	-2.932417
C	6.187055	-0.506288	-0.360605
C	7.066496	1.333355	0.947385
C	3.720982	2.789625	3.067488
C	4.110140	3.327936	0.731083
C	3.589355	-0.458748	3.965633
C	5.989394	-0.258420	3.712183
H	3.658307	-1.025989	-2.502630
C	2.777100	-1.285375	-4.443675
C	1.047652	0.293640	-5.042659
H	0.613719	1.822256	-3.591369
C	-1.065698	3.119194	-0.400618
H	-0.120050	1.207700	-0.110763
C	0.234460	4.797581	-1.555703
H	2.205831	4.198460	-2.179579
C	5.834222	3.795658	-0.957559
H	4.167210	1.700311	-3.721239
C	5.818184	2.957801	-3.211083
H	5.368573	-1.170126	-0.617758
C	7.413735	-0.626583	-1.015716
H	6.929039	2.107785	1.696594
C	8.291065	1.210329	0.291849
H	3.701780	2.076091	3.881222
C	3.382027	4.113768	3.338898
C	3.741777	4.651422	1.025141
H	2.584774	-0.396686	3.559811
C	3.766205	-0.927294	5.266287
H	6.863245	-0.009909	3.120425
C	6.161054	-0.743333	5.011270
H	3.376935	-2.162108	-4.667182
C	1.828647	-0.819825	-5.358286
H	0.303277	0.655712	-5.746135
C	-0.945307	4.410992	-0.913762
H	-1.961979	2.816652	0.127675
H	0.334708	5.802233	-1.957791
C	6.423962	3.722037	-2.216876
H	6.255413	2.893056	-4.203207
H	7.540074	-1.388966	-1.779031
C	8.466236	0.230463	-0.689713
H	9.105333	1.885115	0.540253
C	3.374245	5.048837	2.308551
H	3.108656	4.400276	4.349825
H	2.894817	-1.190443	5.858845
C	5.052581	-1.071148	5.793276
H	7.165026	-0.863358	5.409112
H	1.692339	-1.328961	-6.308378
H	-1.768546	5.110989	-0.807531
H	7.346984	4.258770	-2.415724
H	9.419972	0.140515	-1.202120
H	3.091242	6.080112	2.497204
H	5.190066	-1.445737	6.803791
H	3.758042	5.379597	0.220151
H	6.300621	4.391289	-0.178805
O	0.698839	-0.951190	-0.264442
C	2.792749	-2.200430	1.386335
H	1.787087	-2.268393	1.799483
H	3.552795	-2.247068	2.163835
C	3.087991	-3.019181	0.217171
C	4.323234	-3.493166	-0.098434
C	4.708903	-4.128034	-1.356003
C	3.857555	-4.168316	-2.483463
H	2.877319	-3.708070	-2.426550
C	4.273167	-4.762688	-3.671561
H	3.597566	-4.784631	-4.523011
C	5.551695	-5.321302	-3.782556
H	5.872426	-5.779756	-4.713663
C	6.412181	-5.274930	-2.683614
H	7.410114	-5.699167	-2.755696
C	5.996862	-4.686666	-1.489610
H	6.673875	-4.657181	-0.639105
H	5.128578	-3.351302	0.623441

H	2.268981	-3.176077	-0.478523
C	0.078801	-1.423693	-1.276568
O	-1.061337	-1.125303	-1.640297
O	0.779794	-2.402392	-1.955760
C	0.077923	-3.023112	-3.033582
H	-0.698477	-3.696968	-2.653449
H	-0.382228	-2.281414	-3.689838
H	0.818510	-3.603588	-3.589102

II^{PdCu}

	Value
Charge	0
Electronic Energy, BS1 (a.u.)	-5342.113373
Thermal and entropic correction, BS1 (a.u.)	1.289279
Electronic Energy, BS2 (a.u.)	-6786.799665
Number of Imaginary Frequencies	0
Imaginary frequencies (cm-1)	None

Molecular Geometry in Cartesian Coordinates

C	-1.509141	-0.625904	-2.659610
H	-2.463168	-0.392315	-3.153966
B	-0.703686	1.608476	-3.667259
O	-1.953024	2.189410	-3.542885
O	0.234186	2.511720	-4.152746
C	-1.900050	3.512128	-4.092096
C	-0.392561	3.799809	-4.247505
H	-2.401845	4.209897	-3.415459
H	-2.424847	3.520558	-5.055579
H	-0.009137	4.440890	-3.444225
H	-0.144044	4.262560	-5.208281
Cu	-1.907904	-0.203194	-0.678292
P	-3.606624	-1.768071	0.129037
P	-3.111509	1.748220	0.137539
C	-3.682766	-3.278199	-0.940495
C	-3.561754	-2.522865	1.810486
C	-5.350277	-1.120170	0.029454
C	-4.578786	2.149674	-0.917486
C	-3.821218	1.352409	1.804264
C	-2.462195	3.482092	0.326689
C	-3.690119	-3.108597	-2.336836
C	-3.689140	-4.583407	-0.425119
C	-2.307368	-2.694342	2.414075
C	-4.713678	-2.945269	2.492809
C	-5.775564	-0.059587	0.868832
C	-6.260454	-1.628708	-0.911193
C	-4.714531	1.492591	-2.144465
C	-5.512314	3.134699	-0.559918
C	-3.116263	1.760854	2.954394
C	-4.945821	0.510898	1.979615
C	-1.558812	3.915125	-0.655703
C	-2.870562	4.402698	1.307073
H	-3.672249	-2.109590	-2.755112
C	-3.715121	-4.211080	-3.191042
C	-3.702151	-5.686923	-1.281771
H	-3.675950	-4.746040	0.646496
C	-2.214360	-3.249962	3.691800
H	-1.410102	-2.382255	1.886371
C	-4.616820	-3.503540	3.766981
H	-5.688250	-2.829217	2.028781
C	-7.080701	0.436041	0.735874
H	-5.969386	-2.452186	-1.551347
C	-7.545789	-1.105854	-1.046414
H	-3.978565	0.749401	-2.426113
C	-5.776536	1.803760	-2.997570
H	-5.412561	3.660280	0.384987
C	-6.571405	3.444267	-1.410193

H	-2.224797	2.366892	2.846261
C	-3.522580	1.403525	4.236571
C	-5.357080	0.182937	3.283246
H	-1.251783	3.223981	-1.429435
C	-1.047668	5.213138	-0.639330
H	-3.590227	4.113674	2.064774
C	-2.356761	5.700994	1.325028
H	-3.719480	-4.055542	-4.266176
C	-3.716919	-5.506440	-2.665684
H	-3.698643	-6.689298	-0.862325
C	-3.365859	-3.648948	4.372129
H	-1.240423	-3.356025	4.155215
H	-5.516801	-3.819886	4.287858
C	-7.961001	-0.064494	-0.220096
H	-8.218099	-1.520071	-1.792621
H	-5.872241	1.284225	-3.947119
C	-6.706474	2.777020	-2.632297
H	-7.291635	4.205333	-1.121625
C	-4.661887	0.617944	4.406904
H	-2.948235	1.739656	5.095281
H	-0.328726	5.516762	-1.394842
C	-1.436402	6.108902	0.357268
H	-2.679276	6.393893	2.097628
H	-3.725393	-6.366020	-3.330108
H	-3.289406	-4.072424	5.370162
H	-8.957576	0.356537	-0.315663
H	-7.532601	3.019191	-3.295602
H	-4.996790	0.332314	5.399734
H	-1.025612	7.114086	0.381865
H	-1.359309	-1.710528	-2.718023
C	-0.422445	0.123125	-3.330756
C	0.771359	-0.391712	-3.741726
H	1.486136	0.275348	-4.221636
C	1.197068	-1.827532	-3.638688
H	0.340819	-2.500315	-3.537808
H	1.769267	-2.134278	-4.521441
H	1.844161	-2.009970	-2.771333
H	-6.229330	-0.452211	3.401468
H	-7.392919	1.246449	1.387661
Pd	1.712546	-0.019264	-0.079833
P	2.412103	-2.204994	0.665364
P	3.793888	0.480728	-0.934718
C	1.935047	-2.452759	2.424397
C	1.632825	-3.600297	-0.233650
C	4.211437	-2.665252	0.737036
C	5.053143	0.581715	0.398226
C	4.349068	-0.807778	-2.148393
C	4.030980	2.089742	-1.811131
C	2.210819	-1.417915	3.332942
C	1.437177	-3.670292	2.905860
C	0.338674	-3.405975	-0.744638
C	2.280852	-4.829413	-0.431942
C	5.043595	-2.594751	-0.403324
C	4.773833	-3.114875	1.945705
C	4.648392	1.086510	1.644128
C	6.402198	0.263953	0.191515
C	4.312623	-0.486216	-3.515689
C	4.654183	-2.144752	-1.779440
C	3.111580	2.464888	-2.805040
C	5.058821	2.983277	-1.468727
H	2.599533	-0.472602	2.967498
C	2.008408	-1.603456	4.700214
C	1.241598	-3.856121	4.276959
H	1.218811	-4.479394	2.216534
C	-0.288545	-4.430997	-1.452117
H	-0.166723	-2.454815	-0.602042
C	1.644916	-5.850436	-1.140313
H	3.279609	-4.988786	-0.037812
C	6.384528	-3.006812	-0.289903
H	4.158272	-3.184555	2.832295
C	6.111436	-3.491583	2.043835
H	3.608054	1.352038	1.805623
C	5.578706	1.262212	2.669314
H	6.726272	-0.118350	-0.771019

C	7.327579	0.426524	1.222929
H	4.119557	0.531760	-3.824850
C	4.514364	-1.445503	-4.505527
C	4.818441	-3.101842	-2.795825
H	2.297218	1.806332	-3.078033
C	3.227381	3.693806	-3.452510
H	5.776701	2.727016	-0.698315
C	5.162794	4.219247	-2.110313
H	2.226099	-0.794263	5.391402
C	1.527122	-2.825938	5.175909
H	0.864145	-4.807894	4.640213
C	0.361049	-5.650665	-1.652535
H	-1.276755	-4.265728	-1.861221
H	2.154101	-6.798374	-1.292323
C	6.924123	-3.446032	0.914669
H	6.505662	-3.827854	2.998341
H	5.253794	1.664922	3.624206
C	6.918046	0.925292	2.462150
H	8.368171	0.162365	1.057912
C	4.737361	-2.770965	-4.145499
H	4.473018	-1.152990	-5.550361
H	2.505683	3.950280	-4.220846
C	4.250451	4.577653	-3.104165
H	5.960714	4.900964	-1.829638
H	1.373050	-2.973944	6.240993
H	-0.134078	-6.440909	-2.210124
H	7.967681	-3.742463	0.967328
H	7.641674	1.053177	3.262247
H	4.864550	-3.539288	-4.902242
H	4.335130	5.540385	-3.600470
H	5.036703	-4.124830	-2.505542
H	7.016133	-2.952811	-1.171367
H	0.215397	-0.176240	2.715047
C	-0.489154	0.110132	1.920792
H	-1.489212	-0.187628	2.253279
H	-0.483067	1.207823	1.864381
O	-0.197009	-0.496737	0.685410
C	0.946316	1.907492	-0.415511
H	1.059006	2.101498	-1.480400
H	-0.100251	1.765646	-0.164516
C	1.648315	2.864471	0.437399
C	1.180600	3.409875	1.586297
C	1.914305	4.311154	2.474153
C	3.312550	4.498457	2.403072
H	3.893008	3.943981	1.672346
C	3.965381	5.370249	3.270345
H	5.043073	5.492312	3.194237
C	3.248896	6.078511	4.241254
H	3.762243	6.754926	4.918876
C	1.866278	5.899268	4.332589
H	1.295737	6.441296	5.082592
C	1.210939	5.028210	3.464055
H	0.132937	4.903542	3.534278
H	0.152746	3.200888	1.874928
H	2.644925	3.153129	0.108026

TrMt-SR^{PdCu}

	Value
Charge	0
Electronic Energy, BS1 (a.u.)	-5342.085672
Thermal and entropic correction, BS1 (a.u.)	1.282616
Electronic Energy, BS2 (a.u.)	-6786.771058

Molecular Geometry in Cartesian Coordinates

C	1.723021	0.673024	-2.528547
H	2.631171	0.225626	-2.922256
B	0.648861	-1.476846	-3.207872
O	1.820650	-2.203300	-3.141309

O	-0.368378	-2.186361	-3.822485
C	1.624666	-3.445602	-3.834693
C	0.097527	-3.531068	-4.033625
H	2.024515	-4.264388	-3.230349
H	2.168066	-3.411156	-4.786416
H	-0.379050	-4.188841	-3.296205
H	-0.186062	-3.865204	-5.035983
Cu	2.200440	0.139079	-0.392931
P	4.070007	1.554140	0.001793
P	3.297805	-1.937515	0.139423
C	4.242376	3.074639	-1.041381
C	4.085467	2.229751	1.712413
C	5.748026	0.767734	-0.164987
C	4.667692	-2.480585	-0.982649
C	4.125765	-1.534584	1.750953
C	2.499019	-3.581784	0.459150
C	4.130106	2.947224	-2.437482
C	4.445667	4.355768	-0.503911
C	2.869748	2.250882	2.409073
C	5.246574	2.726322	2.327143
C	6.129021	-0.316595	0.664202
C	6.635252	1.179048	-1.173408
C	4.867462	-1.770700	-2.172705
C	5.482219	-3.589877	-0.707018
C	3.453125	-1.848383	2.948016
C	5.324449	-0.788551	1.836656
C	1.416461	-3.924585	-0.362389
C	2.946532	-4.524495	1.401315
H	3.990896	1.968610	-2.881083
C	4.209776	4.064763	-3.268189
C	4.516143	5.475632	-1.336160
H	4.534706	4.489461	0.568093
C	2.820103	2.764141	3.707464
H	1.977672	1.847469	1.934848
C	5.190411	3.239817	3.621984
H	6.192614	2.703800	1.793667
C	7.369294	-0.936000	0.452224
H	6.378404	2.017825	-1.808600
C	7.853510	0.535295	-1.384380
H	4.232507	-0.920050	-2.389248
C	5.872821	-2.147974	-3.064726
H	5.336796	-4.160042	0.205147
C	6.483669	-3.970561	-1.598843
H	2.513561	-2.385148	2.907351
C	3.960712	-1.488847	4.193392
C	5.835973	-0.458924	3.103419
H	1.071795	-3.214542	-1.099241
C	0.781209	-5.160850	-0.237705
H	3.785405	-4.294991	2.049295
C	2.310690	-5.760125	1.528073
H	4.118469	3.940724	-4.343597
C	4.394560	5.335662	-2.719168
H	4.665230	6.458604	-0.897557
C	3.975351	3.257731	4.314672
H	1.876941	2.761120	4.243936
H	6.093198	3.619665	4.093030
C	8.223707	-0.533037	-0.570721
H	8.509725	0.875771	-2.180439
H	6.021432	-1.580912	-3.979982
C	6.682262	-3.248940	-2.780239
H	7.109938	-4.829737	-1.371996
C	5.169381	-0.799135	4.275996
H	3.409245	-1.748711	5.092481
H	-0.068179	-5.393658	-0.873455
C	1.223095	-6.081692	0.712530
H	2.666666	-6.472321	2.267548
H	4.444529	6.208118	-3.364519
H	3.933932	3.650672	5.327093
H	9.166719	-1.048841	-0.726958
H	7.464412	-3.544956	-3.474070
H	5.581424	-0.513521	5.239411
H	0.723963	-7.040418	0.820575
H	1.730223	1.755701	-2.438469
C	0.518693	-0.008855	-2.714790

C	-0.779624	0.582340	-2.616699
H	-1.545844	0.013805	-3.142118
C	-0.904923	2.078095	-2.829667
H	-0.317889	2.653938	-2.113608
H	-0.539662	2.359846	-3.833105
H	-1.940073	2.413479	-2.750042
H	6.763335	0.103324	3.152880
H	7.648153	-1.764537	1.096250
Pd	-1.902068	0.060946	-0.528365
P	-2.322231	2.160526	0.550732
P	-4.214551	-0.309970	-1.063127
C	-2.005028	1.762832	2.329583
C	-1.306398	3.655503	0.238985
C	-4.047799	2.838188	0.665512
C	-5.668267	-0.297040	0.065326
C	-4.469975	1.086231	-2.260586
C	-4.541497	-1.851345	-2.038319
C	-2.708397	0.679083	2.880105
C	-1.118245	2.476005	3.145390
C	0.087298	3.471733	0.184993
C	-1.848792	4.923387	-0.009167
C	-4.915845	2.889777	-0.446757
C	-4.490368	3.371583	1.890299
C	-5.531538	-0.995301	1.275160
C	-6.899674	0.299499	-0.240782
C	-4.337328	0.829130	-3.635666
C	-4.593394	2.437234	-1.840099
C	-3.571830	-2.303410	-2.953160
C	-5.678561	-2.648901	-1.824254
H	-3.395149	0.117481	2.259621
C	-2.532938	0.313680	4.213585
C	-0.940003	2.106005	4.481543
H	-0.562817	3.317073	2.746300
C	0.917740	4.555136	-0.094577
H	0.493766	2.475658	0.361395
C	-1.008659	5.998919	-0.309960
H	-2.922279	5.076855	0.032237
C	-6.183232	3.481544	-0.287952
H	-3.828164	3.352044	2.747930
C	-5.755027	3.935264	2.033180
H	-4.591498	-1.483387	1.509380
C	-6.598804	-1.088208	2.168441
H	-7.026226	0.824978	-1.181741
C	-7.962164	0.221383	0.660551
H	-4.263503	-0.192135	-3.986413
C	-4.292588	1.854161	-4.578947
C	-4.552448	3.454571	-2.808742
H	-2.669790	-1.730741	-3.126781
C	-3.739995	-3.507033	-3.636443
H	-6.444048	-2.332815	-1.125123
C	-5.835836	-3.861782	-2.499191
H	-3.081712	-0.534699	4.611516
C	-1.641597	1.026093	5.019627
H	-0.257488	2.674312	5.107421
C	0.374446	5.817320	-0.350469
H	1.990031	4.409816	-0.133293
H	-1.438357	6.977637	-0.506878
C	-6.609569	3.992424	0.933260
H	-6.063905	4.332712	2.995698
H	-6.474736	-1.644330	3.093196
C	-7.814358	-0.470239	1.866197
H	-8.908199	0.698176	0.418714
C	-4.387198	3.179632	-4.164111
H	-4.180824	1.609625	-5.631275
H	-2.977527	-3.827963	-4.340505
C	-4.869730	-4.296188	-3.408062
H	-6.720976	-4.464330	-2.313661
H	-1.497198	0.744274	6.058995
H	1.029891	6.651844	-0.585329
H	-7.600089	4.429167	1.023230
H	-8.645021	-0.529741	2.564003
H	-4.341547	3.992430	-4.882839
H	-4.995461	-5.239982	-3.931407
H	-4.650145	4.483919	-2.477024

H	-6.845294	3.520761	-1.147773
H	-0.332108	-0.304838	2.487888
C	0.624424	-0.364730	1.947861
H	1.408938	-0.073516	2.671155
H	0.799576	-1.437284	1.734061
O	0.624888	0.422515	0.798114
C	-1.249374	-1.929903	-0.634449
H	-1.241881	-2.307246	-1.651837
H	-0.248960	-1.733226	-0.250069
C	-2.134129	-2.666272	0.257572
C	-1.969857	-2.819351	1.597168
C	-2.908890	-3.441870	2.526783
C	-4.112317	-4.066370	2.129239
H	-4.366175	-4.129399	1.075598
C	-4.992345	-4.594800	3.070038
H	-5.913300	-5.064551	2.734646
C	-4.702539	-4.523216	4.437211
H	-5.392897	-4.935736	5.167487
C	-3.512606	-3.918001	4.850098
H	-3.271019	-3.857318	5.908116
C	-2.630416	-3.389012	3.909725
H	-1.710937	-2.912290	4.239787
H	-1.075906	-2.401755	2.049246
H	-3.028667	-3.076955	-0.207720

TrMt-SS^{PdCu}

	Value
Charge	0
Electronic Energy, BS1 (a.u.)	-5342.074556
Thermal and entropic correction, BS1 (a.u.)	1.291016
Electronic Energy, BS2 (a.u.)	-6786.759969

Molecular Geometry in Cartesian Coordinates

C	-1.712544	-0.535782	-2.636942
H	-1.860402	0.462759	-3.039684
B	-0.293456	-2.615749	-2.338457
O	-1.341184	-3.495063	-2.507773
O	0.924099	-3.251791	-2.259404
C	-0.820626	-4.831951	-2.468000
C	0.716447	-4.654517	-2.461044
H	-1.176787	-5.383415	-3.344981
H	-1.186100	-5.329299	-1.565262
H	1.173255	-4.945432	-3.414785
H	1.197605	-5.212804	-1.654755
Cu	-2.332987	0.069420	-0.402793
P	-4.199195	-1.090022	0.489548
P	-3.392633	2.140037	-0.665744
C	-4.447975	-2.880217	0.099605
C	-4.144157	-1.107407	2.328986
C	-5.866140	-0.375573	0.083070
C	-4.749276	2.251928	-1.920124
C	-4.195722	2.439339	0.970855
C	-2.492064	3.702769	-1.058548
C	-4.037922	-3.336174	-1.160212
C	-5.000426	-3.800563	1.006280
C	-2.876750	-1.133918	2.929048
C	-5.291990	-1.145560	3.135964
C	-6.232945	0.942940	0.454348
C	-6.757339	-1.116600	-0.712288
C	-4.984184	1.144917	-2.744701
C	-5.511346	3.415336	-2.107093
C	-3.493501	3.182184	1.938690
C	-5.411586	1.821670	1.347322
C	-1.445347	3.573545	-1.981003
C	-2.827268	4.985702	-0.593585
H	-3.571437	-2.652575	-1.855874
C	-4.179799	-4.678765	-1.513959
C	-5.130532	-5.144965	0.656799

H	-5.312623	-3.474975	1.992478
C	-2.763046	-1.201318	4.319272
H	-1.989356	-1.067880	2.303207
C	-5.172903	-1.212129	4.524151
H	-6.276619	-1.116396	2.678839
C	-7.469604	1.451591	0.029339
H	-6.501630	-2.128648	-1.003150
C	-7.969359	-0.583683	-1.146591
H	-4.400443	0.242749	-2.597645
C	-5.972083	1.191781	-3.730680
H	-5.334274	4.287028	-1.484954
C	-6.495883	3.463139	-3.091974
H	-2.534875	3.618876	1.685202
C	-3.998199	3.372274	3.222087
C	-5.918714	2.050711	2.637206
H	-1.179204	2.586060	-2.337642
C	-0.741414	4.691337	-2.429804
H	-3.637849	5.117345	0.115683
C	-2.114936	6.102574	-1.031675
H	-3.842660	-5.010315	-2.491077
C	-4.722910	-5.587677	-0.605224
H	-5.549879	-5.847395	1.371960
C	-3.907394	-1.240644	5.117707
H	-1.775283	-1.204947	4.770674
H	-6.066784	-1.236708	5.141869
C	-8.330286	0.709776	-0.775361
H	-8.627584	-1.182975	-1.769328
H	-6.149914	0.321753	-4.356472
C	-6.728604	2.350442	-3.906335
H	-7.081103	4.368763	-3.226392
C	-5.228808	2.817452	3.571339
H	-3.428962	3.952727	3.942403
H	0.070689	4.565621	-3.140554
C	-1.070579	5.959442	-1.950204
H	-2.378900	7.087905	-0.657178
H	-4.822179	-6.635974	-0.873226
H	-3.816847	-1.286411	6.199775
H	-9.268895	1.141395	-1.110175
H	-7.496306	2.389137	-4.674245
H	-5.639374	2.962224	4.566296
H	-0.517468	6.831727	-2.286905
H	-2.564174	-1.202558	-2.720399
C	-0.444542	-1.068679	-2.470905
C	0.787095	-0.351915	-2.704643
H	1.622734	-1.024792	-2.886069
C	0.733884	0.760301	-3.737844
H	0.260902	0.395945	-4.664365
H	0.155669	1.634173	-3.428080
H	1.736010	1.108243	-4.003821
H	-6.862480	1.587924	2.909174
H	-7.738601	2.462150	0.321786
Pd	1.905644	0.173475	-0.675673
P	2.146456	-1.799213	0.712118
P	4.322202	0.369455	-0.994655
C	1.923991	-1.134973	2.437826
C	1.044809	-3.280811	0.794133
C	3.823958	-2.585917	0.843865
C	5.708591	0.176937	0.206722
C	4.399572	-1.079003	-2.157642
C	4.970973	1.799085	-1.987268
C	2.490825	0.107528	2.758454
C	1.246747	-1.836778	3.447844
C	-0.340658	-3.041428	0.798950
C	1.515668	-4.592214	0.946499
C	4.602432	-2.867241	-0.300790
C	4.290409	-3.014762	2.099792
C	5.643632	0.949948	1.376372
C	6.844986	-0.612449	-0.020727
C	4.320491	-0.835381	-3.539076
C	4.317179	-2.424439	-1.705779
C	4.116727	2.433698	-2.905511
C	6.263164	2.323266	-1.813718
H	2.981093	0.685093	1.985983
C	2.407183	0.627801	4.050270

C	1.147662	-1.309330	4.737898
H	0.793298	-2.798156	3.237204
C	-1.229142	-4.099723	0.984875
H	-0.698735	-2.017756	0.698570
C	0.617300	-5.651069	1.115224
H	2.580534	-4.796883	0.947778
C	5.772428	-3.635393	-0.145110
H	3.705663	-2.795572	2.985661
C	5.473101	-3.736063	2.238332
H	4.791202	1.596799	1.551367
C	6.680584	0.920682	2.308647
H	6.923997	-1.193225	-0.933289
C	7.875310	-0.654446	0.919334
H	4.402042	0.178426	-3.910635
C	4.127711	-1.864501	-4.459070
C	4.124110	-3.444288	-2.650515
H	3.109478	2.061155	-3.032362
C	4.537968	3.543210	-3.636978
H	6.950331	1.865433	-1.112176
C	6.680720	3.442933	-2.537148
H	2.855176	1.593308	4.264780
C	1.731126	-0.078798	5.046402
H	0.620299	-1.871540	5.504345
C	-0.756094	-5.406210	1.143554
H	-2.294229	-3.907765	1.001340
H	0.996907	-6.662680	1.234420
C	6.209708	-4.068624	1.102379
H	5.804999	-4.047145	3.224768
H	6.612925	1.533288	3.203128
C	7.794619	0.108523	2.087093
H	8.743301	-1.282082	0.736842
C	4.013007	-3.178698	-4.012497
H	4.067141	-1.632426	-5.518571
H	3.855960	4.011349	-4.341468
C	5.823532	4.056432	-3.451870
H	7.683440	3.832733	-2.383928
H	1.656166	0.326305	6.051852
H	-1.458795	-6.222895	1.285600
H	7.126586	-4.645278	1.185908
H	8.598899	0.073672	2.816752
H	3.849815	-3.991237	-4.714659
H	6.151824	4.928032	-4.010999
H	4.058500	-4.467919	-2.293787
H	6.363023	-3.860162	-1.028376
H	0.112600	1.107484	2.363587
C	-0.775487	1.131757	1.709277
H	-1.654670	1.134288	2.381870
H	-0.781729	2.124713	1.216016
O	-0.773074	0.075141	0.803967
C	1.351584	2.180204	-0.956096
H	1.356547	2.477626	-1.999622
H	0.337603	2.076469	-0.569216
C	2.258649	2.967020	-0.129285
C	2.102543	3.250392	1.188553
C	3.072112	3.925518	2.049189
C	4.375777	4.277764	1.633008
H	4.698065	4.061037	0.619160
C	5.270393	4.883372	2.511325
H	6.268986	5.136884	2.164799
C	4.898343	5.157301	3.832098
H	5.601305	5.626150	4.514770
C	3.612955	4.818355	4.262505
H	3.308216	5.025082	5.285212
C	2.715645	4.212953	3.384291
H	1.718724	3.948416	3.728317
H	1.190147	2.924318	1.677825
H	3.165166	3.307693	-0.623947

TrMt_SS’^{PdCu}

	Value
Charge	0

	Value
Electronic Energy, BS1 (a.u.)	-5342.076275
Thermal and entropic correction, BS1 (a.u.)	1.287501
Electronic Energy, BS2 (a.u.)	-6786.762338

Molecular Geometry in Cartesian Coordinates

C	1.785582	0.718774	-2.549420
H	2.643499	0.209859	-2.979110
B	0.458963	-1.193925	-3.477791
O	1.570981	-2.014922	-3.536050
O	-0.647318	-1.785186	-4.062521
C	1.228558	-3.189604	-4.288357
C	-0.309021	-3.149112	-4.369519
H	1.608743	-4.075706	-3.771953
H	1.698810	-3.129543	-5.277485
H	-0.779535	-3.802614	-3.624131
H	-0.695322	-3.410098	-5.359224
Cu	2.151949	0.169111	-0.441315
P	4.047788	1.523850	0.039641
P	3.152826	-1.928866	0.099075
C	4.288372	3.045510	-0.984685
C	4.082081	2.183735	1.754148
C	5.696368	0.674663	-0.102755
C	4.532243	-2.495120	-0.994414
C	3.940804	-1.591433	1.741696
C	2.285427	-3.553900	0.295254
C	4.247355	2.929749	-2.386510
C	4.455818	4.322477	-0.425768
C	2.869788	2.251763	2.452272
C	5.267195	2.613072	2.373633
C	6.016196	-0.431371	0.724083
C	6.625457	1.068449	-1.079127
C	4.785141	-1.786316	-2.173371
C	5.294257	-3.637037	-0.705455
C	3.222742	-1.888638	2.916686
C	5.163750	-0.890556	1.868434
C	1.372077	-3.875224	-0.721223
C	2.533341	-4.503801	1.299514
H	4.128959	1.954908	-2.845132
C	4.368405	4.054257	-3.202015
C	4.571852	5.448823	-1.243994
H	4.480590	4.447372	0.650371
C	2.853453	2.726257	3.766500
H	1.955024	1.916313	1.967649
C	5.241951	3.097793	3.679844
H	6.208558	2.558445	1.834612
C	7.241597	-1.087655	0.540546
H	6.415516	1.923401	-1.709669
C	7.827910	0.388225	-1.262699
H	4.189980	-0.909058	-2.396081
C	5.796245	-2.197366	-3.043911
H	5.097105	-4.205437	0.198535
C	6.303044	-4.048507	-1.573944
H	2.260958	-2.380151	2.845984
C	3.709188	-1.562000	4.179365
C	5.651673	-0.594289	3.152229
H	1.192389	-3.160004	-1.513410
C	0.698958	-5.096241	-0.717609
H	3.257947	-4.301814	2.080489
C	1.851240	-5.722237	1.306869
H	4.332153	3.939688	-4.281606
C	4.525213	5.320321	-2.632751
H	4.694021	6.428334	-0.790041
C	4.033020	3.147901	4.380603
H	1.916922	2.746998	4.310719
H	6.163293	3.425997	4.153540
C	8.138664	-0.700592	-0.451652
H	8.517585	0.716771	-2.035066
H	5.987603	-1.632489	-3.952068
C	6.557209	-3.327802	-2.745292

H	6.891042	-4.931478	-1.338461
C	4.941098	-0.921814	4.302598
H	3.121930	-1.806967	5.059796
H	-0.018120	-5.316553	-1.504079
C	0.927345	-6.020565	0.303266
H	2.046714	-6.439882	2.099231
H	4.610637	6.197844	-3.267240
H	4.013704	3.511244	5.404726
H	9.068949	-1.244364	-0.585972
H	7.344863	-3.648914	-3.421354
H	5.336792	-0.661775	5.279912
H	0.391067	-6.965158	0.317443
H	1.893962	1.790339	-2.392582
C	0.508156	0.223817	-2.827570
C	-0.674746	0.980666	-2.553594
H	-0.439417	1.982546	-2.192779
H	6.596676	-0.066532	3.233396
H	7.474476	-1.931997	1.182083
Pd	-1.853193	0.151554	-0.572165
P	-2.220352	2.273025	0.451049
P	-4.152411	-0.329076	-0.836974
C	-1.753081	2.071697	2.228513
C	-1.242185	3.714902	-0.122798
C	-3.929421	2.972943	0.684209
C	-5.337237	-0.375186	0.565119
C	-4.789499	0.958251	-2.015739
C	-4.532123	-1.924624	-1.695508
C	-2.369283	1.028739	2.937744
C	-0.910902	2.953406	2.918248
C	0.134863	3.512779	-0.331750
C	-1.813693	4.956106	-0.435441
C	-4.928957	2.914071	-0.309969
C	-4.232417	3.620941	1.898282
C	-4.937458	-1.088767	1.706563
C	-6.608742	0.212203	0.536208
C	-5.060864	0.582438	-3.342497
C	-4.823461	2.341532	-1.694320
C	-3.710959	-2.335300	-2.762159
C	-5.566499	-2.775743	-1.277139
H	-3.025600	0.342897	2.416188
C	-2.159046	0.870484	4.307017
C	-0.713624	2.802973	4.294538
H	-0.423074	3.768088	2.393365
C	0.916176	4.543020	-0.852556
H	0.570186	2.547084	-0.082818
C	-1.022111	5.983423	-0.955443
H	-2.872797	5.125915	-0.270588
C	-6.183085	3.500540	-0.040792
H	-3.473734	3.691312	2.667197
C	-5.479474	4.183994	2.148887
H	-3.959915	-1.557858	1.730045
C	-5.791478	-1.211395	2.801692
H	-6.935884	0.748969	-0.347842
C	-7.454407	0.108143	1.641594
H	-5.082099	-0.466211	-3.607540
C	-5.273162	1.519422	-4.351724
C	-5.005365	3.270660	-2.733630
H	-2.891881	-1.707783	-3.092417
C	-3.927241	-3.558653	-3.394963
H	-6.212266	-2.485824	-0.456079
C	-5.768866	-4.008431	-1.902709
H	-2.642502	0.050119	4.831007
C	-1.330082	1.761752	4.992559
H	-0.086340	3.514650	4.824931
C	0.341812	5.777355	-1.167843
H	1.971848	4.377421	-1.025434
H	-1.474739	6.942561	-1.192362
C	-6.468077	4.121399	1.169333
H	-5.670698	4.669603	3.101436
H	-5.470324	-1.783163	3.667509
C	-7.049280	-0.603174	2.774220
H	-8.433270	0.578761	1.613612
C	-5.204625	2.877287	-4.055096
H	-5.469805	1.181821	-5.365146

H	-3.286537	-3.852214	-4.221809
C	-4.952276	-4.404437	-2.963117
H	-6.571439	-4.655597	-1.559894
H	-1.169043	1.650914	6.061665
H	0.959150	6.571351	-1.579629
H	-7.450495	4.551676	1.341196
H	-7.713725	-0.686391	3.629694
H	-5.331836	3.625283	-4.832165
H	-5.112917	-5.362861	-3.448804
H	-5.013816	4.326947	-2.481722
H	-6.949181	3.448505	-0.808243
H	-0.436298	-0.329755	2.328205
C	0.541775	-0.300064	1.827129
H	1.275085	0.034114	2.582936
H	0.802398	-1.348804	1.599427
O	0.531407	0.504366	0.689784
C	-1.159084	-1.838668	-0.754259
H	-1.251159	-2.172451	-1.783974
H	-0.124528	-1.648371	-0.473317
C	-1.919973	-2.662362	0.183115
C	-1.596100	-2.974937	1.463990
C	-2.453030	-3.668299	2.427071
C	-3.654606	-4.326152	2.080468
H	-3.958151	-4.382015	1.039436
C	-4.466592	-4.901310	3.054437
H	-5.388510	-5.395628	2.757930
C	-4.108478	-4.845804	4.406148
H	-4.747229	-5.292456	5.162996
C	-2.916456	-4.211973	4.768349
H	-2.622206	-4.162363	5.813868
C	-2.101410	-3.638049	3.793950
H	-1.180997	-3.138039	4.087397
H	-0.642812	-2.637006	1.854600
H	-2.876283	-3.019584	-0.191088
C	-1.777351	1.033055	-3.603176
H	-1.395858	1.418870	-4.565277
H	-2.225727	0.062304	-3.818472
H	-2.571282	1.709960	-3.287954

TrMt_SR’^{PdCu}

	Value
Charge	0
Electronic Energy, BS1 (a.u.)	-5342.0692227
Thermal and entropic correction, BS1 (a.u.)	1.2882797000002029
Electronic Energy, BS2 (a.u.)	-6786.753289

Molecular Geometry in Cartesian Coordinates

C	-1.911212	-0.624150	-2.657340
H	-2.078935	0.338745	-3.139112
B	-0.417574	-2.678395	-2.520224
O	-1.454276	-3.557468	-2.766642
O	0.772881	-3.326213	-2.286383
C	-0.958301	-4.896707	-2.614535
C	0.570747	-4.734413	-2.459816
H	-1.235958	-5.485794	-3.495301
H	-1.416876	-5.346740	-1.729213
H	1.118939	-5.061312	-3.352218
H	0.963909	-5.264670	-1.590015
Cu	-2.221720	-0.047960	-0.585889
P	-4.040068	-1.072032	0.557259
P	-3.285514	2.111405	-0.773093
C	-4.339575	-2.867022	0.225965
C	-3.945958	-1.029363	2.395080
C	-5.715246	-0.346597	0.197086
C	-4.722819	2.209233	-1.937859
C	-3.993194	2.511365	0.885602
C	-2.372757	3.622177	-1.318919
C	-4.012044	-3.354955	-1.046865

C	-4.863717	-3.756664	1.178083
C	-2.671613	-1.016516	2.980102
C	-5.082391	-1.031256	3.219927
C	-6.048821	0.987501	0.542811
C	-6.656673	-1.104033	-0.520950
C	-5.051524	1.079248	-2.696323
C	-5.467678	3.386209	-2.109685
C	-3.253688	3.307991	1.781016
C	-5.176770	1.894459	1.358118
C	-1.418187	3.415893	-2.326736
C	-2.622140	4.935996	-0.887576
H	-3.563800	-2.694725	-1.775891
C	-4.213345	-4.697890	-1.369280
C	-5.048714	-5.103071	0.860433
H	-5.111707	-3.406495	2.173904
C	-2.542326	-0.995976	4.370458
H	-1.788725	-0.998301	2.345835
C	-4.947939	-1.017432	4.607757
H	-6.072766	-1.034133	2.774780
C	-7.302015	1.495077	0.167353
H	-6.428209	-2.128443	-0.789412
C	-7.886173	-0.573270	-0.906242
H	-4.483786	0.166196	-2.558022
C	-6.116010	1.117553	-3.599606
H	-5.219315	4.274385	-1.537204
C	-6.528915	3.425135	-3.011849
H	-2.319364	3.750153	1.457075
C	-3.685518	3.540373	3.084013
C	-5.609738	2.163549	2.666789
H	-1.214883	2.407299	-2.669808
C	-0.720207	4.489049	-2.882015
H	-3.366742	5.129437	-0.122809
C	-1.913396	6.007018	-1.433110
H	-3.945597	-5.053132	-2.359369
C	-4.728121	-5.577035	-0.414976
H	-5.443429	-5.782393	1.611065
C	-3.675467	-0.995611	5.184907
H	-1.551377	-0.962668	4.808817
H	-5.834511	-1.015416	5.236379
C	-8.212771	0.736685	-0.562987
H	-8.583143	-1.186461	-1.470543
H	-6.367561	0.229167	-4.172038
C	-6.855879	2.289330	-3.759185
H	-7.101070	4.341066	-3.133267
C	-4.879591	2.975379	3.529647
H	-3.086981	4.160881	3.744927
H	0.019135	4.305305	-3.656368
C	-0.959558	5.787357	-2.430414
H	-2.111102	7.015921	-1.081180
H	-4.872998	-6.625939	-0.658879
H	-3.569027	-0.972664	6.266261
H	-9.163786	1.167949	-0.861238
H	-7.684275	2.319976	-4.461713
H	-5.232049	3.149788	4.541994
H	-0.408379	6.623319	-2.851708
H	-2.717237	-1.335797	-2.811750
C	-0.595502	-1.125922	-2.626792
C	0.549047	-0.299534	-2.827301
H	0.277101	0.728349	-3.082245
H	-6.527897	1.697816	3.011201
H	-7.544503	2.518272	0.437850
Pd	1.821609	0.132219	-0.714945
P	2.039950	-1.775292	0.774937
P	4.199158	0.286657	-1.018483
C	1.689158	-1.077692	2.459736
C	1.023891	-3.317285	0.817120
C	3.738679	-2.481885	1.094147
C	5.409836	0.370704	0.367515
C	4.602223	-1.253264	-1.975906
C	4.827915	1.669682	-2.085668
C	2.172319	0.203486	2.760804
C	1.091978	-1.824415	3.487077
C	-0.371480	-3.178251	0.725930
C	1.573629	-4.592787	1.010340

C	4.605937	-2.851319	0.043932
C	4.134950	-2.767607	2.414544
C	5.147203	1.290808	1.393258
C	6.600842	-0.367963	0.405639
C	4.859970	-1.148043	-3.351941
C	4.457620	-2.552722	-1.418033
C	4.059873	2.101075	-3.181464
C	6.019722	2.356459	-1.798841
H	2.598900	0.808008	1.971235
C	2.097584	0.717532	4.055601
C	1.016228	-1.310854	4.784415
H	0.706884	-2.818184	3.287915
C	-1.192579	-4.297141	0.859402
H	-0.796379	-2.188883	0.571541
C	0.744815	-5.712885	1.127412
H	2.647220	-4.721416	1.084793
C	5.789393	-3.551412	0.356893
H	3.491547	-2.485058	3.237195
C	5.326243	-3.425179	2.707232
H	4.249999	1.896196	1.354959
C	6.042047	1.454222	2.450001
H	6.834137	-1.060481	-0.395457
C	7.487735	-0.218019	1.472525
H	5.022634	-0.172350	-3.792621
C	4.880420	-2.264157	-4.186888
C	4.440598	-3.659176	-2.281780
H	3.130113	1.597892	-3.412502
C	4.468442	3.177414	-3.967772
H	6.637388	2.053790	-0.961659
C	6.421729	3.442585	-2.579486
H	2.481002	1.714138	4.255116
C	1.522813	-0.041827	5.075618
H	0.568294	-1.912879	5.570586
C	-0.640297	-5.565969	1.060335
H	-2.266018	-4.182882	0.803737
H	1.186970	-6.693925	1.280765
C	6.153717	-3.841070	1.666743
H	5.593979	-3.619969	3.741736
H	5.822790	2.180392	3.227232
C	7.209477	0.689287	2.498188
H	8.398825	-0.809398	1.499281
C	4.622582	-3.525482	-3.656926
H	5.074238	-2.137666	-5.248038
H	3.855944	3.488989	-4.809260
C	5.649553	3.858196	-3.665241
H	7.344413	3.962015	-2.335599
H	1.466358	0.350500	6.087249
H	-1.292296	-6.429268	1.162537
H	7.080821	-4.369760	1.869398
H	7.903359	0.803601	3.326398
H	4.595628	-4.402377	-4.297346
H	5.963273	4.705501	-4.268270
H	4.308774	-4.646184	-1.848091
H	6.449643	-3.835680	-0.456875
H	0.161421	1.324802	2.065574
C	-0.700828	1.212659	1.390562
H	-1.592719	1.156050	2.036262
H	-0.795010	2.165088	0.835244
O	-0.568728	0.111450	0.545821
C	1.395346	2.129634	-1.190924
H	1.518992	2.315480	-2.256136
H	0.344569	2.121559	-0.910462
C	2.245680	2.974707	-0.355254
C	1.940110	3.451594	0.877591
C	2.831666	4.185930	1.774825
C	4.125739	4.622620	1.414571
H	4.494099	4.451944	0.407661
C	4.948736	5.264590	2.336087
H	5.941699	5.585445	2.032039
C	4.510232	5.496239	3.644548
H	5.156917	5.995950	4.360143
C	3.229445	5.080551	4.017519
H	2.872432	5.255781	5.029078
C	2.403681	4.439083	3.095775

H	1.411091	4.112179	3.396333
H	0.957472	3.232308	1.281052
H	3.230321	3.194691	-0.760952
C	1.654272	-0.831711	-3.731925
H	1.273025	-1.038255	-4.747952
H	2.458721	-0.106715	-3.853029
H	2.098404	-1.753009	-3.359663

TrMt-RR^{PdCu}

	Value
Charge	0
Electronic Energy, BS1 (a.u.)	-5342.086353
Thermal and entropic correction, BS1 (a.u.)	1.286923
Electronic Energy, BS2 (a.u.)	-6786.7717868

Molecular Geometry in Cartesian Coordinates

C	-1.760939	-0.612551	-2.521980
H	-2.658167	-0.116202	-2.879506
B	-0.604534	1.519399	-3.128910
O	-1.749194	2.286304	-3.039441
O	0.445023	2.215505	-3.700112
C	-1.500168	3.545161	-3.685189
C	0.031737	3.584848	-3.853861
H	-1.884569	4.355821	-3.060217
H	-2.025925	3.561055	-4.647436
H	0.517551	4.188603	-3.077164
H	0.345689	3.956265	-4.833808
Cu	-2.233249	-0.110387	-0.343320
P	-4.145438	-1.464600	0.002119
P	-3.252266	2.006373	0.214263
C	-4.366743	-2.952097	-1.077846
C	-4.184421	-2.177099	1.697209
C	-5.795594	-0.618541	-0.147223
C	-4.603436	2.609787	-0.900603
C	-4.092702	1.585681	1.815015
C	-2.415219	3.621876	0.581741
C	-4.253135	-2.792774	-2.470466
C	-4.611549	-4.238814	-0.572416
C	-2.973671	-2.247339	2.399511
C	-5.362638	-2.654505	2.294118
C	-6.136393	0.462803	0.702778
C	-6.697574	-0.979262	-1.161681
C	-4.835558	1.915131	-2.093350
C	-5.374807	3.746940	-0.616189
C	-3.406414	1.844531	3.017498
C	-5.315045	0.878097	1.884755
C	-1.318012	3.964198	-0.220696
C	-2.851130	4.547741	1.546168
H	-4.082920	-1.808266	-2.889382
C	-4.371508	-3.885365	-3.329061
C	-4.721307	-5.334003	-1.432761
H	-4.703108	-4.396781	0.496004
C	-2.947084	-2.793051	3.685301
H	-2.067202	-1.858037	1.939889
C	-5.328747	-3.199999	3.576467
H	-6.304551	-2.592659	1.756454
C	-7.351595	1.132851	0.502326
H	-6.472262	-1.815418	-1.812026
C	-7.890563	-0.286456	-1.360226
H	-4.234929	1.042233	-2.317465
C	-5.831647	2.332814	-2.977498
H	-5.203259	4.307508	0.296804
C	-6.366010	4.168866	-1.500956
H	-2.449943	2.351421	2.987694
C	-3.922395	1.468165	4.254408
C	-5.834037	0.531087	3.143698
H	-0.978534	3.266801	-0.971600
C	-0.659025	5.183678	-0.057233

H	-3.699783	4.317862	2.181384
C	-2.191856	5.766460	1.711411
H	-4.278332	-3.737006	-4.401129
C	-4.597767	-5.162897	-2.811985
H	-4.902199	-6.322275	-1.018943
C	-4.118815	-3.268755	4.274546
H	-2.008064	-2.830646	4.227178
H	-6.244533	-3.565222	4.033489
C	-8.219495	0.781186	-0.528293
H	-8.559219	-0.587753	-2.161711
H	-6.006382	1.775825	-3.893816
C	-6.597979	3.461304	-2.684036
H	-6.958097	5.049666	-1.267100
C	-5.153149	0.816958	4.322585
H	-3.360258	1.685120	5.158243
H	0.201485	5.415501	-0.678271
C	-1.090769	6.087624	0.913621
H	-2.539910	6.465413	2.467195
H	-4.678358	-6.016275	-3.479247
H	-4.093906	-3.687156	5.277221
H	-9.141808	1.335243	-0.675654
H	-7.372352	3.789386	-3.372108
H	-5.571736	0.519150	5.279413
H	-0.573069	7.032643	1.051538
H	-1.811891	-1.694898	-2.445913
C	-0.535557	0.026420	-2.699323
C	0.740401	-0.616762	-2.625371
H	1.530521	-0.058023	-3.125741
C	0.818081	-2.108355	-2.882706
H	0.215195	-2.688818	-2.183281
H	0.444253	-2.347918	-3.893840
H	1.844450	-2.474571	-2.815695
H	-6.779367	-0.001263	3.181166
H	-7.599556	1.959062	1.161667
Pd	1.850425	-0.191304	-0.516101
P	2.229777	-2.330888	0.483326
P	4.180358	0.221336	-0.929442
C	1.942116	-1.949340	2.270782
C	1.160323	-3.781470	0.147882
C	3.932538	-3.058322	0.576499
C	5.633884	0.130323	0.201192
C	4.450710	-1.099511	-2.211978
C	4.516201	1.824053	-1.792519
C	2.741971	-0.943840	2.839390
C	0.983991	-2.582168	3.071433
C	-0.227077	-3.549171	0.111434
C	1.657585	-5.057237	-0.149879
C	4.824092	-3.035940	-0.516825
C	4.334840	-3.696527	1.764948
C	5.527898	0.793848	1.434594
C	6.843368	-0.497039	-0.128775
C	4.362826	-0.751326	-3.570517
C	4.537351	-2.478602	-1.880420
C	3.580519	2.310002	-2.724750
C	5.618493	2.632942	-1.472677
H	3.497642	-0.459052	2.234999
C	2.585982	-0.571275	4.173124
C	0.829924	-2.208102	4.409492
H	0.353908	-3.361979	2.659158
C	-1.095675	-4.594217	-0.196899
H	-0.600460	-2.547011	0.322096
C	0.779301	-6.093086	-0.479295
H	2.725829	-5.247184	-0.125855
C	6.078919	-3.659344	-0.375839
H	3.650762	-3.731175	2.605360
C	5.585514	-4.293359	1.890822
H	4.613268	1.319252	1.686537
C	6.600710	0.817638	2.325383
H	6.951564	-0.991103	-1.088522
C	7.911160	-0.488030	0.770082
H	4.324442	0.291929	-3.855169
C	4.317909	-1.710477	-4.580854
C	4.496267	-3.428806	-2.915185
H	2.702931	1.728454	-2.976264

C	3.746115	3.560114	-3.319421
H	6.354695	2.296299	-0.752736
C	5.775135	3.890499	-2.060311
H	3.217040	0.210130	4.587623
C	1.621990	-1.201509	4.963943
H	0.093043	-2.715333	5.025759
C	-0.597214	-5.864349	-0.500259
H	-2.162721	-4.411957	-0.221622
H	1.174187	-7.077969	-0.714513
C	6.466279	-4.274272	0.809681
H	5.864586	-4.774032	2.824047
H	6.501102	1.348946	3.267410
C	7.792534	0.166354	1.998755
H	8.838810	-0.989008	0.506766
C	4.369303	-3.062214	-4.252910
H	4.241032	-1.394028	-5.617070
H	3.009068	3.908441	-4.037208
C	4.840765	4.361085	-2.984291
H	6.632977	4.500865	-1.791228
H	1.493351	-0.915926	6.004216
H	-1.282201	-6.667943	-0.757418
H	7.447465	-4.733876	0.887116
H	8.627182	0.173801	2.694295
H	4.322193	-3.824924	-5.024525
H	4.964268	5.340482	-3.438170
H	4.564123	-4.479763	-2.650218
H	6.761213	-3.642311	-1.220382
H	0.340435	0.177614	2.518892
C	-0.611383	0.304369	1.979781
H	-1.411012	0.064285	2.704500
H	-0.711646	1.386149	1.765994
O	-0.668364	-0.486169	0.831832
C	1.174992	1.806315	-0.519002
H	0.128197	1.538963	-0.377207
H	1.350228	2.363428	-1.435007
C	1.815181	2.366554	0.662431
C	2.779843	3.322131	0.666015
C	3.542569	3.772109	1.828444
C	3.304984	3.308723	3.142779
H	2.493339	2.612833	3.331792
C	4.104494	3.721431	4.205115
H	3.897795	3.349321	5.205583
C	5.166193	4.609795	3.996114
H	5.788677	4.927679	4.827677
C	5.412597	5.082629	2.704975
H	6.234301	5.771242	2.525347
C	4.612541	4.671660	1.640383
H	4.822566	5.030002	0.635866
H	3.070770	3.764281	-0.284992
H	1.515295	1.928864	1.611578

III-SS^{PdCu}

	Value
Charge	0
Electronic Energy, BS1 (a.u.)	-5342.091743
Thermal and entropic correction, BS1 (a.u.)	1.282948
Electronic Energy, BS2 (a.u.)	-6786.777457

Molecular Geometry in Cartesian Coordinates

C	-1.452191	-0.268297	-2.408851
H	-2.453921	0.090188	-2.623791
B	-0.777059	2.079603	-2.756309
O	-2.057272	2.577609	-2.618270
O	0.105563	3.025145	-3.236889
C	-2.079554	3.917731	-3.136044
C	-0.589506	4.284824	-3.299276
H	-2.606267	4.568190	-2.432501
H	-2.619380	3.920490	-4.090221

H	-0.230917	4.925056	-2.483723
H	-0.370629	4.777188	-4.251179
Cu	-2.572372	-0.021890	-0.075007
P	-4.314349	-1.583701	-0.262782
P	-3.764909	1.889904	0.400074
C	-4.243529	-2.907723	-1.548617
C	-4.431078	-2.524504	1.313168
C	-6.014516	-0.871694	-0.484318
C	-5.038879	2.508077	-0.787906
C	-4.693647	1.214564	1.853736
C	-3.075281	3.493305	1.013916
C	-3.920609	-2.538907	-2.866205
C	-4.440039	-4.267606	-1.261234
C	-3.276484	-2.612889	2.104895
C	-5.615214	-3.151347	1.733636
C	-6.529990	0.073770	0.438254
C	-6.778326	-1.186012	-1.619896
C	-5.085441	1.935102	-2.064557
C	-5.927327	3.547637	-0.474733
C	-4.108357	1.357803	3.126228
C	-5.853084	0.414138	1.731489
C	-1.921983	3.968103	0.374251
C	-3.661282	4.283511	2.017615
H	-3.768834	-1.494513	-3.112803
C	-3.793098	-3.500935	-3.867277
C	-4.305743	-5.231046	-2.263643
H	-4.680676	-4.582220	-0.252198
C	-3.316345	-3.320014	3.309336
H	-2.364843	-2.109510	1.778771
C	-5.644544	-3.859604	2.934330
H	-6.513130	-3.077107	1.126613
C	-7.779713	0.657294	0.188488
H	-6.410570	-1.916534	-2.330901
C	-8.009249	-0.577902	-1.862053
H	-4.389876	1.141963	-2.311610
C	-6.013523	2.378160	-3.007807
H	-5.898316	4.013116	0.505102
C	-6.852806	3.993260	-1.417474
H	-3.188726	1.921517	3.230206
C	-4.678241	0.788005	4.261256
C	-6.430943	-0.125229	2.893114
H	-1.472370	3.372002	-0.406629
C	-1.354513	5.191816	0.732510
H	-4.552137	3.940724	2.533465
C	-3.094313	5.507193	2.376137
H	-3.536606	-3.194728	-4.877345
C	-3.979071	-4.852564	-3.566961
H	-4.452869	-6.279725	-2.020732
C	-4.494279	-3.941958	3.725580
H	-2.423767	-3.368140	3.925719
H	-6.564338	-4.340658	3.256063
C	-8.513125	0.352966	-0.956193
H	-8.570804	-0.838036	-2.754900
H	-6.043692	1.918006	-3.991472
C	-6.898850	3.407923	-2.686037
H	-7.538110	4.797097	-1.162569
C	-5.859363	0.055464	4.148853
H	-4.197186	0.917321	5.226434
H	-0.449426	5.529222	0.235161
C	-1.938037	5.963922	1.737882
H	-3.556041	6.103135	3.158665
H	-3.867514	-5.604439	-4.342961
H	-4.520957	-4.486374	4.665830
H	-9.467507	0.839313	-1.135104
H	-7.621192	3.755205	-3.419665
H	-6.319645	-0.391569	5.024994
H	-1.494395	6.912614	2.026393
H	-1.333512	-1.339249	-2.282246
C	-0.405102	0.601196	-2.444657
C	1.019521	0.201719	-2.347692
H	1.649811	0.992028	-2.764528
C	1.350477	-1.122915	-3.020801
H	0.756615	-1.952293	-2.638739
H	1.152410	-1.053785	-4.102971

H	2.397370	-1.382234	-2.893915
H	-7.328859	-0.726556	2.789887
H	-8.165019	1.378159	0.903363
Pd	1.682440	0.269392	-0.307463
P	2.325206	-1.962880	0.330894
P	5.147904	0.632894	-0.605781
C	1.841268	-2.074592	2.118117
C	1.376155	-3.328036	-0.450844
C	4.063183	-2.653086	0.431973
C	6.931680	0.302640	-0.228854
C	4.911027	-0.429398	-2.110908
C	5.207282	2.346823	-1.298314
C	2.501704	-1.224705	3.021246
C	0.884458	-2.970459	2.611030
C	-0.019922	-3.166683	-0.536853
C	1.979489	-4.454580	-1.022475
C	5.030325	-2.479174	-0.584932
C	4.429106	-3.399391	1.568761
C	7.297277	0.199359	1.121083
C	7.913893	0.118753	-1.215783
C	4.839624	0.069534	-3.420483
C	4.798874	-1.825419	-1.914536
C	4.066776	2.859267	-1.947467
C	6.286690	3.215278	-1.067924
H	3.243308	-0.520034	2.658331
C	2.227359	-1.288105	4.386931
C	0.594733	-3.014967	3.977207
H	0.361324	-3.639326	1.938690
C	-0.787907	-4.130495	-1.188520
H	-0.484557	-2.288406	-0.086074
C	1.202811	-5.406436	-1.689478
H	3.053329	-4.593034	-0.952033
C	6.318026	-3.016469	-0.407037
H	3.699358	-3.578052	2.348317
C	5.709117	-3.924440	1.729229
H	6.543036	0.332252	1.891594
C	8.617968	-0.081454	1.479980
H	7.642185	0.189153	-2.265329
C	9.231253	-0.167992	-0.857926
H	4.935417	1.134454	-3.597407
C	4.660533	-0.779967	-4.513622
C	4.635284	-2.668688	-3.023313
H	3.208233	2.216788	-2.108307
C	4.017421	4.186700	-2.370135
H	7.177856	2.852439	-0.565990
C	6.228723	4.550289	-1.477920
H	2.765362	-0.637130	5.067884
C	1.266202	-2.179695	4.870006
H	-0.145994	-3.721149	4.342173
C	-0.180063	-5.245365	-1.774688
H	-1.861510	-4.004688	-1.253484
H	1.682158	-6.272929	-2.137526
C	6.668516	-3.721339	0.739469
H	5.949105	-4.489178	2.625757
H	8.886170	-0.162169	2.529811
C	9.585215	-0.269790	0.491479
H	9.982147	-0.313347	-1.630117
C	4.557922	-2.155902	-4.317697
H	4.610015	-0.362284	-5.515293
H	3.127395	4.550684	-2.876071
C	5.098421	5.040572	-2.133327
H	7.074704	5.205240	-1.286959
H	1.047481	-2.225357	5.933351
H	-0.786828	-5.982162	-2.293950
H	7.676613	-4.109403	0.852516
H	10.611135	-0.496869	0.768533
H	4.418936	-2.826092	-5.160905
H	5.058594	6.077612	-2.454726
H	4.566865	-3.739807	-2.858643
H	7.054477	-2.854478	-1.187548
H	-0.037033	-0.203979	2.761473
C	-0.983742	0.041402	2.253848
H	-1.792864	-0.245885	2.956268
H	-1.029578	1.147459	2.180841

O	-1.081070	-0.597838	1.019570
C	0.995251	2.238446	-0.137697
H	0.969968	2.842600	-1.039338
H	0.020576	2.057458	0.312465
C	2.095479	2.492623	0.752823
C	2.164848	2.077494	2.059128
C	3.314678	2.247310	2.941499
C	4.594677	2.635630	2.485062
H	4.756012	2.831696	1.431557
C	5.663373	2.764693	3.367709
H	6.637674	3.055247	2.983349
C	5.495071	2.512185	4.733815
H	6.333011	2.609125	5.418305
C	4.234377	2.137966	5.206179
H	4.084002	1.947479	6.265840
C	3.162872	2.008894	4.324049
H	2.185738	1.718524	4.699962
H	1.277462	1.637850	2.507420
H	2.960066	2.993618	0.326022

V^{Pd}

	Value
Charge	0
Electronic Energy, BS1 (a.u.)	-2957.985786
Thermal and entropic correction, BS1 (a.u.)	0.756174
Electronic Energy, BS2 (a.u.)	-2958.828073

Molecular Geometry in Cartesian Coordinates

P	0.904338	0.548103	0.788321
P	-1.956268	-1.001753	-0.133892
C	2.407449	1.609312	0.876979
C	-0.029774	1.310335	2.188009
C	1.386790	-1.155181	1.285510
C	-1.476230	-2.705258	-0.669055
C	-1.997076	-1.250643	1.724073
C	-3.745196	-0.884757	-0.555244
C	2.380199	2.782563	0.106689
C	3.490707	1.379692	1.735578
C	-1.146662	2.103576	1.894658
C	0.403498	1.204079	3.517062
C	0.466814	-2.072057	1.848696
C	2.632941	-1.633248	0.842252
C	-0.530663	-2.871958	-1.689840
C	-2.016511	-3.843667	-0.047597
C	-3.174372	-0.999172	2.448051
C	-0.859993	-1.704374	2.440796
C	-4.431185	0.289832	-0.198303
C	-4.439799	-1.883554	-1.254188
H	1.547816	2.957932	-0.568077
C	3.424148	3.703933	0.186612
C	4.539215	2.299117	1.806250
H	3.527478	0.480908	2.342351
C	-1.823797	2.776807	2.914253
H	-1.494020	2.195168	0.870617
C	-0.277068	1.870300	4.534995
H	1.268785	0.592750	3.756658
C	0.843761	-3.421575	1.959627
H	3.337817	-0.950884	0.388600
C	2.982507	-2.976260	0.953924
H	-0.101952	-1.992828	-2.163175
C	-0.125420	-4.150632	-2.078703
H	-2.743277	-3.730434	0.751319
C	-1.616929	-5.119614	-0.441831
H	-4.066713	-0.674103	1.930542
C	-3.248918	-1.175359	3.828558
C	-0.963271	-1.898225	3.828606
H	-3.902152	1.094922	0.301322
C	-5.792341	0.430067	-0.470611

H	-3.930004	-2.789561	-1.561770
C	-5.792709	-1.723286	-1.560579
H	3.397297	4.603325	-0.422571
C	4.509165	3.462373	1.033841
H	5.380292	2.103304	2.465347
C	-1.392029	2.659263	4.235913
H	-2.688679	3.387148	2.669349
H	0.061372	1.772901	5.562810
C	2.079667	-3.880052	1.512288
H	3.953564	-3.302409	0.594453
H	0.616907	-4.263510	-2.863817
C	-0.666677	-5.275470	-1.455767
H	-2.041507	-5.992353	0.046613
C	-2.136079	-1.632051	4.528199
H	-4.180775	-0.964368	4.345383
H	-6.307987	1.337977	-0.171336
C	-6.476373	-0.574583	-1.156837
H	-6.313760	-2.505006	-2.106239
H	5.326804	4.175416	1.090757
H	-1.919973	3.177972	5.031231
H	2.330694	-4.933272	1.598394
H	-0.349397	-6.270510	-1.755213
H	-2.175662	-1.780027	5.603442
H	-7.532289	-0.458878	-1.384283
H	-0.085955	-2.250814	4.362797
H	0.133317	-4.119409	2.391534
C	1.344892	0.768342	-2.580377
H	0.925675	0.421498	-3.527916
H	1.567419	1.836700	-2.648708
C	2.512704	-0.054759	-2.238582
C	3.737382	0.397083	-1.881531
C	4.907044	-0.413810	-1.533826
C	5.002604	-1.797313	-1.798454
H	4.193956	-2.301824	-2.318293
C	6.122923	-2.528402	-1.410417
H	6.170205	-3.591947	-1.630897
C	7.187161	-1.903837	-0.750709
H	8.060088	-2.476898	-0.451254
C	7.115700	-0.533422	-0.490616
H	7.934920	-0.032522	0.018961
C	5.994944	0.199086	-0.878693
H	5.940826	1.261491	-0.657344
H	-4.340377	2.759726	-2.060998
H	-4.252698	1.184817	-3.011535
C	-2.372014	2.040744	-2.436755
C	-3.720888	1.987847	-2.509471
B	-1.734798	3.271131	-1.722391
O	-2.371408	3.960809	-0.706703
O	-0.502996	3.811014	-2.037503
C	-1.477837	4.987545	-0.239375
C	-0.352963	5.033197	-1.293240
H	-1.103258	4.704302	0.750813
H	-2.019825	5.934224	-0.153297
H	0.643667	5.072097	-0.844114
H	-0.459467	5.881050	-1.980568
C	-1.456713	1.009552	-2.993461
Pd	-0.320967	0.465914	-1.240984
H	-0.702920	1.516693	-3.598838
C	-2.081778	-0.124936	-3.791385
H	-2.789307	-0.712713	-3.198906
H	-2.632899	0.251414	-4.668554
H	-1.309918	-0.809527	-4.160795
H	2.353127	-1.133351	-2.286730
H	3.879696	1.472758	-1.799475

V’^{Pd}

	Value
Charge	0
Electronic Energy, BS1 (a.u.)	-2957.988266

	Value
Thermal and entropic correction, BS1 (a.u.)	0.754178
Electronic Energy, BS2 (a.u.)	-2958.830475

Molecular Geometry in Cartesian Coordinates

P	0.610148	1.134373	0.775224
P	-1.464605	-1.418183	0.005597
C	1.726747	2.602025	0.735075
C	-0.619723	1.758518	2.001551
C	1.555101	-0.232508	1.559833
C	-0.339696	-2.877219	-0.145861
C	-1.633828	-1.370293	1.875563
C	-3.113647	-2.038356	-0.532351
C	1.447527	3.570115	-0.242795
C	2.759210	2.838078	1.654820
C	-1.912978	2.065332	1.559838
C	-0.269877	2.023604	3.333368
C	0.930217	-1.290172	2.260957
C	2.910321	-0.362345	1.209920
C	0.846262	-2.770168	-0.884072
C	-0.604912	-4.067493	0.553168
C	-2.899559	-1.422745	2.481771
C	-0.495894	-1.290215	2.720097
C	-4.217574	-1.175654	-0.408051
C	-3.309911	-3.297066	-1.122159
H	0.652780	3.388347	-0.959883
C	2.196167	4.745724	-0.307937
C	3.512628	4.011019	1.580832
H	2.986633	2.103675	2.420733
C	-2.845114	2.625617	2.436740
H	-2.197736	1.864218	0.532135
C	-1.202008	2.577667	4.208914
H	0.729655	1.788480	3.687520
C	1.701027	-2.410548	2.615497
H	3.396076	0.421288	0.645406
C	3.651478	-1.490601	1.547184
H	1.075920	-1.841604	-1.398991
C	1.754289	-3.830771	-0.918704
H	-1.509103	-4.155359	1.148338
C	0.293613	-5.131530	0.501637
H	-3.787922	-1.513243	1.871791
C	-3.061435	-1.388274	3.865846
C	-0.679322	-1.279298	4.112409
H	-4.082907	-0.176824	-0.006509
C	-5.493440	-1.589743	-0.792587
H	-2.473006	-3.971770	-1.260632
C	-4.581246	-3.694215	-1.540657
H	1.976660	5.482250	-1.075917
C	3.234413	4.966073	0.599983
H	4.316573	4.178562	2.292237
C	-2.492008	2.880790	3.762200
H	-3.844523	2.856399	2.078534
H	-0.923672	2.770972	5.241179
C	3.041454	-2.523703	2.257461
H	4.689474	-1.558498	1.236750
H	2.685279	-3.719051	-1.465230
C	1.478565	-5.012523	-0.231398
H	0.076311	-6.047663	1.043933
C	-1.943821	-1.316923	4.691936
H	-4.061386	-1.428634	4.288355
H	-6.334464	-0.912992	-0.672629
C	-5.679135	-2.849759	-1.362625
H	-4.712374	-4.670791	-1.998257
H	3.825001	5.876024	0.543388
H	-3.216586	3.312605	4.446914
H	3.598710	-3.417677	2.521444
H	2.187523	-5.835513	-0.255904
H	-2.050322	-1.292213	5.772492
H	-6.669463	-3.166872	-1.676864
H	0.202182	-1.223906	4.744403
H	1.215829	-3.218644	3.153628

C	1.186505	1.070065	-2.615841
H	0.969822	2.129842	-2.765141
H	1.030201	0.519550	-3.544838
C	2.516348	0.804277	-2.070046
C	3.275153	-0.283330	-2.357364
C	4.587162	-0.605168	-1.799781
C	5.369562	0.316991	-1.069157
H	5.023022	1.339233	-0.947630
C	6.583793	-0.059200	-0.501165
H	7.159862	0.671675	0.060676
C	7.071324	-1.361981	-0.657152
H	8.020395	-1.650962	-0.214906
C	6.327874	-2.279637	-1.403844
H	6.696812	-3.292182	-1.545827
C	5.110247	-1.904207	-1.968642
H	4.540992	-2.627886	-2.547282
H	-4.821283	1.077123	-2.527700
H	-4.157720	-0.487566	-3.238653
C	-2.703029	1.005253	-2.738905
C	-3.953819	0.502094	-2.840964
B	-2.545432	2.461065	-2.201501
O	-3.450741	3.043089	-1.333811
O	-1.519311	3.319070	-2.549628
C	-2.968691	4.357423	-1.001472
C	-1.812852	4.611830	-1.991482
H	-2.626581	4.354007	0.039406
H	-3.782318	5.082312	-1.102003
H	-0.920611	5.012458	-1.501308
H	-2.099711	5.291110	-2.803034
C	-1.466958	0.269455	-3.115154
Pd	-0.353016	0.368442	-1.270775
H	-0.878809	0.912960	-3.771267
C	-1.624039	-1.102325	-3.752836
H	-2.149200	-1.809466	-3.105080
H	-2.192609	-1.047839	-4.695627
H	-0.643033	-1.531398	-3.986067
H	2.904436	1.531730	-1.360750
H	2.863541	-1.023922	-3.044560

VI’^{Pd}

	Value
Charge	0
Electronic Energy, BS1 (a.u.)	-2957.957266
Thermal and entropic correction, BS1 (a.u.)	0.747233
Electronic Energy, BS2 (a.u.)	-2958.803464

Molecular Geometry in Cartesian Coordinates

P	-0.010297	0.186239	0.879826
P	4.567152	0.754603	-1.297557
C	0.536272	1.630021	1.898803
C	0.996297	-1.188411	1.569165
C	0.466846	0.639065	-0.855489
C	4.933875	1.017878	0.500775
C	3.761614	-0.927907	-1.236995
C	6.226699	0.356710	-2.007725
C	0.438031	1.492325	3.295757
C	0.928928	2.866886	1.369294
C	0.350566	-2.409187	1.820828
C	2.349052	-1.045120	1.912739
C	1.473922	0.117443	-1.697459
C	-0.380571	1.647656	-1.365026
C	4.288650	2.091272	1.131223
C	5.746856	0.171518	1.274867
C	4.511654	-2.094108	-1.003903
C	2.357869	-1.040850	-1.357714
C	6.316972	-0.427796	-3.171975
C	7.399844	0.947534	-1.509734
H	0.140228	0.539481	3.725754

C	0.729157	2.563665	4.138277
C	1.210246	3.942350	2.216325
H	1.022784	2.999112	0.297490
C	1.054215	-3.479163	2.376326
H	-0.703453	-2.519545	1.585482
C	3.049830	-2.117079	2.461406
H	2.856879	-0.102469	1.751124
C	1.604889	0.644916	-2.997000
H	-1.184854	2.023647	-0.738478
C	-0.225765	2.164512	-2.646097
H	3.655705	2.752962	0.548405
C	4.425542	2.301743	2.506517
H	6.263747	-0.659293	0.806430
C	5.896328	0.388548	2.643730
H	5.591718	-2.028083	-0.926692
C	3.901673	-3.339513	-0.883460
C	1.756891	-2.305133	-1.245096
H	5.424979	-0.896077	-3.579036
C	7.543519	-0.632362	-3.803346
H	7.360162	1.563462	-0.616507
C	8.626714	0.744202	-2.145196
H	0.652401	2.436898	5.214468
C	1.111107	3.796480	3.600761
H	1.514788	4.893336	1.788026
C	2.406107	-3.336523	2.690882
H	0.543698	-4.419610	2.563847
H	4.099983	-1.993647	2.705839
C	0.780423	1.658249	-3.470798
H	-0.897959	2.940031	-3.000853
H	3.891482	3.119095	2.979917
C	5.226805	1.449097	3.264481
H	6.528068	-0.273619	3.229794
C	2.514724	-3.445483	-1.001340
H	4.505890	-4.222925	-0.697147
H	7.591413	-1.250739	-4.695639
C	8.705007	-0.048195	-3.291525
H	9.523127	1.204750	-1.738614
H	1.329794	4.634527	4.256328
H	2.956672	-4.169211	3.119890
H	0.910539	2.036863	-4.480273
H	5.331494	1.606849	4.334207
H	2.025320	-4.409994	-0.906263
H	9.660541	-0.208178	-3.782868
H	0.679110	-2.382596	-1.351866
H	2.371565	0.228641	-3.643305
C	-4.354785	-0.167364	1.798220
H	-4.053528	0.130439	2.818585
H	-4.801366	-1.164214	1.801882
C	-5.110267	0.841352	1.090953
C	-6.102791	0.587570	0.188836
C	-6.798933	1.565090	-0.644280
C	-6.382838	2.907278	-0.786063
H	-5.499064	3.254541	-0.259182
C	-7.077262	3.794035	-1.605163
H	-6.733201	4.821630	-1.693263
C	-8.202211	3.371507	-2.321126
H	-8.737890	4.064814	-2.963364
C	-8.621111	2.043330	-2.206009
H	-9.489254	1.696335	-2.760749
C	-7.930445	1.156734	-1.382604
H	-8.262123	0.124007	-1.302793
H	-2.048148	-2.231700	-0.262704
H	-1.869588	-0.763864	-1.295861
C	-3.889086	-1.482494	-1.068852
C	-2.516707	-1.305636	-0.603798
B	-4.715652	-2.666296	-0.513500
O	-4.190652	-3.639029	0.313212
O	-6.065826	-2.837239	-0.749186
C	-5.279970	-4.456113	0.775791
C	-6.466534	-4.073269	-0.134528
H	-5.474769	-4.222884	1.829732
H	-5.007242	-5.513063	0.697880
H	-7.397290	-3.921762	0.420400
H	-6.644967	-4.820422	-0.917830

C	-4.504290	-0.571068	-1.876961
Pd	-2.346798	-0.127149	1.146104
H	-5.518006	-0.776629	-2.209781
C	-3.871763	0.697618	-2.364837
H	-4.612574	1.369703	-2.804978
H	-3.377998	1.220798	-1.536457
H	-3.097478	0.504455	-3.120291
H	-4.805634	1.875375	1.257770
H	-6.456490	-0.436263	0.100886

TS-K1-γγ-RR

	Value
Charge	0
Electronic Energy, BS1 (a.u.)	-2957.946750
Thermal and entropic correction, BS1 (a.u.)	0.752201
Electronic Energy, BS2 (a.u.)	-2958.790918

Molecular Geometry in Cartesian Coordinates

P	-0.303597	-0.344444	0.551368
P	4.401154	0.886420	-0.921472
C	0.012194	0.983381	1.807167
C	0.734820	-1.733786	1.156973
C	0.328685	0.379435	-1.036249
C	4.500001	0.918420	0.929718
C	3.774948	-0.850260	-1.196995
C	6.173219	0.738906	-1.426880
C	-0.195370	0.641028	3.156251
C	0.290349	2.319961	1.489010
C	0.179776	-3.022879	1.121797
C	2.012039	-1.551027	1.706948
C	1.465491	0.055390	-1.809884
C	-0.528282	1.401092	-1.498744
C	3.684594	1.844669	1.595029
C	5.275547	0.030368	1.695658
C	4.609231	-1.969079	-1.026766
C	2.414449	-1.064067	-1.516332
C	6.499427	0.152682	-2.663598
C	7.202009	1.339523	-0.682361
H	-0.411270	-0.389908	3.424099
C	-0.130758	1.609835	4.155593
C	0.340223	3.292882	2.491689
H	0.469948	2.613568	0.461356
C	0.900805	-4.115674	1.605084
H	-0.818209	-3.165037	0.715652
C	2.732332	-2.645191	2.182332
H	2.444821	-0.560168	1.763615
C	1.695764	0.773265	-2.999924
H	-1.415540	1.639878	-0.920330
C	-0.275234	2.110635	-2.666697
H	3.077763	2.535637	1.018504
C	3.618952	1.868637	2.991431
H	5.921138	-0.688603	1.202283
C	5.221901	0.061792	3.088225
H	5.659770	-1.821691	-0.799438
C	4.122256	-3.266512	-1.157577
C	1.939335	-2.377996	-1.654816
H	5.722936	-0.317496	-3.260834
C	7.814567	0.147377	-3.127880
H	6.979258	1.805854	0.272490
C	8.517765	1.336667	-1.150708
H	-0.291124	1.324408	5.191537
C	0.127170	2.944470	3.825988
H	0.552733	4.324387	2.224032
C	2.179830	-3.928184	2.131727
H	0.462419	-5.109130	1.571492
H	3.725779	-2.489086	2.590481
C	0.850607	1.790943	-3.427497
H	-0.962060	2.890152	-2.983042

H	2.956770	2.572112	3.485121
C	4.384539	0.975027	3.739062
H	5.826557	-0.630808	3.667822
C	2.777412	-3.472938	-1.470390
H	4.789100	-4.112337	-1.015170
H	8.045749	-0.321011	-4.080925
C	8.830556	0.738432	-2.372408
H	9.299234	1.800850	-0.554930
H	0.162530	3.703107	4.602587
H	2.744402	-4.777824	2.505863
H	1.062613	2.320080	-4.351892
H	4.331399	0.986855	4.824091
H	2.383225	-4.479443	-1.572062
H	9.855050	0.733604	-2.733650
H	0.895173	-2.530868	-1.910398
H	2.563132	0.508211	-3.596890
C	-4.598542	-0.739730	1.611923
H	-4.195126	-1.021557	2.587884
H	-5.389403	-1.383503	1.239599
C	-4.568576	0.601408	1.224674
C	-5.339519	1.167422	0.170499
C	-5.172271	2.574388	-0.193564
C	-3.934161	3.239912	-0.040948
H	-3.087136	2.703375	0.376849
C	-3.772825	4.565711	-0.436974
H	-2.807258	5.048972	-0.305987
C	-4.836022	5.269404	-1.011387
H	-4.707443	6.301399	-1.326133
C	-6.066205	4.624918	-1.180140
H	-6.902296	5.159196	-1.624912
C	-6.231096	3.300478	-0.778235
H	-7.192542	2.810290	-0.915695
H	-2.563700	-2.448412	-1.377610
H	-2.222335	-0.754800	-1.963593
C	-4.297587	-1.185971	-1.488819
C	-2.899226	-1.411618	-1.418794
B	-5.233112	-2.329982	-1.046055
O	-4.813297	-3.625607	-0.806906
O	-6.590185	-2.163162	-0.836423
C	-5.926203	-4.348376	-0.256457
C	-7.150204	-3.454987	-0.546442
H	-5.763507	-4.487127	0.820000
H	-5.997045	-5.333947	-0.727142
H	-7.833419	-3.377572	0.304847
H	-7.717810	-3.802258	-1.419085
C	-4.857116	0.061574	-1.845452
Pd	-2.567652	-0.806634	0.651434
H	-5.925851	0.058651	-2.039959
C	-4.074972	1.105028	-2.595396
H	-4.670594	2.001932	-2.776040
H	-3.175776	1.408734	-2.056317
H	-3.751428	0.711953	-3.569829
H	-3.896513	1.265085	1.771129
H	-6.331802	0.749172	0.032277

3-(*R,R*)-Pd

	Value
Charge	0
Electronic Energy, BS1 (a.u.)	-2957.998801
Thermal and entropic correction, BS1 (a.u.)	0.752580
Electronic Energy, BS2 (a.u.)	-2958.838076

Molecular Geometry in Cartesian Coordinates

P	-0.202885	0.246543	0.075402
P	4.783182	0.745939	-0.851276
C	0.195758	1.818737	0.975733
C	0.605531	-1.021151	1.140843
C	0.687396	0.467239	-1.544196

C	4.728475	1.157794	0.957017
C	4.019354	-0.955485	-0.847950
C	6.569965	0.355219	-1.125458
C	-0.207015	1.900577	2.321561
C	0.741590	2.958930	0.370886
C	-0.055884	-2.249102	1.301229
C	1.805177	-0.794385	1.831934
C	1.910645	-0.069128	-2.005190
C	-0.024578	1.339745	-2.396032
C	3.981546	2.284556	1.328102
C	5.328879	0.386176	1.967280
C	4.707510	-2.065233	-0.325078
C	2.695823	-1.136776	-1.309114
C	6.944165	-0.517104	-2.163684
C	7.583817	1.026565	-0.421946
H	-0.642448	1.030603	2.805766
C	-0.060106	3.084475	3.041016
C	0.873344	4.151906	1.089311
H	1.070918	2.926212	-0.661584
C	0.486466	-3.240966	2.120290
H	-0.990243	-2.431617	0.778665
C	2.345963	-1.789348	2.645287
H	2.321787	0.152880	1.734585
C	2.381152	0.317627	-3.275527
H	-0.985314	1.725739	-2.063309
C	0.463568	1.721105	-3.641031
H	3.511965	2.892126	0.561949
C	3.807838	2.619139	2.674322
H	5.925015	-0.481376	1.705662
C	5.168138	0.725475	3.309865
H	5.733589	-1.948589	0.006784
C	4.109055	-3.318341	-0.236764
C	2.107221	-2.409383	-1.219915
H	6.180641	-1.051077	-2.722620
C	8.287841	-0.725871	-2.473661
H	7.325042	1.709365	0.381575
C	8.928685	0.818533	-0.735421
H	-0.372839	3.123861	4.080834
C	0.474926	4.220513	2.424263
H	1.297350	5.025025	0.600567
C	1.689442	-3.014512	2.791153
H	-0.031953	-4.189810	2.230071
H	3.283540	-1.601971	3.159189
C	1.684374	1.208392	-4.083330
H	-0.110372	2.401877	-4.263117
H	3.199594	3.478151	2.937996
C	4.397300	1.837558	3.666874
H	5.638326	0.119290	4.079685
C	2.796479	-3.489752	-0.680520
H	4.663284	-4.155924	0.177458
H	8.554102	-1.412120	-3.273096
C	9.286963	-0.059511	-1.759625
H	9.697036	1.342494	-0.173141
H	0.576987	5.148406	2.979975
H	2.114565	-3.786781	3.426590
H	2.080748	1.483663	-5.056307
H	4.260615	2.091027	4.714445
H	2.313300	-4.459702	-0.612177
H	10.333644	-0.223138	-2.000046
H	1.092462	-2.539830	-1.582859
H	3.313229	-0.112248	-3.629722
C	-3.637677	1.302723	1.254814
H	-2.970779	2.140676	1.434653
H	-3.919456	0.713728	2.126295
C	-4.317974	1.169440	0.063809
C	-5.525976	0.271392	-0.168260
C	-6.853328	1.012550	-0.276738
C	-6.971434	2.316461	-0.777767
H	-6.084497	2.863729	-1.080832
C	-8.220869	2.931740	-0.891831
H	-8.286931	3.944885	-1.279739
C	-9.378741	2.252483	-0.510479
H	-10.349858	2.731641	-0.598703
C	-9.276687	0.951652	-0.010580

H	-10.170107	0.412748	0.293910
C	-8.027037	0.343262	0.105089
H	-7.952342	-0.668294	0.497955
H	-2.164122	-2.388244	-1.806847
H	-2.796502	-1.024358	-2.853359
C	-3.997267	-1.496252	-1.091035
C	-2.861614	-1.561859	-1.910241
B	-4.044903	-2.505995	0.090927
O	-3.053346	-3.445306	0.331506
O	-5.071396	-2.582698	1.014565
C	-3.326753	-4.044712	1.608887
C	-4.800795	-3.695129	1.886138
H	-2.653326	-3.606527	2.356342
H	-3.143260	-5.122004	1.557920
H	-4.982918	-3.401680	2.923951
H	-5.477501	-4.519658	1.629622
C	-5.258326	-0.664584	-1.398071
Pd	-2.506761	-0.128102	-0.210030
H	-6.111077	-1.356404	-1.461018
C	-5.192544	0.097836	-2.724815
H	-6.114857	0.660432	-2.894637
H	-4.356155	0.804830	-2.748529
H	-5.063679	-0.594239	-3.563093
H	-4.119613	1.911155	-0.710010
H	-5.607275	-0.386809	0.699919

TS-κ^2-γα-11′

	Value
Charge	0
Electronic Energy, BS1 (a.u.)	-2957.945742
Thermal and entropic correction, BS1 (a.u.)	0.756699
Electronic Energy, BS2 (a.u.)	-2958.786821

Molecular Geometry in Cartesian Coordinates

P	0.654240	1.344890	0.697556
P	-1.502788	-1.456189	0.044610
C	2.070698	2.526870	0.493251
C	-0.421985	2.278686	1.880824
C	1.350486	-0.036545	1.710720
C	-0.564283	-3.010718	0.400268
C	-1.959850	-0.979039	1.798080
C	-3.077481	-2.111316	-0.669981
C	2.114086	3.232693	-0.719507
C	3.061461	2.784609	1.456143
C	-1.763663	2.485920	1.536085
C	0.052939	2.783606	3.101646
C	0.523302	-0.871060	2.498790
C	2.695690	-0.402307	1.535080
C	0.708552	-3.200843	-0.147349
C	-1.092379	-3.986539	1.263983
C	-3.295828	-0.890956	2.220695
C	-0.943689	-0.661171	2.737795
C	-4.185691	-1.255035	-0.812304
C	-3.160323	-3.396823	-1.233758
H	1.364286	3.026864	-1.476779
C	3.111199	4.180854	-0.961434
C	4.060098	3.728167	1.212761
H	3.070126	2.232628	2.390251
C	-2.615632	3.186035	2.394324
H	-2.138674	2.103002	0.593876
C	-0.797563	3.480332	3.958328
H	1.085254	2.623534	3.394367
C	1.094479	-1.983100	3.138932
H	3.334206	0.196123	0.898724
C	3.234753	-1.533937	2.141250
H	1.135439	-2.438001	-0.790753
C	1.446291	-4.346342	0.168084
H	-2.076040	-3.842742	1.701867

C	-0.361017	-5.132176	1.568223
H	-4.094644	-1.179412	1.550696
C	-3.639761	-0.460458	3.501925
C	-1.308758	-0.242630	4.025722
H	-4.134847	-0.239255	-0.436371
C	-5.349375	-1.683553	-1.451254
H	-2.319298	-4.077450	-1.160939
C	-4.317080	-3.816025	-1.893720
H	3.129903	4.712429	-1.909200
C	4.086599	4.431357	0.004823
H	4.820239	3.913097	1.966998
C	-2.135471	3.683913	3.605724
H	-3.653835	3.338197	2.112408
H	-0.418336	3.861721	4.902734
C	2.433513	-2.322380	2.965194
H	4.270661	-1.798050	1.951508
H	2.445817	-4.464634	-0.236071
C	0.914214	-5.311774	1.021716
H	-0.780220	-5.879917	2.236210
C	-2.641308	-0.118907	4.409864
H	-4.687393	-0.402899	3.784479
H	-6.193224	-1.004233	-1.537110
C	-5.420252	-2.966451	-1.997502
H	-4.355411	-4.814046	-2.321913
H	4.867704	5.162761	-0.182718
H	-2.798043	4.226246	4.274953
H	2.835987	-3.208355	3.447041
H	1.491675	-6.198421	1.269324
H	-2.891173	0.226646	5.408702
H	-6.322311	-3.297457	-2.504325
H	-0.518325	-0.001568	4.729971
H	0.452703	-2.611906	3.748027
C	0.973642	0.584960	-3.180407
H	1.053829	1.659318	-3.328921
H	0.989809	0.053413	-4.130018
C	2.047075	0.060931	-2.318695
C	2.619820	-1.163991	-2.468594
C	3.823864	-1.639349	-1.791552
C	4.731409	-0.775182	-1.139715
H	4.556546	0.296482	-1.151957
C	5.861837	-1.270875	-0.496030
H	6.541508	-0.580495	-0.002970
C	6.135139	-2.643529	-0.492764
H	7.019548	-3.026885	0.007925
C	5.267732	-3.510878	-1.161779
H	5.474226	-4.577810	-1.183674
C	4.136333	-3.014412	-1.807462
H	3.469858	-3.698036	-2.327729
H	-4.071300	1.781377	-2.188027
H	-3.799991	0.167759	-3.041254
C	-2.058098	1.382214	-2.738828
C	-3.376532	1.086480	-2.652661
B	-1.611053	2.806177	-2.287773
O	-2.334350	3.563825	-1.391155
O	-0.532157	3.491820	-2.819497
C	-1.591926	4.772908	-1.152084
C	-0.599050	4.845157	-2.325672
H	-1.082799	4.686379	-0.185238
H	-2.277145	5.624786	-1.116282
H	0.397124	5.175310	-2.018671
H	-0.955243	5.496229	-3.133156
C	-1.081910	0.499976	-3.452978
Pd	-0.324059	0.287466	-1.160631
H	-0.735177	1.076240	-4.309428
C	-1.549291	-0.869311	-3.916005
H	-2.042737	-1.432806	-3.122726
H	-2.258416	-0.769510	-4.751619
H	-0.705493	-1.466512	-4.276071
H	2.486779	0.758818	-1.613208
H	2.157023	-1.867270	-3.161504

IV$^{Cu,\alpha}$Real

	Value
Charge	0
Electronic Energy, BS1 (a.u.)	-3106.71086
Thermal and entropic correction, BS1 (a.u.)	0.818331
Electronic Energy, BS2 (a.u.)	-4550.804249

Molecular Geometry in Cartesian Coordinates

C	-0.641288	2.048010	-1.419256
H	-1.085459	1.736525	-2.373558
B	-3.135301	2.370135	-0.760577
O	-4.197507	3.074340	-0.231007
O	-3.550135	1.262410	-1.478562
C	-5.388765	2.259069	-0.396285
C	-4.994858	1.332275	-1.605025
Cu	0.033076	0.386465	-0.513490
P	-1.040905	-1.227324	0.721452
P	2.115689	-0.584611	-0.717759
C	-2.269902	-0.612640	1.943904
C	-1.970353	-2.423431	-0.314481
C	0.092069	-2.272076	1.747036
C	3.255915	-0.635629	0.716362
C	1.826863	-2.339377	-1.220790
C	3.152136	0.115723	-2.071937
C	-2.090680	0.692667	2.426329
C	-3.331227	-1.387323	2.437759
C	-2.448673	-1.962596	-1.550973
C	-2.208395	-3.752956	0.066076
C	1.092233	-3.081135	1.149866
C	0.037894	-2.191066	3.148884
C	3.171847	0.421706	1.634627
C	4.211203	-1.644085	0.915432
C	2.004447	-2.699622	-2.567684
C	1.269137	-3.290992	-0.327630
C	2.496335	0.882477	-3.048214
C	4.541640	-0.059674	-2.160258
H	-1.300404	1.311648	2.014400
C	-2.940144	1.205867	3.408059
C	-4.184210	-0.868608	3.412635
H	-3.492087	-2.392769	2.061404
C	-3.152719	-2.824979	-2.393750
H	-2.285918	-0.926802	-1.836267
C	-2.915687	-4.609305	-0.777925
H	-1.827006	-4.121137	1.013965
C	1.957496	-3.806737	1.983242
H	-0.718066	-1.573124	3.619818
C	0.931291	-2.893080	3.956706
H	2.437617	1.207733	1.485268
C	4.038191	0.476959	2.727435
H	4.271656	-2.475824	0.219533
C	5.072072	-1.589789	2.011371
H	2.431553	-1.982114	-3.259701
C	1.646150	-3.961434	-3.039569
C	0.939508	-4.563120	-0.820278
H	1.424323	1.038595	-2.966044
C	3.215390	1.457400	-4.097998
H	5.067044	-0.636236	-1.405860
C	5.259315	0.521801	-3.206454
H	-2.795167	2.220827	3.766171
C	-3.983970	0.424226	3.906101
H	-5.005504	-1.473282	3.787091
C	-3.385589	-4.147381	-2.010453
H	-3.516362	-2.462251	-3.351024
H	-3.093747	-5.638175	-0.477271
C	1.892170	-3.714345	3.371891
H	0.865025	-2.802929	5.037189
H	3.971258	1.308184	3.422877
C	4.987949	-0.528451	2.917262
H	5.806259	-2.376818	2.160459
C	1.110364	-4.900834	-2.161261
H	1.792849	-4.205892	-4.087778

H	2.696810	2.052647	-4.844186
C	4.598104	1.280430	-4.176681
H	6.335808	0.385373	-3.262077
H	-4.650842	0.825228	4.664157
H	-3.929394	-4.817813	-2.670218
H	2.590962	-4.274105	3.986596
H	5.660237	-0.487942	3.769827
H	0.826299	-5.888565	-2.512277
H	5.160539	1.736605	-4.986464
H	0.230961	2.674481	-1.637167
C	-1.638614	2.721015	-0.552223
C	-1.314568	3.492213	0.524326
H	-2.110345	3.924198	1.128167
C	0.102726	3.644580	1.000720
H	0.600696	2.667905	0.874506
H	0.137258	3.880229	2.072485
H	0.524092	-5.290871	-0.130110
H	2.709689	-4.438320	1.520225
C	-5.576643	1.482852	0.910482
H	-6.497086	0.890981	0.903283
H	-4.732429	0.815799	1.094168
H	-5.628640	2.192695	1.740148
C	-5.565889	-0.080546	-1.540432
H	-6.660796	-0.053655	-1.530605
H	-5.249581	-0.651394	-2.417891
H	-5.218383	-0.614707	-0.654817
C	-5.285222	1.963694	-2.971024
H	-4.800367	1.365273	-3.748020
H	-6.358280	2.000633	-3.182397
H	-4.881465	2.978726	-3.027311
C	-6.578676	3.178655	-0.647630
H	-6.394863	3.856787	-1.483214
H	-7.481678	2.596381	-0.859711
H	-6.768947	3.786537	0.242266
C	0.975666	4.688713	0.244178
H	0.744338	4.632564	-0.823559
H	0.706291	5.697592	0.577564
C	2.445864	4.421133	0.464951
C	3.129672	4.952891	1.567181
C	3.135174	3.539007	-0.382565
C	4.462512	4.615450	1.816250
C	4.466170	3.198533	-0.138084
C	5.135063	3.735303	0.964846
H	2.611120	5.636792	2.235736
H	2.619747	3.105888	-1.235161
H	4.975752	5.041304	2.674809
H	4.972449	2.506739	-0.804909
H	6.169912	3.468111	1.160427

TS-IV-IV'Cu_Real

	Value
Charge	0
Electronic Energy, BS1 (a.u.)	-3106.699508
Thermal and entropic correction, BS1 (a.u.)	0.822090
Electronic Energy, BS2 (a.u.)	-4550.793853

Molecular Geometry in Cartesian Coordinates

C	-0.083199	1.975995	-2.283093
H	-0.829667	1.858360	-3.060423
B	-1.969024	2.722554	-0.813061
O	-2.503648	3.282782	0.323062
O	-2.898225	2.579152	-1.820356
C	-3.820255	3.797987	-0.017147
C	-4.199997	2.956076	-1.297589
Cu	0.111462	0.464002	-0.167053
P	-1.646333	-0.857393	0.474362
P	1.734833	-1.093803	-0.493507
C	-2.744603	-0.071431	1.729903

C	-2.767690	-1.534058	-0.817276
C	-1.072322	-2.380766	1.356574
C	2.376874	-1.812792	1.071027
C	0.977128	-2.529639	-1.396578
C	3.265415	-0.686090	-1.448480
C	-2.175282	0.943574	2.513532
C	-4.062629	-0.477798	1.989815
C	-2.794883	-0.885385	-2.060020
C	-3.556212	-2.679685	-0.620414
C	-0.330734	-3.376407	0.675853
C	-1.324764	-2.543575	2.728362
C	2.172442	-1.097722	2.258313
C	3.071952	-3.031895	1.114342
C	1.250912	-2.705547	-2.763336
C	-0.001023	-3.359634	-0.789320
C	3.119153	0.084262	-2.616079
C	4.559251	-1.047394	-1.042147
H	-1.168866	1.283029	2.284936
C	-2.895297	1.515713	3.562888
C	-4.787387	0.107974	3.028925
H	-4.526465	-1.247446	1.381917
C	-3.608514	-1.369672	-3.087084
H	-2.175346	-0.007354	-2.218859
C	-4.369370	-3.159390	-1.647131
H	-3.517587	-3.208797	0.327785
C	0.087069	-4.510623	1.389403
H	-1.885722	-1.786744	3.263633
C	-0.874708	-3.664149	3.424614
H	1.607327	-0.169950	2.225731
C	2.673364	-1.580880	3.468820
H	3.211645	-3.606760	0.203028
C	3.574575	-3.511564	2.322763
H	2.005504	-2.093522	-3.243191
C	0.576044	-3.653067	-3.530376
C	-0.656687	-4.318805	-1.578217
H	2.126718	0.384292	-2.932961
C	4.231161	0.465192	-3.366484
H	4.711289	-1.615572	-0.132545
C	5.672500	-0.659909	-1.791816
H	-2.441619	2.300465	4.161335
C	-4.200646	1.096241	3.825165
H	-5.808545	-0.210408	3.219387
C	-4.396211	-2.504542	-2.882493
H	-3.621486	-0.860997	-4.047307
H	-4.973857	-4.048321	-1.486758
C	-0.170879	-4.659078	2.750070
H	-1.082020	-3.757086	4.486827
H	2.506085	-1.019166	4.383552
C	3.379209	-2.784207	3.501159
H	4.112981	-4.454939	2.347398
C	-0.390982	-4.462229	-2.937401
H	0.811753	-3.756451	-4.585803
H	4.094682	1.060774	-4.264476
C	5.513681	0.094141	-2.955290
H	6.666252	-0.942055	-1.455760
H	-4.765522	1.547097	4.636300
H	-5.024111	-2.883969	-3.684252
H	0.183588	-5.540517	3.276372
H	3.769243	-3.160875	4.442569
H	-0.930509	-5.201656	-3.521873
H	6.382228	0.398422	-3.532302
H	0.954447	1.923124	-2.590438
C	-0.451569	2.387326	-1.006996
C	0.451977	2.515200	0.120947
H	-0.000091	3.000877	0.986365
C	1.902329	2.910838	-0.094597
H	2.326916	2.370288	-0.947496
H	1.980017	3.983288	-0.350580
H	-1.402583	-4.949892	-1.105265
H	0.646529	-5.275883	0.859840
C	-4.750341	3.602911	1.174446
H	-5.763264	3.940534	0.930016
H	-4.792488	2.560400	1.488332
H	-4.391555	4.193564	2.022615

C	-4.951507	1.662271	-0.974267
H	-5.011985	1.046835	-1.875231
H	-4.430983	1.083982	-0.209715
H	-5.968027	1.866944	-0.624560
C	-3.633114	5.292129	-0.305237
H	-2.949981	5.449698	-1.144937
H	-4.583823	5.785102	-0.529306
H	-3.195540	5.769902	0.576096
C	-4.939547	3.733140	-2.383128
H	-5.135161	3.074049	-3.234514
H	-5.901218	4.102738	-2.012284
H	-4.353177	4.580415	-2.743629
C	2.799333	2.644578	1.128299
H	2.362613	3.127392	2.011906
C	4.223925	3.115010	0.936132
C	4.747409	4.200152	1.651538
C	5.052655	2.477077	-0.000163
C	6.059475	4.635116	1.443343
C	6.362145	2.906285	-0.211698
C	6.872918	3.989096	0.510622
H	4.120161	4.709758	2.379685
H	4.664743	1.636944	-0.566439
H	6.445156	5.478909	2.010331
H	6.983307	2.391949	-0.940563
H	7.893625	4.325037	0.347966
H	2.789334	1.565723	1.324907

IV’^{Cu,γ}Real

	Value
Charge	0
Electronic Energy, BS1 (a.u.)	-3106.705016
Thermal and entropic correction, BS1 (a.u.)	0.819973
Electronic Energy, BS2 (a.u.)	-4550.798907

Molecular Geometry in Cartesian Coordinates

C	-0.287547	2.953574	-2.292129
H	-1.015673	3.053450	-3.092333
B	-2.226009	2.749352	-0.760934
O	-2.795769	3.016509	0.466178
O	-3.170284	2.621015	-1.759262
C	-4.181872	3.384301	0.230071
C	-4.486493	2.683335	-1.146613
Cu	0.186457	0.479993	0.175374
P	-1.509427	-1.019239	0.578087
P	1.823072	-1.021593	-0.482424
C	-2.744949	-0.473626	1.829545
C	-2.453822	-1.513946	-0.914661
C	-0.879495	-2.609438	1.284171
C	2.702434	-1.882458	0.876399
C	1.103652	-2.383512	-1.509671
C	3.146381	-0.304315	-1.541127
C	-2.322442	0.519086	2.726831
C	-4.023780	-1.031838	1.980061
C	-2.331086	-0.703386	-2.052998
C	-3.254889	-2.665675	-0.972861
C	-0.069186	-3.486393	0.519209
C	-1.092842	-2.899396	2.642689
C	2.610665	-1.333246	2.163085
C	3.439085	-3.061965	0.683503
C	1.329948	-2.404932	-2.895893
C	0.217270	-3.337777	-0.948196
C	2.747995	0.699715	-2.440921
C	4.501855	-0.655771	-1.468794
H	-1.348817	0.980516	2.584664
C	-3.145154	0.916903	3.781776
C	-4.852378	-0.619728	3.024620
H	-4.376961	-1.784138	1.283007
C	-3.011209	-1.033119	-3.226937

H	-1.711090	0.188345	-2.021524
C	-3.939869	-2.987050	-2.143808
H	-3.321637	-3.323607	-0.111099
C	0.462179	-4.626959	1.140829
H	-1.708993	-2.237748	3.240798
C	-0.531962	-4.023927	3.245994
H	2.021192	-0.433214	2.317813
C	3.255849	-1.945285	3.239352
H	3.493331	-3.509745	-0.304739
C	4.085609	-3.669919	1.758770
H	2.009468	-1.686098	-3.339371
C	0.700514	-3.333031	-3.723704
C	-0.384721	-4.278972	-1.797259
H	1.703673	0.997740	-2.483194
C	3.683702	1.328397	-3.261567
H	4.835142	-1.413498	-0.768317
C	5.438890	-0.014782	-2.283007
H	-2.804656	1.684161	4.471019
C	-4.409276	0.344863	3.934677
H	-5.842337	-1.054572	3.130603
C	-3.818508	-2.170750	-3.272722
H	-2.910001	-0.395774	-4.100458
H	-4.558091	-3.879823	-2.180265
C	0.247478	-4.897065	2.490591
H	-0.712676	-4.215795	4.299843
H	3.174925	-1.514279	4.233055
C	3.995777	-3.111450	3.037379
H	4.653484	-4.582704	1.601508
C	-0.162656	-4.277386	-3.172554
H	0.892999	-3.317916	-4.792667
H	3.361242	2.106399	-3.947341
C	5.033184	0.974992	-3.180674
H	6.488034	-0.287366	-2.210034
H	-5.054736	0.659495	4.749889
H	-4.347096	-2.426955	-4.186744
H	0.690506	-5.778486	2.944761
H	4.496180	-3.589667	3.874695
H	-0.661412	-5.007001	-3.803666
H	5.765903	1.475890	-3.806827
H	0.755199	3.085775	-2.572684
C	-0.683544	2.714579	-1.008755
C	0.206162	2.508447	0.154926
H	-0.291428	2.856136	1.069760
C	1.609592	3.103357	0.057641
H	2.134329	2.709826	-0.819343
H	1.571895	4.198291	-0.086999
H	-1.056131	-5.011723	-1.360167
H	1.074179	-5.299141	0.547146
C	2.476628	2.799443	1.292420
H	1.992310	3.208795	2.188536
H	2.499611	1.708687	1.420412
C	3.888566	3.325821	1.177805
C	4.324798	4.435822	1.912858
C	4.793479	2.719556	0.291570
C	5.625618	4.927124	1.772068
C	6.092215	3.205971	0.147174
C	6.515275	4.313348	0.888397
H	3.636745	4.920460	2.602095
H	4.472492	1.861229	-0.290784
H	5.942949	5.789259	2.353330
H	6.774158	2.718198	-0.544699
H	7.527571	4.693064	0.778305
C	-4.987893	1.245048	-0.988377
H	-4.340508	0.672302	-0.322484
H	-6.006739	1.219981	-0.589668
H	-4.983905	0.751975	-1.963401
C	-5.411853	3.461941	-2.076913
H	-5.550492	2.903398	-3.007774
H	-6.395665	3.604637	-1.617524
H	-4.998499	4.439671	-2.331385
C	-4.214811	4.915231	0.148887
H	-5.234238	5.293330	0.025713
H	-3.802707	5.327477	1.074440
H	-3.603214	5.279325	-0.681793

C	-5.029050	2.898078	1.398995
H	-6.088821	3.110001	1.220572
H	-4.907018	1.828905	1.567262
H	-4.728487	3.417265	2.313705

I-OMeCO2^{PdCu}Real

	Value
Charge	0
Electronic Energy, BS1 (a.u.)	-5769.817983
Thermal and entropic correction, BS1 (a.u.)	1.496691
Electronic Energy, BS2 (a.u.)	-7214.639372

Molecular Geometry in Cartesian Coordinates

Cu	1.550819	-0.007308	0.409510
P	2.926054	0.949640	2.124856
P	2.608524	-2.140024	0.526222
C	3.545517	2.612684	1.607531
C	2.274579	1.282808	3.822484
C	4.542570	0.110592	2.525555
C	4.376007	-2.010438	-0.006671
C	2.593805	-2.685670	2.295939
C	2.115949	-3.674726	-0.387472
C	3.863501	2.778847	0.251028
C	3.807284	3.663565	2.500653
C	0.904033	1.545663	3.965601
C	3.094686	1.310360	4.963149
C	4.563750	-1.203324	3.049617
C	5.767292	0.772937	2.338186
C	4.733737	-0.894488	-0.776360
C	5.339458	-2.998811	0.245716
C	1.586251	-3.587010	2.690219
C	3.357763	-2.053914	3.309880
C	1.312225	-3.532791	-1.525889
C	2.533207	-4.964400	-0.014027
H	3.618059	1.995048	-0.456895
C	4.487094	3.944935	-0.195103
C	4.400515	4.844099	2.047066
H	3.559175	3.559757	3.551672
C	0.367147	1.837394	5.220153
H	0.255524	1.487394	3.099596
C	2.553860	1.595860	6.217677
H	4.157426	1.110000	4.872167
C	5.795544	-1.776208	3.405220
H	5.779499	1.776927	1.933438
C	6.983594	0.176042	2.667336
H	3.993593	-0.127349	-0.982241
C	6.026465	-0.770121	-1.290701
H	5.086044	-3.863016	0.851953
C	6.629478	-2.876418	-0.269178
H	0.981480	-4.075836	1.934711
C	1.317487	-3.852852	4.030539
C	3.068300	-2.330479	4.656534
H	0.985161	-2.544285	-1.826041
C	0.913673	-4.650532	-2.261940
H	3.145807	-5.102288	0.870990
C	2.135306	-6.080754	-0.747528
H	4.719017	4.046149	-1.249396
C	4.759972	4.979632	0.703634
H	4.594188	5.651793	2.748071
C	1.188510	1.861844	6.348395
H	-0.698019	2.022469	5.311494
H	3.200078	1.611406	7.091499
C	7.000032	-1.103946	3.215900
H	7.910134	0.718170	2.500461
H	6.282252	0.101407	-1.887056
C	6.975804	-1.762658	-1.041932
H	7.367173	-3.648157	-0.064319
C	2.052472	-3.209429	5.025124

H	0.525785	-4.549937	4.290763
H	0.284609	-4.519823	-3.135611
C	1.318659	-5.925713	-1.871321
H	2.459163	-7.071370	-0.439347
H	5.236814	5.892567	0.356691
H	0.767041	2.080362	7.326009
H	7.938172	-1.580755	3.485530
H	7.981075	-1.670867	-1.443734
H	1.844830	-3.392020	6.075508
H	1.006541	-6.795318	-2.442667
H	3.658246	-1.834875	5.421699
H	5.796435	-2.781275	3.817414
Pd	-1.940395	-0.375741	0.054572
P	-2.899705	1.613816	1.002013
P	-3.215170	-0.312494	-1.846308
C	-3.254673	1.167910	2.759096
C	-1.969229	3.186133	1.133209
C	-4.574123	2.124862	0.398933
C	-5.027072	-0.584883	-1.714312
C	-3.058394	1.380928	-2.582473
C	-2.696471	-1.493861	-3.171878
C	-3.737873	-0.123474	3.030859
C	-3.107937	2.072395	3.819685
C	-0.568758	3.115372	1.159446
C	-2.606784	4.424900	1.307153
C	-4.792808	2.439306	-0.962078
C	-5.658926	2.188490	1.290047
C	-5.562442	-1.157581	-0.554346
C	-5.885610	-0.250731	-2.774956
C	-2.180816	1.591426	-3.657343
C	-3.732956	2.496810	-2.022832
C	-1.331060	-1.572510	-3.503637
C	-3.590337	-2.375835	-3.797159
H	-3.820458	-0.851431	2.229716
C	-4.087755	-0.495151	4.328160
C	-3.447452	1.694082	5.121970
H	-2.731563	3.073044	3.635797
C	0.182607	4.264635	1.404510
H	-0.074781	2.162072	1.002367
C	-1.849206	5.573245	1.541436
H	-3.690391	4.488919	1.275994
C	-6.085619	2.806825	-1.370735
H	-5.506106	1.960436	2.337712
C	-6.937083	2.542712	0.862076
H	-4.909928	-1.413146	0.272401
C	-6.934482	-1.399720	-0.455210
H	-5.481834	0.205406	-3.674421
C	-7.253640	-0.496053	-2.675858
H	-1.653346	0.756477	-4.098686
C	-1.972018	2.860584	-4.192628
C	-3.508094	3.765315	-2.581132
H	-0.615678	-0.932585	-2.997212
C	-0.880811	-2.473373	-4.466554
H	-4.644740	-2.359138	-3.548021
C	-3.132846	-3.293303	-4.747523
H	-4.461811	-1.498135	4.511218
C	-3.941632	0.414064	5.379021
H	-3.326435	2.405515	5.934455
C	-0.455506	5.490106	1.606797
H	1.264717	4.199220	1.427352
H	-2.347147	6.528723	1.682723
C	-7.153473	2.852433	-0.478586
H	-7.754265	2.577133	1.576878
H	-7.331446	-1.848646	0.450006
C	-7.780164	-1.071381	-1.514968
H	-7.909700	-0.233176	-3.500857
C	-2.644159	3.954043	-3.655742
H	-1.286420	2.983470	-5.025690
H	0.177376	-2.517945	-4.706441
C	-1.782194	-3.339685	-5.091505
H	-3.840076	-3.971076	-5.217380
H	-4.206524	0.125070	6.392185
H	0.133818	6.381945	1.802256
H	-8.143641	3.125671	-0.831567

H	-8.847558	-1.258354	-1.437775
H	-2.501266	4.948280	-4.064484
H	-1.428591	-4.052723	-5.830870
H	-4.034370	4.614949	-2.156607
H	-6.248060	3.042824	-2.417954
H	-1.229125	-0.720099	3.329870
C	-0.433779	-1.023555	2.631752
H	0.520914	-0.945802	3.165474
H	-0.577479	-2.089447	2.415366
O	-0.402406	-0.227692	1.474678
C	-1.458087	-2.419573	-0.193099
H	-0.584570	-2.548433	0.442418
H	-1.222741	-2.636255	-1.230278
C	-2.680201	-3.074204	0.265170
C	-2.969256	-3.455680	1.537232
C	-4.249614	-3.953253	2.035014
C	-4.398403	-4.188529	3.419537
H	-3.545370	-4.023445	4.073663
C	-5.609641	-4.617136	3.960362
H	-5.690039	-4.786076	5.031187
C	-6.714855	-4.830140	3.132876
H	-7.660376	-5.163353	3.550842
C	-6.585472	-4.612929	1.756561
H	-7.434382	-4.780591	1.098440
C	-5.376532	-4.184421	1.214755
H	-5.308640	-4.020392	0.144372
C	0.946263	0.881588	-1.376638
H	0.478468	-0.009603	-1.814495
H	0.148825	1.577379	-1.099622
C	1.889134	1.514317	-2.323041
C	2.653395	0.831013	-3.224198
H	3.318789	1.384382	-3.885491
C	2.592939	-0.655887	-3.415288
H	2.575782	-1.156782	-2.439572
H	1.642127	-0.943528	-3.894952
B	1.954521	3.058521	-2.507036
O	3.046582	3.713416	-3.064209
C	2.584709	5.000409	-3.541188
C	1.325720	5.266890	-2.632195
O	0.900591	3.921512	-2.287376
H	-2.199138	-3.324320	2.294323
H	-3.448785	-3.199716	-0.497993
C	3.712675	6.016913	-3.396029
H	4.070110	6.079090	-2.366440
H	4.555454	5.724566	-4.030960
H	3.380759	7.012575	-3.709406
C	2.232340	4.813431	-5.023296
H	3.115069	4.442877	-5.553261
H	1.435856	4.073021	-5.144065
H	1.914985	5.750360	-5.491331
C	0.173048	5.980816	-3.332761
H	-0.161337	5.439382	-4.217901
H	-0.673835	6.061360	-2.645223
H	0.470630	6.991713	-3.632225
C	1.656035	5.995475	-1.327729
H	0.764128	6.009551	-0.699193
H	2.447462	5.483617	-0.777825
H	1.964991	7.029070	-1.513505
C	3.754188	-1.214414	-4.257078
H	4.699310	-0.944686	-3.773072
H	3.749244	-0.724536	-5.239919
C	3.678367	-2.713065	-4.426878
C	4.311082	-3.571970	-3.516023
C	2.929460	-3.279676	-5.468850
C	4.210456	-4.956950	-3.652750
C	2.820777	-4.665875	-5.605123
C	3.465452	-5.509682	-4.697565
H	4.885452	-3.149424	-2.696298
H	2.436602	-2.624829	-6.185063
H	4.703599	-5.604465	-2.933166
H	2.240062	-5.085431	-6.423157
H	3.384713	-6.588506	-4.801351

V^{Pd}Real

	Value
Charge	0
Electronic Energy, BS1 (a.u.)	-3385.687601
Thermal and entropic correction, BS1 (a.u.)	0.964495
Electronic Energy, BS2 (a.u.)	-3386.667827

Molecular Geometry in Cartesian Coordinates

P	2.107287	-1.264828	-0.755496
P	-0.352468	-0.852822	1.526244
C	3.339379	-1.197492	-2.126888
C	3.271098	-1.456486	0.658645
C	1.152043	-2.814588	-1.038561
C	-1.914611	-1.754766	1.127931
C	0.616342	-2.291411	2.239484
C	-0.749718	0.134677	3.029171
C	3.814872	0.067910	-2.500560
C	3.903456	-2.338261	-2.718976
C	3.435945	-0.351018	1.505600
C	4.058016	-2.598509	0.858567
C	0.562097	-3.572145	-0.001123
C	0.766738	-3.073554	-2.368682
C	-2.504813	-1.634173	-0.136284
C	-2.503471	-2.607189	2.078606
C	1.004199	-2.308792	3.588188
C	0.913253	-3.427038	1.445565
C	0.301463	0.832995	3.648745
C	-2.050050	0.291915	3.530295
H	3.380709	0.952652	-2.047911
C	4.832173	0.194702	-3.447020
C	4.914573	-2.209915	-3.671890
H	3.551265	-3.327026	-2.442172
C	4.369979	-0.389906	2.542752
H	2.828670	0.536829	1.352825
C	4.982272	-2.639617	1.901272
H	3.939778	-3.459116	0.207056
C	-0.365139	-4.573808	-0.335514
H	1.197117	-2.488445	-3.174586
C	-0.162799	-4.063083	-2.678410
H	-2.043196	-1.007804	-0.892187
C	-3.680919	-2.326338	-0.435139
H	-2.044141	-2.727790	3.055254
C	-3.673544	-3.301241	1.777052
H	0.780421	-1.463290	4.225289
C	1.658913	-3.403550	4.152389
C	1.545798	-4.531074	2.037180
H	1.308431	0.758650	3.249331
C	0.068747	1.614780	4.779630
H	-2.886500	-0.205349	3.052924
C	-2.286927	1.108771	4.638327
H	5.186907	1.182742	-3.726722
C	5.381569	-0.944557	-4.037588
H	5.340620	-3.099947	-4.126543
C	5.141112	-1.535435	2.744164
H	4.487905	0.471967	3.193681
H	5.577923	-3.534439	2.058166
C	-0.736058	-4.818145	-1.654278
H	-0.437887	-4.237406	-3.714647
H	-4.129921	-2.210843	-1.415612
C	-4.267298	-3.156391	0.519436
H	-4.121442	-3.952753	2.522387
C	1.925276	-4.528140	3.376667
H	1.944629	-3.374099	5.199957
H	0.897097	2.133938	5.253036
C	-1.228732	1.759507	5.275241
H	-3.303309	1.229571	5.002321
H	6.168798	-0.848761	-4.779985
H	5.863458	-1.570808	3.554827
H	-1.470050	-5.586285	-1.878372

H	-5.182657	-3.693036	0.285231
H	2.425127	-5.392447	3.804168
H	-1.415020	2.384907	6.143683
H	1.757047	-5.397334	1.416740
H	-0.816505	-5.146304	0.468775
C	0.477055	0.966190	-2.533432
H	1.385543	0.560269	-2.976601
H	0.467203	2.051646	-2.622348
C	-0.724137	0.284095	-3.010162
C	-1.940107	0.842957	-3.248161
C	-3.162161	0.140918	-3.631514
C	-3.238183	-1.258892	-3.813302
H	-2.355028	-1.871793	-3.660771
C	-4.435603	-1.876038	-4.165820
H	-4.460374	-2.955208	-4.296548
C	-5.603380	-1.124959	-4.342784
H	-6.536211	-1.612050	-4.611757
C	-5.549637	0.260014	-4.169217
H	-6.445078	0.861610	-4.302735
C	-4.349417	0.880408	-3.825560
H	-4.321304	1.960071	-3.698029
H	0.232141	3.863272	2.587292
H	-1.353045	2.942757	2.417674
C	-0.077259	2.957569	0.687673
C	-0.425431	3.270370	1.958014
B	1.264943	3.549638	0.161401
O	2.301241	3.928492	0.991822
O	1.539858	3.798033	-1.166744
C	3.434881	4.280905	0.153818
C	2.744253	4.608603	-1.223607
C	-0.872712	2.106429	-0.233550
Pd	0.431389	0.382896	-0.460350
H	-0.855067	2.538636	-1.234061
C	-2.302971	1.763442	0.141765
H	-2.353085	1.314084	1.136613
H	-2.683485	1.013405	-0.557011
H	-0.623309	-0.792841	-3.146102
H	-2.040504	1.920699	-3.125786
C	4.174045	5.449072	0.798069
H	3.506371	6.289975	0.994074
H	4.603465	5.131334	1.753119
H	4.992149	5.792344	0.156203
C	4.340962	3.046570	0.098102
H	4.640984	2.777056	1.114605
H	3.812739	2.192574	-0.330444
H	5.245616	3.230720	-0.488979
C	2.284120	6.066433	-1.335395
H	1.643084	6.168495	-2.215642
H	1.700757	6.362015	-0.458592
H	3.129734	6.752535	-1.441730
C	3.543094	4.210017	-2.459552
H	3.747261	3.138530	-2.476715
H	2.974537	4.456309	-3.361219
H	4.494921	4.750054	-2.498069
C	-3.298862	2.950354	0.109676
H	-3.360180	3.333223	-0.917038
H	-2.927126	3.767884	0.735721
C	-4.656590	2.492631	0.587847
C	-5.076042	2.723090	1.905668
C	-5.484387	1.727835	-0.248872
C	-6.288157	2.210437	2.374497
C	-6.695039	1.213158	0.215127
C	-7.103069	1.452872	1.530364
H	-4.442241	3.305723	2.570440
H	-5.168699	1.522969	-1.268676
H	-6.596012	2.403797	3.399150
H	-7.317883	0.621808	-0.451262
H	-8.045844	1.052545	1.893453

TS-V-V’^{Pd}Real

	Value

Charge	0
Electronic Energy, BS1 (a.u.)	-3385.665972
Thermal and entropic correction, BS1 (a.u.)	0.960910
Electronic Energy, BS2 (a.u.)	-3386.64747254

Molecular Geometry in Cartesian Coordinates

P	-2.066337	0.580846	0.873921
P	0.895398	-0.920856	1.486480
C	-3.582088	1.438619	0.258989
C	-2.890125	-0.755640	1.838983
C	-1.257303	1.794016	2.002984
C	2.176214	0.331991	1.937451
C	0.045435	-1.065511	3.153624
C	1.795074	-2.521845	1.367751
C	-4.186219	0.916213	-0.895126
C	-4.217754	2.489160	0.937704
C	-2.728021	-2.074131	1.391735
C	-3.753458	-0.495256	2.912244
C	-0.552083	1.437628	3.173433
C	-1.140620	3.111513	1.516561
C	2.330879	1.477931	1.148442
C	2.946809	0.200818	3.104842
C	0.014366	-2.285354	3.846520
C	-0.546657	0.066360	3.768799
C	1.030978	-3.674757	1.115303
C	3.190467	-2.638793	1.436826
H	-3.714084	0.093005	-1.419199
C	-5.388901	1.439030	-1.369680
C	-5.417971	3.016449	0.457761
H	-3.779648	2.902304	1.839931
C	-3.412997	-3.119697	2.014496
H	-2.063369	-2.277630	0.556905
C	-4.429542	-1.540803	3.538335
H	-3.895416	0.524403	3.258237
C	0.185518	2.425999	3.848486
H	-1.632907	3.388233	0.590668
C	-0.389091	4.071263	2.188940
H	1.718223	1.594434	0.262210
C	3.245116	2.470377	1.505146
H	2.821519	-0.669943	3.741650
C	3.865142	1.188543	3.458367
H	0.471228	-3.165253	3.412750
C	-0.578024	-2.402949	5.103809
C	-1.114872	-0.064607	5.044557
H	-0.047102	-3.595405	1.011865
C	1.641890	-4.922530	0.998616
H	3.808068	-1.761949	1.593230
C	3.803170	-3.884627	1.280847
H	-5.839243	1.023616	-2.266716
C	-6.005090	2.496052	-0.697265
H	-5.895618	3.833439	0.991284
C	-4.261646	-2.854641	3.089811
H	-3.279321	-4.137865	1.659697
H	-5.089088	-1.331426	4.375837
C	0.273667	3.728552	3.368569
H	-0.316396	5.075015	1.781522
H	3.348112	3.353941	0.881657
C	4.016364	2.325560	2.658462
H	4.458904	1.073927	4.360963
C	-1.143143	-1.285882	5.712428
H	-0.583663	-3.367008	5.604232
H	1.033549	-5.803491	0.815683
C	3.032036	-5.030298	1.075827
H	4.886205	-3.955064	1.326367
H	-6.937980	2.910189	-1.068990
H	-4.792865	-3.666787	3.578115
H	0.868756	4.464189	3.901401
H	4.729141	3.096178	2.938374
H	-1.602519	-1.360876	6.693715
H	3.511436	-5.998656	0.964562

H	-1.556891	0.814558	5.504537
H	0.725212	2.142253	4.746484
C	-0.926830	1.198415	-2.061443
H	-2.013610	1.320431	-2.042261
H	-0.684100	0.809963	-3.052900
C	-0.269414	2.529724	-1.802976
C	-0.415889	3.643742	-2.542883
C	0.188251	4.957613	-2.264727
C	0.930857	5.235183	-1.097083
H	1.070601	4.462301	-0.348158
C	1.478025	6.496120	-0.876183
H	2.044896	6.681151	0.032948
C	1.300934	7.522588	-1.810713
H	1.728909	8.505565	-1.635303
C	0.565110	7.268106	-2.968931
H	0.416259	8.054859	-3.703992
C	0.016745	6.004131	-3.189870
H	-0.554778	5.817658	-4.096072
H	1.012744	-4.266387	-2.046580
H	2.411022	-3.249734	-1.418583
C	0.739994	-2.164892	-2.228998
C	1.430762	-3.276671	-1.885006
B	-0.642797	-2.388232	-2.911485
O	-1.409504	-3.520430	-2.708869
O	-1.230125	-1.509186	-3.794511
C	-2.708382	-3.283634	-3.314589
C	-2.384014	-2.168469	-4.379091
C	1.185817	-0.763057	-1.989301
Pd	-0.295595	-0.116967	-0.535765
H	0.961217	-0.155894	-2.868448
C	2.645288	-0.549612	-1.605099
H	2.910854	-1.138477	-0.722994
H	2.808779	0.496605	-1.328620
H	0.364417	2.604994	-0.921577
H	-1.040532	3.598021	-3.436587
C	-3.228855	-4.595448	-3.891811
H	-2.513170	-5.043895	-4.583235
H	-3.406294	-5.309617	-3.081833
H	-4.175806	-4.438963	-4.419068
C	-3.625994	-2.793987	-2.189459
H	-3.675198	-3.557732	-1.408308
H	-3.224937	-1.884036	-1.737801
H	-4.642636	-2.597684	-2.542544
C	-1.928019	-2.736926	-5.727245
H	-1.529611	-1.922007	-6.338340
H	-1.132777	-3.475683	-5.592092
H	-2.753757	-3.205478	-6.271194
C	-3.482872	-1.131102	-4.584253
H	-3.703556	-0.593606	-3.660572
H	-3.161285	-0.396057	-5.328124
H	-4.402027	-1.603294	-4.946759
C	3.660468	-0.890616	-2.728105
H	3.507914	-0.202288	-3.568148
H	3.463714	-1.900533	-3.101685
C	5.076037	-0.792338	-2.213552
C	5.646780	-1.861411	-1.504421
C	5.821385	0.387409	-2.345768
C	6.921491	-1.755902	-0.947116
C	7.098611	0.497694	-1.790984
C	7.653795	-0.573732	-1.088251
H	5.078092	-2.780566	-1.385043
H	5.393495	1.226958	-2.889163
H	7.346062	-2.597440	-0.405180
H	7.660168	1.420975	-1.908525
H	8.647537	-0.489789	-0.656846

V’^{Pd}Real

	Value
Electronic Energy, BS1 (a.u.)	-3385.687554
Thermal and entropic correction, BS1 (a.u.)	0.964637

	Value
Electronic Energy, BS2 (a.u.)	-3386.668290

Molecular Geometry in Cartesian Coordinates

P	1.983120	-1.435267	-0.455212
P	-0.458884	-0.444529	1.663311
C	3.207994	-1.668998	-1.813192
C	3.142565	-1.317448	0.971730
C	1.027394	-3.005906	-0.379004
C	-2.030842	-1.380890	1.413155
C	0.457946	-1.716690	2.695599
C	-0.868215	0.845614	2.911428
C	3.613299	-0.530288	-2.524288
C	3.827122	-2.899296	-2.084401
C	3.322515	-0.054599	1.554586
C	3.905604	-2.399726	1.430105
C	0.406511	-3.479766	0.799769
C	0.672621	-3.585993	-1.611571
C	-2.567369	-1.528409	0.128264
C	-2.663228	-2.015625	2.497130
C	0.821922	-1.444725	4.024002
C	0.742871	-3.005519	2.178887
C	0.184641	1.625798	3.421638
C	-2.179342	1.138065	3.314505
H	3.129983	0.420281	-2.325407
C	4.619598	-0.617342	-3.487476
C	4.827312	-2.985874	-3.052865
H	3.522618	-3.792010	-1.546854
C	4.248698	0.121703	2.584509
H	2.734650	0.788739	1.203468
C	4.823006	-2.223856	2.464939
H	3.772261	-3.382663	0.988560
C	-0.528579	-4.523615	0.699590
H	1.128353	-3.220111	-2.525392
C	-0.262011	-4.614678	-1.689772
H	-2.070078	-1.073365	-0.721196
C	-3.722354	-2.289429	-0.072746
H	-2.246688	-1.925627	3.496073
C	-3.817954	-2.769031	2.296212
H	0.601160	-0.478711	4.457768
C	1.447256	-2.396178	4.829296
C	1.348916	-3.958579	3.012813
H	1.201889	1.444374	3.088008
C	-0.060171	2.625049	4.363253
H	-3.013979	0.574808	2.913030
C	-2.426165	2.167490	4.226140
H	4.919705	0.272306	-4.033940
C	5.226729	-1.845308	-3.755126
H	5.296697	-3.944071	-3.257659
C	4.997363	-0.962993	3.043212
H	4.378292	1.104887	3.028389
H	5.400063	-3.071783	2.823032
C	-0.872805	-5.083923	-0.526974
H	-0.515960	-5.038983	-2.656699
H	-4.120323	-2.399942	-1.075239
C	-4.348349	-2.907107	1.009184
H	-4.300158	-3.252557	3.141476
C	1.708845	-3.666582	4.325142
H	1.713873	-2.140014	5.850699
H	0.768957	3.205210	4.757842
C	-1.368217	2.903224	4.764392
H	-3.449022	2.384267	4.521310
H	6.005316	-1.915972	-4.509414
H	5.713642	-0.829639	3.849083
H	-1.614997	-5.875136	-0.573263
H	-5.245548	-3.499526	0.851644
H	2.187512	-4.422308	4.941112
H	-1.562569	3.694242	5.483052
H	1.553057	-4.943683	2.603363
H	-1.005390	-4.876683	1.608560
C	0.403445	0.368051	-2.692806
H	1.077043	-0.398977	-3.076869

H	0.750722	1.364723	-2.967553
C	-0.995015	0.086771	-3.002866
C	-1.511818	-1.123625	-3.339350
C	-2.928167	-1.455119	-3.473338
C	-3.972374	-0.543700	-3.194317
H	-3.736545	0.467962	-2.880440
C	-5.306981	-0.927707	-3.276731
H	-6.084274	-0.206944	-3.036999
C	-5.652898	-2.234581	-3.639601
H	-6.695869	-2.533010	-3.694660
C	-4.636499	-3.150727	-3.922210
H	-4.885804	-4.171105	-4.202533
C	-3.299117	-2.766189	-3.840497
H	-2.517457	-3.492086	-4.051305
H	0.570208	4.294114	1.754753
H	-1.113167	3.550714	1.730415
C	0.161192	3.059915	0.070304
C	-0.150335	3.663733	1.241621
B	1.561382	3.386249	-0.533890
O	2.620959	3.843329	0.223331
O	1.867068	3.323875	-1.878730
C	3.778502	3.957207	-0.649416
C	3.113671	4.046989	-2.073876
C	-0.732268	2.146188	-0.686104
Pd	0.376381	0.287179	-0.540209
H	-0.659292	2.389610	-1.748130
C	-2.196138	2.059034	-0.277594
H	-2.291504	1.853845	0.791997
H	-2.672424	1.213866	-0.784554
H	-1.684366	0.921195	-2.894608
H	-0.823356	-1.958707	-3.461042
C	4.579728	5.186855	-0.234210
H	3.958826	6.084278	-0.211975
H	4.990352	5.037391	0.769052
H	5.416111	5.355027	-0.920705
C	4.614303	2.689104	-0.450552
H	4.880702	2.597495	0.605857
H	4.052541	1.795719	-0.730208
H	5.538254	2.717632	-1.035902
C	2.728671	5.476914	-2.470851
H	2.098774	5.438627	-3.364125
H	2.156313	5.964839	-1.676550
H	3.608722	6.087292	-2.694143
C	3.899870	3.383199	-3.198364
H	4.086601	2.328783	-2.993888
H	3.334814	3.451579	-4.132734
H	4.862483	3.884325	-3.344384
C	-3.027062	3.327405	-0.599990
H	-2.970655	3.527786	-1.677105
H	-2.581861	4.190191	-0.092650
C	-4.464869	3.148657	-0.176792
C	-4.823606	3.250372	1.176737
C	-5.457648	2.792874	-1.100179
C	-6.131583	3.002519	1.593980
C	-6.769178	2.544944	-0.687784
C	-7.111046	2.647131	0.662131
H	-4.063674	3.518460	1.907063
H	-5.198615	2.711997	-2.153676
H	-6.388418	3.088132	2.646923
H	-7.523824	2.273810	-1.421735
H	-8.130328	2.454273	0.985303

TS-g-a_1-1-a_Real

	Value
Charge	0
Electronic Energy, BS1 (a.u.)	-3385.636672
Thermal and entropic correction, BS1 (a.u.)	0.963876
Electronic Energy, BS2 (a.u.)	-3386.617314

Molecular Geometry in Cartesian Coordinates

P	-2.524707	0.035629	0.998597
P	0.006875	-2.073666	-0.536243
C	-3.232844	1.419849	2.014728
C	-4.005110	-0.629236	0.121864
C	-2.079490	-1.201307	2.309691
C	1.150079	-2.940550	0.629976
C	-1.481351	-3.194793	-0.448866
C	0.739278	-2.458758	-2.186028
C	-2.669155	2.693810	1.855996
C	-4.257796	1.248113	2.961707
C	-4.044585	-0.445660	-1.267883
C	-5.080442	-1.271296	0.752512
C	-1.870665	-2.573638	2.025607
C	-1.688724	-0.715228	3.571921
C	1.929992	-2.162750	1.493902
C	1.255556	-4.339704	0.688946
C	-1.887061	-3.981703	-1.539177
C	-2.247459	-3.256016	0.742329
C	0.008198	-2.124854	-3.343156
C	2.055361	-2.921654	-2.338530
H	-1.887348	2.843898	1.117665
C	-3.112952	3.770074	2.631667
C	-4.706774	2.322734	3.727525
H	-4.693615	0.267506	3.120535
C	-5.144360	-0.880762	-2.010730
H	-3.205215	0.035074	-1.762466
C	-6.178567	-1.705065	0.011421
H	-5.049551	-1.459975	1.820583
C	-1.318257	-3.398850	3.018120
H	-1.824503	0.334601	3.806859
C	-1.121864	-1.546582	4.536222
H	1.833785	-1.083111	1.455235
C	2.803854	-2.765636	2.401111
H	0.651470	-4.955201	0.028346
C	2.124078	-4.943235	1.598016
H	-1.302435	-3.981786	-2.450500
C	-3.024364	-4.786968	-1.482455
C	-3.372123	-4.092661	0.789493
H	-0.995518	-1.721637	-3.246329
C	0.562397	-2.287815	-4.611272
H	2.653130	-3.164692	-1.466829
C	2.618492	-3.059098	-3.610542
H	-2.659224	4.748435	2.502189
C	-4.131888	3.587521	3.566494
H	-5.498898	2.172401	4.456538
C	-6.214218	-1.507953	-1.371807
H	-5.160733	-0.733166	-3.087056
H	-7.002546	-2.206308	0.511015
C	-0.936574	-2.900386	4.261483
H	-0.829806	-1.132687	5.497528
H	3.403168	-2.149896	3.066171
C	2.899877	-4.156756	2.456203
H	2.196426	-6.026973	1.638755
C	-3.776610	-4.842787	-0.312047
H	-3.308560	-5.375919	-2.350158
H	-0.023578	-2.033489	-5.490360
C	1.874418	-2.751400	-4.750555
H	3.642526	-3.409298	-3.703552
H	-4.475208	4.422642	4.170670
H	-7.069027	-1.850688	-1.948194
H	-0.494431	-3.562089	5.000487
H	3.574831	-4.629284	3.164658
H	-4.664113	-5.466016	-0.251116
H	2.312299	-2.865475	-5.738277
H	-3.941999	-4.138370	1.712825
H	-1.165770	-4.448781	2.787927
C	0.767612	1.900135	1.247791
H	0.028074	1.327028	1.811199
H	0.418986	2.911232	1.055887
C	2.088230	1.781444	1.848143
C	3.061236	2.721284	1.797193
C	4.445730	2.571455	2.250031

C	4.910290	1.450437	2.971068
H	4.212916	0.674373	3.270957
C	6.254629	1.318405	3.305274
H	6.585144	0.442130	3.856837
C	7.179951	2.301417	2.935944
H	8.229115	2.192750	3.195506
C	6.737399	3.424922	2.234630
H	7.442898	4.198263	1.942729
C	5.389941	3.558681	1.901278
H	5.057968	4.432517	1.345482
H	-0.249505	1.031866	-3.852152
H	1.304891	0.255936	-3.235515
C	0.360326	1.667955	-1.916138
C	0.483475	0.939108	-3.056337
B	-0.803885	2.708405	-1.873802
O	-1.825005	2.694007	-2.798323
O	-0.860020	3.804897	-1.034321
C	-2.728771	3.787691	-2.480171
C	-1.802291	4.742895	-1.640449
C	1.367856	1.617684	-0.817514
Pd	-0.460246	0.230937	-0.084676
H	1.682765	2.632217	-0.583315
C	2.579047	0.711245	-0.955972
H	2.310076	-0.239904	-1.420153
H	2.967369	0.468771	0.034720
H	2.310895	0.820718	2.307764
H	2.828433	3.670746	1.313947
C	-3.244311	4.381211	-3.787266
H	-2.426756	4.659698	-4.454838
H	-3.865533	3.644243	-4.305328
H	-3.859101	5.266651	-3.594988
C	-3.887265	3.197245	-1.671593
H	-4.358979	2.400210	-2.252125
H	-3.537303	2.763446	-0.732834
H	-4.645889	3.952793	-1.446083
C	-0.964448	5.687947	-2.509694
H	-0.207035	6.167576	-1.882935
H	-0.448172	5.140470	-3.303803
H	-1.578692	6.469131	-2.966934
C	-2.511193	5.526271	-0.544523
H	-3.047833	4.873030	0.141468
H	-1.778959	6.102307	0.030013
H	-3.226490	6.230097	-0.982843
C	3.740851	1.356784	-1.751241
H	3.984507	2.323711	-1.295333
H	3.421044	1.555289	-2.780574
C	4.955626	0.458613	-1.734281
C	5.143116	-0.521451	-2.719007
C	5.876061	0.530489	-0.677628
C	6.222537	-1.405232	-2.652697
C	6.955143	-0.351839	-0.608014
C	7.132646	-1.324138	-1.595762
H	4.431574	-0.597596	-3.537432
H	5.743385	1.281006	0.097030
H	6.354303	-2.156134	-3.427755
H	7.655356	-0.276650	0.219797
H	7.972806	-2.011416	-1.543344

VI^{Pd}Real

	Value
Charge	0
Electronic Energy, BS1 (a.u.)	-3385.676384
Thermal and entropic correction, BS1 (a.u.)	0.958332
Electronic Energy, BS2 (a.u.)	-3386.656882

Molecular Geometry in Cartesian Coordinates

P	0.204585	0.434512	-0.673406
P	4.823005	-1.283462	0.696946

C	0.759910	-0.183239	-2.323977
C	1.223848	1.951626	-0.476170
C	0.685739	-0.938074	0.486719
C	5.207641	-0.453232	-0.916615
C	4.021738	0.120121	1.630049
C	6.478955	-1.385211	1.512478
C	0.598334	0.686161	-3.416540
C	1.220824	-1.486001	-2.559850
C	0.613369	3.106654	0.034170
C	2.570617	2.002568	-0.868048
C	1.728813	-1.002083	1.436976
C	-0.161705	-2.059251	0.338185
C	4.575373	-0.964438	-2.058919
C	6.029158	0.679484	-1.052202
C	4.775666	1.207930	2.105288
C	2.619484	0.141212	1.808464
C	6.567346	-1.413494	2.916146
C	7.650658	-1.597123	0.766877
H	0.240463	1.699031	-3.252523
C	0.890424	0.261760	-4.711576
C	1.503322	-1.912586	-3.860591
H	1.353217	-2.177928	-1.736348
C	1.348679	4.284222	0.181880
H	-0.433060	3.094154	0.315676
C	3.301901	3.178683	-0.712539
H	3.052637	1.128038	-1.287561
C	1.888282	-2.188387	2.180291
H	-0.981587	-2.022463	-0.371159
C	0.024242	-3.226965	1.069761
H	3.939335	-1.839247	-1.972015
C	4.729666	-0.344846	-3.302515
H	6.540436	1.085182	-0.185708
C	6.197503	1.289327	-2.294558
H	5.854918	1.197225	1.997086
C	4.171740	2.296946	2.726948
C	2.025121	1.242395	2.446442
H	5.676792	-1.251754	3.517167
C	7.790477	-1.625695	3.551816
H	7.612412	-1.586776	-0.318153
C	8.873978	-1.812170	1.405053
H	0.759406	0.947133	-5.544109
C	1.337420	-1.043102	-4.939369
H	1.855454	-2.927205	-4.024984
C	2.694336	4.321243	-0.184543
H	0.864994	5.169170	0.586068
H	4.347127	3.193954	-1.003638
C	1.062245	-3.291824	2.000914
H	-0.640402	-4.070025	0.908299
H	4.204392	-0.738793	-4.166690
C	5.538641	0.784342	-3.421225
H	6.836508	2.163804	-2.384226
C	2.785797	2.317107	2.893473
H	4.779490	3.126344	3.077618
H	7.837041	-1.633438	4.637491
C	8.950324	-1.824735	2.798728
H	9.769263	-1.966640	0.808733
H	1.551382	-1.378453	-5.950139
H	3.268372	5.235963	-0.064306
H	1.221011	-4.189771	2.590901
H	5.658646	1.270860	-4.385117
H	2.299891	3.162243	3.371629
H	9.903160	-1.988924	3.293917
H	0.948560	1.243847	2.584853
H	2.680689	-2.224370	2.921735
C	-2.818621	0.144755	-2.294483
H	-2.256862	0.735948	-3.023760
H	-3.885573	0.380016	-2.341742
C	-2.519259	-1.281683	-2.355645
C	-3.342722	-2.315367	-2.039429
C	-2.934982	-3.709052	-1.855753
C	-1.672523	-4.210593	-2.246655
H	-0.970548	-3.561812	-2.762080
C	-1.307603	-5.528056	-1.981805
H	-0.327880	-5.882857	-2.292762

C	-2.186524	-6.394704	-1.322061
H	-1.895896	-7.420626	-1.114125
C	-3.442956	-5.919757	-0.937060
H	-4.138963	-6.572914	-0.416818
C	-3.811168	-4.602527	-1.203572
H	-4.783030	-4.244163	-0.874965
H	-1.353086	2.049135	2.213857
H	-1.902124	0.301221	2.313619
C	-3.242380	1.670149	1.240552
C	-2.025844	1.277699	1.851743
B	-3.450323	3.171697	0.844698
O	-2.493829	4.144965	1.019229
O	-4.594225	3.634654	0.247257
C	-2.922397	5.298110	0.229845
C	-4.480035	5.086578	0.162664
C	-4.084637	0.705256	0.614855
Pd	-2.084441	0.755175	-0.361983
H	-4.911662	1.107540	0.031448
C	-4.274467	-0.727897	1.051057
H	-3.469819	-1.059203	1.714871
H	-4.229041	-1.383975	0.175896
H	-1.485244	-1.519665	-2.596284
H	-4.384702	-2.097145	-1.806646
C	-5.218312	5.655231	1.378314
H	-4.751775	5.327466	2.311906
H	-5.237269	6.748907	1.361809
H	-6.249713	5.291892	1.370052
C	-5.140448	5.549666	-1.130098
H	-6.216163	5.355714	-1.084404
H	-4.995390	6.625277	-1.274219
H	-4.739983	5.021977	-1.997536
C	-2.243025	5.174725	-1.138412
H	-2.492012	6.021312	-1.784618
H	-1.159083	5.152716	-1.004234
H	-2.536126	4.248530	-1.641863
C	-2.471139	6.568008	0.939800
H	-2.849327	7.454717	0.420864
H	-2.813255	6.595393	1.975851
H	-1.378106	6.620638	0.943792
C	-5.634701	-0.964879	1.755528
H	-5.646410	-0.423952	2.709673
H	-6.436646	-0.541952	1.138758
C	-5.879983	-2.440222	1.975447
C	-5.264164	-3.119257	3.036802
C	-6.663798	-3.179005	1.078126
C	-5.423447	-4.496529	3.195708
C	-6.829221	-4.557285	1.234626
C	-6.206290	-5.221525	2.293504
H	-4.653487	-2.560545	3.742599
H	-7.147016	-2.668301	0.248236
H	-4.937349	-5.004238	4.024530
H	-7.443420	-5.110918	0.529423
H	-6.332405	-6.293516	2.417033

TS_VI-VI’^{Pd}Real

	Value
Charge	0
Electronic Energy, BS1 (a.u.)	-3385.656447
Thermal and entropic correction, BS1 (a.u.)	0.963468
Electronic Energy, BS2 (a.u.)	-3386.638759

Molecular Geometry in Cartesian Coordinates

P	-0.134873	-0.241553	-0.398942
P	-4.868751	0.724631	1.115494
C	-0.704273	0.930810	-1.705287
C	-1.038824	-1.790083	-0.793954
C	-0.723703	0.532764	1.187664
C	-5.062454	0.514130	-0.716560

C	-4.100210	-0.912378	1.568678
C	-6.597564	0.520636	1.738183
C	-0.607481	0.506189	-3.041611
C	-1.053318	2.263344	-1.444764
C	-0.381058	-3.010537	-0.581301
C	-2.344013	-1.789705	-1.310393
C	-1.841292	0.220666	1.993899
C	0.105139	1.608000	1.577473
C	-4.398343	1.436432	-1.537132
C	-5.771395	-0.538546	-1.321723
C	-4.850892	-2.101481	1.581921
C	-2.714195	-0.985745	1.834636
C	-6.814450	0.087202	3.058932
C	-7.706809	0.933545	0.981851
H	-0.341972	-0.523091	-3.264657
C	-0.838392	1.397494	-4.087492
C	-1.281116	3.155650	-2.494823
H	-1.143272	2.617125	-0.424099
C	-1.042092	-4.214173	-0.829493
H	0.640620	-3.026203	-0.219436
C	-2.985536	-2.993827	-1.580395
H	-2.857329	-0.855191	-1.501829
C	-2.096598	1.018747	3.126800
H	0.990022	1.830213	0.988277
C	-0.169465	2.389126	2.694382
H	-3.844125	2.253753	-1.087471
C	-4.415527	1.299986	-2.928490
H	-6.301868	-1.258671	-0.707953
C	-5.798868	-0.669970	-2.709148
H	-5.920744	-2.058919	1.407633
C	-4.256854	-3.335529	1.830502
C	-2.128831	-2.237239	2.085596
H	-5.973905	-0.238395	3.665804
C	-8.100308	0.048600	3.597257
H	-7.569528	1.277671	-0.038633
C	-8.993468	0.898096	1.523927
H	-0.752648	1.053528	-5.114416
C	-1.163955	2.729747	-3.817668
H	-1.530647	4.188934	-2.274232
C	-2.346275	-4.207862	-1.324128
H	-0.529977	-5.154139	-0.642719
H	-3.998419	-2.977304	-1.970299
C	-1.289156	2.095153	3.474039
H	0.491725	3.209978	2.956109
H	-3.870043	2.008743	-3.542195
C	-5.111750	0.244517	-3.515500
H	-6.350447	-1.488935	-3.163229
C	-2.883991	-3.406085	2.073497
H	-4.861891	-4.237925	1.829589
H	-8.245747	-0.299729	4.616303
C	-9.196592	0.453380	2.831195
H	-9.838416	1.215752	0.918797
H	-1.320310	3.430970	-4.632191
H	-2.860590	-5.144808	-1.520009
H	-1.521001	2.685785	4.355405
H	-5.121855	0.131738	-4.595973
H	-2.404143	-4.361973	2.259506
H	-10.198396	0.422964	3.250154
H	-1.064474	-2.281002	2.293926
H	-2.948066	0.763134	3.750049
C	2.671201	0.690067	-1.911147
H	2.131926	0.253202	-2.764364
H	3.741044	0.576897	-2.131244
C	2.321744	2.152135	-1.796902
C	2.541551	3.081450	-2.743695
C	2.152774	4.498923	-2.694969
C	1.555060	5.103163	-1.569149
H	1.382871	4.518537	-0.671016
C	1.175982	6.442721	-1.587775
H	0.717495	6.881473	-0.705116
C	1.382420	7.225000	-2.729237
H	1.086111	8.270102	-2.739884
C	1.977046	6.646252	-3.851706
H	2.146802	7.240090	-4.746150

C	2.356268	5.304045	-3.831743
H	2.815043	4.862551	-4.713471
H	1.734354	-2.593631	1.852618
H	2.196512	-0.928723	2.468834
C	3.497366	-1.772669	0.916895
C	2.330018	-1.696609	1.710703
B	3.740501	-3.044798	0.035248
O	2.832079	-4.070386	-0.093942
O	4.868149	-3.219837	-0.725153
C	3.257814	-4.860507	-1.247388
C	4.801282	-4.560590	-1.295169
C	4.225075	-0.591788	0.564825
Pd	2.176583	-0.483973	-0.232537
H	5.024753	-0.732644	-0.160738
C	4.418995	0.607257	1.467691
H	3.543250	0.783548	2.100543
H	4.547684	1.509127	0.857281
H	1.820918	2.477648	-0.886651
H	3.022174	2.766516	-3.671640
C	5.620910	-5.469799	-0.374396
H	5.189913	-5.505535	0.630402
H	5.680986	-6.489632	-0.765654
H	6.636006	-5.071170	-0.293127
C	5.409720	-4.524086	-2.691807
H	6.479137	-4.303940	-2.622496
H	5.294046	-5.492633	-3.189117
H	4.946835	-3.753766	-3.311129
C	2.504827	-4.309971	-2.463561
H	2.749038	-4.870585	-3.370477
H	1.429402	-4.388524	-2.289344
H	2.739970	-3.253972	-2.627148
C	2.888291	-6.317757	-1.002559
H	3.269121	-6.952606	-1.809148
H	3.285153	-6.681251	-0.052988
H	1.799396	-6.423148	-0.976713
C	5.659298	0.446943	2.386172
H	5.539536	-0.468439	2.977634
H	6.550486	0.310532	1.761863
C	5.836318	1.636811	3.300093
C	5.181728	1.687712	4.539720
C	6.603250	2.742371	2.905933
C	5.292572	2.808366	5.364480
C	6.717375	3.865545	3.727443
C	6.061918	3.902347	4.960488
H	4.583694	0.837145	4.859899
H	7.116316	2.719432	1.947078
H	4.781594	2.826091	6.323656
H	7.319976	4.710786	3.405630
H	6.151772	4.774584	5.602038

VI'^{Pd}Real

	Value
Charge	0
Electronic Energy, BS1 (a.u.)	-3385.676536
Thermal and entropic correction, BS1 (a.u.)	0.959412
Electronic Energy, BS2 (a.u.)	-3386.656605

Molecular Geometry in Cartesian Coordinates

P	0.009259	0.500237	0.784514
P	-4.677106	-0.751421	-0.708630
C	-0.630888	-0.057584	2.421578
C	-0.834256	2.116277	0.560999
C	-0.587296	-0.809355	-0.396023
C	-5.026404	0.108144	0.898003
C	-3.746439	0.581413	-1.624184
C	-6.317992	-0.714921	-1.559877
C	-0.409971	0.795793	3.516899
C	-1.220563	-1.307773	2.647321

C	-0.096891	3.189282	0.038281
C	-2.169223	2.319730	0.942575
C	-1.572155	-0.745087	-1.404981
C	0.123402	-2.017903	-0.221039
C	-4.480157	-0.465017	2.055115
C	-5.736759	1.315586	1.016633
C	-4.397349	1.728052	-2.113205
C	-2.345695	0.479660	-1.783805
C	-6.375937	-0.747774	-2.965063
C	-7.520903	-0.812851	-0.841077
H	0.050229	1.767716	3.359996
C	-0.770544	0.405986	4.805115
C	-1.575140	-1.700205	3.941118
H	-1.397896	-1.988797	1.823737
C	-0.696071	4.439202	-0.126435
H	0.941587	3.053549	-0.242396
C	-2.763472	3.568833	0.772346
H	-2.746950	1.508092	1.367899
C	-1.799233	-1.890638	-2.194590
H	0.882392	-2.081651	0.552151
C	-0.127574	-3.141844	-0.999616
H	-3.931302	-1.398167	1.982264
C	-4.608102	0.164162	3.296981
H	-6.182347	1.771356	0.138995
C	-5.878619	1.937079	2.256437
H	-5.475161	1.809157	-2.022268
C	-3.693417	2.758160	-2.729910
C	-1.650004	1.522126	-2.416517
H	-5.460509	-0.673186	-3.545621
C	-7.598074	-0.853581	-3.628443
H	-7.506772	-0.796063	0.244449
C	-8.743644	-0.921504	-1.506972
H	-0.591123	1.077073	5.640214
C	-1.349099	-0.848628	5.022814
H	-2.025668	-2.676400	4.096783
C	-2.030110	4.630336	0.235632
H	-0.116796	5.260169	-0.539854
H	-3.801838	3.704202	1.057375
C	-1.097690	-3.075037	-2.001940
H	0.440969	-4.049493	-0.823614
H	-4.148793	-0.281610	4.173394
C	-5.304272	1.367660	3.398557
H	-6.430757	2.870094	2.332261
C	-2.309073	2.656684	-2.877926
H	-4.222381	3.634943	-3.092806
H	-7.619969	-0.866847	-4.714874
C	-8.788443	-0.939595	-2.901899
H	-9.662918	-0.988258	-0.931233
H	-1.618366	-1.158127	6.028707
H	-2.497893	5.602159	0.103660
H	-1.303791	-3.936129	-2.631183
H	-5.402411	1.862004	4.360940
H	-1.745220	3.453761	-3.353180
H	-9.740490	-1.021072	-3.418630
H	-0.576614	1.429132	-2.542615
H	-2.546318	-1.829018	-2.980254
C	2.953137	-0.050318	2.435668
H	2.259262	0.318551	3.195445
H	3.941737	0.402692	2.558117
C	2.982192	-1.507573	2.326891
C	2.064744	-2.378901	2.821429
C	1.960304	-3.797488	2.479755
C	2.603780	-4.365458	1.356081
H	3.210619	-3.738768	0.710678
C	2.445329	-5.711248	1.034338
H	2.948821	-6.109866	0.157193
C	1.626179	-6.535742	1.813801
H	1.494483	-7.582943	1.556268
C	0.968603	-5.990192	2.919728
H	0.322968	-6.616086	3.531010
C	1.131563	-4.644652	3.245282
H	0.611263	-4.229929	4.105533
H	1.664839	1.798979	-2.132727
H	1.954205	-0.012445	-2.175484

C	3.524376	1.189242	-1.222746
C	2.235933	0.952888	-1.763719
B	3.958264	2.658980	-0.899368
O	3.141105	3.749047	-1.090591
O	5.171798	2.978109	-0.346959
C	3.746386	4.859059	-0.357395
C	5.261360	4.433773	-0.310931
C	4.252135	0.136996	-0.602685
Pd	2.308787	0.547713	0.474945
H	5.181448	0.421307	-0.113974
C	4.121201	-1.329246	-0.969578
H	3.082529	-1.664834	-0.880872
H	4.692678	-1.928200	-0.253705
H	3.789864	-1.916406	1.721415
H	1.295884	-1.995847	3.489038
C	6.045572	4.854471	-1.557569
H	5.518157	4.566463	-2.471640
H	6.218446	5.934453	-1.578822
H	7.015750	4.349851	-1.553839
C	6.007884	4.841201	0.953560
H	7.044076	4.494780	0.897457
H	6.019629	5.930726	1.061021
H	5.554926	4.405417	1.845839
C	3.088793	4.884151	1.026741
H	3.471942	5.707610	1.636096
H	2.010109	5.015865	0.912930
H	3.255714	3.943008	1.559260
C	3.459836	6.151926	-1.110155
H	3.964694	6.996866	-0.630802
H	3.783769	6.094522	-2.150859
H	2.384183	6.352727	-1.101946
C	4.597526	-1.692861	-2.399463
H	4.065934	-1.062712	-3.121291
H	5.666404	-1.473984	-2.503059
C	4.316811	-3.154198	-2.667644
C	3.022286	-3.568778	-3.017991
C	5.297812	-4.133662	-2.464847
C	2.716976	-4.922140	-3.162575
C	4.997561	-5.490494	-2.610091
C	3.705170	-5.889831	-2.958186
H	2.244312	-2.823356	-3.164591
H	6.304188	-3.830026	-2.185645
H	1.707901	-5.221524	-3.433034
H	5.772879	-6.234931	-2.450021
H	3.470094	-6.944486	-3.071351

TS-γγ'-RR_Real

	Value
Charge	0
Electronic Energy, BS1 (a.u.)	-3385.645175
Thermal and entropic correction, BS1 (a.u.)	0.954768
Electronic Energy, BS2 (a.u.)	-3386.626738

Molecular Geometry in Cartesian Coordinates

P	1.004447	-1.095386	0.935610
P	5.197279	1.692797	0.039670
C	1.918134	-1.618349	2.458628
C	1.912503	-2.011403	-0.378473
C	1.279306	0.743296	0.859427
C	5.926437	0.105145	0.661979
C	4.159056	1.064255	-1.377820
C	6.615140	2.481402	-0.846273
C	1.989850	-3.001022	2.708301
C	2.457524	-0.735279	3.403579
C	1.153989	-2.686290	-1.348166
C	3.310837	-2.119482	-0.407237
C	2.022838	1.523597	-0.052029
C	0.536085	1.408375	1.860664

C	5.569062	-0.292637	1.958363
C	6.742046	-0.746792	-0.103165
C	4.743957	0.601217	-2.569580
C	2.755127	0.982460	-1.238989
C	6.371424	3.365789	-1.912746
C	7.933705	2.348059	-0.379654
H	1.577396	-3.701539	1.986721
C	2.590129	-3.484995	3.868652
C	3.047880	-1.222646	4.573442
H	2.426072	0.334822	3.233564
C	1.783882	-3.429728	-2.347668
H	0.069826	-2.630598	-1.316669
C	3.937171	-2.856482	-1.410271
H	3.912002	-1.628695	0.347893
C	2.006769	2.925910	0.087764
H	-0.072801	0.818413	2.540823
C	0.545745	2.791960	1.992303
H	4.939672	0.353122	2.562418
C	5.986741	-1.524237	2.471222
H	7.043090	-0.454265	-1.103713
C	7.172593	-1.968400	0.412479
H	5.818372	0.673278	-2.700092
C	3.974009	0.063517	-3.597068
C	1.989094	0.449146	-2.288212
H	5.361325	3.488442	-2.293623
C	7.415125	4.075628	-2.506220
H	8.151560	1.677659	0.446190
C	8.977016	3.061293	-0.973549
H	2.640119	-4.556439	4.041362
C	3.117360	-2.595799	4.810561
H	3.460329	-0.522360	5.294717
C	3.176450	-3.510014	-2.384118
H	1.185122	-3.944501	-3.093979
H	5.020780	-2.914967	-1.426379
C	1.289379	3.560423	1.093910
H	-0.038983	3.264617	2.775896
H	5.670511	-1.826777	3.464375
C	6.786338	-2.364628	1.697849
H	7.802774	-2.616620	-0.190648
C	2.587490	-0.016162	-3.454292
H	4.454048	-0.292631	-4.504210
H	7.205018	4.745665	-3.335564
C	8.723628	3.925304	-2.040202
H	9.990511	2.936962	-0.601565
H	3.576596	-2.971867	5.720375
H	3.669371	-4.083890	-3.164083
H	1.297290	4.643855	1.167037
H	7.109323	-3.324365	2.091239
H	1.974716	-0.436553	-4.245953
H	9.536606	4.477081	-2.503513
H	0.910223	0.402169	-2.175101
H	2.568768	3.517746	-0.628360
C	-3.233557	-2.354943	1.570319
H	-2.666110	-3.072662	2.179645
H	-3.769469	-2.800454	0.733869
C	-3.828675	-1.270452	2.224393
C	-4.855583	-0.495713	1.648232
C	-5.627186	0.523398	2.376573
C	-5.207347	1.102829	3.591836
H	-4.285445	0.770329	4.059671
C	-5.949959	2.111866	4.203835
H	-5.601528	2.537912	5.141549
C	-7.129641	2.581417	3.618997
H	-7.703241	3.371402	4.095716
C	-7.559891	2.021637	2.412729
H	-8.472179	2.377736	1.941041
C	-6.821250	1.008830	1.804687
H	-7.159968	0.586961	0.861211
H	-1.210838	-0.711177	-1.464870
H	-1.152453	0.685359	-0.300087
C	-3.118312	-0.121216	-0.711810
C	-1.704223	-0.185229	-0.648030
B	-3.833616	-1.155637	-1.604505
O	-3.198360	-1.892342	-2.585295

O	-5.174984	-1.463309	-1.498967
C	-4.123453	-2.920832	-3.029842
C	-5.521271	-2.312104	-2.627280
C	-3.878669	0.755646	0.094981
Pd	-1.273132	-1.575455	0.986976
H	-4.888448	0.960698	-0.247014
C	-3.233001	1.883777	0.858978
H	-3.904592	2.225472	1.650934
H	-2.312142	1.542010	1.343666
H	-3.409645	-0.969794	3.183600
H	-5.405535	-0.984958	0.849371
C	-6.560072	-3.329182	-2.167535
H	-7.486379	-2.813316	-1.896398
H	-6.789073	-4.040080	-2.968220
H	-6.217350	-3.884946	-1.292406
C	-3.771656	-4.197642	-2.257620
H	-4.385400	-5.045992	-2.575171
H	-2.721009	-4.443353	-2.440025
H	-3.899290	-4.057601	-1.180901
C	-3.925851	-3.140119	-4.526330
H	-2.928003	-3.550788	-4.710119
H	-4.661936	-3.851691	-4.914933
H	-4.012377	-2.206340	-5.085307
C	-6.111604	-1.390036	-3.699933
H	-5.376447	-0.649456	-4.028782
H	-6.459369	-1.951193	-4.572355
H	-6.962469	-0.851052	-3.273242
C	-2.909434	3.100792	-0.047342
H	-2.438416	3.876261	0.570223
H	-2.177190	2.808146	-0.807142
C	-4.152700	3.650918	-0.711730
C	-5.114567	4.338378	0.043551
C	-4.414745	3.417535	-2.068322
C	-6.301080	4.783170	-0.539849
C	-5.600647	3.862704	-2.657925
C	-6.548480	4.547592	-1.895284
H	-4.938179	4.509698	1.103032
H	-3.685769	2.872535	-2.663029
H	-7.034791	5.309707	0.064823
H	-5.784773	3.670456	-3.711905
H	-7.472570	4.892828	-2.351126

3-(RR)-Pd_Real

	Value
Charge	0
Electronic Energy, BS1 (a.u.)	-3385.691654
Thermal and entropic correction, BS1 (a.u.)	0.959628
Electronic Energy, BS2 (a.u.)	-3386.668194

Molecular Geometry in Cartesian Coordinates

P	-0.860457	0.008881	0.586742
P	-5.744298	-1.384563	-0.117412
C	-1.482241	0.155783	2.327787
C	-1.670179	1.459017	-0.212805
C	-1.594357	-1.605392	0.018018
C	-5.960553	0.061949	1.023531
C	-4.790734	-0.592436	-1.511628
C	-7.425080	-1.569482	-0.866012
C	-1.169545	1.347596	3.007154
C	-2.124243	-0.869110	3.034595
C	-0.910081	2.210411	-1.122039
C	-2.976877	1.870686	0.090683
C	-2.673146	-1.873650	-0.854109
C	-0.908128	-2.700311	0.588859
C	-5.377136	-0.040832	2.294174
C	-6.604900	1.261445	0.673886
C	-5.404035	0.315626	-2.393231
C	-3.406879	-0.843316	-1.654144

C	-7.564653	-2.139987	-2.144300
C	-8.589096	-1.297938	-0.127678
H	-0.658287	2.148195	2.478623
C	-1.501255	1.512427	4.349771
C	-2.442113	-0.709120	4.387654
H	-2.383887	-1.796928	2.537204
C	-1.460383	3.334283	-1.742296
H	0.117066	1.930126	-1.331345
C	-3.524246	2.990229	-0.533597
H	-3.570893	1.318019	0.808311
C	-3.029826	-3.215614	-1.094714
H	-0.058739	-2.503270	1.237955
C	-1.285736	-4.017161	0.351981
H	-4.876377	-0.959763	2.580177
C	-5.403988	1.035651	3.185567
H	-7.075898	1.360578	-0.298241
C	-6.647433	2.330620	1.567558
H	-6.469815	0.500603	-2.312202
C	-4.678746	0.974176	-3.381570
C	-2.689192	-0.176162	-2.660724
H	-6.681682	-2.359168	-2.738197
C	-8.826457	-2.409727	-2.673733
H	-8.512645	-0.862450	0.863938
C	-9.851641	-1.570930	-0.658249
H	-1.256056	2.442985	4.854426
C	-2.135357	0.479713	5.049318
H	-2.938655	-1.517006	4.918561
C	-2.769304	3.722612	-1.454324
H	-0.861999	3.903701	-2.448344
H	-4.542731	3.282683	-0.298834
C	-2.361723	-4.278032	-0.498533
H	-0.736782	-4.830381	0.818011
H	-4.915518	0.944285	4.150404
C	-6.036716	2.223534	2.822174
H	-7.149759	3.251581	1.283574
C	-3.310140	0.731319	-3.511862
H	-5.178226	1.674806	-4.044821
H	-8.910646	-2.840829	-3.667722
C	-9.976555	-2.124858	-1.933350
H	-10.738866	-1.345132	-0.072674
H	-2.382647	0.601935	6.100111
H	-3.199999	4.594468	-1.939427
H	-2.667432	-5.298871	-0.708153
H	-6.056461	3.064062	3.510224
H	-2.729680	1.242471	-4.273935
H	-10.959368	-2.333398	-2.346483
H	-1.628244	-0.380969	-2.766270
H	-3.848404	-3.412822	-1.780277
C	2.303165	-0.001842	2.609011
H	1.501333	-0.386242	3.232437
H	2.633140	1.013162	2.823711
C	3.054096	-0.830217	1.803183
C	4.396045	-0.480693	1.177235
C	5.598965	-1.159578	1.827840
C	5.485720	-2.159003	2.802716
H	4.505338	-2.461039	3.157235
C	6.621491	-2.773649	3.339267
H	6.506549	-3.545595	4.095869
C	7.894339	-2.401656	2.907420
H	8.776832	-2.882037	3.321036
C	8.023012	-1.400798	1.940107
H	9.007522	-1.099851	1.592239
C	6.888823	-0.787497	1.411438
H	7.002520	-0.012949	0.657863
H	1.566023	0.324778	-2.350454
H	2.004116	-1.410496	-1.968583
C	3.200539	0.113263	-0.954987
C	2.120707	-0.359522	-1.714426
B	3.368891	1.656840	-0.880552
O	2.473397	2.562321	-1.423541
O	4.424597	2.283344	-0.249182
C	2.788144	3.862553	-0.849761
C	4.317005	3.708843	-0.519241
C	4.333009	-0.749952	-0.365056

Pd	1.481790	-0.034424	0.417628
H	5.274216	-0.376390	-0.785462
C	4.249570	-2.248066	-0.700897
H	4.868266	-2.805173	0.010795
H	3.224564	-2.614497	-0.570130
H	2.788783	-1.888172	1.775950
H	4.541842	0.596995	1.293084
C	4.793882	4.471745	0.710898
H	5.862290	4.293025	0.865051
H	4.644588	5.548736	0.581349
H	4.266329	4.150483	1.610980
C	1.917444	4.020548	0.403227
H	2.073684	4.991788	0.881965
H	0.865480	3.939113	0.119967
H	2.128858	3.228749	1.128190
C	2.453241	4.943141	-1.870534
H	1.372083	4.971560	-2.035890
H	2.764798	5.927939	-1.507150
H	2.937328	4.754529	-2.830588
C	5.224860	3.998996	-1.718753
H	4.885341	3.458400	-2.606973
H	5.259598	5.067653	-1.950660
H	6.239134	3.662643	-1.485636
C	4.740412	-2.588407	-2.126799
H	4.589285	-3.661329	-2.300930
H	4.131951	-2.057514	-2.866201
C	6.200532	-2.240026	-2.327081
C	7.201860	-2.972406	-1.672058
C	6.585169	-1.140548	-3.106258
C	8.545537	-2.617776	-1.791042
C	7.929660	-0.780038	-3.228408
C	8.915081	-1.517661	-2.570037
H	6.922423	-3.820907	-1.052210
H	5.820842	-0.557117	-3.614322
H	9.303983	-3.196594	-1.270796
H	8.205110	0.078212	-3.835702
H	9.961234	-1.238800	-2.661821

TS-γγ'-SR_Real

	Value
Charge	0
Electronic Energy, BS1 (a.u.)	-3385.638623
Thermal and entropic correction, BS1 (a.u.)	0.960709
Electronic Energy, BS2 (a.u.)	-3386.619089

Molecular Geometry in Cartesian Coordinates

P	-1.110127	-1.160117	-0.536097
P	-5.587426	1.217319	-0.888144
C	-2.100215	-2.517748	-1.312437
C	-1.759723	-1.130293	1.186007
C	-1.595716	0.354866	-1.499147
C	-6.097578	-0.469698	-0.310435
C	-4.395774	1.703499	0.462329
C	-7.063405	2.254203	-0.481602
C	-2.073867	-3.771346	-0.673846
C	-2.765975	-2.402414	-2.540525
C	-0.840728	-0.941615	2.231058
C	-3.111920	-1.346185	1.491779
C	-2.417500	1.446174	-1.140154
C	-0.999841	0.356710	-2.780071
C	-5.806149	-1.546859	-1.159257
C	-6.688980	-0.736317	0.936901
C	-4.844521	2.038941	1.752052
C	-3.003985	1.673643	0.218019
C	-6.904668	3.631582	-0.242951
C	-8.367453	1.736899	-0.555700
H	-1.563967	-3.880596	0.279723
C	-2.698718	-4.876266	-1.248109

C	-3.381433	-3.515410	-3.120683
H	-2.813319	-1.446898	-3.050477
C	-1.275337	-0.947608	3.557666
H	0.211938	-0.784883	2.016766
C	-3.541013	-1.345061	2.818131
H	-3.831224	-1.513293	0.699366
C	-2.626221	2.472154	-2.083147
H	-0.335602	-0.460681	-3.049346
C	-1.231808	1.370697	-3.702392
H	-5.348669	-1.359288	-2.125242
C	-6.068874	-2.862157	-0.764915
H	-6.935329	0.081051	1.606171
C	-6.964185	-2.046222	1.326503
H	-5.909815	2.091220	1.949659
C	-3.952889	2.323354	2.782092
C	-2.116291	1.972814	1.264361
H	-5.907239	4.058010	-0.179395
C	-8.012844	4.459016	-0.063497
H	-8.521414	0.678763	-0.744275
C	-9.476123	2.567518	-0.378757
H	-2.671378	-5.834608	-0.737080
C	-3.349901	-4.754473	-2.479845
H	-3.892331	-3.406280	-4.073476
C	-2.624588	-1.145779	3.854075
H	-0.556396	-0.795887	4.357831
H	-4.593401	-1.499067	3.034369
C	-2.056857	2.440454	-3.350103
H	-0.760466	1.331650	-4.680093
H	-5.806220	-3.682024	-1.425299
C	-6.644564	-3.113532	0.479682
H	-7.421492	-2.236829	2.293847
C	-2.578904	2.284735	2.538156
H	-4.328501	2.573262	3.770402
H	-7.866243	5.518269	0.130249
C	-9.304496	3.930124	-0.129325
H	-10.475958	2.144975	-0.432978
H	-3.827732	-5.618041	-2.933768
H	-2.962573	-1.147229	4.886827
H	-2.241480	3.250783	-4.049197
H	-6.845454	-4.134606	0.791670
H	-1.870917	2.499248	3.332916
H	-10.167696	4.574403	0.011521
H	-1.049339	1.958376	1.063608
H	-3.245473	3.315507	-1.793139
C	3.012597	-2.727872	-1.137854
H	2.710927	-3.580769	-0.521077
H	2.586351	-2.711946	-2.150345
C	4.318228	-2.261074	-0.993787
C	4.898225	-1.298093	-1.829414
H	4.382944	-1.126516	-2.774699
H	1.004084	0.751730	0.444117
H	1.402523	0.984773	-1.311420
C	3.061722	0.417124	-0.022848
C	1.687973	0.515317	-0.369599
B	3.406892	-0.072635	1.400280
O	2.522841	-0.085330	2.464627
O	4.654906	-0.547224	1.750945
C	3.158055	-0.828943	3.542819
C	4.684130	-0.664760	3.198242
C	4.134301	0.642328	-0.906362
Pd	1.173105	-1.569486	-0.648994
H	5.101503	0.718506	-0.424898
C	4.014729	1.465990	-2.163330
H	4.865915	1.255274	-2.819783
H	3.104939	1.219634	-2.722815
H	4.866117	-2.549768	-0.098219
C	5.563635	-1.851027	3.574854
H	6.601483	-1.645199	3.295659
H	5.533388	-2.029384	4.654797
H	5.250524	-2.761965	3.061056
C	2.668659	-2.277304	3.431438
H	3.072905	-2.903079	4.232575
H	1.577324	-2.294184	3.500116
H	2.949884	-2.710820	2.467540

C	2.733803	-0.222153	4.874847
H	1.661500	-0.376262	5.028952
H	3.265443	-0.700469	5.703990
H	2.927039	0.851662	4.907685
C	5.286451	0.641308	3.728873
H	4.653111	1.496151	3.474935
H	5.423947	0.615012	4.814052
H	6.260348	0.797840	3.256674
C	4.022642	2.986243	-1.838409
H	4.030749	3.547523	-2.781695
H	3.096866	3.244522	-1.313256
C	5.216644	3.367936	-0.989360
C	6.511491	3.332317	-1.527044
C	5.070425	3.658054	0.373599
C	7.627772	3.573430	-0.726219
C	6.184693	3.906397	1.178806
C	7.468288	3.861793	0.631818
H	6.646944	3.088764	-2.577927
H	4.074389	3.672634	0.809055
H	8.622585	3.524939	-1.160495
H	6.049093	4.126536	2.234567
H	8.337230	4.046600	1.257486
C	6.330567	-0.961868	-1.834207
C	7.105781	-0.938623	-0.654677
C	6.954822	-0.578497	-3.037183
C	8.446726	-0.565233	-0.688846
C	8.299112	-0.203354	-3.068596
C	9.054967	-0.196860	-1.894604
H	6.630539	-1.164255	0.295250
H	6.375582	-0.584953	-3.958001
H	9.019599	-0.544202	0.235239
H	8.755093	0.083863	-4.013113
H	10.100499	0.098584	-1.915017

3-(SR)-Pd_Real

	Value
Charge	0
Electronic Energy, BS1 (a.u.)	-3385.699007
Thermal and entropic correction, BS1 (a.u.)	0.96395
0Electronic Energy, BS2 (a.u.)	-3386.668194

Molecular Geometry in Cartesian Coordinates

P	-0.870499	-0.682865	0.344793
P	-5.733510	-0.384468	-1.167599
C	-1.611698	-2.125776	1.242688
C	-1.566231	0.746989	1.275472
C	-1.607329	-0.831295	-1.358507
C	-5.946847	-0.733361	0.641844
C	-4.696929	1.164802	-1.106094
C	-7.387011	0.288508	-1.650586
C	-1.362034	-2.208342	2.624698
C	-2.283673	-3.184869	0.618723
C	-0.726816	1.848291	1.507709
C	-2.867345	0.756156	1.800174
C	-2.653006	-0.111162	-1.978374
C	-0.972105	-1.856237	-2.094054
C	-5.455608	-1.958073	1.115186
C	-6.506055	0.168856	1.563698
C	-5.241642	2.394439	-0.693837
C	-3.317575	1.103465	-1.408297
C	-7.491980	1.165960	-2.745222
C	-8.570784	-0.172334	-1.050849
H	-0.833184	-1.400794	3.124042
C	-1.778649	-3.315036	3.360486
C	-2.686574	-4.303650	1.355532
H	-2.500517	-3.146383	-0.442918
C	-1.192390	2.950833	2.226657
H	0.292954	1.843407	1.135205

C	-3.330362	1.861682	2.511697
H	-3.522954	-0.093300	1.650974
C	-3.033959	-0.467504	-3.287456
H	-0.144388	-2.391647	-1.635747
C	-1.372808	-2.202785	-3.379583
H	-5.020109	-2.666110	0.418057
C	-5.491786	-2.266949	2.478200
H	-6.903673	1.117767	1.220664
C	-6.555260	-0.142608	2.921550
H	-6.303928	2.464217	-0.485179
C	-4.453261	3.533544	-0.562733
C	-2.535423	2.263084	-1.277304
H	-6.592657	1.539633	-3.227145
C	-8.738584	1.584044	-3.210202
H	-8.521217	-0.854024	-0.207254
C	-9.818370	0.244857	-1.519717
H	-1.580438	-3.356754	4.428003
C	-2.437377	-4.373797	2.725943
H	-3.203614	-5.116315	0.852307
C	-2.496100	2.962665	2.724569
H	-0.533054	3.798086	2.395117
H	-4.345951	1.857514	2.894570
C	-2.419703	-1.502527	-3.981899
H	-0.864340	-3.003885	-3.908321
H	-5.077342	-3.207799	2.825189
C	-6.038169	-1.358239	3.383199
H	-6.989981	0.565288	3.622263
C	-3.088317	3.464820	-0.848700
H	-4.900507	4.468377	-0.236786
H	-8.795474	2.270544	-4.050708
C	-9.908285	1.125977	-2.598472
H	-10.721086	-0.118410	-1.035808
H	-2.750066	-5.244099	3.296103
H	-2.861194	3.822400	3.279923
H	-2.742946	-1.747992	-4.989277
H	-6.063290	-1.592252	4.443799
H	-2.458239	4.342955	-0.744493
H	-10.879215	1.452556	-2.960025
H	-1.478660	2.207770	-1.519334
H	-3.826700	0.101977	-3.763077
C	2.223440	-2.543607	1.250295
H	1.949251	-2.601506	2.300712
H	1.837091	-3.343023	0.620686
C	3.282814	-1.748058	0.837693
C	3.983648	-1.862007	-0.517770
H	3.337076	-2.468430	-1.165650
H	1.357674	1.789554	-1.056166
H	1.612970	0.444623	-2.275084
C	3.079659	0.551912	-0.663932
C	1.880659	0.871101	-1.310522
B	3.487409	1.385715	0.576301
O	2.732502	2.405822	1.128116
O	4.651724	1.152534	1.282710
C	3.280824	2.664898	2.452429
C	4.774063	2.200503	2.283597
C	4.130940	-0.453359	-1.188050
Pd	1.455730	-0.642838	0.285603
H	5.098210	-0.051096	-0.874428
C	4.188691	-0.593669	-2.714779
H	4.926985	-1.370688	-2.950807
H	3.226673	-0.956592	-3.099842
H	3.820872	-1.168967	1.585059
C	5.413645	1.604184	3.531808
H	6.440850	1.300027	3.308650
H	5.446429	2.340400	4.341615
H	4.870341	0.723984	3.880680
C	2.492083	1.796763	3.440212
H	2.824670	1.956035	4.470183
H	1.431619	2.052429	3.376840
H	2.591660	0.735072	3.195385
C	3.096619	4.142096	2.777357
H	2.029767	4.370686	2.862870
H	3.572558	4.390710	3.731547
H	3.516673	4.781937	1.999159

C	5.676437	3.282389	1.682331
H	5.215420	3.727384	0.796704
H	5.890490	4.076376	2.404187
H	6.620055	2.825539	1.373201
C	4.573139	0.706074	-3.453438
H	4.673586	0.483153	-4.523471
H	3.760805	1.433172	-3.356551
C	5.856856	1.317905	-2.933349
C	7.080681	0.648412	-3.086190
C	5.847566	2.533598	-2.237315
C	8.258740	1.177266	-2.559351
C	7.026419	3.071428	-1.713907
C	8.236212	2.393524	-1.870557
H	7.106735	-0.300773	-3.616043
H	4.905736	3.059279	-2.100066
H	9.195120	0.640316	-2.685598
H	6.996957	4.016737	-1.179190
H	9.153471	2.807603	-1.461039
C	5.324674	-2.572320	-0.393510
C	6.357443	-2.007839	0.374466
C	5.564750	-3.788655	-1.043553
C	7.591123	-2.646891	0.487936
C	6.800995	-4.431446	-0.932111
C	7.818507	-3.862158	-0.165573
H	6.190547	-1.056986	0.874575
H	4.775859	-4.235854	-1.644087
H	8.379353	-2.194160	1.083822
H	6.967421	-5.374724	-1.445807
H	8.781068	-4.358636	-0.078063

TS-γα’-11’-b_Real

	Value
Charge	0
Electronic Energy, BS1 (a.u.)	-3385.632128
Thermal and entropic correction, BS1 (a.u.)	0.959866
Electronic Energy, BS2 (a.u.)	-3386.612426

Molecular Geometry in Cartesian Coordinates

P	-0.126124	0.455809	0.434223
P	-4.985434	-1.105192	-0.502904
C	-0.547460	-0.005498	2.179229
C	-1.150651	1.972966	0.212961
C	-0.784083	-0.999775	-0.518473
C	-5.377694	-0.079138	0.993710
C	-4.124728	0.138848	-1.595823
C	-6.631818	-1.256908	-1.331256
C	0.028296	0.776519	3.196034
C	-1.355861	-1.091436	2.537833
C	-0.575338	3.075951	-0.436488
C	-2.454617	2.080344	0.717135
C	-1.898165	-1.112258	-1.379106
C	0.005508	-2.144761	-0.281992
C	-4.753350	-0.454072	2.192799
C	-6.209286	1.054305	0.996202
C	-4.825303	1.227687	-2.144961
C	-2.734102	0.040869	-1.833617
C	-6.716848	-1.376472	-2.729960
C	-7.806734	-1.418035	-0.576617
H	0.674537	1.610216	2.928977
C	-0.209891	0.486539	4.538462
C	-1.590272	-1.385270	3.884556
H	-1.790188	-1.723163	1.771096
C	-1.301872	4.256523	-0.600298
H	0.442206	3.006696	-0.809717
C	-3.182124	3.256986	0.543072
H	-2.909027	1.247390	1.240357
C	-2.185378	-2.371117	-1.943472

H	0.879007	-2.062398	0.354513
C	-0.305761	-3.382996	-0.831060
H	-4.118498	-1.333664	2.210713
C	-4.918115	0.300880	3.357313
H	-6.723366	1.352826	0.088359
C	-6.391287	1.798473	2.161641
H	-5.895992	1.305078	-1.989766
C	-4.181538	2.205276	-2.897454
C	-2.099299	1.031214	-2.602286
H	-5.825675	-1.253559	-3.338868
C	-7.939169	-1.629686	-3.353259
H	-7.771189	-1.336507	0.505709
C	-9.028939	-1.670922	-1.202032
H	0.245088	1.099116	5.312213
C	-1.020633	-0.598973	4.887414
H	-2.217174	-2.233791	4.145893
C	-2.608070	4.347000	-0.116770
H	-0.844994	5.101980	-1.107822
H	-4.197010	3.314204	0.923098
C	-1.416106	-3.496773	-1.670466
H	0.318931	-4.240943	-0.602013
H	-4.399133	0.008031	4.265540
C	-5.736574	1.430007	3.342749
H	-7.039013	2.671249	2.148315
C	-2.807341	2.108269	-3.123698
H	-4.748329	3.038599	-3.303073
H	-7.982585	-1.708842	-4.436249
C	-9.101478	-1.776023	-2.592420
H	-9.926395	-1.782768	-0.599246
H	-1.197774	-0.833572	5.933177
H	-3.176976	5.263280	-0.249569
H	-1.674054	-4.451145	-2.120540
H	-5.868897	2.020251	4.245200
H	-2.289284	2.866550	-3.703241
H	-10.053493	-1.970233	-3.078395
H	-1.031705	0.944305	-2.781870
H	-3.030717	-2.449278	-2.620590
C	4.017899	0.004483	1.459639
H	3.841041	0.964913	1.941478
H	5.073156	-0.245273	1.440125
C	3.140149	-1.082647	1.823460
C	3.384915	-2.404940	1.586086
C	2.495768	-3.519187	1.900329
C	1.362800	-3.395633	2.735469
H	1.138345	-2.444403	3.206768
C	0.516615	-4.477221	2.962970
H	-0.351891	-4.345456	3.603052
C	0.774181	-5.721555	2.377311
H	0.110572	-6.563089	2.557159
C	1.898489	-5.867319	1.559129
H	2.114455	-6.827263	1.096369
C	2.745545	-4.784779	1.324923
H	3.606345	-4.904989	0.671525
H	2.050182	2.049704	-2.674223
H	2.455416	0.258664	-2.713914
C	3.658386	1.490755	-1.354185
C	2.572615	1.216834	-2.211975
B	3.857641	2.950133	-0.848980
O	3.048687	4.009642	-1.199480
O	4.838860	3.297686	0.052138
C	3.335582	5.084380	-0.256020
C	4.807680	4.748723	0.184665
C	4.466605	0.451138	-0.721933
Pd	2.060923	0.795770	-0.085490
H	5.387201	0.844253	-0.302516
C	4.563350	-0.946167	-1.295183
H	4.358956	-0.911799	-2.372604
H	3.784373	-1.592587	-0.869501
H	2.197665	-0.800850	2.290166
H	4.307804	-2.675182	1.079611
C	5.864701	5.302341	-0.776341
H	5.623381	5.051887	-1.813549
H	5.956779	6.389216	-0.691606
H	6.833158	4.854455	-0.536286

C	5.151673	5.111043	1.624429
H	6.188169	4.832731	1.837488
H	5.049672	6.188948	1.787544
H	4.509345	4.586855	2.334588
C	2.326158	4.952274	0.890143
H	2.455756	5.746194	1.631637
H	1.312021	5.017996	0.489912
H	2.422693	3.982805	1.388538
C	3.161076	6.419187	-0.969406
H	3.445787	7.248461	-0.313433
H	3.760039	6.471387	-1.880501
H	2.111185	6.552896	-1.247558
C	5.941891	-1.598809	-1.065307
H	6.700843	-1.017459	-1.605350
H	6.203395	-1.538452	-0.003062
C	5.984186	-3.044710	-1.510205
C	5.838341	-3.379269	-2.864467
C	6.139491	-4.084653	-0.583893
C	5.836936	-4.711408	-3.278550
C	6.138815	-5.420878	-0.993339
C	5.983970	-5.738874	-2.342812
H	5.726422	-2.586670	-3.600406
H	6.265694	-3.846764	0.468863
H	5.723001	-4.948271	-4.332485
H	6.260559	-6.210187	-0.256595
H	5.982131	-6.776467	-2.664271

TS-γα’-31’_Real

	Value
Charge	0
Electronic Energy, BS1 (a.u.)	-3385.647680
Thermal and entropic correction, BS1 (a.u.)	0.959246
Electronic Energy, BS2 (a.u.)	-3386.626734

Molecular Geometry in Cartesian Coordinates

P	-0.601394	-1.063517	-0.500757
P	-5.156321	1.167679	-0.658011
C	-1.670074	-2.528598	-0.877823
C	-1.007589	-0.748729	1.266090
C	-1.263913	0.272804	-1.620668
C	-5.541499	-0.405287	0.244929
C	-3.834717	1.890135	0.442188
C	-6.611944	2.234923	-0.256849
C	-1.671234	-3.591590	0.043068
C	-2.372177	-2.685800	-2.082475
C	0.040862	-0.438875	2.144924
C	-2.312942	-0.864460	1.770178
C	-2.060789	1.402306	-1.331740
C	-0.839569	0.080208	-2.954686
C	-5.317273	-1.606512	-0.442770
C	-5.983028	-0.463962	1.578512
C	-4.138230	2.428097	1.705536
C	-2.482083	1.841584	0.035857
C	-6.474792	3.634952	-0.258418
C	-7.898713	1.691913	-0.104397
H	-1.131532	-3.496801	0.979853
C	-2.353511	-4.774955	-0.234480
C	-3.043105	-3.878636	-2.364848
H	-2.409092	-1.878691	-2.805174
C	-0.219099	-0.218123	3.499183
H	1.060306	-0.363692	1.783989
C	-2.567139	-0.642669	3.121736
H	-3.131864	-1.126209	1.111878
C	-2.415171	2.267424	-2.386794
H	-0.184917	-0.757471	-3.180184
C	-1.215310	0.934196	-3.984867
H	-4.971651	-1.580479	-1.471410
C	-5.507427	-2.838874	0.189303

H	-6.175590	0.452346	2.126398
C	-6.179848	-1.692171	2.207767
H	-5.173663	2.493548	2.022676
C	-3.140259	2.895215	2.555921
C	-1.486327	2.326442	0.899303
H	-5.491081	4.081744	-0.373538
C	-7.584914	4.462793	-0.092302
H	-8.036178	0.614899	-0.104507
C	-9.009503	2.522343	0.058915
H	-2.344315	-5.581229	0.493699
C	-3.033978	-4.929152	-1.445661
H	-3.580271	-3.979214	-3.303959
C	-1.521314	-0.315506	3.989762
H	0.601440	0.030483	4.166167
H	-3.585197	-0.723502	3.489433
C	-2.014898	2.041855	-3.697439
H	-0.871681	0.744710	-4.997627
H	-5.306343	-3.755645	-0.354030
C	-5.934069	-2.883191	1.515750
H	-6.520839	-1.721956	3.239212
C	-1.804717	2.838888	2.152266
H	-3.403372	3.300573	3.528945
H	-7.453953	5.541557	-0.086547
C	-8.857974	3.909934	0.068377
H	-9.994794	2.080733	0.182559
H	-3.556211	-5.855626	-1.667138
H	-1.723150	-0.140898	5.043150
H	-2.309054	2.732031	-4.482603
H	-6.076914	-3.839498	2.011114
H	-1.015024	3.195844	2.806456
H	-9.722500	4.554730	0.198567
H	-0.452217	2.298749	0.570853
H	-3.012618	3.142646	-2.150488
C	3.526917	-2.121838	-2.225086
H	2.937018	-1.912392	-3.119359
C	4.793285	-1.514460	-2.164902
C	5.114378	-0.382008	-2.878415
H	4.482060	-0.070487	-3.706206
H	1.411601	0.998490	0.123892
H	1.581260	1.291495	-1.648467
C	3.426193	0.867410	-0.573775
C	2.004624	0.804016	-0.768687
B	3.987989	0.305209	0.752216
O	3.306635	0.330163	1.956502
O	5.241010	-0.259148	0.869134
C	4.073217	-0.452523	2.913742
C	5.518892	-0.424888	2.284458
C	4.344435	1.345921	-1.509629
Pd	1.662747	-1.325831	-0.980779
H	5.364273	1.442880	-1.150778
C	3.965042	2.308749	-2.611481
H	4.806226	2.427594	-3.304254
H	3.121620	1.926596	-3.199788
H	5.486077	-1.824763	-1.386023
H	6.128952	0.001001	-2.884511
C	3.079671	-3.249997	-1.424902
C	1.793940	-3.812281	-1.674862
C	3.821034	-3.784700	-0.328793
C	1.294257	-4.860577	-0.877644
C	3.309566	-4.807125	0.448682
C	2.035972	-5.352720	0.185169
H	1.248099	-3.526513	-2.570306
H	4.799115	-3.373846	-0.101165
H	0.320608	-5.279395	-1.109485
H	3.900415	-5.193701	1.275727
H	1.649492	-6.159473	0.801818
C	6.343256	0.795885	2.709426
H	7.242800	0.847851	2.089312
H	5.781321	1.721507	2.554709
H	6.648506	0.738529	3.758522
C	6.325636	-1.704983	2.472173
H	5.814158	-2.567350	2.040012
H	7.296070	-1.604005	1.976616
H	6.505214	-1.900990	3.534433

C	3.961573	0.209117	4.283398
H	4.596269	-0.303201	5.013965
H	4.248345	1.261927	4.247836
H	2.928066	0.153267	4.638989
C	3.450093	-1.852031	2.945372
H	3.473517	-2.316898	1.956977
H	3.963141	-2.506255	3.656828
H	2.402922	-1.773518	3.249746
C	3.574710	3.707755	-2.051988
H	2.640963	3.606984	-1.488892
H	3.374507	4.381368	-2.894878
C	4.643947	4.298621	-1.159388
C	5.756915	4.952003	-1.707192
C	4.584684	4.143158	0.233972
C	6.784119	5.431859	-0.891049
C	5.608915	4.621557	1.054427
C	6.714926	5.265724	0.494324
H	5.819094	5.082568	-2.785524
H	3.733452	3.630374	0.673497
H	7.637829	5.936192	-1.336652
H	5.542377	4.491086	2.131617
H	7.513616	5.637622	1.131012

3-I-Pd_Real

	Value
Charge	0
Electronic Energy, BS1 (a.u.)	-3385.708903
Thermal and entropic correction, BS1 (a.u.)	0.964375
Electronic Energy, BS2 (a.u.)	-3386.684310

Molecular Geometry in Cartesian Coordinates

P	-0.539277	-0.600945	-0.406914
P	-5.414883	1.020916	-0.734569
C	-1.330764	-2.246434	-0.735747
C	-1.083246	-0.311558	1.332762
C	-1.394607	0.529148	-1.617535
C	-5.639095	-0.540174	0.241269
C	-4.179509	1.925896	0.332395
C	-6.973742	1.952135	-0.386642
C	-1.002705	-3.290943	0.146354
C	-2.158831	-2.528657	-1.830647
C	-0.133800	0.159822	2.251440
C	-2.378899	-0.607538	1.782801
C	-2.345079	1.552632	-1.410352
C	-0.940803	0.296299	-2.935467
C	-5.219494	-1.733063	-0.364987
C	-6.132460	-0.590407	1.556586
C	-4.545164	2.469221	1.576712
C	-2.827447	2.011412	-0.070855
C	-6.977669	3.358290	-0.425710
C	-8.202945	1.287101	-0.241027
H	-0.344049	-3.099362	0.988905
C	-1.505153	-4.574602	-0.049996
C	-2.648345	-3.822520	-2.036599
H	-2.436671	-1.743461	-2.524668
C	-0.483141	0.362939	3.588373
H	0.882085	0.358515	1.929482
C	-2.727279	-0.395637	3.115464
H	-3.118761	-0.999174	1.096030
C	-2.810553	2.280345	-2.524155
H	-0.188504	-0.469886	-3.101707
C	-1.425401	1.012455	-4.023800
H	-4.833037	-1.712237	-1.378931
C	-5.261008	-2.944312	0.331491
H	-6.475709	0.317466	2.041400
C	-6.189730	-1.800182	2.247185
H	-5.581795	2.427629	1.893508
C	-3.608955	3.072902	2.411096

C	-1.895212	2.630920	0.777587
H	-6.041872	3.898905	-0.536836
C	-8.168222	4.074397	-0.301804
H	-8.232210	0.202056	-0.213670
C	-9.393989	2.005479	-0.118861
H	-1.233801	-5.367318	0.640720
C	-2.329081	-4.847671	-1.146027
H	-3.290051	-4.020940	-2.891054
C	-1.781513	0.093133	4.021456
H	0.263380	0.732287	4.286509
H	-3.740141	-0.613208	3.439003
C	-2.372365	2.017527	-3.815803
H	-1.056480	0.795824	-5.022249
H	-4.898852	-3.849194	-0.145614
C	-5.743931	-2.978867	1.638892
H	-6.573942	-1.823787	3.263520
C	-2.273557	3.149769	2.011014
H	-3.919281	3.477894	3.370211
H	-8.146003	5.160663	-0.324606
C	-9.382291	3.401079	-0.145916
H	-10.332234	1.470418	0.001244
H	-2.711023	-5.852056	-1.306632
H	-2.056687	0.256377	5.060022
H	-2.755209	2.599329	-4.649108
H	-5.774062	-3.917673	2.184746
H	-1.530844	3.611125	2.654833
H	-10.309254	3.958886	-0.047580
H	-0.860572	2.695578	0.454911
H	-3.527703	3.077136	-2.351653
C	2.513953	-2.085583	-2.153771
H	1.927181	-1.875959	-3.048130
C	3.535598	-1.207898	-1.821913
C	3.916955	0.030823	-2.610652
H	3.084662	0.322205	-3.265137
H	1.663325	2.004616	0.661188
H	1.699967	2.377027	-1.132982
C	3.348081	1.161247	-0.390579
C	2.102638	1.790567	-0.308793
B	3.899628	0.500046	0.898515
O	3.291740	0.589803	2.140292
O	5.034883	-0.285099	0.927349
C	3.881854	-0.443226	2.978703
C	5.296177	-0.635147	2.314097
C	4.253407	1.197664	-1.647759
Pd	1.761121	-0.390408	-0.729030
H	5.274601	1.031978	-1.285451
C	4.238474	2.541643	-2.392802
H	4.901855	2.459262	-3.264136
H	3.232545	2.731071	-2.789825
H	4.279201	-1.520059	-1.091671
H	4.777212	-0.175881	-3.267339
C	2.343098	-3.448759	-1.609590
C	1.442466	-4.330207	-2.231593
C	3.050659	-3.919071	-0.485236
C	1.257991	-5.629134	-1.758428
C	2.862823	-5.213380	-0.009343
C	1.966295	-6.080598	-0.643990
H	0.871799	-3.985439	-3.090035
H	3.749682	-3.261182	0.020900
H	0.549128	-6.284104	-2.257067
H	3.420814	-5.549500	0.861087
H	1.821897	-7.090724	-0.271255
C	6.343222	0.355704	2.831908
H	7.217161	0.322654	2.176430
H	5.952671	1.376518	2.821307
H	6.660056	0.112697	3.850599
C	5.843529	-2.057177	2.358231
H	5.176426	-2.761122	1.857187
H	6.813524	-2.094006	1.853325
H	5.985569	-2.386803	3.392675
C	3.909210	0.055369	4.419032
H	4.424812	-0.661006	5.066987
H	4.405645	1.023884	4.502844
H	2.885489	0.167813	4.789139

C	2.988849	-1.684934	2.864647
H	2.915770	-2.027946	1.829098
H	3.363440	-2.505869	3.483378
H	1.979670	-1.435776	3.200848
C	4.673418	3.757154	-1.546934
H	3.968671	3.887724	-0.719310
H	4.600455	4.656775	-2.170678
C	6.079256	3.634842	-1.000765
C	7.188205	3.836722	-1.836070
C	6.310247	3.281766	0.335604
C	8.488754	3.692918	-1.351776
C	7.610315	3.140269	0.826578
C	8.704572	3.344756	-0.015632
H	7.026904	4.112561	-2.875922
H	5.461040	3.115487	0.993852
H	9.333979	3.857014	-2.015039
H	7.767070	2.869581	1.866667
H	9.716418	3.236087	0.364938

Allene_1^{M}

	Value
Charge	0
Electronic Energy, BS1 (a.u.)	-155.988274
Thermal and entropic correction, BS1 (a.u.)	0.057154
Electronic Energy, BS2 (a.u.)	-156.047271

Molecular Geometry in Cartesian Coordinates

C	1.942242	-0.213901	0.000003
H	2.487370	-0.383381	-0.927430
H	2.487205	-0.383616	0.927497
C	0.693749	0.175742	-0.000025
C	-0.557210	0.561088	0.000012
H	-0.768967	1.631765	0.000108
C	-1.747692	-0.367116	-0.000016
H	-1.439345	-1.415901	-0.000484
H	-2.376154	-0.192198	0.882146
H	-2.376642	-0.191547	-0.881677

B2ed2

	Value
Charge	0
Electronic Energy, BS1 (a.u.)	-508.026547
Thermal and entropic correction, BS1 (a.u.)	0.103697
Electronic Energy, BS2 (a.u.)	-508.233801

Molecular Geometry in Cartesian Coordinates

B	0.851821	0.000152	0.000048
O	1.607605	1.108540	0.296226
O	1.607301	-1.108426	-0.296179
C	2.997637	0.723035	0.271874
C	2.997440	-0.723238	-0.271976
H	3.399692	0.788480	1.289376
H	3.552958	1.417651	-0.366082
H	3.552635	-1.418026	0.365898
H	3.399370	-0.788762	-1.289525
B	-0.851828	0.000184	0.000009
O	-1.607604	1.108601	-0.296014
O	-1.607330	-1.108442	0.296083
C	-2.997622	0.722942	-0.272138
C	-2.997437	-0.723158	0.272111
H	-3.399229	0.788032	-1.289845

H	-3.553297	1.417672	0.365373
H	-3.552868	-1.418074	-0.365413
H	-3.399126	-0.788320	1.289785

Bed-OMe

	Value
Charge	0
Electronic Energy, BS1 (a.u.)	-369.197185
Thermal and entropic correction, BS1 (a.u.)	0.080898
Electronic Energy, BS2 (a.u.)	-369.350304

Molecular Geometry in Cartesian Coordinates

B	0.260694	-0.291272	-0.010062
O	-0.766376	-1.206971	-0.075247
O	-0.159402	1.022349	0.064714
C	-1.988043	-0.472754	0.100217
C	-1.588872	1.009639	-0.087995
H	-2.381903	-0.666135	1.106091
H	-2.729044	-0.807496	-0.632271
H	-2.045580	1.670150	0.655388
H	-1.844519	1.380958	-1.088245
O	1.558617	-0.679686	-0.022645
C	2.583664	0.312862	0.018685
H	2.442766	1.073181	-0.758189
H	2.612243	0.813526	0.994015
H	3.539374	-0.191842	-0.146500

CO2

	Value
Charge	0
Electronic Energy, BS1 (a.u.)	-188.577848
Thermal and entropic correction, BS1 (a.u.)	-0.009134
Electronic Energy, BS2 (a.u.)	-188.669893

Molecular Geometry in Cartesian Coordinates

C	0.000000	0.000000	0.000000
O	0.000000	0.000000	1.169451
O	-0.000000	-0.000000	-1.169451

Cu_BiPhep_MeO

	Value
Charge	0
Electronic Energy, BS1 (a.u.)	-2384.078129
Thermal and entropic correction, BS1 (a.u.)	0.497426
Electronic Energy, BS2 (a.u.)	-3827.933942

Molecular Geometry in Cartesian Coordinates

Cu	0.123614	-0.845266	-1.479572
P	-1.713970	-0.086334	-0.277658
P	1.809992	0.042291	-0.334819
C	-3.153140	0.466355	-1.274941
C	-2.366574	-1.452305	0.759864
C	-1.401015	1.299689	0.896780
C	1.973421	1.865023	-0.229231
C	1.463930	-0.555069	1.383280

C	3.516158	-0.550743	-0.701052
C	-2.896748	0.921793	-2.576794
C	-4.474008	0.448729	-0.800964
C	-2.198138	-2.756225	0.263766
C	-3.002135	-1.253179	1.994249
C	-0.398220	1.188717	1.892531
C	-2.093893	2.514560	0.763901
C	1.517275	2.617286	-1.321065
C	2.540904	2.525984	0.870583
C	2.172574	-1.682513	1.834311
C	0.402537	-0.050629	2.180182
C	3.627391	-1.681150	-1.525143
C	4.682171	0.050618	-0.202536
H	-1.878182	0.905806	-2.956319
C	-3.941218	1.370900	-3.386721
C	-5.517075	0.892006	-1.614002
H	-4.686429	0.082108	0.198807
C	-2.671814	-3.843953	0.999715
H	-1.692020	-2.904082	-0.691359
C	-3.467599	-2.346320	2.726177
H	-3.121831	-0.247588	2.387163
C	-0.171106	2.282844	2.741872
H	-2.859174	2.611876	0.001885
C	-1.826678	3.600297	1.596091
H	1.059123	2.108209	-2.165529
C	1.638807	4.007753	-1.320558
H	2.865240	1.955651	1.736054
C	2.662589	3.914991	0.869334
H	2.983198	-2.078214	1.232149
C	1.858631	-2.311396	3.036961
C	0.116123	-0.693243	3.396514
H	2.726439	-2.139202	-1.923045
C	4.881584	-2.211474	-1.833909
H	4.617957	0.934872	0.422760
C	5.934196	-0.476008	-0.519107
H	-3.732570	1.717942	-4.394678
C	-5.251747	1.357321	-2.905357
H	-6.537489	0.871769	-1.241485
C	-3.302868	-3.642143	2.229597
H	-2.539871	-4.851153	0.614107
H	-3.956060	-2.186970	3.683742
C	-0.865901	3.481839	2.598183
H	-2.376638	4.527901	1.465521
H	1.279874	4.581828	-2.169981
C	2.213608	4.657114	-0.226452
H	3.098594	4.419225	1.727174
C	0.820981	-1.815419	3.823746
H	2.424469	-3.183064	3.352732
H	4.953678	-3.087799	-2.471728
C	6.036213	-1.609072	-1.331720
H	6.831471	-0.000795	-0.132420
H	-6.066773	1.699059	-3.537263
H	-3.662769	-4.492433	2.802531
H	-0.650756	4.315760	3.259906
H	2.304397	5.739637	-0.222792
H	0.558743	-2.296071	4.761627
H	7.013490	-2.015655	-1.576534
H	-0.692662	-0.302161	4.005918
H	0.584840	2.187602	3.515758
H	-0.031574	-3.831521	-3.870362
C	0.482498	-3.120751	-3.192761
H	1.034428	-3.750202	-2.462185
H	1.253539	-2.619134	-3.813323
O	-0.422515	-2.246651	-2.602005

Allene_1

	Value
Charge	0
Electronic Energy, BS1 (a.u.)	-426.376060

	Value
Thermal and entropic correction, BS1 (a.u.)	0.157183
Electronic Energy, BS2 (a.u.)	-426.527743

Molecular Geometry in Cartesian Coordinates

H	4.774411	1.405856	0.693413
H	5.212397	0.729823	-0.977563
C	3.221285	0.488585	-0.371974
C	4.455379	0.890953	-0.211530
C	1.989810	0.076766	-0.535097
H	1.266260	0.753047	-0.991501
C	1.474717	-1.279715	-0.106643
H	1.061260	-1.797900	-0.982574
H	2.303406	-1.888250	0.269837
C	0.372542	-1.192571	0.979196
H	0.786520	-0.692304	1.861785
H	0.111654	-2.214085	1.281527
C	-0.871143	-0.469759	0.509740
C	-1.831476	-1.136772	-0.265303
C	-1.072289	0.888073	0.794744
C	-2.960552	-0.468649	-0.740622
C	-2.200391	1.561480	0.320545
C	-3.148620	0.884947	-0.449257
H	-1.692419	-2.191281	-0.493517
H	-0.336580	1.419991	1.392880
H	-3.695478	-1.004766	-1.335166
H	-2.338761	2.613639	0.554550
H	-4.028253	1.406206	-0.816458

B2pin2

	Value
Charge	0
Electronic Energy, BS1 (a.u.)	-822.624345
Thermal and entropic correction, BS1 (a.u.)	0.317062
Electronic Energy, BS2 (a.u.)	-822.924293

Molecular Geometry in Cartesian Coordinates

B	-0.851305	0.000024	-0.000033
O	-1.612547	-0.614633	-0.966167
O	-1.612519	0.614668	0.966124
C	-2.999368	-0.598767	-0.508499
C	-2.999372	0.598766	0.508503
B	0.851302	0.000026	-0.000035
O	1.612550	0.614605	-0.966208
O	1.612505	-0.614581	0.966162
C	2.999391	0.598733	-0.508516
C	2.999354	-0.598738	0.508526
C	3.908559	0.420927	-1.719331
H	3.827502	1.294364	-2.373232
H	4.953894	0.326790	-1.406904
H	3.636762	-0.460851	-2.302334
C	3.253754	-1.956660	-0.154789
H	4.294810	-2.060887	-0.474771
H	3.032256	-2.750525	0.564128
H	2.604596	-2.098090	-1.023686
C	3.908487	-0.420957	1.719374
H	3.827368	-1.294386	2.373279
H	4.953836	-0.326869	1.406979
H	3.636710	0.460837	2.302362
C	3.253786	1.956652	0.154804
H	2.604625	2.098083	1.023698
H	4.294841	2.060877	0.474791
H	3.032292	2.750518	-0.564114
C	-3.908536	-0.421026	-1.719330
H	-4.953872	-0.326870	-1.406913

H	-3.636734	0.460719	-2.302380
H	-3.827478	-1.294500	-2.373182
C	-3.253751	-1.956663	0.154876
H	-3.032277	-2.750555	-0.564020
H	-2.604564	-2.098073	1.023756
H	-4.294795	-2.060876	0.474897
C	-3.253808	1.956651	-0.154871
H	-4.294876	2.060856	-0.474821
H	-3.032291	2.750557	0.563997
H	-2.604683	2.098052	-1.023798
C	-3.908483	0.421004	1.719370
H	-3.636658	-0.460746	2.302401
H	-3.827402	1.294470	2.373231
H	-4.953831	0.326851	1.406993

Pd-0-BIPHEP

	Value
Charge	0
Electronic Energy, BS1 (a.u.)	-2199.511967
Thermal and entropic correction, BS1 (a.u.)	0.460896
Electronic Energy, BS2 (a.u.)	-2200.091165

Molecular Geometry in Cartesian Coordinates

Pd	-0.000023	-0.000105	-1.719550
P	-1.942892	-0.115568	-0.535929
P	1.942901	0.115626	-0.536025
C	-3.553968	0.591392	-1.113061
C	-2.405424	-1.708122	0.267568
C	-1.488422	0.961899	0.916289
C	2.405448	1.708176	0.267436
C	1.488449	-0.961792	0.916274
C	3.553938	-0.591409	-1.113128
C	-3.478334	1.570057	-2.119135
C	-4.817451	0.210368	-0.636985
C	-2.033193	-2.903421	-0.361927
C	-3.081254	-1.766045	1.497288
C	-0.384253	0.649691	1.755310
C	-2.161598	2.178049	1.114382
C	2.033282	2.903448	-0.362158
C	3.081220	1.766158	1.497179
C	2.161622	-2.177935	1.114422
C	0.384300	-0.649538	1.755314
C	3.478292	-1.570081	-2.119191
C	4.817418	-0.210473	-0.636970
H	-2.503470	1.854914	-2.509038
C	-4.633381	2.171672	-2.618690
C	-5.974605	0.800202	-1.150469
H	-4.905913	-0.552983	0.128382
C	-2.339026	-4.135321	0.220210
H	-1.483999	-2.861315	-1.298880
C	-3.390480	-2.996632	2.076377
H	-3.348007	-0.846550	2.010701
C	-0.019817	1.566277	2.752203
H	-3.010144	2.426653	0.486699
C	-1.764880	3.084758	2.097643
H	1.484168	2.861308	-1.299154
C	2.339108	4.135381	0.219927
H	3.347944	0.846685	2.010646
C	3.390441	2.996771	2.076208
H	3.010178	-2.426550	0.486759
C	1.764921	-3.084601	2.097727
C	0.019883	-1.566085	2.752252
H	2.503434	-1.854860	-2.509171
C	4.633330	-2.171824	-2.618621
H	4.905879	0.552919	0.128359
C	5.974561	-0.800439	-1.150323
H	-4.555686	2.930965	-3.392001
C	-5.886335	1.785153	-2.136294

H	-6.946606	0.489187	-0.777127
C	-3.018861	-4.183575	1.438478
H	-2.038666	-5.055192	-0.273687
H	-3.912117	-3.029638	3.029043
C	-0.684654	2.780185	2.921110
H	-2.304931	4.019974	2.217172
H	2.038781	5.055220	-0.274049
C	3.018873	4.183689	1.438224
H	3.912059	3.029833	3.028883
C	0.684707	-2.779993	2.921197
H	2.304975	-4.019810	2.217300
H	4.555629	-2.931116	-3.391933
C	5.886276	-1.785428	-2.136115
H	6.946562	-0.489516	-0.776905
H	-6.788533	2.242828	-2.532436
H	-3.252236	-5.141773	1.894401
H	-0.359126	3.474085	3.690579
H	3.252234	5.141897	1.894134
H	0.359183	-3.473873	3.690686
H	6.788463	-2.243223	-2.532140
H	-0.818460	-1.319598	3.395857
H	0.818532	1.319818	3.395812

3-l

	Value
Charge	0
Electronic Energy, BS1 (a.u.)	-1186.162410
Thermal and entropic correction, BS1 (a.u.)	0.476684
Electronic Energy, BS2 (a.u.)	-1186.573199

Molecular Geometry in Cartesian Coordinates

C	2.110893	-2.334926	-0.102404
H	2.205293	-2.962431	0.784281
C	0.899777	-2.214485	-0.666982
C	-0.348990	-2.868692	-0.154810
H	-0.110840	-3.545836	0.675079
H	-0.401225	-0.658637	3.476029
H	-1.350118	-2.194250	3.095805
C	-0.827507	-0.961193	1.424515
C	-0.865872	-1.292407	2.724724
B	-0.105998	0.356983	1.008548
O	0.472441	1.227346	1.901923
O	-0.004102	0.776601	-0.295957
C	1.234127	2.199239	1.126850
C	0.525833	2.133178	-0.278865
C	-1.414442	-1.839202	0.322279
H	-1.632205	-1.186055	-0.531039
C	-2.720113	-2.564803	0.694866
H	-3.047498	-3.142159	-0.179536
H	-2.532749	-3.297418	1.490567
H	0.761328	-1.546407	-1.514811
H	-0.798994	-3.476859	-0.953377
C	3.343554	-1.648472	-0.518020
C	4.441703	-1.624377	0.361441
C	3.470440	-0.971318	-1.747240
C	5.609102	-0.930892	0.042793
C	4.636285	-0.278690	-2.065963
C	5.711039	-0.249402	-1.171318
H	4.366391	-2.142814	1.314191
H	2.651732	-0.990585	-2.459770
H	6.438434	-0.921386	0.744836
H	4.708959	0.237610	-3.019438
H	6.618513	0.292011	-1.422839
C	-0.680288	3.070559	-0.395241
H	-1.241742	2.812995	-1.296860
H	-1.349758	2.956433	0.461940
H	-0.372493	4.118271	-0.461953

C	1.452704	2.306777	-1.476349
H	2.235219	1.546738	-1.492444
H	0.876114	2.220710	-2.402596
H	1.923255	3.295371	-1.460510
C	1.149942	3.550484	1.827729
H	1.645638	4.325301	1.233742
H	0.115524	3.852169	2.003181
H	1.653133	3.493810	2.797632
C	2.681615	1.699968	1.096967
H	2.755734	0.730277	0.601442
H	3.342270	2.403777	0.582428
H	3.037586	1.580975	2.124360
C	-3.871270	-1.635233	1.136227
H	-3.593736	-1.136540	2.069829
H	-4.749648	-2.254800	1.355428
C	-4.230973	-0.596230	0.096341
C	-4.993414	-0.945450	-1.028160
C	-3.778044	0.725569	0.207669
C	-5.297708	-0.003012	-2.011212
C	-4.083636	1.673762	-0.771268
C	-4.843691	1.312612	-1.885191
H	-5.354311	-1.966706	-1.129415
H	-3.180185	1.009922	1.069821
H	-5.893128	-0.293276	-2.872884
H	-3.726723	2.693828	-0.662690
H	-5.082902	2.049261	-2.647148

3-(R,R)

	Value
Charge	0
Electronic Energy, BS1 (a.u.)	-1186.145031
Thermal and entropic correction, BS1 (a.u.)	0.475286
Electronic Energy, BS2 (a.u.)	-1186.556893

Molecular Geometry in Cartesian Coordinates

C	-1.826056	0.153214	1.096186
H	-2.016120	-0.631540	1.838592
C	-1.082934	1.267505	1.797139
C	-0.562976	1.174706	3.021296
H	-0.655654	0.267698	3.615572
H	-0.475964	1.567841	-2.787945
H	-1.839759	0.362326	-2.498347
C	-0.336749	0.478012	-0.977196
C	-0.917130	0.813247	-2.140606
B	1.018867	1.147252	-0.600702
O	1.405761	2.399160	-1.015166
O	1.965316	0.517921	0.172535
C	2.612193	2.741168	-0.269594
C	3.176622	1.324458	0.120061
C	-0.917922	-0.537077	0.000034
H	-0.077837	-0.941383	0.574790
C	-1.646696	-1.720785	-0.658023
H	-2.090290	-2.341281	0.131310
H	-2.484282	-1.359324	-1.263730
H	-0.957114	2.195847	1.242154
H	-0.024294	2.001397	3.477303
C	-3.179151	0.603149	0.560540
C	-4.311124	-0.194598	0.785354
C	-3.342658	1.786177	-0.178184
C	-5.563441	0.166571	0.286220
C	-4.594080	2.151572	-0.678206
C	-5.709756	1.343786	-0.450079
H	-4.207367	-1.111319	1.360596
H	-2.488407	2.423904	-0.374709
H	-6.423910	-0.469291	0.476691
H	-4.695722	3.071904	-1.247192
H	-6.683139	1.630836	-0.838118
C	4.066969	0.711316	-0.965506

H	4.257696	-0.337578	-0.721465
H	3.578027	0.747553	-1.943363
H	5.029111	1.226991	-1.036605
C	3.862679	1.249767	1.479185
H	3.181641	1.523391	2.286898
H	4.204879	0.226709	1.661910
H	4.734375	1.911497	1.511418
C	3.518560	3.576841	-1.165150
H	4.469959	3.785154	-0.664939
H	3.723503	3.074855	-2.112540
H	3.036254	4.533234	-1.388170
C	2.154502	3.557267	0.943999
H	1.508718	2.960881	1.594587
H	3.003789	3.919681	1.530509
H	1.581635	4.421285	0.595442
C	-0.738190	-2.611825	-1.535855
H	-0.263438	-1.989200	-2.301721
H	-1.372095	-3.334484	-2.063084
C	0.319128	-3.355044	-0.747815
C	0.049794	-4.622466	-0.211128
C	1.572418	-2.778345	-0.493403
C	1.001795	-5.296651	0.555890
C	2.525138	-3.446620	0.277801
C	2.244999	-4.709629	0.803810
H	-0.916443	-5.085551	-0.399838
H	1.795354	-1.789053	-0.880408
H	0.773951	-6.280435	0.957912
H	3.486880	-2.977742	0.469538
H	2.987496	-5.232003	1.400895

3-(R,S)

	Value
Charge	0
Electronic Energy, BS1 (a.u.)	-1186.144855
Thermal and entropic correction, BS1 (a.u.)	0.476748
Electronic Energy, BS2 (a.u.)	-1186.560770

Molecular Geometry in Cartesian Coordinates

C	1.794865	0.201512	1.070418
H	1.253961	-0.679807	1.432034
C	1.952759	1.133186	2.250889
C	1.227927	1.039831	3.366249
H	0.482189	0.257812	3.496345
H	0.634233	-1.301748	-2.810512
H	1.807202	0.099282	-2.555081
C	0.389539	-0.249936	-0.989853
C	0.974359	-0.487999	-2.174495
B	-0.774792	-1.167666	-0.518082
O	-1.414400	-2.090513	-1.311498
O	-1.256823	-1.137278	0.769851
C	-2.237950	-2.911055	-0.431014
C	-2.461604	-1.955466	0.799338
C	0.838654	0.833802	-0.020558
H	-0.044416	1.129724	0.555263
C	1.396155	2.106666	-0.674970
H	1.668737	2.810133	0.120995
H	2.317527	1.892190	-1.226832
H	2.680764	1.937672	2.163192
H	1.345541	1.744217	4.185684
C	3.118296	-0.279154	0.494141
C	4.199140	0.585134	0.258912
C	3.269269	-1.628612	0.141990
C	5.385546	0.117049	-0.309379
C	4.453183	-2.102384	-0.425007
C	5.518254	-1.229354	-0.654312
H	4.119081	1.636359	0.516993
H	2.444077	-2.314896	0.312609
H	6.207738	0.806856	-0.480576

H	4.542659	-3.153308	-0.686892
H	6.442109	-1.594129	-1.094567
C	-3.642725	-0.997990	0.612387
H	-3.595702	-0.221261	1.380222
H	-3.596391	-0.505038	-0.361855
H	-4.601172	-1.518462	0.698845
C	-2.544753	-2.649067	2.153731
H	-1.632177	-3.204236	2.378854
H	-2.687584	-1.902833	2.941044
H	-3.393107	-3.340540	2.183531
C	-3.504851	-3.308573	-1.178958
H	-4.186039	-3.857493	-0.520573
H	-4.028300	-2.436780	-1.575635
H	-3.248706	-3.959202	-2.020453
C	-1.407089	-4.151608	-0.086239
H	-0.496784	-3.877661	0.455434
H	-1.974373	-4.861931	0.522325
H	-1.110966	-4.649966	-1.013582
C	0.397546	2.805358	-1.627016
H	0.254625	2.189116	-2.519332
H	0.845203	3.749487	-1.961590
C	-0.946463	3.075709	-0.985992
C	-1.087936	4.080000	-0.017152
C	-2.066977	2.295517	-1.303429
C	-2.311395	4.299330	0.616675
C	-3.295334	2.514608	-0.675023
C	-3.421526	3.515960	0.289403
H	-0.228096	4.693522	0.242597
H	-1.970375	1.506085	-2.044517
H	-2.399979	5.083301	1.364184
H	-4.153351	1.902477	-0.938998
H	-4.375730	3.686936	0.780024

Allylic_Carbonate_2

	Value
Charge	0
Electronic Energy, BS1 (a.u.)	-770.071845
Thermal and entropic correction, BS1 (a.u.)	0.248150
Electronic Energy, BS2 (a.u.)	-770.361401

Molecular Geometry in Cartesian Coordinates

C	-4.057642	-0.666818	0.280904
O	-3.026934	0.332441	0.633320
C	-2.065734	0.651878	-0.233959
O	-1.907950	0.225763	-1.363322
O	-1.273907	1.557071	0.365887
C	-0.141824	2.019092	-0.427178
H	-0.500182	2.251477	-1.435582
H	0.172836	2.941457	0.065831
C	0.959503	1.007748	-0.465911
C	2.171608	1.226951	0.064820
C	3.326771	0.317165	0.059199
C	3.312199	-0.942898	-0.570077
H	2.419631	-1.278821	-1.088772
C	4.432966	-1.767657	-0.539358
H	4.400453	-2.735746	-1.031571
C	5.597759	-1.356290	0.117410
H	6.470758	-2.002381	0.137742
C	5.629682	-0.109563	0.743798
H	6.528701	0.221152	1.256414
C	4.506318	0.716406	0.713147
H	4.537427	1.686216	1.203930
H	2.346134	2.182355	0.561358
H	0.714675	0.074979	-0.966824
C	-3.395796	-2.019890	0.013956
H	-4.168243	-2.785132	-0.112252
H	-2.783667	-1.989944	-0.888090

H	-2.765217	-2.307081	0.861374
C	-4.876366	-0.168834	-0.911325
H	-5.277197	0.828851	-0.705893
H	-4.270335	-0.126695	-1.816950
H	-5.718497	-0.846604	-1.083980
C	-4.910000	-0.715406	1.548458
H	-5.338341	0.267982	1.763780
H	-5.727893	-1.430837	1.421539
H	-4.307392	-1.024734	2.407563

Allylic_Carbonate_2[M]

	Value
Charge	0
Electronic Energy, BS1 (a.u.)	-652.0995828
Thermal and entropic correction, BS1 (a.u.)	0.168620
Electronic Energy, BS2 (a.u.)	-652.354878

Molecular Geometry in Cartesian Coordinates

H	-5.541942	1.407408	-1.551464
C	-4.877456	1.361390	-0.687941
H	-4.273130	2.270817	-0.626506
H	-5.459096	1.253907	0.231904
O	-4.044739	0.207863	-0.905164
C	-3.145527	-0.022521	0.060485
O	-2.996418	0.645096	1.064833
O	-2.450140	-1.114797	-0.278072
C	-1.409433	-1.509086	0.665939
H	-1.831123	-1.467748	1.675816
H	-1.193068	-2.547530	0.406667
C	-0.195801	-0.643278	0.550817
C	0.998232	-1.111080	0.157543
C	2.256303	-0.358929	0.041321
C	2.403289	0.968913	0.487412
H	1.568345	1.477464	0.959386
C	3.614589	1.639175	0.341769
H	3.707059	2.662861	0.693945
C	4.711167	1.000907	-0.247378
H	5.655340	1.526666	-0.356985
C	4.584390	-0.317482	-0.687544
H	5.429971	-0.824562	-1.143822
C	3.370776	-0.988771	-0.541611
H	3.278400	-2.015349	-0.887890
H	1.068302	-2.164697	-0.116158
H	-0.341858	0.400039	0.817447

3-(R,R)[M]

	Value
Charge	0
Electronic Energy, BS1 (a.u.)	-758.453674
Thermal and entropic correction, BS1 (a.u.)	0.265301
Electronic Energy, BS2 (a.u.)	-758.729072

Molecular Geometry in Cartesian Coordinates

C	0.139807	2.119831	2.300539
H	0.084310	3.062627	2.838720
H	0.883029	1.404883	2.648002

C	-0.659756	1.852817	1.267617
C	-0.595847	0.585016	0.456209
C	-1.933941	-0.120671	0.261448
C	-3.170607	0.537644	0.265949
H	-3.220659	1.605161	0.453886
C	-4.357328	-0.165373	0.037121
H	-5.305338	0.365995	0.047113
C	-4.328933	-1.539625	-0.200339
H	-5.252069	-2.084983	-0.375840
C	-3.101635	-2.208599	-0.207566
H	-3.065628	-3.279760	-0.388086
C	-1.920357	-1.504654	0.020968
H	-0.964951	-2.024528	0.009661
H	3.294217	2.290742	-0.735307
H	1.718404	3.106786	-1.234592
C	1.595833	1.027777	-0.740668
C	2.225322	2.200372	-0.912572
B	2.428369	-0.209989	-0.290759
O	3.775010	-0.177752	-0.016066
O	1.871965	-1.461261	-0.130188
C	4.161424	-1.478979	0.460984
C	2.932680	-2.379143	0.194467
C	0.102065	0.826028	-0.944565
H	-0.017922	-0.122940	-1.482749
C	-0.591835	1.916813	-1.764570
H	-1.650122	1.676243	-1.904881
H	-0.534940	2.897214	-1.281520
H	-1.374861	2.610690	0.953422
H	0.049824	-0.117479	0.993278
H	2.644531	-2.974754	1.065873
H	4.400895	-1.408281	1.528665
H	5.055518	-1.813252	-0.074103
H	3.087166	-3.055087	-0.654626
H	-0.130397	2.007239	-2.753205

η^1-VIPd_Real

	Value
Charge	0
Electronic Energy, BS1 (a.u.)	-3385.653182
Thermal and entropic correction, BS1 (a.u.)	0.955897
Electronic Energy, BS2 (a.u.)	-3386.635729

Molecular Geometry in Cartesian Coordinates

P	0.411548.	0.456697	0.480996
P	4.814746.	-1.076202	-0.288640
C	1.039012.	0.265369	-1.231004
C	1.037377.	2.075802	1.090571
C	1.226233.	-0.900965	1.443199
C	5.050577.	-0.150212	-1.869656
C	4.582814.	0.269260	0.975915
C	6.546887.	-1.602875	0.118937
C	0.740610.	1.266122	-2.173996
C	1.628524.	-0.927764	-1.669121
C	0.466704.	2.559723	2.280380
C	1.955093.	2.879960	0.402898
C	2.550378.	-0.946795	1.941237
C	0.400845.	-2.030908	1.606853
C	5.534610.	-0.873028	-2.976223
C	4.640918.	1.175505	-2.069573

C	5.501182.	1.330041	1.081041
C	3.523745.	0.193765	1.904973
C	6.684026.	-2.668968	1.023201
C	7.707764.	-1.007712	-0.400019
H	0.243513.	2.178287	-1.856456
C	1.072855.	1.091877	-3.515652
C	1.949315.	-1.102035	-3.018065
H	1.848268.	-1.719177	-0.962071
C	0.826710.	3.809113	2.783794
H	-0.264030.	1.957215	2.815764
C	2.303650.	4.136385	0.899780
H	2.397810.	2.527521	-0.520422
C	2.987029.	-2.117556	2.583421
H	-0.614688.	-2.000544	1.225540
C	0.861482.	-3.192917	2.223357
H	5.851213.	-1.905548	-2.849702
C	5.619998.	-0.282005	-4.235533
H	4.267712.	1.756225	-1.234811
C	4.712134.	1.762949	-3.335000
H	6.324121.	1.389626	0.375471
C	5.383781.	2.296338	2.074988
C	3.434448.	1.159013	2.922322
H	5.794994.	-3.155082	1.417788
C	7.946712.	-3.114875	1.416580
H	7.626015.	-0.195484	-1.115691
C	8.970830.	-1.460893	-0.015107
H	0.851070.	1.878889	-4.230926
C	1.681700.	-0.092866	-3.941325
H	2.423785.	-2.024049	-3.339095
C	1.745096.	4.603289	2.091164
H	0.380863.	4.166932	3.707362
H	3.020754.	4.746349	0.357933
C	2.164485.	-3.234566	2.716827
H	0.203834.	-4.052035	2.310234
H	6.005421.	-0.855583	-5.074130
C	5.203716.	1.039562	-4.421450
H	4.380449.	2.789154	-3.467518
C	4.346209.	2.205337	3.006560
H	6.104417.	3.107302	2.132316
H	8.034154.	-3.938760	2.119650
C	9.094142.	-2.511559	0.897145
H	9.859776.	-0.991150	-0.427648
H	1.946248.	-0.225390	-4.985820
H	2.018504.	5.581617	2.475738
H	2.540020.	-4.127258	3.208954
H	5.260383.	1.497739	-5.404766
H	4.247795.	2.945326	3.794952
H	10.078124.	-2.862086	1.195887
H	2.629843.	1.082795	3.645853
H	3.998308.	-2.139915	2.979146
C	-2.397953.	-0.510259	-1.184232
H	-1.531506.	-0.353902	-1.830526
H	-3.275692.	0.010247	-1.569159
C	-2.625781.	-1.911476	-0.837974
C	-1.711936.	-2.902358	-0.979409
C	-1.874281.	-4.296658	-0.568942
C	-3.123834.	-4.864078	-0.236932
H	-4.028937.	-4.270461	-0.320059
C	-3.219973.	-6.189134	0.177648
H	-4.195938.	-6.602570	0.419107
C	-2.077365.	-6.991784	0.270768
H	-2.157777.	-8.026262	0.592457
C	-0.834133.	-6.450662	-0.064994
H	0.061758.	-7.063007	-0.003202
C	-0.735510.	-5.123963	-0.482969

H	0.237033.	-4.708882	-0.735955
H	-3.866797.	1.198635	1.937523
H	-4.508590.	-0.242793	1.091385
C	-4.686213.	1.632590	-0.005196
C	-4.015828.	0.722586	0.952920
B	-4.154101.	3.091249	-0.066234
O	-3.207323.	3.582257	0.813338
O	-4.514919.	4.017772	-1.020103
C	-2.704175.	4.826015	0.251018
C	-3.910931.	5.286990	-0.645749
C	-5.625531.	1.236845	-0.903812
Pd	-1.975958.	0.501066	0.590807
H	-6.033165.	1.982055	-1.585327
C	-6.157762.	-0.168722	-1.025469
H	-5.334926.	-0.884889	-0.941131
H	-6.599327.	-0.329812	-2.015718
H	-3.590866.	-2.145927	-0.392320
H	-0.736189.	-2.640496	-1.388568
C	-4.972651.	6.071477	0.132535
H	-5.255236.	5.546104	1.049385
H	-4.622648.	7.073760	0.397324
H	-5.867355.	6.170749	-0.488686
C	-3.521568.	6.034495	-1.916051
H	-4.421424.	6.306347	-2.476113
H	-2.981540.	6.955706	-1.673135
H	-2.894286.	5.421425	-2.565851
C	-1.452673.	4.467030	-0.559041
H	-0.975368.	5.356255	-0.981718
H	-0.737088.	3.970853	0.099126
H	-1.700431.	3.778426	-1.372299
C	-2.342651.	5.771718	1.390084
H	-2.046078.	6.751080	0.999798
H	-3.177006.	5.908310	2.080780
H	-1.498218.	5.361318	1.951299
C	-7.211703.	-0.543535	0.055587
H	-6.850867.	-0.194540	1.028807
H	-8.148996.	-0.012920	-0.146111
C	-7.437892.	-2.037310	0.100786
C	-6.570559.	-2.856202	0.841770
C	-8.452544.	-2.650285	-0.645864
C	-6.712832.	-4.244707	0.837783
C	-8.599009.	-4.039753	-0.653913
C	-7.728507.	-4.842468	0.086270
H	-5.778765.	-2.397061	1.429761
H	-9.132905.	-2.031898	-1.227030
H	-6.032226.	-4.857500	1.422916
H	-9.393956.	-4.495058	-1.238697
H	-7.842036.	-5.922953	0.081006

IV^{Pd}-Me

	Value
Charge	0
Electronic Energy, BS1 (a.u.)	-2471.298906
Thermal and entropic correction, BS1 (a.u.)	0.593598
Electronic Energy, BS2 (a.u.)	-2471.973732

Molecular Geometry in Cartesian Coordinates

Pd	-0.126211	-0.869997	-1.231280
P	1.657051	0.199873	-0.034787
P	-1.778329	0.175686	-0.053935

C	2.977294	-1.040082	0.295313
C	2.494316	1.602517	-0.865984
C	1.275062	0.828967	1.661008
C	-2.049730	-0.270304	1.703645
C	-1.393516	1.995264	-0.074783
C	-3.459715	0.072644	-0.814843
C	2.590630	-2.373204	0.510162
C	4.336205	-0.701872	0.362032
C	2.424948	1.645271	-2.268099
C	3.171204	2.615842	-0.170523
C	0.331065	1.865097	1.845954
C	1.891225	0.265305	2.789988
C	-1.611484	-1.528955	2.137249
C	-2.701122	0.582720	2.607780
C	-2.033033	2.806799	-1.026480
C	-0.376420	2.569730	0.729029
C	-3.544470	0.055384	-2.218704
C	-4.642048	0.002828	-0.065048
H	1.543847	-2.652824	0.424087
C	3.546725	-3.346155	0.802313
C	5.291364	-1.680554	0.642194
H	4.651192	0.322282	0.189102
C	3.035460	2.692210	-2.961070
H	1.891617	0.859456	-2.800999
C	3.775035	3.661113	-0.870289
H	3.218115	2.592234	0.914353
C	0.068921	2.320861	3.146451
H	2.617924	-0.528495	2.664970
C	1.594713	0.710742	4.077560
H	-1.098621	-2.186916	1.441763
C	-1.829024	-1.932435	3.455924
H	-3.026465	1.567477	2.284699
C	-2.918631	0.176142	3.923541
H	-2.822131	2.394420	-1.642984
C	-1.680312	4.143411	-1.205531
C	-0.044067	3.920476	0.539775
H	-2.633398	0.074343	-2.809754
C	-4.783496	-0.000844	-2.854795
H	-4.606235	-0.005040	1.018331
C	-5.881624	-0.067790	-0.705536
H	3.235414	-4.374378	0.962804
C	4.898968	-3.002261	0.866866
H	6.342550	-1.409330	0.684675
C	3.707148	3.700254	-2.265645
H	2.979370	2.722420	-4.045650
H	4.295322	4.445301	-0.327086
C	0.684443	1.749733	4.258045
H	2.083565	0.250854	4.931548
H	-1.483300	-2.908504	3.784321
C	-2.482855	-1.081980	4.349016
H	-3.419376	0.843823	4.618929
C	-0.674972	4.704901	-0.422299
H	-2.194283	4.737879	-1.955243
H	-4.829851	-0.010475	-3.939932
C	-5.956748	-0.063869	-2.098644
H	-6.788622	-0.126663	-0.110389
H	5.644660	-3.762035	1.083394
H	4.174866	4.517046	-2.808541
H	0.448601	2.111357	5.254619
H	-2.648386	-1.394644	5.376172
H	-0.384397	5.743056	-0.553207
H	-6.922212	-0.119542	-2.593420
H	0.736884	4.349790	1.159709
H	-0.649041	3.124783	3.278521
H	2.393689	-2.839247	-2.245528
C	2.313291	-1.909486	-2.842351
H	3.086244	-1.224268	-2.440513
H	2.634316	-2.169510	-3.868340
O	1.032801	-1.370509	-2.879724
C	-1.391780	-2.380151	-1.974412
H	-1.355956	-2.241956	-3.056507
H	-2.413831	-2.340454	-1.593554
C	-0.630448	-3.544641	-1.496888
C	-0.933985	-4.310140	-0.426720

H	0.287764	-3.764722	-2.041536
H	-1.853819	-4.103793	0.124236
C	-0.086649	-5.438946	0.084552
H	0.816606	-5.566788	-0.522268
H	0.228569	-5.269250	1.124941
H	-0.630063	-6.394428	0.083259

V^{Pd}Real-Me

	Value
Charge	0
Electronic Energy, BS1 (a.u.)	-3193.924046
Thermal and entropic correction, BS1 (a.u.)	0.913173
Electronic Energy, BS2 (a.u.)	-3194.838017

Molecular Geometry in Cartesian Coordinates

P	2.117819	-1.024978	-0.628170
P	-0.873879	-1.152016	0.932202
C	3.635466	-0.636869	-1.600022
C	2.886861	-1.302324	1.022062
C	1.486476	-2.607081	-1.326004
C	-2.136047	-2.099251	-0.029967
C	0.024491	-2.601219	1.715134
C	-1.798153	-0.445742	2.358897
C	3.970092	0.715371	-1.760560
C	4.514433	-1.619328	-2.081762
C	2.679486	-0.317842	1.999339
C	3.729894	-2.384848	1.306162
C	0.743090	-3.550716	-0.580544
C	1.508904	-2.718084	-2.730026
C	-2.352683	-1.802864	-1.381341
C	-2.845132	-3.160489	0.558765
C	0.029153	-2.783390	3.107031
C	0.670909	-3.576097	0.914000
C	-1.059623	0.231630	3.345659
C	-3.196347	-0.470950	2.462431
H	3.285864	1.476729	-1.401217
C	5.162588	1.081548	-2.386352
C	5.702109	-1.251749	-2.714508
H	4.268553	-2.671050	-1.971535
C	3.302369	-0.418118	3.244973
H	2.027184	0.523614	1.782985
C	4.342684	-2.488822	2.554011
H	3.896648	-3.152952	0.557028
C	0.080540	-4.583622	-1.265531
H	2.056028	-1.986519	-3.315585
C	0.835347	-3.742230	-3.389988
H	-1.793170	-1.002820	-1.853058
C	-3.269013	-2.544871	-2.129773
H	-2.674390	-3.413388	1.601061
C	-3.762201	-3.897559	-0.188707
H	-0.469285	-2.067068	3.746668
C	0.643426	-3.882799	3.706106
C	1.259038	-4.690413	1.532810
H	0.020763	0.298605	3.259925
C	-1.698768	0.813491	4.439783
H	-3.793946	-0.956848	1.699649
C	-3.836962	0.147080	3.539228
H	5.406336	2.133383	-2.506740
C	6.029701	0.098619	-2.866224
H	6.373592	-2.020249	-3.087297
C	4.131156	-1.505461	3.525102
H	3.133824	0.350348	3.994339
H	4.983372	-3.339006	2.770653
C	0.114315	-4.682612	-2.653213
H	0.871335	-3.798834	-4.474076
H	-3.423606	-2.302022	-3.176826
C	-3.975947	-3.590256	-1.536216

H	-4.306980	-4.713302	0.278568
C	1.257978	-4.849599	2.915663
H	0.625689	-3.981850	4.787699
H	-1.109562	1.319839	5.198901
C	-3.091448	0.778335	4.536867
H	-4.921588	0.125913	3.597322
H	6.955065	0.381371	-3.360088
H	4.611001	-1.589501	4.496150
H	-0.423571	-5.482334	-3.153548
H	-4.688644	-4.167512	-2.118831
H	1.733315	-5.716252	3.365822
H	-3.592223	1.248838	5.378202
H	1.741672	-5.432949	0.903834
H	-0.490251	-5.302247	-0.685757
C	0.686738	1.187624	-2.573205
H	1.683325	0.852251	-2.863167
H	0.661863	2.277838	-2.557447
C	-0.353156	0.559766	-3.410050
C	-1.481260	1.126385	-3.883508
H	-0.948326	3.353419	2.673786
H	-2.374360	2.419154	1.979678
C	-0.717854	2.741859	0.647667
C	-1.387652	2.843640	1.821049
B	0.663599	3.460157	0.566530
O	1.401207	3.801616	1.683254
O	1.261243	3.881622	-0.604120
C	2.669447	4.346954	1.230727
C	2.332971	4.793763	-0.241314
C	-1.199920	2.013523	-0.553410
Pd	0.250886	0.407648	-0.625903
H	-0.963853	2.588624	-1.449193
C	-2.655565	1.582505	-0.601534
H	-2.921388	0.986693	0.276153
H	-2.810250	0.938716	-1.473196
H	-0.200835	-0.500618	-3.630176
H	-1.666374	2.181706	-3.679812
C	3.075766	5.477822	2.170286
H	2.288260	6.227961	2.262775
H	3.273725	5.074288	3.168042
H	3.988558	5.968327	1.816209
C	3.692362	3.208424	1.299935
H	3.733789	2.827177	2.324014
H	3.406561	2.380564	0.648033
H	4.694832	3.544985	1.018851
C	1.746703	6.208079	-0.322230
H	1.353347	6.370678	-1.329715
H	0.921304	6.331560	0.384899
H	2.501162	6.973584	-0.117682
C	3.472923	4.638583	-1.241251
H	3.825917	3.608395	-1.293397
H	3.129784	4.928062	-2.238950
H	4.316153	5.282402	-0.969875
C	-3.668187	2.752029	-0.708875
H	-3.485351	3.296087	-1.643559
H	-3.497063	3.457671	0.111010
C	-5.084850	2.234149	-0.667962
C	-5.702105	1.951489	0.560835
C	-5.784600	1.933916	-1.845164
C	-6.977682	1.388110	0.612126
C	-7.062112	1.371100	-1.799419
C	-7.663938	1.095109	-0.569698
H	-5.169272	2.170185	1.483265
H	-5.320238	2.145058	-2.806051
H	-7.438521	1.180079	1.574687
H	-7.587910	1.149242	-2.724625
H	-8.658098	0.658132	-0.532100
C	-2.546672	0.398467	-4.652539
H	-3.505704	0.400169	-4.114447
H	-2.741020	0.863551	-5.629414
H	-2.267738	-0.646464	-4.832046

TS_V-V'^{Pd}Real-Me

	Value
Charge	0
Electronic Energy, BS1 (a.u.)	-3193.910317
Thermal and entropic correction, BS1 (a.u.)	0.912354
Electronic Energy, BS2 (a.u.)	-3194.824807

Molecular Geometry in Cartesian Coordinates

P	-2.117064	-0.977297	0.610780
P	0.907761	-1.282295	-0.834446
C	-3.647960	-0.487650	1.512638
C	-2.846780	-1.335889	-1.041852
C	-1.552732	-2.531849	1.417679
C	2.072180	-2.193183	0.272636
C	0.003552	-2.755629	-1.565361
C	1.933375	-0.707485	-2.250500
C	-3.949593	0.880470	1.573608
C	-4.565792	-1.412906	2.033283
C	-2.569593	-0.432066	-2.077886
C	-3.724954	-2.402116	-1.278030
C	-0.833563	-3.548761	0.749262
C	-1.608369	-2.552567	2.824949
C	2.165772	-1.829965	1.621935
C	2.810397	-3.295084	-0.190812
C	0.052688	-3.021315	-2.942882
C	-0.708329	-3.660951	-0.737743
C	1.271326	-0.070924	-3.315884
C	3.332897	-0.787787	-2.278778
H	-3.235005	1.598744	1.187194
C	-5.149293	1.317661	2.136400
C	-5.760684	-0.973722	2.603985
H	-4.344968	-2.475406	2.002160
C	-3.157382	-0.596642	-3.333758
H	-1.891843	0.398172	-1.900222
C	-4.303171	-2.570582	-2.535213
H	-3.946328	-3.107927	-0.483124
C	-0.237128	-4.567717	1.512132
H	-2.130723	-1.760323	3.351241
C	-0.997422	-3.562645	3.562134
H	1.567668	-1.002230	1.989790
C	2.991507	-2.543563	2.492557
H	2.729591	-3.602750	-1.229296
C	3.640124	-4.002973	0.677437
H	0.602017	-2.360855	-3.600679
C	-0.571906	-4.134962	-3.503590
C	-1.307085	-4.791016	-1.316681
H	0.191661	0.042539	-3.290031
C	1.985647	0.412077	-4.411557
H	3.873390	-1.239307	-1.454821
C	4.049251	-0.267409	-3.359451
H	-5.367990	2.381014	2.178547
C	-6.056282	0.391419	2.654110
H	-6.462739	-1.698230	3.007304
C	-4.021460	-1.667820	-3.564962
H	-2.934192	0.109397	-4.128863
H	-4.972068	-3.407945	-2.713042
C	-0.307680	-4.580084	2.901543
H	-1.057070	-3.548754	4.646530
H	3.046196	-2.254125	3.538007
C	3.732459	-3.627463	2.021503
H	4.210283	-4.850653	0.307490
C	-1.252844	-5.032465	-2.686349
H	-0.510688	-4.299406	-4.575605
H	1.454811	0.887693	-5.231179
C	3.378931	0.320129	-4.434071
H	5.133651	-0.329939	-3.359051
H	-6.987792	0.730073	3.098814
H	-4.474508	-1.801579	-4.543302
H	0.181028	-5.371117	3.462494

H	4.375466	-4.183800	2.697902
H	-1.738130	-5.908950	-3.105827
H	3.938522	0.713945	-5.277749
H	-1.840751	-5.479301	-0.667596
H	0.314719	-5.344252	0.991731
C	-0.760131	1.399313	2.371084
H	-1.627096	0.884560	2.798125
H	-1.076282	2.413587	2.107689
C	0.347396	1.421758	3.399763
C	0.266954	2.000534	4.604690
H	1.184136	3.074827	-2.865412
H	2.557460	2.170322	-2.040452
C	0.849493	2.619917	-0.813712
C	1.573223	2.621288	-1.958212
B	-0.517245	3.368889	-0.863106
O	-1.215216	3.578389	-2.036712
O	-1.136846	3.950602	0.223353
C	-2.481764	4.205249	-1.699590
C	-2.182139	4.824005	-0.282152
C	1.264278	1.976528	0.463640
Pd	-0.220315	0.413568	0.581765
H	1.021507	2.640890	1.295761
C	2.711317	1.519326	0.583878
H	2.985030	0.872417	-0.254090
H	2.840118	0.910819	1.485803
H	1.289737	0.927005	3.155605
H	-0.656107	2.515487	4.882320
C	-2.825427	5.219787	-2.785618
H	-2.014728	5.933846	-2.941533
H	-3.001589	4.699869	-3.732306
H	-3.735342	5.771917	-2.528124
C	-3.533644	3.092176	-1.664689
H	-3.554764	2.588039	-2.634648
H	-3.291757	2.344201	-0.907430
H	-4.534094	3.485335	-1.460587
C	-1.576796	6.230705	-0.353167
H	-1.214457	6.510284	0.640154
H	-0.726277	6.257942	-1.040473
H	-2.312120	6.973990	-0.675521
C	-3.353351	4.803727	0.693888
H	-3.716936	3.790012	0.866397
H	-3.036781	5.215605	1.656749
H	-4.182067	5.412775	0.317916
C	3.742384	2.676017	0.653904
H	3.569092	3.251099	1.571531
H	3.577038	3.357105	-0.187397
C	5.153314	2.141507	0.621951
C	5.769147	1.836307	-0.602348
C	5.849956	1.852194	1.803718
C	7.040909	1.263582	-0.644669
C	7.123266	1.279133	1.766818
C	7.724198	0.982086	0.541626
H	5.238901	2.045836	-1.528314
H	5.386715	2.080106	2.761247
H	7.501487	1.039952	-1.603866
H	7.646815	1.066357	2.695425
H	8.715468	0.538026	0.510964
C	1.365631	2.012655	5.630716
H	1.048727	1.538062	6.569829
H	2.255763	1.484070	5.270917
H	1.665012	3.038164	5.887406

V'^{Pd}Real-Me

	Value
Charge	0
Electronic Energy, BS1 (a.u.)	-3193.924377
Thermal and entropic correction, BS1 (a.u.)	0.9140449
Electronic Energy, BS2 (a.u.)	-3194.838453

Molecular Geometry in Cartesian Coordinates

P	2.104826	-1.042886	-0.465511
P	-0.956099	-1.060224	0.953653
C	3.669058	-0.713539	-1.384770
C	2.780730	-1.144340	1.245252
C	1.544215	-2.701580	-1.035439
C	-2.162905	-2.093697	0.011820
C	-0.096035	-2.426956	1.909465
C	-1.950987	-0.239027	2.267021
C	3.979658	0.622142	-1.677816
C	4.599918	-1.715621	-1.700718
C	2.518363	-0.067209	2.104640
C	3.601906	-2.190286	1.687008
C	0.757397	-3.574416	-0.248396
C	1.667132	-2.959773	-2.413670
C	-2.288911	-1.932104	-1.374018
C	-2.918316	-3.088387	0.656220
C	-0.168704	-2.486213	3.310434
C	0.598740	-3.462307	1.235466
C	-1.259902	0.521568	3.227392
C	-3.352245	-0.264629	2.308987
H	3.257784	1.398212	-1.446753
C	5.198209	0.952333	-2.273131
C	5.814004	-1.384680	-2.302574
H	4.373934	-2.755702	-1.486571
C	3.065373	-0.040012	3.389056
H	1.883007	0.747166	1.768169
C	4.138627	-2.166426	2.973478
H	3.808530	-3.030885	1.031561
C	0.143493	-4.676336	-0.867780
H	2.252626	-2.288335	-3.031875
C	1.042335	-4.051344	-3.010044
H	-1.681748	-1.194756	-1.889220
C	-3.168717	-2.737977	-2.100537
H	-2.814386	-3.238465	1.726846
C	-3.796134	-3.890645	-0.070843
H	-0.706145	-1.721929	3.855817
C	0.417143	-3.521380	4.038317
C	1.158475	-4.510626	1.983253
H	-0.177039	0.588484	3.185220
C	-1.949626	1.184487	4.241728
H	-3.913383	-0.813922	1.561715
C	-4.042795	0.433456	3.302853
H	5.422527	1.990985	-2.499090
C	6.116512	-0.050305	-2.588417
H	6.525177	-2.169002	-2.546457
C	3.872542	-1.091155	3.826419
H	2.853941	0.799464	4.045629
H	4.761907	-2.989134	3.312436
C	0.270346	-4.915054	-2.233034
H	1.153623	-4.217740	-4.077576
H	-3.254594	-2.601030	-3.174014
C	-3.924249	-3.714709	-1.452211
H	-4.377067	-4.654369	0.438868
C	1.081266	-4.547245	3.372435
H	0.338731	-3.524158	5.121756
H	-1.396811	1.754352	4.982990
C	-3.345087	1.147063	4.279284
H	-5.128708	0.410047	3.313287
H	7.062358	0.204281	-3.058230
H	4.292546	-1.075888	4.828232
H	-0.232847	-5.764851	-2.684620
H	-4.607052	-4.341873	-2.018859
H	1.536300	-5.365372	3.923164
H	-3.884837	1.679039	5.057575
H	1.680369	-5.300242	1.450388
H	-0.461635	-5.338815	-0.256737
C	0.783380	1.024552	-2.634674
H	1.734330	0.575863	-2.928367
H	0.886666	2.110261	-2.595142
C	-0.314638	0.544455	-3.494079
C	-0.262685	-0.516182	-4.323836
H	-1.057260	3.565282	2.341308

H	-2.472680	2.606825	1.661015
C	-0.754584	2.793553	0.380869
C	-1.471030	2.998409	1.512321
B	0.641673	3.485225	0.315420
O	1.337098	3.876117	1.443013
O	1.290404	3.845557	-0.848538
C	2.620233	4.408849	1.017524
C	2.344366	4.779463	-0.487981
C	-1.205516	1.986443	-0.784116
Pd	0.231477	0.363341	-0.681475
H	-0.936564	2.508233	-1.705374
C	-2.670301	1.574944	-0.837843
H	-2.967009	1.074771	0.087677
H	-2.824138	0.841480	-1.636683
H	-1.265765	1.074552	-3.417268
H	0.676948	-1.061845	-4.415586
C	2.976716	5.589178	1.915733
H	2.179269	6.334182	1.937503
H	3.135860	5.237549	2.939723
H	3.899286	6.071508	1.576119
C	3.648731	3.286362	1.187200
H	3.647102	2.953679	2.228737
H	3.402514	2.425140	0.563213
H	4.659430	3.621980	0.935990
C	1.757844	6.184976	-0.665659
H	1.407893	6.294261	-1.696102
H	0.902099	6.340282	-0.002186
H	2.500400	6.963771	-0.467989
C	3.525549	4.579982	-1.430495
H	3.890007	3.552754	-1.409402
H	3.221242	4.809618	-2.455988
H	4.351260	5.247181	-1.161844
C	-3.655239	2.748376	-1.082558
H	-3.465853	3.172379	-2.076389
H	-3.458321	3.541529	-0.353772
C	-5.086357	2.282315	-0.971034
C	-5.703702	2.183051	0.286062
C	-5.804140	1.854754	-2.096748
C	-6.997626	1.676661	0.413751
C	-7.099739	1.347231	-1.974400
C	-7.702088	1.256048	-0.717753
H	-5.157189	2.501195	1.170662
H	-5.340373	1.922108	-3.078483
H	-7.458700	1.613039	1.396347
H	-7.639276	1.024618	-2.861222
H	-8.710503	0.863088	-0.620783
C	-1.415010	-1.045134	-5.126687
H	-1.613300	-2.100119	-4.889405
H	-2.333816	-0.478941	-4.935604
H	-1.217881	-1.006515	-6.207906

VI^{Pd}Real-Me

	Value
Charge	0
Electronic Energy, BS1 (a.u.)	-3193.913873
Thermal and entropic correction, BS1 (a.u.)	0.908263
Electronic Energy, BS2 (a.u.)	-3194.827957

Molecular Geometry in Cartesian Coordinates

P	0.070260	-0.214628	0.674856
P	4.794401	1.309167	-0.429133
C	0.677852	0.108425	2.389399
C	0.949118	-1.767727	0.234394
C	0.661811	1.259235	-0.293997
C	5.073282	0.220329	1.045883
C	3.919528	0.122040	-1.571713
C	6.475159	1.403345	-1.193695

C	0.531311	-0.928686	3.327289
C	1.152409	1.352037	2.829049
C	0.240048	-2.759931	-0.458725
C	2.279127	-2.009838	0.611430
C	1.710840	1.369043	-1.232952
C	-0.109190	2.408758	-0.007449
C	4.469432	0.611512	2.249157
C	5.786033	-0.991051	1.012432
C	4.605616	-0.929062	-2.205759
C	2.522245	0.225753	-1.757142
C	6.601047	1.651222	-2.572769
C	7.640989	1.390756	-0.410058
H	0.164241	-1.899403	3.005992
C	0.852742	-0.726186	4.668065
C	1.464585	1.555960	4.175983
H	1.282151	2.168207	2.127661
C	0.863256	-3.962429	-0.797488
H	-0.796875	-2.600391	-0.732181
C	2.897774	-3.210036	0.266938
H	2.834835	-1.265018	1.167648
C	1.956200	2.624250	-1.824425
H	-0.936700	2.333670	0.691963
C	0.157696	3.640316	-0.594681
H	3.914280	1.542932	2.292263
C	4.547182	-0.197880	3.386439
H	6.271237	-1.309290	0.095876
C	5.876401	-1.792842	2.149163
H	5.681603	-1.008606	-2.092988
C	3.938339	-1.864432	-2.991199
C	1.862859	-0.721796	-2.557023
H	5.715616	1.665682	-3.202434
C	7.853004	1.858646	-3.151098
H	7.574663	1.207285	0.657979
C	8.893613	1.601865	-0.990344
H	0.734032	-1.540014	5.377780
C	1.314078	0.520862	5.099383
H	1.829449	2.527181	4.498574
C	2.193294	-4.187740	-0.441046
H	0.304561	-4.719736	-1.340574
H	3.932246	-3.373017	0.551479
C	1.204795	3.750318	-1.511321
H	-0.456450	4.500375	-0.344586
H	4.045273	0.109345	4.297947
C	5.247378	-1.402311	3.336678
H	6.430732	-2.726743	2.108480
C	2.556459	-1.763863	-3.162847
H	4.493605	-2.668914	-3.465275
H	7.927496	2.038372	-4.220246
C	9.005896	1.833674	-2.362128
H	9.783215	1.580758	-0.366488
H	1.552739	0.682902	6.146693
H	2.680094	-5.121654	-0.708598
H	1.424604	4.700244	-1.989664
H	5.305229	-2.036347	4.216954
H	2.021617	-2.490208	-3.767279
H	9.981214	1.994289	-2.812735
H	0.790277	-0.628755	-2.696678
H	2.753955	2.696711	-2.557346
C	-2.820882	0.055351	2.402042
H	-2.174268	-0.578358	3.017624
H	-3.861380	-0.270123	2.505696
C	-2.647052	1.486258	2.708569
C	-3.605368	2.433878	2.746733
H	-1.754265	-1.227588	-2.419959
H	-2.120163	0.559838	-2.219551
C	-3.539382	-0.823055	-1.275287
C	-2.322958	-0.460473	-1.902849
B	-3.868128	-2.342101	-1.080948
O	-3.012642	-3.358796	-1.440650
O	-5.021995	-2.788796	-0.488722
C	-3.507056	-4.570276	-0.790972
C	-5.034791	-4.242033	-0.607590
C	-4.250324	0.106955	-0.458515
Pd	-2.229751	-0.284924	0.378200

H	-5.084604	-0.303410	0.108636
C	-4.311689	1.602369	-0.661304
H	-3.568432	1.937250	-1.392515
H	-4.069346	2.112472	0.279955
H	-1.617702	1.810454	2.879720
H	-4.644841	2.133388	2.599970
C	-5.875396	-4.572874	-1.844714
H	-5.423755	-4.158598	-2.750823
H	-5.992968	-5.652750	-1.974692
H	-6.868191	-4.129180	-1.728766
C	-5.675011	-4.825210	0.646289
H	-6.729779	-4.537941	0.689474
H	-5.621336	-5.918771	0.634749
H	-5.189242	-4.459145	1.552496
C	-2.758666	-4.694947	0.540719
H	-3.053601	-5.598929	1.081279
H	-1.684370	-4.744975	0.348782
H	-2.943248	-3.825588	1.178858
C	-3.204213	-5.762761	-1.689191
H	-3.635917	-6.678610	-1.272668
H	-3.594609	-5.618561	-2.698213
H	-2.121520	-5.903735	-1.763209
C	-5.711769	2.088943	-1.111099
H	-5.959275	1.646465	-2.083148
H	-6.458337	1.724746	-0.394145
C	-5.763951	3.597410	-1.187312
C	-5.544951	4.271792	-2.395765
C	-5.960750	4.359464	-0.025293
C	-5.525619	5.667795	-2.445564
C	-5.941037	5.754088	-0.069500
C	-5.722869	6.414055	-1.282044
H	-5.389100	3.696163	-3.305432
H	-6.128276	3.851087	0.921296
H	-5.357968	6.171770	-3.393810
H	-6.098814	6.325135	0.841647
H	-5.709371	7.499834	-1.319216
C	-3.360577	3.899115	2.974107
H	-2.293676	4.108592	3.115316
H	-3.708581	4.504685	2.125261
H	-3.893003	4.273943	3.860153

VI’^{Pd}Real-Me

	Value
Charge	0
Electronic Energy, BS1 (a.u.)	-3193.913157
Thermal and entropic correction, BS1 (a.u.)	0.908620
Electronic Energy, BS2 (a.u.)	-3194.827682

Molecular Geometry in Cartesian Coordinates

P	-0.112182	-0.450150	0.832091
P	4.639325	0.938540	-0.318638
C	0.586632	-0.614675	2.530732
C	0.595054	-1.917165	-0.019742
C	0.580268	1.166442	0.221819
C	4.953103	-0.489344	0.823789
C	3.570118	0.122945	-1.611555
C	6.248718	1.107071	-1.213090
C	0.274874	-1.790347	3.235220
C	1.319340	0.387180	3.179485
C	-0.244807	-2.672551	-0.851713
C	1.921316	-2.331191	0.176785
C	1.527551	1.424284	-0.791529
C	-0.024024	2.262994	0.878868
C	4.485487	-0.364740	2.139598
C	5.561275	-1.696691	0.437708
C	4.102225	-0.805321	-2.524184
C	2.181197	0.381862	-1.644128

C	6.275565	1.651868	-2.509707
C	7.472558	0.849216	-0.573312
H	-0.297260	-2.575504	2.748232
C	0.688723	-1.958372	4.555085
C	1.727792	0.220496	4.505550
H	1.571930	1.304155	2.660408
C	0.244247	-3.807691	-1.500573
H	-1.278094	-2.378052	-0.996627
C	2.407803	-3.458974	-0.481941
H	2.576817	-1.774269	0.835233
C	1.826700	2.764905	-1.108353
H	-0.754744	2.076706	1.661783
C	0.292012	3.577616	0.555547
H	4.014765	0.560686	2.454660
C	4.589695	-1.427253	3.042183
H	5.942631	-1.813284	-0.571087
C	5.680900	-2.751894	1.340778
H	5.169878	-0.997449	-2.528132
C	3.292395	-1.475590	-3.436212
C	1.378054	-0.293333	-2.576860
H	5.343555	1.859974	-3.028015
C	7.486243	1.910099	-3.152180
H	7.483163	0.433965	0.429843
C	8.683835	1.110824	-1.217086
H	0.437921	-2.872622	5.085445
C	1.412803	-0.949044	5.197726
H	2.290933	1.010561	4.994533
C	1.571839	-4.198925	-1.322875
H	-0.415539	-4.380617	-2.146404
H	3.441994	-3.752934	-0.334519
C	1.225211	3.831864	-0.452183
H	-0.200751	4.393473	1.075761
H	4.189667	-1.319798	4.045341
C	5.184855	-2.622946	2.643020
H	6.153780	-3.679021	1.027863
C	1.919913	-1.221455	-3.459339
H	3.730000	-2.193014	-4.124719
H	7.483272	2.321307	-4.158068
C	8.696615	1.639139	-2.509011
H	9.618902	0.896502	-0.706446
H	1.724855	-1.074375	6.230700
H	1.955273	-5.078206	-1.833381
H	1.477006	4.851661	-0.728535
H	5.264082	-3.452451	3.340051
H	1.274673	-1.740450	-4.161731
H	9.639549	1.838919	-3.010076
H	0.314411	-0.081101	-2.597154
H	2.544855	2.954818	-1.900386
C	-2.966037	-0.252117	2.639756
H	-2.378471	-1.016006	3.159494
H	-4.021244	-0.547789	2.630414
C	-2.769333	1.090730	3.224618
C	-1.825341	1.450360	4.114912
H	-1.973230	-0.550946	-2.347510
H	-2.077621	1.191205	-1.778020
C	-3.713339	-0.125674	-1.145351
C	-2.438396	0.170489	-1.683341
B	-4.269145	-1.583127	-1.275345
O	-3.565365	-2.611911	-1.859979
O	-5.481859	-1.974466	-0.768273
C	-4.232820	-3.850225	-1.467356
C	-5.699692	-3.355269	-1.183710
C	-4.300085	0.719438	-0.157995
Pd	-2.400000	-0.184970	0.591771
H	-5.234547	0.371122	0.276586
C	-4.051876	2.213905	-0.073892
H	-2.980015	2.432276	-0.022201
H	-4.483196	2.603116	0.854371
H	-3.434144	1.875205	2.853341
H	-1.154174	0.685956	4.504683
C	-6.571696	-3.301198	-2.441798
H	-6.060220	-2.775561	-3.253414
H	-6.841023	-4.303195	-2.788559
H	-7.491910	-2.756339	-2.213082

C	-6.423004	-4.090017	-0.061570
H	-7.426235	-3.672589	0.065791
H	-6.525699	-5.153837	-0.299588
H	-5.894543	-3.992000	0.888389
C	-3.517511	-4.357240	-0.210068
H	-3.943014	-5.302956	0.137683
H	-2.460692	-4.519216	-0.436206
H	-3.576855	-3.623739	0.599666
C	-4.098071	-4.857383	-2.602343
H	-4.656493	-5.771257	-2.375020
H	-4.460156	-4.451440	-3.548614
H	-3.045870	-5.128670	-2.732004
C	-4.631082	3.008010	-1.274024
H	-4.257949	2.553957	-2.199500
H	-5.722841	2.913067	-1.288245
C	-4.221497	4.461867	-1.212689
C	-2.901637	4.830611	-1.519341
C	-5.109688	5.459743	-0.792194
C	-2.482179	6.156009	-1.408970
C	-4.695328	6.790032	-0.683917
C	-3.379938	7.142733	-0.990705
H	-2.196695	4.068179	-1.843136
H	-6.134965	5.191587	-0.547699
H	-1.455709	6.418641	-1.651463
H	-5.401016	7.549994	-0.358889
H	-3.056534	8.176519	-0.906007
C	-1.594532	2.850744	4.606565
H	-0.579946	3.200177	4.364773
H	-2.303302	3.558075	4.160205
H	-1.690855	2.925196	5.699207

TS-γγ'-RR_Real-Me

	Value
Charge	0
Electronic Energy, BS1 (a.u.)	-3193.886086
Thermal and entropic correction, BS1 (a.u.)	0.909310
Electronic Energy, BS2 (a.u.)	-3194.800652

Molecular Geometry in Cartesian Coordinates

P	-0.628412	-1.476706	-0.706440
P	-4.780806	1.513962	-0.284152
C	-1.697301	-2.518840	-1.802280
C	-1.330645	-1.851176	0.954052
C	-0.983624	0.262381	-1.267227
C	-5.519772	-0.184726	-0.198564
C	-3.529213	1.419317	1.097455
C	-6.094853	2.576768	0.464469
C	-1.707553	-3.901107	-1.540863
C	-2.424859	-2.036551	-2.898440
C	-0.431390	-2.153756	1.988767
C	-2.709148	-1.915131	1.205976
C	-1.623285	1.330366	-0.601877
C	-0.423145	0.506592	-2.541505
C	-5.333950	-1.015566	-1.312890
C	-6.180425	-0.706009	0.927487
C	-3.921313	1.428513	2.447570
C	-2.156859	1.268941	0.794200
C	-5.742418	3.784332	1.094345
C	-7.458904	2.294103	0.282205
H	-1.149963	-4.294079	-0.694414
C	-2.431262	-4.773946	-2.350550
C	-3.139283	-2.915683	-3.717792
H	-2.443394	-0.975506	-3.118964
C	-0.901886	-2.482116	3.261443
H	0.636900	-2.134827	1.794663
C	-3.175763	-2.235572	2.479181
H	-3.418622	-1.712552	0.413502

C	-1.691577	2.583593	-1.243380
H	0.108061	-0.297588	-3.043839
C	-0.518377	1.744380	-3.166235
H	-4.824178	-0.628878	-2.189798
C	-5.768199	-2.344274	-1.296837
H	-6.344947	-0.078866	1.797391
C	-6.629531	-2.025728	0.938808
H	-4.968303	1.561045	2.698706
C	-2.993482	1.285832	3.475577
C	-1.230311	1.138052	1.841138
H	-4.694774	4.028399	1.247314
C	-6.722171	4.667740	1.546831
H	-7.761282	1.373226	-0.207013
C	-8.438527	3.181255	0.733260
H	-2.430952	-5.837617	-2.129290
C	-3.146997	-4.284413	-3.447667
H	-3.696571	-2.522867	-4.563977
C	-2.274344	-2.515904	3.510144
H	-0.194960	-2.711129	4.054064
H	-4.245440	-2.263010	2.659888
C	-1.159808	2.796565	-2.508643
H	-0.077449	1.889719	-4.147997
H	-5.582996	-2.979975	-2.156729
C	-6.414581	-2.851262	-0.170454
H	-7.139191	-2.414937	1.816211
C	-1.639484	1.135877	3.170407
H	-3.326184	1.289931	4.509736
H	-6.426995	5.589783	2.040402
C	-8.075563	4.369275	1.369744
H	-9.487984	2.938872	0.588653
H	-3.703479	-4.965334	-4.085445
H	-2.642978	-2.765778	4.501314
H	-1.229364	3.777286	-2.969675
H	-6.750122	-3.884314	-0.152830
H	-0.905228	1.019511	3.961812
H	-8.838619	5.056788	1.723487
H	-0.177421	1.035724	1.596982
H	-2.169867	3.403016	-0.715593
C	3.625498	-2.643775	-1.360135
H	3.114982	-3.556644	-1.709876
H	4.222193	-2.810135	-0.462265
C	4.202262	-1.831592	-2.367975
C	5.227647	-0.919500	-2.154116
H	1.822317	-0.290884	1.127850
H	1.522464	0.616717	-0.416244
C	3.590999	0.213564	0.048977
C	2.191453	-0.043070	0.132477
B	4.497391	-0.321948	1.176085
O	4.024842	-0.907149	2.334642
O	5.877376	-0.275749	1.134982
C	5.164948	-1.486824	3.025325
C	6.362251	-0.638317	2.456171
C	4.187497	0.805212	-1.071438
Pd	1.675751	-1.898568	-0.886746
H	5.197795	1.180868	-0.940114
C	3.379024	1.466371	-2.159875
H	4.000065	1.618439	-3.048395
H	2.544821	0.817454	-2.450631
H	3.731313	-1.844175	-3.353061
H	5.824135	-1.050434	-1.254695
C	7.676953	-1.394435	2.303192
H	8.440089	-0.727372	1.890998
H	8.031461	-1.757479	3.273544
H	7.574500	-2.245044	1.626798
C	5.229345	-2.962833	2.617517
H	6.018140	-3.497303	3.155481
H	4.270859	-3.437151	2.847229
H	5.407525	-3.067029	1.543830
C	4.940998	-1.361682	4.528249
H	4.087872	-1.978804	4.826635
H	5.819799	-1.708364	5.081940
H	4.728076	-0.331265	4.819045
C	6.585121	0.673988	3.216645
H	5.646485	1.223424	3.334619

H	7.016858	0.501596	4.207102
H	7.272613	1.302513	2.643516
C	2.805891	2.847075	-1.734805
H	2.202482	3.240294	-2.561429
H	2.127732	2.708672	-0.886957
C	3.887376	3.838072	-1.368733
C	4.497002	4.630991	-2.351042
C	4.348968	3.943498	-0.048165
C	5.536147	5.505505	-2.026816
C	5.388909	4.814558	0.280821
C	5.986604	5.599836	-0.707954
H	4.151328	4.561340	-3.380263
H	3.892649	3.326553	0.721267
H	5.992461	6.114353	-2.803020
H	5.732330	4.880002	1.309959
H	6.794568	6.280174	-0.453047
C	5.921779	-0.218934	-3.294203
H	6.281257	0.777776	-3.011936
H	6.801940	-0.788992	-3.624137
H	5.263020	-0.105901	-4.162356

TS-γγ'-RS_Real-Me

	Value
Charge	0
Electronic Energy, BS1 (a.u.)	-3193.880967
Thermal and entropic correction, BS1 (a.u.)	0.907392
Electronic Energy, BS2 (a.u.)	-3194.794736

Molecular Geometry in Cartesian Coordinates

P	0.185243	0.232989	1.624493
P	4.415728	0.957158	-1.389642
C	1.006230	1.151955	3.000069
C	1.073434	-1.380953	1.685599
C	0.654476	1.246206	0.136854
C	5.134665	0.830440	0.311339
C	3.340405	-0.561720	-1.512191
C	5.849291	0.463126	-2.453777
C	0.427196	1.072516	4.276137
C	2.193285	1.881442	2.834872
C	0.302591	-2.546590	1.559187
C	2.445756	-1.492647	1.945918
C	1.281335	0.863068	-1.067349
C	0.113894	2.548334	0.229077
C	5.404622	2.036679	0.977372
C	5.455375	-0.378440	0.950000
C	3.890628	-1.818959	-1.815366
C	1.939277	-0.459743	-1.317690
C	5.611138	0.310865	-3.832619
C	7.163322	0.322479	-1.983660
H	-0.497393	0.516109	4.411557
C	1.025743	1.703378	5.367515
C	2.788015	2.515122	3.926873
H	2.657796	1.955994	1.856992
C	0.898712	-3.802461	1.682850
H	-0.756867	-2.461283	1.337419
C	3.040340	-2.748673	2.057848
H	3.053895	-0.604235	2.066758
C	1.295804	1.779496	-2.140578
H	-0.382184	2.848520	1.148296
C	0.175919	3.449665	-0.825324
H	5.158039	2.979968	0.496711
C	5.984000	2.036694	2.248884
H	5.245141	-1.321179	0.456318
C	6.024097	-0.379236	2.224191
H	4.959984	-1.907961	-1.977382
C	3.089857	-2.953609	-1.926697
C	1.145641	-1.609379	-1.443267

H	4.601374	0.420538	-4.221137
C	6.650508	0.005805	-4.708887
H	7.377961	0.437294	-0.926279
C	8.207412	0.028105	-2.865671
H	0.565578	1.635148	6.349366
C	2.207203	2.427993	5.193985
H	3.710315	3.069669	3.784086
C	2.268937	-3.906527	1.929325
H	0.293193	-4.699508	1.587918
H	4.106535	-2.820056	2.252097
C	0.754607	3.053157	-2.034228
H	-0.239959	4.446327	-0.710441
H	6.191774	2.978911	2.748306
C	6.288828	0.828121	2.876939
H	6.261099	-1.322130	2.709589
C	1.712217	-2.846504	-1.741551
H	3.541054	-3.914313	-2.159026
H	6.443275	-0.117640	-5.768444
C	7.955915	-0.135318	-4.227783
H	9.218546	-0.078240	-2.481675
H	2.671327	2.924635	6.041543
H	2.733640	-4.883954	2.025618
H	0.785322	3.731007	-2.882037
H	6.728624	0.825142	3.870158
H	1.075880	-3.720729	-1.828779
H	8.768133	-0.367673	-4.910792
H	0.071759	-1.540780	-1.299829
H	1.755444	1.467194	-3.073069
C	-4.166392	0.261732	2.551689
H	-3.668316	-0.031106	3.492688
H	-4.435000	1.320162	2.503233
C	-5.081737	-0.654146	2.010365
C	-6.070354	-0.339685	1.080625
H	-6.425991	0.687580	1.088235
H	-1.546263	-0.165788	-0.702749
H	-2.482994	1.362040	-0.467777
C	-3.651209	-0.446837	-0.755969
C	-2.487166	0.275571	-0.388140
B	-3.524653	-1.975563	-0.934408
O	-2.359156	-2.690430	-0.719644
O	-4.575949	-2.791071	-1.304864
C	-2.714336	-4.102276	-0.724229
C	-4.026450	-4.106445	-1.588451
C	-4.935459	0.123088	-0.825025
Pd	-2.158326	0.117653	1.780736
H	-5.684377	-0.463892	-1.349263
C	-5.119573	1.621669	-0.865958
H	-6.172975	1.888401	-0.726643
H	-4.567313	2.086034	-0.039726
H	-4.893892	-1.712917	2.204632
C	-5.057080	-5.160734	-1.201422
H	-5.936324	-5.069826	-1.846221
H	-4.647112	-6.168327	-1.326975
H	-5.386017	-5.043538	-0.167299
C	-2.966894	-4.505279	0.733348
H	-3.198306	-5.570797	0.822126
H	-2.073990	-4.298712	1.327739
H	-3.793360	-3.929890	1.160077
C	-1.551903	-4.898635	-1.304282
H	-0.686774	-4.820160	-0.640248
H	-1.815302	-5.957351	-1.397412
H	-1.259946	-4.523540	-2.286937
C	-3.751016	-4.156306	-3.095461
H	-3.005431	-3.409392	-3.383487
H	-3.398035	-5.142929	-3.410198
H	-4.677034	-3.933193	-3.632833
C	-4.638081	2.244532	-2.205077
H	-3.626934	1.887839	-2.422215
H	-5.283394	1.882717	-3.014490
C	-4.648407	3.754348	-2.157664
C	-3.514978	4.454927	-1.718591
C	-5.796644	4.486003	-2.490687
C	-3.527964	5.846775	-1.616015
C	-5.814112	5.878661	-2.389852

C	-4.679125	6.564381	-1.950980
H	-2.614501	3.900820	-1.463745
H	-6.683107	3.957714	-2.834810
H	-2.638676	6.373153	-1.278607
H	-6.712837	6.428577	-2.656716
H	-4.690428	7.648094	-1.873880
C	-7.060273	-1.380608	0.613945
H	-6.550409	-2.302109	0.316402
H	-7.785417	-1.628268	1.402424
H	-7.635418	-1.030321	-0.249882

3-(RR)-Pd_Real-Me

	Value
Charge	0
Electronic Energy, BS1 (a.u.)	-3193.947548
Thermal and entropic correction, BS1 (a.u.)	0.913337
Electronic Energy, BS2 (a.u.)	-3194.856484

Molecular Geometry in Cartesian Coordinates

P	0.501822	0.599380	-0.781586
P	5.291806	-1.156152	-0.368538
C	1.376407	1.806565	-1.884811
C	1.075587	1.140749	0.884529
C	1.243032	-1.033220	-1.285339
C	5.597910	0.669097	-0.241777
C	4.081343	-1.408063	1.028353
C	6.839998	-1.879536	0.337987
C	1.112787	3.170317	-1.661790
C	2.185110	1.449370	-2.971359
C	0.136980	1.155499	1.928320
C	2.381067	1.590382	1.132556
C	2.185509	-1.858508	-0.633066
C	0.725578	-1.460939	-2.528190
C	5.224913	1.452029	-1.343392
C	6.112028	1.312255	0.897637
C	4.485338	-1.312568	2.372054
C	2.713802	-1.628728	0.748041
C	6.807864	-3.142294	0.956844
C	8.089168	-1.269484	0.134300
H	0.478335	3.465376	-0.829952
C	1.651545	4.147225	-2.495512
C	2.712296	2.430337	-3.818055
H	2.413482	0.407225	-3.164567
C	0.507083	1.587422	3.203466
H	-0.884579	0.837017	1.745654
C	2.748752	2.013956	2.408766
H	3.113528	1.607457	0.334698
C	2.585544	-3.055048	-1.260908
H	-0.023768	-0.847303	-3.021549
C	1.144877	-2.637165	-3.139329
H	4.826653	0.972134	-2.231093
C	5.329170	2.845408	-1.302060
H	6.421701	0.727298	1.757130
C	6.231394	2.700762	0.935411
H	5.534675	-1.165232	2.603971
C	3.571880	-1.416832	3.416637
C	1.804352	-1.739564	1.812866
H	5.855517	-3.636063	1.128797
C	7.984122	-3.763910	1.375818
H	8.145539	-0.296832	-0.344978
C	9.265924	-1.894161	0.552321
H	1.440594	5.195498	-2.302397
C	2.450717	3.780024	-3.583691
H	3.335485	2.132632	-4.657150
C	1.813875	2.012020	3.447736
H	-0.228868	1.589613	4.003119
H	3.768425	2.341695	2.584732

C	2.088847	-3.442749	-2.499194
H	0.729009	-2.926454	-4.100192
H	5.003031	3.432346	-2.154704
C	5.829696	3.471529	-0.161416
H	6.630684	3.184096	1.823155
C	2.220611	-1.626187	3.134742
H	3.912278	-1.332926	4.444902
H	7.934549	-4.734989	1.861091
C	9.219092	-3.141820	1.176644
H	10.220612	-1.400328	0.391848
H	2.861048	4.540259	-4.242479
H	2.104072	2.343708	4.441076
H	2.422725	-4.371657	-2.952176
H	5.908793	4.554374	-0.123350
H	1.495008	-1.703025	3.938828
H	10.134947	-3.625430	1.504405
H	0.757353	-1.915918	1.586143
H	3.297119	-3.691796	-0.743989
C	-2.426828	1.829542	-2.730172
H	-2.197785	2.879648	-2.565863
H	-1.926737	1.368736	-3.580277
C	-3.542398	1.245756	-2.146972
C	-4.168134	-0.078683	-2.571999
H	-3.430223	-0.612357	-3.188816
H	-1.904318	-1.248932	1.150382
H	-2.010713	-2.181613	-0.423314
C	-3.521290	-0.631885	-0.137029
C	-2.337894	-1.320839	0.156280
B	-4.008229	0.418719	0.892371
O	-3.342931	0.729756	2.067522
O	-5.146937	1.178705	0.718178
C	-3.907412	1.981054	2.551280
C	-5.354621	1.943926	1.936496
C	-4.466933	-0.963463	-1.319707
Pd	-1.815508	0.542733	-0.964789
H	-5.474551	-0.697162	-0.978411
C	-4.489198	-2.454123	-1.694273
H	-5.155796	-2.588017	-2.555536
H	-3.490923	-2.766048	-2.028195
H	-4.172795	1.858457	-1.503033
C	-5.934460	3.303063	1.562495
H	-6.933070	3.172276	1.134794
H	-6.024423	3.943466	2.446173
H	-5.315308	3.813199	0.822141
C	-3.044044	3.113274	1.981167
H	-3.391086	4.093551	2.321037
H	-2.011100	2.980332	2.311608
H	-3.046399	3.095096	0.887239
C	-3.852208	1.986442	4.074218
H	-2.809269	2.002204	4.405509
H	-4.347114	2.876459	4.476533
H	-4.326457	1.099652	4.498717
C	-6.351381	1.153881	2.790881
H	-5.936288	0.184311	3.079484
H	-6.628962	1.700129	3.697372
H	-7.254043	0.972041	2.202115
C	-4.952672	-3.398797	-0.563729
H	-4.930190	-4.428413	-0.942062
H	-4.237206	-3.349385	0.262994
C	-6.339368	-3.077983	-0.050477
C	-7.475847	-3.435712	-0.790847
C	-6.522253	-2.378450	1.150170
C	-8.756738	-3.107436	-0.345150
C	-7.802172	-2.050162	1.602607
C	-8.924593	-2.412981	0.855900
H	-7.352073	-3.979071	-1.725109
H	-5.650758	-2.086438	1.730863
H	-9.624326	-3.396600	-0.932500
H	-7.920818	-1.510043	2.537599
H	-9.921209	-2.159183	1.206411
C	-5.425415	0.164929	-3.424153
H	-6.162901	0.748505	-2.860233
H	-5.179391	0.719551	-4.336073
H	-5.902722	-0.775704	-3.720597

3-(RS)-Pd_Real-Me

	Value
Charge	0
Electronic Energy, BS1 (a.u.)	-3193.941029
Thermal and entropic correction, BS1 (a.u.)	0.911960
Electronic Energy, BS2 (a.u.)	-3194.850200

Molecular Geometry in Cartesian Coordinates

P	-0.456335	-0.214868	-0.694460
P	-5.337815	1.248411	-0.210488
C	-1.206508	-1.089914	-2.147716
C	-1.115487	-1.207555	0.712352
C	-1.230470	1.477342	-0.780023
C	-5.536305	-0.547198	-0.630585
C	-4.251829	1.134596	1.301793
C	-6.976287	1.678830	0.530918
C	-0.920527	-2.461827	-2.274142
C	-1.912105	-0.456101	-3.178937
C	-0.244042	-1.489545	1.775611
C	-2.416200	-1.733765	0.736639
C	-2.257445	2.064761	-0.007515
C	-0.643824	2.248715	-1.808034
C	-5.052623	-0.965028	-1.878322
C	-6.073564	-1.510978	0.240527
C	-4.753077	0.668094	2.530258
C	-2.876911	1.449083	1.207857
C	-7.069005	2.731793	1.459274
C	-8.165918	1.086353	0.075551
H	-0.364974	-2.969264	-1.489804
C	-1.335342	-3.177901	-3.394525
C	-2.314242	-1.172491	-4.311239
H	-2.155627	0.598077	-3.107595
C	-0.678324	-2.254738	2.860397
H	0.779263	-1.129932	1.747682
C	-2.847571	-2.493044	1.822888
H	-3.095840	-1.548145	-0.086079
C	-2.665693	3.378474	-0.312981
H	0.168221	1.815689	-2.386511
C	-1.072413	3.537528	-2.105192
H	-4.632938	-0.234601	-2.562118
C	-5.074755	-2.315092	-2.240554
H	-6.464016	-1.210087	1.206769
C	-6.109873	-2.855854	-0.124538
H	-5.810519	0.445249	2.625202
C	-3.925908	0.497821	3.636383
C	-2.055526	1.277767	2.334444
H	-6.164995	3.207424	1.829641
C	-8.308627	3.162899	1.931063
H	-8.126145	0.275067	-0.644889
C	-9.406504	1.521059	0.546938
H	-1.108797	-4.238020	-3.469444
C	-2.029701	-2.533291	-4.424057
H	-2.858651	-0.661368	-5.100847
C	-1.981798	-2.751996	2.889425
H	0.006110	-2.460750	3.678959
H	-3.863116	-2.875931	1.831598
C	-2.098230	4.108663	-1.349933
H	-0.600340	4.093144	-2.910389
H	-4.664919	-2.619539	-3.198085
C	-5.600416	-3.262162	-1.362979
H	-6.527854	-3.589709	0.559484
C	-2.566074	0.797871	3.535446
H	-4.339565	0.129895	4.571159
H	-8.355673	3.971346	2.655768
C	-9.483847	2.558400	1.477605
H	-10.313605	1.043138	0.186642
H	-2.342089	-3.087734	-5.304558

H	-2.322264	-3.343736	3.734943
H	-2.441485	5.118419	-1.555142
H	-5.614874	-4.312874	-1.638765
H	-1.907006	0.662798	4.387689
H	-10.449321	2.893467	1.845963
H	-1.002106	1.527459	2.253180
H	-3.442924	3.827946	0.297665
C	2.526155	-0.978273	-2.850458
H	1.655697	-0.918092	-3.497269
H	2.902774	-1.978761	-2.643706
C	3.277610	0.134074	-2.540285
C	4.688924	0.127262	-1.970877
H	2.225174	0.738671	1.852703
H	2.509849	2.173435	0.755990
C	3.725881	0.402406	0.333942
C	2.677719	1.114887	0.939227
B	3.969785	-1.034981	0.871092
O	3.170899	-1.650036	1.821834
O	4.994397	-1.856159	0.444644
C	3.495294	-3.067719	1.792855
C	4.979777	-3.048458	1.277356
C	4.755601	0.977498	-0.665149
Pd	1.884208	-0.061470	-0.788374
H	5.753523	0.801843	-0.240112
C	4.628156	2.491488	-0.903532
H	5.226062	2.771569	-1.777116
H	3.589893	2.751787	-1.143988
H	2.951383	1.096202	-2.943000
H	4.918986	-0.900325	-1.674925
C	5.383229	-4.242326	0.420106
H	6.424209	-4.133234	0.101077
H	5.297980	-5.174695	0.987992
H	4.763435	-4.319544	-0.475233
C	2.526194	-3.729708	0.804693
H	2.684595	-4.810892	0.749260
H	1.500706	-3.546203	1.133783
H	2.635246	-3.302538	-0.196643
C	3.303081	-3.643476	3.190642
H	2.242576	-3.613425	3.458327
H	3.630731	-4.687650	3.227411
H	3.857670	-3.075579	3.940019
C	6.001931	-2.818338	2.395356
H	5.715801	-1.965705	3.017986
H	6.107339	-3.699680	3.035073
H	6.974866	-2.598008	1.946968
C	5.109893	3.362213	0.282457
H	4.909633	4.413829	0.041645
H	4.528856	3.125939	1.178446
C	6.583233	3.184743	0.579146
C	7.553573	3.774104	-0.244994
C	7.016051	2.388728	1.648872
C	8.914318	3.575274	-0.008726
C	8.376998	2.185386	1.889475
C	9.331421	2.778364	1.060954
H	7.235700	4.395440	-1.079458
H	6.276668	1.921605	2.295044
H	9.649848	4.043936	-0.657314
H	8.690247	1.563614	2.724016
H	10.390580	2.622928	1.246810
C	5.716720	0.534653	-3.040906
H	6.719405	0.624588	-2.607507
H	5.756344	-0.217832	-3.835924
H	5.468921	1.492169	-3.511846

12. References

1. J. Kuang, S. Ma. *J. Org. Chem.* **2009**, *74*, 1763.
2. M. L. Cooke, K. Xu, B. Breit, *Angew. Chem. Int. Ed.* **2012**, *51*, 10876.
3. R. Y. Liu, Y. Yang, S. L. Buchwald, *Angew. Chem. Int. Ed.* **2016**, *55*, 14077.
4. Z. Liu, B. Breit, *Org. Lett.* **2018**, *20*, 300.
5. Z.-K. Liu, Y. Yang, Z.-P. Zhan, *J. Org. Chem.* **2022**, *87*, 1589-1597.
6. M. Piñeiro-Suarez, A. Álvarez-Constantino, M. Fañanás-Mastral, *ACS Catal.* **2023**, *13*, 5578.
7. T. Kippo, T. Fukuyama, I. Ryu, *Org. Lett.* **2011**, *13*, 3864-3867.
8. Q. Yuan, K. Yao, D. Liu, W. Zhang, *Chem. Commun*. **2015**, *51*, 11834.
9. a) Y. Lee, J. Park, S. H. Cho, *Angew. Chem. Int. Ed.* **2018**, *57*, 12930. b) M. Han, M. Yang, R. Wu, Y. Li, T. Jia, Y. Gao, H.-L. Ni, P. Hu, B.-Q. Wang, P. Cao, *J. Am. Chem. Soc.* **2020**, *142*, 13398.
10. M. Hayashi, L. E. Brown, J. A. Porco, *Eur. J. Org. Chem.* **2016**, *2016*, 4800-4804.
11. D. J. Weix, D. Marković, M. Ueda, J. F. Hartwig, *Org. Lett.* **2009**, *11*, 2944-2947.
12. X.-D. Bai, Q.-F. Zhang, Y. He, *Chem. Commun.* **2019**, *55*, 5547-5550.
13. J. Štambaský, A. V. Malkov, P. Kočovský, *Collect. Czechoslov. Chem. Commun.* **2008**, *73*, 705-732.
14. J. Zheng, C. Nopper, R. Bibi, A. Nikbakht, F. Bauer, B. Breit, *ACS Catal.* **2022**, *12*, 5949.
15. Q. H. Pham, A. J. Tague, C. Richardson, C. J. T. Hyland, S. G. Pyne, *Chem. Sci.* **2021**, *12*, 12695-12703.
16. A. R. Haight, E. J. Stoner, M. J. Peterson, V. K. Grover, *J. Org. Chem.* **2003**, *68*, 8092-8096.
17. C. Li, J. Xing, J. Zhao, P. Huynh, W. Zhang, P. Jiang, Y. J. Zhang, *Org. Lett.* **2012**, *14*, 390-393.
18. T. Jia, P. Cao, B. Wang, Y. Lou, X. Yin, M. Wang, J. Liao, *J. Am. Chem. Soc.* **2015**, *137*, 13760.
19. J. Tsuji, K. Sato, H. Okumoto, *J. Org. Chem.* **1984**, *49*, 1341-1344.
20. M. J. Frisch, G. W. Trucks, H. B. Schlegel, G. E. Scuseria, M. A. Robb, J. R. Cheeseman, G. Scalmani, V. Barone, B. Mennucci, G. A. Petersson, H. Nakatsuji, M. Caricato, X. Li, H. P. Hratchian, A. F. Izmaylov, J. Bloino, G. Zheng, J. L. Sonnenberg, M. Hada, M. Ehara, K. Toyota, R. Fukuda, J. Hasegawa, M. Ishida, T. Nakajima, Y. Honda, O. Kitao, H. Nakai, T. Vreven, J. A. Montgomery, Jr., J. E. Peralta, F. Ogliaro, M. Bearpark, J. J. Heyd, E. Brothers, K. N. Kudin, V. N. Staroverov, T. Keith, R. Kobayashi, J. Normand, K. Raghavachari, A. Rendell, J. C. Burant, S. S. Iyengar, J. Tomasi, M. Cossi, N. Rega, J. M. Millam, M. Klene, J. E. Knox, J. B. Cross, V. Bakken, C. Adamo, J. Jaramillo, R. Gomperts, R. E. Stratmann, O. Yazyev, A. J. Austin, R. Cammi, C. Pomelli, J. W. Ochterski, R. L. Martin, K. Morokuma, V. G. Zakrzewski, G. A. Voth, P. Salvador, J. J. Dannenberg, S. Dapprich, A. D. Daniels, Ö. Farkas, J. B. Foresman, J. V. Ortiz, J. Cioslowski, D. J. Fox, *Gaussian 09, revision D.01*, Gaussian, Inc., Wallingford, CT, **2010**.
21. A. V. Marenich, C. J. Cramer, D. G. Truhlar, J. Phys. Chem. B 2009, 113, 6378-6396.
22. a) C. Lee, W. Yang, R. G. Parr, *Phys. Rev. B* **1988**, *37*, 785-789; b) A. D. Becke, *J. Phys. Chem.* **1993**, *98*, 5648-5652.
23. S. Grimme, J. Antony, S. Ehrlich, H. Krieg, *J. Phys. Chem.* **2010**, *132*, 154104.
24. a) M. M. Francl, W. J. Pietro, W. J. Hehre, J. S. Binkley, M. S. Gordon, D. J. DeFrees, J. A. Pople, *J. Phys. Chem.* **1982**, *77*, 3654-3665; b) W. J. Hehre, R. Ditchfield, J. A. Pople, *J. Phys. Chem.* **1972**, *56*, 2257-2261.
25. D. Andrae, U. Häußermann, M. Dolg, H. Stoll, H. Preuß, *Theor. Chim. Acta* **1990**, *77*, 123-141.
26. A. Ehlers, M. Böhme, S. Dapprich, A. Gobbi, A. Höllwarth, V. Jonas, K. Köhler, R. Stegmann, A. Veldkamp, G. Frenking, *Chem. Phys. Lett.* **1993**, *208*, 111-114.
27. a) F. Weigend, *Phys. Chem. Chem. Phys.* **2006**, *8*, 1057-1065; b) F. Weigend, R. Ahlrichs, *Phys. Chem. Chem. Phys.* **2005**, *7*, 3297-3305.
28. V. S. Bryantsev, M. S. Diallo, W. A. Goddard III, *J. Phys. Chem. B* **2008**, *112*, 9709-9719.
